# Development of Sheep Duodenum Intestinal Organoids and Implementation of High-Throughput Screening Platform for Veterinary Applications

**DOI:** 10.3390/ijms26073452

**Published:** 2025-04-07

**Authors:** Giulio Galli, Estela Melcón-Fernández, María Gracia de Garnica García, Beatriz Martínez-Fernández, Mahsa Dehnavi, Sonia Andrés, Yolanda Pérez-Pertejo, Rosa M. Reguera, Carlos García-Estrada, María Martínez-Valladares, Rafael Balaña-Fouce

**Affiliations:** 1 Departamento de Ciencias Biomédicas, Facultad de Veterinaria, Universidad de León, Campus de Vegazana s/n, 24007 León, Spain; ggal@unileon.es (G.G.); emelf@unileon.es (E.M.-F.); myperp@unileon.es (Y.P.-P.); rmregt@unileon.es (R.M.R.); cgare@unileon.es (C.G.-E.); 2 MicrosVeterinaria, C/Profesor Pedro Cármenes, Campus de Vegazana, 24007 León, Spaininfo@microsvet.es (B.M.-F.); 3 Instituto de Ganadería de Montaña, CSIC-Universidad de León, Finca Marzanas s/n, Grulleros, 24346 León, Spain; mahsa.d@csic.es (M.D.); sonia.andres@eae.csic.es (S.A.); mmarva@csic.es (M.M.-V.); 4 Instituto de Biomedicina (IBIOMED), Universidad de León, Campus de Vegazana s/n, 24007 León, Spain

**Keywords:** sheep, duodenum, intestinal organoid, 3D culture, high-throughput screening (HTS), veterinary pharmacology, drug discovery, toxicity test

## Abstract

New therapeutic molecules for farm animals are needed to address worldwide problems in the food industry, like the rise of resistance among ruminant parasites and pathogenic microbes. Since in vivo testing would involve an excessive number of animals, with consequent ethical and economic issues, the generation of sheep intestinal organoids represents a promising close-to-reality in vitro model for veterinary drug development; however, the characterization and application of such organoids remain limited. In this study, ovine intestinal organoids were generated from adult LGR5+ stem cells from the intestinal crypts of freshly slaughtered lambs, and developed in an in vitro culture system. Morphological analysis via brightfield microscopy and immunocytochemical staining revealed a pseudostratified epithelium with multiple cell types, and distinct apical–basal polarity, while RNA sequencing validated the preservation of the physiological characteristics of the original organ. The development and characterization of a robust and reproducible protocol for culturing sheep duodenum intestinal organoids in a high-throughput screening (HTS) compatible format demonstrated reliability in HTS applications, with Z’-factor tests indicating robust assay performance. Dose–response studies using pre-identified compounds showed comparable pharmacodynamic profiles between mouse and sheep organoids. These findings establish sheep intestinal organoids as an innovative tool for veterinary pharmacology and toxicology, offering a cost-effective and sustainable platform to address challenges such as drug resistance and improve livestock health.

## 1. Introduction

The development of new veterinary medicines, particularly for livestock, is a critical need in the face of growing challenges such as drug resistance, zoonotic diseases, and the demand for sustainable agriculture [1,2,3]. Worldwide, sheep account for 1.3% of total milk production and produce 9 million tons of meat per year, and together with goats, they account for 20.8% of dairy products [4], which places the ovine industry as a pivotal source of meat, wool, and milk. The growing demand for these products has led to an increased use of veterinary medicines in the aim of sustaining this industry. However, in a context where the number of new molecules has halved since the 1990s, despite global R&D spending having doubled, the development of new drugs for species like sheep represents an attractive market [5].

Drug resistance in sheep due to the widespread use of anthelmintic drugs represents a serious challenge, resulting in significant economic losses that restrict the development of the sheep industry in pastoral areas. It has been reported that the annual cost of gastrointestinal nematode infections resistant to macrocyclic lactones is estimated to be EUR 38 million [6]. Gastrointestinal nematodes, including *Ostertagia ostertagi*, *Cooperia oncophora*, *Teladorsagia circumcincta*, and *Haemonchus contortus*, are responsible for production losses on 10–50% of farms worldwide. These parasites cause direct tissue damage, divert the host’s energy and protein resources from production to immunological defence, and reduce feed intake, which is the major mechanism underlying subclinical production impacts. In sheep, these infections can lead to reduced weight gain (10–47%) and diminished wool growth (0–21%). Conversely, anthelmintic treatment has demonstrated practical benefits, including increases in whole-lactation milk production of 9–40% [7]. This situation, along with a constant increase in helminthic and microbial resistance, makes the discovery of new leads in pharmaceutical research necessary [1,2,5,8]. The gut plays a crucial role in the metabolism of drugs, serving as the primary site for drug absorption and initial biotransformation. The enterocytes lining the intestinal wall are equipped with a variety of enzymes, particularly of the cytochrome P450 (CYP) superfamily, which can metabolize drugs before they enter systemic circulation. This process, known as the first-pass metabolism, significantly influences the bioavailability and therapeutic efficacy of orally administered medications, and is stronger in the duodenal tract. The interplay between drugs and gut enzymes can lead to the activation, inactivation, or even production of toxic metabolites, underscoring the importance of the gut in pharmacokinetics and pharmacodynamics [9].

Organoids represent a promising frontier in the study of gut–drug interactions, providing a three-dimensional (3D), physiologically relevant modeling system, derived from stem cells, that is well aligned with the three Rs principle for animal experimentation [10,11,12]. These miniaturized and simplified stem cell-derived versions of the intestine can reproduce much of the complexity and functionality of their in vivo counterparts, given their ability to generate multicellular, intestinal crypt-like structures that retain the same polarity and microanatomical distribution as found in vivo [13,14,15,16]. Organoids can propagate in vitro for a long term (>1 year) without undergoing significant genetic drift, and can replicate the phenotype of the individual donor [12]. Crucially, intestinal organoids express many drug-metabolizing enzymes of both phase I and II, like uridine diphosphate-glucuronosyltransferases, carboxylesterases, and CYPs, which are not as expressed in monolayer cultures of immortalized gut cells such as CaCo-2 [11,17,18,19]. So far, major drawbacks of organoid research include a higher variability compared to 2D models and a lack of crucial components of the immunological, nervous, and cardiovascular systems [12,20,21,22].

The duodenum, in particular, has an enhanced role in drug metabolism. This region of the small intestine exhibits high enzymatic activity, with rich expression of metabolizing enzymes such as various CYP isoforms, which play a pivotal role in the initial biotransformation of drugs. The duodenal epithelium’s rapid cellular turnover and constant exposure to ingested compounds make it a critical site for understanding first-pass effects, thereby providing a more accurate reflection of in vivo drug metabolism [23,24].

Given the advantages indicated above, sheep intestinal organoids have recently garnered attention due to their potential applications in veterinary medicine and agricultural science. Despite this growing interest, the literature on sheep intestinal organoids remains relatively sparse compared to that on human and murine organoids. Initial studies have demonstrated the feasibility of culturing sheep organoids using protocols adapted from other species [25,26,27]. Many existing models exhibit incomplete maturation, failing to fully recapitulate the complex architecture and functionality of the native intestinal epithelium. This limited model results in a suboptimal representation of key physiological processes, such as nutrient absorption, barrier integrity, and metabolic activity. Additionally, there is inadequate functional validation of these models, with few studies comprehensively assessing parameters like drug metabolism, enzyme expression, and response to pathogenic challenges. The variability in organoid differentiation and the absence of standardized protocols further constrain reproducibility and broader application in translational research [12,13,14,20,21,25]. Also, the use of 3D cultures in drug discovery and development has been hampered by difficulties in achieving a high throughput, automation, and integration in existing laboratory pipelines, as well as by relatively high associated costs [15].

In this study, a robust and cost-effective method for culturing sheep duodenum intestinal organoids has been developed from tissues obtained from slaughterhouses. Using this type of organoid, a reliable system for growing sheep organoids in 384-well plates that is suitable for HTS assays has been established. This platform enables the screening of large libraries of compounds for their efficacy and toxicity, providing a valuable resource for veterinary drug development.

## 2. Results

### 2.1. Development and Morphological Characterization of Sheep Duodenum Intestinal Organoids

**Figure 1:**
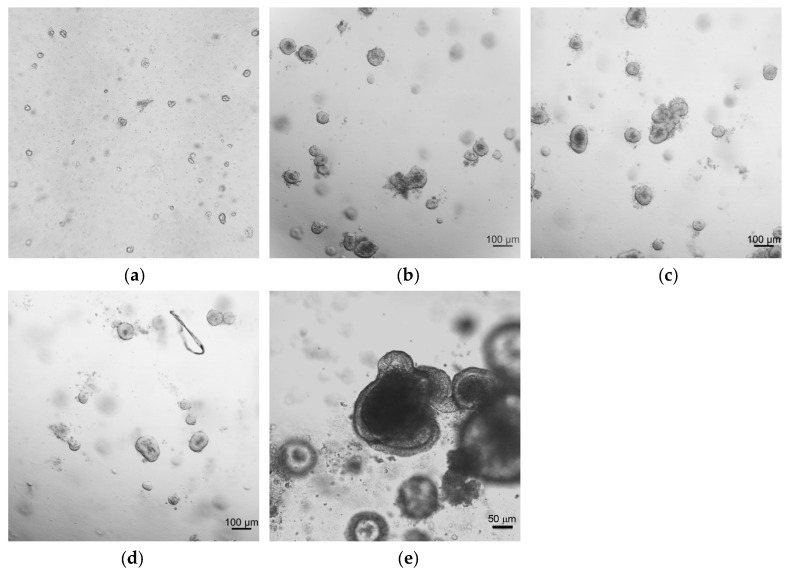
Representative images of brightfield microscopy of duodenum intestinal sheep organoids: (**a**) at 0 h (crypts immediately after extraction); (**b**) after 3 days in culture; (**c**) after 5 days in culture; (**d**) after 7 days in culture; and (**e**) after 10 days in culture. Pictures were taken with Zeiss confocal Observer Z1 microscope, except for crypts in panel (**a**), which were observed with Hund Wetzlar Wilovert S inverted microscope with 4× magnification.

Our research group previously developed mouse intestinal organoids to assess cytotoxicity during the characterization of compounds with antileishmanial [28,29] and anthelmintic [30] activity. Based on this experience, it was decided to generate intestinal duodenum organoids from young sheep to implement an HTS platform for drug discovery purposes focused on this species. Hence, sheep duodenum from a local slaughterhouse was collected and processed as indicated in Materials and Methods. After the growth and culture of stem cell-rich intestinal crypts, organoids were visualized to confirm their structure, and brightfield images were taken throughout the 10 days after seeding (Figure 1).

After 24 h, small and rounded organoids were formed, and underwent budding starting from the fifth day in culture. After the seventh day, if not subcultured, the organoid wall ruptured under the pressure of the lumen’s waste. In the duodenum intestinal organoid, three layers can be distinguished: a central lumen which blackened throughout the culture time due to cells shredding, a wall made of organoid cells, and a fine line surrounding the organoid, likely corresponding to the basal membrane (Figure 1e). This architecture suggests that the apical side is in the inner part of the organoid facing the lumen, whereas the basal side is in contact with the matrix. Overall, this morphology is in line with the previous literature on intestinal organoids [26,27,31,32,33].

**Figure 2:**
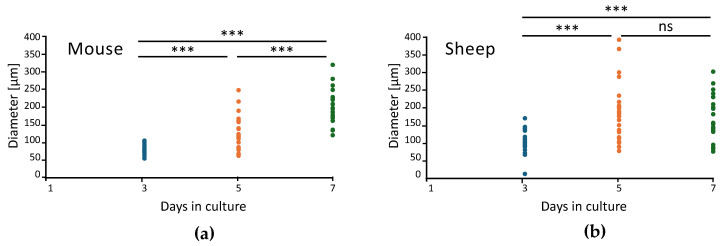
Diameter (μm) of 24 organoids from (**a**) mouse and (**b**) sheep at 3, 5, and 7 days in culture. ns, nonsignificant; *** *p* < 0.001.

After comparison of 24 organoids from mouse and sheep, it was found that sheep organoids exhibited rapid growth up to day 5, but with higher variability, which was subsequently reduced up to day 7 (Figure 2, Table S1).

As it was previously observed with organoids developed from the sheep gastrointestinal tract [27], the organoids generated in this work demonstrated their potential to be stored in liquid nitrogen after two passages (21–30 days) of in vitro culture and to resuscitate 6 months later, showing, upon resuscitation, a similar morphology to the parental organoids.

**Figure 3:**
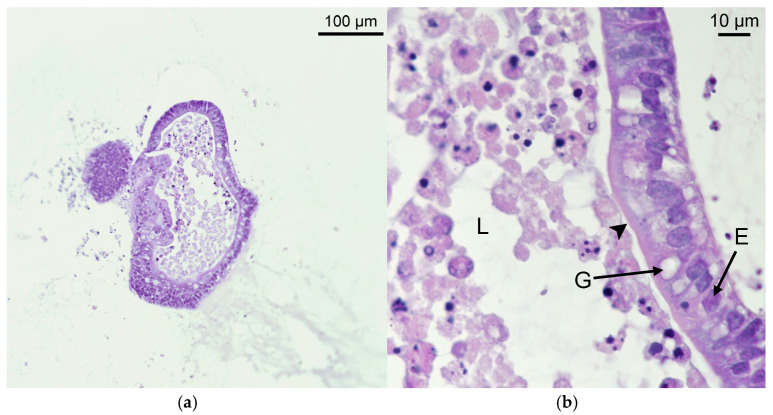
Bright field microscopy images of duodenum intestinal sheep organoid stained with H&E: (**a**) representative image of duodenum sheep organoids after 7 days in culture; (**b**) detail of organoid wall. Lumen with apoptotic cells (L), goblet cells (G), enterocytes (E), and microvilli brush (black arrowhead). Pictures were taken with Nikon Eclipse Ni microscope.

To further confirm the microanatomy, a histological preparation with haematoxylin and eosin (H&E) was made (Figure 3). The organoid wall is composed of a pseudostratified epithelium of cylindrical cells, with many mucous-reach cells, suggesting the presence of enterocytes and goblet cells. A microvilli brush can be spotted on the internal side, confirming the polarity of the structure. The lumen shows a lot of cell debris and apoptotic cells, proving that, like in vivo, cell shredding also occurs in vitro.

### 2.2. Immunocytochemistry Reveals Key Structures of Sheep Duodenum Intestinal Organoids

**Figure 4:**
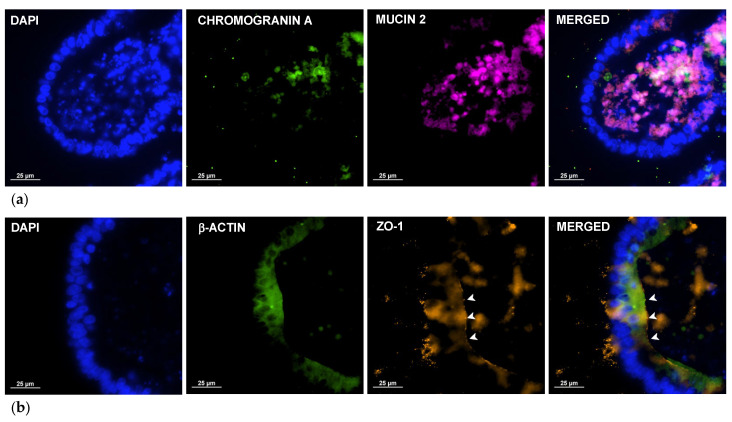
Immunofluorescence confocal microscopy of sheep duodenum intestinal organoids at 7 days of in vitro culture: (**a**) representative image of duodenum sheep organoids hybridized with anti-chromogranin A (green) and anti-mucin 2 (magenta); (**b**) representative image of duodenum sheep organoids hybridized with anti-β-actin (green) and anti-ZO-1 (orange). White arrowheads represent ZO-1 protein on apical side of organoid. Pictures were taken with Nikon Eclipse Ni microscope. Scale bars represent 25 μm.

Immunofluorescence staining (Figure 4) was used to confirm the presence of different cell types in the organoid and the polarity of the structure. Detection of chromogranin A (Figure 4a, green staining), a marker for enteroendocrine cells, confirmed the presence of the rare cell type which is responsible for the production of signals to control digestive enzyme secretion, bowel movements, and metabolism under neuroendocrine stimulation. On the other hand, detection of mucin 2 (Figure 4a, magenta staining), produced and secreted by goblet cells, revealed the presence of abundant secretion inside the organoid lumen and the presence of some cells in the epithelial wall. The presence of a β-actin boundary (Figure 4b, green staining), together with the detection of zonula occludens-1 (ZO-1) (Figure 4b, orange staining), a tight-junction protein naturally present on the apical side of intestinal cells, confirmed the polarity of orientation of the cells, with the apical side facing the internal lumen and the basal side in contact with the matrix.

### 2.3. Transcriptional Analysis of Sheep Duodenum Intestinal Organoids and Expression of Cell- and Tissue-Specific Genes in Intestinal Organoids and Tissue

**Figure 5:**
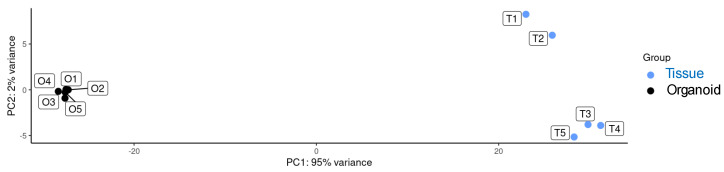
Principal component analysis of RNA-seq expression, visualizing samples of sheep duodenum tissue (T1–T5, blue dots) and duodenum intestinal organoids (O1–O5, black dots).

RNA-seq analysis was carried out to compare gene expression profiles from ovine duodenum tissue (n = 5) and ovine duodenum organoids (grown for 21 days after one subculture, five replicates), the latter derived from the same tissue. The RNA sequencing quality control confirmed appropriate fragment size, and sequencing quality was assessed with FastQC, providing metrics such as read counts, GC content, and Q20/Q30 scores, confirming the adequacy of these parameters. Post-mapping quality control was performed using HTSeq to quantify reads mapped to each gene, and most samples had over 90% of reads successfully aligned. Expression profiles (Table S2) were compared by principal component analysis (Figure 5), which resulted in statistically significant clusters (95% confidence intervals) for each sample type.

**Figure 6:**
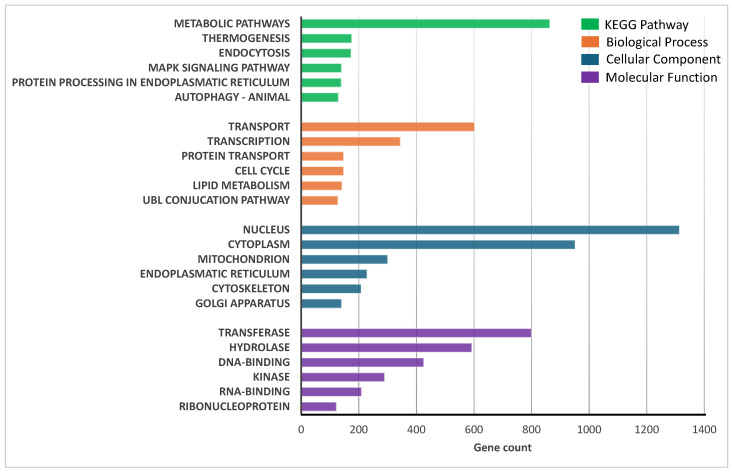
Functional enrichment analysis of co-expressed genes in the duodenum intestinal organoids and intestinal tissue. The six most represented pathways in each category (KEGG pathway, biological process, cellular compartment, and molecular function) are represented with dotted boxes.

The similarity between sheep intestinal organoids and intestinal tissues was confirmed after analysis of the transcriptional profile, thus revealing a total of 8917 genes co-expressed in both intestinal organoids and tissues (1906 were specific to the intestinal tissue and 807 were specific to intestinal organoids). Functional enrichment analysis of co-expressed genes was performed using the DAVID free software (v2024q4) to identify the six most significant metabolic pathways, based on the Kyoto Encyclopedia of Genes and Genomes (KEGG), as well as biological, cellular, and molecular categories (Figure 6). The results indicate that both the organoids and duodenal tissue exhibit high expression of genes associated with transport and transcription. The nucleus was identified as the most active cellular site, while transferases and hydrolases were the most abundantly transcribed enzyme classes.

**Figure 7:**
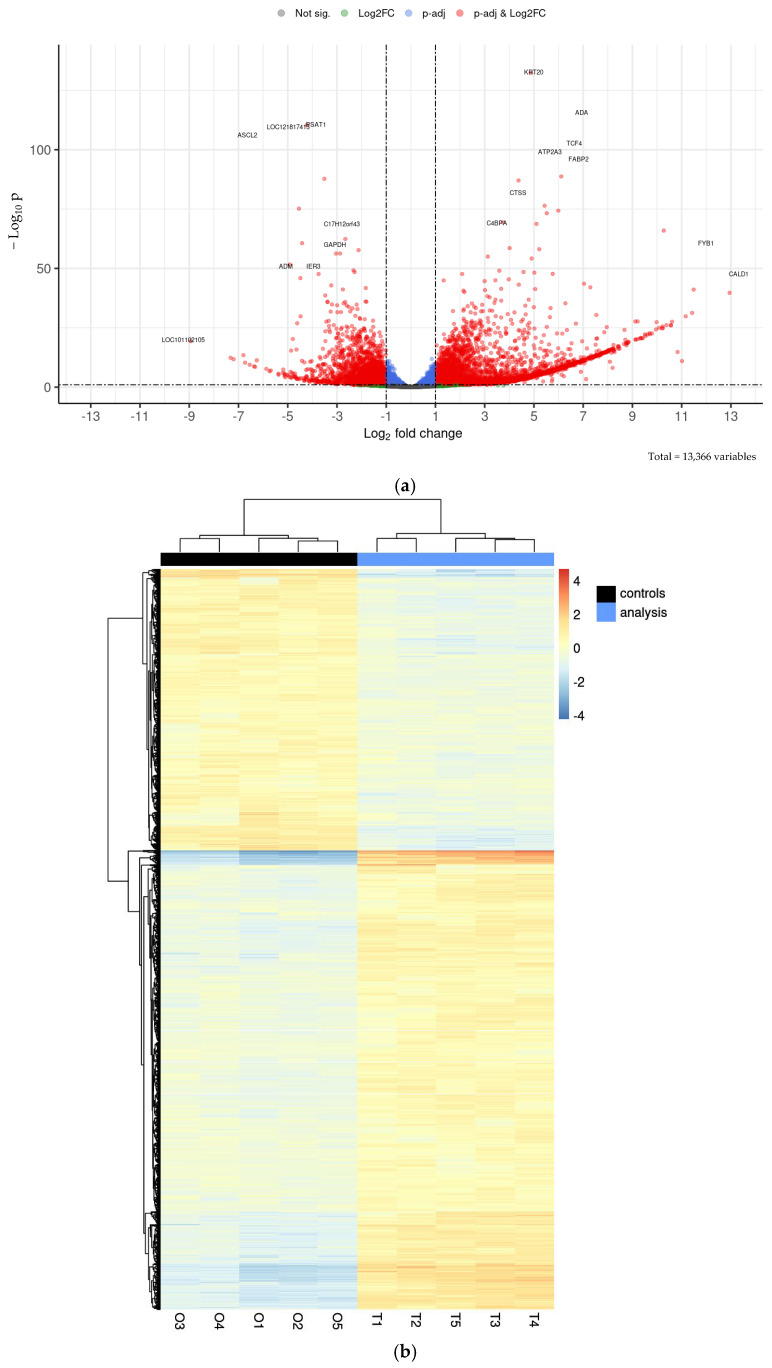
Differential expression of duodenum tissue and duodenum organoids: (**a**) volcano plot showing comparison of expressed genes between sheep duodenum intestinal tissue and duodenum intestinal organoids. Plot represents statistical significance (*p*-value, Y axis) versus magnitude of change (fold change, X axis). Grey dots represent neither statistical significance nor differences in expression. Blue dots represent statistical significance, but not differences in expression. Green dots represent no statistical significance, but changes in expression. Red dots represent both statistical significance and differences in expression. (**b**) Heatmap displaying most significantly overexpressed and repressed genes between sheep duodenum intestinal tissue (T1–T5) and duodenum intestinal organoids (O1–O5). Applied filters are Log2FC < −1 or >1, and q-value < 0.1. Colours indicate level of expression from lower (blue) to higher (red). Dendrograms indicate similarity between samples.

Differential expression analysis with DESeq2 identified 3496 overexpressed genes and 2140 repressed genes in the intestinal tissue compared to the organoids, which correspond to 26.2% and 16.0% of the 13,366 transcripts, respectively. These results are visualized in a volcano plot (Figure 7a) and a heatmap (Figure 7b), showing distinct expression patterns based on significant fold changes and q-values.

The most upregulated genes in the intestinal tissue compared to the organoids were KRT20, TCF4, OLFM4, ADA, FABP2, and LOC114117878. The first one encodes keratin-20, a cytoskeletal protein expressed in differentiated intestinal epithelial cells, playing a key role in maintaining structural integrity [34]. TCF4, a transcription factor, is essential for the WNT signalling pathway and intestinal epithelial homeostasis, particularly in stem cell renewal and proliferation [35]. OLFM4 is a marker of intestinal stem cells, associated with their self-renewal capacity and defence against microbial infections [36]. ADA encodes adenosine deaminase, involved in purine metabolism and modulating local immune responses in the gut [37]. FABP2, or fatty acid-binding protein 2, is highly expressed in enterocytes, and facilitates fatty acid absorption [38]. Finally, LOC114117878, an uncharacterized gene, may represent a novel factor with potential relevance in organoid-specific processes, requiring further study. Conversely, six genes were markedly downregulated: PSAT1, LOC121817413, ASCL2, C17H12orf43, PRTFDC1, and RPL37A. The first one is involved in serine biosynthesis, a pathway critical for rapidly proliferating cells, such as those in native intestinal tissue [39]. ASCL2 is a transcription factor pivotal for intestinal stem cell maintenance [40]. RPL37A, a ribosomal protein, plays a fundamental role in protein synthesis and cell growth [41]. PRTFDC1, linked to purine metabolism [42], and C17H12orf43, a poorly characterized gene, may contribute to metabolic and regulatory differences between organoids and tissue. Lastly, LOC121817413, another uncharacterized gene, highlights the need for further research into its function in intestinal biology. Overall, the organoids exhibited lower expression levels of genes associated with innate immunity, probably reflecting the absence of immune stimulation due to their culture in a sterile environment. Also, lower expression of cell maturation markers and upregulation of genes related to tissue renewal indicate an enhanced cell proliferation cycle in organoids compared to the ex vivo tissue (Table S2). These results are aligned with a recent study by Wang and co-authors [32], indicating that organoids retain a stem-like phenotype.

### 2.4. Development of HTS for Drug Discovery Purposes Based on Sheep Duodenum Organoids

To determine applications for sheep duodenum organoids, an HTS to assess cytotoxicity during screening of compound libraries was developed in a search for anthelmintic molecules.

First, a Z’-factor test was performed with four 384-well plates, using duodenum organoids on the seventh day in culture. Hydrogen peroxide (0.03% *v*/*v*) was added as a death control, while 0.2% DMSO (*v*/*v*) was added as a viability control. Cell viability was assessed using the alamarBlue™ HS staining reagent (Invitrogen™, Thermo Fisher Scientific™, Waltham, MA, USA). On average, the Z’-factor obtained with the four replicates was 0.5 (Table 1), which is remarkable considering that organoids have inherently higher variability compared to classical 2D models. Therefore, sheep duodenum organoids were considered suitable for use in HTS.

**Figure 8:**
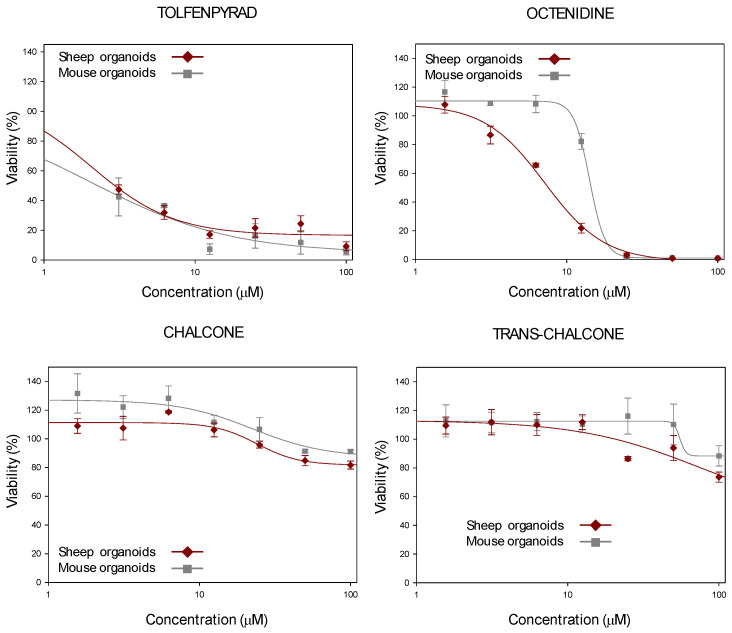
Dose–response curves adjusted with the Sigma Plot 10.1 statistical package, showing the cytotoxicity of tolfenpyrad, octenidine, chalcone, and trans-chalcone on sheep intestinal organoids (red line) and mouse intestinal organoids (grey line). The above-mentioned compounds were tested at different concentrations, ranging from 1.6 μM and 100 μM. Dose–response curves were created, plotting the percentage of viability obtained with the alamarBlue™ HS staining method (Invitrogen™, Thermo Fisher Scientific™, USA) from organoids after 7 days of incubation in the presence of the anthelmintic drugs. Hydrogen peroxide (0.03% *v*/*v*) was used as a death control, while 0.2% DMSO *v*/*v* served as a viability control. The data represent the mean ± standard deviation of two independent experiments carried out in triplicate.

As proof of concept, four anthelmintic hits (tolfenpyrad, octenidine, chalcone, and trans-chalcone), which were previously identified in a previous study [30], were tested using this new HTS platform and using mouse intestinal organoids. As a result, dose–response curves were obtained, testing different concentrations of the above-mentioned molecules in the HTS containing sheep duodenum organoids, and were compared to data provided by mouse intestinal organoids (Figure 8). Calculation of the cytotoxic concentration 50% (CC_50_) values for both types of organoids (Table 2) showed no significant differences between them (two-sided paired Student’s *t*-test, *p* = 0.572).

## 3. Discussion

Sheep play a significant role in global agriculture by producing essential products, such as wool, meat, and milk, while sustaining rural livelihoods [43]. However, sheep farming faces several challenges, with parasitic infections by gastrointestinal nematodes being among the most critical ones. These infections can adversely affect the health, growth, and reproduction of sheep, leading to decreased productivity and, in severe cases, death [44]. Consequently, the discovery of novel anthelmintic compounds is crucial for ensuring the sustainability of sheep farming and safeguarding animal welfare.

In this regard, organoids represent a breakthrough in drug discovery by providing physiologically relevant, accurate, efficient, and robust models for testing drugs, studying diseases, developing personalized treatments, and investigating the effects of pre- and probiotics on intestinal health [45]. Intestinal organoids have emerged as powerful tools in drug discovery, offering unique advantages for studying gastrointestinal diseases, drug absorption, and toxicity, as well as for testing both human and veterinary drugs. Despite these advantages, only a limited number of studies have reported the use of sheep intestinal organoids [25,26,27], even though sheep are frequently used as models for studying various gastrointestinal diseases, due to their similarities to humans in terms of digestion and gut function [46].

To implement the use of ovine intestinal organoids in drug discovery, organoids from the duodenal region of the sheep intestine were generated. The duodenum was selected as the source for organoids, due to its key role in gastrointestinal nematode infections and its importance in drug metabolism and absorption [9,47,48,49]. To the best of our knowledge, this is the first report of sheep intestinal organoids developed from the duodenum. Compared to mouse intestinal organoids, which were also implemented by our group [28,29,30], sheep intestinal crypts at day 0 exhibited a different morphology, being smaller in size and more rounded. After 24 h, small, rounded organoids formed, which grew and underwent budding starting from the fifth day in culture, albeit to a lesser extent than mouse or human differentiated organoids. These differences may be attributed to the distinct developmental and structural characteristics of the intestine among different species [12]. Microscopy analysis confirmed that the sheep duodenum organoids generated in this study recapitulated key structural and functional properties of sheep intestinal tissue, including cell polarity and a diverse cell population. The presence of abundant mucus and cell shedding suggests a metabolically active profile that closely resembles in vivo microphysiology, making the model likely capable of processing drugs. A comparative analysis of gene expression profiles between tissue and organoids revealed differences in the global transcriptome. These differences can be attributed to the complexity of ex vivo tissue, which is a vascularized structure comprising immune cells and intestinal flora mixed with digested material; these factors influence gene expression via paracrine and endocrine signals. A similar phenomenon was observed in a comparative analysis between ovine gastric and ileal organoids and their respective tissues by other authors [27]. Despite these differences, the overall features of the organoids support their status as one of the most physiologically representative in vitro cell culture systems available [16]. Similarly, the cell diversity, structure, and conserved expression of tissue-specific gene markers in duodenum-derived sheep organoids further validate them as a realistic alternative model for in vitro studies.

Furthermore, as a proof of concept for their potential use in drug discovery, these organoids were implemented in a 384-well plate format for HTS, an approach widely used in the biopharmaceutical industry with 2D cultures that is now expanding to 3D cultures [50,51]. To the best of our knowledge, this is the first assay using sheep intestinal organoids of any kind in a 384-well format. The suitability of this platform was assessed by calculating the Z’ factor, which, despite having a higher variability than classical 2D models, proved to be suitable for HTS. Four lead compounds were tested from a previous screening of several commercial libraries that had shown anthelmintic activity [30]. The comparison of dose–response curves between mouse and sheep intestinal organoids revealed similar CC_50_ values, confirming the assay’s ability to maintain specificity and sensitivity, although further tests with a larger set of compounds are necessary to validate these findings.

The integration of this HTS platform to assess safety within the sheep anthelmintic discovery strategy could facilitate the massive screening of chemical libraries against parasitic nematodes, which are responsible for significant production losses [52,53]. Beyond anthelmintic research, HTS paves the way for testing drug toxicity for other applications, as well as for assessing environmental contaminants that sheep might ingest [54]. Additionally, the higher expression of drug-metabolizing enzymes in these organoids can detect pro-drug and pro-toxic effects with greater precision compared to 2D cell models.

Nonetheless, there are limitations to both the HTS platform and the sheep duodenum organoids. The absence of immune, vascular, nervous, and mesenchymal components deprives epithelial cells of paracrine and endocrine signals that modulate immune responses and other functions. The lack of intestinal flora further removes an important aspect of drug metabolism from the HTS platform. In addition, the absence of peristaltic movement and mechanical stimuli alters cellular expression profiles, and a culture medium enriched in stem cell factors seems to drive organoids toward a more undifferentiated and less active state [55]. Furthermore, organoids are grown in an animal-derived matrix, which is richer in cytokine signals compared to synthetic matrices, but suffers from batch-to-batch variation and an undefined composition [56]. Finally, HTS also has inherent disadvantages, such as the inefficient removal of reagents from wells and difficulties in subsequent washing of cells and organoids [50,51,57,58].

In summary, despite its limitations, sheep duodenum organoids and the HTS platform enable rapid and cost-effective screening of hundreds to thousands of compounds, with improved translational relevance compared to 2D models. Future analyses should incorporate high-content screening and organ-on-a-chip technologies to further elucidate candidate compounds’ modes of action and ADMET properties, before advancing to animal clinical trials.

## 4. Materials and Methods

### 4.1. Preparation and Culture of Sheep Duodenum Intestinal Organoids and Mouse Intestinal Organoids

Sheep intestinal tissue was obtained from a local slaughterhouse and stored in cold PBS until processing. Mouse organoids were obtained from C57BL/6 mice housed in the facilities of University of León. All animal procedures were performed in compliance with Spanish and EU legislation (RD 53/2013 and 2010/63/EU), and were approved by the Ethics Committee of the University of León, project licence OEBA-ULE-010-2023.

The preparation and maintenance of sheep intestinal organoids were adapted from protocols published by Stemcell Technologies™ for mouse intestinal organoids (Intestinal Epithelial Organoid Culture with an IntestiCult™ Organoid Growth Medium (Mouse) technical bulletin, https://cdn.stemcell.com/media/files/techbulletin/TB28223-Intestinal_Epithelial_Organoid_Culture_with_IntestiCult_Organoid_Growth_Medium_(Mouse).pdf?_ga=1.255806536.1701332796.1451577632) (accessed on 6 September 2022)) [59], with some modifications. A segment of the small intestine was washed with ice-cold PBS, cut into approximately 2 mm pieces, and washed another 15–20 times with ice-cold PBS. The tissue pieces were then incubated with Gentle Cell Dissociation Reagent (GCDR, Stemcell Technologies™, Vancouver, BC, Canada) for 15 min on a rocking platform. Following incubation, the tissue pieces were resuspended in ice-cold PBS containing 0.1% BSA and precipitated by decantation. The supernatant was collected and filtered through a 70 µm strainer. This process was repeated four times to obtain different fractions. Crypts from these fractions were centrifuged at 300× *g* for 5 min at 4 °C, washed with ice-cold PBS + 0.1% BSA, and resuspended in 10 mL of ice-cold DMEM/F12 supplemented with glutamine and 15 mM HEPES (Gibco, Thermo Fisher Scientific, Waltham, MA, USA). After microscopic evaluation, the fraction with the highest crypt number and the least debris was selected. This fraction was centrifuged at 200× *g* for 5 min at 4 °C, and the pellet was resuspended in a mixture of 50% Geltrex™ GFR LDEV-free (Gibco, Fisher Scientific, Waltham, MA, USA) and 50% IntestiCult™ Mouse Organoid Growth Medium (Intesticult mouse OGM, Stemcell Technologies™, Vancouver, BC, Canada). Fifty microliters of this suspension was pipetted into the centre of each well of a pre-heated 24-well plate, and incubated at 37 °C for 10 min to allow matrix polymerization. Finally, 500 µL of IntestiCult™ Mouse OGM was added to each well. The plates were incubated at 37 °C with 5% CO_2_, and the culture medium was changed every 2–3 days.

Organoids were split at a 1:4 to 1:8 ratio every 7 days, as follows: the medium was removed and GCDR (800 µL) was added to the domes. After incubation for 1 min, the organoids were dissociated by pipetting. The suspension was transferred to a 15 mL conical tube, rinsed with additional reagent, and incubated on a rocking platform for 10 min at 20 rpm. The cells were centrifuged at 300× *g* for 5 min, washed with cold DMEM/F-12 + HEPES, and centrifuged again at 200× *g*. The pellet was resuspended in IntestiCult™ Mouse OGM and Geltrex™ GFR LDEV-free, and 50 µL of the 1:1 mixture was plated onto pre-warmed 24-well plates to form domes. The domes were incubated at 37 °C for 10 min to solidify. Finally, 500 µL of fresh medium was added to each well.

Mouse intestinal organoids were generated from C57BL/6 mice duodenum and jejunum stem cells, as previously described [30].

### 4.2. Histological Microscopy and Immunofluorescence Microscopy

Organoids were fixed in 4% (*w*/*v*) paraformaldehyde buffered with PBS at room temperature for 30 min. Subsequently, the samples were pre-embedded in 1% agarose (iNtRON Biotechnology, Seongnam, Korea), following the protocol outlined by Fujii et al. [60]. For microtome sectioning, samples were dehydrated and embedded in paraffin using a Myr STP-120 tissue processor. Three consecutive 5 μm thick sections were obtained with a Leica RM2255 rotary microtome. One section was stained with H&E, dehydrated, and mounted with DPX mounting medium (Sigma-Aldrich, Cat. No. 0652, St. Louis, MO, USA). The remaining two sections were stained using multiplex immunofluorescence with the following antibody panels: anti-chromogranin A C-12 AF 488, anti-mucin 2 AF 790, anti-actin AF 488, and anti-OZ-1 AF 594 (Santa Cruz Biotechnology, Dallas, TX, USA), at a dilution of 1:50. Stained sections were then dried and mounted with UltraCruz Hard-set Mounting Medium containing DAPI (Santa Cruz Biotechnology, TX, USA). The resulting sections were observed under a light and fluorescence microscope (Nikon H600L, Tokyo, Japan), and microphotographs were taken with a Prime BSI Scientific CMOS camera (Photometrics, Tucson, AZ, USA).

### 4.3. RNAseq Analysis

Five pieces of intestinal tissue (1 cm) were stored in RNA*later* (Fisher Scientific, Waltham, MA, USA) overnight at 4 °C to allow impregnation, and then they were kept at −80 °C for long-term storage. The remaining tissue was used to establish intestinal organoid cultures, which were grown for 21 days (one passage in total). Organoids were retrieved using ice-cold PBS, centrifuged at 300× *g* for 5 min at 4 °C, resuspended in RNA*later*, and stored at −80 °C as five independent samples.

Samples were sent, frozen, to Seplexing Genetest (Valencia, Spain), where RNA was extracted from both tissue and organoid samples using a commercial total RNA extraction kit, following the manufacturer’s protocol. RNA quality and concentration were measured using a Quantus Fluorometer (Promega, Wisconsin, USA), and libraries were prepared and sequenced using an Illumina NovaSeq X platform (2 × 150 bp, paired-end). Raw sequencing data were analyzed bioinformatically, starting with quality control using FastQC and trimming for adapter sequences. Reads were aligned to the UIRambv2 sheep genome using STAR, accounting for transcript splicing, and duplicate sequences were removed with UMI-tools. Gene expression levels were quantified using HTSeq-count, normalized with DESeq2, and analyzed for differential expression with False Discovery Rate correction using the Benjamini–Hochberg method. Results were visualized with R packages such as ggplot and pheatmap, generating heatmaps, volcano plots, PCA plots, and dispersion curves.

A KEGG pathway and biological, cellular, and molecular function analyses were performed using the DAVID software of the NIH (https://davidbioinformatics.nih.gov/, accessed on 20 March 2025). Differentially expressed genes were classified as upregulated or downregulated based on a Log2FC < −1 or >1, respectively, and a q-value < 0.1. Gene list annotation was standardized to ENTREZ GENE ID format, achieving a conversion success rate of over 99%.

### 4.4. Cytotoxicity Test in 384-Well Plates

Drug toxicity assays with sheep duodenum intestinal organoids were conducted in 384-well plates, based on previously reported protocols, with several modifications [61]. Briefly, the organoids were prepared by discarding the culture medium and adding 0.8 mL of GCDR directly onto the matrix domes. After 1 min of incubation at room temperature, the matrix was disrupted by pipetting, and the contents of the wells were collected. A consequent wash step with 0.8 mL of GCDR was performed. The suspension was incubated at room temperature on a rocking platform at 20 rpm for 10 min, and then centrifuged at 300× *g* for 5 min at 4 °C. The pellet was resuspended in 10 mL of ice-cold DMEM/F12 supplemented with glutamine and 15 mM HEPES, and centrifuged again at 200× *g* for 5 min at 4 °C. Cells were resuspended in a 1:1 mixture of Geltrex™ GFR LDEV-free and IntestiCult™ Mouse OGM. The mixture was kept on ice and distributed into a frozen 384-well plate (8 µL/well), and then incubated at 37 °C for 10 min, using frozen pipette tips and a frozen dispenser. Then, 32 µL of IntestiCult™ Mouse OGM was added to each well. The plate was incubated at 37 °C with 5% CO_2_, and on the 4th day, mature organoids were exposed to drugs. Hydrogen peroxide at a concentration of 0.03% (*v*/*v*) was used as a positive control, while 0.2% DMSO (*v*/*v*) was added as a negative control. The viability of organoids on the 7th day was assessed by adding 20% (*v*/*v*) alamarBlue™ HS (Invitrogen™, Thermo Fisher Scientific™, USA). Following an incubation time of 5 h, fluorescence was assessed using a Varioskan™ LUX microplate reader (Thermo Scientific, USA, Instrument software ver. 1.00.37, Skanlt software ver. 5.0.0.42).

### 4.5. Statistical Analysis

In order to quantify the organoids’ growth over time, photos were taken of a random culture plate’s well, and the larger diameter of 24 randomly selected organoids was retrieved using the Zeiss confocal Observer Z1 with the software ZEN 2.6 (Blue Edition, v 2.6.76.00000, file v 2.6.18299.3). Data were imported into IBM SPSS v29 (IBM Corporation, 2022, build 29.0.2.0, North Castle, NY, USA), and normality was confirmed via Kolmogorov–Smirnov with Lilliefors Significance Correction and Shapiro–Wilk tests. One-way ANOVA was subsequently performed with a Games–Howell test, due to the lack of homogeneity of variance.

To ensure the robustness and reliability of the assays, key statistical metrics were evaluated. These included the Z’-factor, S/B ratio, S/N ratio, SW, and AVR, following the guidelines provided by Iversen and colleagues [62]. The Z’-factor [63] was calculated using a 2 × 2 format, where 0.02% (*v*/*v*) hydrogen peroxide was used to establish the minimum signal, and 0.2% (*v*/*v*) DMSO was used to represent the maximum signal [64,65,66].

For dose–response curves, a minimum of six data points were obtained from at least two independent experiments. All collected data were normalized as percentages of controls, and exported to SigmaPlot^®^ 10.0 (Grafiti LLC, Palo Alto, CA, USA, Built 10.0.0.54) for 4-parameter curve fitting and analysis. Logarithmic normalization was applied to compound concentrations, and dose–response curves were considered valid when they achieved an R-value > 0.90 and a standard error <10%.

To identify any significant difference in CC_50_ values between mouse and sheep organoids, normality was confirmed through a Shapiro–Wilk test, followed by a two-way paired Student’s *t*-test. The tests were performed using IBM SPSS v29 (IBM Corporation, 2022, build 29.0.2.0).

Unless otherwise specified, statistical calculations and graphical elaborations were performed using Microsoft^®^ Office Professional Plus 2019.

## 5. Conclusions

A robust and reproducible protocol for culturing sheep duodenal intestinal organoids in an HTS-compatible format has been successfully developed. These findings highlight the potential of sheep organoids as a first-of-its-kind tool for veterinary drug development, particularly in combating challenges like drug resistance and improving livestock health. The method is cost-effective, sustainable, and adaptable for large-scale applications, paving the way for innovative approaches in veterinary pharmacology and toxicology.

## Figures and Tables

**Table 1:** Metrics and statistical values obtained from Z’-factor tests and respective averages. P1-P4 indicate four experiments (two replicates on two different days). Optimal reference values: Z’ ≥ 0.5, SW > 2, AVR < 0.5.

Metrics ^1^	P1	P2	P3	P4	Average
S/B	36	89	85	37	61
S/N	54	259	169	26	127
Z’	0.7	0.4	0.5	0.6	0.5
SW	7.0	2.0	3.5	5.2	4.4
AVR	0.3	0.6	0.5	0.4	0.5

^1^ S/B (Signal over Background); S/N (Signal over Noise); SW (Signal Window); AVR (Assay Variability Ratio).

**Table 2:** Comparisons between the CC_50_ values obtained in an HTS including mouse intestinal organoids and sheep duodenum intestinal organoids after testing with four compounds with anthelmintic activity. The data represent the mean ± standard deviation of two independent experiments carried out in triplicate.

	CC_50_ Values (μM)	
	Sheep Intestinal Organoids	Mouse Intestinal Organoids ^1^
Tolfenpyrad	<3	<1
Octenidine	7.2 ± 0.2	14.2 ± 0.41
Chalcone	>50	>50
Trans-chalcone	>50	>50

^1^ Galli et al., 2025 [30].

## Data Availability

Data supporting the reported results are available in the Zenodo repository at https://doi.org/10.5281/zenodo.14901208.

## Supplementary Materials

The following supporting information can be downloaded at https://www.mdpi.com/article/10.3390/ijms26073452/s1.

## Abbreviations

The following abbreviations are used in this manuscript:

2D	Bidimensional
3D	Three-dimensional
ADMET	Absorption, Distribution, Metabolism, Excretion, Toxicity
CC_50_	Cytotoxic Concentration 50%
CES	Carboxylesterases
CYP	Cytochrome P450
DMEs	Drug-metabolizing enzymes
H&E	Haematoxylin and eosin staining
HTS	High-throughput screening
KEGG	Kyoto Encyclopedia of Genes and Genomes
R&D	Research and Development
UGT	Uridine diphosphate-glucuronosyltransferase
