# Supplementary material for: Development of Sheep Duodenum Intestinal Organoids and Implementation of High-Throughput Screening Platform for Veterinary Applications
Source: Int J Mol Sci. 2025 Apr 7;26(7):3452. doi: 10.3390/ijms26073452 (PMC11989482; doi:10.3390/ijms26073452)
Supplement: Supplementary file 1 [file ijms-26-03452-s001.zip › ijms-3517296-supplementary.pdf]

International Journal of *Molecular Sciences*

MDPI

Supporting Information

Development of Sheep Duodenum Intestinal Organoids and Implementation of High Throughput Screening Platform for Veterinary Applications

Giulio Galli [1], Estela Melcón-Fernández [1], María-Gracia De Garnica [2], Beatriz Martínez-Fernández [2], Mahsa Dehnavi [3], Sonia Andrés [3], Yolanda Pérez-Pertejo [1,4], Rosa-María Reguera [1,4], Carlos García-Estrada [1,4], María Martínez-Valladares [3] and Rafael Balaña-Fouce [1,4,*]

[1] Departamento de Ciencias Biomédicas, Facultad de Veterinaria, Universidad de León, Campus de Vegazana s/n, 24071, León, Spain; ggal@unileon.es (G.G.); emelf@unileon.es (E.M.-F.); myperp@unileon.es (Y.P.-P.); rmregt@unileon.es (R.-M.R.); c.gestrada@unileon.es (C.G.-E.)
[2] MicrosVeterinaria, Profesor Pedro Cármenes, Campus de Vegazana, 24007, León, Spain; mgarng00@estudiantes.unileon.es (M.-G. De G.); info@microsvet.es (B.M.-F.)
[3] Instituto de Ganadería de Montaña, CSIC-Universidad de León, Finca Marzanas s/n, Grulleros, 24346 León, Spain; mahsa.d@csic.es (M.D.); sonia.andres@eae.csic.es (S.A.); mmarva@csic.es (M.M.-V.)
[4] Instituto de Biomedicina (IBIOMED), Universidad de León, Campus de Vegazana s/n, 24071 León, Spain
* Correspondence: rbalf@unileon.es; Tel.: (+34 987291590)

Table S1 Average diameter of sheep and mouse duodenal organoids at day 3, 5, and 7 with relative standard error.

	Diameter (mm) of organoids (average)	
	Sheep intestinal organoids	Mouse intestinal organoids
Day 3	95.3 ± 6.7	72.5 ± 2.8
Day 5	174.8 ± 17.9	120.0 ± 9.8
Day 7	165.0 ± 13.3	192.6 ± 10.4

Table S2. RNA expression of the 13366 transcripts during RNA seq anlysis.

	baseMean	log2FoldChange	lfcSE	stat	pvalue	padj
KRT20	472.7501486	4.872173913	0.195393628	24.93517293	3.0927E-137	4.1338E-133
PSAT1	221.4266229	-4.22398822	0.185397332	-22.78343588	6.693E-115	4.473E-111
TCF4	273.8702485	6.110010843	0.298531638	20.4668788	4.24982E-93	1.89344E-89
LOC121817413	238.3847073	-3.514056599	0.172756518	-20.34109421	5.56744E-92	1.86036E-88
OLFM4	841.5527457	4.37704441	0.21613631	20.25131461	3.45885E-91	9.24621E-88
ADA	293.2302963	5.432650245	0.285973414	18.99704654	1.80415E-80	4.01905E-77
ASCL2	167.6482193	-4.543417166	0.241124713	-18.8426027	3.37932E-79	6.45258E-76
FABP2	416.8256558	5.989860486	0.319790906	18.73055294	2.79003E-78	4.66145E-75
LOC114117878	182.5738496	5.521347746	0.297013491	18.58955202	3.90425E-77	5.79825E-74
CTSS	314.4676174	3.729814197	0.206018199	18.10429477	2.94762E-73	3.93979E-70
SH3PXD2A	204.0528602	5.103941424	0.283299839	18.01604067	1.45813E-72	1.77176E-69
JCHAIN	840.7805969	10.27169433	0.581982008	17.64950495	1.02652E-69	1.14337E-66

C17H12orf43	164.509073	-2.658428112	0.15476388	-17.17731623	3.92662E-66	4.03716E-63
PRTFDC1	145.9176797	-4.415192768	0.260713369	-16.93504551	2.48166E-64	2.36928E-61
ATP2A3	106.3197074	4.012374589	0.240996106	16.64912623	3.07053E-62	2.73604E-59
RETSAT	152.8226557	5.215963285	0.314606203	16.57934024	9.83021E-62	8.21191E-59
RPL37A	510.283429	-2.122976327	0.128483661	-16.52331756	2.493E-61	1.96008E-58
GAPDH	185.8535115	-2.875548765	0.176184537	-16.32123234	6.97139E-60	5.17665E-57
PKM	209.0808741	-3.036676358	0.186155571	-16.31257312	8.03361E-60	5.65143E-57
C4BPA	135.6421458	3.125672971	0.193764256	16.13131869	1.53703E-58	1.0272E-55
MTTP	120.1930093	4.906157497	0.306374975	16.01357129	1.0274E-57	6.53913E-55
MMP7	93.82672753	-4.926694912	0.314877007	-15.64641051	3.51494E-55	2.13549E-52
HMGA1	425.8515822	-2.326805398	0.152341857	-15.27357903	1.14701E-52	6.66563E-50
SPINK4	413.7763079	3.595795991	0.235793238	15.24978418	1.65178E-52	9.19905E-50
GGTA1	93.56399429	4.579253328	0.301866364	15.16980315	5.60446E-52	2.99637E-49
PGAM1	338.3264012	-2.28393415	0.15071921	-15.15357033	7.176E-52	3.68902E-49
CELF2	84.21048252	5.016171124	0.331784517	15.11876194	1.21811E-51	6.03011E-49
LOC114112057	104.2556547	5.761744658	0.383104089	15.03963235	4.03786E-51	1.9275E-48
LOC106990096	311.6660179	2.086471724	0.138827426	15.0292474	4.72342E-51	2.17701E-48
IER3	111.4782351	-3.747044786	0.24936292	-15.02647137	4.92554E-51	2.19449E-48
ADM	89.3007014	-4.484453255	0.303900306	-14.75633018	2.80114E-49	1.20774E-46
MGAT5	79.43359684	3.977160508	0.271070797	14.67203607	9.73786E-49	4.06738E-46
LOC101122398	71.81561049	3.43196231	0.23488818	14.61104732	2.38818E-48	9.67286E-46
PIGR	3089.435148	1.332319294	0.091271615	14.59730168	2.92183E-48	1.14862E-45
SLC4A4	253.4624133	2.995987509	0.205914832	14.54964402	5.87078E-48	2.24197E-45
MAF	174.8942368	7.035048634	0.489251954	14.37919372	6.99042E-47	2.59539E-44
LOC101107420	224.8497905	4.383962956	0.306240092	14.31544423	1.75241E-46	6.33045E-44
CSKMT	167.7133041	-3.233240481	0.226763707	-14.25819202	3.98649E-46	1.4022E-43
SOX6	154.1136885	7.289896123	0.516004914	14.12757112	2.56851E-45	8.80276E-43
U2AF1	342.2729944	-1.830199257	0.13001472	-14.07686192	5.26984E-45	1.76092E-42
SLC40A1	78.65018537	3.670177669	0.26176866	14.02069166	1.16474E-44	3.79705E-42
NEXN	88.82009133	5.049155935	0.360735564	13.99683437	1.6297E-44	5.18632E-42
SLC25A3	149.8251875	-2.760203025	0.197558443	-13.97157711	2.32422E-44	7.22455E-42
CALD1	856.6602755	11.48857037	0.823071159	13.95817389	2.8053E-44	8.52173E-42
LOC654331	270.2350402	3.013106314	0.216375441	13.92536187	4.44286E-44	1.31963E-41
PLPP1	187.0669981	2.136284958	0.153864159	13.88422731	7.89471E-44	2.29393E-41
GPX2	348.2739408	2.174413917	0.157794994	13.77999306	3.36305E-43	9.56393E-41
LOC114108841	775.9365312	12.94585851	0.942787948	13.73146372	6.57908E-43	1.832E-40
KIF12	166.4977682	-3.47533685	0.256699579	-13.53853742	9.26146E-42	2.5263E-39
PDZRN3	109.2111628	3.111131522	0.230622132	13.49016893	1.78691E-41	4.77676E-39
CREBRF	124.401671	3.201248042	0.23876098	13.40775213	5.44647E-41	1.4274E-38
CNTN1	59.91538625	4.789525206	0.361825419	13.23711644	5.35662E-40	1.37686E-37
CHP2	152.2031257	3.437700156	0.259803858	13.23190574	5.74129E-40	1.44789E-37
LOC101111337	114.7659506	3.67146262	0.27903736	13.15760234	1.53892E-39	3.80912E-37

RPS21	612.8002002	-1.838409206	0.140339667	-13.09971191	3.3051E-39	8.03199E-37
DTX4	113.6355392	-3.383066446	0.258604088	-13.08203001	4.17149E-39	9.95645E-37
H2AZ1	209.8432848	-1.803345519	0.137982336	-13.06939407	4.92563E-39	1.15502E-36
LMNB1	91.99840648	-2.664023234	0.203983232	-13.06001092	5.57198E-39	1.28405E-36
LRRC42	66.31504374	-3.385512822	0.259543285	-13.04411639	6.86483E-39	1.55517E-36
PRSS8	106.0453372	3.394738115	0.261552816	12.97916869	1.60614E-38	3.57795E-36
FYB1	93.52470062	6.277679591	0.484490722	12.95727516	2.13705E-38	4.68259E-36
LOC101102543	105.7337165	-2.731966839	0.211039644	-12.9452779	2.49857E-38	5.38644E-36
ZNF180	114.9681863	2.482077455	0.191799023	12.94103287	2.64055E-38	5.60217E-36
DDR1	190.3378812	-3.201637459	0.248866708	-12.86486846	7.09626E-38	1.48201E-35
EGLN3	161.0737841	-2.200749813	0.171280778	-12.84878451	8.73723E-38	1.79664E-35
CCDC47	112.1828476	2.897765819	0.225646693	12.84204869	9.53182E-38	1.93034E-35
LOC114109594	99.58746953	-2.991779246	0.233787937	-12.79697869	1.70455E-37	3.40045E-35
SLC7A11	75.16533199	-2.60468767	0.203846113	-12.77771568	2.1839E-37	4.29265E-35
C9	75.18200344	4.650059761	0.364215147	12.76734313	2.49527E-37	4.8336E-35
LPCAT3	125.8204967	2.309782415	0.18144968	12.7296031	4.04892E-37	7.73113E-35
LOC443295	367.4589288	2.572921358	0.202644915	12.69669832	6.16773E-37	1.1611E-34
CTTN	103.3088218	-2.523392804	0.19919156	-12.66817131	8.87595E-37	1.64772E-34
KITLG	54.45602449	4.995997644	0.394926868	12.65043747	1.11254E-36	2.03701E-34
LOC101109746	66.44237667	5.674108116	0.451063295	12.57940554	2.74085E-36	4.95056E-34
TGOLN2	178.2612425	2.641077615	0.210020121	12.57535514	2.88501E-36	5.14147E-34
NENF	225.4864626	-2.36913567	0.188745442	-12.55201529	3.8752E-36	6.81525E-34
LOC114116957	485.9089605	-2.168580409	0.173495539	-12.49934389	7.52699E-36	1.30657E-33
ATXN1	120.0697357	2.155369854	0.17255232	12.49110912	8.34817E-36	1.43053E-33
RALGPS2	70.93133115	3.200929148	0.258378852	12.38851059	3.01593E-35	5.10264E-33
TMEM263	106.7018634	2.13291845	0.172416336	12.37074456	3.76317E-35	6.28731E-33
SCIN	110.9239436	3.307281811	0.267447801	12.36608338	3.98798E-35	6.58066E-33
FOLH1	103.1880218	2.926613793	0.237168941	12.33978525	5.53032E-35	9.01294E-33
KMT2C	309.9068529	1.71056074	0.138632414	12.33882244	5.59684E-35	9.01294E-33
AHCYL1	115.8695612	-2.462198292	0.199923974	-12.31567302	7.45903E-35	1.18687E-32
FAM13B	84.52404327	2.856146325	0.231991452	12.31142917	7.86186E-35	1.23625E-32
S100A4	109.8428402	-3.25560703	0.264817665	-12.29376834	9.78366E-35	1.52056E-32
MCM2	100.6520294	-2.836891182	0.231425639	-12.2583271	1.51597E-34	2.32902E-32
NEURL1B	73.00217567	-2.986100065	0.243911917	-12.2425345	1.8419E-34	2.79759E-32
BAZ2A	195.0773458	2.431121296	0.198732617	12.23312678	2.06821E-34	3.10604E-32
ANKRD44	70.47348837	4.208092031	0.34410592	12.22906026	2.1744E-34	3.22922E-32
MYH11	270.859855	11.4250034	0.937464028	12.18713792	3.63981E-34	5.34613E-32
TLN1	167.6092142	2.613712325	0.215401227	12.13415707	6.96232E-34	1.0115E-31
LOC101103860	93.7137257	3.93562794	0.325492008	12.09131973	1.17386E-33	1.68707E-31
RYR2	61.93216567	4.665192863	0.386557885	12.06854923	1.5484E-33	2.20169E-31
LOC101109747	110.1154673	7.534157231	0.626795759	12.0201152	2.78583E-33	3.91951E-31
CBX3	204.7953439	-2.251316697	0.188462746	-11.94568554	6.83842E-33	9.52107E-31

LOC114117773	69.97794966	3.447427343	0.289209525	11.92017221	9.29163E-33	1.28033E-30
GTL2	225.9287499	11.16380959	0.937489672	11.90819476	1.07275E-32	1.46309E-30
IGFBP2	43.3051636	-4.476245128	0.376028127	-11.90401675	1.12785E-32	1.52271E-30
ARHGAP30	61.24532014	5.647375972	0.477050237	11.83811585	2.47961E-32	3.31425E-30
SAMHD1	52.94362487	3.667481336	0.31053651	11.81014539	3.45963E-32	4.57835E-30
MSN	76.84427874	3.124950537	0.265646068	11.76358664	6.01248E-32	7.87871E-30
SLC7A5	196.904716	-2.07565631	0.176519768	-11.75877543	6.36508E-32	8.25977E-30
MYO6	468.3961646	2.145517005	0.183221629	11.70995485	1.1334E-31	1.45664E-29
SLC12A2	444.0827033	-2.059312568	0.176148252	-11.69079193	1.42056E-31	1.8083E-29
RPL36A	247.5224624	-1.873319941	0.160629065	-11.6623971	1.98378E-31	2.50143E-29
ABCG2	42.77873344	4.554047455	0.391301133	11.6382169	2.63466E-31	3.29111E-29
TRIM31	57.19796314	4.069444525	0.350519184	11.60976264	3.67635E-31	4.54982E-29
LOC101120733	85.0175836	2.802834878	0.242084417	11.57792355	5.3322E-31	6.53855E-29
ENO1	217.4192984	-1.536532251	0.133121809	-11.542303	8.07343E-31	9.80995E-29
MEIS2	82.34156724	2.169618954	0.188066566	11.5364416	8.64271E-31	1.04071E-28
RPL15	227.6894503	-3.334966422	0.289374274	-11.52475089	9.89981E-31	1.18144E-28
BAZ2B	355.3354575	2.478330843	0.215364432	11.50761443	1.20773E-30	1.42854E-28
PRAP1	178.014665	9.212585594	0.80285394	11.47479652	1.76593E-30	2.07047E-28
GSN	74.38719592	2.853155141	0.248687481	11.47285391	1.80603E-30	2.09908E-28
SORBS1	56.73728876	4.485823183	0.39105447	11.47109554	1.84311E-30	2.12371E-28
CNN1	166.1165721	9.116988102	0.795245596	11.46436792	1.99209E-30	2.27575E-28
LOC101119706	55.22544186	-3.434507643	0.299859995	-11.45370406	2.25308E-30	2.55209E-28
PTPRC	229.7837585	10.61005493	0.926643174	11.44998985	2.35173E-30	2.64145E-28
LOC101117756	145.7729183	3.148036293	0.275390678	11.43116504	2.92162E-30	3.2542E-28
MAP9	154.3609963	2.269098412	0.198693378	11.42010082	3.31849E-30	3.66569E-28
MYLK	219.1505938	9.983615682	0.87491639	11.41093686	3.68736E-30	4.03978E-28
ATP10B	83.62584727	2.624999141	0.230751047	11.37589264	5.51358E-30	5.99143E-28
ST6GALNAC4	90.75387086	2.444846226	0.215043631	11.36907063	5.9619E-30	6.42635E-28
FNBP1	84.02341041	2.48946713	0.219164072	11.35891985	6.69675E-30	7.15435E-28
MYL9	343.0896424	4.81338734	0.42377705	11.35830112	6.74434E-30	7.15435E-28
PCDH19	40.81832533	-4.615675304	0.408149801	-11.30877753	1.18732E-29	1.24959E-27
STK40	90.2982316	2.786197764	0.246610945	11.29794853	1.34319E-29	1.40259E-27
GSTA1	219.4497255	2.803604437	0.248243268	11.29377831	1.40849E-29	1.45937E-27
AKAP12	72.31331017	6.705823132	0.594407732	11.2815207	1.61922E-29	1.66481E-27
CASP1	55.016899	4.124219571	0.366224965	11.26143754	2.03413E-29	2.07543E-27
DBI	644.8056033	1.752140596	0.155676478	11.25501181	2.18797E-29	2.21548E-27
ALDOB	63.10389695	3.376394608	0.300117309	11.25024953	2.30938E-29	2.32084E-27
GPCPD1	127.7360734	2.258784033	0.200824763	11.24753739	2.38149E-29	2.37544E-27
ETV4	54.18204898	-3.129406615	0.280677563	-11.14947196	7.20326E-29	7.13176E-27
ACTG2	131.6903461	10.38326203	0.931646975	11.14506063	7.56927E-29	7.43904E-27
COL4A1	149.2824273	10.56536285	0.948599617	11.13785274	8.20743E-29	8.00734E-27
MAOA	44.97186083	4.743500696	0.425967575	11.13582575	8.39632E-29	8.13226E-27

GCNT4	79.17417546	2.656237862	0.238629111	11.13123982	8.83973E-29	8.50014E-27
GTF3C1	180.9478722	2.873519144	0.258367319	11.12183676	9.82286E-29	9.37802E-27
LOC101114579	148.2028797	10.55663541	0.949606376	11.11685397	1.0387E-28	9.84632E-27
STK4	120.5356838	2.437048268	0.219470643	11.1042107	1.1967E-28	1.12641E-26
LOC101103222	218.9192454	2.590685732	0.233342814	11.10248776	1.22E-28	1.14032E-26
S100A2	151.0092657	-3.320790814	0.300061845	-11.06702126	1.81328E-28	1.68308E-26
IGF2R	102.0410708	-2.395747884	0.216593808	-11.06101741	1.93886E-28	1.78722E-26
LOC101110577	117.811206	10.22543871	0.926803618	11.03301553	2.64833E-28	2.42449E-26
UGDH	63.01031423	2.895573968	0.262559265	11.02826812	2.7919E-28	2.53854E-26
KEF53_p11	1142.477063	2.456227178	0.222842041	11.02227912	2.98406E-28	2.69493E-26
HNRNPA0	106.1765363	-2.613040175	0.2371988	-11.01624534	3.19093E-28	2.86241E-26
HNF4G	73.1675799	2.202518724	0.200337596	10.99403588	4.08253E-28	3.63781E-26
DST	123.4721372	2.117525157	0.192656044	10.99122098	4.21189E-28	3.72822E-26
SNRPB2	259.9592361	-1.526078018	0.138914208	-10.98575908	4.47461E-28	3.93471E-26
ADAMDEC1	45.04498063	5.5215544	0.50306197	10.97589309	4.99102E-28	4.36013E-26
OVAR-DRB1	97.69483174	7.95643652	0.726113889	10.95756002	6.11252E-28	5.30519E-26
PGM2	90.45386029	2.524410202	0.230658021	10.94438506	7.06958E-28	6.09626E-26
VIL1	121.6539417	2.264914477	0.207226162	10.92967439	8.31462E-28	7.12393E-26
RFC3	44.35303604	-2.803205133	0.257040964	-10.90567468	1.08287E-27	9.21888E-26
C21H11orf86	52.66981826	3.539897502	0.324621096	10.90470567	1.09447E-27	9.25865E-26
COLGALT1	74.35714378	-2.557271013	0.234567993	-10.90204584	1.12694E-27	9.47342E-26
C1QB	106.3560657	10.07669591	0.924944806	10.89437537	1.22605E-27	1.02421E-25
TMEM214	51.02294099	2.621148408	0.241314734	10.86194932	1.74974E-27	1.45261E-25
MGP	118.9130858	10.2360159	0.942738211	10.8577501	1.83207E-27	1.51157E-25
FOS	38.29252906	3.859517129	0.355632258	10.85255074	1.93936E-27	1.59028E-25
RPS13	190.9472472	-1.904454776	0.175497385	-10.85175583	1.95631E-27	1.59439E-25
IGF1	115.7820024	10.19692848	0.940201478	10.84547165	2.09552E-27	1.69273E-25
RDX	48.61357073	4.814383383	0.443919324	10.84517641	2.1023E-27	1.69273E-25
ASPH	104.0341358	1.815255011	0.167560906	10.83340411	2.39098E-27	1.91364E-25
LOC132657910	151.9548535	-1.889971012	0.174544471	-10.82801993	2.53579E-27	2.01747E-25
NR2F1	39.37323363	5.019315878	0.463651491	10.82562222	2.60305E-27	2.05872E-25
WIPF1	86.34869561	2.125257415	0.196344031	10.82415088	2.6452E-27	2.07975E-25
SELENOH	113.2106612	-3.017992207	0.279182788	-10.81009409	3.08354E-27	2.41021E-25
TM4SF1	98.96422843	-2.285239321	0.211743318	-10.79249795	3.73497E-27	2.90242E-25
EPS8L2	56.17104534	3.167477085	0.293729792	10.78364255	4.1127E-27	3.17748E-25
LOC101115586	190.4454084	2.535525002	0.235186858	10.78089577	4.23739E-27	3.255E-25
OCIAD2	94.25958655	2.823136959	0.261920965	10.77858339	4.34526E-27	3.31878E-25
FCF1	133.4732135	-2.838018093	0.263624598	-10.76537665	5.0156E-27	3.80901E-25
RXRA	103.2256586	1.851791891	0.172152031	10.75672405	5.5094E-27	4.16037E-25
DES	97.50069441	9.956546951	0.927550364	10.73423864	7.0296E-27	5.27852E-25
MYO1D	123.8484969	-1.904879343	0.177583907	-10.72664398	7.63177E-27	5.69867E-25
DVL2	54.1094984	-3.311388132	0.309228244	-10.70855653	9.27974E-27	6.89072E-25

CD74	123.3195389	8.675109188	0.810811949	10.69928631	1.02565E-26	7.57395E-25
ABCC2	69.66259447	4.60699852	0.430691163	10.69675654	1.05403E-26	7.74077E-25
MON1A	52.07092467	3.24449376	0.303383158	10.69437664	1.08144E-26	7.89868E-25
CAMK2N1	96.2810362	2.318268799	0.216786044	10.69381016	1.08807E-26	7.90389E-25
PALM2AKAP2	55.98220454	6.339576302	0.593347782	10.68441898	1.20401E-26	8.69878E-25
LOC101121563	35.98997282	4.372749467	0.409896361	10.66793922	1.4378E-26	1.0332E-24
MCM4	75.04760279	-2.019908384	0.189397382	-10.66492244	1.48523E-26	1.06158E-24
KEF53_p09	175.9089039	3.16637111	0.297511032	10.64286957	1.88241E-26	1.33831E-24
CCDC88A	39.72436982	4.430800763	0.417001643	10.62537962	2.27086E-26	1.60465E-24
EIF3F	182.4754493	-1.616875849	0.152177088	-10.62496248	2.28104E-26	1.60465E-24
CYBB	72.16095127	2.993410393	0.282195431	10.60757925	2.74776E-26	1.92286E-24
NASP	171.543664	-1.861201679	0.175685704	-10.59392791	3.17963E-26	2.21349E-24
TENT5C	180.1435369	2.178200983	0.205746001	10.58684481	3.42957E-26	2.37511E-24
LOC101119393	66.01370607	2.17371232	0.205420805	10.58175352	3.62117E-26	2.49487E-24
PSMB10	140.6202363	2.212450051	0.209098231	10.58091235	3.65383E-26	2.50447E-24
PLEC	243.5506836	1.741650213	0.164701134	10.57460975	3.90801E-26	2.66502E-24
MTHFD1L	47.61100742	-3.47350569	0.328696694	-10.56751027	4.21537E-26	2.86003E-24
SNRPD1	181.8795194	-2.102716111	0.199693687	-10.52970749	6.303E-26	4.25485E-24
RLF	52.90716212	-2.36458081	0.224742601	-10.52128435	6.89275E-26	4.62957E-24
SHMT2	48.19273124	-2.604409652	0.247558885	-10.52036429	6.96039E-26	4.65163E-24
PSMB9	110.4754774	2.830208221	0.269289522	10.50990845	7.7769E-26	5.17145E-24
NFKB1	66.45057923	2.385659028	0.227032279	10.5080169	7.93444E-26	5.25009E-24
FSTL1	37.11858552	3.855804656	0.366984089	10.50673522	8.04298E-26	5.29569E-24
P4HA1	113.1884803	-2.026529353	0.192953093	-10.50270468	8.394E-26	5.49971E-24
PPM1K	55.8726563	3.05383083	0.291029949	10.49318408	9.28458E-26	6.05354E-24
BRI3	205.9166555	-3.28388521	0.313562615	-10.47282123	1.15159E-25	7.47191E-24
LOC101107171	89.5863114	7.856683444	0.750289019	10.47154263	1.16725E-25	7.53695E-24
CISD3	76.8787259	2.309344032	0.220721022	10.46272808	1.28113E-25	8.23249E-24
COL18A1	37.34337832	4.735085365	0.452682629	10.46005537	1.31779E-25	8.42753E-24
RPS7	963.8766973	-1.273510635	0.121816488	-10.45433712	1.39974E-25	8.90902E-24
LOC114114252	39.17504807	3.427203412	0.328140726	10.44430982	1.55583E-25	9.85555E-24
AGFG1	170.235116	1.68253724	0.161214705	10.4366239	1.68704E-25	1.05864E-23
VAMP8	84.26654932	2.170219701	0.207942553	10.43663105	1.68691E-25	1.05864E-23
VSIR	33.33216008	4.56058973	0.437178646	10.43186755	1.77367E-25	1.1078E-23
MAP1B	67.08395078	3.604733625	0.346093412	10.41549333	2.10702E-25	1.30988E-23
HYOU1	85.92866738	1.9967783	0.191767175	10.4125135	2.17404E-25	1.34529E-23
EDEM1	75.04357233	2.470722394	0.237436122	10.40584041	2.33189E-25	1.43632E-23
TNFRSF12A	60.8818586	-2.963650258	0.285424063	-10.38332308	2.95313E-25	1.81062E-23
LOC132657206	81.49228212	9.692097246	0.933825352	10.37891852	3.09259E-25	1.88747E-23
SLC27A2	33.37594125	3.881693138	0.374115807	10.37564591	3.20041E-25	1.94439E-23
PIK3R5	48.74077907	5.935373858	0.573210881	10.3546078	3.98818E-25	2.41204E-23
SPARCL1	87.70559877	9.794772159	0.946007053	10.35380458	4.02179E-25	2.42141E-23

LGALS1	35.08471641	4.787470451	0.462749391	10.34570881	4.37665E-25	2.62325E-23
HNRNPAB	180.0378312	-1.496221088	0.144629855	-10.34517448	4.40114E-25	2.62614E-23
FILIP1	48.12371745	6.150641763	0.594873388	10.3394132	4.67391E-25	2.77651E-23
KMT5C	45.55693828	-2.947259978	0.285977281	-10.30592351	6.62522E-25	3.91826E-23
TM4SF20	61.00543381	3.862343982	0.374786812	10.30544262	6.65844E-25	3.92056E-23
TPD52L2	156.1921208	-2.077472206	0.201865326	-10.29137717	7.70661E-25	4.51783E-23
PSPH	91.41825589	-2.183011493	0.212333953	-10.28102886	8.58067E-25	5.00826E-23
TPM2	123.1062156	9.707540182	0.94433614	10.2797508	8.69521E-25	5.05305E-23
CKB	217.3711801	2.484816142	0.241753824	10.27829096	8.82791E-25	5.10796E-23
RBM47	270.9728385	1.344788745	0.13098304	10.26689217	9.93538E-25	5.72398E-23
COL3A1	106.824758	9.506687313	0.926236023	10.26378491	1.02605E-24	5.88589E-23
ITPR3	55.15497645	-2.768334303	0.269868538	-10.25808463	1.08844E-24	6.21714E-23
TBX3	216.957231	2.223307074	0.217030279	10.24422528	1.25627E-24	7.14525E-23
COL6A3	76.68235231	9.605145556	0.937745302	10.2428085	1.27481E-24	7.21712E-23
MAPRE2	38.59665476	3.071443921	0.29987431	10.24243765	1.27971E-24	7.21712E-23
LOC101113715	152.4386441	3.199518238	0.312610533	10.23483825	1.38426E-24	7.77397E-23
LPAR1	43.12050677	2.840302654	0.277874599	10.22152679	1.58819E-24	8.88193E-23
STK39	135.759283	2.262681694	0.221658074	10.20798229	1.82623E-24	1.01706E-22
LOC114112490	367.6509065	4.537649478	0.445393976	10.18794533	2.24458E-24	1.24486E-22
PKIB	37.75812218	5.401384114	0.530387767	10.18383993	2.34136E-24	1.29316E-22
SYNM	51.80758642	2.456943793	0.24127932	10.18298538	2.36202E-24	1.29921E-22
RSL24D1	167.6858844	-1.49526702	0.146897617	-10.17897394	2.46143E-24	1.34834E-22
BCAS1	59.83336213	2.749505009	0.270291623	10.17236485	2.63434E-24	1.43717E-22
RPS15	153.9274124	-2.375937648	0.233608813	-10.17058224	2.683E-24	1.45776E-22
HOOK3	163.2160265	1.702066244	0.167493135	10.16200601	2.92985E-24	1.58544E-22
AHCY	214.3705261	-2.176621623	0.214789093	-10.13376235	3.91297E-24	2.1089E-22
ACO1	101.8951917	1.919561465	0.1894965	10.12979907	4.07486E-24	2.18441E-22
SSBP3	112.9869359	1.724984893	0.170292558	10.12953774	4.08576E-24	2.18441E-22
CCNI	134.3936275	-2.1283364	0.210315584	-10.11972748	4.51671E-24	2.4052E-22
DCN	72.54937281	9.527714347	0.943088643	10.10267106	5.37577E-24	2.85129E-22
PIP4K2A	94.55015922	1.95058219	0.193340684	10.08883467	6.19E-24	3.27018E-22
HELLS	78.00676491	-2.078958213	0.206213058	-10.08160316	6.663E-24	3.50621E-22
HNRNPDL	810.3320403	-1.227214242	0.121765524	-10.07850337	6.87655E-24	3.60439E-22
RPS2	104.8775015	-2.605488683	0.258699214	-10.07149825	7.38445E-24	3.85549E-22
MYO5A	45.08464566	3.66379606	0.363825589	10.07019893	7.48266E-24	3.89156E-22
LOC121819392	99.09693584	-1.786178627	0.177537056	-10.0608778	8.22616E-24	4.26166E-22
USP40	113.0355084	1.578576291	0.157134502	10.04601964	9.56533E-24	4.9363E-22
IMP3	71.29948898	-2.067416573	0.206286413	-10.02206857	1.21924E-23	6.26783E-22
DCBLD1	86.67225037	-1.795119315	0.17921513	-10.01656121	1.2891E-23	6.60159E-22
YBX1	246.3989436	-2.07554095	0.207309385	-10.01180409	1.35263E-23	6.90048E-22
EPHB3	121.4908702	-2.359734636	0.236203618	-9.990256112	1.68147E-23	8.54543E-22
SMAD1	179.8103885	2.018985529	0.202218499	9.984178213	1.78776E-23	9.05123E-22

FXR2	60.40081681	-2.535783799	0.254048449	-9.981496878	1.83675E-23	9.26413E-22
BCL3	55.93141181	2.307835008	0.231295376	9.977869196	1.90514E-23	9.57297E-22
MOV10	87.56081041	2.784235598	0.279414904	9.9645207	2.17922E-23	1.09092E-21
TMA16	151.3338846	-1.532183648	0.153771834	-9.964007132	2.19052E-23	1.09248E-21
COX5A	82.96609224	-2.082853651	0.20915152	-9.958587208	2.31327E-23	1.14941E-21
SOX9	187.3719991	-2.218368845	0.22278352	-9.957508717	2.33849E-23	1.15764E-21
LMNA	72.5269038	-2.207963808	0.22182135	-9.953793044	2.4275E-23	1.19727E-21
LOC101115671	40.77344149	4.716470036	0.474202845	9.94610236	2.62254E-23	1.28871E-21
CLDN4	58.20544444	-2.926495551	0.29465522	-9.931931801	3.0234E-23	1.48025E-21
NR1D2	41.69454536	2.621729888	0.264105493	9.926828328	3.18217E-23	1.55229E-21
FLYWCH2	145.6194193	1.888710832	0.19034282	9.922679685	3.31728E-23	1.60648E-21
GNG11	33.73971311	5.070193461	0.510969839	9.922686379	3.31706E-23	1.60648E-21
LOC101110918	62.3861522	-3.019485419	0.304360882	-9.920740804	3.38236E-23	1.63208E-21
CDH26	63.60957596	9.334595954	0.941749188	9.911976638	3.69267E-23	1.7754E-21
EIF4A1	135.3056736	-2.374674075	0.23967424	-9.907923661	3.84555E-23	1.84228E-21
CLUH	168.4982961	-1.781485046	0.180083059	-9.892574334	4.48347E-23	2.14022E-21
NMNAT1	174.0069155	1.692939223	0.171239585	9.886377761	4.76975E-23	2.26877E-21
DDIT4	132.4515343	-3.032784857	0.306819563	-9.884587641	4.85577E-23	2.3015E-21
UHMK1	161.8885279	2.113708947	0.213879459	9.88271131	4.94758E-23	2.33673E-21
MAP7D3	65.3496901	9.371863283	0.948547365	9.880226997	5.07179E-23	2.38696E-21
VDR	42.4124044	2.708596571	0.274187542	9.878627421	5.15339E-23	2.41685E-21
CCDC80	67.84272501	9.42621459	0.95438571	9.876734834	5.25162E-23	2.45431E-21
CLIC5	31.18752237	4.390195402	0.444790571	9.870252856	5.60232E-23	2.60908E-21
TNS1	74.20475167	7.565957307	0.767095965	9.863117071	6.01524E-23	2.79166E-21
ITSN2	223.1838822	1.732938578	0.175729841	9.861379106	6.12029E-23	2.83058E-21
MEF2C	93.63393821	9.315557447	0.94535475	9.85403357	6.58471E-23	3.03487E-21
ABCB1	49.35912996	4.605164218	0.467446365	9.851748919	6.73615E-23	3.094E-21
IGFBP5	74.532494	7.538754479	0.766857003	9.830717392	8.30252E-23	3.80039E-21
UBE2D2	126.6802398	-1.637394555	0.166576462	-9.82968742	8.38787E-23	3.82636E-21
RCC1	128.936369	-1.834221664	0.186681182	-9.825423441	8.75055E-23	3.97823E-21
LOC121819972	73.83088131	2.670138459	0.271967633	9.817853794	9.43304E-23	4.27397E-21
IFNE	78.12493858	-1.828181245	0.186514528	-9.801816839	1.10579E-22	4.99322E-21
RPL23A	722.1865344	-2.044555575	0.208606137	-9.801032721	1.1144E-22	5.01519E-21
TUBB2B	149.2359397	-4.791703262	0.489264207	-9.793692637	1.19838E-22	5.375E-21
GFPT1	741.7128676	1.385205448	0.141473232	9.79129005	1.2272E-22	5.48587E-21
AGR2	410.6510734	2.641767415	0.269901609	9.787890569	1.26916E-22	5.65453E-21
DPT	58.44585166	9.209120942	0.941222976	9.784207546	1.31622E-22	5.84473E-21
GNL3	73.82213574	-2.230361388	0.228070136	-9.779278527	1.38192E-22	6.11614E-21
DENND5B	120.6255839	1.994706784	0.204099497	9.773207722	1.46731E-22	6.47264E-21
CFB	350.769639	1.730780154	0.177606964	9.745001619	1.93776E-22	8.51976E-21
ARHGEF11	83.66320302	2.312434257	0.237345839	9.742889388	1.97847E-22	8.67025E-21
VEGFA	78.6287316	-2.456448113	0.25213919	-9.742428842	1.98746E-22	8.68118E-21

COL1A2	55.12236617	9.127864783	0.937213327	9.739367247	2.04826E-22	8.91759E-21
PRNP	66.25905911	2.336317381	0.2399161	9.738060031	2.07477E-22	9.0037E-21
DIO2	54.71610253	9.124850566	0.937441029	9.733786215	2.16385E-22	9.3599E-21
ADTRP	49.9381815	2.737165478	0.281248176	9.732207038	2.19772E-22	9.47572E-21
PNPLA8	47.48054443	2.747958495	0.28244449	9.729198442	2.2637E-22	9.7288E-21
DLC1	30.07623414	4.606926402	0.473864877	9.72202546	2.42901E-22	1.04058E-20
ARHGAP26	69.21458915	2.197260196	0.226028578	9.721161005	2.44972E-22	1.04501E-20
PATZ1	82.90534581	-2.344901479	0.241221629	-9.720942062	2.455E-22	1.04501E-20
ADAMTS1	91.17785869	8.710381919	0.896200828	9.719229943	2.49662E-22	1.05936E-20
CDS1	132.0328423	1.795907895	0.18499666	9.707785495	2.7934E-22	1.18154E-20
CCDC186	150.7626578	1.874797457	0.193184588	9.704694731	2.87937E-22	1.21406E-20
CASP7	94.8166885	1.847259624	0.19044167	9.699870972	3.0188E-22	1.26885E-20
MAGT1	197.3106551	1.546584435	0.159576245	9.691821229	3.26651E-22	1.36866E-20
MFSD2A	66.99366151	2.035427918	0.210398048	9.674176812	3.88203E-22	1.62148E-20
FAM114A1	112.8313813	2.354205809	0.243758545	9.657941663	4.54912E-22	1.89419E-20
LPP	246.8266357	1.579382402	0.163553595	9.656665741	4.60612E-22	1.91197E-20
SLC33A1	64.60121375	2.134050135	0.22113167	9.650585724	4.88758E-22	2.02252E-20
DLG5	40.99719386	-2.779716351	0.288196133	-9.64522431	5.14985E-22	2.12447E-20
LOC114117131	37.78723817	4.238715198	0.439840983	9.636926447	5.58347E-22	2.29627E-20
CHD9	218.3835951	1.604695706	0.166836056	9.61839872	6.68639E-22	2.74142E-20
PABPC4	143.4636585	-2.16235076	0.224891667	-9.615077306	6.90575E-22	2.8227E-20
STMN1	96.24662067	-2.864550896	0.297955679	-9.614016783	6.97727E-22	2.84324E-20
LGALS9	180.7150272	1.615210367	0.168437706	9.589363339	8.86302E-22	3.6007E-20
EPAS1	50.26905224	2.637470844	0.27513818	9.585986383	9.15784E-22	3.7092E-20
ALDH9A1	116.4443936	-2.172838981	0.226764694	-9.581910416	9.52662E-22	3.84691E-20
ATP1B1	599.085453	-1.169306731	0.122088361	-9.577544697	9.93793E-22	4.00092E-20
ANTXR2	76.34480133	2.386196305	0.249161708	9.576898181	1.00003E-21	4.01394E-20
PHGDH	37.09438489	-3.468965185	0.362257615	-9.575962072	1.00913E-21	4.03835E-20
LOC101102105	41.20713372	-8.94050265	0.934372124	-9.568460386	1.08509E-21	4.32937E-20
SLC8A1	26.85005792	4.227081809	0.44191604	9.565350499	1.11822E-21	4.44825E-20
MSH6	121.473801	-1.508528793	0.157749784	-9.562794635	1.14619E-21	4.54599E-20
LOC100125357	59.04409634	6.956117565	0.727818312	9.557491818	1.20645E-21	4.77083E-20
ABCB6	101.9542394	1.76802903	0.185077991	9.55288641	1.26132E-21	4.9731E-20
ATF6B	114.8254341	-2.024978149	0.21201097	-9.551289489	1.28092E-21	5.03552E-20
PPM1A	131.4957026	1.52756555	0.160053817	9.54407447	1.37328E-21	5.38279E-20
C9H8orf33	110.3765294	-1.660986677	0.17425067	-9.532168119	1.54032E-21	6.01986E-20
LOC101107908	34.6841848	3.499839467	0.367622444	9.520200761	1.72845E-21	6.7354E-20
TMBIM6	159.1708943	1.506983461	0.158323708	9.518368894	1.75918E-21	6.83525E-20
SELENOP	90.32300469	1.866771777	0.196264807	9.511495224	1.87941E-21	7.28123E-20
ELK4	93.2040383	1.598193252	0.168328845	9.494470521	2.21333E-21	8.55012E-20
LAPTM5	45.45327018	8.845072755	0.931800551	9.492452803	2.25661E-21	8.69217E-20
PACSIN3	111.3686009	-2.289488298	0.241246395	-9.49024874	2.30484E-21	8.85243E-20

GNB4	66.56216043	8.81927628	0.929748257	9.485660466	2.40853E-21	9.22418E-20
SIGLEC10	31.15014588	5.307379255	0.559940158	9.478475837	2.58024E-21	9.85358E-20
RIOK3	54.76640639	2.121898158	0.224436161	9.454350639	3.25033E-21	1.23772E-19
METTL16	76.84314196	-2.096868648	0.22187514	-9.450669628	3.3667E-21	1.27839E-19
LOC101115927	43.64403218	2.894925528	0.306475344	9.445867606	3.52472E-21	1.3346E-19
DAZAP1	94.48730957	-1.696766386	0.179643941	-9.445163451	3.5485E-21	1.33981E-19
FAM13A	93.98862185	2.130215474	0.225580217	9.443272549	3.61315E-21	1.36038E-19
RPS9	158.4278145	-1.836375004	0.194786816	-9.427614473	4.19521E-21	1.57509E-19
TMEM200A	29.88984134	-2.929271542	0.311464749	-9.404825258	5.21167E-21	1.95124E-19
SYNE1	44.61444568	8.822190125	0.938125766	9.404059072	5.24978E-21	1.96002E-19
PDLIM3	42.13187592	8.737941696	0.930698836	9.388581309	6.08138E-21	2.26417E-19
S100A14	195.5397384	-1.646690538	0.175959995	-9.358323375	8.1012E-21	3.00779E-19
IGF2	44.74334057	6.527942101	0.698025694	9.352008327	8.5999E-21	3.18411E-19
LOC101109941	117.7468437	1.875206721	0.20053687	9.350932448	8.68784E-21	3.20778E-19
PLCB1	42.61268141	8.754926039	0.936723084	9.34633318	9.0739E-21	3.3411E-19
CAPN1	92.69964152	-2.224747997	0.238372951	-9.333055551	1.02862E-20	3.77707E-19
TMEM63A	30.07149928	3.422182376	0.367231024	9.318881456	1.17573E-20	4.30543E-19
CPS1	43.02347092	8.770912557	0.941692422	9.313988683	1.2312E-20	4.49623E-19
BEX2	97.04084544	-1.721772898	0.185271107	-9.293261761	1.49631E-20	5.44951E-19
CXCL8	58.1750655	3.215814357	0.346678705	9.276065453	1.75853E-20	6.38708E-19
LOC105609544	476.6029376	-1.557672303	0.168032396	-9.27007138	1.86021E-20	6.73811E-19
PRRG2	44.08987066	2.440191857	0.263314947	9.267198424	1.91099E-20	6.90333E-19
POSTN	39.68794827	8.656124507	0.934238316	9.265435122	1.94284E-20	6.99944E-19
HES1	230.9459014	-1.534731534	0.1658563	-9.25338101	2.17501E-20	7.81485E-19
GPS2	132.6366179	2.663072411	0.28849428	9.230936606	2.68276E-20	9.61333E-19
GALE	72.24067649	1.775326259	0.19234426	9.229941476	2.7078E-20	9.67712E-19
KCTD12	27.47485595	4.60073253	0.498490279	9.229332494	2.72324E-20	9.70634E-19
ZEB1	33.30935359	5.613541021	0.608384318	9.226965345	2.78408E-20	9.8968E-19
SYNCRIP	263.6903495	-1.13035181	0.122583221	-9.221097339	2.94075E-20	1.0426E-18
REG4	43.50076658	2.323689301	0.252139326	9.215893983	3.08695E-20	1.09154E-18
PSMC2	39.94575953	-2.340177887	0.25406351	-9.210995656	3.23114E-20	1.13951E-18
SH3BGRL2	84.85629515	1.83257802	0.199028073	9.207635863	3.33386E-20	1.17264E-18
GPI	56.35943369	-2.170272749	0.235797594	-9.203964778	3.4498E-20	1.21024E-18
TAGLN	54.47216684	7.50539021	0.816197163	9.195560273	3.73045E-20	1.30527E-18
PDE4D	50.08600281	2.309581914	0.251435654	9.185578388	4.09322E-20	1.42846E-18
LOC121818276	2073.834508	2.399552757	0.261456053	9.177652331	4.40592E-20	1.53358E-18
SYNPO2	65.14525217	8.228240193	0.897298827	9.170011087	4.7297E-20	1.64201E-18
POMP	317.5611993	-1.489564284	0.162764651	-9.151644904	5.60731E-20	1.94164E-18
LOC101108745	37.62554616	4.990668783	0.546395953	9.133795288	6.6139E-20	2.28427E-18
PRKG1	42.75116028	8.755355567	0.958624937	9.133244118	6.64767E-20	2.29002E-18
GAB2	134.700196	1.725351039	0.188956253	9.130955005	6.78977E-20	2.33296E-18
NEK6	184.683974	-1.25925129	0.1379264	-9.12987863	6.85761E-20	2.35023E-18

PAG1	75.62754299	1.933117624	0.211883288	9.123502098	7.27351E-20	2.48639E-18
ARPC3	213.592627	-1.925144859	0.211022371	-9.122942016	7.31121E-20	2.4929E-18
S100A11	56.88252124	-2.855526384	0.313039782	-9.121928102	7.37995E-20	2.50993E-18
ONECUT2	565.7082777	1.051072586	0.115254267	9.119598059	7.54035E-20	2.55798E-18
TCP1	60.64415127	-2.265535049	0.248433998	-9.119263341	7.56367E-20	2.55939E-18
AP3D1	290.933187	1.808530884	0.198730361	9.100425694	8.99785E-20	3.037E-18
CTBP2	152.8470446	2.083409458	0.228975067	9.098848558	9.12945E-20	3.07366E-18
RASGEF1B	50.47808976	2.222700791	0.24461468	9.08653885	1.02242E-19	3.43358E-18
INTS1	61.80071956	2.320894252	0.255732023	9.075493267	1.13164E-19	3.79084E-18
EEF2	325.2947994	-1.398632559	0.154134768	-9.074088719	1.14633E-19	3.83045E-18
CPM	74.05341368	-2.439741998	0.268901903	-9.072981524	1.15804E-19	3.85994E-18
CSDE1	328.7920396	-1.237592093	0.136475732	-9.068220969	1.20976E-19	4.0223E-18
ST3GAL1	77.30365819	2.178932406	0.240642916	9.054629325	1.37032E-19	4.54485E-18
AIMP1	96.52106636	-1.519817057	0.167913755	-9.05117665	1.41436E-19	4.67929E-18
RPL37	703.5133497	-1.618247513	0.178959943	-9.042512446	1.53111E-19	5.05306E-18
COL1A1	34.25595122	8.445262856	0.9349345	9.032999488	1.67029E-19	5.49881E-18
LOC114109611	50.02803797	8.406680186	0.931993356	9.020107424	1.87905E-19	6.17085E-18
HSD17B11	109.1933078	1.960728424	0.217430299	9.017733193	1.92021E-19	6.29058E-18
SEPTIN6	45.40918217	2.942162724	0.326364987	9.014945969	1.96968E-19	6.43684E-18
ATP5F1A	112.3688064	-2.049239018	0.22761479	-9.003101325	2.1943E-19	7.15341E-18
LOC101119116	56.53032486	2.179309999	0.242075246	9.002614001	2.20406E-19	7.16776E-18
OTULINL	30.31758542	3.369293485	0.37520951	8.979765695	2.71344E-19	8.80287E-18
MRPL17	140.2589794	1.744329918	0.194475886	8.969389253	2.98163E-19	9.64951E-18
LUM	34.14329897	8.436344813	0.941270551	8.962720448	3.16765E-19	1.02268E-17
GCLC	53.3649449	2.120479245	0.236765535	8.956030041	3.36578E-19	1.08403E-17
PCDH7	53.24448662	2.467895682	0.275828517	8.94721007	3.6458E-19	1.17139E-17
LGALS3	61.28238382	-3.019608863	0.337610866	-8.944051179	3.75159E-19	1.20249E-17
RPS19	989.7426478	-1.999737365	0.223744751	-8.937583386	3.97774E-19	1.27192E-17
FHL1	24.73874491	3.440866437	0.385189335	8.932922405	4.14901E-19	1.32352E-17
NCOA3	77.48535219	1.750136766	0.196025598	8.92810317	4.33375E-19	1.37917E-17
AZIN1	125.4192171	1.501111522	0.168152403	8.9270893	4.37364E-19	1.38855E-17
NIBAN1	100.1990177	2.260363429	0.253258106	8.92513754	4.45146E-19	1.40991E-17
FKBP11	151.132352	1.584664497	0.177734928	8.915886791	4.83928E-19	1.52912E-17
LOC101106554	297.6307532	1.285881784	0.144352671	8.907918211	5.19997E-19	1.63922E-17
RCN2	67.50486242	2.224733463	0.249886274	8.90298386	5.43651E-19	1.70975E-17
UBE2J1	117.104359	1.688990657	0.189803669	8.898619647	5.65455E-19	1.77415E-17
EIF4G2	313.559311	-1.447754307	0.162797669	-8.892967041	5.94985E-19	1.86243E-17
FAM162A	200.6323363	-1.654824392	0.186208738	-8.886932006	6.28194E-19	1.96179E-17
ATP5MC1	226.0024015	2.014778375	0.226751798	8.885390959	6.36964E-19	1.98454E-17
LYRM9	35.83031992	-2.582158886	0.29094248	-8.875152535	6.98376E-19	2.17081E-17
GHR	23.74676559	4.296325938	0.484167715	8.873631607	7.07985E-19	2.19557E-17
LOC101117272	39.1060455	2.684514959	0.302584043	8.871964726	7.18665E-19	2.22354E-17

PEAK1	38.17879801	2.78981529	0.314475645	8.87132386	7.22814E-19	2.23121E-17
DNAJC3	222.2815935	1.232539681	0.13912117	8.859468912	8.03965E-19	2.47599E-17
MEX3A	27.94509043	-2.948596391	0.333866622	-8.831659712	1.03134E-18	3.16893E-17
CTSZ	203.0050324	1.389048777	0.157410966	8.824345666	1.10102E-18	3.37527E-17
KEF53_p01	229.4086246	2.311386219	0.262039455	8.820756479	1.13689E-18	3.47727E-17
GUCY2C	28.00354463	3.604897943	0.408987012	8.8142113	1.20531E-18	3.67811E-17
MAGOH	84.51110996	-2.146141862	0.243831494	-8.801741824	1.34709E-18	4.10141E-17
TAPBPL	26.31814635	3.07802356	0.349744584	8.800775472	1.35874E-18	4.12748E-17
RTP3	25.04445631	4.029307271	0.458008853	8.797444081	1.39968E-18	4.24219E-17
AEN	44.22117151	-2.018241298	0.22943094	-8.796726811	1.40865E-18	4.25972E-17
ACTA2	30.93061784	5.98531258	0.68049142	8.795573909	1.42319E-18	4.29398E-17
LOC101107675	227.847335	2.500672739	0.284330283	8.79495743	1.43102E-18	4.30789E-17
KHSRP	209.9569249	-1.343907249	0.152826	-8.793708189	1.44703E-18	4.3463E-17
SLC5A3	95.6135789	2.39853695	0.273003721	8.785729888	1.55352E-18	4.6557E-17
HNRNPK	51.31261362	-2.51420887	0.286207135	-8.784577895	1.56953E-18	4.69313E-17
CD52	44.07186866	7.153649387	0.814834129	8.779270689	1.64538E-18	4.90896E-17
SNX19	24.24548068	4.156621375	0.473523832	8.778061626	1.66316E-18	4.95096E-17
LOC101111069	26.19804878	5.023989353	0.572392317	8.777178176	1.67627E-18	4.9789E-17
SMARCA2	117.5483304	1.801063683	0.205291657	8.773194717	1.73667E-18	5.14687E-17
LOC105614852	33.64137204	8.416856714	0.959528196	8.77187012	1.75723E-18	5.19627E-17
DDC	75.64475007	-2.009947232	0.22916402	-8.770780126	1.77433E-18	5.23524E-17
LOC101107687	87.45891677	2.068563646	0.235918161	8.768140786	1.81641E-18	5.3476E-17
MUC2	27.16023973	5.253508124	0.599440355	8.764021436	1.88406E-18	5.53458E-17
LOC101113965	29.02256765	8.209174613	0.936819456	8.762813962	1.90436E-18	5.58194E-17
LOC101120093	28.86177484	8.202603984	0.936889025	8.755150041	2.03832E-18	5.96153E-17
LOC114113424	88.21213574	1.869076632	0.213566631	8.75172598	2.10114E-18	6.13184E-17
GREM1	29.48582931	8.229287774	0.940650068	8.74851133	2.16186E-18	6.29529E-17
PPARGC1B	85.34638128	1.859713859	0.212590246	8.747879507	2.17399E-18	6.31687E-17
KEF53_p10	1360.808054	1.812068949	0.207291564	8.74164346	2.29744E-18	6.66107E-17
CD84	35.0947502	8.475379851	0.969811705	8.739201444	2.34764E-18	6.79189E-17
NEUROG3	26.53522207	-3.571367007	0.408689164	-8.738589924	2.36038E-18	6.79932E-17
RPL18A	214.5607669	-1.721248743	0.196965863	-8.738817553	2.35563E-18	6.79932E-17
ARID3A	75.47163278	2.116535511	0.242311103	8.734785511	2.44119E-18	7.01697E-17
LOC101119116_1	50.65948283	1.976913574	0.226457532	8.729732061	2.55277E-18	7.32195E-17
RPS16	490.9996521	-1.164394834	0.133489246	-8.722761326	2.71498E-18	7.77054E-17
RPS23	692.1790554	-1.292336372	0.148162453	-8.722428269	2.72298E-18	7.77679E-17
FYN	26.91157433	4.307431524	0.49449508	8.710767196	3.01825E-18	8.60169E-17
RGS5	33.22917527	6.393623462	0.73426918	8.707465377	3.10745E-18	8.83706E-17
DPF3	47.46055635	2.478705411	0.284781541	8.703883683	3.20716E-18	9.10125E-17
TENT5A	48.60370594	2.109639231	0.242466269	8.700753481	3.29688E-18	9.33603E-17
MPP1	76.70366134	1.874368065	0.215505105	8.697557588	3.39104E-18	9.58237E-17
SEPTIN9	77.59230511	1.543607822	0.177558063	8.693538287	3.51323E-18	9.90672E-17

SLC2A1	38.39820079	-2.780480288	0.320158948	-8.684687112	3.79786E-18	1.06868E-16
RGL1	34.8570963	6.473943872	0.745912539	8.679226492	3.98469E-18	1.1189E-16
FBXO32	47.39771941	3.606593596	0.415624387	8.677531233	4.04452E-18	1.13331E-16
CAVIN2	28.00319457	8.147790376	0.939666573	8.670937767	4.28576E-18	1.1984E-16
SSPN	41.17048774	8.123250852	0.936905283	8.670301044	4.30979E-18	1.2026E-16
RBMS1	125.9668606	1.465791489	0.16914572	8.665850287	4.48156E-18	1.24793E-16
SLC9A2	28.29824432	2.906316317	0.335407805	8.665022912	4.51422E-18	1.25441E-16
ATP5IF1	59.81721835	-2.491368082	0.287824477	-8.65585897	4.89213E-18	1.3566E-16
DNAL4	23.59335069	-3.184143053	0.368147826	-8.64908829	5.19125E-18	1.43657E-16
INPP5B	42.31084121	2.952018963	0.341503003	8.644196215	5.41854E-18	1.49637E-16
ANKRD37	22.39288449	-4.640396	0.536887026	-8.643151675	5.46833E-18	1.507E-16
IL7R	28.33121558	8.161539857	0.94570083	8.630149828	6.12717E-18	1.6851E-16
KRTCAP2	61.42040955	2.067386797	0.239842053	8.619784442	6.70803E-18	1.84106E-16
PDCL3	37.2365491	-2.390105079	0.27729925	-8.619226626	6.74078E-18	1.84626E-16
MPST	68.1642438	1.805194802	0.209571066	8.613759689	7.07028E-18	1.93254E-16
NID1	30.22403735	8.259419737	0.95903664	8.612204569	7.16688E-18	1.95495E-16
LOC101117285	121.5368397	1.572405035	0.182594491	8.611459319	7.21363E-18	1.9637E-16
CLEC2D	29.74192962	8.229061076	0.956161792	8.606347951	7.54251E-18	2.04905E-16
LOC132658625	23.39202244	4.24575824	0.493418686	8.604777968	7.64646E-18	2.07308E-16
MAT2B	112.0901051	1.511006991	0.175700277	8.599912422	7.9777E-18	2.1585E-16
GJB1	43.51895988	-2.708552464	0.315015836	-8.598146996	8.10135E-18	2.18753E-16
NFRKB	59.24554971	-2.001373678	0.232883159	-8.593896112	8.4069E-18	2.26546E-16
C7	38.9836591	8.049328065	0.936809093	8.592282164	8.52587E-18	2.29289E-16
NPM1	548.8021493	-1.512892428	0.176387153	-8.577112365	9.72846E-18	2.61106E-16
ACSS1	56.93353239	1.795858152	0.209581076	8.56879919	1.0457E-17	2.80096E-16
PDE5A	64.98061451	1.97919897	0.231132597	8.563045613	1.09924E-17	2.9385E-16
ZFP36L2	536.0493739	-1.013139041	0.118340355	-8.561230355	1.11669E-17	2.97919E-16
PLN	25.96632152	8.037068696	0.938954762	8.559590967	1.13269E-17	3.01583E-16
A2M	25.52584855	8.018269262	0.936984855	8.557522806	1.15319E-17	3.06431E-16
FUS	65.57902397	-2.735625475	0.32001261	-8.548492757	1.24706E-17	3.30719E-16
COG3	48.91481952	2.598450767	0.303992297	8.547752003	1.25509E-17	3.31743E-16
ZEB2	26.4910168	8.066778407	0.943739087	8.547678609	1.25589E-17	3.31743E-16
LOC101105090	28.47155397	-2.792239348	0.326714749	-8.546413506	1.26973E-17	3.34737E-16
PSME1	178.6558767	2.136197742	0.250165101	8.539151676	1.35211E-17	3.55753E-16
LOC101115328	43.03533396	7.6382551	0.894647646	8.537724472	1.36891E-17	3.59466E-16
LOC101119640	28.85363972	5.400743225	0.633084475	8.530841358	1.45287E-17	3.80766E-16
NAAA	39.30219457	2.399437569	0.281305431	8.529652499	1.46788E-17	3.83947E-16
PCYT2	52.96449053	2.295355326	0.269225798	8.525762908	1.51806E-17	3.96296E-16
DDOST	272.117503	-2.115536183	0.248169664	-8.524555952	1.53397E-17	3.9967E-16
PLEKHS1	21.26261349	-4.862207234	0.570525039	-8.522338026	1.56364E-17	4.06608E-16
ISX	26.53949362	8.07182803	0.947473673	8.519316427	1.60498E-17	4.16546E-16
DOCK8	24.89475012	3.721194435	0.436883416	8.517591418	1.62906E-17	4.21976E-16

RUNX3	24.91393615	7.986565779	0.938049987	8.514008731	1.68021E-17	4.34386E-16
FBLN1	25.61269756	5.465656552	0.642229564	8.510440596	1.73274E-17	4.471E-16
HMGN5	207.8045853	2.374056787	0.279072692	8.506947674	1.78572E-17	4.59264E-16
LOC121817553	116.1667276	2.013412363	0.236680454	8.506880605	1.78675E-17	4.59264E-16
RASSF3	101.6754212	2.112525408	0.248340612	8.506564378	1.79163E-17	4.59634E-16
GPATCH8	241.4957885	1.762786922	0.207234502	8.506242488	1.79661E-17	4.60029E-16
BMP3	27.4302771	5.543166834	0.651781779	8.504636081	1.82166E-17	4.65551E-16
GPC3	131.0587092	1.432411523	0.168445954	8.503686115	1.83664E-17	4.68483E-16
PCCA	26.72870561	2.725273297	0.320947005	8.49134983	2.04251E-17	5.19054E-16
TRAM2	45.94729435	2.24699995	0.264622509	8.491340966	2.04266E-17	5.19054E-16
CMIP	410.1231787	1.213788114	0.142985171	8.488909089	2.08586E-17	5.29024E-16
LOC101104787	36.41403592	-2.455437595	0.289425574	-8.483830771	2.17899E-17	5.51597E-16
SFRP1	196.4861036	2.006991054	0.236788503	8.475880507	2.33308E-17	5.89489E-16
FLNA	183.5885779	1.95163064	0.230389737	8.470996413	2.43303E-17	6.13582E-16
GRK5	36.51072184	2.498249481	0.295132147	8.464850416	2.56482E-17	6.43177E-16
PIGN	62.74111314	1.845912419	0.218061295	8.465108043	2.55915E-17	6.43177E-16
VNN2	52.36566532	-2.871316098	0.339204406	-8.464854968	2.56472E-17	6.43177E-16
LOC132659429	80.76865115	1.733013858	0.2048164	8.461304145	2.64404E-17	6.61802E-16
FHIP1B	114.7262394	-1.995652785	0.235939097	-8.458338663	2.71214E-17	6.77578E-16
TCERG1	113.6495884	-1.388063716	0.164144599	-8.456347202	2.75883E-17	6.87958E-16
BTBD6	24.72053721	-3.045161264	0.360159737	-8.455029669	2.79016E-17	6.94475E-16
SPART	48.83772938	2.164720363	0.256034508	8.454799227	2.79568E-17	6.94555E-16
NPNT	43.75530704	2.191501905	0.259237237	8.453653985	2.82325E-17	7.00104E-16
TFF3	94.31933036	2.047232469	0.24232432	8.448316148	2.95534E-17	7.31502E-16
LOC105605730	29.84529639	8.238717857	0.975339675	8.447024215	2.98822E-17	7.38272E-16
LOC114112489	431.6704416	2.300371767	0.272408552	8.444565156	3.05179E-17	7.51202E-16
NARS1	208.4692101	-1.85800086	0.220018308	-8.444755691	3.04682E-17	7.51202E-16
TIMM13	150.6630958	-1.449166822	0.171766393	-8.436847281	3.26012E-17	8.01007E-16
WWC1	148.3691916	-1.761709576	0.208947508	-8.431350008	3.417E-17	8.38012E-16
PPP1R12A	290.1130413	1.931907454	0.229293325	8.425484909	3.59259E-17	8.79461E-16
LOC132657302	40.57981485	7.540802429	0.89505071	8.425000221	3.6075E-17	8.81495E-16
NDUFS2	133.1628366	-1.793105826	0.212895885	-8.422454131	3.68679E-17	8.99226E-16
RPS3	418.3917649	-1.186763079	0.140955969	-8.419388622	3.78454E-17	9.21387E-16
ZNF217	85.82945941	1.580270079	0.187731633	8.417708058	3.83921E-17	9.32998E-16
HNRNPD	256.4264323	-1.287729947	0.153026264	-8.415091068	3.9259E-17	9.52333E-16
ARHGEF28	72.59233187	-1.767117362	0.210015462	-8.414225064	3.95501E-17	9.57656E-16
TCEAL9	56.9200873	2.01531651	0.239523568	8.413854747	3.96752E-17	9.58949E-16
MYCBP2	173.2401161	1.495089158	0.177874953	8.405282094	4.26834E-17	1.0298E-15
LOC101112891	24.07253841	7.929587918	0.944334998	8.397007351	4.58001E-17	1.103E-15
LRRFIP1	256.768408	1.456900231	0.173514929	8.396396989	4.60387E-17	1.10675E-15
AK4	21.24351444	-3.308441489	0.394089041	-8.395162376	4.65251E-17	1.11643E-15
OTOP3	30.30992534	5.684575153	0.677272208	8.393338881	4.72527E-17	1.13186E-15

TRAP1	34.92742694	-2.788088644	0.332471396	-8.385950419	5.03175E-17	1.20312E-15
LRRK1	70.12346059	1.996723485	0.238128278	8.38507505	5.06934E-17	1.20994E-15
COL6A1	24.52810958	7.955388505	0.94900009	8.382916488	5.16321E-17	1.23015E-15
RASGRP3	23.40541029	7.897195757	0.943613293	8.369101853	5.80591E-17	1.38081E-15
FAM98B	69.57775007	-1.647220422	0.196887358	-8.366308717	5.94513E-17	1.41141E-15
EPB41	214.8258785	1.278875024	0.152868959	8.365825405	5.96955E-17	1.4147E-15
MRC1	25.03423149	7.983142741	0.954346461	8.365036247	6.00964E-17	1.42168E-15
RPL11	436.1955808	-1.239374841	0.148175348	-8.364244484	6.05013E-17	1.42873E-15
CCL19	25.2097131	8.003560048	0.956988745	8.363274999	6.10008E-17	1.43798E-15
RNF13	40.93271053	1.990772642	0.238100388	8.361064266	6.2155E-17	1.46261E-15
TFF2	343.5285638	8.272403528	0.990100527	8.355114759	6.53691E-17	1.53554E-15
TUBB4B	118.5829397	-2.014767558	0.241179166	-8.353820917	6.60895E-17	1.54974E-15
KDM4A	57.31587406	-2.184974837	0.261599814	-8.352356245	6.69145E-17	1.56634E-15
LOC105606144	179.6325466	10.83504442	1.297384209	8.351453906	6.74278E-17	1.57559E-15
CXCL14	23.24572713	7.886739457	0.945564842	8.34077062	7.38079E-17	1.72167E-15
TKT	384.9880787	-1.923258944	0.230617886	-8.339591423	7.45477E-17	1.7359E-15
NCOA1	108.7346368	1.371007864	0.164592673	8.329701656	8.10463E-17	1.88394E-15
HPS5	121.931505	2.031377866	0.24392474	8.327887807	8.22974E-17	1.9097E-15
TICAM1	30.17328068	-2.839166122	0.340953892	-8.3271263	8.28284E-17	1.91869E-15
NOC2L	32.51153978	-2.32189509	0.278938917	-8.324027062	8.50242E-17	1.96615E-15
LOC132658164	36.25625555	7.9412189	0.954646636	8.318490423	8.90907E-17	2.05663E-15
CREBL2	28.35626216	2.738210669	0.329519093	8.309717797	9.59299E-17	2.21069E-15
KIF1C	146.1561954	-1.821982992	0.219552225	-8.298631422	1.05318E-16	2.42285E-15
KLHL24	93.86684095	2.036282549	0.245494194	8.294626099	1.08927E-16	2.50158E-15
ADGRF5	22.72119908	3.54430756	0.427341551	8.293851955	1.09639E-16	2.51361E-15
RCC2	67.15649662	-1.866109208	0.22528542	-8.283311031	1.19797E-16	2.74179E-15
ARID1A	70.1031163	1.648543861	0.199077521	8.280914148	1.22233E-16	2.79277E-15
SRSF5	169.9841612	-1.353849356	0.163511424	-8.279845658	1.23335E-16	2.81313E-15
HSP90B1	790.6964952	1.387485698	0.167619964	8.277568272	1.25716E-16	2.86256E-15
LOC114115312	56.53283511	-1.684156168	0.203568745	-8.273156902	1.30458E-16	2.96548E-15
KIRREL1	21.80128215	4.615408186	0.557973269	8.271737093	1.32021E-16	2.99592E-15
ECM2	23.51520274	7.897277323	0.95496382	8.269713634	1.34281E-16	3.04204E-15
C5AR1	32.0513996	2.562553404	0.309951333	8.267599237	1.36683E-16	3.09122E-15
WFDC1	21.91570275	7.795772356	0.943032464	8.266706237	1.37711E-16	3.10919E-15
ZBTB8OS	30.49593578	2.566839491	0.310677203	8.262078657	1.43157E-16	3.22671E-15
MYO1A	39.93099999	2.206183765	0.267051754	8.26125922	1.44144E-16	3.24348E-15
LMOD1	28.13566941	8.149337977	0.986513724	8.26074466	1.44766E-16	3.25201E-15
ZBTB16	27.80698508	6.127862933	0.742098011	8.257484651	1.48774E-16	3.33644E-15
EML2	58.07998457	-1.97667775	0.239398085	-8.256865327	1.49548E-16	3.34817E-15
DQA	21.80301813	7.784534941	0.944108146	8.245384782	1.6463E-16	3.67966E-15
IL32	37.52405412	3.25896749	0.395369869	8.242832215	1.68181E-16	3.75277E-15
LOC101117786	61.95916828	-1.822306655	0.22111149	-8.241573748	1.6996E-16	3.78614E-15

DMBT1	1415.506316	1.322447809	0.160562444	8.236345807	1.7755E-16	3.94865E-15
CXCL9	31.70636866	7.752479786	0.941822343	8.231361086	1.85098E-16	4.108E-15
MYO15B	70.44046991	2.08020357	0.252721454	8.231210844	1.8533E-16	4.108E-15
LOC101109388	22.15174002	7.812768636	0.949220988	8.230716274	1.86097E-16	4.11817E-15
BACH1	44.94133138	2.104611381	0.255742793	8.229406417	1.88143E-16	4.15657E-15
PDK4	31.9673315	3.945136197	0.479538997	8.226935087	1.92064E-16	4.23619E-15
MCAM	22.3692822	7.817184121	0.950319889	8.225845017	1.93819E-16	4.26084E-15
RPL3	809.8453058	-1.32234912	0.160751765	-8.22603171	1.93518E-16	4.26084E-15
SLC20A1	50.3003057	-1.749270816	0.212723717	-8.223205404	1.98135E-16	4.34856E-15
ALDOC	20.34996239	-3.304058369	0.402178136	-8.215410213	2.1144E-16	4.63297E-15
GALM	99.54378093	-2.131227319	0.259433049	-8.214941496	2.12268E-16	4.64349E-15
LOC101104893	22.58179989	7.842205492	0.954665386	8.214611745	2.12852E-16	4.64866E-15
PPIA	131.9724259	-1.901847219	0.231641525	-8.210303493	2.2063E-16	4.81067E-15
VCAN	31.86398959	7.762987625	0.945900928	8.206977492	2.26826E-16	4.93772E-15
TRAF3IP3	22.00696078	7.791133294	0.949521322	8.205327371	2.29963E-16	4.99787E-15
PTMS	135.9051408	3.861412156	0.470714454	8.203300589	2.33875E-16	5.07464E-15
LTBP1	25.00044754	3.570913292	0.435456222	8.200395607	2.39597E-16	5.19037E-15
CWF19L2	264.907947	1.800184093	0.219572191	8.198597838	2.43207E-16	5.26004E-15
LOC114117126	33.67650976	2.537481899	0.309773111	8.191420795	2.5816E-16	5.56544E-15
SDC1	202.9041723	-1.571312184	0.19182412	-8.191421312	2.58159E-16	5.56544E-15
GOLIM4	94.7850096	1.612206336	0.197078517	8.180528037	2.82603E-16	6.08256E-15
LOC101123290	21.52903654	7.762519518	0.949475062	8.175590731	2.9442E-16	6.32671E-15
NDUFV1	52.12691303	1.96766487	0.24073443	8.17359142	2.99342E-16	6.42217E-15
IGFBP3	80.98240924	2.110680884	0.258272876	8.172290164	3.0259E-16	6.48143E-15
RORA	185.0782377	1.514667492	0.185509515	8.164904607	3.21689E-16	6.87952E-15
SPICE1	74.90716528	-1.659390959	0.203243251	-8.164556276	3.22619E-16	6.88838E-15
CST6	27.54625899	-2.793179371	0.342256811	-8.161062926	3.32089E-16	7.07927E-15
BIRC3	131.8222329	1.339752413	0.164246789	8.156947374	3.43598E-16	7.31295E-15
PGM5	20.5386489	7.700865153	0.945829034	8.14192087	3.89056E-16	8.26727E-15
STOML2	87.585389	-1.877983749	0.230706768	-8.140132886	3.94844E-16	8.37697E-15
HIC2	37.37695376	-2.843227324	0.349320556	-8.139307209	3.97546E-16	8.42092E-15
DOT1L	50.5452379	-2.237502907	0.275368825	-8.125476466	4.45608E-16	9.42405E-15
LRP8	97.4403474	-1.79650285	0.22111416	-8.124775245	4.48192E-16	9.46372E-15
LOC101104237	48.7351295	-2.768736685	0.341157277	-8.115719263	4.82916E-16	1.01808E-14
ARHGAP17	68.60059184	1.697093583	0.209121176	8.115359785	4.84348E-16	1.01949E-14
XRN2	37.45534354	-2.394459298	0.295066116	-8.114992418	4.85815E-16	1.02098E-14
POLR1C	28.31402646	-2.53968611	0.313083678	-8.111844487	4.98571E-16	1.04614E-14
KLF12	31.07770648	7.717708372	0.951642484	8.109882127	5.06689E-16	1.06151E-14
LOC101106528	21.00550099	7.730184857	0.953597228	8.106341576	5.21667E-16	1.09117E-14
C1QA	20.26727401	7.681556218	0.947879142	8.10394055	5.32071E-16	1.1112E-14
GOLGB1	508.3777512	1.259720988	0.155457588	8.103309739	5.34838E-16	1.11523E-14
RAB8B	37.99367244	2.119111294	0.26187214	8.092160133	5.86158E-16	1.22034E-14

RPS27A	869.6416246	-1.607193326	0.19875227	-8.086414931	6.14466E-16	1.27729E-14
MOCS2	104.9827664	1.473550568	0.1822828	8.083870591	6.27429E-16	1.30221E-14
ATOX1	29.91059423	2.342314771	0.290083318	8.074627609	6.76829E-16	1.40256E-14
ANAPC16	35.86388933	2.196214513	0.272063865	8.072422666	6.89168E-16	1.42592E-14
CD59	39.45944615	2.105552904	0.260991136	8.067526493	7.17366E-16	1.48197E-14
UTRN	323.7160894	1.132157564	0.140341898	8.067138769	7.19647E-16	1.48438E-14
NEMF	216.0292917	1.65845987	0.205735672	8.061119639	7.55988E-16	1.55694E-14
RUNX2	19.59543472	4.412808752	0.547442959	8.06076447	7.58188E-16	1.55907E-14
ABRACL	45.38311046	-2.89752444	0.359494783	-8.059990238	7.63005E-16	1.56656E-14
AHCYL2	22.64398211	5.033944064	0.6246056	8.059396299	7.66722E-16	1.57178E-14
RBM25	1002.549738	1.748347469	0.217077636	8.054019302	8.01186E-16	1.63992E-14
ABCC5	37.2022762	-2.433276343	0.302314874	-8.048814508	8.35998E-16	1.70856E-14
MAP1A	29.97990346	6.247517879	0.776353446	8.047259805	8.46683E-16	1.72775E-14
SORD	67.9696732	2.308683863	0.287364507	8.033991006	9.43521E-16	1.92242E-14
C14H19orf33	513.3462513	1.539646538	0.191822964	8.026393224	1.0038E-15	2.0416E-14
PDGFRA	26.93824859	7.517801374	0.936653096	8.026238753	1.00507E-15	2.0416E-14
PLPP3	24.57525389	5.959880731	0.742717779	8.02442179	1.02005E-15	2.0689E-14
SREK1	395.003826	1.789287352	0.222997555	8.023798086	1.02525E-15	2.07628E-14
PRRC2C	937.1693468	1.637286207	0.204109237	8.021617416	1.04362E-15	2.11029E-14
RPL7	640.511016	-1.321731194	0.164789265	-8.020736016	1.05113E-15	2.12227E-14
POU2F2	22.42315382	5.51316013	0.687502511	8.019112719	1.06512E-15	2.14726E-14
OAF	47.81289194	-2.523560056	0.314780348	-8.01689202	1.08454E-15	2.18313E-14
LOC101119773	19.56723517	7.642684261	0.953449397	8.015825789	1.09399E-15	2.19885E-14
TMF1	118.4935704	1.43819523	0.179425368	8.015562384	1.09634E-15	2.20026E-14
FCGRT	103.1575796	-1.560499346	0.194711759	-8.01440731	1.10669E-15	2.2177E-14
PDIA4	372.8069917	1.41215442	0.176222422	8.013477534	1.1151E-15	2.23119E-14
HCLS1	18.97887634	7.592306603	0.948173835	8.007293937	1.1726E-15	2.34275E-14
RPL12	1065.253583	-1.020150302	0.127428378	-8.005675986	1.18812E-15	2.37021E-14
SDF2L1	242.7576137	1.530421867	0.191246073	8.002370174	1.22047E-15	2.43111E-14
HHIP	21.05547065	7.734992895	0.966650807	8.001848069	1.22566E-15	2.43781E-14
NDRG1	65.8273499	-2.424836496	0.303126957	-7.999408959	1.25018E-15	2.4829E-14
DDR2	27.14631789	6.492730656	0.811718912	7.998742622	1.25696E-15	2.49266E-14
SOX4	374.4902467	-1.732337133	0.21661327	-7.997373062	1.27102E-15	2.5168E-14
DGKQ	65.58755069	-2.162180436	0.270380361	-7.996810216	1.27684E-15	2.52459E-14
TMEM120A	78.31015732	2.074732703	0.259563867	7.993149145	1.31535E-15	2.59689E-14
MLEC	483.9440979	1.270933898	0.159374755	7.97449946	1.53E-15	3.01621E-14
BNIP3	20.72067008	-3.047643622	0.382378683	-7.97022365	1.58387E-15	3.11783E-14
TLE3	100.2245551	1.649035571	0.206985177	7.966925927	1.6267E-15	3.19742E-14
TK1	55.56032448	-2.544567538	0.31946496	-7.965091186	1.65102E-15	3.24046E-14
DHRS11	30.10334827	2.471530459	0.310325474	7.964317026	1.66139E-15	3.25603E-14
DACT3	20.23550134	7.676749303	0.964171481	7.962016569	1.69258E-15	3.3123E-14
TMEM65	95.99196572	2.004804718	0.251813743	7.961458703	1.70023E-15	3.3224E-14

STK17A	79.80230065	1.755864883	0.220561155	7.960898103	1.70795E-15	3.33262E-14
C1R	22.39244226	5.531057066	0.694803736	7.960603523	1.71202E-15	3.3357E-14
LOC101112716	30.63961702	-6.798935749	0.854363423	-7.957896568	1.74989E-15	3.40451E-14
PDE1A	19.73217132	7.649263687	0.961371548	7.95661542	1.76809E-15	3.43493E-14
SPARC	18.29289796	7.544450146	0.949647684	7.944472747	1.95018E-15	3.78319E-14
APOB	20.12433045	7.670147163	0.965692562	7.942638749	1.97925E-15	3.834E-14
C1S	35.05061368	7.320364521	0.921738572	7.94190972	1.99092E-15	3.85103E-14
RPL35A	148.0942803	-1.547286919	0.195002797	-7.934690899	2.1102E-15	4.07586E-14
DOCK10	18.57018174	7.556385869	0.952833495	7.93043686	2.18376E-15	4.21186E-14
LOC101102809	342.2908477	-1.639615871	0.206814455	-7.927955875	2.22782E-15	4.29065E-14
DUT	214.7861042	-1.26708762	0.159854326	-7.926514407	2.25383E-15	4.33448E-14
FUBP1	252.6531695	-1.160307142	0.146389462	-7.926165741	2.26016E-15	4.34041E-14
NBEAL1	141.3960921	1.35465813	0.170936755	7.924908448	2.28314E-15	4.37827E-14
LIMK1	18.72244908	-3.393399411	0.428481982	-7.919584835	2.38305E-15	4.5633E-14
SLIT3	18.18112611	7.518873892	0.949478217	7.918953546	2.39518E-15	4.57996E-14
ETS1	106.2940789	1.532618762	0.193632501	7.915090472	2.47073E-15	4.71262E-14
TOX2	18.16297997	7.527216729	0.95100094	7.915046571	2.47161E-15	4.71262E-14
PLEKHA3	74.95904702	1.54174318	0.195076329	7.903281712	2.71655E-15	5.17227E-14
CPEB3	31.28952871	2.431300847	0.308067143	7.892113471	2.97111E-15	5.64892E-14
ATF4	620.7033367	-1.252559517	0.158761305	-7.889576837	3.03213E-15	5.75674E-14
KDM1A	107.6675638	-1.318040289	0.167148469	-7.885446358	3.13413E-15	5.94196E-14
PTN	17.99291203	7.511364971	0.953248786	7.879752985	3.28029E-15	6.21025E-14
PIK3CD	23.92126072	5.874994625	0.74663008	7.868681943	3.58397E-15	6.77558E-14
TRIP13	19.36044276	-3.145918117	0.399980923	-7.865170401	3.68595E-15	6.95854E-14
PIK3AP1	34.2547335	2.412296685	0.306802407	7.86270457	3.75927E-15	7.08694E-14
NUCB2	125.8103185	1.777263598	0.226167875	7.85816111	3.89814E-15	7.33838E-14
PLXNC1	19.65800261	5.034814202	0.640832384	7.856678796	3.94453E-15	7.41527E-14
SUSD6	55.23296851	1.899219325	0.241750232	7.856122049	3.96209E-15	7.43782E-14
LMO7	71.58268643	1.667360972	0.212267648	7.854993402	3.99793E-15	7.49458E-14
APBB1IP	26.56705211	7.487906572	0.953338471	7.854405129	4.01674E-15	7.51929E-14
ADAM19	18.02699829	7.517632988	0.958615013	7.842181569	4.42785E-15	8.2773E-14
CDK14	17.6534521	7.480418986	0.954750646	7.834945195	4.69047E-15	8.75599E-14
LOC105606646	18.02129657	7.525228106	0.960543048	7.834347582	4.71283E-15	8.78546E-14
MICALL2	52.86424358	2.026699842	0.258815853	7.830663462	4.85302E-15	9.03419E-14
FCER1G	17.64476246	7.479254061	0.955434812	7.828115498	4.95237E-15	9.20631E-14
BCAP31	186.4988808	1.656114807	0.211748075	7.821156362	5.23403E-15	9.71639E-14
OTC	25.98958358	2.804301723	0.3589143	7.813290581	5.57139E-15	1.03283E-13
PECAM1	25.52175421	7.424793586	0.950350028	7.812693606	5.59786E-15	1.0363E-13
VEGFB	25.04397275	3.647730108	0.467076584	7.809704524	5.73222E-15	1.05971E-13
FBN1	16.85254765	7.429347822	0.951489969	7.808119964	5.80473E-15	1.07163E-13
PCP4	18.22265562	7.514901543	0.962501754	7.80767569	5.82522E-15	1.07393E-13
LOC101123672	18.15398877	4.122530434	0.528219706	7.804575228	5.97023E-15	1.09915E-13

TACC3	28.55583387	-2.518810384	0.322743347	-7.804375848	5.97967E-15	1.09937E-13
TUT7	280.7649256	1.375724802	0.176478101	7.795441996	6.41834E-15	1.1784E-13
CYTH3	26.76623863	2.417535491	0.3101394	7.794996342	6.44103E-15	1.18094E-13
LOC121816219	19.33020401	3.752951997	0.481469259	7.794790483	6.45154E-15	1.18125E-13
NLRC3	16.83430747	7.411863955	0.950991745	7.79382576	6.50102E-15	1.18868E-13
SAMD4A	17.76037974	3.744875431	0.480508802	7.793562605	6.51458E-15	1.18953E-13
LOC101113604	37.94716631	-2.262408142	0.29034477	-7.7921436	6.58818E-15	1.20133E-13
MRPS7	23.09265253	-2.698095356	0.346304936	-7.791097033	6.64299E-15	1.20968E-13
MRPS18B	37.33059966	-2.150256348	0.276332786	-7.781401461	7.17255E-15	1.30433E-13
LOC101110545	19.47916094	7.62275681	0.979961937	7.778625397	7.33168E-15	1.33146E-13
LOXL3	25.43094547	2.844225788	0.365868692	7.773897719	7.61072E-15	1.38026E-13
SLCO3A1	18.37301643	4.693616031	0.603997934	7.770914047	7.79218E-15	1.41125E-13
LOC101110066	52.82655251	-2.867947685	0.369517387	-7.76133353	8.4041E-15	1.52002E-13
SLC27A4	24.01630966	3.652913661	0.470676329	7.76098868	8.42698E-15	1.5221E-13
TRPM2	16.59974872	7.388285715	0.952202206	7.759156265	8.54963E-15	1.54216E-13
TNFSF10	23.74379929	7.334769129	0.94562931	7.756495121	8.73087E-15	1.57273E-13
CDC123	91.03184661	-1.7718521	0.228649198	-7.749216319	9.24614E-15	1.66331E-13
MAFA	17.01252312	3.766686606	0.486400656	7.743999842	9.63371E-15	1.7307E-13
IRAG1	16.33225494	7.364145655	0.951324505	7.74093973	9.86847E-15	1.7705E-13
STC2	25.08845724	-3.700058932	0.478009335	-7.740557903	9.89815E-15	1.77344E-13
CLDN2	210.2784223	-1.302366132	0.168351698	-7.73598452	1.02606E-14	1.83592E-13
PAPPA	16.43892393	7.376748754	0.953724653	7.734673448	1.03669E-14	1.85246E-13
CCK	17.37593889	3.912973607	0.50636131	7.727631495	1.09566E-14	1.95522E-13
PGK1	256.0988348	-1.1881149	0.153803872	-7.724869885	1.11968E-14	1.99541E-13
CALB2	23.0378841	-2.939879721	0.380614003	-7.724045091	1.12695E-14	2.0057E-13
ME2	69.32371093	1.640112387	0.212343659	7.72385854	1.1286E-14	2.00597E-13
NRP2	21.47323667	3.776675353	0.4892122	7.719912448	1.1641E-14	2.06631E-13
LOC101117691	122.3854021	-3.476895491	0.450416613	-7.719287857	1.16981E-14	2.07371E-13
PRMT7	25.11545943	3.059642682	0.396514562	7.7163438	1.19714E-14	2.11934E-13
C20H6orf132	68.83696648	-1.781962541	0.230958196	-7.715519817	1.2049E-14	2.13025E-13
TNXB	17.07704669	7.425303922	0.962578463	7.713972638	1.21961E-14	2.1534E-13
LOC101109907	15.82431972	7.322284862	0.94931234	7.713251533	1.22652E-14	2.16275E-13
ALYREF	56.33443993	-2.082084367	0.269962019	-7.712508507	1.23368E-14	2.17252E-13
TUBB	232.7463492	-1.398247042	0.181314956	-7.711702737	1.2415E-14	2.18341E-13
ANTXR1	21.24892741	5.406044815	0.701273935	7.708891697	1.26915E-14	2.2289E-13
HEXB	76.52423669	-2.036008889	0.264117107	-7.708735382	1.27071E-14	2.2289E-13
NPEPPS	249.0568288	-1.253458602	0.162629239	-7.707461542	1.28345E-14	2.24831E-13
FABP5	74.14772821	-1.492445632	0.193709347	-7.704561801	1.31293E-14	2.29547E-13
THOC2	272.2118831	1.285957639	0.166910444	7.704476757	1.3138E-14	2.29547E-13
ZBTB20	376.5673501	1.231724619	0.159951534	7.700611461	1.35417E-14	2.3629E-13
PDE4B	16.99625496	4.056336271	0.526814094	7.699748962	1.36334E-14	2.3758E-13
PRKCQ	28.80561441	7.031949828	0.913337266	7.699181986	1.3694E-14	2.38326E-13

RPL31	533.5449338	-1.79142138	0.232828035	-7.694182433	1.42402E-14	2.47509E-13
MAB21L4	69.3388943	-1.979737724	0.257329184	-7.69340536	1.4327E-14	2.48694E-13
ITPR1	18.01844527	4.132386427	0.537186294	7.692650522	1.44118E-14	2.49842E-13
GFRA4	16.30692079	7.366742654	0.95785573	7.6908687	1.46139E-14	2.53018E-13
RHOH	26.08854561	6.901582623	0.898128081	7.684408016	1.53706E-14	2.65774E-13
KAT6B	84.61154685	1.897262683	0.247257528	7.67322514	1.67725E-14	2.89639E-13
RPS28	1326.549065	-1.280968813	0.166974972	-7.671621653	1.69835E-14	2.92906E-13
INPP5D	22.3864061	7.23895205	0.943919346	7.669036642	1.73293E-14	2.98484E-13
NOB1	46.47664639	-2.339154204	0.305168039	-7.665134966	1.78643E-14	3.07303E-13
TGIF2	28.69505502	-2.340873219	0.305421678	-7.664397732	1.79672E-14	3.08676E-13
ARHGAP1	40.40315866	-2.075105096	0.270770138	-7.663714747	1.80631E-14	3.09925E-13
ALDOA	323.1895777	-1.446197681	0.188711512	-7.663537146	1.80881E-14	3.09956E-13
KMT5B	108.3423058	1.623178841	0.211847183	7.662027027	1.83021E-14	3.13222E-13
AIF1	22.30311126	7.237643454	0.945052169	7.658459178	1.88177E-14	3.21634E-13
ERAP1	58.76509641	1.748675833	0.228402101	7.656128499	1.91622E-14	3.27103E-13
PACS2	24.49317558	-2.724285472	0.355880544	-7.655055947	1.93228E-14	3.29424E-13
PTGIS	15.66396441	7.301582903	0.953925459	7.654248911	1.94445E-14	3.31077E-13
TMEM86B	21.30586683	4.507346046	0.588981923	7.652774846	1.96688E-14	3.3447E-13
ZNF655	82.01060904	1.775145819	0.232099367	7.648214827	2.03789E-14	3.46104E-13
ARGLU1	885.0525748	1.65501513	0.216623568	7.640051106	2.17136E-14	3.68304E-13
DENND2D	28.01249323	2.271982305	0.29740246	7.639420008	2.18202E-14	3.69644E-13
TBC1D10C	16.81073994	7.402251377	0.969918408	7.631828943	2.31446E-14	3.91584E-13
SASH3	16.81960488	7.408836058	0.971453858	7.626544477	2.4113E-14	4.07451E-13
ANGPTL8	13.41240572	-7.320405103	0.960466971	-7.621714564	2.50328E-14	4.2246E-13
BCR	52.250335	2.144659199	0.281436904	7.620390823	2.52909E-14	4.26277E-13
PIK3R3	56.73635093	-1.677544644	0.220160166	-7.619655604	2.54353E-14	4.28172E-13
AMPD3	22.50263254	5.170004951	0.678854838	7.615773886	2.62116E-14	4.40685E-13
LOC132657225	21.67403107	7.195780918	0.945640261	7.609427407	2.75313E-14	4.6229E-13
LDHB	56.83139602	-1.637633034	0.215245376	-7.608214683	2.77908E-14	4.66062E-13
CXXC5	32.08618969	-2.083785375	0.274040983	-7.603918778	2.87296E-14	4.81202E-13
MAP3K2	106.4133278	1.180643853	0.155350327	7.599880039	2.96405E-14	4.95839E-13
SGK1	22.24579046	6.198287185	0.815606372	7.599606134	2.97033E-14	4.96269E-13
PHLDB3	21.13685019	-2.859734173	0.37643961	-7.596794013	3.03557E-14	5.06535E-13
POLR3GL	82.41341673	1.575046306	0.207367408	7.595438078	3.06753E-14	5.11229E-13
JAG1	26.978106	2.656017944	0.349785801	7.593269748	3.11932E-14	5.19214E-13
PLPP2	179.9649867	-1.160994132	0.152907028	-7.592810783	3.1304E-14	5.20409E-13
ZBTB40	39.39215722	2.333093856	0.307325014	7.591617187	3.15937E-14	5.24574E-13
LOC101102403	27.48614758	2.563483096	0.338142125	7.58108175	3.42686E-14	5.6828E-13
LOC105606567	166.7645554	1.365497478	0.180166244	7.579097199	3.47968E-14	5.76324E-13
STK19	48.72696832	-1.850965654	0.244313719	-7.576183871	3.55867E-14	5.88679E-13
LOC114111549	161.2294058	2.352089443	0.310490161	7.575407342	3.58003E-14	5.91479E-13
GLRX5	36.23197847	2.289602395	0.302261158	7.574914392	3.59365E-14	5.92996E-13

ATP6AP1	68.85049999	-1.748675055	0.230973352	-7.570895253	3.70661E-14	6.10882E-13
GUCY1B1	15.26858163	7.265254749	0.959695975	7.570371181	3.72159E-14	6.12596E-13
ARHGAP6	15.87268518	7.316829212	0.966619375	7.569503988	3.74652E-14	6.15941E-13
C18H14orf132	20.15030815	-2.753053543	0.363841246	-7.566634003	3.83019E-14	6.28923E-13
DUSP4	75.28565634	-2.076168496	0.274498334	-7.563501271	3.92362E-14	6.43474E-13
GLB1	82.19683339	-1.600235296	0.212517895	-7.529884921	5.07851E-14	8.31604E-13
KCNMB1	14.48187825	7.194698465	0.955501147	7.52976434	5.0832E-14	8.31604E-13
TMEM183A	62.60922713	1.695241173	0.22521149	7.527329848	5.17884E-14	8.46215E-13
PFKFB3	36.92534898	-3.169340294	0.421258454	-7.523505504	5.33268E-14	8.70287E-13
CSRP1	49.98446824	2.263733281	0.300914313	7.522850139	5.35948E-14	8.73596E-13
ZNF703	42.36101442	-1.868747838	0.24845794	-7.52138506	5.4199E-14	8.82367E-13
IRF1	20.78836577	2.743345175	0.36485656	7.518969027	5.52099E-14	8.97731E-13
TPM1	230.5580391	1.781507279	0.236978721	7.517583314	5.5798E-14	9.06192E-13
PSMD3	82.01670194	-1.557627553	0.207232124	-7.516342189	5.633E-14	9.13722E-13
DAAM2	21.07126918	7.15206757	0.95182796	7.514033914	5.73327E-14	9.2886E-13
TAOK1	178.664805	1.54716385	0.20602895	7.509448793	5.93768E-14	9.60812E-13
PURA	109.1843262	1.253575135	0.16700556	7.506188014	6.0874E-14	9.83847E-13
ING5	39.58242517	1.956701958	0.260695135	7.50570953	6.10968E-14	9.86255E-13
FLNC	22.61210016	7.248044537	0.965721151	7.505318209	6.12796E-14	9.88013E-13
CA9	16.04701898	-3.665563457	0.48841264	-7.505054447	6.14031E-14	9.88811E-13
C15H11orf96	18.87986885	4.473437526	0.596647326	7.497624365	6.49848E-14	1.04523E-12
LRP4	35.47940456	-2.342487818	0.313114489	-7.481250155	7.36189E-14	1.18268E-12
GJA1	21.46910326	6.145482563	0.822120963	7.475156138	7.71122E-14	1.23731E-12
CANX	899.3448049	0.85191556	0.114000447	7.472914176	7.8438E-14	1.25708E-12
EIF3D	149.8096989	-1.43647877	0.192365965	-7.467426848	8.17782E-14	1.30904E-12
SLC16A5	31.66933161	2.126552751	0.284963562	7.462542703	8.48685E-14	1.35688E-12
LOC114113983	23.16872534	-3.066666278	0.411132716	-7.459066517	8.71376E-14	1.3915E-12
VDAC3	26.67538184	-2.191865246	0.293944494	-7.45673176	8.8695E-14	1.41467E-12
DEPTOR	19.6476479	2.955593142	0.396452263	7.45510473	8.97965E-14	1.43054E-12
CHMP5	59.15413628	-1.878637408	0.252082597	-7.45246769	9.16103E-14	1.45769E-12
TAF3	72.17443159	1.877901144	0.252476509	7.437924231	1.0228E-13	1.62553E-12
BASP1	19.82265515	3.559517829	0.47869812	7.435829967	1.03913E-13	1.64953E-12
SLC13A2	15.67636966	7.315958269	0.984636819	7.430108371	1.08509E-13	1.72044E-12
KLF5	132.2142982	1.760478318	0.237108051	7.424793519	1.12956E-13	1.78883E-12
CELF3	18.78429838	-7.200855421	0.969942104	-7.4240054	1.13631E-13	1.79738E-12
RSRC2	444.8306468	1.501062004	0.202239379	7.422204375	1.15187E-13	1.81984E-12
MRPL11	65.96555267	-1.561483659	0.210414501	-7.420988819	1.16249E-13	1.83446E-12
GLIPR2	17.87719768	3.181633412	0.428755744	7.420619905	1.16573E-13	1.83741E-12
FGL2	19.51014788	7.030749442	0.947956285	7.416744375	1.20034E-13	1.88973E-12
ATXN2L	60.06563352	-2.13305823	0.287642434	-7.415659089	1.21021E-13	1.90303E-12
SEL1L	196.4701989	1.14342989	0.154295864	7.410632137	1.25699E-13	1.97425E-12
PIGV	80.07624707	2.290164862	0.309250288	7.405538322	1.3062E-13	2.04913E-12

LOC121816974	32.40710561	2.425611265	0.327552477	7.405260035	1.30894E-13	2.05103E-12
PRKCB	22.01454148	6.642822564	0.897137148	7.404467173	1.31678E-13	2.0609E-12
RRBP1	502.4251775	1.425002855	0.192634935	7.39742694	1.38849E-13	2.17058E-12
FUOM	55.22109004	1.976307215	0.267186229	7.396740556	1.39568E-13	2.17928E-12
ARHGAP18	57.81347589	1.725712942	0.233351094	7.395349709	1.41037E-13	2.19964E-12
RPL6	128.5894206	-2.356840417	0.31874153	-7.394205634	1.42256E-13	2.21608E-12
SCAP	64.34392632	-1.93913551	0.262309177	-7.392556867	1.44032E-13	2.24113E-12
MARVELD1	69.24927201	1.805689971	0.244428578	7.38739301	1.49736E-13	2.32717E-12
INSR	53.0747916	1.737991427	0.235324836	7.38549936	1.51882E-13	2.35779E-12
GAS7	15.51588422	4.239684167	0.574103015	7.384883994	1.52586E-13	2.36597E-12
CAVIN1	135.1456916	1.428966068	0.193746121	7.375456406	1.63783E-13	2.53664E-12
UHRF1	72.6969154	-1.778093278	0.241140302	-7.373687692	1.65972E-13	2.56757E-12
CALR	225.2911278	1.12871948	0.15308708	7.373055122	1.66761E-13	2.5768E-12
CFL2	23.63801843	2.531520333	0.343413901	7.371630337	1.68554E-13	2.60149E-12
FERMT2	39.29170034	2.226872703	0.302312526	7.3661278	1.75655E-13	2.70797E-12
RPRD1B	55.68003158	1.583692493	0.215101514	7.362535313	1.8045E-13	2.77868E-12
AP1S1	162.9985791	1.429398381	0.19416597	7.361734808	1.81535E-13	2.78897E-12
LAGE3	27.41439581	-2.53990987	0.345011835	-7.361805055	1.8144E-13	2.78897E-12
ELMO1	13.26788039	7.072667224	0.961029656	7.359468235	1.84644E-13	2.83347E-12
CDYL2	17.22354994	3.481027552	0.473536412	7.351129642	1.96539E-13	3.01254E-12
LOC101106806	42.21610852	3.320800522	0.451755854	7.350874363	1.96914E-13	3.01484E-12
PCBP4	33.79588698	-2.061833691	0.280605458	-7.347803236	2.01491E-13	3.08138E-12
PPP1R37	48.34574345	-1.647588646	0.224255084	-7.346939983	2.02796E-13	3.09779E-12
LOC101119842	23.74355312	6.273410872	0.853962944	7.346233135	2.03871E-13	3.11066E-12
SLTM	496.5473746	1.515289297	0.206499822	7.337969033	2.16859E-13	3.30506E-12
EMILIN2	12.67006628	7.018262764	0.95672992	7.335678147	2.20602E-13	3.35827E-12
ATP9A	68.56727836	1.894230002	0.25825161	7.334823599	2.22014E-13	3.37592E-12
LLGL2	43.09791819	-2.10912569	0.287564925	-7.334433041	2.22662E-13	3.38193E-12
PPP1R16B	13.00146964	7.032306748	0.959289852	7.330742351	2.28881E-13	3.47245E-12
COL12A1	12.56825978	7.003441557	0.956069181	7.325245594	2.38462E-13	3.61369E-12
TMEM236	17.24762814	3.972480805	0.542492463	7.322647001	2.43127E-13	3.68022E-12
TEAD3	68.04239948	1.738813923	0.237518181	7.320761372	2.46568E-13	3.72809E-12
EMB	16.26567436	4.36179381	0.59582578	7.320585907	2.46891E-13	3.72875E-12
SMOC1	16.53778952	3.332316548	0.455208622	7.320416153	2.47203E-13	3.72925E-12
FUT1	42.19018293	-2.336929737	0.319317843	-7.318506579	2.50746E-13	3.77843E-12
PHF20L1	126.6428111	1.206676551	0.165023381	7.312155043	2.62892E-13	3.95699E-12
TRNASTOP-UCA	19.88268737	6.015693769	0.822815172	7.311111866	2.64941E-13	3.98335E-12
STX11	17.26626466	4.886264952	0.668571427	7.308515967	2.70109E-13	4.05649E-12
GPM6A	18.66835629	3.178011528	0.434930935	7.306933747	2.73307E-13	4.09992E-12
NCAM1	18.48736288	6.956383032	0.95243662	7.303775268	2.79804E-13	4.19266E-12
CPEB4	71.2563052	1.481482305	0.202920206	7.300812137	2.86036E-13	4.28125E-12
PALS1	60.94090468	1.415379834	0.193900344	7.299522031	2.88792E-13	4.31766E-12

UPF2	204.3344499	1.509059251	0.206869026	7.294756888	2.99199E-13	4.46826E-12
DOCK2	17.93109251	6.909495649	0.947239593	7.294348445	3.00108E-13	4.47683E-12
USH1C	57.5242189	1.933959071	0.26519932	7.292473719	3.04315E-13	4.53453E-12
TCEA3	53.4564334	1.878706324	0.257775364	7.288153111	3.14233E-13	4.6771E-12
ACTR2	119.1939466	1.487277221	0.204141875	7.285507794	3.20461E-13	4.7645E-12
HOXB4	14.0070802	7.139277926	0.980143411	7.283911561	3.24278E-13	4.81533E-12
LOC121816723	44.03532039	2.235546153	0.306921264	7.283777355	3.24601E-13	4.81533E-12
TRA2B	398.7270723	1.368840287	0.1879377	7.283478975	3.2532E-13	4.82065E-12
ZNF407	25.87883511	3.004470143	0.41257609	7.282220695	3.28369E-13	4.86045E-12
CDV3	41.9206825	-1.877332091	0.257826061	-7.281389944	3.30398E-13	4.88507E-12
JAK3	12.34221776	6.976656247	0.95857928	7.278121272	3.38501E-13	4.99934E-12
CLMP	12.43723027	6.982429606	0.959684107	7.275758297	3.4448E-13	5.08203E-12
LOC101116867	128.3505421	1.313609498	0.180554709	7.275409796	3.4537E-13	5.08955E-12
LOC101114082	20.48638788	-6.258419135	0.860289163	-7.274785508	3.46971E-13	5.10751E-12
RPL34	339.9445738	-1.584333453	0.217801082	-7.274222138	3.48422E-13	5.12323E-12
PSMA4	243.8446138	1.655886297	0.227670889	7.273157767	3.5118E-13	5.1581E-12
APH1A	146.8965472	-1.524407529	0.209605473	-7.272746786	3.5225E-13	5.16814E-12
CD3E	12.59210686	6.99156691	0.961950209	7.268117251	3.64532E-13	5.34248E-12
PPP4R2	140.9730956	1.415489204	0.194815689	7.265786517	3.70874E-13	5.42946E-12
CTBP1	32.62166178	-1.965541277	0.270557416	-7.264784347	3.73634E-13	5.46388E-12
LOC101120395	21.19622319	6.605700064	0.909525777	7.262795874	3.7917E-13	5.53878E-12
U2AF2	38.05581404	-2.077432888	0.286274351	-7.256790147	3.96384E-13	5.78392E-12
LOC101102973	12.91490722	7.025128325	0.968521629	7.253455285	4.06272E-13	5.92173E-12
S1PR1	12.93977806	7.024440958	0.968673047	7.251611862	4.11841E-13	5.99636E-12
BANK1	13.3605643	7.076615491	0.976316032	7.248283608	4.22086E-13	6.13885E-12
HERPUD1	91.40171717	1.678176973	0.231672221	7.243755713	4.36427E-13	6.34053E-12
SELENOK	23.9918094	2.79892939	0.386719535	7.237620892	4.56624E-13	6.62674E-12
RPS18	1094.708629	-1.261283253	0.17429331	-7.236555758	4.60223E-13	6.67173E-12
INHBA	20.73855815	6.55958129	0.906488638	7.236253181	4.6125E-13	6.67938E-12
FEN1	70.22494816	-1.478808118	0.204445085	-7.233277909	4.71473E-13	6.82003E-12
KCNA3	12.06531947	6.928992719	0.958398314	7.229763053	4.83837E-13	6.99132E-12
HCFC1R1	27.49145881	2.190461461	0.303137771	7.225960176	4.97574E-13	7.18204E-12
CD34	12.80598904	7.02882615	0.973292986	7.221696088	5.13431E-13	7.40294E-12
GRK3	15.17931977	4.45725636	0.617459258	7.218705203	5.24849E-13	7.55941E-12
UQCRH	175.878349	-0.949729663	0.131585345	-7.217594495	5.29152E-13	7.61319E-12
ATG4D	41.68508554	1.971304051	0.273141142	7.217162651	5.30835E-13	7.62918E-12
IRF4	11.866428	6.924281865	0.959528254	7.216339732	5.34055E-13	7.66722E-12
DHX16	79.36985336	1.881725257	0.260963088	7.210695088	5.5667E-13	7.98331E-12
SH3BP5L	66.20799613	-1.697181898	0.235403811	-7.209661932	5.6091E-13	8.0355E-12
RPS29	120.952518	-4.421164433	0.613252513	-7.209370262	5.62112E-13	8.0441E-12
LOC101116002	15.06931165	-3.152036613	0.437235143	-7.209019373	5.63562E-13	8.05383E-12
TMEM134	34.93066931	-1.91380514	0.265477579	-7.208914404	5.63997E-13	8.05383E-12

SLC43A1	25.17848408	2.56455894	0.355779495	7.208282023	5.66622E-13	8.08268E-12
SF3B5	81.62480761	-1.553236647	0.215493808	-7.207801753	5.68623E-13	8.10258E-12
AMN	20.7645229	4.861775556	0.674734155	7.20546829	5.78447E-13	8.23378E-12
FMC1	36.78907801	-1.952929354	0.271134797	-7.202798655	5.8989E-13	8.38774E-12
ZMIZ1	47.8735029	1.552467577	0.21563345	7.199567487	6.04039E-13	8.57979E-12
EMCN	12.67105413	6.997979383	0.97241219	7.196515487	6.17708E-13	8.76463E-12
ECHDC1	84.34381895	1.280153885	0.178004	7.191714147	6.39829E-13	9.06888E-12
TMEM11	66.98585332	-1.424292527	0.198070539	-7.190834829	6.43963E-13	9.11781E-12
TAF1D	64.70736496	-1.516337076	0.210885832	-7.190322193	6.46386E-13	9.14243E-12
PRR14	64.45913792	-1.628187182	0.226499277	-7.188487334	6.55131E-13	9.25632E-12
BTK	12.68172426	7.015722427	0.976407299	7.185241684	6.70884E-13	9.46889E-12
LOC101121420	16.98906555	-4.027556479	0.560620795	-7.184101119	6.76508E-13	9.53819E-12
AGRN	47.1880013	-1.619119437	0.225508744	-7.179852132	6.97869E-13	9.82899E-12
PRKAR2B	31.75679347	2.297389916	0.32010484	7.176992124	7.12618E-13	1.00262E-11
LOC101108901	203.4362834	11.01447861	1.535790648	7.171862014	7.39845E-13	1.03967E-11
NARF	73.790057	-1.463876578	0.204117393	-7.171738557	7.40513E-13	1.03967E-11
FOXA3	114.2097238	-1.296409441	0.180780512	-7.17117918	7.43545E-13	1.04284E-11
LOC114114253	17.47635817	3.186282809	0.444568237	7.167140028	7.65807E-13	1.07293E-11
PLEKHO1	40.26579071	1.914358369	0.267113533	7.166834083	7.6752E-13	1.07421E-11
ANKRD12	507.1316003	1.292803891	0.18039754	7.166416424	7.69864E-13	1.07636E-11
TXNDC17	48.82199544	-1.972127337	0.275333422	-7.162687793	7.91104E-13	1.1049E-11
EHBP1	76.53756104	1.245425911	0.17404428	7.155799142	8.31869E-13	1.16062E-11
MGST3	210.5961705	1.423422814	0.198985863	7.153386643	8.46627E-13	1.17998E-11
MAB21L2	12.90664547	7.022083867	0.981886543	7.15162451	8.57569E-13	1.19399E-11
SLC16A1	82.39263341	-1.485510054	0.207765674	-7.14993012	8.68221E-13	1.20756E-11
RPS20	950.695264	-0.869512971	0.121631381	-7.148755227	8.75683E-13	1.21667E-11
GALNT5	156.8820882	1.467037302	0.205327805	7.144854546	9.00912E-13	1.25043E-11
CDH5	12.30070741	6.948076943	0.972530109	7.144330934	9.04353E-13	1.2539E-11
LOC114111046	21.77146349	3.267255658	0.457465289	7.142084296	9.19262E-13	1.27325E-11
VPREB3	21.4824568	6.102286275	0.854692884	7.139741523	9.35066E-13	1.2938E-11
IRAG2	22.04457436	3.068598862	0.429942168	7.137236333	9.5226E-13	1.31623E-11
A1CF	69.38342104	1.841127423	0.258030252	7.135316153	9.65649E-13	1.33335E-11
FCGBP	110.5197321	1.873940482	0.2626899	7.13366018	9.77345E-13	1.34811E-11
AKT3	19.03365116	5.951718425	0.834773377	7.12974154	1.00558E-12	1.38562E-11
RNFT2	18.01956355	-2.714181095	0.380876198	-7.126150459	1.03215E-12	1.42077E-11
VIM	71.29002894	1.441697752	0.202338271	7.125185672	1.0394E-12	1.42929E-11
IL1RN	23.89801706	2.383458321	0.334534379	7.124703683	1.04305E-12	1.43282E-11
PEA15	32.26629573	2.413000137	0.338687775	7.124556344	1.04416E-12	1.43289E-11
LNPK	64.68917113	1.751708366	0.245915105	7.123223958	1.05431E-12	1.44533E-11
CXCL12	12.04993091	6.920477912	0.971701292	7.122021932	1.06355E-12	1.45501E-11
FERMT1	52.2859637	-1.508453499	0.211801276	-7.122022704	1.06355E-12	1.45501E-11
LOC101122151	22.07821092	6.662257744	0.935737991	7.119789736	1.08092E-12	1.47726E-11

LLGL1	27.03711639	-2.570997751	0.361166156	-7.118600974	1.09028E-12	1.48853E-11
GSAP	26.52063496	3.001082419	0.421657324	7.117349205	1.10022E-12	1.50057E-11
BRAP	80.19716511	1.432239628	0.201425421	7.110520701	1.15606E-12	1.57512E-11
ELAC2	24.07309196	-2.595277679	0.365007354	-7.110206546	1.15869E-12	1.5771E-11
USP33	77.55059797	1.553071105	0.218445887	7.109637663	1.16348E-12	1.582E-11
AMD1	160.4050151	-1.24513885	0.175238842	-7.105381656	1.1999E-12	1.62987E-11
PTH1R	17.07171919	6.839301214	0.962877231	7.102983634	1.22092E-12	1.65673E-11
LOC101108696	19.20032594	6.445828919	0.907511528	7.102751555	1.22297E-12	1.65783E-11
CALCRL	16.35734243	6.793370915	0.956901874	7.099339129	1.25355E-12	1.69756E-11
CES2	15.97927046	3.325606303	0.4685024	7.098376237	1.26231E-12	1.7077E-11
TMC5	29.54367982	2.425705028	0.341810262	7.096641902	1.27825E-12	1.72751E-11
GRN	182.0894964	-1.498848647	0.211219084	-7.096180046	1.28252E-12	1.73154E-11
RPS4X	418.5690091	-1.119335362	0.157794385	-7.093632397	1.30637E-12	1.76195E-11
CEP85L	16.06583338	6.760022677	0.953161874	7.092208426	1.31989E-12	1.77839E-11
RASA3	18.37853146	6.41036664	0.904158521	7.089870292	1.34238E-12	1.80687E-11
SIPA1	18.61681012	6.393487269	0.901845565	7.089337153	1.34756E-12	1.81202E-11
MPDZ	16.3273421	5.374457178	0.758168779	7.088734501	1.35344E-12	1.8181E-11
IL6ST	44.52577461	1.763581197	0.248849598	7.086936093	1.37114E-12	1.84002E-11
RPS27	255.02646	-1.199334899	0.169309455	-7.083685319	1.4037E-12	1.88184E-11
CSNK2A2	67.0835562	-1.390135899	0.196315113	-7.081145588	1.42968E-12	1.91473E-11
SH3BGR	26.77872365	8.084496813	1.141967268	7.079447054	1.44731E-12	1.93641E-11
LOC132658028	17.04620759	-4.086836624	0.57734812	-7.078635027	1.45581E-12	1.94584E-11
RACK1	157.6980097	-2.015039527	0.284799385	-7.075294519	1.49132E-12	1.9913E-11
LOC101105359	122.8575907	-1.328068056	0.187720381	-7.074714263	1.49757E-12	1.99766E-11
KDM5A	205.6542957	1.485049895	0.21001809	7.071057041	1.53758E-12	2.04898E-11
SERPING1	18.09879016	6.381276308	0.902775172	7.068511086	1.56605E-12	2.08484E-11
LOC101110848	12.15923235	6.926059026	0.98136006	7.057612498	1.69388E-12	2.25104E-11
NUP93	134.7089839	-1.317425613	0.186668152	-7.057581055	1.69426E-12	2.25104E-11
SFT2D1	41.20954081	1.915522767	0.271575141	7.053380352	1.74623E-12	2.31778E-11
LOC101116121	14.68855731	4.130251184	0.586171917	7.046143061	1.83945E-12	2.4391E-11
RPS12	117.2401481	-2.284819235	0.324386753	-7.043503507	1.87465E-12	2.48331E-11
HPGD	21.80015309	2.622563704	0.37263015	7.037980426	1.95046E-12	2.58117E-11
RPL4	138.3980914	-1.527337153	0.217034272	-7.037308616	1.95988E-12	2.59108E-11
COL4A2	11.07261477	6.806279932	0.967396485	7.035667422	1.98309E-12	2.61917E-11
BAAT	17.88832304	4.279495499	0.608309135	7.035066968	1.99165E-12	2.62788E-11
EML5	14.53169483	4.640627551	0.659807503	7.03330521	2.01697E-12	2.65867E-11
PPA1	222.5004064	-1.372631639	0.195229125	-7.030875333	2.05242E-12	2.70272E-11
CCT8	214.8953335	-1.141835993	0.162408157	-7.030656678	2.05564E-12	2.7043E-11
LOC101121244	30.06372955	1.992214239	0.283414711	7.029325432	2.07534E-12	2.72552E-11
TTC28	18.19516893	5.897026387	0.838921848	7.029291705	2.07585E-12	2.72552E-11
ABCC9	10.80162117	6.773949685	0.9641523	7.025808767	2.1283E-12	2.79164E-11
FIS1	39.37652874	2.166758373	0.308463606	7.024356631	2.15055E-12	2.81806E-11

EPSTI1	82.43523053	1.500278283	0.213774048	7.018056217	2.24976E-12	2.94518E-11
OTUD5	68.77624097	-1.532083625	0.218442875	-7.013658034	2.32166E-12	3.03634E-11
FAAH	14.67110635	4.368572361	0.623507655	7.006445437	2.44449E-12	3.19384E-11
LOC132657210	36.54852729	1.791390595	0.255785968	7.003474846	2.49691E-12	3.25915E-11
COL5A1	10.70035811	6.764092155	0.965884979	7.002999634	2.50539E-12	3.26703E-11
CD2	11.37365629	6.833521411	0.975933686	7.002034572	2.52272E-12	3.28642E-11
LOC101118514	10.96173306	6.788640255	0.970008317	6.998538195	2.58647E-12	3.36619E-11
PLCL1	10.93460759	6.790917151	0.97127437	6.991759855	2.71459E-12	3.5295E-11
ADSS2	202.2414333	-0.953650008	0.136449608	-6.989027086	2.76799E-12	3.59543E-11
SLAMF6	14.63744875	6.634248357	0.949273501	6.988763879	2.77319E-12	3.59868E-11
LOC114117376	13.15017633	-4.323348346	0.619296568	-6.981062981	2.92955E-12	3.7979E-11
EFEMP2	14.82571992	6.651815873	0.952952005	6.980221289	2.94715E-12	3.81702E-11
LOXL1	11.08826753	6.820588726	0.977814043	6.975343391	3.05125E-12	3.94419E-11
SEMA3D	10.96970287	6.789552431	0.973353544	6.975422728	3.04952E-12	3.94419E-11
QNG1	51.94816676	2.084392823	0.298861022	6.974455243	3.07058E-12	3.96535E-11
ASL	15.27448512	3.884204119	0.55707435	6.972505773	3.11345E-12	4.01683E-11
LOC101119159	19.0991563	6.429484302	0.923105978	6.965055429	3.28274E-12	4.23116E-11
SNRNP25	46.83848828	1.61396343	0.231763965	6.963823875	3.31159E-12	4.26423E-11
ZCCHC24	15.30541721	4.593322734	0.66025359	6.956906867	3.47825E-12	4.47452E-11
SNRPC	88.22705496	-1.368293608	0.196792941	-6.952960802	3.57698E-12	4.59711E-11
MFF	49.86471929	-1.567756288	0.225527771	-6.951499949	3.61423E-12	4.63925E-11
YKT6	28.11354643	-2.432604776	0.349944438	-6.951402876	3.61671E-12	4.63925E-11
IGF1R	68.93032956	1.424634985	0.205213424	6.942211475	3.86009E-12	4.94669E-11
LOC114113021	12.95063375	-6.732370197	0.969861605	-6.94157822	3.87743E-12	4.96416E-11
LOC101105123	30.51043174	2.048910219	0.295173191	6.94138317	3.88279E-12	4.96626E-11
NEU1	16.73378418	-3.032305867	0.437031658	-6.93841238	3.96531E-12	5.06695E-11
LOC101112936	14.34952794	6.609167269	0.952620775	6.937878578	3.98032E-12	5.08127E-11
RRM1	75.9593392	-1.246608459	0.179742399	-6.935528097	4.04706E-12	5.16155E-11
SEMA6A	15.30848324	4.909121221	0.707836234	6.935391246	4.05098E-12	5.16163E-11
IFT46	30.56489311	-2.350402712	0.339232689	-6.928585559	4.25069E-12	5.41093E-11
SIPA1L3	18.93537935	2.643778848	0.38182866	6.923992692	4.39089E-12	5.58407E-11
LOC101107119	145.3033846	1.620384027	0.234030895	6.923803917	4.39674E-12	5.58621E-11
SRRT	405.4476556	1.514326226	0.218731718	6.923212782	4.41514E-12	5.59893E-11
TSPOAP1	17.18581425	5.440239152	0.785781953	6.923344487	4.41103E-12	5.59893E-11
NDUFB11	49.98704779	1.664443326	0.240475425	6.921469529	4.46982E-12	5.66291E-11
CAPRIN1	133.7080933	-1.25850136	0.182019468	-6.91410305	4.70833E-12	5.95942E-11
USP32	43.62242204	1.702268092	0.246235826	6.913161755	4.73969E-12	5.99344E-11
HOXB9	17.48668275	2.882334851	0.41697912	6.9124201	4.76454E-12	6.01918E-11
S100G	11.64695274	6.886364405	0.996617345	6.909737667	4.85551E-12	6.12252E-11
SNX3	76.03072619	1.308784768	0.189409317	6.90982254	4.8526E-12	6.12252E-11
SPATS2L	209.3713819	1.031271109	0.149326079	6.906168811	4.97917E-12	6.27254E-11
MRPL15	24.67454386	-2.143128415	0.31068885	-6.897989467	5.27437E-12	6.63815E-11

RABGAP1L	200.1496904	1.298690932	0.188364517	6.894562499	5.40308E-12	6.79375E-11
DENR	45.97894916	-1.723930914	0.250085172	-6.893375169	5.44839E-12	6.84429E-11
OGN	11.71006737	6.892011711	0.999903858	6.892674388	5.47531E-12	6.87164E-11
NDUFA4	22.94252583	-2.190866865	0.318048355	-6.888470989	5.63952E-12	7.07109E-11
TTLL4	26.49446819	-2.599880949	0.377484118	-6.887391615	5.68246E-12	7.11826E-11
IGF2BP2	49.82144261	-1.451463278	0.210775048	-6.886314527	5.72563E-12	7.16562E-11
AKAP9	554.5689516	1.131568857	0.164402981	6.882897452	5.86472E-12	7.32597E-11
ARHGAP15	17.37902808	6.320412858	0.918267059	6.882978971	5.86137E-12	7.32597E-11
TFEC	10.47744002	6.740172656	0.979625488	6.880356563	5.97029E-12	7.45088E-11
MEP1B	25.02425459	2.46443635	0.358221735	6.87963936	6.00043E-12	7.4815E-11
MIER1	143.2644707	1.046360734	0.152185623	6.875555741	6.17487E-12	7.69182E-11
CYREN	25.67309964	-2.045367587	0.297516581	-6.874802015	6.2076E-12	7.71982E-11
WDR83	33.92673549	1.970423333	0.286616521	6.874772346	6.20889E-12	7.71982E-11
SNU13	22.79618724	-2.446900075	0.356088893	-6.871598983	6.34862E-12	7.88622E-11
GALNT4	81.94348544	1.485959889	0.216290215	6.870213196	6.4106E-12	7.95336E-11
SNX25	31.24986598	1.778709642	0.258904999	6.87012476	6.41458E-12	7.95336E-11
TRANK1	13.84590774	3.485867746	0.507420503	6.869781028	6.43005E-12	7.96516E-11
RPL19	210.9202284	-1.288017618	0.187510732	-6.86903413	6.4638E-12	7.99956E-11
CARHSP1	43.89651942	-1.860526442	0.270994739	-6.865544495	6.62381E-12	8.19E-11
RPL24	762.4847877	-1.166175017	0.169889361	-6.864320448	6.68085E-12	8.25289E-11
BNC2	17.71759813	6.31904613	0.921735871	6.855593156	7.10172E-12	8.76469E-11
SGMS2	151.1306433	1.25026531	0.18244927	6.85267369	7.24823E-12	8.93725E-11
GOLT1A	16.40290568	3.108408743	0.453753809	6.850430083	7.36283E-12	9.07019E-11
TOB1	40.78101306	1.733929243	0.253226116	6.84735551	7.52276E-12	9.25868E-11
NRG1	15.02445922	3.697174212	0.539973601	6.84695364	7.54392E-12	9.27617E-11
VPS37B	51.87831839	-1.564122758	0.228467604	-6.846146802	7.58656E-12	9.32004E-11
ICA1	67.78007061	-1.574179878	0.230003675	-6.844150976	7.69308E-12	9.44221E-11
ERF	49.28430143	-2.068739707	0.302332981	-6.842586929	7.77757E-12	9.53715E-11
NOL7	106.3744515	-1.198479284	0.175182555	-6.841316378	7.84687E-12	9.61332E-11
MBTPS1	51.66204694	1.471277574	0.215136139	6.838821129	7.98475E-12	9.77328E-11
SDC2	15.78515693	6.179550755	0.904762377	6.830026217	8.48991E-12	1.03821E-10
PLCB2	13.09683188	7.049658062	1.032242358	6.829460165	8.52348E-12	1.04136E-10
UTP18	90.70397927	-1.225073074	0.179407933	-6.828421989	8.58537E-12	1.04796E-10
HLX	10.37516594	6.708577039	0.982706903	6.826630626	8.69321E-12	1.05986E-10
LOC101120084	17.89871742	2.692689796	0.394444322	6.82653963	8.69873E-12	1.05986E-10
FAT4	10.26286921	6.690038637	0.981001543	6.819600523	9.1294E-12	1.11133E-10
CCL20	34.69263526	-2.248363218	0.329773178	-6.817908079	9.23757E-12	1.12347E-10
NAT1	25.44072662	2.569167317	0.376841221	6.817638761	9.2549E-12	1.12455E-10
LOC132657599	15.5903644	5.679544247	0.833088365	6.817457171	9.2666E-12	1.12495E-10
RBM17	87.18454306	-1.696676707	0.248978093	-6.814562232	9.45512E-12	1.1468E-10
DAXX	42.31330756	-1.704750509	0.250307836	-6.810615813	9.71818E-12	1.17764E-10
NUP188	26.43937659	-2.660007195	0.390601346	-6.810030805	9.75778E-12	1.18136E-10

DSEL	15.40454927	5.29208585	0.777281174	6.808457517	9.86507E-12	1.19327E-10
TBC1D8B	31.01038799	1.984350614	0.291617818	6.804627462	1.01311E-11	1.22434E-10
DLST	35.16131514	-2.05124886	0.301462006	-6.804336254	1.01516E-11	1.22571E-10
LOC101112694	190.1933035	-0.959261023	0.140993236	-6.803596064	1.02039E-11	1.23092E-10
KIAA1755	9.727543511	6.618290199	0.973062861	6.801503237	1.03533E-11	1.24683E-10
NTMT1	25.91611658	-2.238383808	0.329102113	-6.8014872	1.03545E-11	1.24683E-10
SH3RF1	62.5106164	-1.60629584	0.236280278	-6.798264568	1.05887E-11	1.27388E-10
COL6A2	9.732461865	6.631006873	0.975555815	6.797157858	1.06703E-11	1.28255E-10
IPCEF1	9.744956367	6.62043697	0.974472044	6.793870598	1.09164E-11	1.31095E-10
USP22	136.6268352	-1.697165262	0.249844937	-6.792874341	1.09921E-11	1.31886E-10
FLT1	9.608887236	6.596394175	0.971391278	6.790666464	1.11617E-11	1.338E-10
GADD45A	98.0524299	1.225077725	0.180443512	6.789258919	1.12711E-11	1.34888E-10
SHROOM1	14.71940342	-3.107033315	0.45764084	-6.789239604	1.12726E-11	1.34888E-10
PFN1	107.4414383	1.801012861	0.265482369	6.783926428	1.16953E-11	1.3982E-10
TRNAU1AP	29.24009345	-1.918337642	0.282834167	-6.782552692	1.18071E-11	1.41031E-10
MMP14	13.96086827	6.56822381	0.968694714	6.780488953	1.1977E-11	1.42932E-10
GJC1	9.448524625	6.575638775	0.970127438	6.778118543	1.21751E-11	1.45167E-10
ING1	77.85054497	-1.481255472	0.218645417	-6.774692527	1.24671E-11	1.48516E-10
HOOK1	67.22119419	-1.447570942	0.213708029	-6.773591766	1.25624E-11	1.49518E-10
ELOVL7	13.33378223	-3.50986124	0.518405048	-6.770499731	1.28338E-11	1.52613E-10
NCKAP1L	15.69838736	5.243937421	0.774620333	6.769687285	1.29061E-11	1.53336E-10
PRSS23	95.80192583	-1.946736763	0.287744127	-6.765513465	1.32837E-11	1.57682E-10
SLC25A37	253.9754238	1.118835613	0.165579581	6.757086856	1.40794E-11	1.66979E-10
LOC101122400	21.73597411	2.822865696	0.417931209	6.754378803	1.43449E-11	1.69976E-10
LOC114114909	149.9072148	-1.508201731	0.223369442	-6.752050398	1.4577E-11	1.72574E-10
HDDC2	37.41691118	-1.834221119	0.271752357	-6.74960518	1.48248E-11	1.75352E-10
TAX1BP1	221.1406078	1.021375325	0.151329518	6.749346317	1.48513E-11	1.7551E-10
MSMO1	181.5409654	0.922401062	0.136698755	6.747691754	1.50216E-11	1.77366E-10
CRLF3	21.07254219	2.193621996	0.325178226	6.745906767	1.52074E-11	1.79402E-10
CMTM4	34.33016197	-1.731016038	0.256657792	-6.744451525	1.53606E-11	1.81049E-10
KLF9	14.3428786	5.559101662	0.82430506	6.743985853	1.541E-11	1.81471E-10
EPS8L3	188.4226659	1.344630566	0.199435157	6.742194249	1.56012E-11	1.83562E-10
ATP5MC2	42.98732587	-2.085335017	0.309364383	-6.740708146	1.57617E-11	1.85212E-10
CCNB1	15.03765931	-2.913047986	0.432162007	-6.74063878	1.57692E-11	1.85212E-10
FKBP14	23.37330556	2.573445736	0.381914374	6.738279332	1.60273E-11	1.88078E-10
ANKRD17	304.2567847	1.122792941	0.166730945	6.734160504	1.64879E-11	1.93314E-10
CNN2	12.72991556	6.437275323	0.956125204	6.732669842	1.66578E-11	1.95134E-10
FTL	38.8502864	-2.720243755	0.404063942	-6.732211095	1.67104E-11	1.95579E-10
PHLDA1	85.71971335	-1.403224391	0.20851263	-6.729685334	1.7003E-11	1.9883E-10
MME	37.08802487	1.69869712	0.25260263	6.724780011	1.75858E-11	2.05465E-10
BBC3	26.80911494	-2.211185172	0.329099275	-6.718900154	1.83101E-11	2.13646E-10
UBE2H	153.0271803	0.975764181	0.14522812	6.718837811	1.8318E-11	2.13646E-10

COL5A2	15.2806197	6.106722753	0.908994457	6.718107803	1.84099E-11	2.14531E-10
LOC114111253	19.9983314	3.997220142	0.595135347	6.716489221	1.86155E-11	2.16738E-10
SHISA3	13.44661362	6.513476249	0.969933019	6.715387682	1.87567E-11	2.18191E-10
ENSA	261.6153698	-1.098873875	0.163652274	-6.714687497	1.8847E-11	2.19051E-10
SQSTM1	51.11819878	-1.788759545	0.266409117	-6.714333069	1.88928E-11	2.19393E-10
PRKACB	52.67535337	1.665101653	0.248033621	6.713209469	1.90389E-11	2.20898E-10
SCG2	23.49431249	2.590957615	0.38619366	6.708959474	1.96017E-11	2.2723E-10
MSX2	12.72589234	-3.565263567	0.53150134	-6.707910779	1.97431E-11	2.2867E-10
LOC114115573	13.72518785	-3.579602013	0.533676848	-6.707433577	1.98077E-11	2.29221E-10
TAF7	64.94002106	-1.507412036	0.22477943	-6.706183207	1.99781E-11	2.30992E-10
PTPRE	28.98412561	1.947524274	0.29043392	6.705567564	2.00625E-11	2.31768E-10
JAK1	345.452582	1.046506333	0.156069076	6.705404834	2.00849E-11	2.31826E-10
HMGN1	140.091968	-1.225764571	0.182834002	-6.704248423	2.02446E-11	2.33467E-10
RNF216	40.99684418	1.78320091	0.266234099	6.697868215	2.11482E-11	2.43678E-10
LOC101118441	9.742325448	6.621890761	0.988748021	6.697248055	2.12381E-11	2.44503E-10
TRMT13	108.0257831	1.260579994	0.188286644	6.695004844	2.15664E-11	2.4807E-10
NDFIP1	36.54896217	1.707320837	0.255019077	6.694874973	2.15856E-11	2.48077E-10
RNF220	55.17412254	-1.42992902	0.21359992	-6.694426747	2.16519E-11	2.48624E-10
TM4SF4	141.9934243	1.464282826	0.218757835	6.693624601	2.17709E-11	2.49777E-10
ERICH4	17.62641381	3.114710918	0.465549259	6.690400337	2.22561E-11	2.55124E-10
ATP8B4	15.12199433	3.163545598	0.472917603	6.689422377	2.24053E-11	2.56615E-10
CCBE1	12.91592233	6.445318152	0.963753488	6.687724851	2.26667E-11	2.59386E-10
SMLR1	16.05426577	4.492382087	0.671865813	6.686427555	2.28684E-11	2.61471E-10
POM121C	60.16875108	-1.596920792	0.238837426	-6.68622511	2.29001E-11	2.61609E-10
AMOT	66.94547807	-1.423554222	0.212917369	-6.685946902	2.29436E-11	2.61883E-10
CYTIP	22.8509787	2.163364684	0.323691235	6.683420645	2.33428E-11	2.66212E-10
SRGN	9.794107555	6.618031678	0.990242703	6.683242059	2.33713E-11	2.66309E-10
POLA2	18.64383271	-2.495947361	0.37363082	-6.680250199	2.38535E-11	2.71572E-10
ADCY6	40.35057109	-1.922813239	0.28795877	-6.67739078	2.43234E-11	2.76686E-10
NSUN2	136.9170134	-1.539671118	0.230681049	-6.674458619	2.48146E-11	2.82035E-10
NDUFA5	50.92280996	1.404546591	0.210555541	6.67067028	2.54638E-11	2.89166E-10
PSME2	146.728757	1.367881141	0.205098709	6.669379574	2.56887E-11	2.91473E-10
HPSE2	9.751933295	6.627627557	0.993791337	6.669033335	2.57494E-11	2.91914E-10
FCGR3A	8.998747653	6.518999685	0.977894259	6.666364616	2.62217E-11	2.97017E-10
EEF1A1	359.239246	-0.855089877	0.128275599	-6.666036925	2.62803E-11	2.97428E-10
CCL5	8.898971636	6.498904235	0.975094743	6.664895163	2.64854E-11	2.99496E-10
CCL21	9.254191749	6.567495257	0.985597769	6.663464006	2.67448E-11	3.02173E-10
FABP1	419.2484164	2.625368841	0.394456833	6.655655627	2.8204E-11	3.18391E-10
SAMSN1	8.967031991	6.510780017	0.978672399	6.652665407	2.87832E-11	3.24655E-10
CTSD	193.9652205	-1.23319951	0.185383441	-6.652155663	2.88831E-11	3.25507E-10
DPYSL2	64.85188626	1.612454149	0.242558519	6.647691267	2.97726E-11	3.35249E-10
NDUFB9	33.91582698	-1.83593225	0.276249306	-6.645925283	3.01318E-11	3.39008E-10

RGS18	8.617978499	6.459991626	0.972186826	6.644804736	3.03619E-11	3.4131E-10
LOC101110277	138.9909742	1.473994734	0.221941173	6.641375806	3.10769E-11	3.49053E-10
KIT	19.36302411	2.358805934	0.35519788	6.640822101	3.11938E-11	3.50073E-10
CDK2	61.75498485	-1.751966155	0.263834538	-6.640397298	3.12839E-11	3.50789E-10
EDNRB	9.237423217	6.539924637	0.984992594	6.639567318	3.14605E-11	3.52474E-10
PDS5B	86.74770061	1.394190422	0.210088373	6.636209325	3.21853E-11	3.60292E-10
AASDHPPT	142.0340027	-0.942405009	0.142042847	-6.634653074	3.25267E-11	3.63201E-10
PRELID1	268.4922133	-1.612394611	0.243025859	-6.634662729	3.25245E-11	3.63201E-10
SPDEF	15.34374706	3.731282597	0.562385498	6.634741848	3.25071E-11	3.63201E-10
LOC101108654	7.561663553	-6.544394692	0.986840624	-6.631663242	3.31925E-11	3.70018E-10
LOC106991031	12.68921651	-3.450969851	0.520368742	-6.631777758	3.31668E-11	3.70018E-10
RPL30	195.7871752	-2.831658931	0.427071295	-6.630412688	3.3475E-11	3.72855E-10
GPT2	53.9807659	-2.396160473	0.361450876	-6.629283902	3.37319E-11	3.75405E-10
MICU2	106.4668319	1.100039939	0.166038311	6.625217595	3.46737E-11	3.85565E-10
TRAIP	15.74466291	-2.854533597	0.43093933	-6.623980219	3.49654E-11	3.88485E-10
RUVBL1	50.15935647	-1.507137291	0.227566847	-6.622833311	3.52378E-11	3.91187E-10
MIDEAS	59.99336676	1.762488921	0.266146582	6.622248943	3.53775E-11	3.92086E-10
PTPN7	8.533696135	6.433838511	0.971533343	6.622354816	3.53521E-11	3.92086E-10
RCOR2	12.59987262	-3.564931502	0.538341563	-6.622062544	3.54221E-11	3.92255E-10
MMD	26.71422988	2.090658249	0.315813603	6.619911967	3.59413E-11	3.97675E-10
MLKL	17.9693963	2.771830921	0.418805898	6.61841425	3.63072E-11	4.01392E-10
ST8SIA4	8.612155075	6.442166118	0.973753552	6.615807567	3.69529E-11	4.08192E-10
DCTPP1	13.88065898	-3.106377765	0.469583924	-6.615170597	3.71124E-11	4.09615E-10
MAP2K2	77.57193892	1.437313838	0.217381348	6.611946475	3.79299E-11	4.18293E-10
NABP1	24.21795265	2.304699348	0.348743526	6.608579583	3.88025E-11	4.27563E-10
RIMBP2	16.37660594	-2.645992849	0.400464109	-6.607315837	3.91351E-11	4.30872E-10
MAN2A2	20.34950152	-2.212339618	0.334865702	-6.606647399	3.93121E-11	4.3211E-10
TCN2	12.94715439	3.663514054	0.554518919	6.606652952	3.93106E-11	4.3211E-10
PON3	14.93797262	4.867441256	0.736918079	6.605132101	3.97163E-11	4.36194E-10
RBMS3	12.1758705	6.362511574	0.963344753	6.604605	3.98579E-11	4.37389E-10
NBAS	53.25081284	1.528428284	0.231434502	6.604150501	3.99803E-11	4.38303E-10
SMARCA1	61.99678668	1.456295266	0.220515387	6.604052843	4.00067E-11	4.38303E-10
LIMCH1	15.38861724	6.118970784	0.927720326	6.595706281	4.23236E-11	4.63307E-10
OCIAD1	189.0562072	-1.330591907	0.201747569	-6.595330559	4.24309E-11	4.64101E-10
PLXNA4	12.88949355	6.427274019	0.975123969	6.591237856	4.36174E-11	4.76689E-10
PDGFA	78.06220949	-1.333417811	0.20240814	-6.587767731	4.46488E-11	4.87562E-10
SLC37A2	15.94546111	6.172133393	0.937136559	6.586162214	4.51341E-11	4.92459E-10
TOMM7	46.36149607	-1.645849067	0.250005008	-6.583264392	4.6023E-11	5.01748E-10
RBM14	26.28689893	2.366474197	0.359628609	6.58032798	4.69412E-11	5.11341E-10
TMEM139	15.5744909	3.089254015	0.469722373	6.576765746	4.80791E-11	5.23311E-10
NRARP	16.09407911	-2.911744136	0.442804374	-6.575689642	4.84282E-11	5.26681E-10
HAUS5	46.01479204	-1.580881061	0.240424661	-6.575369815	4.85324E-11	5.27385E-10

CNTLN	12.95289483	6.450289916	0.981014295	6.575123268	4.86129E-11	5.27402E-10
DNAAF5	62.39391498	-1.462490387	0.222423845	-6.575241011	4.85744E-11	5.27402E-10
ZNF446	15.75960694	-3.091165611	0.470364126	-6.571856656	4.96917E-11	5.38669E-10
FBL	35.60354371	-2.288083521	0.348235656	-6.570503283	5.01455E-11	5.43148E-10
ASRGL1	12.39121858	6.372223614	0.969962282	6.56955815	5.04648E-11	5.46164E-10
PCOLCE2	10.01600239	6.684909317	1.017851933	6.567663823	5.11108E-11	5.52708E-10
MCTP1	13.64023278	4.513491291	0.687264832	6.567324682	5.12273E-11	5.5352E-10
CHCHD1	62.43392815	1.817569924	0.276854333	6.565076682	5.20061E-11	5.61481E-10
C4BPB	13.33278175	4.290559707	0.654036219	6.560125546	5.37625E-11	5.79975E-10
CREB3L3	17.57449793	2.814395956	0.429084988	6.559064132	5.41465E-11	5.83647E-10
TAOK3	55.52674243	-1.645557824	0.250893675	-6.558785617	5.42477E-11	5.84267E-10
MAN1A1	25.05015389	2.100380977	0.320248786	6.558591533	5.43184E-11	5.84557E-10
IRF2	106.0292888	1.246238676	0.190028924	6.558152581	5.44785E-11	5.85808E-10
FOXF1	11.82792869	6.303525577	0.9614328	6.556387067	5.5127E-11	5.92305E-10
CDKN2D	27.41527321	1.920550448	0.292936945	6.556190604	5.51997E-11	5.92609E-10
GUCD1	68.98517539	-1.2330723	0.188139411	-6.554035074	5.60028E-11	6.00749E-10
ARHGAP25	9.723531441	6.616235991	1.009741779	6.552403917	5.66181E-11	6.06863E-10
IL16	8.16649993	6.37730931	0.973338134	6.551997795	5.67724E-11	6.08029E-10
CD276	40.53609657	-1.585850645	0.242088828	-6.55069735	5.7269E-11	6.12856E-10
WNT2B	11.48565899	6.276084333	0.958207326	6.549818773	5.76069E-11	6.1598E-10
IL4R	30.81546996	2.072871164	0.316490469	6.549553192	5.77095E-11	6.16583E-10
CEP152	41.89699035	-1.583298653	0.241830289	-6.547147837	5.86463E-11	6.26091E-10
CEP350	157.8741806	1.04052207	0.15907671	6.541008232	6.11055E-11	6.51824E-10
LOC114111326	14.53244465	3.298648819	0.504342483	6.540493676	6.13161E-11	6.53549E-10
LOC105614210	13.27391714	-3.046599305	0.465842791	-6.539973066	6.15299E-11	6.55306E-10
BLVRA	46.97287597	-1.656828088	0.253393996	-6.538545174	6.21201E-11	6.61065E-10
ZNF518B	41.39181299	2.565453788	0.392403363	6.537797665	6.24313E-11	6.63848E-10
LOC105611302	17.4148821	-3.754487749	0.574576235	-6.534359617	6.38823E-11	6.78737E-10
PSMD1	244.7765244	1.238141551	0.189624853	6.529426562	6.6022E-11	7.00913E-10
LYPD1	8.913203539	6.49181726	0.994613657	6.526973782	6.71118E-11	7.11918E-10
MYADM	170.7579832	1.623859479	0.248840561	6.525702537	6.76835E-11	7.17413E-10
TNFAIP6	8.555829352	6.433219397	0.986030362	6.524362381	6.82914E-11	7.23283E-10
GUCY1A1	8.594564649	6.437825611	0.987493301	6.519361299	7.06074E-11	7.4722E-10
LOC114117997	13.11535306	5.38091339	0.82543525	6.518880057	7.08343E-11	7.49028E-10
WASF3	14.06519144	5.522261294	0.847494642	6.515983727	7.22148E-11	7.63022E-10
ARID4A	316.9165716	1.237666365	0.189977111	6.514818318	7.27777E-11	7.68362E-10
PRDM1	13.60826714	5.94799774	0.913077523	6.514230816	7.30631E-11	7.70766E-10
LOC105608416	9.56016194	6.576813059	1.009716668	6.513523316	7.34082E-11	7.73797E-10
HIBCH	49.655184	-2.243092671	0.344510107	-6.510963316	7.46704E-11	7.86481E-10
RPL10	34.57345871	-2.518715218	0.387007562	-6.508180891	7.60663E-11	8.00553E-10
RFLNA	14.91270242	-3.742027298	0.575061877	-6.507173313	7.6578E-11	8.05305E-10
PLEKHG1	84.02143041	1.309886588	0.201312247	6.506740703	7.67988E-11	8.06991E-10

LOC101109370	21.2443067	2.28781202	0.351667766	6.505606267	7.73807E-11	8.12467E-10
B4GALT2	66.8620573	-1.305826394	0.200842606	-6.501739951	7.93963E-11	8.32975E-10
C16H5orf34	65.24039278	2.143236391	0.329665136	6.501252817	7.96538E-11	8.35022E-10
CAST	161.9423421	1.501517415	0.230966066	6.501030386	7.97717E-11	8.35602E-10
CHORDC1	123.2307635	1.135851745	0.174819353	6.497288333	8.17806E-11	8.55975E-10
DSE	13.18567898	5.461606117	0.840907445	6.494895663	8.3091E-11	8.69009E-10
SPOCK2	13.92405213	2.995795299	0.461463048	6.491950567	8.47321E-11	8.8548E-10
UBL3	59.88978198	1.356964789	0.209029514	6.491737753	8.48519E-11	8.86039E-10
XPR1	36.11932396	1.861533589	0.286787114	6.490994535	8.52716E-11	8.89727E-10
ADAMTS5	13.3259435	5.92680987	0.913294782	6.489481802	8.61321E-11	8.98005E-10
EHBP1L1	12.39075595	3.896267753	0.600491473	6.488464746	8.67154E-11	9.03382E-10
MRPS21	48.81954326	-1.550617629	0.239163044	-6.48351687	8.96089E-11	9.32797E-10
MSRB2	33.69053911	1.701491722	0.262453218	6.483028618	8.98994E-11	9.35094E-10
CRTAP	84.61412612	-1.155119104	0.178253922	-6.480189005	9.16078E-11	9.52123E-10
AKR1B1	13.04016212	5.448586366	0.840863818	6.479748862	9.18754E-11	9.54162E-10
LRRK2	13.113773	3.177745297	0.490545506	6.47798269	9.2957E-11	9.63897E-10
PPP6R2	24.33349048	-2.166217412	0.334392683	-6.478064627	9.29066E-11	9.63897E-10
LOC101110664	74.20472478	-1.688769905	0.260742828	-6.476764548	9.37102E-11	9.70954E-10
MRPL36	105.4610921	-1.663514128	0.256926087	-6.474679732	9.50133E-11	9.83693E-10
CREB3	63.11686641	1.562580192	0.241438665	6.471955064	9.67429E-11	1.00082E-09
DNPH1	28.84764688	-2.402557431	0.371235667	-6.471785035	9.68518E-11	1.00118E-09
JAM2	7.892846906	6.333586453	0.978978375	6.469587697	9.82707E-11	1.01506E-09
FLI1	8.570049302	6.43356182	0.994708202	6.46778805	9.94478E-11	1.02642E-09
AKAP13	65.09111938	1.393336127	0.215446657	6.467197725	9.9837E-11	1.02965E-09
MLX	51.39294163	-1.444121643	0.223390953	-6.464548457	1.01602E-10	1.04704E-09
LOC101101976	7.984296039	6.333271791	0.979728932	6.464310261	1.01762E-10	1.04788E-09
RAB27A	24.74774383	1.9914251	0.308085661	6.46386817	1.0206E-10	1.05014E-09
SATB1	20.869777	2.26618139	0.350627488	6.463216572	1.025E-10	1.05386E-09
GNA11	176.9359104	1.171560635	0.18129277	6.462257908	1.03152E-10	1.05975E-09
AKAP8L	139.4261279	1.889346513	0.292698178	6.454930893	1.08268E-10	1.11145E-09
TMPRSS2	130.5242958	1.155072051	0.178980904	6.453604973	1.0922E-10	1.12037E-09
LOC105605807	122.5938656	1.218339424	0.188802168	6.452994896	1.09661E-10	1.12403E-09
RPL23	1195.532783	-0.750350199	0.116293161	-6.452229817	1.10216E-10	1.12885E-09
HSP90AA1	3011.318748	1.11920303	0.173623279	6.446157675	1.14721E-10	1.17409E-09
SUSD3	11.63115193	6.291504913	0.976110871	6.445481861	1.15234E-10	1.17843E-09
AFF3	12.51093195	3.40746234	0.528919747	6.442305018	1.17672E-10	1.20185E-09
KEF53_p12	4628.714731	1.680963944	0.260927473	6.442265065	1.17703E-10	1.20185E-09
ACTA1	8.911082906	6.477667921	1.006252405	6.437418575	1.21522E-10	1.2399E-09
NAXD	18.16776882	-2.636879739	0.409649654	-6.436914354	1.21927E-10	1.24308E-09
GDA	12.71324147	5.344938568	0.830465553	6.436074981	1.22602E-10	1.24901E-09
TOPORS	58.3324046	1.213248165	0.188511586	6.435934195	1.22716E-10	1.24922E-09
MRCL3	34.49046701	-1.775637993	0.276020813	-6.432985892	1.25121E-10	1.27273E-09

LOC114113408	14.85867775	3.264865569	0.507678085	6.430975983	1.26787E-10	1.2887E-09
MGAT4A	149.4134725	1.206920218	0.187732223	6.428945443	1.28492E-10	1.30504E-09
SDF4	66.72418876	-1.479874663	0.230194721	-6.428794964	1.28619E-10	1.30534E-09
MIA3	185.8931173	1.404994673	0.21861251	6.426872236	1.30256E-10	1.32094E-09
TRPV2	34.54669452	-1.651719942	0.257124707	-6.42380874	1.32906E-10	1.3468E-09
CAPN5	37.91724961	2.031271771	0.316324439	6.421482252	1.34954E-10	1.36634E-09
MAML3	88.67915191	-1.184328361	0.184435004	-6.421386065	1.35039E-10	1.36634E-09
DTNA	12.93719173	3.903388514	0.608135866	6.418612575	1.37522E-10	1.39041E-09
TMEM243	29.95662743	2.040975679	0.318087874	6.416389441	1.39544E-10	1.40979E-09
SLC46A1	26.01693612	1.951343908	0.304133391	6.416079153	1.39829E-10	1.41159E-09
CD244	11.0859595	6.216547375	0.969023495	6.415270017	1.40573E-10	1.41804E-09
SNAPC1	50.02798554	-1.314236126	0.204968265	-6.411900511	1.43717E-10	1.44865E-09
SLC25A36	50.70630558	-1.623025882	0.253150022	-6.411320329	1.44265E-10	1.45308E-09
TTC3	171.8736993	1.346906429	0.210333398	6.403673604	1.51682E-10	1.52664E-09
SAE1	51.27366849	-1.888686094	0.295093001	-6.400307999	1.55064E-10	1.55951E-09
KMT2A	167.7155172	1.313421661	0.205284573	6.398053397	1.5737E-10	1.58151E-09
YIPF4	72.23107715	1.268108597	0.198251835	6.39645325	1.59027E-10	1.59697E-09
LOC101116816	94.92319005	1.42408469	0.222645016	6.396211846	1.59279E-10	1.59829E-09
NIPBL	368.5337147	0.919371862	0.143872879	6.390167954	1.65704E-10	1.66151E-09
TALDO1	270.534291	0.80817937	0.126529681	6.387271048	1.68872E-10	1.69201E-09
LOC114116075	7.143259545	-6.367229009	0.997134006	-6.385529897	1.70805E-10	1.71003E-09
RIPK1	50.6060563	1.631575912	0.255515779	6.385421358	1.70926E-10	1.71003E-09
SLITRK6	10.56974878	6.149306514	0.963190421	6.384310284	1.72172E-10	1.7212E-09
LOC101122734	20.88398908	-2.225296113	0.348641899	-6.382755825	1.73929E-10	1.73747E-09
PLAC8	29.73412238	2.213382122	0.346929219	6.379924205	1.77176E-10	1.76858E-09
COL15A1	8.102874906	6.356050541	0.996928433	6.375633725	1.82208E-10	1.81746E-09
DERL2	116.9579797	1.160235151	0.181993446	6.375148	1.82786E-10	1.82187E-09
LOC101113775	25.63547672	2.327001882	0.365079433	6.373960494	1.84208E-10	1.83467E-09
HAS2	7.920387552	6.325816697	0.992500457	6.373615903	1.84623E-10	1.83743E-09
NEURL4	19.01421849	-2.432681928	0.381754808	-6.37236749	1.86132E-10	1.85107E-09
HSD17B2	23.87202748	2.043195912	0.320654073	6.371963067	1.86624E-10	1.85458E-09
COL14A1	13.69099869	5.478366692	0.860117129	6.369326349	1.8986E-10	1.88534E-09
CIRBP	210.4078999	-1.107077549	0.173817832	-6.369182807	1.90038E-10	1.88571E-09
PIK3CG	12.55861181	5.838188371	0.916810687	6.367932286	1.91593E-10	1.89973E-09
SEC11C	56.92257911	1.714807488	0.269337621	6.366758129	1.93065E-10	1.91291E-09
PAOX	14.65668847	2.764135624	0.434194029	6.366129975	1.93857E-10	1.91933E-09
ARL3	15.64045747	-2.694954258	0.423585503	-6.362243844	1.98827E-10	1.96378E-09
FAM98A	46.35796713	1.448107187	0.227612468	6.362161098	1.98935E-10	1.96378E-09
HOXA5	10.91673239	6.194648298	0.973652806	6.362276431	1.98785E-10	1.96378E-09
LOC106991783	60.86135138	-1.458560948	0.22925014	-6.362312124	1.98739E-10	1.96378E-09
ZNF711	30.64496721	-1.819228857	0.285984095	-6.361293819	2.00061E-10	1.97345E-09
ACIN1	323.5881801	1.358949767	0.213664143	6.36021443	2.01472E-10	1.9859E-09

LARP1B	77.65858564	1.114296665	0.175208108	6.359846463	2.01956E-10	1.98919E-09
EMILIN1	7.337562933	6.231333827	0.980175346	6.357366414	2.05242E-10	2.02008E-09
MEDAG	7.789929645	6.318748713	0.99425071	6.355287099	2.08038E-10	2.04609E-09
LOC101119038	237.409469	-0.947354637	0.149115253	-6.353170568	2.10922E-10	2.07293E-09
EVA1B	11.88571341	5.287836991	0.83242939	6.352294924	2.12126E-10	2.08323E-09
BLTP1	125.2691844	1.135853056	0.178819723	6.35194506	2.12609E-10	2.08644E-09
PLSCR4	24.86511539	2.064960184	0.325149152	6.35080908	2.14185E-10	2.10037E-09
KDM4B	31.13618126	-1.663158008	0.26192729	-6.349693483	2.15744E-10	2.1141E-09
CMTM8	22.1154262	-2.861638	0.450699385	-6.349327496	2.16258E-10	2.11759E-09
SLC28A2	13.14449538	5.428228459	0.8550881	6.348151098	2.17918E-10	2.13228E-09
RPL10A	50.93762884	-2.326741526	0.366622623	-6.34642104	2.20382E-10	2.15481E-09
NCKIPSD	24.44874846	-2.209212483	0.348309214	-6.342675977	2.25808E-10	2.20625E-09
SSBP1	47.43421291	-1.892704244	0.298461283	-6.341540267	2.27479E-10	2.22096E-09
TENM4	10.32901728	6.116259729	0.965214148	6.336686779	2.34759E-10	2.29035E-09
SERPINA1	11.28557071	-3.302309463	0.521161472	-6.336442038	2.35132E-10	2.29232E-09
ZNF608	49.98834644	1.576822405	0.248903332	6.335079525	2.37219E-10	2.31098E-09
ARPC1B	255.9940401	-1.278736473	0.201929951	-6.332574583	2.41104E-10	2.34712E-09
MCM7	85.18641701	-1.227101593	0.19378071	-6.332423858	2.41339E-10	2.3477E-09
LOC114110053	23.34121502	-2.133095482	0.337071796	-6.328311962	2.47858E-10	2.40936E-09
TMCO4	92.38161342	1.915271225	0.302737657	6.326504757	2.50777E-10	2.43596E-09
LOC101108297	7.710208956	6.303347339	0.996430723	6.325926321	2.51718E-10	2.44333E-09
SPON1	7.333856277	6.229522775	0.984903554	6.325007915	2.5322E-10	2.45612E-09
PANK3	67.57714914	1.491674068	0.235858705	6.324439332	2.54154E-10	2.46339E-09
ZDHHC7	66.59688242	-2.25044031	0.355852109	-6.324088719	2.54731E-10	2.4672E-09
HSPB8	39.96737667	1.474686298	0.233290929	6.321232921	2.59485E-10	2.51142E-09
NUMB	100.5385345	-1.178107413	0.186383522	-6.320877531	2.60082E-10	2.51538E-09
NKTR	353.3244063	0.913165073	0.14448219	6.320260462	2.61123E-10	2.52362E-09
TIMM8B	25.9099209	-2.30049929	0.364026048	-6.319600766	2.6224E-10	2.53259E-09
PMEPA1	249.7699585	0.935415577	0.14804738	6.318352775	2.64366E-10	2.55127E-09
APCDD1	11.65511091	-3.798996021	0.601319207	-6.317769291	2.65366E-10	2.55908E-09
PGD	138.8760332	-1.537894421	0.243430103	-6.317601646	2.65654E-10	2.56001E-09
NDUFAB1	28.78875735	-2.363898958	0.374249762	-6.316367304	2.67783E-10	2.57867E-09
AHNAK	107.9338788	1.239976599	0.196341677	6.315401902	2.6946E-10	2.59295E-09
KCNK6	17.47969776	2.318967314	0.367258317	6.314267652	2.71444E-10	2.61016E-09
NHP2	43.41629076	1.541164068	0.244135419	6.312742624	2.74133E-10	2.63412E-09
RAD54L	40.09952153	-1.504082185	0.238291448	-6.311943618	2.75553E-10	2.64586E-09
RAPGEF5	32.1680833	2.093721303	0.331753708	6.311071286	2.77111E-10	2.65891E-09
CFHR5	7.766095655	6.324620728	1.002508472	6.308795292	2.81216E-10	2.69636E-09
FRMD6	19.75712863	2.209676896	0.350332285	6.307374425	2.83809E-10	2.71927E-09
CETN3	370.4968593	-1.243756427	0.197259522	-6.305178134	2.87863E-10	2.75614E-09
MANF	74.18906165	1.427968799	0.226502906	6.304417115	2.8928E-10	2.76773E-09
LOC101120029	7.327462832	6.211311024	0.985367797	6.303545787	2.90912E-10	2.78135E-09

GATA3	10.94860279	6.191067592	0.982457587	6.301613088	2.94564E-10	2.81425E-09
ZC3H11A	213.9631685	0.984375865	0.156308527	6.297646589	3.02199E-10	2.88513E-09
POMGNT1	15.44055249	2.679634905	0.425509684	6.297471029	3.02541E-10	2.88634E-09
PRKAR2A	494.5387412	0.836853916	0.132898293	6.296950073	3.03559E-10	2.89399E-09
C6H4orf3	134.4548308	-1.109275241	0.176206771	-6.295304287	3.06797E-10	2.92278E-09
LOC101111713	10.16047112	6.112916345	0.971065775	6.295058999	3.07283E-10	2.92532E-09
LAMA4	10.0965584	6.0855189	0.966824613	6.294335931	3.08719E-10	2.93689E-09
PCDH11X	33.90874994	-1.693233167	0.269079768	-6.292681093	3.12029E-10	2.96627E-09
PRELP	7.108724367	6.183307975	0.982784485	6.291621478	3.14167E-10	2.98447E-09
INHBE	6.7050026	-6.325728416	1.005873507	-6.288791159	3.19948E-10	3.03723E-09
EAF1	33.84535935	-1.922403737	0.305701483	-6.288499868	3.20548E-10	3.04077E-09
JUND	449.4404702	1.291474271	0.205389692	6.287921554	3.21744E-10	3.04779E-09
SELENOW	139.5147566	-1.197181628	0.190392946	-6.287951603	3.21682E-10	3.04779E-09
LOC105608433	27.75786833	2.090190551	0.332475299	6.286754404	3.24172E-10	3.06861E-09
CLIP1	90.03326642	1.122731687	0.178616401	6.285714412	3.26349E-10	3.08704E-09
CSNK1G2	84.07439788	-1.261758204	0.200796044	-6.283780192	3.30438E-10	3.1235E-09
DONSON	33.3933745	-1.572593685	0.250386361	-6.280668327	3.37121E-10	3.18442E-09
DNASE1L3	8.108277328	6.349367913	1.011191934	6.279092721	3.40554E-10	3.21458E-09
LOC121816361	16.36323785	3.32771383	0.530076377	6.277800664	3.43396E-10	3.23912E-09
ATN1	21.20198775	2.082637178	0.331786861	6.277033313	3.45094E-10	3.25284E-09
AFAP1	29.19762862	-1.69898122	0.27084323	-6.272932207	3.54311E-10	3.33737E-09
LOC101120875	7.101956957	6.175191941	0.98452005	6.272286624	3.55784E-10	3.34888E-09
IRF2BP2	325.2762441	-0.902623338	0.14391429	-6.271950754	3.56552E-10	3.35375E-09
LOC101119572	7.336944362	6.223245955	0.992265984	6.271751784	3.57008E-10	3.35568E-09
GOT2	347.651095	-1.104603145	0.176312335	-6.2650361	3.72739E-10	3.50108E-09
APOD	7.824249971	6.285060821	1.003473312	6.263306402	3.76899E-10	3.53766E-09
TMEM30B	36.84261753	1.511288209	0.24143673	6.259562121	3.8606E-10	3.62111E-09
RPLP0	1696.435872	-1.118182424	0.178691841	-6.25760202	3.90942E-10	3.66433E-09
HES2	14.77270305	-2.932675468	0.46888062	-6.254631443	3.98456E-10	3.73214E-09
SREBF2	356.3447558	-1.40284211	0.224365778	-6.25247808	4.0399E-10	3.78133E-09
PSMD13	49.19621792	-1.846834374	0.295404102	-6.251891431	4.05511E-10	3.79291E-09
MAPK9	32.61791867	1.740826334	0.278526633	6.250125218	4.10124E-10	3.83337E-09
CMKLR1	7.06078019	6.170796585	0.987611448	6.248202769	4.15202E-10	3.87812E-09
MMP28	31.82856224	1.778583922	0.284794646	6.245145221	4.23407E-10	3.95199E-09
S100A9	7.225230355	6.186219346	0.991081293	6.241888925	4.32318E-10	4.03235E-09
PPP1R3G	11.63203308	-2.950085269	0.472844525	-6.23901751	4.40328E-10	4.1042E-09
MGRN1	20.5469652	-2.009762494	0.322165243	-6.238297071	4.4236E-10	4.11781E-09
PCNA	29.70963689	-1.9340849	0.310034895	-6.238281332	4.42404E-10	4.11781E-09
LOC105603754	83.77068513	1.554269567	0.249287739	6.234841615	4.52235E-10	4.20639E-09
LMO2	11.81245142	3.831775868	0.614651497	6.23406253	4.54492E-10	4.22443E-09
PRXL2A	56.01497424	-1.261424364	0.202357347	-6.23364747	4.55698E-10	4.2327E-09
CD86	6.84778348	6.129049233	0.983676839	6.230754849	4.64193E-10	4.30861E-09

JMJD1C	202.3719349	0.908628066	0.145863218	6.229315929	4.68476E-10	4.34535E-09
RAP1A	73.31618279	1.201182964	0.192860603	6.228244371	4.71691E-10	4.37214E-09
BCAS3	31.30693124	1.615843777	0.259479268	6.227255808	4.74676E-10	4.39675E-09
REM1	7.134541092	6.167638078	0.990444875	6.227139168	4.75029E-10	4.39698E-09
TM4SF5	57.56162629	1.840295846	0.295551659	6.226646982	4.76523E-10	4.40776E-09
GAS1	7.523499986	6.239616296	1.002148497	6.226239242	4.77764E-10	4.41618E-09
TNRC6B	313.3517724	0.952546911	0.153040049	6.224167582	4.84119E-10	4.47183E-09
PTPN18	137.7893407	1.38011659	0.22173966	6.224040334	4.84512E-10	4.47237E-09
GSDMB	22.72155329	2.058432952	0.330846772	6.221710861	4.91763E-10	4.53616E-09
PLAAT3	93.56866187	-1.211537798	0.194869453	-6.217176587	5.0618E-10	4.66593E-09
HNF1A	19.04268398	-2.259601651	0.363517707	-6.215932841	5.10206E-10	4.6998E-09
SMARCA4	305.9573792	-0.915642402	0.147324195	-6.215152938	5.12747E-10	4.71995E-09
ICAM3	11.81934975	5.733821629	0.922708684	6.214119069	5.16134E-10	4.74786E-09
PDLIM2	37.42028943	-2.124466661	0.341939671	-6.212986795	5.19868E-10	4.77893E-09
FDFT1	74.33012239	-1.330601085	0.214262523	-6.21014383	5.29361E-10	4.86285E-09
SFMBT2	6.834920351	6.112155326	0.984681933	6.207238219	5.39238E-10	4.95018E-09
KCNQ1	90.40518746	-1.303770033	0.210222058	-6.201870744	5.57959E-10	5.11852E-09
SEC62	250.7982467	0.963049557	0.155313854	6.200667446	5.62242E-10	5.15427E-09
NFKBIA	186.2399072	1.062448959	0.171464202	6.196331046	5.77945E-10	5.29459E-09
TEAD4	13.19340401	-2.797466966	0.45164303	-6.193977948	5.86644E-10	5.37061E-09
PIEZO2	10.91764181	4.191682936	0.676807599	6.193315419	5.89117E-10	5.38955E-09
ADCY5	7.242682094	6.183872291	0.998542019	6.192901426	5.90667E-10	5.40004E-09
CD6	6.783403951	6.1063236	0.986096611	6.192419214	5.92477E-10	5.41288E-09
FNDC3A	148.6345075	1.131906341	0.182803725	6.191921631	5.94351E-10	5.4263E-09
LOC101108520	38.65948511	1.558531108	0.25175118	6.1907599	5.98749E-10	5.46271E-09
LSP1	6.753158802	6.101707328	0.985932022	6.188770821	6.06352E-10	5.52831E-09
GPSM1	15.23735791	-2.677731649	0.432706687	-6.188329718	6.0805E-10	5.54002E-09
RPS14	1089.343988	-0.773291932	0.124974079	-6.187618544	6.10799E-10	5.56127E-09
RGS1	6.878272412	6.11815719	0.988931752	6.186632369	6.14631E-10	5.59234E-09
CBX5	105.1861258	-1.234928506	0.199618132	-6.186454585	6.15324E-10	5.59484E-09
SPEG	11.12085101	5.159065583	0.833970601	6.186148018	6.16521E-10	5.60192E-09
HSD17B4	164.2520374	1.560685826	0.252324894	6.185223352	6.20146E-10	5.63103E-09
SCAPER	92.56546041	1.145442477	0.185242376	6.183479737	6.27038E-10	5.68974E-09
CLEC4A	9.787183769	6.05506276	0.979584037	6.181259117	6.35923E-10	5.76645E-09
CHST12	13.78526546	3.268811215	0.528877023	6.180664071	6.38325E-10	5.78431E-09
TOLLIP	48.10370496	-1.648264543	0.267052799	-6.172054923	6.74081E-10	6.10418E-09
DNMT3A	41.05986261	1.63294605	0.264689373	6.169292064	6.85964E-10	6.20687E-09
TAC1	6.938383382	6.15160617	0.997147594	6.16920324	6.8635E-10	6.20687E-09
ZNF652	117.0389637	1.445858244	0.234585156	6.163468618	7.11685E-10	6.43163E-09
LAMA5	18.77070724	-2.270587469	0.368427534	-6.162914705	7.1418E-10	6.44982E-09
KDM6B	62.47008601	1.349794526	0.219055216	6.161891726	7.1881E-10	6.48725E-09
SLC25A30	30.81220368	-1.701150128	0.276142996	-6.160395702	7.25634E-10	6.54442E-09

CENPA	27.31876074	-1.804374564	0.292909177	-6.160184469	7.26603E-10	6.54873E-09
LOC132660031	30.28892804	1.75780481	0.285365715	6.159831823	7.28222E-10	6.55891E-09
ANK2	10.0739787	6.056647951	0.983497496	6.158274905	7.35416E-10	6.61924E-09
LOC101119648	11.71732507	5.714570592	0.928083401	6.157389073	7.3954E-10	6.65188E-09
DNAJB7	11.91629006	4.875105353	0.791867261	6.156467872	7.43853E-10	6.68617E-09
FOXF2	9.276289831	5.95069313	0.966756962	6.155314482	7.49287E-10	6.73049E-09
CADM1	104.5699528	1.197641089	0.19458798	6.154753695	7.51943E-10	6.74981E-09
GNAS	209.6755578	1.398633045	0.227332107	6.152377973	7.63297E-10	6.84713E-09
RIPOR2	9.621994374	6.028207992	0.979848302	6.152184967	7.64227E-10	6.85087E-09
LOC101113705	7.262729734	6.194072867	1.006897011	6.151644908	7.66834E-10	6.86964E-09
SLC30A1	156.171318	-1.383713067	0.22505776	-6.148257516	7.83388E-10	7.01324E-09
LSM12	84.63012989	-1.176353148	0.191420299	-6.145393954	7.97653E-10	7.13616E-09
IL10RA	6.871572398	6.104590152	0.993610464	6.143846482	8.05467E-10	7.20125E-09
MARF1	20.41963616	2.346758968	0.381982067	6.143636504	8.06533E-10	7.20596E-09
RNF144A	9.155799012	5.945961618	0.968198627	6.141262189	8.18683E-10	7.30963E-09
CDC5L	186.6123179	1.15763486	0.188525766	6.140459669	8.2283E-10	7.34176E-09
RPL22	552.869107	-0.902064809	0.146965389	-6.137940475	8.35982E-10	7.45412E-09
CAPN13	7.027798201	6.159171064	1.003651778	6.136760976	8.4221E-10	7.50465E-09
CAMSAP1	23.91781476	-1.986615353	0.323772352	-6.135840017	8.47104E-10	7.54323E-09
SESN3	175.4515089	-0.858797438	0.139986691	-6.13485061	8.52393E-10	7.58527E-09
MED4	18.48578663	-2.356760331	0.384176254	-6.134580956	8.5384E-10	7.58804E-09
RAB35	25.5564791	-1.98337406	0.323310226	-6.134584988	8.53818E-10	7.58804E-09
TASL	7.339536001	6.212409993	1.012712938	6.134423446	8.54686E-10	7.59052E-09
NIP7	47.31646248	-1.703065576	0.277676196	-6.133278988	8.6086E-10	7.64027E-09
NHSL3	34.30918444	1.812714463	0.295595634	6.132412836	8.65561E-10	7.6769E-09
AQP5	28.7941444	8.195629427	1.336584698	6.131769604	8.69069E-10	7.6978E-09
EEF1B2	29.41251481	-2.057630368	0.335564841	-6.131841354	8.68677E-10	7.6978E-09
DNAJB11	79.93275162	1.265263635	0.206366154	6.131158677	8.72413E-10	7.71863E-09
PLXDC2	11.29834943	5.193445871	0.847061903	6.131129085	8.72576E-10	7.71863E-09
ABHD2	37.4732041	-1.860240196	0.303485499	-6.129585102	8.81085E-10	7.78875E-09
KDR	6.767282856	6.090768672	0.993929886	6.127966126	8.90095E-10	7.86319E-09
VPS13B	97.34731219	1.036980796	0.169385727	6.122008134	9.24033E-10	8.15761E-09
NPB	14.12348974	-2.661555079	0.434763221	-6.121849665	9.24953E-10	8.16034E-09
TASOR2	31.84837434	1.655829365	0.270623969	6.118561371	9.44239E-10	8.325E-09
NLRC5	11.29566025	3.921309395	0.641257412	6.115031689	9.65378E-10	8.50576E-09
PATJ	108.6016661	0.925208323	0.15133666	6.113576993	9.74223E-10	8.57804E-09
SKIL	36.51806507	1.494798978	0.244514459	6.113335732	9.75698E-10	8.58537E-09
MAT2A	129.1603139	-1.314086754	0.215085509	-6.109601535	9.98802E-10	8.78289E-09
TIGIT	6.832497768	6.100278878	0.998738749	6.107982577	1.00898E-09	8.86658E-09
PIGU	36.3233842	-1.622016827	0.265607482	-6.106819033	1.01636E-09	8.92557E-09
EPHX1	25.64922331	1.97414122	0.323297582	6.106266583	1.01989E-09	8.95062E-09
RB1CC1	78.31999679	1.174971016	0.192429043	6.105996257	1.02161E-09	8.9599E-09

PLVAP	7.106078389	6.154584505	1.008077695	6.105268011	1.02628E-09	8.99495E-09
CKS1B	52.4368056	-1.419141244	0.23248267	-6.104288302	1.0326E-09	9.04436E-09
MCM3	16.94913933	-2.594196789	0.425096639	-6.102604798	1.04354E-09	9.13419E-09
ADAMTSL3	7.364507727	6.21948159	1.019456134	6.100783922	1.0555E-09	9.22678E-09
NR3C1	29.0432166	1.833427181	0.300519788	6.100853428	1.05504E-09	9.22678E-09
HES6	87.59327041	-1.763412161	0.289059216	-6.100522195	1.05723E-09	9.23586E-09
HEATR5B	57.43537794	1.324850108	0.217189225	6.099980836	1.06081E-09	9.26114E-09
IKZF1	6.535247603	6.044159041	0.990899312	6.099670239	1.06288E-09	9.2731E-09
PRELID3B	139.4967489	-1.272598909	0.208643577	-6.099391734	1.06473E-09	9.28321E-09
EBF1	9.317699104	5.959785682	0.977154375	6.099123979	1.06651E-09	9.29271E-09
AGPAT5	40.3012565	-1.486640623	0.243832144	-6.096983764	1.08088E-09	9.40779E-09
RBM15	76.75635127	-1.135779099	0.186286506	-6.09694778	1.08113E-09	9.40779E-09
ATG4B	39.39779149	-1.632094286	0.267736396	-6.095899958	1.08823E-09	9.46346E-09
ERAP2	22.74267026	1.938831541	0.318097539	6.095085008	1.09379E-09	9.50562E-09
LOC105611292	12.06707967	-2.806416226	0.460618366	-6.092714559	1.11012E-09	9.64123E-09
FASN	81.31412601	-1.327808996	0.217956687	-6.092077359	1.11455E-09	9.67341E-09
NUB1	215.8984234	0.939764779	0.154303764	6.090355502	1.1266E-09	9.77168E-09
PRMT1	174.272867	-1.311012718	0.215288081	-6.089574067	1.13211E-09	9.81313E-09
HMCN1	6.996083293	6.15060969	1.010640181	6.085855091	1.15871E-09	1.00372E-08
PRIMA1	6.354954826	6.018318589	0.989125663	6.084483312	1.16868E-09	1.01169E-08
GCG	31.81435995	1.772956873	0.291500555	6.082173235	1.18564E-09	1.02572E-08
FAM78A	9.344730606	5.972224036	0.982097191	6.081092674	1.19366E-09	1.03199E-08
PHTF2	32.3923418	1.610364758	0.264820142	6.08097535	1.19454E-09	1.03207E-08
PDE4DIP	38.3924273	2.012770649	0.331027813	6.080367171	1.19908E-09	1.03533E-08
RPL9	320.2053976	-1.146073194	0.188580806	-6.077358665	1.22178E-09	1.05425E-08
ELK3	29.29530645	1.699484534	0.279866018	6.072493346	1.25939E-09	1.086E-08
CSPG4	7.668923906	6.251655866	1.029622705	6.071792932	1.2649E-09	1.09005E-08
LOC121820342	17.37762641	2.463072962	0.40574144	6.070548181	1.27474E-09	1.09782E-08
RAB5A	48.51451896	1.541406501	0.253928383	6.070241074	1.27718E-09	1.09922E-08
RSPO3	7.172178333	6.159823418	1.014841842	6.069737338	1.2812E-09	1.10196E-08
NOL10	70.01018571	1.310008681	0.215862249	6.068725253	1.28929E-09	1.10821E-08
ERRFI1	97.16260803	-1.268100312	0.208966732	-6.068431563	1.29165E-09	1.10953E-08
CNBP	168.9027042	1.244862013	0.205171239	6.067429419	1.29974E-09	1.11575E-08
LOC114116445	27.0709561	2.10091612	0.346272236	6.067238142	1.30129E-09	1.11637E-08
MYOCD	6.473471662	6.035447501	0.994781738	6.067107256	1.30235E-09	1.11656E-08
LIMS2	6.349488049	6.009389547	0.991147769	6.063061165	1.33555E-09	1.14429E-08
LOC121819119	20.91444205	2.103538564	0.346974006	6.06252494	1.34001E-09	1.14738E-08
SENP3	49.98669479	-2.151782438	0.354998231	-6.061389177	1.34951E-09	1.15477E-08
LOC105616444	43.17224448	-1.639508819	0.270488973	-6.061277838	1.35044E-09	1.15483E-08
MTHFD2L	21.37582395	-1.935465408	0.319399125	-6.059707927	1.36369E-09	1.16514E-08
THBD	6.71724734	6.083896951	1.004002482	6.059643335	1.36424E-09	1.16514E-08
CSF2RB	6.345272649	6.017477642	0.993405906	6.057420845	1.38321E-09	1.18059E-08

SERINC1	46.71081053	1.476906954	0.243829463	6.057130822	1.38571E-09	1.18196E-08
CAP2	10.98216849	4.129615242	0.681895357	6.056083528	1.39476E-09	1.18892E-08
CCNK	24.81634042	-1.956716482	0.323243781	-6.053377043	1.4184E-09	1.20754E-08
PTBP1	188.0659297	-0.86545465	0.14296833	-6.05347107	1.41758E-09	1.20754E-08
RNF145	95.61941905	-1.050650459	0.173577994	-6.052901271	1.4226E-09	1.21034E-08
EBAG9	51.27662122	1.337175216	0.220930884	6.052459449	1.42651E-09	1.21267E-08
LONP1	17.1782688	-2.416393345	0.399246328	-6.052387157	1.42715E-09	1.21267E-08
TSPAN6	18.1163386	-2.07130454	0.342251975	-6.051987113	1.4307E-09	1.21491E-08
SYNE3	14.28297199	2.632962761	0.435147482	6.050736526	1.44185E-09	1.22361E-08
ARFGEF2	112.5204363	0.991392783	0.163909203	6.04842658	1.46267E-09	1.24049E-08
FARP1	76.38955901	-1.408595731	0.232899247	-6.048090521	1.46573E-09	1.24229E-08
HMBS	27.66858013	2.279671384	0.377277056	6.04243313	1.51807E-09	1.28584E-08
SYNGR1	9.588440407	-3.656361392	0.605170867	-6.041866177	1.52342E-09	1.28955E-08
CHMP2A	55.0164214	-1.569265205	0.259742352	-6.041622365	1.52572E-09	1.29068E-08
CCPG1	127.6270626	1.129656366	0.187032736	6.039885805	1.54223E-09	1.30262E-08
LOC105603415	110.8216384	1.014939466	0.168035877	6.040016472	1.54098E-09	1.30262E-08
TBCD	12.51950204	-2.769167631	0.458484268	-6.039831297	1.54275E-09	1.30262E-08
LOC101106419	23.53756604	-2.202135681	0.364672295	-6.038670088	1.5539E-09	1.3112E-08
MRPS2	126.7509107	1.575889695	0.26099062	6.038108545	1.55931E-09	1.31494E-08
SMOC2	96.33433608	-1.422011669	0.235548982	-6.037010457	1.56996E-09	1.32308E-08
ERP44	95.24794465	1.026349146	0.170073099	6.034753013	1.59206E-09	1.34002E-08
KANSL1L	36.20508245	1.707838327	0.28299964	6.034772087	1.59187E-09	1.34002E-08
TAGAP	6.382673795	6.015092062	0.99691009	6.033735761	1.60212E-09	1.34764E-08
NDUFA10	33.07061378	1.635409578	0.27123318	6.029533628	1.64433E-09	1.38228E-08
INPP4B	10.67037417	3.375330934	0.559987917	6.027506722	1.66508E-09	1.39884E-08
NDUFS4	89.16633719	-1.280649758	0.212484017	-6.027040423	1.66989E-09	1.402E-08
GINS1	26.74176593	-1.889104844	0.313528573	-6.02530362	1.68792E-09	1.41625E-08
ZNF664	26.68927774	-1.961060121	0.325484349	-6.025051982	1.69055E-09	1.41756E-08
BOD1L1	167.7758713	1.170397898	0.19430513	6.02350489	1.7068E-09	1.43029E-08
SCARA5	6.332108712	6.014067992	0.998835434	6.021079936	1.73257E-09	1.45056E-08
SLC7A1	113.2940968	-1.131555108	0.187933968	-6.021024935	1.73316E-09	1.45056E-08
IER5L	19.75688142	-2.222714898	0.369529217	-6.014990955	1.79897E-09	1.50469E-08
PIGS	124.4805242	0.858553447	0.142778466	6.013185803	1.81912E-09	1.5206E-08
LOC101104557	6.612182869	6.05630892	1.008316215	6.006358751	1.89736E-09	1.58501E-08
VARS1	49.40138684	-1.745040557	0.290651972	-6.003883426	1.92653E-09	1.60837E-08
CCND2	14.80761885	-2.5439931	0.4238069	-6.002717505	1.94042E-09	1.61896E-08
CDKN2AIPNL	118.5228832	-0.943383419	0.157205883	-6.00094221	1.96176E-09	1.63574E-08
DIPK2B	6.817390774	6.101887071	1.017136477	5.99908391	1.98434E-09	1.65353E-08
STK17B	19.76602922	2.155458231	0.359437942	5.996746526	2.0131E-09	1.67645E-08
SLC51B	9.306118189	5.970304035	0.995796595	5.995505571	2.02853E-09	1.68826E-08
MGME1	16.87379116	-2.132113609	0.355657996	-5.994842335	2.03683E-09	1.6941E-08
MBNL1	191.0407731	0.860063097	0.143485162	5.994090852	2.04627E-09	1.7009E-08

PRICKLE2	6.321588828	6.013848388	1.003400673	5.993466569	2.05414E-09	1.70638E-08
CCL25	39.84817553	4.826967796	0.80544329	5.992933151	2.0609E-09	1.71037E-08
LOC121820433	267.6824532	1.305529682	0.217846617	5.992884818	2.06151E-09	1.71037E-08
CDK11B	251.9663625	1.218270859	0.203344482	5.991167529	2.0834E-09	1.72746E-08
SAMM50	26.30890789	-1.856943555	0.310163839	-5.986976304	2.13778E-09	1.77145E-08
ILDR1	94.50698605	-1.191171875	0.199046989	-5.984375248	2.17222E-09	1.79888E-08
EDNRA	7.949623578	6.315217988	1.055303448	5.984267367	2.17366E-09	1.79896E-08
B2M	872.5104613	0.957827314	0.160074077	5.983650381	2.18191E-09	1.80467E-08
LOC101119517	8.453864817	5.85095905	0.978184217	5.981449044	2.21161E-09	1.8281E-08
KLHL42	38.31577116	1.486267456	0.24851208	5.980664824	2.22229E-09	1.83579E-08
WLS	56.46713675	-1.361487554	0.22775001	-5.977991197	2.25906E-09	1.86501E-08
CYYR1	6.430406527	6.036098318	1.009807543	5.977473983	2.26624E-09	1.86979E-08
TMEM208	38.47716305	-1.455619057	0.243637939	-5.974517211	2.30773E-09	1.90284E-08
ARHGDIB	6.256228716	5.981932888	1.001544267	5.972709429	2.33345E-09	1.9228E-08
CORO2A	57.94975661	1.698783799	0.284428843	5.972614386	2.33481E-09	1.9228E-08
LOC132657290	7.346209501	-5.868071895	0.982839317	-5.970530274	2.36484E-09	1.94633E-08
OSTM1	11.42860355	3.027605224	0.507205383	5.96918985	2.38435E-09	1.96118E-08
CCDC102B	6.306271921	5.989681101	1.003604316	5.968169929	2.39929E-09	1.97226E-08
HERC2	159.5934629	1.152344004	0.193099829	5.967607582	2.40758E-09	1.97785E-08
ATXN2	67.9119884	1.292537776	0.216713836	5.964260508	2.45744E-09	2.01758E-08
HOXA4	10.92574975	3.213272306	0.539092685	5.960519212	2.51438E-09	2.06306E-08
SLC51A	10.07186417	5.503839522	0.92350497	5.959729184	2.52656E-09	2.07178E-08
LOC114109663	37.65247458	1.497149056	0.251225445	5.959384637	2.5319E-09	2.07488E-08
LOC121820007	39.92662426	2.161703102	0.362766459	5.958938736	2.53881E-09	2.07927E-08
FKBP2	151.3361452	1.170528558	0.196442001	5.958647092	2.54335E-09	2.08171E-08
COLEC12	6.186702855	5.964152172	1.00098456	5.958285884	2.54897E-09	2.08504E-08
RIOK2	43.58007578	-1.54792146	0.2598454	-5.957086244	2.56775E-09	2.09911E-08
SMAD7	14.58358087	2.558540523	0.429545907	5.956384362	2.57879E-09	2.10686E-08
B3GALNT2	18.50628569	-2.016871697	0.338657478	-5.955491397	2.59292E-09	2.1171E-08
PHACTR2	45.06352532	1.457120662	0.244698699	5.954754426	2.60463E-09	2.12536E-08
RPL38	406.1734923	-0.735494529	0.12354499	-5.953252553	2.62865E-09	2.14366E-08
PDCD2L	9.457154771	-3.635719215	0.610991961	-5.950518909	2.67294E-09	2.17844E-08
HOXA3	8.944369796	5.89357789	0.990598559	5.949511874	2.68943E-09	2.19035E-08
SLC6A8	31.99648548	-1.745273553	0.293351515	-5.949427432	2.69082E-09	2.19035E-08
HDGFL3	46.37077792	1.574279553	0.264664589	5.948206205	2.71097E-09	2.20541E-08
TSR1	53.13905077	-1.422420316	0.239164381	-5.947458864	2.72337E-09	2.21415E-08
COG5	17.78600007	2.09130322	0.351786105	5.944814736	2.7677E-09	2.24882E-08
EIF4G3	85.63245394	1.302993175	0.21918757	5.944649017	2.7705E-09	2.24973E-08
LOC114111244	10.69801925	-3.369670087	0.566955291	-5.943449405	2.79086E-09	2.26488E-08
LOC101111463	5.89636415	5.911911559	0.994904563	5.942189613	2.8124E-09	2.28098E-08
C1H1orf52	25.91352865	1.813432391	0.305198098	5.94182075	2.81874E-09	2.28473E-08
SLC39A14	43.34972845	1.476889264	0.248612442	5.940528378	2.84105E-09	2.30142E-08

FAM53B	21.26868741	1.982202244	0.333761517	5.938977817	2.86805E-09	2.32188E-08
CLPTM1L	46.69815978	-1.672701359	0.281724641	-5.937362634	2.89644E-09	2.34345E-08
EPHB2	89.5721759	-1.206900417	0.203299963	-5.936550097	2.91082E-09	2.35366E-08
RABEPK	14.70115916	-2.416547238	0.407121457	-5.935691177	2.9261E-09	2.36459E-08
ITIH5	6.137645239	5.971602478	1.006237602	5.934584902	2.9459E-09	2.37915E-08
PDE1C	10.18939827	5.044544685	0.850143295	5.933758123	2.96078E-09	2.38972E-08
APOOL	57.77351181	-1.474067303	0.248489497	-5.932111089	2.99064E-09	2.41237E-08
DNAJC13	50.57552572	1.453049424	0.245012919	5.930501264	3.02011E-09	2.43467E-08
LOC105607861	6.240389612	5.999346874	1.011728671	5.92979822	3.03307E-09	2.44364E-08
RPLP1	1432.602999	-0.662761944	0.111798598	-5.92817762	3.06315E-09	2.46639E-08
TNFAIP8	99.55170363	1.0494397	0.177069088	5.926724494	3.09037E-09	2.48681E-08
COL4A5	5.984249931	5.933948539	1.001322735	5.926109867	3.10195E-09	2.49463E-08
SPTLC2	165.8004569	-1.059785557	0.178862442	-5.925143053	3.12026E-09	2.50784E-08
SIL1	38.97047259	1.419001323	0.239498843	5.924877578	3.1253E-09	2.51038E-08
IFRD2	90.93764615	-1.054505292	0.178002517	-5.924103248	3.14006E-09	2.52072E-08
CCDC134	21.7744969	2.211205197	0.373263415	5.923980519	3.14241E-09	2.52109E-08
SLC39A11	85.76841665	-1.388857955	0.234459318	-5.923662869	3.14849E-09	2.52445E-08
RAB3GAP2	87.65541803	0.986094062	0.166503171	5.922374071	3.17327E-09	2.5428E-08
ECSCR	10.88910799	5.08949756	0.859599804	5.920775621	3.20427E-09	2.5661E-08
AOPEP	111.524138	-0.919206887	0.155327397	-5.917867069	3.26143E-09	2.61032E-08
GTF2E2	64.88180997	-1.210234827	0.204577485	-5.915777218	3.30312E-09	2.6421E-08
P2RY1	6.087027395	5.957189995	1.007116708	5.915093997	3.31686E-09	2.65151E-08
ZDHHC14	20.286299	2.247624166	0.380017488	5.914528247	3.32828E-09	2.65904E-08
EFNB1	30.4686678	2.086142321	0.352804195	5.913031514	3.35868E-09	2.68173E-08
FAM81A	9.678590772	-4.188567728	0.708382275	-5.912863538	3.36211E-09	2.68286E-08
MRPS27	40.19242592	-1.691592792	0.286113645	-5.912310801	3.37341E-09	2.69028E-08
TTC7A	29.58993666	1.69265271	0.286306027	5.912040101	3.37896E-09	2.6931E-08
AMPD2	24.2494566	-1.916091312	0.324143207	-5.911249322	3.39523E-09	2.70445E-08
OXA1L	30.95334131	-1.70546117	0.288588617	-5.909661945	3.42811E-09	2.72901E-08
SPATA2	19.73113061	-1.99141583	0.336999573	-5.909253274	3.43662E-09	2.73416E-08
HSPA5	778.7100484	0.805900645	0.136455332	5.90596667	3.50585E-09	2.78758E-08
PIK3C2G	39.35395377	-2.299610017	0.389410832	-5.90535709	3.51884E-09	2.79624E-08
EDF1	83.54999415	1.152384553	0.195203595	5.903500671	3.55868E-09	2.82622E-08
REV3L	123.5542676	1.110270746	0.188119957	5.901929619	3.59274E-09	2.85158E-08
LOC114116078	5.903375343	5.903542595	1.000647852	5.899720451	3.64118E-09	2.88831E-08
CHMP4B	182.0242032	-0.958437079	0.162495466	-5.898238903	3.67402E-09	2.91263E-08
PLK1	34.91562099	-1.89170547	0.320747196	-5.897808289	3.68362E-09	2.91851E-08
MND1	29.69006296	-1.52907205	0.259273449	-5.897526549	3.68991E-09	2.92176E-08
FAM171A2	9.650838881	-4.076162864	0.691197889	-5.897244373	3.69622E-09	2.92503E-08
PHF12	32.62047885	1.529125287	0.259299443	5.897140667	3.69855E-09	2.92513E-08
GDF15	11.44064679	-3.240828161	0.549891728	-5.893575042	3.77928E-09	2.98722E-08
ATE1	62.20988522	1.143603447	0.194069254	5.892759548	3.79799E-09	3.00023E-08

MOCS1	6.057840199	5.92157471	1.005164824	5.891147969	3.83522E-09	3.02785E-08
TNFSF14	7.630908807	6.256657536	1.062072536	5.890988913	3.83891E-09	3.02898E-08
PCMTD2	52.78193393	1.413830635	0.240010227	5.890709951	3.8454E-09	3.03231E-08
VANGL1	27.91645043	-1.727547839	0.293394899	-5.888131808	3.90586E-09	3.07816E-08
ITGA4	5.8560189	5.881027377	0.998854963	5.887769088	3.91444E-09	3.08311E-08
UBE2J2	29.57283836	-1.562136262	0.265427399	-5.885361761	3.97184E-09	3.12648E-08
ZCCHC17	89.67294152	1.219761241	0.207326111	5.883297759	4.02171E-09	3.16387E-08
APLF	23.00396371	1.907203214	0.3242203	5.882429977	4.04286E-09	3.17864E-08
ARHGEF2	58.22363238	1.218236145	0.207173437	5.88027194	4.09593E-09	3.21847E-08
EIF2S1	24.80830125	-1.872063886	0.318426383	-5.879110487	4.12477E-09	3.23923E-08
VAPB	122.8838489	-0.942797336	0.160402912	-5.877682188	4.16051E-09	3.26538E-08
ACAD8	16.69629307	-2.775469569	0.472578277	-5.873036707	4.27884E-09	3.35628E-08
ZFYVE26	18.70770951	2.044703091	0.348310567	5.870344703	4.3489E-09	3.40923E-08
ZNF865	35.85902092	-1.71122848	0.291602747	-5.86835515	4.4014E-09	3.44836E-08
BRMS1L	28.94809747	2.371415696	0.404202302	5.866902992	4.4401E-09	3.47665E-08
IL18R1	7.86508077	5.727878311	0.976333281	5.866724432	4.44488E-09	3.47836E-08
AASS	42.64911861	1.36905469	0.233391255	5.865921102	4.46646E-09	3.4932E-08
LOC105605886	39.67949795	-1.352246342	0.230537893	-5.865614215	4.47473E-09	3.49762E-08
KRCC1	106.9790807	1.488979282	0.253875359	5.865001194	4.49129E-09	3.50851E-08
SLAMF7	5.989297673	5.939456882	1.012756481	5.864644656	4.50096E-09	3.51401E-08
PRRG1	32.00073511	-1.611343914	0.27479126	-5.863883418	4.52165E-09	3.5281E-08
LCP2	8.022938905	5.732986702	0.978437548	5.859328185	4.64743E-09	3.62413E-08
MED16	23.34649833	-2.202910482	0.376321289	-5.853802452	4.80459E-09	3.7445E-08
CRIM1	21.1529704	2.073843439	0.354496833	5.850104274	4.91265E-09	3.82648E-08
JAK2	40.38735636	1.563749088	0.2673711	5.848609238	4.957E-09	3.85878E-08
DHRS3	9.255646475	5.410047499	0.925252614	5.847103179	5.00207E-09	3.8916E-08
IRX3	9.844990565	-5.180820033	0.886209821	-5.846042223	5.03406E-09	3.91421E-08
ACOT12	8.227469149	5.784078526	0.989432506	5.845854559	5.03974E-09	3.91635E-08
VCF1	39.60107809	-1.510784811	0.258503526	-5.84434895	5.08553E-09	3.94964E-08
EP300	176.8283858	1.129886463	0.193503655	5.83909623	5.24847E-09	4.07153E-08
NFE2L2	217.1702646	1.231265147	0.210865823	5.839092989	5.24858E-09	4.07153E-08
PDE3A	9.862414348	6.026365707	1.032181443	5.838475153	5.26807E-09	4.08428E-08
NIBAN2	20.5154803	-2.335467922	0.400084768	-5.837432739	5.30113E-09	4.10753E-08
NAGS	12.41049061	2.627660219	0.450183197	5.836868709	5.3191E-09	4.11906E-08
EMID1	5.59882974	5.832455497	0.999329636	5.836367989	5.3351E-09	4.12906E-08
XPO5	29.32152053	-1.590858224	0.272655687	-5.834678308	5.38944E-09	4.16871E-08
CYFIP2	9.746899947	4.290554437	0.735626489	5.832517592	5.45972E-09	4.22063E-08
RNF152	8.378894071	5.823837015	0.99886604	5.830448513	5.52786E-09	4.27083E-08
TMSB4X	736.1785884	0.859631176	0.147454973	5.829787628	5.5498E-09	4.2853E-08
LOC101118216	9.436958003	-4.022243488	0.690164208	-5.827951436	5.61119E-09	4.32771E-08
TRAF2	13.67332785	-2.643610184	0.453602442	-5.828033399	5.60844E-09	4.32771E-08
LOC443398	10.50500487	4.639254405	0.796161021	5.827030315	5.64224E-09	4.34914E-08

C5H1orf35	147.8837044	1.659560724	0.284849726	5.826092052	5.67403E-09	4.37113E-08
ADAM15	23.70925922	-1.716583164	0.294662874	-5.825583466	5.69134E-09	4.38194E-08
FXR1	152.1256572	-0.922760832	0.158615824	-5.817583706	5.97044E-09	4.59418E-08
LOC101120874	16.60946721	2.273861929	0.391096108	5.81407456	6.09702E-09	4.68619E-08
ZNF281	40.18186055	1.603360169	0.275768098	5.814161171	6.09387E-09	4.68619E-08
LOC132659584	11.72497232	2.82562791	0.486070272	5.81320865	6.12866E-09	4.7078E-08
WDR33	80.46372279	-1.077651733	0.185415634	-5.812086645	6.16989E-09	4.73675E-08
HNRNPLL	35.93029808	1.383625791	0.238080059	5.811598812	6.1879E-09	4.74784E-08
HCFC1	75.78634582	1.025027375	0.176396152	5.810939555	6.21232E-09	4.76385E-08
MORF4L1	406.1414027	0.942900846	0.162369324	5.807136629	6.35503E-09	4.87049E-08
SNRPD3	375.2596916	-1.278427101	0.220161049	-5.806781476	6.36852E-09	4.87803E-08
ITGA2	48.44133136	-1.454174676	0.250439334	-5.806494738	6.37943E-09	4.88079E-08
WDR5	18.48405609	-2.684270144	0.462282624	-5.806556438	6.37708E-09	4.88079E-08
PHF10	31.72640789	-1.606134107	0.276632402	-5.806023057	6.39742E-09	4.89175E-08
BIN2	9.685316921	3.681503754	0.634115967	5.805726315	6.40876E-09	4.89762E-08
TNFRSF11A	10.84604381	4.194298748	0.722470413	5.80549552	6.41759E-09	4.90157E-08
IKZF3	6.404595166	6.03481212	1.039669899	5.804546351	6.45405E-09	4.9266E-08
NGDN	40.11756126	-1.511743493	0.260509536	-5.803025557	6.51289E-09	4.96868E-08
DEDD2	22.84295508	1.809567293	0.312229184	5.795637902	6.8062E-09	5.18948E-08
LACTB	58.77704438	1.339676338	0.231224141	5.793842868	6.87938E-09	5.24229E-08
VAV2	158.9994983	1.28438034	0.221709325	5.79308219	6.91063E-09	5.2631E-08
LOC101113279	5.4419061	5.797821118	1.001449714	5.789428103	7.06265E-09	5.37582E-08
RELN	5.880449891	5.902524778	1.019580196	5.789171664	7.07344E-09	5.38097E-08
RAD21	148.504841	-0.876675667	0.151578503	-5.783641152	7.31008E-09	5.55782E-08
MINDY2	22.15690083	2.145683605	0.371017638	5.78323881	7.32759E-09	5.56797E-08
ASF1B	16.14101101	-2.167336792	0.374920884	-5.780784386	7.43531E-09	5.64661E-08
SGIP1	10.74958991	3.067069688	0.530589468	5.780494849	7.44812E-09	5.65313E-08
SNX1	79.48916176	-1.110803019	0.192183089	-5.779920733	7.47358E-09	5.66923E-08
GNS	31.91736664	-1.432412692	0.247905271	-5.778064695	7.55648E-09	5.72724E-08
SLC30A4	12.85133091	2.688598886	0.465315158	5.778016984	7.55862E-09	5.72724E-08
CNPY3	13.10604384	2.512213796	0.434826756	5.777505094	7.58164E-09	5.74143E-08
AQP8	8.748014891	-4.251582777	0.735900149	-5.777390832	7.58679E-09	5.74208E-08
CEP290	156.9743976	1.014200105	0.175558207	5.777001954	7.60434E-09	5.7521E-08
LOC132657483	20.5766799	1.965322222	0.340227682	5.776491224	7.62745E-09	5.76632E-08
NDUFA1	95.39978081	-1.428520707	0.247318245	-5.776042543	7.6478E-09	5.77844E-08
PANX1	73.0845353	1.051277921	0.182067786	5.774101749	7.73647E-09	5.84213E-08
LAP3	144.477798	1.0884034	0.188526525	5.773210962	7.7775E-09	5.86979E-08
CCND3	98.73753213	1.140230299	0.197507252	5.77310597	7.78235E-09	5.87014E-08
EIF2AK2	95.65774284	1.277237467	0.221283396	5.771953485	7.83577E-09	5.9071E-08
ERCC3	49.36747869	-1.552993583	0.269110395	-5.770842045	7.88764E-09	5.94285E-08
INAVA	44.08141494	1.505884547	0.260957414	5.77061416	7.89831E-09	5.94754E-08
KLHL20	64.30664423	1.329508486	0.230507351	5.767748741	8.03375E-09	6.04612E-08

SACS	5.698324591	5.864687631	1.017257256	5.765196166	8.15629E-09	6.13489E-08
LOC101102001	5.614113875	5.824749192	1.010363598	5.765003019	8.16564E-09	6.13847E-08
JPH2	5.517524175	5.792422484	1.004919024	5.764068888	8.21099E-09	6.16876E-08
LDHA	29.72709884	-1.658939698	0.287811338	-5.763983129	8.21516E-09	6.16876E-08
F2RL2	11.3486569	3.044533633	0.528221304	5.763746385	8.2267E-09	6.17395E-08
TESC	5.427578646	5.79480345	1.006100303	5.759667732	8.42797E-09	6.32145E-08
EXOSC2	31.35458326	-1.743004103	0.302718745	-5.757833401	8.52004E-09	6.38334E-08
SLC2A4	8.281507624	5.787365653	1.005120045	5.75788502	8.51743E-09	6.38334E-08
POMGNT2	11.41336707	-2.899153457	0.503547302	-5.757460013	8.5389E-09	6.39389E-08
BYSL	27.10219203	-1.907319591	0.331290472	-5.757242515	8.5499E-09	6.39855E-08
ROBO2	10.26720754	3.144781606	0.546251151	5.757025129	8.56092E-09	6.4032E-08
SLC1A5	65.12254973	-1.933227995	0.335846551	-5.756283604	8.59859E-09	6.42778E-08
LOC121818849	54.7121868	2.105344443	0.365830023	5.75497994	8.66521E-09	6.47396E-08
PTGR1	46.79181402	1.426007904	0.247848342	5.7535503	8.73884E-09	6.52533E-08
PLP2	191.9766137	-1.225898806	0.213072408	-5.753437606	8.74467E-09	6.52604E-08
TBC1D10B	15.37847733	3.421732543	0.595190847	5.748967006	8.97903E-09	6.6972E-08
ASPN	5.401179951	5.782928428	1.00625853	5.746960899	9.08617E-09	6.77045E-08
PTGIR	7.591891952	5.651345332	0.983366255	5.746938443	9.08738E-09	6.77045E-08
ZDHHC3	70.54120016	-1.050440206	0.18278608	-5.746828241	9.0933E-09	6.77109E-08
ABCB8	70.24841444	1.067195284	0.185751454	5.745286309	9.17656E-09	6.82928E-08
RBM10	68.97073675	-1.157828437	0.201541923	-5.744851598	9.20016E-09	6.84304E-08
APPL2	27.07366419	1.764997063	0.307282021	5.743899545	9.25207E-09	6.87782E-08
PLLP	13.19502265	3.279929896	0.571155337	5.742623216	9.3221E-09	6.92602E-08
NCALD	7.554212687	5.670618655	0.987839006	5.740427967	9.44376E-09	7.00514E-08
NUP85	32.94735899	-1.660051545	0.289183453	-5.740479017	9.44091E-09	7.00514E-08
PDK1	18.45067205	-1.9821858	0.345303379	-5.740418196	9.4443E-09	7.00514E-08
SLA	5.659026565	5.835993661	1.016959484	5.738668798	9.54236E-09	7.07394E-08
LOC105611355	9.146449989	4.485130199	0.78161873	5.738258346	9.56551E-09	7.08718E-08
LOC101108781	7.000113497	6.149402453	1.072034198	5.736199893	9.68244E-09	7.16983E-08
GSDMD	26.691531	1.853983915	0.323412179	5.732572971	9.89185E-09	7.32084E-08
SUDS3	126.6404658	1.076645266	0.187816376	5.732435528	9.89987E-09	7.32272E-08
ZNF692	23.68671601	-1.894049612	0.330530684	-5.73032915	1.00236E-08	7.41014E-08
FZD1	9.688004576	4.921583767	0.858985342	5.729531725	1.00708E-08	7.4385E-08
GAMT	32.78208948	1.64141911	0.286485895	5.729493621	1.00731E-08	7.4385E-08
KCNN3	9.668177757	4.590088031	0.801247983	5.728673431	1.01219E-08	7.47042E-08
CDH17	296.5959175	1.082921968	0.189050977	5.728200856	1.01501E-08	7.48712E-08
NOP53	127.1306733	-1.435336096	0.250592671	-5.727765661	1.01762E-08	7.50221E-08
ECRG4	5.907524529	5.91614084	1.033001033	5.727139326	1.02138E-08	7.5258E-08
PPP1CA	51.43577668	1.25437853	0.219056118	5.726288511	1.02652E-08	7.55946E-08
LOC101107641	16.84009717	2.451760591	0.428187244	5.725907596	1.02882E-08	7.57227E-08
TMEM47	7.288390358	5.612191305	0.98021642	5.725461429	1.03153E-08	7.58802E-08
CRISPLD2	9.059087642	5.323766794	0.92987219	5.725267247	1.03271E-08	7.59023E-08

LOC101115252	46.28177449	1.766869773	0.308611397	5.725225275	1.03297E-08	7.59023E-08
STT3A	116.7440575	-0.967259979	0.168988838	-5.723809874	1.04161E-08	7.64956E-08
ARSB	10.2692318	3.411855775	0.596382749	5.720916275	1.05951E-08	7.77673E-08
SKAP1	9.156990192	3.945827569	0.690127976	5.717530233	1.08083E-08	7.92889E-08
SMARCB1	17.97140982	-1.973482568	0.345187347	-5.717134731	1.08335E-08	7.943E-08
PTGER3	5.670209267	5.821510191	1.018407944	5.716285133	1.08878E-08	7.97842E-08
HIVEP2	80.86593204	0.99806064	0.174617756	5.715688163	1.09261E-08	8.00209E-08
APOA4	10.32117992	4.652397259	0.81412816	5.714576019	1.09978E-08	8.05019E-08
SHOC2	119.3726821	0.925010763	0.161900277	5.713460045	1.10702E-08	8.09875E-08
LOC114114896	5.237597504	5.736095668	1.004213836	5.712026129	1.11639E-08	8.16125E-08
ZC3HAV1	115.8324415	1.168739383	0.204612449	5.711966142	1.11678E-08	8.16125E-08
TGFBI	9.286414097	4.554369676	0.797588378	5.710175579	1.1286E-08	8.24307E-08
CKAP5	134.5845463	-0.994823173	0.17426536	-5.708668505	1.13863E-08	8.31184E-08
LPIN2	90.63754193	1.153111875	0.202001035	5.708445385	1.14013E-08	8.3182E-08
LOC105613002	7.188231075	5.593474219	0.979884594	5.708298973	1.14111E-08	8.32082E-08
MX1	70.30605622	-1.191885041	0.208862114	-5.706564087	1.1528E-08	8.40145E-08
RSBN1L	127.4832742	1.275168093	0.223556044	5.704019759	1.17015E-08	8.52325E-08
NTNG1	5.629906407	5.814792698	1.019561446	5.703229283	1.17559E-08	8.55822E-08
TLNRD1	38.95572662	-1.436683112	0.251916377	-5.703015937	1.17706E-08	8.56428E-08
LOC114114532	20.80343142	-2.167094988	0.38018363	-5.700127037	1.19718E-08	8.70595E-08
LOC114117340	28.76722932	1.833437564	0.321723294	5.698802655	1.20652E-08	8.76907E-08
ZMYND8	114.1672943	1.031688841	0.181044563	5.698535337	1.20841E-08	8.77805E-08
RERG	5.677964194	5.837442757	1.024437158	5.698195066	1.21082E-08	8.79081E-08
SLC39A1	35.63023235	-1.716452275	0.301242573	-5.697907355	1.21287E-08	8.80087E-08
ZBTB34	30.00551421	1.595499283	0.280053462	5.69712393	1.21845E-08	8.8366E-08
LHFPL6	5.544681239	5.808010625	1.019768667	5.695419769	1.23069E-08	8.92048E-08
AURKA	37.75194594	-1.360765463	0.238975568	-5.694161434	1.2398E-08	8.98164E-08
TCOF1	182.9570582	1.557153269	0.273474338	5.693964857	1.24123E-08	8.98712E-08
DIS3	58.47226326	-1.158548004	0.203656057	-5.688748082	1.27974E-08	9.26097E-08
AP1G1	73.74323241	-1.129804481	0.198640188	-5.687693384	1.28767E-08	9.31329E-08
STK16	55.88422493	-1.344472934	0.236416765	-5.686876458	1.29384E-08	9.35287E-08
CDH11	5.168280407	5.713906606	1.004860482	5.686268602	1.29845E-08	9.38113E-08
MRPL30	23.21144394	-1.672160588	0.294163396	-5.684461796	1.31225E-08	9.47573E-08
FAM107B	37.82080039	1.415640816	0.249054664	5.684056627	1.31537E-08	9.49309E-08
LOC101112122	32.16927103	-1.601998684	0.2818743	-5.683379727	1.32059E-08	9.52561E-08
CD19	5.432479259	5.785724389	1.018141093	5.682635178	1.32635E-08	9.56203E-08
POC1A	15.37851368	-2.364286236	0.416109569	-5.68188384	1.33219E-08	9.59896E-08
MPV17L	9.460196083	3.853939121	0.678357562	5.681279813	1.33691E-08	9.62774E-08
TTYH3	81.26011675	-1.403495609	0.247074154	-5.680463071	1.34331E-08	9.66862E-08
CRABP2	6.524070412	-5.690678255	1.001936866	-5.679677481	1.34949E-08	9.7079E-08
PPP2R2A	112.6059352	-1.149630838	0.202422896	-5.679351795	1.35206E-08	9.72117E-08
DACT1	5.554282144	5.793816006	1.020554593	5.677125012	1.36977E-08	9.84322E-08

CXADR	171.6683851	-0.894232819	0.157528403	-5.676644974	1.37362E-08	9.86557E-08
PREX1	9.635660062	4.13215583	0.728251688	5.674076556	1.39439E-08	1.00093E-07
THOC3	45.85145147	-1.435290267	0.253059578	-5.671748436	1.41347E-08	1.01409E-07
TRIM35	54.48209983	1.233645976	0.217519243	5.671433744	1.41607E-08	1.01541E-07
CCDC136	10.49115461	5.566553372	0.981729804	5.670148089	1.42674E-08	1.02251E-07
PPP3R1	103.4434982	-1.251259294	0.220703873	-5.669403417	1.43296E-08	1.02641E-07
SPAG9	156.27479	1.096893489	0.193532616	5.667744857	1.44689E-08	1.03584E-07
PEBP1	57.9990195	-1.278928846	0.225695023	-5.666624043	1.45638E-08	1.04208E-07
EMC1	142.2484891	-1.361813904	0.240371726	-5.665449626	1.4664E-08	1.04868E-07
CNTNAP2	53.23612761	-1.778727554	0.313968392	-5.665307715	1.46761E-08	1.0487E-07
IFT56	17.77487252	-1.879730356	0.331799331	-5.665262645	1.468E-08	1.0487E-07
TMEM80	12.77063414	-3.198494809	0.56491997	-5.661854741	1.49746E-08	1.06918E-07
BCAP29	33.7536681	1.613666708	0.285149249	5.659024925	1.52235E-08	1.08637E-07
LOC101104661	33.6723514	-2.058379825	0.363794859	-5.658078376	1.53077E-08	1.0918E-07
LOC101113346	69.40106132	1.574567343	0.278305466	5.657694646	1.5342E-08	1.09366E-07
MAPK13	67.31810623	1.181924505	0.208947414	5.656564408	1.54433E-08	1.09971E-07
WAPL	155.7611497	1.046537661	0.185012105	5.656590219	1.5441E-08	1.09971E-07
LOC114117945	5.604065034	5.838461743	1.032656398	5.653828084	1.56913E-08	1.11677E-07
MAN1A2	156.7491354	1.052735865	0.186236072	5.652695828	1.57951E-08	1.12356E-07
GID4	21.38856716	-1.870687601	0.330957159	-5.652355751	1.58264E-08	1.12519E-07
MICU3	16.54346743	2.32512713	0.411476532	5.650691953	1.59803E-08	1.13553E-07
LOC132659095	8.505500149	-3.492550182	0.618153233	-5.649974789	1.60471E-08	1.13967E-07
LOC114118411	7.047247095	5.569533082	0.986735977	5.64440054	1.65758E-08	1.17659E-07
SLC24A3	8.014775186	5.738217328	1.017036994	5.642093024	1.67995E-08	1.19184E-07
GTF2F2	105.0398474	-1.007553306	0.178632576	-5.640367091	1.69688E-08	1.20321E-07
LOC132658724	5.722770963	5.84269096	1.035910674	5.640149395	1.69903E-08	1.20409E-07
CBX2	11.64877474	-2.758628982	0.489295754	-5.637958143	1.72078E-08	1.21887E-07
FAM20B	63.83382995	1.113579219	0.197538173	5.6372862	1.72751E-08	1.22298E-07
AEBP1	8.625171684	5.851791695	1.038208106	5.636434218	1.73607E-08	1.22839E-07
AGT	8.334710712	5.253680972	0.93243766	5.634350902	1.75719E-08	1.24268E-07
TMSB10	236.7792938	-1.128110603	0.200413913	-5.628903629	1.81359E-08	1.28188E-07
SAMD12	21.25599963	-2.233414715	0.396976252	-5.626066299	1.84366E-08	1.30245E-07
MIA2	54.31814434	1.289452305	0.229218214	5.625435612	1.8504E-08	1.30652E-07
PRRT1B	59.5609238	-1.262188323	0.22437796	-5.625277656	1.8521E-08	1.30703E-07
VMP1	127.3262961	-0.970386688	0.172576258	-5.622944316	1.8773E-08	1.32412E-07
LRP10	79.99162222	1.184379853	0.210652572	5.622432433	1.88287E-08	1.32735E-07
LOC105614315	7.391058956	5.618002857	0.999511724	5.620747333	1.90133E-08	1.33965E-07
DKC1	335.4901005	1.375548722	0.244789158	5.619320444	1.9171E-08	1.35005E-07
LOC101109377	8.815074912	4.159955988	0.74032195	5.619117451	1.91935E-08	1.35093E-07
MCP1	5.324723416	5.782046082	1.029525425	5.616224661	1.95175E-08	1.373E-07
LOC101103766	8.250684583	5.22427716	0.930346089	5.615412608	1.96094E-08	1.37802E-07
PITHD1	29.227343	-2.604356094	0.463783517	-5.615456353	1.96044E-08	1.37802E-07

GPA33	17.82810113	2.114923784	0.376653858	5.615032842	1.96525E-08	1.38032E-07
DHX15	58.54055792	1.125204192	0.20044282	5.61359192	1.98169E-08	1.39114E-07
LOC101119050	76.12776314	1.151119882	0.205154568	5.610988291	2.01174E-08	1.41149E-07
PCNX4	49.97354003	1.52116429	0.271237557	5.608236225	2.04399E-08	1.43337E-07
BAZ1B	221.4968515	1.324290369	0.236181259	5.607093357	2.05753E-08	1.4421E-07
GLI1	5.151385565	5.693102834	1.016479494	5.600804412	2.13359E-08	1.49385E-07
TMEM126A	20.64632492	-1.989769047	0.35526489	-5.600804085	2.1336E-08	1.49385E-07
RIF1	172.9198562	1.067762665	0.190677997	5.599821073	2.14573E-08	1.50156E-07
SRP72	302.6223664	0.993473672	0.177491807	5.597293132	2.17724E-08	1.52282E-07
TGFBR3	9.423412951	3.669402586	0.655632077	5.596740477	2.18419E-08	1.52608E-07
TMED3	82.8235532	1.259755447	0.225086305	5.596766302	2.18387E-08	1.52608E-07
FAM110D	5.151843127	5.712934523	1.021148457	5.594617003	2.21109E-08	1.54407E-07
ARHGAP45	8.333011227	5.253876932	0.939178449	5.594119984	2.21744E-08	1.54769E-07
RPRML	9.427608374	-3.969370852	0.709659871	-5.593342692	2.22739E-08	1.55383E-07
ELF2	159.1846655	1.002754203	0.179312786	5.59220691	2.24201E-08	1.56321E-07
MFAP4	5.323304485	5.727148949	1.024273258	5.591426803	2.25211E-08	1.56933E-07
SMCHD1	134.2287229	0.971408886	0.173734288	5.591348116	2.25313E-08	1.56933E-07
SLC35B2	8.39304817	-3.888667498	0.69601171	-5.587071947	2.3093E-08	1.60761E-07
PTPN3	52.04460603	-1.26791278	0.227000298	-5.585511515	2.33013E-08	1.62127E-07
RSF1	269.6575862	0.785058969	0.140609745	5.583247216	2.36069E-08	1.64167E-07
PPP2R1A	16.20459175	-2.015529919	0.361023683	-5.58281912	2.36651E-08	1.64486E-07
TFRC	121.9061625	-0.869250955	0.155816077	-5.578698746	2.42324E-08	1.68342E-07
VGLL4	102.4717586	1.213570064	0.217586563	5.577412722	2.44122E-08	1.69503E-07
PANK2	8.237331827	-3.664121495	0.657165416	-5.575645649	2.46613E-08	1.71144E-07
LAD1	32.74088392	1.520739394	0.272761181	5.575351251	2.47031E-08	1.71345E-07
RPL7A	690.0453188	-0.945165622	0.169530272	-5.575202656	2.47242E-08	1.71402E-07
EPB41L3	9.467903362	3.656018675	0.65590235	5.57402893	2.48914E-08	1.72472E-07
KLF2	4.84856889	5.628465774	1.0099936	5.572773706	2.50715E-08	1.7363E-07
ERBB2	45.35084722	1.566092723	0.2812005	5.569309875	2.5575E-08	1.77025E-07
LOC121820071	38.06831101	1.539723069	0.276562125	5.567367801	2.58616E-08	1.78824E-07
RP9	73.66420352	1.530996161	0.274992646	5.567407648	2.58557E-08	1.78824E-07
SRP19	46.85255849	1.199363529	0.215504856	5.565366607	2.61602E-08	1.80795E-07
LOC101116001	37.97262461	-1.470459995	0.264224452	-5.565192724	2.61863E-08	1.80882E-07
PPRC1	33.75817044	1.445486801	0.25977274	5.564428347	2.63013E-08	1.81582E-07
MXRA7	8.221604436	4.741527208	0.852305616	5.563177247	2.64907E-08	1.82795E-07
WWTR1	49.46466344	1.473857455	0.264979435	5.562157887	2.66459E-08	1.83771E-07
MDFIC	25.76786	1.802443827	0.324094463	5.561476772	2.67501E-08	1.84395E-07
RANBP10	20.25454928	-1.917268538	0.344804463	-5.560451622	2.69077E-08	1.85386E-07
LOC101102690	239.4883504	-1.154701991	0.207679606	-5.560016287	2.69749E-08	1.85753E-07
TTPAL	45.81475432	-1.216586971	0.218829429	-5.559521763	2.70515E-08	1.86184E-07
RUBCNL	5.099546434	5.703987859	1.026024622	5.559308943	2.70845E-08	1.86316E-07
MPC2	129.891725	1.09500465	0.196978904	5.558994516	2.71333E-08	1.86556E-07

HDLBP	70.78446133	1.45678626	0.262089514	5.558353858	2.72331E-08	1.87145E-07
LAMTOR2	123.1971137	1.113117255	0.200315967	5.556807446	2.74754E-08	1.88616E-07
MARCHF8	59.25977541	-1.508438125	0.271455924	-5.556843644	2.74697E-08	1.88616E-07
NCLN	60.61071763	1.08763995	0.195912616	5.551658539	2.82972E-08	1.94158E-07
TRIM59	15.67313096	-1.984521376	0.35750281	-5.551065119	2.83934E-08	1.94719E-07
MIGA1	10.87501244	2.829242055	0.509722755	5.550550823	2.84771E-08	1.95192E-07
IL1RL1	5.291112056	5.753874919	1.037356728	5.546669497	2.91162E-08	1.99471E-07
SLC66A3	11.96137749	2.469686748	0.445357325	5.54540502	2.93275E-08	2.00815E-07
HMGCS1	303.8614386	-0.945149838	0.170551647	-5.541722149	2.99511E-08	2.0498E-07
NSD2	112.7017115	-1.245855562	0.224861612	-5.540543577	3.01534E-08	2.06259E-07
LPAR5	6.866693223	5.544690986	1.000784958	5.540342047	3.01881E-08	2.06391E-07
CNPY4	24.79786242	-1.562890467	0.282150546	-5.539207657	3.03843E-08	2.07626E-07
NIT2	56.28938645	-1.252641218	0.226170308	-5.538486592	3.05097E-08	2.08376E-07
PRSS22	4.698517876	-5.739478637	1.036507161	-5.537326565	3.07124E-08	2.09653E-07
TAF10	33.25714034	1.363946271	0.246388593	5.535752506	3.09895E-08	2.11437E-07
PRKCE	30.28898334	1.61084471	0.291020166	5.535165242	3.10935E-08	2.12039E-07
TMCC3	15.61040208	2.195829987	0.39674165	5.534659606	3.11833E-08	2.12543E-07
LOC105614728	130.3834564	-1.707321925	0.308538596	-5.533576502	3.13766E-08	2.13751E-07
MDC1	35.0782808	1.491009804	0.269466208	5.533197705	3.14445E-08	2.14104E-07
WDR4	16.22636058	-2.229552657	0.402953033	-5.533033571	3.14739E-08	2.14196E-07
LOC132658839	11.8203544	-2.696186378	0.487392285	-5.531861013	3.16851E-08	2.15523E-07
TGM2	12.80968911	2.743253197	0.496069881	5.529973298	3.2028E-08	2.17744E-07
CHST6	30.16996572	1.503925953	0.27209703	5.52716783	3.25442E-08	2.21029E-07
CSNK1E	50.22352996	-1.293922133	0.234102049	-5.527171316	3.25435E-08	2.21029E-07
ANGPT1	4.91707754	5.621090391	1.017088767	5.526646811	3.26409E-08	2.21574E-07
KIAA2013	33.02701595	-1.423632553	0.257619644	-5.526102478	3.27423E-08	2.22149E-07
REL	96.75803942	0.945626766	0.17115335	5.525026334	3.29436E-08	2.23402E-07
LRR1	16.57218547	-1.96572154	0.355890547	-5.523387898	3.32524E-08	2.25381E-07
FCGR2B	5.260772181	5.766347582	1.044279218	5.521844618	3.35459E-08	2.27255E-07
RORC	66.38895723	-1.247201761	0.225928702	-5.520333401	3.38357E-08	2.29102E-07
PHB2	121.0391846	-0.933468112	0.16912014	-5.519556168	3.39857E-08	2.30001E-07
GNPTAB	80.19521855	1.168851755	0.211807064	5.518473907	3.41956E-08	2.31305E-07
PSTPIP2	15.63872064	2.395003166	0.434035551	5.517988469	3.42902E-08	2.31827E-07
BMP5	4.921705857	5.631528337	1.020669255	5.517486011	3.43883E-08	2.32373E-07
ATRNL1	8.009494533	5.146338555	0.932904481	5.516468897	3.45879E-08	2.33604E-07
SOCS3	11.05493789	2.937517861	0.532649859	5.514913435	3.48952E-08	2.3556E-07
AP4E1	26.00799119	1.833605959	0.332514391	5.514365721	3.5004E-08	2.36175E-07
RAE1	30.19011831	-1.604999669	0.291098747	-5.513591824	3.51583E-08	2.37097E-07
PEX1	32.9317902	1.607116745	0.291545945	5.512396161	3.53981E-08	2.38594E-07
CRADD	13.38502886	2.418346299	0.438765558	5.511704954	3.55374E-08	2.39412E-07
MTHFD1	31.41019896	-1.628717112	0.29555536	-5.510700632	3.57408E-08	2.40661E-07
CLEC3B	5.49145129	5.787173652	1.050310782	5.509963098	3.58909E-08	2.4155E-07

PLIN2	30.51005475	-1.546665818	0.280766187	-5.508732498	3.61427E-08	2.43122E-07
RPF2	45.0837923	1.535541298	0.278873032	5.506238041	3.66583E-08	2.46466E-07
TIAL1	133.8531687	1.093980477	0.198732951	5.504776488	3.69637E-08	2.48395E-07
LYVE1	4.957196712	5.639543337	1.024533469	5.504498885	3.7022E-08	2.48661E-07
UTP23	31.36351949	1.53350928	0.278639722	5.503555873	3.72206E-08	2.4987E-07
BTF3L4	72.77572923	-1.307060147	0.237544973	-5.502369215	3.74721E-08	2.51432E-07
UEVLD	46.35104841	1.349151651	0.245231113	5.501551715	3.76463E-08	2.52474E-07
LOC101122718	8.419943883	5.209883677	0.947016274	5.501366577	3.76859E-08	2.52613E-07
NRP1	8.840671326	3.444875157	0.626370575	5.499739762	3.80352E-08	2.54826E-07
HDAC11	21.71198138	2.012657498	0.36596765	5.499550301	3.80761E-08	2.54973E-07
LOC101121718	46.22534888	-1.454411194	0.264486778	-5.498993948	3.81964E-08	2.5565E-07
NR2F2	166.424451	1.108726504	0.201701146	5.496877551	3.86575E-08	2.58607E-07
RNF20	111.4543477	1.204705229	0.219171638	5.49662922	3.87119E-08	2.58841E-07
LOC132659610	10.48602864	2.874194388	0.523022828	5.495351701	3.89933E-08	2.60592E-07
SPAG7	114.6752394	-1.187669465	0.216165242	-5.494266588	3.92337E-08	2.62068E-07
PVR	12.82016725	-2.468843656	0.449482643	-5.492634017	3.95983E-08	2.64371E-07
TMEM150C	4.719388867	5.573704913	1.014857533	5.492105768	3.97169E-08	2.64989E-07
TRMT112	40.04248508	-1.71193005	0.311710823	-5.49204559	3.97305E-08	2.64989E-07
BCL2L1	42.2825318	-1.314465578	0.239376581	-5.491203744	3.99204E-08	2.66122E-07
IFNGR1	47.58723656	1.366022986	0.248847643	5.489394923	4.03313E-08	2.68728E-07
MYO1B	41.70395672	-1.486362666	0.270867748	-5.487411025	4.07867E-08	2.71627E-07
GPX3	5.247192648	5.71410438	1.041344608	5.487236728	4.0827E-08	2.7176E-07
PIK3IP1	8.499465529	-3.507308906	0.639290671	-5.48625072	4.10554E-08	2.73144E-07
AXL	5.108048135	5.710885584	1.041209854	5.484855492	4.13808E-08	2.75158E-07
SELPLG	4.770384928	5.582616085	1.017838366	5.48477663	4.13992E-08	2.75158E-07
ZNF205	32.19253495	-1.362164722	0.248411564	-5.483499643	4.16993E-08	2.77014E-07
MESD	154.970603	-0.914294906	0.166768101	-5.482432797	4.19516E-08	2.78552E-07
LHPP	31.3276121	-1.735217199	0.317112545	-5.471928588	4.45164E-08	2.95435E-07
RASSF9	10.77972488	2.88092994	0.52653849	5.471451746	4.46364E-08	2.96084E-07
IFFO2	14.57178407	-2.106064204	0.385017904	-5.470042254	4.49928E-08	2.98301E-07
RAB5C	85.63307133	-1.485526566	0.271610355	-5.469329648	4.51741E-08	2.99354E-07
SLX9	12.69500748	-2.335818694	0.427175479	-5.468054245	4.55003E-08	3.01366E-07
GRB10	27.33270176	-1.504132064	0.275167914	-5.466233474	4.59699E-08	3.04326E-07
CX3CL1	22.76067238	-1.725217966	0.315811195	-5.462814466	4.68644E-08	3.10094E-07
CBS	12.90445667	-2.302266583	0.421508939	-5.461963835	4.70896E-08	3.1143E-07
CHD5	6.772790674	5.498362829	1.006975538	5.460274478	4.75399E-08	3.14252E-07
RGCC	8.049702342	5.194869712	0.951518017	5.459560008	4.77316E-08	3.15364E-07
KDM2A	111.4060045	0.971470631	0.177996801	5.457798273	4.82075E-08	3.1835E-07
LOC101117764	7.328824307	5.607321033	1.027763562	5.455847277	4.87399E-08	3.21707E-07
CHAC1	8.003708268	-4.886116288	0.895960005	-5.453498213	4.93884E-08	3.25827E-07
BDP1	215.8645266	0.994401742	0.182348519	5.453303098	4.94427E-08	3.26024E-07
IARS1	44.48472394	-1.166247823	0.213875727	-5.452922782	4.95486E-08	3.26561E-07

FNIP1	58.34899597	-1.094762381	0.200775769	-5.452661873	4.96214E-08	3.2688E-07
LOC101113636	7.480569731	5.093393995	0.934167712	5.452333587	4.97131E-08	3.27323E-07
LOC101116991	4.686214673	5.576184602	1.022899326	5.451352306	4.99882E-08	3.28972E-07
ABI3BP	4.695883797	5.597938422	1.027011084	5.450708865	5.01694E-08	3.30002E-07
C1H1orf146	15.29437334	-2.02975056	0.372418859	-5.450182	5.03183E-08	3.30819E-07
GDI2	26.2669993	2.024389075	0.371508119	5.449111263	5.06221E-08	3.32653E-07
LOC132657645	17.74584403	6.909137778	1.268027799	5.448727372	5.07315E-08	3.33208E-07
KHK	18.8362132	2.158853542	0.39639253	5.446251829	5.14423E-08	3.3771E-07
PTGDS	4.731797117	5.56530948	1.02230754	5.443870129	5.21352E-08	3.42091E-07
LOC105609393	27.99643149	1.654715055	0.303986358	5.443385897	5.22772E-08	3.42854E-07
LOC101105810	4.635881431	5.54737316	1.019153157	5.443120225	5.23553E-08	3.4303E-07
LOX	8.062815443	5.211357555	0.957405941	5.443205784	5.23301E-08	3.4303E-07
MTPN	218.94483	0.900546367	0.165531734	5.440324619	5.31836E-08	3.48286E-07
USP13	34.68076838	-1.424404764	0.261941121	-5.437881465	5.39178E-08	3.52921E-07
BMP4	19.43292034	1.871369959	0.344177133	5.437229199	5.41155E-08	3.54042E-07
PTPN6	9.324166057	3.30577267	0.608327558	5.434198442	5.50433E-08	3.59936E-07
ZNF644	100.7929467	0.959982839	0.176676941	5.433549125	5.52441E-08	3.61072E-07
CAPZB	9.999044772	3.092280124	0.56929366	5.431783873	5.57935E-08	3.64485E-07
CCDC28A	11.03887699	2.511198599	0.462399894	5.430794058	5.61038E-08	3.66333E-07
LOC105608279	8.622135562	3.806165024	0.700885451	5.430509392	5.61934E-08	3.66739E-07
CHTOP	24.16353469	-1.578753501	0.290736907	-5.430179187	5.62975E-08	3.67239E-07
DNAJC7	217.4685146	-1.036699753	0.190987214	-5.428110775	5.69537E-08	3.71338E-07
NDUFS6	110.409582	-1.16473016	0.214636207	-5.426531601	5.74597E-08	3.74454E-07
CITED1	10.9803534	-2.480827887	0.457181544	-5.426351786	5.75175E-08	3.74649E-07
CD58	78.69013078	-1.137202036	0.209676389	-5.4236056	5.84087E-08	3.80268E-07
ERBB4	4.623160424	5.533497169	1.020485688	5.422415264	5.87991E-08	3.82623E-07
HMGB3	28.69457612	-1.762182367	0.325062928	-5.421049941	5.925E-08	3.8537E-07
TMOD3	243.6296113	1.047833591	0.19346522	5.416134195	6.09014E-08	3.95918E-07
LRRC25	4.816499264	5.583835774	1.031285685	5.414441268	6.14804E-08	3.99488E-07
MCRIP2	31.1362327	1.492630665	0.275719494	5.41358408	6.17756E-08	4.01211E-07
TMEM131	86.48385812	0.959651985	0.177278897	5.413233062	6.18968E-08	4.01803E-07
B9D2	17.05660313	2.011508679	0.371602609	5.413063926	6.19554E-08	4.01988E-07
EIF2AK3	42.51730765	1.336721821	0.246958295	5.412743149	6.20665E-08	4.02514E-07
RBP2	42.82105496	3.383281647	0.625307688	5.410587	6.28185E-08	4.07193E-07
ISOC2	28.3639293	2.074134444	0.383377979	5.410155401	6.29701E-08	4.07978E-07
ENDOG	30.9226076	1.488905791	0.275221855	5.40983852	6.30816E-08	4.08333E-07
TARS1	444.9354515	-0.84047472	0.15536077	-5.409825932	6.3086E-08	4.08333E-07
TMEM219	48.73875613	1.4765131	0.273025601	5.407965752	6.37446E-08	4.12396E-07
COL4A6	4.520374914	5.534984295	1.023727514	5.40669682	6.41977E-08	4.15126E-07
SLC2A3	7.980714162	4.29194911	0.793940492	5.40588262	6.449E-08	4.16815E-07
PIN1	74.88693836	-1.589220432	0.294003877	-5.405440385	6.46494E-08	4.17643E-07
AREG	13.27743208	-2.291386388	0.423975763	-5.404522123	6.49814E-08	4.19585E-07

TMEM50A	47.9454405	1.355379444	0.250792355	5.404388992	6.50297E-08	4.19694E-07
PHOSPHO2	38.49602483	-1.437167015	0.265950849	-5.403882025	6.52138E-08	4.2068E-07
OGFRL1	27.83751517	1.455005583	0.269283502	5.403248156	6.54448E-08	4.21966E-07
HDAC1	153.9856341	0.845235482	0.156454217	5.402446151	6.57382E-08	4.23653E-07
DLGAP5	34.38604119	-1.541506696	0.285382379	-5.401548274	6.60682E-08	4.25574E-07
GIPC2	52.98987848	1.074141471	0.198876827	5.401038851	6.62561E-08	4.26579E-07
ERCC6L2	25.3019356	1.63763605	0.303251925	5.400249477	6.65483E-08	4.28254E-07
GMPS	48.05354125	-1.170644015	0.216813764	-5.399306726	6.68989E-08	4.30304E-07
ANKH	59.74717531	-1.09765419	0.203307874	-5.398975292	6.70226E-08	4.30892E-07
LGI4	8.155520575	5.216082471	0.966383676	5.397527502	6.75655E-08	4.33965E-07
SPRY1	23.36056792	1.766251517	0.327229338	5.397595244	6.754E-08	4.33965E-07
SDCCAG8	23.55995738	1.750367185	0.324300364	5.397364237	6.7627E-08	4.34151E-07
SLC35D1	57.11458632	1.198652854	0.222091217	5.397119582	6.77193E-08	4.34535E-07
PPP4R3B	217.1204805	0.675848069	0.12533041	5.392530592	6.94723E-08	4.45569E-07
PEX11B	23.86965902	1.616775335	0.299866522	5.391649998	6.98136E-08	4.47544E-07
FOXA2	33.92208947	-1.37316751	0.254703622	-5.39123668	6.99744E-08	4.4836E-07
MED13	87.98586473	1.114270723	0.206756578	5.389287888	7.07374E-08	4.53031E-07
ZNF768	25.51693373	-1.633844156	0.303258319	-5.387631779	7.13921E-08	4.57005E-07
ROCK2	228.9631056	0.938483371	0.174218036	5.386832471	7.17102E-08	4.58822E-07
DUOX2	6.049051241	5.951774854	1.105055286	5.385952113	7.20621E-08	4.60853E-07
LOC106991099	29.29532228	1.574139058	0.292276645	5.385784615	7.21293E-08	4.61062E-07
FCMR	6.349679038	5.375193677	0.998101236	5.385419316	7.2276E-08	4.61778E-07
KRT19	297.7579399	-1.373533429	0.255163538	-5.382953386	7.32735E-08	4.67928E-07
ADAM10	93.56383246	0.918662489	0.170669584	5.382696018	7.33784E-08	4.68374E-07
PPCDC	9.848122914	3.0662218	0.569827738	5.380962696	7.40885E-08	4.72681E-07
RFXAP	45.02297073	-1.261804296	0.234514594	-5.380493712	7.42818E-08	4.73688E-07
NRIP1	87.24751091	1.144206354	0.212794159	5.377057152	7.57131E-08	4.82586E-07
PPIP5K2	144.3129733	0.904480683	0.168245657	5.375952625	7.61788E-08	4.85322E-07
C1QC	4.836346256	5.615032747	1.044613737	5.375223919	7.64876E-08	4.87057E-07
LOC114108595	8.09533192	4.714797068	0.877337187	5.373985209	7.70152E-08	4.90183E-07
ATP1A1	861.2171854	0.804923051	0.149801022	5.373281443	7.73165E-08	4.91867E-07
LOC101117971	4.68304607	5.569266428	1.036746999	5.371866457	7.79258E-08	4.95507E-07
LOC101107266	261.860992	-0.853826301	0.158987128	-5.370411516	7.85572E-08	4.99284E-07
LTB	9.194512423	3.170302283	0.590339081	5.370307315	7.86026E-08	4.99336E-07
MARK3	88.51477201	1.033053984	0.19241517	5.368880143	7.9227E-08	5.03064E-07
STOM	7.953791578	4.264966362	0.794869386	5.365619103	8.0672E-08	5.11995E-07
ALKBH3	20.46080352	-2.078462806	0.387480676	-5.364042476	8.13798E-08	5.16242E-07
SLC16A6	7.445854027	5.057693713	0.943291685	5.36174949	8.24198E-08	5.22591E-07
LOC132657189	9.396855728	-3.173856027	0.592017082	-5.361088596	8.27219E-08	5.2401E-07
NFIX	80.07772112	1.319042085	0.246038121	5.361128908	8.27035E-08	5.2401E-07
VEGFC	4.726165148	5.602582821	1.045215261	5.360219112	8.31211E-08	5.26289E-07
PCDH9	10.12260939	2.868026322	0.535098448	5.359810582	8.33093E-08	5.27231E-07

UBXN4	157.8429649	1.075881309	0.200739682	5.3595846	8.34135E-08	5.27641E-07
GOLM2	26.16417554	1.520167646	0.283743712	5.357537743	8.43637E-08	5.33399E-07
NOP56	250.5836333	1.307684627	0.244129853	5.356512569	8.48436E-08	5.36179E-07
TMTC1	4.543826823	5.517672857	1.030105726	5.356414119	8.48898E-08	5.36218E-07
GDE1	46.01119401	-2.083298773	0.38898945	-5.355669091	8.52404E-08	5.38178E-07
CNTNAP1	6.166465306	5.331351695	0.995756634	5.35407098	8.59971E-08	5.42608E-07
PPIL2	23.32523047	-1.805756156	0.337271343	-5.354015966	8.60233E-08	5.42608E-07
UCHL1	6.623247141	5.454001191	1.018795967	5.353379253	8.63266E-08	5.44265E-07
WDTC1	27.46361794	1.479289083	0.27645214	5.350977138	8.74806E-08	5.5128E-07
C3AR1	4.922393324	5.611298526	1.048713222	5.350651075	8.76384E-08	5.52014E-07
ZNF346	24.726505	-1.811709793	0.338632694	-5.350073476	8.79185E-08	5.53518E-07
CMC4	16.06242113	1.923338262	0.359509816	5.349890813	8.80073E-08	5.53816E-07
YWHAE	399.5426339	-0.929028893	0.173772023	-5.346251221	8.97946E-08	5.64797E-07
GTPBP10	21.04510002	-1.857051743	0.347441311	-5.344936488	9.04488E-08	5.68644E-07
CA8	15.45304146	2.292503811	0.428971621	5.344185259	9.08246E-08	5.70739E-07
MARCHF1	4.384051946	5.463756371	1.022807531	5.341920358	9.19671E-08	5.77375E-07
NLRP3	4.586781426	5.529512002	1.035103731	5.341988284	9.19326E-08	5.77375E-07
NKAP	84.26437471	1.136066632	0.212681073	5.341644263	9.21073E-08	5.77984E-07
UNC5D	11.90627386	-2.312163523	0.432888921	-5.34123977	9.23131E-08	5.79003E-07
BAK1	88.97362953	1.028243651	0.192514898	5.341112109	9.23781E-08	5.7914E-07
LOC101113211	4.809671829	5.624914211	1.053183892	5.340866162	9.25035E-08	5.79654E-07
POLB	28.02467273	-1.49324824	0.279687275	-5.338992417	9.34646E-08	5.85402E-07
ENPP1	9.849800133	3.07874365	0.57688971	5.336797656	9.46025E-08	5.92252E-07
BLNK	9.207380741	3.230967372	0.605443185	5.336532732	9.47408E-08	5.9284E-07
HEG1	10.25044411	2.706089247	0.50738673	5.33338593	9.63981E-08	6.02928E-07
SCT	8.030883272	4.003388756	0.750673836	5.333060197	9.65713E-08	6.03716E-07
SUMO3	74.05355861	-1.036338547	0.194326379	-5.332979244	9.66144E-08	6.03716E-07
TRIAP1	65.33775552	-1.182802073	0.221862976	-5.331227835	9.75509E-08	6.09283E-07
COL21A1	30.75844346	-2.319828863	0.435352044	-5.328627477	9.89577E-08	6.17781E-07
GARS1	109.3559653	-1.100252441	0.206498006	-5.328150446	9.92179E-08	6.19116E-07
CDKN1A	51.73508337	-1.630845124	0.306135506	-5.327200188	9.97382E-08	6.22072E-07
GRPEL1	39.44475218	1.254900597	0.235713742	5.323832998	1.01603E-07	6.33409E-07
YWHAQ	88.98601523	-1.060972184	0.199368315	-5.321669016	1.0282E-07	6.40693E-07
PTGS1	8.921801283	3.204513611	0.602189973	5.321433029	1.02953E-07	6.41226E-07
ENOX1	8.641292628	4.441235297	0.83476536	5.320339717	1.03574E-07	6.44791E-07
TBC1D19	19.54567099	-1.778055891	0.334206268	-5.320235025	1.03633E-07	6.44861E-07
CCN4	7.650176828	4.632660228	0.870974018	5.318941934	1.04372E-07	6.49159E-07
UBR4	156.4386042	1.199168009	0.225490212	5.318049059	1.04886E-07	6.52048E-07
REEP1	4.740883871	5.579057983	1.049225254	5.317311953	1.05311E-07	6.5439E-07
RPS15A	108.9496615	-2.533127054	0.476540717	-5.315657113	1.06273E-07	6.60059E-07
CCR9	4.773039353	5.580392375	1.04986513	5.315342149	1.06457E-07	6.60894E-07
GRAMD2B	32.54130511	1.490532942	0.280439662	5.31498623	1.06665E-07	6.6188E-07

RAB34	12.33629507	-2.43465406	0.458155437	-5.31403507	1.07224E-07	6.65037E-07
RECK	5.941187268	5.322208842	1.001763441	5.312839962	1.0793E-07	6.69105E-07
COQ4	25.19643579	1.671165704	0.314826118	5.308218116	1.10702E-07	6.85973E-07
NDUFA2	59.54737781	-1.054899887	0.198752144	-5.307615143	1.11069E-07	6.87927E-07
DTX1	4.576260789	5.527617208	1.041703071	5.306327076	1.11856E-07	6.92482E-07
HS2ST1	74.25080832	-0.984680458	0.185582633	-5.30588688	1.12126E-07	6.93835E-07
SMG1	201.4465925	0.747097118	0.140816178	5.305477872	1.12378E-07	6.9507E-07
LOC105602100	4.380053754	5.476167637	1.03229027	5.304871894	1.12752E-07	6.97061E-07
POGZ	83.73898002	-1.01120094	0.190668847	-5.303440796	1.1364E-07	7.02225E-07
TMEM126B	17.20333219	-1.911131064	0.360553583	-5.300546592	1.15456E-07	7.1312E-07
LGALS15	36.37784036	2.246411621	0.423933357	5.298973488	1.16456E-07	7.18958E-07
UBE2E1	89.0909941	-0.919316715	0.173522189	-5.297977861	1.17092E-07	7.22555E-07
GSTK1	78.00340542	-1.340073208	0.253187466	-5.292810217	1.20451E-07	7.42938E-07
PLGRKT	23.85214516	1.597946212	0.30193685	5.292319279	1.20775E-07	7.44592E-07
POU2AF1	5.846312303	5.300729139	1.001642704	5.292035893	1.20962E-07	7.45403E-07
PKHD1	8.574893229	-3.153428683	0.595924106	-5.291661556	1.2121E-07	7.46587E-07
SMARCD2	220.2660533	-0.888476794	0.167914722	-5.291238229	1.21491E-07	7.47973E-07
LAMB3	20.84839813	-1.693340572	0.320106461	-5.289929371	1.22364E-07	7.52998E-07
HCFC2	29.73393939	1.477419571	0.279307477	5.289581176	1.22597E-07	7.53739E-07
PRDM8	6.98271154	4.980590722	0.941570957	5.289660526	1.22544E-07	7.53739E-07
PTPRD	7.721931729	3.628487572	0.686226033	5.287598254	1.23933E-07	7.61603E-07
SLC25A20	23.66323891	1.711230427	0.323674139	5.286892654	1.24412E-07	7.64194E-07
LIF	7.234445381	-4.018476587	0.760108041	-5.286717636	1.24531E-07	7.64573E-07
SULF2	59.66613566	1.131134303	0.214006947	5.285502736	1.2536E-07	7.69312E-07
SPI1	4.253543961	5.425246162	1.026702596	5.284145751	1.26293E-07	7.7468E-07
KAT14	56.07423641	-1.284175422	0.243031267	-5.28399262	1.26398E-07	7.74973E-07
RARRES1	4.80961674	5.638158409	1.067065481	5.283797958	1.26533E-07	7.75441E-07
CCT3	127.4044647	-0.976718216	0.184874508	-5.283141656	1.26987E-07	7.77869E-07
MS4A15	7.721446911	5.104257085	0.966925547	5.278852233	1.29996E-07	7.95933E-07
RNF111	47.44870075	1.142956874	0.216750981	5.273133572	1.34114E-07	8.20772E-07
APLP2	406.1146823	-0.905598467	0.171811464	-5.270884981	1.35768E-07	8.30512E-07
ACVRL1	4.410866242	5.490210397	1.042113929	5.268339905	1.37663E-07	8.41722E-07
LOC121819799	5.446483911	5.777109067	1.096963536	5.266454972	1.39083E-07	8.50017E-07
RGS2	20.32089276	1.738997121	0.330224079	5.266112419	1.39343E-07	8.51215E-07
FBXO9	27.14263918	-1.522308902	0.289135592	-5.265034625	1.40163E-07	8.55833E-07
LOC132658251	9.319962457	5.365397957	1.019217587	5.264232118	1.40776E-07	8.59187E-07
ABCD2	4.786817955	5.58415905	1.0611779	5.26222705	1.42321E-07	8.6782E-07
UBC	69.94538775	-1.111766415	0.211269691	-5.262309099	1.42257E-07	8.6782E-07
MTMR11	12.16039081	-2.353138471	0.447190267	-5.26205207	1.42456E-07	8.6825E-07
CDC37	43.60216717	-1.297027277	0.246691189	-5.257696003	1.45871E-07	8.88659E-07
ZBTB37	18.02737559	1.987486794	0.378025982	5.257540196	1.45995E-07	8.89006E-07
SPP1	4.194608885	5.434228707	1.033631442	5.257414281	1.46095E-07	8.8921E-07

CD274	5.60388189	5.244179411	0.997589753	5.256849717	1.46544E-07	8.91537E-07
LAT2	4.347920596	5.446618066	1.036190652	5.256386031	1.46914E-07	8.9338E-07
KEF53_r02	1660.739536	-1.292175587	0.24591139	-5.254639024	1.48315E-07	9.01491E-07
NANS	113.313322	0.954410548	0.181662105	5.253767955	1.49019E-07	9.05356E-07
PCK2	24.48130711	-2.009726507	0.382544085	-5.253581442	1.4917E-07	9.05862E-07
SRSF11	562.2255636	1.135424206	0.216170528	5.252446831	1.50092E-07	9.11048E-07
GEM	4.287751101	5.421106654	1.033189705	5.24696155	1.54628E-07	9.38157E-07
AK6	23.59733789	-2.040038985	0.388863077	-5.246162734	1.553E-07	9.41533E-07
PRKAG2	19.52694093	1.861214582	0.354778441	5.246132146	1.55326E-07	9.41533E-07
LOC101116687	47.62037937	-1.55458937	0.296372804	-5.245384701	1.55957E-07	9.4493E-07
JAZF1	7.359051905	5.012350644	0.95579949	5.244144508	1.57009E-07	9.50876E-07
H3F3A	199.9657191	-1.132872645	0.216049806	-5.24357169	1.57498E-07	9.53402E-07
SMIM30	31.1866066	-1.50838243	0.287691132	-5.243061967	1.57933E-07	9.55608E-07
ALDH1A3	27.85974818	1.719332964	0.328054675	5.240995153	1.59713E-07	9.65938E-07
LSM6	24.14936133	1.578086761	0.301135115	5.240460776	1.60176E-07	9.68301E-07
TFCP2	66.85489459	-0.989521043	0.188840117	-5.239993817	1.60582E-07	9.70316E-07
LOC101122123	41.09395163	1.290311215	0.246259306	5.239644486	1.60886E-07	9.71715E-07
CLEC7A	4.175260266	5.395713828	1.03043263	5.236357693	1.63776E-07	9.88724E-07
LRPAP1	16.36848611	-1.958862342	0.374102871	-5.236159613	1.63952E-07	9.89339E-07
OLFML2B	8.633372916	3.933501649	0.751627139	5.23331509	1.66497E-07	1.00424E-06
ROCK1	342.2532162	0.735717227	0.140586169	5.233212017	1.66589E-07	1.00435E-06
CHRDL1	4.410055865	5.472454848	1.045754355	5.233021333	1.66761E-07	1.0048E-06
MRNIP	8.127232075	-4.821835349	0.921435164	-5.232962164	1.66815E-07	1.0048E-06
LOC105616782	58.8688954	1.333455436	0.254852578	5.2322619	1.67448E-07	1.00816E-06
TP53BP1	66.1093466	-1.131057704	0.216177768	-5.232072252	1.6762E-07	1.00874E-06
LOC114113987	80.56689617	-1.636810016	0.31286873	-5.231619083	1.68032E-07	1.01076E-06
P2RY13	4.186290871	5.411908726	1.034740444	5.230208947	1.69319E-07	1.01804E-06
SNX20	4.149574468	5.390876381	1.030847984	5.229555149	1.69918E-07	1.02119E-06
PEG3	8.528616073	3.405965974	0.651734885	5.225999177	1.73217E-07	1.04055E-06
SCEL	6.923208636	-5.199380242	0.995409752	-5.223356745	1.75708E-07	1.05504E-06
LOC105602450	19.17513309	1.757784305	0.336589835	5.222333299	1.76683E-07	1.06041E-06
LAS1L	84.34572552	1.386926845	0.265598747	5.221887749	1.77108E-07	1.06249E-06
LOC114111291	7.785640167	-3.282836815	0.628751871	-5.2211961	1.77771E-07	1.06551E-06
RDM1	8.592101624	-2.979946304	0.570736532	-5.221229299	1.77739E-07	1.06551E-06
ARHGEF25	4.054179121	5.37159519	1.02911794	5.219610874	1.79299E-07	1.07371E-06
KRT18	279.4868979	-1.340413787	0.256800398	-5.219671758	1.79241E-07	1.07371E-06
LOC105604994	11.6227725	2.570168668	0.492610577	5.217445156	1.81408E-07	1.08585E-06
CD83	4.179537697	5.39714666	1.034593235	5.216684664	1.82154E-07	1.08983E-06
LOC101107504	4.538181722	5.499630688	1.054510604	5.215339387	1.83481E-07	1.09727E-06
PPARA	66.86856707	1.135570124	0.217755481	5.214886531	1.8393E-07	1.09947E-06
CYSLTR2	4.211175164	5.418166764	1.03925341	5.213518388	1.85292E-07	1.10662E-06
VIP	4.029475834	5.365480391	1.029136489	5.213575118	1.85235E-07	1.10662E-06

BCAT2	18.95953335	2.619659053	0.502516704	5.213078556	1.85732E-07	1.10875E-06
SDHB	24.742605	-1.574124299	0.301995353	-5.212412328	1.86401E-07	1.11225E-06
CCDC66	45.07039507	1.161822395	0.222923179	5.211761289	1.87056E-07	1.11566E-06
AHSA2P	16.61393386	1.880923101	0.360946065	5.211091852	1.87732E-07	1.11919E-06
GPRIN3	30.91415633	1.586900924	0.304542704	5.210766509	1.88062E-07	1.12066E-06
GABARAP	217.9402269	0.95459212	0.183288437	5.208141528	1.90741E-07	1.13612E-06
PRKCD	35.63324319	1.388969083	0.26674707	5.207064063	1.91852E-07	1.14222E-06
ATIC	83.52081035	-0.968131145	0.186001376	-5.204967655	1.9403E-07	1.15468E-06
AOC3	4.093156702	5.390290473	1.035706349	5.20445827	1.94563E-07	1.15734E-06
SPG11	14.5521475	-2.010159664	0.386261931	-5.204136111	1.94901E-07	1.15883E-06
C5H19orf44	23.78995015	1.658888436	0.31877435	5.203958327	1.95088E-07	1.15942E-06
NDRG2	125.6512958	-2.450172564	0.470854734	-5.203669813	1.95391E-07	1.16071E-06
PREX2	4.13037416	5.398077107	1.037579237	5.202568551	1.96553E-07	1.16678E-06
SNX10	5.535121092	5.213964426	1.002196677	5.202536135	1.96587E-07	1.16678E-06
HNRNPC	186.2100654	0.756870949	0.145488628	5.202268789	1.9687E-07	1.16794E-06
LRRN4CL	24.41554154	-1.476014482	0.283775457	-5.201346502	1.9785E-07	1.17323E-06
DPYSL3	7.473662674	3.62852836	0.698307827	5.196173121	2.03433E-07	1.2058E-06
CWC25	78.4864308	1.081434101	0.208124897	5.196082328	2.03532E-07	1.20586E-06
LOC132660224	21.17902532	1.674449096	0.322273215	5.195743909	2.03903E-07	1.20752E-06
GABRE	9.083415957	-3.191342979	0.614280517	-5.19525346	2.04441E-07	1.20963E-06
NAPA	21.97785824	1.964785818	0.378186377	5.195284495	2.04407E-07	1.20963E-06
FLNB	150.146761	-1.481590882	0.285192275	-5.195059657	2.04654E-07	1.21036E-06
GGCX	29.20836301	1.512344669	0.291155155	5.194291229	2.05501E-07	1.21483E-06
CORO1A	5.714048806	5.244957584	1.009893987	5.193572445	2.06297E-07	1.21899E-06
RPL35	174.8006345	-0.883945357	0.170215539	-5.193094368	2.06827E-07	1.22159E-06
WRAP73	11.94282492	-2.474563192	0.476520031	-5.192988817	2.06945E-07	1.22174E-06
LOC105615264	4.087194233	5.365938672	1.033811352	5.190442782	2.09795E-07	1.23802E-06
ITGA5	7.282302922	4.524382513	0.871693022	5.190339259	2.09911E-07	1.23816E-06
CCND1	673.9467531	-0.691589589	0.133283138	-5.188875341	2.11568E-07	1.24738E-06
LOC101121770	9.355420516	3.439877524	0.66302019	5.188194231	2.12343E-07	1.2514E-06
LOC132658765	21.25491472	1.68975067	0.325730353	5.187575099	2.1305E-07	1.25501E-06
LIX1L	4.511789969	5.515343161	1.06325422	5.187229033	2.13446E-07	1.25679E-06
PLEKHG3	15.5861638	-2.552083077	0.492049727	-5.186636511	2.14126E-07	1.26024E-06
TNFSF13B	3.995624944	5.356800216	1.033300973	5.184162559	2.16988E-07	1.27652E-06
DNM1	4.025930943	5.353303003	1.032767128	5.183456033	2.17812E-07	1.2808E-06
TUFT1	34.19828497	-1.431339041	0.276190735	-5.182429605	2.19014E-07	1.28731E-06
CD27	4.016162086	5.349425084	1.032349801	5.181795047	2.19761E-07	1.29113E-06
MRPL13	37.57915878	-1.221149774	0.235719621	-5.180518143	2.2127E-07	1.29943E-06
LOC101117107	4.074262208	5.385175277	1.039624693	5.17992244	2.21978E-07	1.30301E-06
NUP133	30.50891987	-1.487026202	0.287079585	-5.179839594	2.22077E-07	1.30302E-06
PXN	19.66903582	-1.922894997	0.371251156	-5.179499009	2.22483E-07	1.30483E-06
GCSH	10.79265314	-2.446322623	0.472441288	-5.178045789	2.24222E-07	1.31445E-06

USO1	69.00782926	1.085348685	0.209631927	5.177401644	2.24997E-07	1.31842E-06
WIPI2	39.50474776	1.348912827	0.260630877	5.175568003	2.27219E-07	1.33085E-06
ARHGEF15	4.04941487	5.381688879	1.040024431	5.174579286	2.28425E-07	1.33733E-06
TET2	44.62801102	1.151037476	0.222474206	5.17380194	2.29378E-07	1.34232E-06
MMRN2	4.287183008	5.41362531	1.046888444	5.171157767	2.32648E-07	1.36086E-06
LOC101115435	8.365066196	3.789816782	0.733310983	5.168089485	2.36499E-07	1.38279E-06
PCDH18	4.005290562	5.335263345	1.032595604	5.166846853	2.38076E-07	1.3914E-06
SPTSSB	9.99852257	-2.694152809	0.521560024	-5.165566159	2.39712E-07	1.40035E-06
LOC114117060	8.915557372	4.153829374	0.804339988	5.164270628	2.41378E-07	1.40946E-06
LOC101109369	8.158329228	3.942509939	0.763522669	5.16357942	2.42272E-07	1.41406E-06
RBBP6	358.6212996	0.800729485	0.155076124	5.163460779	2.42425E-07	1.41434E-06
CHEK1	41.88590158	-1.329045886	0.257418875	-5.162969836	2.43062E-07	1.41744E-06
GLUL	84.90524592	-1.3534972	0.262218506	-5.161715012	2.44698E-07	1.42635E-06
RABEP1	115.9611028	0.900921729	0.174595078	5.160063723	2.46866E-07	1.43836E-06
KLRK1	4.173029883	5.416455333	1.049737221	5.159820215	2.47187E-07	1.43961E-06
SMIM14	62.46306262	1.315485818	0.254980078	5.159170974	2.48046E-07	1.44398E-06
ELF3	260.1563982	0.775161379	0.150254684	5.158983116	2.48295E-07	1.4448E-06
HYI	24.47730097	-1.435473069	0.278289399	-5.158202478	2.49332E-07	1.4502E-06
FMOD	4.543712031	5.495163918	1.065412344	5.157781352	2.49893E-07	1.45221E-06
VDAC2	54.30662523	-1.147597873	0.222495964	-5.157836808	2.49819E-07	1.45221E-06
CHP1	158.9998526	-0.971910199	0.188487962	-5.156351568	2.51808E-07	1.4627E-06
TAB1	16.29206193	-1.831206883	0.355162087	-5.155975122	2.52314E-07	1.465E-06
STK38	59.30518258	1.159918692	0.225013397	5.154887254	2.53784E-07	1.47225E-06
TFE3	66.85107437	1.073974188	0.208337937	5.154962202	2.53682E-07	1.47225E-06
THAP4	24.616145	-1.44709448	0.280756059	-5.154276944	2.54611E-07	1.47641E-06
GET4	95.01223593	-1.149202299	0.222989916	-5.153606587	2.55524E-07	1.48106E-06
SENP2	82.34907629	1.020030853	0.197986958	5.15201033	2.57709E-07	1.49308E-06
ANKRD22	31.21994847	-1.465193162	0.284400944	-5.151857592	2.57919E-07	1.49365E-06
TCAF2	17.31871154	2.31363696	0.449164078	5.150983961	2.59123E-07	1.49998E-06
ARHGEF40	22.40764376	-1.616790029	0.313906787	-5.150541798	2.59735E-07	1.50287E-06
LOC101116972	4.221247111	5.418451917	1.052272268	5.149287004	2.61479E-07	1.5123E-06
LOC114118110	7.284881124	-3.600604905	0.699361591	-5.148416712	2.62694E-07	1.51867E-06
KIF1B	52.73432734	1.119505609	0.217461311	5.148067973	2.63183E-07	1.52084E-06
ATP6V1C2	7.712997735	4.692977441	0.911651525	5.147775561	2.63594E-07	1.52255E-06
LOC100527962	34.28710877	-1.364065781	0.26504298	-5.14658333	2.65274E-07	1.5316E-06
ACBD3	164.7733211	0.909893265	0.176822926	5.14578785	2.664E-07	1.53744E-06
FANCD2	17.82071188	-1.824437974	0.35456179	-5.145613621	2.66648E-07	1.5382E-06
EDN3	7.780631135	4.615811221	0.897082265	5.145360022	2.67008E-07	1.53962E-06
PCMTD1	49.39499928	1.139190683	0.221424659	5.14482302	2.67773E-07	1.54336E-06
CDH6	3.920694337	5.329433698	1.035929188	5.144592663	2.68102E-07	1.54459E-06
NR1H4	8.055756088	3.678368845	0.71507249	5.144050287	2.68878E-07	1.54839E-06
SLC27A5	9.354990592	3.046790169	0.592526976	5.142027776	2.71789E-07	1.56448E-06

NAP1L4	94.73952182	-1.742774115	0.338936377	-5.141891614	2.71986E-07	1.56494E-06
SURF4	126.9512289	0.768786247	0.149703442	5.135394599	2.81553E-07	1.61929E-06
HNF4A	67.40352962	1.017867392	0.198313031	5.132629908	2.85721E-07	1.64256E-06
KIAA0825	38.86071097	-1.269303105	0.247453024	-5.129470988	2.90558E-07	1.66964E-06
RBM38	15.19798446	-2.827477967	0.551342169	-5.128354271	2.92286E-07	1.67885E-06
DCAF4	22.39208034	-1.540012314	0.300373477	-5.126991671	2.94409E-07	1.69032E-06
SLC16A7	50.60946642	1.178066288	0.229792603	5.126650163	2.94943E-07	1.69266E-06
MED22	30.89596407	-1.827485764	0.356477177	-5.126515474	2.95154E-07	1.69314E-06
DZIP1	96.05366155	-1.062302985	0.207284804	-5.124847385	2.97779E-07	1.70747E-06
SLC5A9	11.47507459	2.601982513	0.507789543	5.124135673	2.98906E-07	1.7132E-06
VRK3	28.71745377	1.375021775	0.26837753	5.123460879	2.99978E-07	1.7186E-06
LOC101115765_1	10.6779215	-2.307228385	0.450366366	-5.123003317	3.00707E-07	1.72204E-06
SLC15A1	6.972423739	4.982380796	0.972611421	5.122683826	3.01217E-07	1.72423E-06
IMMP2L	315.0032238	0.956128234	0.18666502	5.12216072	3.02054E-07	1.72828E-06
MRPL54	29.65081817	1.694445756	0.330871307	5.121162576	3.03658E-07	1.73671E-06
MMP12	7.011799116	-4.702319601	0.918281921	-5.120779896	3.04275E-07	1.73949E-06
HSPA9	192.1594084	-0.686855436	0.134155401	-5.119849284	3.0578E-07	1.74735E-06
PRUNE1	22.76331607	-1.586527791	0.310119548	-5.115858706	3.12317E-07	1.78394E-06
EIF4E3	5.93014948	5.284520803	1.033042833	5.115490503	3.12927E-07	1.78666E-06
SVEP1	4.020285842	5.327540839	1.041803238	5.113768748	3.15794E-07	1.80226E-06
BCL2L13	31.36986005	-1.742266979	0.340735285	-5.113256702	3.16652E-07	1.80639E-06
TP53	14.30542739	-2.007098388	0.392544746	-5.113043568	3.17009E-07	1.80766E-06
FBXW2	102.6895156	-0.99820344	0.195254731	-5.112313725	3.18237E-07	1.81388E-06
RPL27A	1302.278262	-0.707881267	0.138567343	-5.108572133	3.24602E-07	1.84938E-06
LOC101104162	74.45532506	-1.112924782	0.217857905	-5.108489319	3.24745E-07	1.8494E-06
KCNB2	7.401517607	-3.242322415	0.634737891	-5.108128034	3.25366E-07	1.85215E-06
NDUFA4L2	6.97733937	-5.127696995	1.004478826	-5.104833333	3.31086E-07	1.88391E-06
CAPN8	5.233113969	5.123931603	1.004088065	5.103069922	3.34187E-07	1.90074E-06
ENG	6.568469756	4.880557354	0.956742126	5.101225523	3.37461E-07	1.91855E-06
CHD3	175.4697423	1.302670702	0.255373795	5.101035136	3.37801E-07	1.91966E-06
LOC114113058	16.49202546	-2.11015091	0.413695909	-5.100729459	3.38347E-07	1.92195E-06
CYFIP1	31.45292879	1.341543549	0.263028752	5.100368459	3.38993E-07	1.9248E-06
SMAGP	22.0161021	-1.906468529	0.373870302	-5.099277796	3.40952E-07	1.9351E-06
SECISBP2L	89.04374136	0.896111723	0.175906262	5.0942571	3.50111E-07	1.98624E-06
CHD1	259.0398377	0.871260885	0.171031417	5.094156961	3.50296E-07	1.98645E-06
ACKR4	4.198228861	5.430368413	1.066141159	5.093479758	3.51551E-07	1.99272E-06
TNFRSF21	20.73178776	-1.735942967	0.340922925	-5.091892746	3.54507E-07	2.00862E-06
CSPP1	163.8555138	1.142091059	0.224362219	5.090389392	3.57329E-07	2.02375E-06
PRELID3A	48.95107722	-1.127770747	0.221688582	-5.087184638	3.63418E-07	2.05737E-06
SEC13	52.29000664	-1.124487558	0.221164915	-5.084384908	3.68819E-07	2.08706E-06
CCNJ	14.29428729	-2.277505829	0.448173329	-5.081752259	3.73969E-07	2.11531E-06
P2RX1	4.154475835	5.384569848	1.059736015	5.081048275	3.75358E-07	2.12226E-06

LOC101111911	59.147377	-1.610334409	0.316936892	-5.080930786	3.7559E-07	2.12268E-06
HMGCR	146.4095387	0.951064883	0.187230733	5.07964087	3.78149E-07	2.13624E-06
C12H1orf115	9.095985515	2.81108349	0.553474169	5.078978654	3.79469E-07	2.14279E-06
LOC101112822	4.521482587	5.542231498	1.091241019	5.078833549	3.79759E-07	2.14352E-06
KCTD14	19.38096001	-1.725324788	0.339717212	-5.078708788	3.80009E-07	2.14403E-06
CCDC69	5.839980556	5.261783425	1.036083903	5.078530234	3.80366E-07	2.14514E-06
SCMH1	57.49029769	1.195607232	0.235444835	5.078077969	3.81272E-07	2.14934E-06
ZNF660	24.59371751	1.877965068	0.369844392	5.077716763	3.81998E-07	2.15252E-06
EMP2	9.390929286	4.11045705	0.809589872	5.077209084	3.83019E-07	2.15682E-06
SOAT1	33.45623736	-1.292671299	0.254604318	-5.077177443	3.83083E-07	2.15682E-06
IQGAP2	246.1310327	0.637639375	0.125600235	5.076737112	3.83972E-07	2.16091E-06
NFATC4	5.100181712	5.09419103	1.003627946	5.075776389	3.85917E-07	2.17095E-06
SSX2IP	22.15029265	1.599546545	0.315161507	5.075323319	3.86838E-07	2.17521E-06
HECTD2	7.940702154	3.455473503	0.680849126	5.075241154	3.87005E-07	2.17523E-06
FAM13C	4.056031287	5.361855643	1.056646452	5.074408412	3.88703E-07	2.18386E-06
NUP58	13.60633102	2.203520482	0.434278346	5.073981931	3.89576E-07	2.18693E-06
TBX2	3.781150034	5.271492569	1.03892477	5.073988724	3.89562E-07	2.18693E-06
LOC105605322	16.43310736	-2.340137301	0.461274106	-5.07320327	3.91174E-07	2.19498E-06
LDLR	297.0968668	-1.123947805	0.221635155	-5.07116213	3.95394E-07	2.21772E-06
NUGGC	7.95790795	3.484322371	0.687320593	5.06942816	3.99013E-07	2.23708E-06
ZBTB11	32.17704681	1.258718374	0.248334767	5.068635338	4.00678E-07	2.24548E-06
UBN2	73.89527496	0.937537897	0.185036483	5.066773217	4.04616E-07	2.26659E-06
CAP1	110.3179067	1.035246341	0.2043475	5.066107203	4.06033E-07	2.27358E-06
CHST1	5.750759512	5.222639307	1.030959584	5.06580412	4.0668E-07	2.27625E-06
OLFML1	3.749264919	5.262960479	1.039156198	5.064648117	4.09155E-07	2.28915E-06
HCST	4.056737242	5.336594843	1.053894376	5.063690407	4.11217E-07	2.29972E-06
GXYLT1	90.3531281	-0.918299057	0.181397874	-5.062347418	4.14125E-07	2.31502E-06
CIST1	155.5400055	-0.996755224	0.196933959	-5.061367933	4.16259E-07	2.32597E-06
LOC105605766	5.18573252	5.134834158	1.014801613	5.0599389	4.19391E-07	2.34249E-06
LOC132660282	21.66014816	1.761802385	0.348193845	5.059832076	4.19626E-07	2.34282E-06
RNH1	28.42110947	-1.395610177	0.275830231	-5.059670852	4.19981E-07	2.34383E-06
LOC114112702	64.00082902	2.541489581	0.502342083	5.059280648	4.20841E-07	2.34765E-06
AIG1	15.10066497	2.037793497	0.4028275	5.058724887	4.22069E-07	2.35352E-06
ATP5PO	324.1804371	0.699608642	0.138312991	5.058155694	4.23331E-07	2.3587E-06
BICRA	15.4380833	2.062090903	0.407677188	5.058146411	4.23351E-07	2.3587E-06
ESRP2	29.50862638	-1.404505965	0.277718164	-5.057306815	4.25219E-07	2.36811E-06
ANKRD1	3.374741048	-5.379948523	1.063858015	-5.057017429	4.25864E-07	2.37072E-06
MITF	7.605155584	3.228206925	0.638493626	5.055973617	4.28201E-07	2.38174E-06
TACC1	28.38752788	1.50979345	0.298613263	5.056016038	4.28105E-07	2.38174E-06
STAT2	36.00413824	1.247767957	0.246941144	5.052896156	4.35161E-07	2.41945E-06
CHRM2	3.86539269	5.279699471	1.045151323	5.051612485	4.38096E-07	2.43476E-06
SIAH2	70.42484839	1.064343367	0.210705529	5.051330973	4.38742E-07	2.43733E-06

LOC101111607	8.531443102	3.44648054	0.682414485	5.05042114	4.40837E-07	2.44796E-06
ELOVL6	140.9555083	0.853733512	0.169127709	5.04786304	4.46779E-07	2.47952E-06
ENAH	184.9565822	0.931341716	0.184503951	5.047814472	4.46893E-07	2.47952E-06
RASSF2	3.928608703	5.320313522	1.054225781	5.046654728	4.49613E-07	2.49358E-06
RND1	9.712862617	-2.707806351	0.53675181	-5.044801531	4.53992E-07	2.51682E-06
IDH2	56.76127557	-1.10187311	0.218443295	-5.044206594	4.55407E-07	2.52362E-06
SENP7	61.19085948	1.159599479	0.229919365	5.043505049	4.57081E-07	2.53184E-06
GINM1	31.46658563	1.445297111	0.28661945	5.042564664	4.59333E-07	2.54327E-06
NBN	39.29279122	1.339846927	0.265732435	5.042090277	4.60474E-07	2.54849E-06
PRR12	12.40070746	2.142269735	0.424883762	5.042013661	4.60658E-07	2.54849E-06
SAP30	14.69960066	-1.831082486	0.363183737	-5.041752419	4.61288E-07	2.55092E-06
CD53	7.947571338	4.623747689	0.917123966	5.041573288	4.6172E-07	2.55225E-06
CDKN2AIP	32.93142305	1.241644251	0.246291985	5.041350623	4.62258E-07	2.55417E-06
TSC1	91.40345382	-0.838678195	0.166375825	-5.040865727	4.63431E-07	2.55959E-06
LOC101108690	28.86837578	1.433127346	0.284389198	5.039317093	4.67196E-07	2.57932E-06
LOC121820289	26.71364255	-2.13917383	0.424588793	-5.038224907	4.69869E-07	2.59301E-06
RASSF7	15.90772202	-1.792017328	0.35570692	-5.037904031	4.70657E-07	2.59629E-06
CENPK	94.10924135	-1.161757071	0.230635711	-5.037195088	4.72403E-07	2.60484E-06
MSR1	3.99249447	5.333597605	1.059066875	5.036129192	4.7504E-07	2.6183E-06
PALB2	41.82201356	-1.233850287	0.245129084	-5.033471616	4.81676E-07	2.65379E-06
KCNJ6	8.022504421	-2.871156963	0.570529213	-5.032445137	4.84263E-07	2.66694E-06
MTLN	9.584557419	-2.566097576	0.510157629	-5.030009219	4.90456E-07	2.69993E-06
ZNRF1	64.23542801	1.011003797	0.201099463	5.027381878	4.97222E-07	2.73605E-06
LOC114109639	12.95536193	-2.014688784	0.401068914	-5.023298273	5.07916E-07	2.79375E-06
LOC114116901	7.855973721	-2.863282968	0.570174149	-5.021769177	5.11977E-07	2.81493E-06
GTDC1	26.53588184	1.461703349	0.291195159	5.019669122	5.17606E-07	2.8447E-06
LOC114108618	23.84057768	-1.534085375	0.305660606	-5.018917542	5.19634E-07	2.85468E-06
ARHGAP11A	85.23601811	-1.011153606	0.201479845	-5.01863403	5.20402E-07	2.85772E-06
LUC7L2	218.0389579	0.990120681	0.197420246	5.015294543	5.29523E-07	2.90661E-06
LOC101108390	86.43979722	-1.181680905	0.235760919	-5.012200114	5.38112E-07	2.95255E-06
ZFC3H1	131.4891705	0.972001437	0.193964433	5.011235425	5.40817E-07	2.96617E-06
LOC101104574	98.91137642	0.826463913	0.16492935	5.011017829	5.41429E-07	2.96831E-06
COMT	265.8387507	-0.909697493	0.181556305	-5.010553023	5.42738E-07	2.97305E-06
WDR82	72.09122463	0.99906837	0.199389914	5.010626413	5.42531E-07	2.97305E-06
IL1R1	7.638837283	3.878569974	0.774215907	5.009674872	5.45221E-07	2.98542E-06
HSPB6	4.349626775	5.427499246	1.084291972	5.005569889	5.5697E-07	3.04851E-06
MED13L	124.1094847	0.865081705	0.17283238	5.005321949	5.57687E-07	3.05073E-06
SF3A2	55.59855913	-1.075364975	0.214846472	-5.005271734	5.57833E-07	3.05073E-06
LOC132658125	31.74013936	1.51008717	0.301711083	5.005076897	5.58397E-07	3.05257E-06
LOC132657955	19.96518641	1.558202661	0.31144039	5.003213164	5.63825E-07	3.08099E-06
EXOSC10	35.45849062	-1.640207556	0.327892049	-5.002279138	5.66565E-07	3.09469E-06
TRPS1	18.19906509	1.898737242	0.37960008	5.001941099	5.67559E-07	3.09886E-06

GTF2IRD1	15.58136052	-2.044744851	0.408822747	-5.001543734	5.68731E-07	3.10398E-06
SERTM1	3.655064283	5.228298249	1.045533307	5.000604204	5.71509E-07	3.11787E-06
GULP1	60.96290565	0.981921861	0.19637116	5.000336401	5.72304E-07	3.12093E-06
PCSK5	44.26820666	1.201321485	0.240271187	4.999856618	5.7373E-07	3.12743E-06
PIH1D1	34.0283761	1.316643734	0.263467276	4.997371028	5.81172E-07	3.16671E-06
PI16	5.146128919	5.119270473	1.025078606	4.99402723	5.91331E-07	3.22075E-06
KCNJ8	3.829545584	5.284273961	1.058189513	4.993693373	5.92354E-07	3.22501E-06
ACTN4	48.51802452	-1.125360436	0.225428729	-4.992089712	5.97295E-07	3.24927E-06
ERG	3.651276355	5.199118736	1.041467084	4.992110471	5.97231E-07	3.24927E-06
AHSA1	136.3772078	-1.056907494	0.211720113	-4.992003264	5.97562E-07	3.2494E-06
CHRNB1	8.12676907	-2.74157806	0.549203137	-4.991919882	5.97821E-07	3.24948E-06
ASCC3	25.35817406	1.514406198	0.30342147	4.99109769	6.00371E-07	3.26202E-06
AKAP5	8.141548981	2.888124293	0.578900209	4.988984714	6.06974E-07	3.29655E-06
PPT1	78.65267582	-0.841497043	0.168750956	-4.986620894	6.14445E-07	3.33577E-06
SGSM1	20.65277181	-1.755765417	0.352156089	-4.985759073	6.1719E-07	3.34932E-06
KCNAB1	3.753776516	5.253880156	1.054279734	4.983383429	6.2482E-07	3.38934E-06
IFI35	90.28736619	0.947556932	0.190151637	4.983164741	6.25526E-07	3.39123E-06
SKIC8	12.2043595	-2.078230596	0.417054215	-4.983118554	6.25676E-07	3.39123E-06
RNF157	19.55300615	-1.720543518	0.345291857	-4.982867345	6.26489E-07	3.39427E-06
SLC22A23	149.9967316	0.798924635	0.160364521	4.981928852	6.29536E-07	3.40939E-06
HNRNPUL2	578.8034128	-0.884059697	0.177458374	-4.981786297	6.3E-07	3.41052E-06
SLC25A13	38.01371007	-1.186838811	0.238262033	-4.981233457	6.31803E-07	3.4189E-06
GGH	14.18462883	-2.129830709	0.427579976	-4.981128277	6.32146E-07	3.41937E-06
MSL3	57.02784433	1.067129914	0.214246097	4.980860457	6.33022E-07	3.42272E-06
LOC114116588	79.96606022	1.142025092	0.229310454	4.980257434	6.34997E-07	3.43202E-06
TRAPPC4	32.42021927	1.26068675	0.253144999	4.980097385	6.35523E-07	3.43347E-06
TMEM125	22.07018513	1.567219553	0.314711136	4.979866857	6.3628E-07	3.43617E-06
SH3BGRL	75.58436337	1.073878757	0.215683245	4.978962349	6.39261E-07	3.45087E-06
CHODL	3.824150455	5.267771338	1.058088382	4.978574028	6.40544E-07	3.45641E-06
KLF6	282.2850115	1.039083598	0.208718126	4.978406127	6.411E-07	3.45801E-06
SUCO	80.91537771	1.089742295	0.218905929	4.978130569	6.42013E-07	3.46154E-06
DNAJC15	23.74317298	-1.443790125	0.290064982	-4.977471309	6.44203E-07	3.47194E-06
LOC132657598	54.20762489	-1.158075379	0.232717844	-4.976306749	6.48089E-07	3.49148E-06
PRUNE2	7.028067761	4.974526515	0.999667168	4.976182748	6.48504E-07	3.49231E-06
LOC101108339	87.72967163	-2.343220442	0.470915979	-4.975877963	6.49526E-07	3.4964E-06
CD24	438.4357333	0.782505908	0.157266982	4.975652861	6.50281E-07	3.49906E-06
ARMCX1	3.595518725	5.187016667	1.042750034	4.974362502	6.54627E-07	3.52096E-06
TACSTD2	7.546495574	-4.772463768	0.959426466	-4.974288219	6.54878E-07	3.52096E-06
LOC101107489	3.585393617	5.19839116	1.045375835	4.972748545	6.60103E-07	3.54762E-06
CADM4	7.47539442	-3.480735885	0.699996767	-4.972502799	6.6094E-07	3.55069E-06
PGGHG	11.14675281	2.506332758	0.504049917	4.972389986	6.61325E-07	3.55133E-06
NSDHL	55.06728586	1.106064443	0.222484794	4.971415905	6.64657E-07	3.56779E-06

RPL21	236.4077914	-1.37244229	0.276086174	-4.971064897	6.65862E-07	3.57282E-06
LOC101114456	5.159205749	5.105616847	1.027334721	4.969769583	6.70325E-07	3.59533E-06
SHPRH	36.04746901	1.296509646	0.260962529	4.968183166	6.75831E-07	3.62341E-06
LOC101105214	46.00990052	1.45590894	0.293111132	4.967088526	6.79656E-07	3.64245E-06
SLC2A4RG	20.08382563	-1.627130243	0.327606854	-4.966716126	6.80962E-07	3.64799E-06
LOC101122689	3.577337129	5.194460318	1.045903162	4.966483043	6.8178E-07	3.65039E-06
PYCR1	36.43636867	-1.661537935	0.334553568	-4.966433164	6.81955E-07	3.65039E-06
WSB1	58.52756268	-1.242540245	0.250215434	-4.965881709	6.83896E-07	3.65931E-06
RUNX1T1	20.92723608	1.684942814	0.339332399	4.965464015	6.8537E-07	3.66573E-06
BGN	5.036831041	5.040112219	1.015197846	4.96466008	6.88215E-07	3.67947E-06
GNA12	39.45889876	-1.129686111	0.227697968	-4.961335941	7.001E-07	3.74152E-06
PSTPIP1	3.881001135	5.305652948	1.069726389	4.959822438	7.05576E-07	3.76928E-06
EDEM3	46.60217572	1.168006174	0.235511445	4.95944548	7.06947E-07	3.77509E-06
GFRA1	4.886876506	5.054700354	1.01925056	4.959232353	7.07723E-07	3.77772E-06
DNAJC5	55.11407757	-1.334756542	0.269275687	-4.956840172	7.16489E-07	3.82146E-06
PLEKHH1	48.56244011	1.317270225	0.26574582	4.956880316	7.16341E-07	3.82146E-06
COPB2	99.75352323	0.783565188	0.158082576	4.956682824	7.17069E-07	3.82303E-06
LOC101120455	17.2825644	-1.690229354	0.341069894	-4.955668566	7.2082E-07	3.8415E-06
SLC39A13	30.30871009	-1.249023082	0.252103768	-4.954400696	7.25536E-07	3.86509E-06
LOC105605823	7.381993262	3.846552761	0.776784929	4.95188902	7.34965E-07	3.91376E-06
LOC132659918	7.001243284	3.700852898	0.747378026	4.951781787	7.3537E-07	3.91436E-06
TMTC4	27.39448032	-1.368632281	0.276400306	-4.951630851	7.35941E-07	3.91584E-06
APOC3	5.237519271	5.711188185	1.153471605	4.951303664	7.3718E-07	3.92087E-06
RPL22L1	92.55390103	-1.040264634	0.210124625	-4.950703095	7.39458E-07	3.93142E-06
RPS10	1358.518199	-0.775141329	0.156643516	-4.948441836	7.48099E-07	3.97578E-06
MICU1	30.2547286	1.745129271	0.352684687	4.948128842	7.49303E-07	3.9806E-06
OLA1	34.25648211	-1.294149146	0.261621084	-4.946654624	7.54998E-07	4.00926E-06
SLCO2B1	3.796429558	5.285698854	1.069006258	4.94449758	7.63405E-07	4.05229E-06
ACOX1	80.42764172	1.115356022	0.225750344	4.940661452	7.7858E-07	4.12956E-06
RAMP2	11.00641849	2.413216616	0.488433664	4.940725416	7.78325E-07	4.12956E-06
ATP6V0E1	45.60206286	1.208354827	0.244604094	4.940043351	7.81052E-07	4.14103E-06
MOB3B	41.47778472	-1.130838993	0.228929082	-4.93969129	7.82463E-07	4.14687E-06
FERMT3	3.715297309	5.232085602	1.059602338	4.937782238	7.9016E-07	4.186E-06
ATP6V1H	23.27456977	1.440015691	0.291676791	4.937025281	7.93232E-07	4.20061E-06
DUSP16	49.22775028	1.234580105	0.250083526	4.936671051	7.94673E-07	4.20658E-06
SYPL1	96.9022572	-1.11577845	0.226097225	-4.934949786	8.01714E-07	4.24216E-06
APAF1	66.78699423	0.971235504	0.196945553	4.931492425	8.16037E-07	4.31625E-06
TNRC6C	48.3269758	1.385892915	0.281120426	4.92989049	8.22757E-07	4.35007E-06
CENPW	25.46559877	-1.49643485	0.303573282	-4.929402352	8.24815E-07	4.35923E-06
SSRP1	432.243646	-0.888443194	0.180250591	-4.928933586	8.26797E-07	4.36797E-06
MYOC	3.582758834	5.183506211	1.051855507	4.927964133	8.30909E-07	4.38796E-06
SLC9A9	3.498555061	5.161149408	1.047418436	4.927495287	8.32904E-07	4.39503E-06

XPA	79.66478719	1.241012054	0.251851469	4.927555347	8.32648E-07	4.39503E-06
NDUFV3	65.05960412	-1.005197222	0.204106175	-4.924874145	8.44147E-07	4.45259E-06
GRAP2	3.605092299	5.188512315	1.053588677	4.924609032	8.45292E-07	4.45687E-06
CPED1	3.462070141	5.139427142	1.04382098	4.92366722	8.49372E-07	4.47662E-06
SMAD5	130.9094787	1.054211036	0.214154584	4.922663874	8.5374E-07	4.49717E-06
UBE2S	127.0361494	-0.981532841	0.199392473	-4.922617311	8.53943E-07	4.49717E-06
PRMT3	42.90143422	-1.092307025	0.222037389	-4.919473375	8.67774E-07	4.5682E-06
CTPS1	29.3467735	-1.461483886	0.297174196	-4.917936712	8.74612E-07	4.60239E-06
PARM1	6.711647891	4.447248362	0.904486353	4.916877237	8.79357E-07	4.62553E-06
VASH1	3.489092772	5.145486497	1.046606514	4.916352446	8.81716E-07	4.63612E-06
ZHX1	30.94801371	1.754859808	0.357106829	4.914103193	8.91898E-07	4.68781E-06
BRCA1	12.29420896	-1.965792843	0.40004447	-4.913935803	8.9266E-07	4.68997E-06
B4GALNT4	9.330363824	-2.461713356	0.501253242	-4.911117079	9.0559E-07	4.7552E-06
LIPH	22.46440397	-1.538059712	0.313181897	-4.911074776	9.05785E-07	4.7552E-06
TMPRSS15	3.882426576	5.295542732	1.078360996	4.910732818	9.07367E-07	4.76163E-06
MDH2	160.0444566	-0.716498483	0.145931425	-4.909829979	9.11554E-07	4.78172E-06
TES	127.3787321	0.747421748	0.152266531	4.908641056	9.17097E-07	4.80891E-06
VPS13C	165.05199	0.678184443	0.138165724	4.908485428	9.17824E-07	4.81084E-06
AHSG	4.192818932	-5.111261704	1.041694477	-4.906680237	9.26309E-07	4.85341E-06
NLRP6	61.59265727	1.232054667	0.251109761	4.906438768	9.27449E-07	4.85748E-06
NKD2	9.621479007	-2.3842723	0.48615495	-4.904346447	9.37389E-07	4.90762E-06
GPRC5C	7.927439379	2.915632566	0.594533493	4.904067807	9.38721E-07	4.91266E-06
SGSH	21.3428291	-1.593354583	0.324962955	-4.903188382	9.42935E-07	4.93279E-06
PTPRS	4.66678351	4.962586561	1.012233219	4.902611838	9.45707E-07	4.94535E-06
TAP2	94.72829317	0.814842176	0.166210044	4.902484572	9.4632E-07	4.94662E-06
MVD	141.5652098	1.073286598	0.218947537	4.902026354	9.48531E-07	4.95624E-06
ITSN1	95.29023866	1.041674885	0.212630046	4.899001374	9.6325E-07	5.03118E-06
RGS19	6.210361371	4.273337499	0.872381159	4.898475232	9.65832E-07	5.0427E-06
COPS6	45.77119995	-1.040641238	0.212460662	-4.898041953	9.67964E-07	5.05186E-06
SLIRP	43.27299732	-1.423315348	0.290688214	-4.896364149	9.76261E-07	5.09317E-06
STRAP	65.45602949	-1.125784084	0.229941099	-4.89596722	9.78234E-07	5.10147E-06
PFN2	7.1053252	3.283698069	0.671269238	4.891774986	9.99307E-07	5.20933E-06
NIN	69.69161812	1.016840484	0.207983973	4.889032882	1.01333E-06	5.28036E-06
PGP	26.07454354	-1.711173414	0.350047621	-4.888401789	1.01658E-06	5.29318E-06
PHIP	238.3775134	0.755784675	0.154607373	4.888412888	1.01652E-06	5.29318E-06
LOC121819502	3.639151953	5.197469657	1.063582151	4.886759009	1.02509E-06	5.33544E-06
RBM18	31.30556863	-1.330313989	0.272362412	-4.884352358	1.03769E-06	5.39892E-06
ADAM23	3.475241768	5.143181308	1.053130365	4.883708116	1.04109E-06	5.41449E-06
POLR2A	94.65722043	-1.043291768	0.213694014	-4.882175917	1.04922E-06	5.45462E-06
CDH13	3.874824142	5.273731168	1.080361417	4.881450859	1.05308E-06	5.47259E-06
RALY	122.3317954	-0.912437037	0.187040959	-4.87827394	1.07018E-06	5.55929E-06
ARHGEF6	4.842073573	5.020014998	1.029162008	4.877769447	1.07292E-06	5.57136E-06

COPZ2	3.431268453	5.130337096	1.051874695	4.877327235	1.07533E-06	5.58169E-06
PBDC1	110.8062541	1.061202724	0.217619605	4.8764114	1.08033E-06	5.6033E-06
SMAD4	139.6788561	1.148521886	0.235525437	4.876423977	1.08026E-06	5.6033E-06
PRMT2	60.38069922	1.177458263	0.241515891	4.875282771	1.08653E-06	5.63325E-06
CKAP2L	42.60282947	-1.199914426	0.24624048	-4.872937335	1.09951E-06	5.69836E-06
LOC114110037	4.863904089	5.003245019	1.026767107	4.87281389	1.1002E-06	5.69971E-06
SNCAIP	3.436141307	5.116753527	1.050295672	4.871726756	1.10627E-06	5.72895E-06
ATXN7L3	31.64040265	-1.676647895	0.3441895	-4.871292973	1.1087E-06	5.7371E-06
NME3	17.08781295	-1.77826494	0.365049804	-4.871294054	1.1087E-06	5.7371E-06
CHPT1	89.03154329	1.057754011	0.217156055	4.870939528	1.11069E-06	5.74515E-06
C3	36.73186589	1.222229214	0.251007358	4.869296349	1.11996E-06	5.7879E-06
FGF7	3.53313849	5.15882065	1.059470251	4.869245402	1.12025E-06	5.7879E-06
PSIP1	58.4698351	-1.014949072	0.208436496	-4.869344346	1.11969E-06	5.7879E-06
PDGFRL	3.676928633	5.194775317	1.067120518	4.86803058	1.12716E-06	5.82133E-06
CTXND1	15.245797	6.121230399	1.258462709	4.86405386	1.15006E-06	5.93729E-06
LOC105613374	3.664305858	5.192630045	1.067627196	4.863710914	1.15205E-06	5.9453E-06
LOC101114226	5.531098041	4.63938031	0.954159723	4.862268025	1.16048E-06	5.9865E-06
IL2RB	3.379687339	5.116810715	1.052477016	4.861684043	1.16391E-06	6.00188E-06
LOC132658540	16.46178935	1.791544778	0.36854033	4.861190575	1.16682E-06	6.01454E-06
B3GNT5	34.60882085	1.213194391	0.249586568	4.860816023	1.16903E-06	6.02361E-06
CTDP1	11.26957489	-2.161515988	0.444749919	-4.860070564	1.17344E-06	6.044E-06
PAX9	3.485803677	-5.370068361	1.104997464	-4.859801525	1.17503E-06	6.04989E-06
LOC101111694	6.41382346	4.344814849	0.894228141	4.858731961	1.1814E-06	6.08032E-06
SNAP23	56.91485228	0.953022283	0.196254429	4.856054911	1.19748E-06	6.16068E-06
F13A1	3.81540457	5.304955274	1.092508444	4.85575677	1.19928E-06	6.16759E-06
GHRL	6.062333728	4.316868908	0.889180218	4.854886356	1.20456E-06	6.19235E-06
LOC114116862	4.791094861	4.986757544	1.027240125	4.85451982	1.20679E-06	6.20143E-06
PRPF38B	476.1637684	1.038203944	0.213873309	4.854294118	1.20816E-06	6.20611E-06
CSTPP1	8.958231714	-2.834702549	0.584072523	-4.853340015	1.21399E-06	6.23163E-06
LOC101119721	75.30426294	1.084338433	0.223421599	4.853328587	1.21406E-06	6.23163E-06
XRCC4	12.91828293	1.995530674	0.411247969	4.852378188	1.2199E-06	6.25917E-06
VSTM4	3.512882515	5.164617943	1.064486854	4.851744224	1.2238E-06	6.27681E-06
LOC121819124	30.74269255	-1.233719321	0.254332072	-4.85082086	1.22952E-06	6.30368E-06
SLC19A2	22.02438931	-2.060656431	0.424817724	-4.850683745	1.23037E-06	6.30563E-06
LOC132657149	220.5427027	0.775624194	0.159917857	4.850141238	1.23374E-06	6.31805E-06
ST3GAL6	45.83444787	1.097067069	0.226189487	4.850212454	1.23329E-06	6.31805E-06
TBC1D12	27.88178186	1.288921717	0.265857942	4.84815953	1.24612E-06	6.37904E-06
ROMO1	76.91705432	-1.592452839	0.328483834	-4.847888012	1.24783E-06	6.38533E-06
MATN2	3.440382537	5.1183911	1.056015046	4.84689221	1.25411E-06	6.41499E-06
LRRC40	35.2165115	-1.145613882	0.236460779	-4.844836802	1.26716E-06	6.47928E-06
POLR1D	60.28355058	-1.049269245	0.216613605	-4.843967411	1.27272E-06	6.50522E-06
MRC2	3.349119816	5.096204418	1.052185836	4.84344518	1.27607E-06	6.51986E-06

RPS3A	97.3840254	-1.043061443	0.21536288	-4.843274031	1.27717E-06	6.52298E-06
MRPL22	13.8055535	-1.833996681	0.37867607	-4.843180823	1.27777E-06	6.52355E-06
CERS2	40.521704	-1.502045427	0.310178721	-4.84251603	1.28205E-06	6.54292E-06
REX1BD	23.33056271	-1.436257853	0.296638779	-4.841773755	1.28685E-06	6.56491E-06
ANKRD31	3.347858067	5.080162036	1.049349106	4.841250643	1.29025E-06	6.57971E-06
LOC121816250	18.18284264	-1.861535168	0.384684737	-4.83911886	1.30416E-06	6.64813E-06
SLC4A1AP	130.1375033	1.069654862	0.221136702	4.837075219	1.31764E-06	6.71427E-06
SERP1	53.74565826	1.016345876	0.210147877	4.836336632	1.32254E-06	6.73668E-06
TNFRSF1B	13.75412041	2.016816275	0.417037772	4.836051813	1.32443E-06	6.74377E-06
MTMR6	78.09128823	-0.882791782	0.182562363	-4.835562853	1.32769E-06	6.75779E-06
PLAUR	6.300321361	-5.016726858	1.037609726	-4.834888042	1.33221E-06	6.77817E-06
UGP2	43.42354049	1.07954213	0.223309558	4.834285376	1.33625E-06	6.79615E-06
GIT2	10.92158228	2.115497863	0.437693191	4.833289401	1.34295E-06	6.82766E-06
RANBP17	16.53317925	-1.833275853	0.379325285	-4.832991431	1.34497E-06	6.83529E-06
TLL1	3.582371747	5.172685466	1.070314783	4.832863701	1.34583E-06	6.83708E-06
TRIM65	35.14730554	-1.451687469	0.300414463	-4.832282223	1.34977E-06	6.85448E-06
CHN2	94.09274483	0.826135909	0.171042643	4.829999678	1.36533E-06	6.92826E-06
ISG15	17.91676306	-1.66489855	0.344699426	-4.830000935	1.36532E-06	6.92826E-06
WNK1	93.1249194	-0.843518214	0.174673919	-4.829102229	1.3715E-06	6.95691E-06
KCNMA1	35.26908568	1.373295742	0.284452969	4.827848163	1.38016E-06	6.9982E-06
LOC114115224	3.651983888	5.234245571	1.084445646	4.826655529	1.38845E-06	7.03755E-06
EEIG1	39.12022549	1.507368763	0.312331305	4.826185339	1.39173E-06	7.0515E-06
VWF	4.831536482	4.983411537	1.032691205	4.825655057	1.39544E-06	7.06761E-06
LOC114117256	89.46398372	1.149854518	0.238420886	4.822792744	1.41562E-06	7.16459E-06
TLR5	18.9382164	-1.536444108	0.318580103	-4.822787398	1.41566E-06	7.16459E-06
FAM200B	28.99906793	1.229878789	0.255038785	4.822320615	1.41898E-06	7.17722E-06
TXNIP	335.7228717	-0.896912697	0.185993283	-4.822285422	1.41923E-06	7.17722E-06
STEAP3	18.10305823	1.733735378	0.359578157	4.821581465	1.42425E-06	7.19987E-06
GPC6	10.58752561	2.190654796	0.454441077	4.820547499	1.43165E-06	7.23456E-06
CXXC1	57.80741457	1.310952882	0.271998045	4.819714351	1.43764E-06	7.26209E-06
ANKRD11	468.1662169	0.99971899	0.207461467	4.818817702	1.44411E-06	7.29204E-06
MRPL10	40.24947106	-1.03917048	0.215676477	-4.818191091	1.44866E-06	7.31221E-06
CENPV	13.0297288	-1.970085519	0.409085686	-4.815826083	1.46592E-06	7.39657E-06
STX4	39.8386342	1.222143867	0.253797085	4.81543698	1.46878E-06	7.4082E-06
SLIT2	4.466242522	4.896466126	1.017567901	4.811930605	1.49479E-06	7.53655E-06
STARD7	63.50243541	-1.648152492	0.342741299	-4.8087362	1.51887E-06	7.65508E-06
HAND2	3.369654276	5.102167343	1.061384833	4.80708522	1.53147E-06	7.71564E-06
TMEM129	15.6229567	-1.961565422	0.408063784	-4.807006891	1.53207E-06	7.71575E-06
LOC121818749	4.586513306	4.898187075	1.019168542	4.806061877	1.53932E-06	7.74937E-06
ZW10	37.43649791	-1.268192509	0.263883382	-4.805882423	1.5407E-06	7.75341E-06
KRTCAP3	44.48534013	-1.265700844	0.263377697	-4.805649295	1.5425E-06	7.75953E-06
LOC101107809	21.65699542	1.558291723	0.324342898	4.804457662	1.55172E-06	7.80294E-06

MMP11	17.53655047	-1.664878462	0.34659834	-4.803480772	1.55931E-06	7.83523E-06
ZSWIM6	76.75138583	0.982177521	0.204469203	4.80354747	1.55879E-06	7.83523E-06
DNAJB5	8.660974783	2.543196677	0.529462912	4.803351884	1.56031E-06	7.83733E-06
LOC114109767	11.38276014	5.688742986	1.184481946	4.802726632	1.5652E-06	7.8589E-06
GMFG	4.500854703	4.907145473	1.02180249	4.802440317	1.56744E-06	7.8672E-06
LARGE2	5.992231893	-3.68602468	0.767622715	-4.80187025	1.57191E-06	7.88667E-06
BPI	3.239680864	5.051225529	1.052113506	4.801027169	1.57854E-06	7.91698E-06
RHOJ	6.016873901	4.722125822	0.983596993	4.800874604	1.57974E-06	7.92004E-06
LOC114110105	3.423521214	5.100995216	1.062853018	4.799342083	1.59188E-06	7.97789E-06
ASH1L	213.175268	0.879054827	0.183247181	4.797098779	1.6098E-06	8.0647E-06
GTF3C2	21.20588827	1.456976352	0.30373981	4.796790888	1.61228E-06	8.07407E-06
CCR5	3.508164836	5.161214146	1.076121121	4.796127542	1.61762E-06	8.09781E-06
TLCD2	39.56117246	1.406162828	0.29320023	4.795913117	1.61935E-06	8.10344E-06
ANO1	6.150703018	4.813611143	1.003804516	4.795367094	1.62377E-06	8.12251E-06
PYROXD2	3.293759645	5.079092688	1.05928568	4.794828048	1.62814E-06	8.14133E-06
MALRD1	29.32211218	1.393683039	0.290718056	4.793933545	1.63542E-06	8.17468E-06
C1QTNF3	3.323578069	5.086336142	1.061127882	4.793330029	1.64035E-06	8.19625E-06
ARL11	28.67008296	1.659420371	0.346323453	4.791533339	1.65512E-06	8.26692E-06
LOC101115694	11.61045757	-2.10018577	0.438339802	-4.791227627	1.65764E-06	8.27643E-06
WNT5A	5.937108291	4.277208419	0.892815435	4.790697218	1.66203E-06	8.29524E-06
HSD17B8	106.6188839	0.807826128	0.168646733	4.790049082	1.66741E-06	8.31898E-06
LOC105616425	14.77686082	1.819543235	0.379930003	4.789153851	1.67486E-06	8.35305E-06
STIM1	15.5838379	1.720368207	0.359326629	4.787755956	1.68657E-06	8.40829E-06
CALHM5	3.257202323	5.05638078	1.056750411	4.784839187	1.71124E-06	8.52814E-06
GAS6	16.17853821	-2.356813431	0.492616704	-4.784274284	1.71606E-06	8.54897E-06
LOC101112223	46.89492539	-1.0390888	0.217208251	-4.78383669	1.7198E-06	8.56442E-06
LOC114115595	38.62302174	1.187658632	0.248307684	4.783012004	1.72688E-06	8.59644E-06
DHX57	23.73416392	1.627000282	0.34030796	4.780964523	1.74456E-06	8.68124E-06
YY1AP1	9.576931468	-2.458026047	0.514171794	-4.780554041	1.74813E-06	8.69575E-06
ATG7	54.1321479	0.971676469	0.203321653	4.779011252	1.76159E-06	8.75947E-06
SFI1	5.953506973	-3.98497325	0.833946326	-4.778452914	1.76649E-06	8.78056E-06
EFEMP1	6.136307759	3.938527397	0.824402035	4.777435315	1.77545E-06	8.82182E-06
PRPF40B	59.37088976	1.108215954	0.232030946	4.776155817	1.78678E-06	8.8748E-06
CMTM3	5.819303391	4.216211108	0.882890024	4.775465793	1.79292E-06	8.90198E-06
KIF2C	12.59526036	-2.086607205	0.437185578	-4.772818016	1.81666E-06	9.01651E-06
EIF3L	53.7333071	-1.217425501	0.255180405	-4.770842416	1.83457E-06	9.10203E-06
GIP	6.362153163	3.693737768	0.774423504	4.76966124	1.84536E-06	9.15216E-06
SNTB2	100.6475893	1.014162497	0.212682081	4.768443556	1.85655E-06	9.20423E-06
HDGF	271.9691313	-0.639290212	0.134127449	-4.766289187	1.8765E-06	9.2997E-06
CUL4A	68.54385123	-0.966338331	0.20280261	-4.764920596	1.88928E-06	9.35957E-06
ZNF197	24.33147499	1.424570811	0.298992668	4.764567704	1.89259E-06	9.37249E-06
PLCG1	12.53971599	1.989425715	0.417618677	4.763737413	1.9004E-06	9.40767E-06

MFGE8	13.69140501	-2.097136055	0.440323167	-4.762720225	1.91001E-06	9.45173E-06
LOC105607367	3.325040465	5.07562451	1.066115262	4.760859065	1.92771E-06	9.5358E-06
MOB4	102.9158178	-1.262154731	0.265138522	-4.760359684	1.93248E-06	9.55274E-06
SPPL3	58.20471419	-1.090319893	0.229041887	-4.760351501	1.93256E-06	9.55274E-06
PLTP	3.244868403	5.05053201	1.061086947	4.759772064	1.93812E-06	9.57666E-06
TJP3	13.54598308	-1.992935022	0.419103669	-4.755231628	1.98219E-06	9.79082E-06
TGFB2	6.354735838	3.602429501	0.757736625	4.754197408	1.99236E-06	9.83743E-06
PITPNA	76.29195996	0.941105435	0.197968613	4.753811321	1.99617E-06	9.8526E-06
LOC101106024	13.30941074	1.935350135	0.40723844	4.75237587	2.0104E-06	9.91917E-06
TUBAL3	10.90698511	2.25451569	0.474415222	4.752199308	2.01216E-06	9.92418E-06
LOC105613831	3.32224199	5.088859653	1.071122168	4.750961005	2.02452E-06	9.98147E-06
ACADM	46.13169141	1.136898162	0.239354674	4.749847337	2.0357E-06	1.00329E-05
CCNQ	17.08176257	-1.601328836	0.337142652	-4.749707073	2.03712E-06	1.00362E-05
WFS1	7.909654559	2.648206443	0.55756678	4.749577162	2.03842E-06	1.00389E-05
ICOS	3.512291274	5.126261599	1.079348973	4.749401472	2.0402E-06	1.00439E-05
RNPEPL1	9.447663781	2.394690535	0.504535372	4.746328338	2.07142E-06	1.01939E-05
FOXN2	15.76254942	1.66404297	0.350742578	4.74434264	2.09185E-06	1.02896E-05
SPRING1	64.89535233	0.908740957	0.191544181	4.744289031	2.0924E-06	1.02896E-05
POLR2C	26.03019012	-1.478559121	0.311660076	-4.74414029	2.09394E-06	1.02933E-05
SLC39A5	65.82286161	-1.04441956	0.220237677	-4.742238348	2.1137E-06	1.03866E-05
SEC24A	19.30804726	1.771598149	0.373785415	4.739612832	2.14127E-06	1.05183E-05
SMIM10L1	175.0511533	-0.677232002	0.142924557	-4.738387971	2.15425E-06	1.05781E-05
ADD3	149.6286087	0.680668579	0.143688542	4.737111046	2.16786E-06	1.06411E-05
SCOC	64.52978057	-1.295658657	0.273534668	-4.736725564	2.17199E-06	1.06574E-05
LOC101122728	14.27376199	-1.942801232	0.410195566	-4.736280435	2.17676E-06	1.06769E-05
CLSTN1	104.1205751	-0.803892813	0.169744302	-4.735904554	2.1808E-06	1.06928E-05
LOC101102142	3.181892745	5.034362496	1.06315044	4.735324659	2.18705E-06	1.07195E-05
NR5A2	19.34818972	2.019084616	0.426400485	4.735183679	2.18857E-06	1.0723E-05
THBS1	18.76943246	1.566269634	0.330867288	4.733830418	2.20322E-06	1.07908E-05
SPRED2	35.84169613	-1.166614776	0.246525211	-4.732233161	2.22063E-06	1.08721E-05
H1-4	12.55212952	2.223593448	0.469893952	4.732117614	2.2219E-06	1.08744E-05
LOC132659432	4.597392397	4.932015725	1.04232923	4.731725432	2.22619E-06	1.08914E-05
CREB3L4	18.23700487	2.570115031	0.543224891	4.731217357	2.23177E-06	1.09147E-05
LOC114117860	3.166891277	4.999424425	1.056787339	4.730776232	2.23663E-06	1.09305E-05
LOC132659433	3.166891277	4.999424425	1.056787339	4.730776232	2.23663E-06	1.09305E-05
CELA1	4.164557645	-4.971153733	1.050837249	-4.730659994	2.23791E-06	1.09327E-05
MRPS18C	19.92369038	-1.6277984	0.344221569	-4.728926206	2.2571E-06	1.10225E-05
ELF1	60.4753182	0.952140386	0.201352126	4.728732721	2.25926E-06	1.10289E-05
ATF7IP	121.7247952	0.714828201	0.151207358	4.727469693	2.27335E-06	1.10937E-05
TNIP1	46.01433953	1.003909647	0.212515187	4.723943087	2.31315E-06	1.12838E-05
ZBTB45	9.054169332	2.658668496	0.562876285	4.723362076	2.31977E-06	1.1312E-05
LOC114110633	3.249914575	5.038749226	1.066875913	4.722900916	2.32504E-06	1.13335E-05

CHD1L	58.48921567	1.079359772	0.228598153	4.721646946	2.33943E-06	1.13995E-05
LOC121819984	4.505504347	4.933509523	1.045310396	4.719659865	2.36239E-06	1.15072E-05
PARP14	119.0018832	0.814842485	0.172700542	4.718239292	2.37895E-06	1.15836E-05
LOC101121646	3.284533451	5.032616955	1.06668354	4.718003764	2.3817E-06	1.15918E-05
SF3A1	71.19668585	-0.936449538	0.198486647	-4.717947288	2.38236E-06	1.15918E-05
LACC1	119.2288017	0.756580501	0.160446398	4.715472021	2.41151E-06	1.17293E-05
HUNK	10.93694884	-2.136597013	0.453188039	-4.714592683	2.42195E-06	1.17715E-05
RTL6	18.10056265	-1.624065748	0.344472593	-4.714644304	2.42133E-06	1.17715E-05
CCDC59	41.026472	1.377318645	0.29216109	4.714243925	2.4261E-06	1.17874E-05
DOCK1	45.13651262	1.047718584	0.222289456	4.713307611	2.43728E-06	1.18375E-05
MPDU1	70.75911002	-1.033927435	0.219390114	-4.712734841	2.44414E-06	1.18665E-05
KANK2	11.2165333	2.376279874	0.504279036	4.712232121	2.45018E-06	1.18915E-05
TXN2	37.96227941	1.266192177	0.268756756	4.711294324	2.46148E-06	1.1942E-05
PIK3CA	100.3863224	0.795503053	0.168888811	4.710217629	2.47452E-06	1.20009E-05
GLG1	61.75624343	0.879541359	0.186749072	4.709749559	2.48021E-06	1.20241E-05
STX3	21.76335617	-1.67605719	0.355907796	-4.709245516	2.48635E-06	1.20495E-05
SLC16A10	52.48125624	-1.238876466	0.263092981	-4.708892134	2.49067E-06	1.20661E-05
LOC101114285	3.228681095	5.059039149	1.074413599	4.708651446	2.49361E-06	1.20759E-05
MAPK8IP3	7.960811943	-2.665804743	0.566212494	-4.708134791	2.49994E-06	1.21022E-05
CEP104	8.902189708	2.646651016	0.562194455	4.707714552	2.5051E-06	1.21228E-05
TSHZ3	3.197690284	5.025498528	1.067546458	4.707522083	2.50746E-06	1.21298E-05
MARCHF7	106.8765087	-0.793536343	0.16858029	-4.707171534	2.51178E-06	1.21463E-05
LOC114108769	9.055314311	-2.584354155	0.549504516	-4.703062635	2.56288E-06	1.23845E-05
NR1H3	33.00294023	1.512613323	0.321622935	4.703064232	2.56286E-06	1.23845E-05
MZB1	3.209365995	5.025605496	1.068674923	4.702651282	2.56805E-06	1.2405E-05
LOC114109735	7.233012201	-3.000172864	0.638012041	-4.702376558	2.57151E-06	1.24172E-05
PCBP2	274.43651	0.770291744	0.163822387	4.701993152	2.57634E-06	1.2436E-05
LOC101111409	3.172430457	5.017531837	1.067232737	4.701441087	2.58332E-06	1.24652E-05
TRIM28	12.84461198	-1.970336529	0.419114736	-4.701186478	2.58654E-06	1.24763E-05
SMPD3	22.27173222	1.444878742	0.30738932	4.700484524	2.59545E-06	1.25147E-05
DDX5	278.3437726	0.740645845	0.157652607	4.697961275	2.62771E-06	1.26657E-05
PNISR	1119.766323	0.912575174	0.194320848	4.696228866	2.65009E-06	1.27689E-05
PCNX1	63.33161632	1.008164195	0.214683703	4.696044369	2.65248E-06	1.27759E-05
KPNA2	55.09739522	-1.054130446	0.224557476	-4.694256739	2.67578E-06	1.28834E-05
LOC105611592	27.30774634	1.768055873	0.376806403	4.692212925	2.70266E-06	1.30082E-05
OAZ2	11.3473249	-2.12188193	0.452658125	-4.687603759	2.76423E-06	1.32997E-05
SP110	34.55146669	1.27389635	0.271818475	4.68657014	2.77822E-06	1.33622E-05
LOC101121285	4.399853139	4.854303647	1.036249724	4.684492102	2.80655E-06	1.34936E-05
SMO	3.171655528	4.98487884	1.064274114	4.683829828	2.81564E-06	1.35325E-05
CNOT1	59.74863522	-0.993157762	0.212053023	-4.683535033	2.81969E-06	1.35471E-05
NR0B2	3.607674321	5.183571569	1.107216544	4.681624022	2.84611E-06	1.36691E-05
DGKB	3.17131083	5.016960477	1.071778138	4.68096922	2.85522E-06	1.37079E-05

CAMKMT	6.351598386	-3.409028526	0.728304656	-4.680772665	2.85796E-06	1.37161E-05
MRPL43	62.13967502	1.033507156	0.220812882	4.680465855	2.86224E-06	1.37318E-05
IFNGR2	64.91758155	0.889504159	0.190198413	4.676717034	2.91504E-06	1.39801E-05
SLC20A2	40.57598486	1.133548784	0.242399037	4.676374954	2.91991E-06	1.39984E-05
SOBP	6.517745167	3.568915135	0.763301744	4.675628167	2.93055E-06	1.40444E-05
LOC105601873	210.8310083	-0.718899477	0.153797784	-4.674316237	2.94935E-06	1.41243E-05
PTPN22	4.135910207	4.793959723	1.025590416	4.674341381	2.94898E-06	1.41243E-05
CTU2	19.17580471	-1.449099159	0.310023406	-4.674160498	2.95158E-06	1.4125E-05
SWAP70	43.50528329	1.001095844	0.214176689	4.674158755	2.95161E-06	1.4125E-05
LOC132659254	4.388114418	4.823982005	1.032101758	4.673940303	2.95475E-06	1.4135E-05
MX2	7.664991599	-2.530253845	0.541381103	-4.673701817	2.95819E-06	1.41464E-05
ATP5F1E	174.3151022	-0.794596666	0.17004464	-4.672870984	2.97018E-06	1.41987E-05
EPHB4	35.72327207	-1.44862823	0.310151415	-4.670712947	3.00156E-06	1.43435E-05
C17H22orf39	36.45766208	1.132502238	0.242527452	4.669583702	3.01811E-06	1.44174E-05
STAT3	7.894438512	2.743412006	0.587554325	4.669205707	3.02367E-06	1.44388E-05
FADS2	18.28826784	-1.620244894	0.347203181	-4.66656121	3.06282E-06	1.46206E-05
PDLIM5	90.01321035	-0.853649849	0.182963398	-4.665686459	3.07588E-06	1.46777E-05
FUT11	22.10606523	-1.700033456	0.364379116	-4.665562266	3.07774E-06	1.46813E-05
DDI2	119.1738585	0.823504929	0.176565008	4.664032461	3.10072E-06	1.47857E-05
NAGLU	16.62604905	-2.170813013	0.465500323	-4.66339744	3.11031E-06	1.48261E-05
DICER1	100.4585513	-0.798177945	0.171197335	-4.662326937	3.12654E-06	1.48982E-05
GPX4	81.92541396	-1.349458553	0.289456257	-4.662046575	3.1308E-06	1.49132E-05
ODC1	45.11420983	1.18849777	0.254974858	4.661235153	3.14317E-06	1.49667E-05
DHX32	23.95156312	1.392064821	0.298676246	4.660781839	3.1501E-06	1.49944E-05
CCDC107	16.59768065	1.586492063	0.340430347	4.660254527	3.15819E-06	1.50275E-05
ENPP2	5.102119707	4.507800004	0.96730646	4.660157035	3.15968E-06	1.50293E-05
CYTH2	6.760402249	-3.170170267	0.680512216	-4.658506038	3.18512E-06	1.51449E-05
FKBP7	14.15033068	1.855059937	0.398221577	4.658361182	3.18737E-06	1.51502E-05
BTF3	4.390946915	-5.131852439	1.102305827	-4.65556138	3.23099E-06	1.53521E-05
LOC121817090	8.811134378	-2.490204716	0.535037355	-4.654263277	3.25141E-06	1.54436E-05
MGAT4B	31.6262045	-1.651396834	0.354824605	-4.654121529	3.25365E-06	1.54488E-05
CLINT1	133.0547314	-0.676325786	0.145329239	-4.653748916	3.25954E-06	1.54712E-05
SLC30A9	55.67827881	-1.010661327	0.217216885	-4.652775153	3.27497E-06	1.5539E-05
GSDME	6.566517113	3.578103106	0.769422348	4.650375852	3.31331E-06	1.57153E-05
GBF1	47.43586976	1.146007529	0.246590419	4.647413048	3.36124E-06	1.5937E-05
CDH3	11.42526548	-2.630773238	0.566136591	-4.646887836	3.3698E-06	1.59719E-05
NOX5	5.175024509	4.490278155	0.966485545	4.645985837	3.38456E-06	1.60362E-05
ELOF1	40.66569597	1.177668408	0.253506325	4.64551883	3.39223E-06	1.60668E-05
MYH14	35.50665619	1.219910574	0.262681435	4.644068489	3.41614E-06	1.61743E-05
ITGA1	137.1999694	0.762358281	0.164222905	4.64221651	3.44691E-06	1.63143E-05
LOC101121820	11.79131473	2.036579397	0.438761681	4.641652833	3.45633E-06	1.6353E-05
MS4A8	47.44521833	1.45133022	0.312725836	4.640902836	3.4689E-06	1.64067E-05

MANSC1	45.58197809	-1.200621364	0.258728763	-4.640463438	3.47629E-06	1.643E-05
RABL6	78.90186247	1.214635614	0.261747601	4.640484233	3.47594E-06	1.643E-05
YWHAZ	283.9957246	-0.630756467	0.135929745	-4.640312288	3.47883E-06	1.64362E-05
AHCTF1	52.69100508	1.045961652	0.225514964	4.638103096	3.51621E-06	1.6607E-05
PTPN1	122.5078267	0.749319074	0.161610341	4.636578744	3.54223E-06	1.67239E-05
LOC105605770	3.174738248	5.002999653	1.07927745	4.63550837	3.56061E-06	1.68048E-05
PARD3	69.11067806	1.006864167	0.21721198	4.635398868	3.5625E-06	1.68078E-05
LOC132659914	9.055990525	2.496579581	0.538821589	4.633406735	3.59697E-06	1.69533E-05
LRRC8B	16.61962713	-1.629392465	0.351662691	-4.633395888	3.59716E-06	1.69533E-05
TMUB2	15.18714134	1.777844429	0.383696607	4.633464029	3.59597E-06	1.69533E-05
LOC101121371	16.92830036	-2.041953024	0.440777766	-4.632613491	3.61078E-06	1.70115E-05
ABCA1	8.95260734	2.354146458	0.508192025	4.632395519	3.61459E-06	1.70235E-05
C7H15orf48	16.46537653	2.251548453	0.486078218	4.63207025	3.62027E-06	1.70442E-05
GIMAP6	3.224259614	5.058862635	1.092832174	4.629130396	3.67204E-06	1.72819E-05
MGST1	128.3284615	0.8484657	0.183485566	4.624155007	3.76129E-06	1.76956E-05
DIP2B	26.64336271	1.317970288	0.285027624	4.624008956	3.76394E-06	1.77019E-05
GATAD2A	72.72132745	-0.942702354	0.203921356	-4.622872123	3.78463E-06	1.7793E-05
GTF2E1	44.13011369	-1.011437347	0.218793277	-4.622799022	3.78596E-06	1.7793E-05
TTC33	25.23087542	1.260304716	0.272641895	4.622564394	3.79025E-06	1.78069E-05
LOC132657289	5.230749275	4.58329233	0.991532923	4.622430808	3.79269E-06	1.78121E-05
TMEM176B	3.229134396	5.030736677	1.0888714	4.620138502	3.83484E-06	1.80037E-05
ESS2	24.46773785	-1.522627825	0.329606315	-4.619534743	3.84601E-06	1.80498E-05
CCSER2	53.59611529	1.033569858	0.22375424	4.619219083	3.85187E-06	1.80709E-05
AKAP7	18.68342237	1.600867355	0.346668536	4.617861695	3.87715E-06	1.81831E-05
NUPR1	69.96357353	1.217880351	0.263758917	4.617399721	3.88578E-06	1.82173E-05
HCK	3.055656002	4.935676083	1.068967978	4.61723474	3.88887E-06	1.82253E-05
FABP6	4.15990159	4.803790531	1.04051314	4.616751436	3.89794E-06	1.82614E-05
ATP5MG	368.1727206	-0.843456491	0.182743672	-4.615516819	3.92118E-06	1.83639E-05
LOC105607964	8.294017004	-2.417612447	0.523919159	-4.614476116	3.94088E-06	1.84377E-05
MRPL3	7.810206999	-3.370821291	0.730490122	-4.61446526	3.94109E-06	1.84377E-05
NMRK1	75.94873312	-0.803919446	0.174214491	-4.614538334	3.9397E-06	1.84377E-05
BUB1	18.66360419	-1.47411625	0.319483046	-4.614067214	3.94865E-06	1.84666E-05
SGK2	4.557817245	4.933513844	1.069350643	4.613560458	3.95829E-06	1.85053E-05
TACC2	82.38227956	0.783072123	0.169761704	4.612772517	3.97333E-06	1.85691E-05
IER3IP1	75.03047011	0.940295248	0.203949019	4.61044262	4.01813E-06	1.87719E-05
GMPPA	10.04875521	2.076951043	0.450616879	4.60912837	4.04361E-06	1.88843E-05
C11H17orf58	4.21910324	4.814531772	1.044884677	4.607715931	4.07116E-06	1.89997E-05
PRPF8	53.98586409	-0.981273936	0.212962048	-4.607740884	4.07067E-06	1.89997E-05
LMNB2	34.67613537	-1.147060374	0.248980971	-4.607020244	4.0848E-06	1.90567E-05
KAT7	92.85370865	0.84493343	0.183459518	4.605557891	4.11362E-06	1.91844E-05
MLLT3	57.44969769	1.231760214	0.267460714	4.605387434	4.11699E-06	1.91935E-05
XPOT	152.201255	-0.83429453	0.18116999	-4.605037138	4.12392E-06	1.92191E-05

MMP19	3.057416124	4.951575129	1.075306643	4.604802884	4.12857E-06	1.9234E-05
ATP5MK	18.7344325	-2.244874143	0.48752387	-4.604644573	4.13171E-06	1.9242E-05
RPS6	201.6816523	-0.808671215	0.175640144	-4.604136607	4.14181E-06	1.92823E-05
UST	2.968436546	4.925386447	1.069947953	4.60338882	4.15671E-06	1.93449E-05
ZNF384	9.914496024	2.118853629	0.460329827	4.602903191	4.16642E-06	1.93833E-05
ATP6V1G1	91.1664512	-0.860984703	0.187067858	-4.602526113	4.17397E-06	1.94117E-05
NR4A1	5.378735228	4.097571342	0.890316029	4.602378489	4.17693E-06	1.94187E-05
NDUFC2	40.37417434	-1.12533595	0.244553009	-4.601603374	4.19251E-06	1.94844E-05
TEX2	59.35786978	-1.163295322	0.252806792	-4.601519262	4.1942E-06	1.94855E-05
ZNG1A	31.88556389	1.220189683	0.265219462	4.600679273	4.21115E-06	1.95574E-05
TET3	35.50331149	1.248644228	0.271453285	4.599849394	4.22797E-06	1.96287E-05
RASAL3	5.01203169	4.464356427	0.970718783	4.599021371	4.2448E-06	1.97E-05
PTGFR	10.92665297	2.057441668	0.447459437	4.598051793	4.2646E-06	1.97831E-05
ROBO4	3.046865045	4.965473553	1.07992036	4.597999758	4.26566E-06	1.97831E-05
PBXIP1	66.4944767	0.907973708	0.197500697	4.597318998	4.27962E-06	1.98409E-05
OMG	2.945799949	4.919775829	1.070198639	4.597067919	4.28478E-06	1.9858E-05
LRRFIP2	75.58119921	-0.815236951	0.177341631	-4.596985742	4.28647E-06	1.98589E-05
KLHDC8B	5.154356104	4.072148318	0.885943599	4.596396797	4.2986E-06	1.99082E-05
ELAVL1	29.67161879	1.31851918	0.286866073	4.596288323	4.30083E-06	1.99117E-05
STAP1	3.177575607	4.974091911	1.082642408	4.59439966	4.33997E-06	2.00859E-05
TSKU	15.31006809	-2.164862854	0.471423439	-4.592183324	4.38633E-06	2.02934E-05
ID3	23.96801711	-1.401775532	0.305310027	-4.591318354	4.40455E-06	2.03707E-05
CLSTN2	2.974429027	4.91016168	1.070243352	4.587892717	4.47743E-06	2.07005E-05
B3GALT6	36.74101479	-1.167495109	0.25455416	-4.586431075	4.50887E-06	2.08387E-05
SERPINF1	3.061303785	4.954933064	1.080668258	4.585063942	4.53848E-06	2.09683E-05
SDR16C5	6.109176725	-3.701805498	0.807619018	-4.583603672	4.5703E-06	2.1108E-05
NDN	2.973273199	4.909736298	1.071572647	4.581804427	4.60981E-06	2.12831E-05
AGO3	89.60202978	0.800638557	0.174791333	4.580539229	4.63779E-06	2.14049E-05
TCF7L2	96.03438956	0.720008896	0.157220053	4.579625077	4.6581E-06	2.14913E-05
MDFI	6.97957867	-2.913418846	0.636381895	-4.578098256	4.69222E-06	2.16412E-05
KCTD1	5.910364371	3.512271992	0.767257416	4.577697026	4.70123E-06	2.16753E-05
FAM83E	26.02998249	1.440147386	0.31461076	4.577552865	4.70447E-06	2.16827E-05
PUS7	7.804841091	-2.918665466	0.637772481	-4.576342743	4.73175E-06	2.1801E-05
SLC35F1	3.23991503	5.039741963	1.101704668	4.574494516	4.77371E-06	2.19867E-05
LOC101121256	11.09242061	2.552893847	0.558157641	4.573786435	4.78988E-06	2.20536E-05
VTI1A	77.8904208	0.865152044	0.189173837	4.573317632	4.80062E-06	2.20954E-05
SVIL	17.11079807	1.785650204	0.390479378	4.572969289	4.80861E-06	2.21246E-05
SFXN5	6.355549243	3.423225956	0.74872916	4.572048398	4.82979E-06	2.22144E-05
KCNE1	5.423235881	-4.238043206	0.926973552	-4.571913832	4.8329E-06	2.2221E-05
CCDC34	87.65957184	1.102272712	0.24117213	4.570481303	4.86605E-06	2.23615E-05
MAGOHB	13.48016795	-1.824115225	0.399110738	-4.570448881	4.86681E-06	2.23615E-05
DZIP3	10.4049562	2.115481109	0.462890586	4.570153671	4.87367E-06	2.23854E-05

FAM149A	188.8963932	1.245884024	0.272620335	4.570033361	4.87647E-06	2.23905E-05
LOC121819482	2.908999591	4.873514903	1.066424718	4.569956811	4.87825E-06	2.2391E-05
LOC105613829	5.420471065	4.534083521	0.992235924	4.569561947	4.88745E-06	2.24255E-05
NRXN1	4.144408727	4.763721188	1.042651534	4.568852617	4.90401E-06	2.24938E-05
FGD4	100.104703	-0.788947432	0.172710564	-4.56803229	4.92324E-06	2.25743E-05
CAT	17.46548243	1.766907463	0.386856287	4.567348456	4.93933E-06	2.26248E-05
FAM204A	44.58784385	-1.232432733	0.269835066	-4.567355733	4.93915E-06	2.26248E-05
TBKBP1	10.72074424	-2.17943328	0.477171891	-4.567396617	4.93819E-06	2.26248E-05
RC3H2	77.62672459	0.918598749	0.2011296	4.567198197	4.94287E-06	2.26332E-05
PLAC9	3.198018022	5.008503351	1.097028702	4.565517147	4.98264E-06	2.28075E-05
GPM6B	3.088983871	4.964801417	1.088549913	4.560931346	5.09272E-06	2.33034E-05
KIF2A	106.811132	-0.738482124	0.162014101	-4.558134869	5.16099E-06	2.36077E-05
TMEM98	45.54718789	-1.27073509	0.278827856	-4.557417994	5.17863E-06	2.36803E-05
PLAU	7.391731316	-4.007018654	0.879525603	-4.555886311	5.21651E-06	2.38454E-05
ZZEF1	43.33369214	1.063832233	0.233608171	4.553917049	5.26561E-06	2.40616E-05
CENPS	26.24178234	-1.330007527	0.29207824	-4.553600186	5.27355E-06	2.40897E-05
WBP1	13.71935529	-1.714479067	0.376547946	-4.553149434	5.28487E-06	2.41331E-05
CUL1	70.37294522	-0.960601622	0.210986288	-4.552910195	5.29089E-06	2.41523E-05
BCHE	5.126852071	4.493414263	0.987218817	4.551588956	5.32423E-06	2.42962E-05
ZNF777	14.94841793	-1.812897935	0.39831662	-4.551399175	5.32903E-06	2.43098E-05
INPP5E	8.841637873	-2.265671505	0.497845109	-4.550956639	5.34026E-06	2.43527E-05
CCDC86	16.4523839	-1.834469324	0.40310182	-4.55088326	5.34212E-06	2.43529E-05
KMT5A	125.0549896	-0.883354674	0.194118129	-4.550603694	5.34922E-06	2.4377E-05
FAF2	44.31438179	1.021715715	0.224639322	4.548249633	5.40939E-06	2.46428E-05
LOC101102085	2.859836054	4.87691345	1.072367644	4.547799886	5.42096E-06	2.46871E-05
CNR2	3.046227232	4.936052991	1.085403575	4.547666053	5.42441E-06	2.46944E-05
SOX11	3.323319227	5.053630109	1.111642334	4.546093607	5.46507E-06	2.4871E-05
CIART	5.811819266	3.468183826	0.763015581	4.545364357	5.48403E-06	2.49488E-05
CRYBG3	9.601122419	2.472840675	0.544087123	4.544935121	5.49522E-06	2.49912E-05
THOC5	13.23972103	-1.715836239	0.377671892	-4.543192852	5.54085E-06	2.51901E-05
PTPRB	16.36831417	1.698064246	0.373786384	4.542873463	5.54926E-06	2.52198E-05
ARHGEF10	3.878115854	4.686129502	1.032312775	4.53944736	5.64019E-06	2.56243E-05
CXCL10	7.011522277	3.122493789	0.68791952	4.539039375	5.65111E-06	2.56652E-05
ITCH	67.53912777	-0.90788905	0.200026832	-4.538836311	5.65655E-06	2.56812E-05
LOC105614340	11.52042833	-2.019587803	0.445043329	-4.537957706	5.68016E-06	2.57797E-05
SGPL1	17.30762429	-1.618888971	0.35680767	-4.537147336	5.70203E-06	2.58701E-05
ADIPOR1	23.4767441	-1.490868724	0.328751774	-4.534937424	5.76206E-06	2.61336E-05
NAV3	5.144179159	4.006372739	0.883527087	4.53452169	5.77342E-06	2.61762E-05
LOC121816832	3.154028147	4.968254585	1.095712608	4.534267972	5.78036E-06	2.61988E-05
MFSD9	20.34063619	1.429682197	0.315366358	4.533401105	5.80414E-06	2.62903E-05
PRKCH	6.806925181	3.371038392	0.743602312	4.533388797	5.80448E-06	2.62903E-05
SECISBP2	31.68833791	-1.222007441	0.269631664	-4.532136252	5.83902E-06	2.64378E-05

GLP1R	10.95457327	6.21654661	1.371989091	4.531046677	5.86922E-06	2.65655E-05
ZDHHC4	43.11597839	-0.982838894	0.217049513	-4.528178295	5.94944E-06	2.69195E-05
SEMA6D	22.41418534	1.475299598	0.325896317	4.526898658	5.98556E-06	2.70738E-05
RHOU	62.78784068	-0.860191301	0.190215329	-4.522197589	6.12009E-06	2.76729E-05
DCTN5	36.58506751	-1.074467305	0.23763696	-4.521465459	6.1413E-06	2.77594E-05
P3H4	13.7645713	1.916797565	0.424041438	4.520307197	6.175E-06	2.79023E-05
SPATA6	23.0766984	1.407890766	0.311469497	4.520156163	6.1794E-06	2.79128E-05
TCP11L2	4.821910793	4.394825479	0.972442021	4.519370189	6.20239E-06	2.80071E-05
ATG5	28.40691425	1.220908227	0.270181241	4.518848984	6.21767E-06	2.80667E-05
PANK1	34.8135735	1.282694571	0.283909544	4.517969183	6.24356E-06	2.8174E-05
HAP1	4.419752152	4.857814758	1.075269262	4.517765858	6.24955E-06	2.81915E-05
AKIRIN2	15.48736185	1.630683717	0.361065885	4.516305153	6.2928E-06	2.8377E-05
ANKRD27	76.89343832	-1.063334656	0.235474639	-4.515707772	6.31056E-06	2.84476E-05
COQ3	16.55140468	-1.568693024	0.347444157	-4.51495008	6.33317E-06	2.85398E-05
HHLA2	5.361299684	4.059730792	0.899431154	4.513664857	6.37169E-06	2.87037E-05
EFCC1	3.091609029	4.949577159	1.096832426	4.512610168	6.40347E-06	2.88372E-05
LOC101116099	8.9063955	2.371620678	0.525694509	4.5114047	6.43997E-06	2.89918E-05
GRAMD1B	8.891294503	2.319849164	0.514352823	4.510229283	6.47576E-06	2.91431E-05
PELI2	3.77792037	4.650348996	1.031246894	4.509442913	6.49981E-06	2.92415E-05
DDAH2	53.8474364	-1.076605381	0.23876232	-4.509109231	6.51004E-06	2.92777E-05
DPH5	34.37198997	-1.118013426	0.248071416	-4.506820842	6.58062E-06	2.9578E-05
KMT2B	35.81245532	1.571625831	0.348723215	4.506800129	6.58126E-06	2.9578E-05
FILIP1L	141.3550469	0.928900221	0.20627257	4.50326586	6.69171E-06	3.00643E-05
ATXN7L3B	205.5957365	-0.937213188	0.208123465	-4.503159632	6.69506E-06	3.00693E-05
ZC3H12A	10.35700915	-2.108384563	0.468282189	-4.50238043	6.71966E-06	3.01696E-05
ZNF7	24.84102939	-1.446113939	0.321217859	-4.501972415	6.73257E-06	3.02174E-05
PTGER4	61.8467587	1.18652707	0.263571449	4.50172838	6.74031E-06	3.0242E-05
CNGB1	2.994503997	4.90398713	1.089563741	4.500872178	6.76752E-06	3.03539E-05
SMURF2	34.76508755	1.151776641	0.255966898	4.499709343	6.80464E-06	3.05102E-05
MTG1	10.60932459	-2.00940202	0.4466149	-4.499182679	6.82152E-06	3.05756E-05
RASSF1	29.53835558	1.248193198	0.277465834	4.498547375	6.84194E-06	3.06568E-05
BCAS4	11.53995122	-1.933395133	0.429896857	-4.497346523	6.88068E-06	3.08201E-05
MYPOP	6.024724875	-3.178553756	0.706846229	-4.496810799	6.89803E-06	3.08875E-05
C2H9orf40	12.15862454	-2.314447449	0.514697824	-4.496711158	6.90126E-06	3.08916E-05
GPR21	5.552522753	3.671622831	0.816615268	4.496147667	6.91957E-06	3.09632E-05
OGFOD3	14.62810348	2.083605776	0.463511321	4.495264042	6.94837E-06	3.10816E-05
NOSTRIN	44.68460374	1.037082661	0.230723105	4.494923298	6.9595E-06	3.1121E-05
SNRPF	260.2599384	-0.859616645	0.191248625	-4.494759862	6.96485E-06	3.11345E-05
TRIM66	22.82056245	-1.337765204	0.297680962	-4.493956199	6.9912E-06	3.12419E-05
ACD	17.90258698	-1.548172326	0.344550745	-4.493307156	7.01255E-06	3.13268E-05
XCR1	2.779792238	4.81592535	1.071860032	4.493054323	7.02089E-06	3.13535E-05
RNF41	10.16548042	-2.014295618	0.44837735	-4.492411622	7.04211E-06	3.14378E-05

PSMB6	11.51928058	-2.549327812	0.567944022	-4.488695564	7.16606E-06	3.19805E-05
FGFRL1	7.999248956	-2.308232064	0.514240855	-4.488620537	7.16859E-06	3.19811E-05
RFLNB	29.49570768	1.379297443	0.307311196	4.488275921	7.18019E-06	3.20222E-05
DDX6	120.0131879	0.870678352	0.194006945	4.487872084	7.19381E-06	3.20722E-05
UBB	8.037843397	-2.622898153	0.584554139	-4.487006386	7.22309E-06	3.2192E-05
LOC101108674	57.37154009	-1.012905422	0.225824638	-4.485362763	7.279E-06	3.24304E-05
PFKL	54.43023283	-0.983517607	0.219277547	-4.485263642	7.28238E-06	3.24346E-05
FRMD3	5.863679809	3.45404239	0.770119974	4.48507052	7.28898E-06	3.24532E-05
PSMA3	61.64802363	-1.148404005	0.256081609	-4.484523545	7.3077E-06	3.25257E-05
YIF1B	5.625698006	3.468405604	0.773588314	4.483528956	7.34186E-06	3.26669E-05
MCM6	53.09314022	-1.004718526	0.22409895	-4.483370074	7.34733E-06	3.26804E-05
TRAPPC3L	14.93536569	-1.678625729	0.374686388	-4.480081969	7.46144E-06	3.31768E-05
ENDOV	16.43775996	-1.476072117	0.329484514	-4.479943828	7.46627E-06	3.31873E-05
PPIL4	50.22302137	1.111929686	0.248209516	4.479802802	7.4712E-06	3.31982E-05
CD300LG	2.907980879	4.876617695	1.088791913	4.478925344	7.50198E-06	3.33238E-05
CH25H	2.932864418	4.881735349	1.090554579	4.476378754	7.59198E-06	3.37124E-05
LOC101121414	6.5538326	2.800766109	0.625748527	4.475865288	7.61025E-06	3.37823E-05
CDA	7.433988635	-2.592764393	0.579367865	-4.47516086	7.63538E-06	3.38826E-05
COL16A1	3.797208646	4.649825434	1.039092781	4.474889555	7.64508E-06	3.39144E-05
EIF1	65.90665644	-1.092373507	0.244137697	-4.47441555	7.66206E-06	3.39785E-05
ITGA9	8.323537648	2.542932623	0.568569943	4.472506247	7.73081E-06	3.4272E-05
ENDOD1	51.46404804	-1.050126932	0.234892467	-4.470670971	7.79746E-06	3.45514E-05
PLCB3	47.37021636	1.099004594	0.245827753	4.470628639	7.799E-06	3.45514E-05
PPP6R1	24.03811384	-1.438499747	0.321775598	-4.470506017	7.80347E-06	3.45597E-05
FOLR2	3.805186038	4.672316746	1.045404969	4.469384481	7.8445E-06	3.4724E-05
TRMT6	34.42870441	-1.165435718	0.260764237	-4.469308101	7.8473E-06	3.4724E-05
WRNIP1	69.05719027	-0.824740424	0.184535443	-4.469279238	7.84836E-06	3.4724E-05
LOC114111288	3.419217076	-4.719044112	1.056169368	-4.468075153	7.89266E-06	3.48932E-05
OXTR	3.408590777	-4.759046912	1.065133918	-4.468026818	7.89444E-06	3.48932E-05
ZNF335	11.24604481	-2.159405755	0.483299027	-4.468053179	7.89347E-06	3.48932E-05
BATF	3.12280317	4.946377149	1.10722983	4.467344552	7.91965E-06	3.49931E-05
TRAK2	28.16329623	1.150858613	0.257634379	4.467022676	7.93157E-06	3.50342E-05
MYO18A	35.77636651	1.225186777	0.274297178	4.466640108	7.94576E-06	3.50853E-05
LOC101114575	8.023230835	2.501798671	0.560148576	4.466312649	7.95793E-06	3.51274E-05
XYLB	36.98791782	1.271770664	0.284825392	4.465088779	8.00356E-06	3.53171E-05
SP5	5.247324192	-4.225723385	0.946431078	-4.464903448	8.01049E-06	3.5336E-05
PIAS4	39.60149517	-1.048219224	0.234865993	-4.46305236	8.08003E-06	3.5631E-05
MMP1	25.06701478	-1.730101742	0.387688822	-4.462604137	8.09696E-06	3.56939E-05
GCDH	22.67435159	1.312735095	0.294171743	4.46247856	8.1017E-06	3.57031E-05
SPDYA	14.72859066	1.873977388	0.419994637	4.461907898	8.12331E-06	3.57865E-05
DNASE2	24.48391762	-1.455032141	0.32631797	-4.458939674	8.23661E-06	3.62736E-05
SF3B3	188.4735104	-0.820682415	0.184078336	-4.458332443	8.25997E-06	3.63646E-05

HSD3B7	6.703541582	-2.695851922	0.604701791	-4.458151046	8.26696E-06	3.63834E-05
KEF53_p08	2534.342251	1.082033628	0.2427542	4.457321971	8.29899E-06	3.65123E-05
LOC101108032	18.74317653	1.60116163	0.359282549	4.456552748	8.32881E-06	3.66314E-05
RPS6KB1	151.8047237	-0.726367386	0.162992012	-4.456460036	8.33241E-06	3.66352E-05
MCRIP1	86.21048498	1.19098646	0.267274169	4.456047749	8.34844E-06	3.66936E-05
BDKRB2	2.797773187	4.838583206	1.085893354	4.455854885	8.35595E-06	3.67146E-05
SH3KBP1	20.06055513	1.418165658	0.31837372	4.454405533	8.4126E-06	3.69513E-05
LOC114108602	8.307786942	-3.108236739	0.697943292	-4.453423041	8.4512E-06	3.71087E-05
ZG16	2.829694502	4.850164081	1.089451637	4.45193152	8.51013E-06	3.73551E-05
LRFN4	8.287022344	-2.552371349	0.573365181	-4.451563217	8.52474E-06	3.7407E-05
IHH	37.50087184	1.07400288	0.24141114	4.448853846	8.63297E-06	3.78695E-05
KEF53_p04	51.400695	1.283595198	0.288651444	4.446869142	8.71309E-06	3.82084E-05
SETD4	15.99303514	-1.711643888	0.385057302	-4.445166676	8.78238E-06	3.84996E-05
LRRCC1	91.14624658	1.013038637	0.227920851	4.444694859	8.80167E-06	3.85715E-05
TAF1	94.76723723	0.882852995	0.198669492	4.443827716	8.83724E-06	3.87147E-05
TRPC6	2.739634181	4.80364795	1.081200846	4.442882158	8.87618E-06	3.88725E-05
SHTN1	38.53753794	-1.061136148	0.23888817	-4.441978632	8.91354E-06	3.90234E-05
FRK	23.42321759	1.301390348	0.293014229	4.441389592	8.93798E-06	3.9109E-05
LOC121816163	2.89182709	4.901617663	1.103628349	4.441366213	8.93895E-06	3.9109E-05
CCT4	31.60712368	-1.268717136	0.285694735	-4.440813858	8.96193E-06	3.91967E-05
PITPNM1	18.43634316	1.547577676	0.348599197	4.439418361	9.02023E-06	3.94388E-05
RFX6	6.23097358	-2.819093478	0.63502869	-4.439316714	9.02449E-06	3.94445E-05
LIMK2	23.74836409	-2.153656892	0.485147986	-4.439175164	9.03043E-06	3.94576E-05
GLP2R	2.821250926	4.829638722	1.088298643	4.437788059	9.0888E-06	3.96997E-05
OAT	95.29344181	0.977394804	0.220256092	4.437538116	9.09936E-06	3.97328E-05
BAZ1A	319.4519775	0.960350875	0.216454243	4.436738509	9.13321E-06	3.98676E-05
LOC114114817	3.235018542	-4.697305009	1.058857653	-4.43620065	9.15605E-06	3.99542E-05
ELP3	38.79629741	-1.280592113	0.28885106	-4.433399391	9.27588E-06	4.04639E-05
EFR3B	35.29349117	-1.332269721	0.300539534	-4.432926686	9.29625E-06	4.05395E-05
LONRF1	67.50967709	-0.914351582	0.206285908	-4.432448105	9.31691E-06	4.06164E-05
GEMIN6	18.19361764	-1.449580926	0.327125871	-4.431263482	9.36825E-06	4.08269E-05
RXRB	34.07039389	-1.174217909	0.26499702	-4.431060811	9.37706E-06	4.0852E-05
FAM110A	14.95937815	-1.73051484	0.390618852	-4.430187719	9.41511E-06	4.10044E-05
SLC43A2	13.56395696	1.719901076	0.388347471	4.428768571	9.47727E-06	4.12616E-05
TNFRSF9	2.704131773	4.773449875	1.078422503	4.426326288	9.58515E-06	4.17177E-05
PSMB5	51.74230271	0.933986866	0.211109419	4.424183763	9.68076E-06	4.21201E-05
MRPL20	54.30301334	-0.900383532	0.203635421	-4.421546738	9.79969E-06	4.26237E-05
MYCL	78.99805591	-0.863829319	0.195407427	-4.420657547	9.8401E-06	4.27856E-05
LOC101105020	4.674487716	4.382152605	0.991320163	4.420522016	9.84628E-06	4.27985E-05
COPS7A	27.90306435	-1.569085314	0.355089135	-4.418849126	9.92279E-06	4.3117E-05
CEP170	24.25945589	1.448941487	0.327931917	4.418421666	9.94243E-06	4.31883E-05
BCL6B	2.946351483	4.904870636	1.110472951	4.416920404	1.00117E-05	4.34751E-05

MMRN1	7.853665706	2.464053844	0.55802928	4.415635405	1.00714E-05	4.372E-05
SEMA3G	13.79242159	-1.825391247	0.413405954	-4.415493367	1.0078E-05	4.37345E-05
ARMCX3	85.16749676	0.790106219	0.178942677	4.415415207	1.00816E-05	4.37361E-05
NMT1	220.4214226	1.021059966	0.231264868	4.415110589	1.00958E-05	4.37836E-05
CFAP126	13.16949684	1.808158426	0.409622849	4.414203046	1.01383E-05	4.39534E-05
GANAB	76.12261083	-1.03304814	0.234046508	-4.413858384	1.01544E-05	4.40092E-05
RGS10	5.67399402	3.675730026	0.832788731	4.413760527	1.0159E-05	4.40148E-05
ACADS	56.50505021	-0.911851571	0.206611939	-4.413353726	1.01781E-05	4.40833E-05
LMO4	74.84620388	1.033777917	0.23431546	4.411906569	1.02464E-05	4.43647E-05
USP10	71.71647927	0.954026132	0.216294873	4.41076628	1.03005E-05	4.45845E-05
CYP2C18	3.196269369	-4.66815055	1.058426694	-4.410461847	1.0315E-05	4.46328E-05
ABI1	37.71575481	-1.098356503	0.249048311	-4.410214621	1.03268E-05	4.46694E-05
RAB3GAP1	41.60061262	1.17627312	0.266723892	4.410077821	1.03333E-05	4.46831E-05
MYCT1	3.096132179	4.956034584	1.123950045	4.409479412	1.03619E-05	4.47923E-05
ZNF76	35.12398084	-1.217886156	0.276209083	-4.409290758	1.0371E-05	4.48168E-05
LYPLA1	40.2326417	1.015776487	0.230455415	4.40769199	1.04478E-05	4.51342E-05
CCT6A	93.96404253	0.760858531	0.172642388	4.407136267	1.04746E-05	4.52355E-05
ABLIM1	169.087906	-0.650706301	0.147652725	-4.407005015	1.0481E-05	4.52483E-05
LOC101117706	2.660852114	4.759738129	1.080128785	4.406639463	1.04987E-05	4.53101E-05
FABP7	2.849215256	4.872584244	1.105777082	4.406479683	1.05064E-05	4.53288E-05
HADHA	84.75726726	-0.828141019	0.187986677	-4.405317604	1.05629E-05	4.55578E-05
ADCK2	27.36442701	-1.279051885	0.290441834	-4.403814236	1.06364E-05	4.58601E-05
CSE1L	35.82688898	-1.490368379	0.338451762	-4.403488318	1.06524E-05	4.59142E-05
KRIT1	20.23438541	1.38649179	0.314961694	4.402096557	1.0721E-05	4.61949E-05
MBOAT7	65.03081249	-1.342409352	0.304964751	-4.401850867	1.07331E-05	4.62256E-05
RMI2	50.45416333	-1.044183872	0.237216776	-4.401812922	1.0735E-05	4.62256E-05
PDCD1LG2	2.991148227	4.916240233	1.116953174	4.401473892	1.07518E-05	4.6283E-05
FZD9	2.48318179	-4.931821928	1.120530049	-4.401329472	1.0759E-05	4.62989E-05
GTF2I	75.53188194	-0.843706562	0.191763597	-4.39972223	1.0839E-05	4.66281E-05
RNF126	24.26159136	-1.386032544	0.315121042	-4.398413173	1.09045E-05	4.68951E-05
GNG8	3.908207328	4.674585162	1.063883685	4.39388744	1.11341E-05	4.78672E-05
MROH6	2.957821112	4.892872039	1.113865632	4.392695043	1.11954E-05	4.81151E-05
RBMX	123.4193457	-0.841486772	0.191639104	-4.390997206	1.12832E-05	4.84768E-05
SEC61A1	101.1701369	0.983715312	0.224184572	4.387970598	1.14413E-05	4.91403E-05
SPRYD3	35.13675201	-1.312595688	0.299144553	-4.387830821	1.14487E-05	4.91561E-05
INPP5A	20.06465937	1.324923261	0.302106887	4.385610916	1.15661E-05	4.96311E-05
TRAPPC11	24.20142059	1.316181618	0.300114478	4.385598545	1.15667E-05	4.96311E-05
CHCHD4	48.36165347	0.992391318	0.22629375	4.385411954	1.15766E-05	4.96577E-05
WDFY1	35.599171	1.128365171	0.257573543	4.38074951	1.18272E-05	5.07161E-05
ETF1	107.3325298	-0.894500138	0.20429644	-4.378442117	1.19531E-05	5.12395E-05
PUM3	107.7833845	-0.758282078	0.173250894	-4.376785944	1.20442E-05	5.16137E-05
MSL1	58.27501385	0.907731233	0.20745769	4.375500537	1.21154E-05	5.19021E-05

LOC101117129	5.337298422	-4.247425516	0.970898969	-4.374734808	1.2158E-05	5.2068E-05
PLEKHO2	5.91328005	3.225157628	0.737279019	4.374405814	1.21764E-05	5.21298E-05
METTL6	8.044124077	-2.215972788	0.506790355	-4.372563063	1.22796E-05	5.25551E-05
BAG1	42.8046932	-1.121583044	0.256574748	-4.371369558	1.2347E-05	5.28035E-05
FBXL12	26.16914991	-1.182032902	0.270406142	-4.371324155	1.23495E-05	5.28035E-05
TRPA1	3.511023204	4.556936165	1.042447531	4.371381801	1.23463E-05	5.28035E-05
CDC20	12.28454651	-1.97176844	0.451408624	-4.368034496	1.2537E-05	5.35878E-05
FAIM	12.84159203	-1.84697247	0.42309531	-4.365381571	1.26901E-05	5.4225E-05
PIAS3	16.89327609	-2.128259792	0.487571431	-4.365021528	1.2711E-05	5.42971E-05
ACSL5	59.68829585	1.078405084	0.247087527	4.364465901	1.27434E-05	5.44179E-05
PREPL	59.75730053	0.880407719	0.201730865	4.364268806	1.27549E-05	5.44495E-05
MED26	40.35750723	-1.00536228	0.230417403	-4.363221995	1.28161E-05	5.46934E-05
ZDHHC20	149.8440637	0.695072329	0.159317942	4.362800067	1.28408E-05	5.47815E-05
SEMA4A	2.631907597	4.748567013	1.088439984	4.362727466	1.28451E-05	5.47822E-05
TADA2B	23.92976206	1.242375965	0.284822459	4.361931182	1.28919E-05	5.49552E-05
UBE4B	30.45247978	1.061877726	0.243443938	4.361898408	1.28939E-05	5.49552E-05
LOC114117664	2.752310117	4.792791466	1.099043079	4.360876801	1.29542E-05	5.51948E-05
DAPP1	7.538659204	2.428758959	0.556957158	4.360764419	1.29609E-05	5.52056E-05
CHD2	327.6315747	0.828171948	0.189982902	4.359192002	1.30544E-05	5.5586E-05
TAF9	39.49994094	-1.118385756	0.256568527	-4.359013819	1.3065E-05	5.56136E-05
WDFY4	2.720252868	4.812581811	1.10407842	4.358913029	1.3071E-05	5.56215E-05
ARHGAP22	2.740400239	4.805631357	1.102518214	4.358777295	1.30791E-05	5.56383E-05
TSC2	14.14327609	1.59973723	0.367041042	4.358469621	1.30975E-05	5.56988E-05
LOC101114790	3.724275249	4.623639546	1.060982972	4.357882897	1.31327E-05	5.58305E-05
EXOC6	62.78450651	0.879404413	0.201801604	4.3577672	1.31396E-05	5.58423E-05
CREG1	46.88846694	1.083500922	0.248646799	4.35759047	1.31502E-05	5.58696E-05
PAPPA2	2.668629364	4.743130951	1.088680786	4.356769232	1.31996E-05	5.60617E-05
LOC105613004	3.103350208	4.960051054	1.138562054	4.356416972	1.32209E-05	5.61341E-05
NUP43	28.96491832	-1.237295377	0.284074713	-4.355528043	1.32747E-05	5.63446E-05
KLHL25	7.012058914	-2.957344582	0.67905049	-4.355117367	1.32996E-05	5.64324E-05
ACER2	42.39777396	-1.081778777	0.248425731	-4.354535951	1.33349E-05	5.65644E-05
ADAP2	4.833599051	3.94997288	0.907318958	4.35345569	1.34008E-05	5.68259E-05
CDC34	18.91081233	-1.348370691	0.309791055	-4.352516536	1.34584E-05	5.7044E-05
ZNF839	10.86022972	-1.828385607	0.420079287	-4.352477408	1.34608E-05	5.7044E-05
LOC105605780	20.07804254	1.420584085	0.326403578	4.352231959	1.34759E-05	5.70898E-05
NKX2-3	2.789365811	4.818339924	1.107137807	4.352068816	1.34859E-05	5.71142E-05
ERLIN1	25.79571042	-1.499872068	0.344723544	-4.350941775	1.35554E-05	5.73904E-05
RAB17	13.31491559	1.837056139	0.422372287	4.349376592	1.36525E-05	5.77832E-05
ABCD1	5.469344945	3.726556698	0.856819511	4.349290197	1.36579E-05	5.77877E-05
ADIPOQ	4.928814798	4.396600941	1.011414653	4.346981654	1.38024E-05	5.83806E-05
MTA2	16.20886052	-1.588729881	0.365509921	-4.3466122	1.38256E-05	5.84604E-05
SPTY2D1	42.39627974	1.028829433	0.236752319	4.345593891	1.38899E-05	5.87137E-05

SULF1	2.740508089	4.801426681	1.10502814	4.345071864	1.3923E-05	5.88349E-05
PHACTR1	3.505246935	4.551960013	1.047692805	4.344746849	1.39436E-05	5.89035E-05
MUC5AC	14.55490099	4.048586909	0.931935903	4.344276144	1.39736E-05	5.90113E-05
DENND11	19.06237633	-1.457886736	0.335736152	-4.342358513	1.40961E-05	5.95101E-05
THUMPD3	68.77792713	-0.826544682	0.190388126	-4.341366752	1.41599E-05	5.97605E-05
FBXO7	14.18927969	-1.787856711	0.411836647	-4.341179255	1.4172E-05	5.97926E-05
BLTP2	56.70972297	0.987296271	0.227453226	4.34065627	1.42058E-05	5.99162E-05
MTRF1	16.99063866	-1.452932975	0.334788899	-4.339848121	1.42581E-05	6.0118E-05
CCSAP	26.75869803	-1.291692086	0.297672708	-4.339303041	1.42935E-05	6.02293E-05
MACF1	217.3956288	-0.605382404	0.139510907	-4.33931954	1.42925E-05	6.02293E-05
TRUB1	20.624919	-1.283843567	0.295979053	-4.337616316	1.44036E-05	6.06741E-05
LOC121817120	7.011861406	-2.484822924	0.57296654	-4.336767947	1.44593E-05	6.08895E-05
ANAPC4	34.52668087	-1.073664235	0.247586569	-4.33652051	1.44756E-05	6.09384E-05
WDR37	59.90483956	-1.029583071	0.237425179	-4.33645276	1.44801E-05	6.09384E-05
ADAMTS12	2.744605268	4.807190416	1.108637077	4.336126325	1.45016E-05	6.10097E-05
GLYCTK	5.197217219	3.660876124	0.844316784	4.335903531	1.45163E-05	6.10523E-05
MEST	8.575502773	2.332595237	0.538132462	4.334611645	1.46018E-05	6.13926E-05
ITGB6	9.845715426	-2.310710852	0.533207276	-4.333607133	1.46686E-05	6.16542E-05
N4BP2L1	14.82978545	1.644490941	0.379488816	4.333437165	1.46799E-05	6.16824E-05
ABO	6.912719227	2.807962717	0.648096479	4.332630726	1.47338E-05	6.18894E-05
LOC101105609	2.688565718	4.787611417	1.105368426	4.331235905	1.48275E-05	6.22633E-05
ZMYND19	8.977684444	-2.239438805	0.517122132	-4.330580081	1.48717E-05	6.24294E-05
CAPN10	6.951001961	-2.506874754	0.578936547	-4.330137328	1.49016E-05	6.25354E-05
TINAGL1	5.58448628	-3.19170702	0.737398958	-4.328331341	1.50243E-05	6.30305E-05
IMP4	57.76365026	-1.069291583	0.247060937	-4.328047955	1.50437E-05	6.30918E-05
BCL2A1	3.731583562	4.57021934	1.056225308	4.326936028	1.51198E-05	6.33912E-05
ST6GALNAC3	12.29832969	-1.757421812	0.406224351	-4.326234523	1.5168E-05	6.35733E-05
B4GALT1	207.0835804	-0.958944973	0.221682927	-4.325750244	1.52014E-05	6.36932E-05
LOC132659522	13.8055157	1.725950604	0.399038078	4.325277964	1.5234E-05	6.38098E-05
FKBP10	4.420051246	4.334779501	1.002256095	4.325021843	1.52517E-05	6.3864E-05
LGMN	24.41196266	1.296600436	0.299797876	4.324915345	1.5259E-05	6.38748E-05
GGPS1	8.937163369	2.142137324	0.49541805	4.323898423	1.53296E-05	6.41501E-05
ERCC5	34.18463394	1.198071059	0.277239163	4.321435136	1.55018E-05	6.48503E-05
TANK	22.83912706	1.312866453	0.30381364	4.32128871	1.55121E-05	6.4873E-05
CPNE2	11.882001	1.985053923	0.459380805	4.321151209	1.55217E-05	6.48931E-05
TCAF1	183.1521259	-0.837685161	0.193887722	-4.320465219	1.55701E-05	6.50749E-05
BAG6	7.7247282	-2.438239161	0.564377453	-4.320227797	1.55868E-05	6.51246E-05
PPP1R7	23.79213568	1.435405972	0.332315409	4.319408416	1.56448E-05	6.53464E-05
ZSCAN2	6.173151961	-2.797253198	0.647648848	-4.319089285	1.56674E-05	6.54205E-05
GOLGA4	591.5249252	0.66476797	0.153938929	4.318387655	1.57173E-05	6.56083E-05
ANKRD13A	25.43374194	-1.233856839	0.28574117	-4.318092622	1.57383E-05	6.5655E-05
HSPH1	47.29762886	1.133283614	0.262447795	4.318129679	1.57357E-05	6.5655E-05

LOC114109056	2.505393205	-4.840242234	1.121082052	-4.317473665	1.57825E-05	6.58188E-05
LOC101105425	35.37677117	1.019540546	0.236199512	4.316437986	1.58567E-05	6.61061E-05
PTPRM	57.2407645	-0.905135964	0.209698214	-4.316374221	1.58613E-05	6.61061E-05
FBXO30	16.09903062	1.454990181	0.337094331	4.316270097	1.58688E-05	6.61166E-05
LRRC47	18.24632936	-1.368691428	0.317176008	-4.315242626	1.59428E-05	6.64042E-05
LOC101120797	44.58700726	1.050971548	0.243566579	4.314925106	1.59657E-05	6.64552E-05
METTL17	66.11524386	-0.803396898	0.186190549	-4.314917715	1.59662E-05	6.64552E-05
RAB1B	45.99710587	1.05854365	0.245328674	4.314797909	1.59749E-05	6.64552E-05
TRIP11	125.3331039	0.737683602	0.170965222	4.314816748	1.59735E-05	6.64552E-05
NTN1	2.596612024	4.738983773	1.098446571	4.314259701	1.60139E-05	6.65965E-05
VIPR2	6.413067906	-2.623666925	0.608405992	-4.312362073	1.6152E-05	6.715E-05
RAP1GDS1	18.94068372	-1.334241951	0.309500673	-4.310950085	1.62555E-05	6.75592E-05
LOC132658513	2.827391718	4.874652072	1.130892699	4.310446142	1.62926E-05	6.76713E-05
SARDH	8.210648391	2.514477933	0.583344891	4.310448197	1.62924E-05	6.76713E-05
LPL	20.21444593	1.306465342	0.303124862	4.309990726	1.63261E-05	6.77897E-05
PPP1R14D	48.31844631	0.962710513	0.223372072	4.309896516	1.63331E-05	6.77976E-05
CFAP410	9.348448314	-2.08540919	0.483909516	-4.309502341	1.63622E-05	6.78974E-05
PRPF39	98.4754304	0.906395092	0.210332295	4.309348172	1.63736E-05	6.79237E-05
CPE	91.72172119	1.083752538	0.251493296	4.30927009	1.63794E-05	6.79266E-05
TPST2	19.86275662	1.505220991	0.349376345	4.308308257	1.64508E-05	6.82014E-05
TMEM74	3.845693222	4.660890475	1.081980142	4.307741236	1.6493E-05	6.83552E-05
KCTD18	8.230216121	2.161659889	0.501823062	4.307613683	1.65025E-05	6.83735E-05
GPR65	2.618165195	4.744875134	1.101832257	4.306349813	1.65971E-05	6.87225E-05
RASA1	69.09227646	-0.841974315	0.195516756	-4.306404885	1.65929E-05	6.87225E-05
BRI3BP	146.3852724	0.768293151	0.178416128	4.306186654	1.66093E-05	6.87519E-05
TOR4A	16.21273367	1.649498152	0.383119274	4.305442888	1.66652E-05	6.89619E-05
NR4A3	3.492661356	4.566452808	1.060790367	4.304764587	1.67163E-05	6.91522E-05
EIF1AX	72.33839857	-0.782639682	0.181812552	-4.304651541	1.67249E-05	6.91661E-05
RNF19A	60.52885411	0.935567625	0.217425215	4.302939862	1.68547E-05	6.96812E-05
BRSK2	5.845184273	-3.129062367	0.727330864	-4.30211685	1.69174E-05	6.9919E-05
ATG2A	34.48744172	-1.148533773	0.26698387	-4.301884506	1.69352E-05	6.99707E-05
LOC121820612	2.945928194	4.893397744	1.137637424	4.30136847	1.69747E-05	7.01122E-05
SLC26A9	2.642154744	-5.102605033	1.18719303	-4.298041603	1.72314E-05	7.11507E-05
POFUT2	15.84483385	-1.622771899	0.377594984	-4.297652166	1.72617E-05	7.12537E-05
RPL39	18.11613789	-1.457755169	0.339226949	-4.297285855	1.72902E-05	7.13495E-05
N4BP3	7.150566163	-2.374207803	0.552556343	-4.296770516	1.73304E-05	7.14934E-05
TBC1D8	17.78686639	1.453622065	0.338342554	4.296302816	1.7367E-05	7.16223E-05
SEC23B	83.82210554	-0.917288233	0.213538369	-4.295660023	1.74174E-05	7.1808E-05
PDCD10	58.68320495	-0.892708824	0.207825112	-4.295481019	1.74315E-05	7.18438E-05
SPCS3	72.72963739	1.05463374	0.245539006	4.295178014	1.74553E-05	7.19198E-05
MFSD13A	5.906567536	-3.073354573	0.715628469	-4.294623128	1.7499E-05	7.20777E-05
NDRG4	5.621384058	3.140760697	0.73137042	4.294350183	1.75206E-05	7.21442E-05

CD63	405.6499637	-0.678579571	0.158043076	-4.29363682	1.7577E-05	7.23319E-05
TIMP4	9.455521141	2.123551871	0.494576069	4.293681001	1.75735E-05	7.23319E-05
LOC105613737	12.21801557	1.734621733	0.404006534	4.293548708	1.7584E-05	7.23384E-05
BACH2	6.074141246	2.925652943	0.681511557	4.29288823	1.76364E-05	7.25316E-05
LRRC4C	3.673790833	4.585364951	1.068880675	4.289875435	1.78773E-05	7.34774E-05
PPP1R3B	27.53345117	-1.174084426	0.273683966	-4.289927703	1.78731E-05	7.34774E-05
MAP1LC3A	52.23493943	1.016021624	0.236847323	4.289774566	1.78855E-05	7.34882E-05
PRICKLE1	6.522444794	3.048688386	0.710719239	4.289581907	1.7901E-05	7.35293E-05
MROH1	35.32590405	1.15213316	0.268615367	4.289155801	1.79354E-05	7.36479E-05
DOK6	2.55617473	4.703975298	1.096772603	4.288924874	1.7954E-05	7.37019E-05
ZFAND6	113.1221671	0.635424681	0.148158691	4.2888114	1.79632E-05	7.37169E-05
BCAT1	8.104648065	2.285823587	0.533213574	4.28688184	1.81199E-05	7.43142E-05
PHLDB2	24.81215154	1.274775115	0.297364741	4.286907422	1.81178E-05	7.43142E-05
LSR	20.1695134	-1.9086979	0.445256071	-4.286742004	1.81313E-05	7.43359E-05
PUM2	100.0945719	0.70705616	0.164942575	4.286680736	1.81363E-05	7.43359E-05
KEF53_p07	2132.784336	2.143078739	0.500058203	4.285658602	1.82199E-05	7.46556E-05
TLE5	319.3028119	0.764246675	0.178337984	4.285383624	1.82424E-05	7.47251E-05
RPAP3	87.49720892	-0.758996129	0.177139836	-4.284728649	1.82962E-05	7.49226E-05
NSFL1C	19.91470813	-1.485574589	0.346885072	-4.282613203	1.84711E-05	7.56156E-05
CSF1R	2.79843833	4.837109534	1.129509088	4.282488371	1.84815E-05	7.56305E-05
SGCD	2.614629174	4.760439018	1.111620202	4.282432985	1.84861E-05	7.56305E-05
SDHAF2	45.38897511	-1.239242744	0.289515679	-4.280399412	1.86558E-05	7.63016E-05
PVRIG	2.682956818	4.750729121	1.110157459	4.279329102	1.87458E-05	7.6646E-05
FRG1	93.40983065	-1.039653105	0.24296212	-4.279074884	1.87672E-05	7.67101E-05
PHF14	283.0124483	0.814991621	0.19047217	4.278796323	1.87907E-05	7.67827E-05
ATXN7	34.82860894	1.000420074	0.233901047	4.277108152	1.89337E-05	7.73434E-05
NDUFB10	49.16488312	0.925431635	0.216374942	4.27698156	1.89444E-05	7.73637E-05
PLA2G12B	5.940812448	3.483510177	0.814494554	4.276898057	1.89516E-05	7.73691E-05
UBE2E2	18.47710998	1.555810831	0.363903675	4.275336958	1.90848E-05	7.78895E-05
KCNK2	2.503396581	4.667516951	1.091784046	4.275128372	1.91027E-05	7.79386E-05
ZCWPW2	5.60782832	3.297525049	0.771373534	4.274874499	1.91245E-05	7.80037E-05
DPYD	194.7347601	0.894180761	0.209176122	4.27477455	1.91331E-05	7.80149E-05
CIB2	6.456897253	2.711229341	0.634292979	4.274411717	1.91643E-05	7.81182E-05
LOC114112144	3.518688555	5.137237134	1.201950593	4.274083448	1.91925E-05	7.82095E-05
SPIDR	12.56528443	-1.796662438	0.420369819	-4.274004359	1.91993E-05	7.82134E-05
SLC52A2	45.97868795	-0.96451631	0.22570469	-4.273355207	1.92553E-05	7.84177E-05
FCHSD2	16.7207276	1.49057976	0.348846355	4.272883284	1.92961E-05	7.85599E-05
GPR55	2.862673413	4.84669861	1.134391362	4.272510152	1.93285E-05	7.86676E-05
CD40LG	2.586867816	4.716118416	1.103884948	4.272291622	1.93474E-05	7.87208E-05
KDM1B	30.25705403	1.149028329	0.268954683	4.272200491	1.93553E-05	7.8729E-05
PSMC4	10.95536328	-1.769499982	0.41432922	-4.270758361	1.94809E-05	7.92158E-05
KATNAL1	31.56170482	1.088992348	0.255063887	4.269488569	1.95922E-05	7.96196E-05

MFSD14A	39.82820686	1.067811489	0.250102642	4.26949304	1.95918E-05	7.96196E-05
KPNA5	17.64828613	1.583539651	0.370904286	4.269402405	1.95997E-05	7.96262E-05
DDHD1	25.44454603	-1.256937722	0.294417118	-4.269241312	1.96139E-05	7.96595E-05
LAMP3	2.576041364	4.730155732	1.108428627	4.26744277	1.97726E-05	8.02798E-05
LMO3	2.837546837	4.823483967	1.130351009	4.267244358	1.97902E-05	8.03268E-05
RPS6KL1	2.720959576	4.779532785	1.12022052	4.266599925	1.98475E-05	8.05347E-05
YOD1	30.58763538	1.115677018	0.261522524	4.266083861	1.98934E-05	8.06966E-05
STX19	12.27074871	-1.70277769	0.39941247	-4.263206127	2.01514E-05	8.17185E-05
STXBP6	14.14277266	1.791809871	0.420304388	4.263124348	2.01588E-05	8.17236E-05
SOCS2	24.54878647	1.237009769	0.290226428	4.262223043	2.02403E-05	8.20292E-05
NACA	101.5160729	-2.261069449	0.530646363	-4.260972288	2.03539E-05	8.24646E-05
LOC121819958	5.763937871	3.016775806	0.708095039	4.260410875	2.04051E-05	8.26456E-05
PODN	2.672104536	4.744487557	1.113638748	4.26034705	2.0411E-05	8.26456E-05
RIOX1	45.70826253	-1.145973729	0.269019151	-4.259822113	2.0459E-05	8.28148E-05
LOC114108666	6.366744133	2.979291022	0.699417089	4.259677192	2.04722E-05	8.28434E-05
CBX6	45.84100406	1.01296855	0.237814608	4.259488344	2.04895E-05	8.28884E-05
GUK1	28.58461729	1.438317454	0.337708457	4.25905074	2.05297E-05	8.30257E-05
CCDC163	9.055021635	-1.992207334	0.467845625	-4.258257913	2.06026E-05	8.32954E-05
ZBTB8A	20.70581871	1.410449391	0.331243583	4.258042911	2.06224E-05	8.33503E-05
SC5D	69.05422775	0.852159329	0.200140999	4.257794924	2.06453E-05	8.34176E-05
PHYHD1	2.696978458	4.774118852	1.12150073	4.256902135	2.07279E-05	8.37259E-05
TEAD2	14.74313956	-1.682133676	0.395284144	-4.255505061	2.08578E-05	8.4225E-05
MPG	16.11811227	-1.477496688	0.347205857	-4.255391031	2.08684E-05	8.42425E-05
EZR	90.63516273	0.796809881	0.187252229	4.255275817	2.08791E-05	8.42605E-05
HACL1	39.61794919	1.180881681	0.277544585	4.254745882	2.09287E-05	8.44093E-05
LOC132657741	2.943658929	4.927200429	1.158039442	4.254777729	2.09257E-05	8.44093E-05
GK	3.38598736	4.494497014	1.056532853	4.254005922	2.0998E-05	8.46633E-05
CNEP1R1	8.19903437	2.102407607	0.4942834	4.253445705	2.10506E-05	8.48243E-05
LOC114108620	46.34224283	1.047564274	0.246282866	4.253500419	2.10455E-05	8.48243E-05
SIRT2	42.23192167	1.003171565	0.2359483	4.251658378	2.12193E-05	8.54785E-05
TOE1	39.83775828	-0.926737631	0.217993327	-4.251220185	2.12609E-05	8.56201E-05
NHSL2	5.305246576	3.565655331	0.838842535	4.250684942	2.13118E-05	8.57992E-05
LRRC19	11.72690307	1.979324569	0.465709017	4.250131517	2.13645E-05	8.59855E-05
SREK1IP1	104.919127	0.934941462	0.219990633	4.249914876	2.13852E-05	8.60428E-05
RAB4A	30.32527623	-1.051945299	0.247558266	-4.249283682	2.14455E-05	8.62596E-05
ACSL4	78.23926491	0.788351256	0.18563496	4.246782274	2.16863E-05	8.72017E-05
PDIA3	370.71205	0.614384977	0.14474883	4.244490102	2.19091E-05	8.80713E-05
CBLIF	9.717300948	2.021913733	0.476453536	4.243674528	2.19889E-05	8.83656E-05
PABPC1	1359.371379	-0.753819021	0.177708474	-4.241885633	2.2165E-05	8.90463E-05
FRAT2	17.17098021	1.487065741	0.350604592	4.241432589	2.22098E-05	8.91994E-05
ZMYM3	21.42448264	-1.228423529	0.289699238	-4.240340909	2.23181E-05	8.96074E-05
ZDHHC18	14.05018316	-1.562843243	0.368593028	-4.240023891	2.23496E-05	8.97071E-05

APOLD1	4.134958365	4.20271991	0.991444423	4.238986888	2.24531E-05	9.00954E-05
C20H6orf47	36.28135148	-1.303994781	0.307639969	-4.238704042	2.24814E-05	9.01819E-05
TEX14	18.8237791	1.574485431	0.371487095	4.238331434	2.25187E-05	9.03046E-05
VPS11	5.898875128	-2.879879683	0.679521578	-4.238098943	2.2542E-05	9.0371E-05
TIGD5	8.883461728	-2.110058006	0.497946675	-4.23751801	2.26004E-05	9.05779E-05
GADD45G	12.92840568	-1.715833112	0.404998815	-4.236637361	2.26892E-05	9.08949E-05
RITA1	10.1339177	-2.196761426	0.518520076	-4.236598595	2.26931E-05	9.08949E-05
TBCEL	47.75703664	1.07125092	0.252866249	4.236432989	2.27099E-05	9.09347E-05
RABGAP1	52.88909739	-0.88276233	0.208381404	-4.236281712	2.27252E-05	9.09687E-05
IDI1	69.74675206	-0.936256311	0.22103129	-4.235854177	2.27685E-05	9.11147E-05
PRDM4	16.03309766	-1.711101574	0.404122687	-4.234114121	2.29454E-05	9.17955E-05
CCDC102A	9.417414814	1.96472435	0.464070878	4.233673005	2.29905E-05	9.19483E-05
PSMD7	163.3835604	0.837314307	0.197807312	4.232979544	2.30615E-05	9.22048E-05
LOC114116333	4.019800222	4.178707596	0.987226863	4.232773389	2.30827E-05	9.22618E-05
ATRX	715.4887689	0.90379691	0.21359905	4.231277763	2.32368E-05	9.28497E-05
PDZD2	2.515484782	4.672474201	1.104454286	4.230572747	2.33097E-05	9.31134E-05
ACE	3.666443994	4.613771396	1.091528752	4.226889477	2.36944E-05	9.46218E-05
PAFAH1B1	155.232892	-0.692700823	0.163910674	-4.226087323	2.3779E-05	9.49312E-05
NCOA2	108.3696806	0.814852117	0.192869746	4.224883037	2.39065E-05	9.54118E-05
ACTR6	19.61812123	1.380555871	0.326777608	4.224756647	2.39199E-05	9.54344E-05
AOC1	4.085646443	4.204056243	0.995114657	4.224695331	2.39264E-05	9.54344E-05
OVOL1	52.71147096	-0.949524704	0.224810641	-4.223664413	2.40362E-05	9.58436E-05
CDK3	8.910601466	2.085264871	0.493726203	4.223524819	2.40511E-05	9.58744E-05
RPS24	824.9968133	-0.617928468	0.146334254	-4.222719218	2.41372E-05	9.61891E-05
LOC101112639	2.552711111	4.720005685	1.118028903	4.22172063	2.42444E-05	9.65876E-05
B9D1	8.064453678	-2.355173035	0.557950774	-4.22111259	2.43099E-05	9.68197E-05
HSPB1	102.9938476	0.882107885	0.209023764	4.220132038	2.44159E-05	9.72128E-05
B3GNTL1	18.22725993	1.427867115	0.338397015	4.219502697	2.44842E-05	9.74555E-05
RFXANK	17.29925616	1.476402348	0.349961633	4.218754877	2.45655E-05	9.77501E-05
LSM2	79.99515972	-0.757720421	0.17964906	-4.217781165	2.46718E-05	9.81439E-05
CEP43	7.284719214	-2.470128959	0.58571803	-4.217266387	2.47282E-05	9.83388E-05
COL17A1	18.04379148	1.63172285	0.386987273	4.216476783	2.48149E-05	9.86543E-05
MYC	12.52161163	1.797596902	0.426368099	4.216068007	2.48599E-05	9.88038E-05
EIF4A3	61.68846011	-0.935049937	0.221812394	-4.215499059	2.49226E-05	9.90238E-05
RAN	11.92398877	-2.680355559	0.636110206	-4.213665387	2.51259E-05	9.98019E-05
NEURL3	8.690892494	-2.228460485	0.528927162	-4.213170818	2.5181E-05	9.99911E-05
OLFML3	4.752802293	3.816565503	0.905892072	4.213046586	2.51949E-05	0.000100016
IPO9	40.89715764	0.938108081	0.222682259	4.212765235	2.52263E-05	0.000100111
PPIF	17.49653138	-1.520301219	0.361063426	-4.210620929	2.5467E-05	0.000101036
TMEM184A	17.93957071	1.545245841	0.367007256	4.210395883	2.54924E-05	0.000101107
ACO2	27.0896202	1.120021678	0.26601937	4.210301225	2.5503E-05	0.000101119
METTL4	12.12610974	1.743925	0.414310358	4.209223752	2.5625E-05	0.000101573

ASB2	2.555536917	4.667327959	1.109103272	4.208199612	2.57413E-05	0.000102004
PDGFD	2.416204456	4.615466518	1.096805436	4.208099602	2.57527E-05	0.000102019
BMAL2	2.60632772	4.754920627	1.130357296	4.206564282	2.59282E-05	0.000102683
TMEM79	38.03320946	-1.2022475	0.285878918	-4.20544302	2.60571E-05	0.000103163
SDR42E1	29.43833695	-1.090630149	0.259416115	-4.204172699	2.62039E-05	0.000103714
MSANTD4	26.86455986	1.194025461	0.284156854	4.201994235	2.64574E-05	0.000104686
TOMM5	42.72830363	-0.964466569	0.229550531	-4.201543614	2.65101E-05	0.000104864
POLR2L	59.09862589	0.774621651	0.184373014	4.201383034	2.65289E-05	0.000104907
SPC24	26.11281986	-1.25460552	0.298753649	-4.199465087	2.67546E-05	0.000105768
FANCM	22.13483008	1.216432095	0.289673608	4.199319721	2.67718E-05	0.000105802
MED29	13.94671474	1.580143179	0.376290956	4.199258985	2.6779E-05	0.000105802
LOC101115555	2.407235176	4.632877289	1.103350021	4.198918931	2.68192E-05	0.00010593
CD200	19.24816166	1.383218706	0.329470054	4.198313895	2.6891E-05	0.000106182
ADAM28	13.21006716	-1.995308064	0.475273093	-4.198234854	2.69004E-05	0.000106187
CACNA1D	24.66727491	1.468785192	0.349898206	4.197749989	2.6958E-05	0.000106383
LOC132657452	7.590881475	2.318624529	0.552597449	4.195865423	2.71832E-05	0.00010724
HSPG2	24.57882584	1.41356638	0.33693299	4.19539321	2.72399E-05	0.000107403
LOC114109492	4.375587673	-3.97317497	0.947033992	-4.195387921	2.72405E-05	0.000107403
VAT1	44.00841348	-0.961791317	0.229264639	-4.195114089	2.72734E-05	0.000107501
PYM1	6.392029339	-2.63997124	0.629357738	-4.19470689	2.73225E-05	0.000107663
SNRK	29.57838596	1.062722605	0.253408081	4.193720275	2.74416E-05	0.0001081
CNOT9	27.84749912	-1.160547913	0.276754212	-4.193424571	2.74774E-05	0.00010821
SMARCD3	4.833867606	3.949837686	0.941988908	4.193083008	2.75189E-05	0.000108341
RASGEF1A	6.008103848	-2.699942904	0.644013354	-4.192370995	2.76054E-05	0.00010865
PSMG3	13.45508767	-1.740063431	0.415069894	-4.192217883	2.7624E-05	0.000108691
PDPN	2.706871723	4.801561466	1.145751498	4.190752947	2.7803E-05	0.000109363
EBPL	10.33619292	-1.909445521	0.455641339	-4.190676649	2.78124E-05	0.000109368
PMP22	4.410589646	3.844306375	0.917386216	4.190499384	2.78341E-05	0.000109421
PIGG	32.27020023	1.017460746	0.242930918	4.188271943	2.81087E-05	0.000110468
PROM1	74.91650017	-0.935038064	0.223298428	-4.187392065	2.82178E-05	0.000110864
CFAP69	25.94616394	-1.164414041	0.278085612	-4.187250216	2.82354E-05	0.000110875
STAT1	18.7974034	1.286309745	0.30719787	4.187235228	2.82373E-05	0.000110875
ATF5	277.9318724	-0.854972187	0.204190087	-4.187138555	2.82493E-05	0.00011089
TMTC2	30.03933942	1.295038178	0.309321085	4.186711617	2.83025E-05	0.000111066
EMG1	43.30369141	-0.929445122	0.222107269	-4.184667736	2.85584E-05	0.000112037
MLH1	10.33036614	-2.041971719	0.487985318	-4.184494173	2.85802E-05	0.00011209
PTGR2	14.20975763	1.737290024	0.415193788	4.184287135	2.86062E-05	0.000112159
CDC42EP5	5.302033354	3.24311479	0.775140826	4.183903983	2.86545E-05	0.000112304
PPIC	11.64055437	1.789674072	0.427756596	4.183860842	2.866E-05	0.000112304
RGS7BP	2.491405431	4.645127747	1.11027162	4.183775991	2.86707E-05	0.000112313
EGFLAM	3.25120431	4.457029059	1.065477995	4.183126335	2.87528E-05	0.000112602
LOC101120367	2.585957707	4.718079468	1.128132643	4.182202774	2.88698E-05	0.000113027

KIAA0319L	116.5205259	-0.991811754	0.237193167	-4.181451617	2.89654E-05	0.000113368
ATP6V0A1	51.29456181	0.875507378	0.209382691	4.181374182	2.89753E-05	0.000113373
SCAF8	93.83963711	0.732992241	0.175310272	4.181114043	2.90084E-05	0.00011347
FITM2	21.98556693	1.361981344	0.325802696	4.180386964	2.91013E-05	0.0001138
ARHGEF39	19.13103104	-1.28918822	0.308484489	-4.179102242	2.92662E-05	0.000114314
GPKOW	24.00252726	-1.331523982	0.318608638	-4.179183554	2.92557E-05	0.000114314
HIF1AN	84.25807587	-0.832842544	0.19928615	-4.179129072	2.92628E-05	0.000114314
STK25	38.73906216	-1.024095726	0.24505199	-4.179095728	2.9267E-05	0.000114314
TFPI	7.905691969	2.27829098	0.545384211	4.177405458	2.94853E-05	0.000115133
SEMA5A	31.413121	1.088517825	0.260632903	4.176440546	2.96106E-05	0.000115589
PBRM1	201.7570568	0.719542506	0.172291827	4.176300866	2.96288E-05	0.000115626
TMEM204	2.531040466	4.679498101	1.120633188	4.175762553	2.96989E-05	0.000115866
PXMP4	65.75206494	0.810892322	0.194229365	4.174921347	2.98089E-05	0.000116261
ERBIN	66.40913463	0.77232977	0.185032701	4.174017699	2.99274E-05	0.000116689
LOC101121719	3.465118892	4.501304588	1.078582311	4.173352876	3.0015E-05	0.000116996
ATP6V1B2	31.09623536	-1.139322924	0.273065169	-4.172348051	3.01477E-05	0.000117479
LOC114109375	9.103760533	2.548136916	0.610762683	4.17205731	3.01862E-05	0.000117595
RFC4	7.49316775	-2.312283869	0.554255139	-4.171876285	3.02102E-05	0.000117654
EIF4E2	126.9093932	-0.96659088	0.23169646	-4.171798219	3.02205E-05	0.00011766
MLXIP	29.01061784	1.084277423	0.259913338	4.171688269	3.02351E-05	0.000117683
NDUFB1	34.66760054	-1.430677272	0.342976589	-4.171355475	3.02793E-05	0.00011782
CDK5RAP3	11.20664203	-1.789493986	0.429208003	-4.169293146	3.05546E-05	0.000118857
ZBED3	2.463761546	4.633483545	1.111447078	4.168874646	3.06107E-05	0.000119041
BRD1	27.18993955	-1.229395292	0.295067836	-4.166483569	3.09334E-05	0.000120261
CASTOR2	9.180670922	-2.1126066	0.507075672	-4.166255095	3.09644E-05	0.000120346
MMP16	3.342566763	4.452081441	1.068688985	4.165928069	3.10088E-05	0.000120484
ARMC7	7.12789663	-2.695037136	0.646936571	-4.16584447	3.10202E-05	0.000120493
NUP35	20.95306382	-1.298545234	0.311800076	-4.164672597	3.118E-05	0.000121078
CERCAM	3.941903331	4.146261019	0.995745827	4.163975292	3.12754E-05	0.000121414
BICC1	4.365549865	3.824523089	0.919023954	4.161505337	3.16157E-05	0.000122685
CTPS2	27.21404113	1.128259528	0.271120803	4.161464255	3.16213E-05	0.000122685
ANKRD10	36.40557524	-1.166979555	0.280444084	-4.161184422	3.16601E-05	0.0001228
ATP6AP2	134.0621962	-0.724733223	0.174172224	-4.161014921	3.16836E-05	0.000122836
CCDC12	26.75063076	-1.18094146	0.283812996	-4.160984446	3.16879E-05	0.000122836
RBM22	54.83228211	0.971569028	0.233532315	4.160319433	3.17803E-05	0.000123159
POLR2F	18.93605095	-1.488482234	0.357803681	-4.160052877	3.18174E-05	0.000123267
TSPYL4	17.59487269	1.454216909	0.349614876	4.159482355	3.1897E-05	0.00012354
DDX54	109.4416959	-1.100218662	0.264513985	-4.159396941	3.19089E-05	0.00012355
LOC105616150	2.384420256	4.604810074	1.107123917	4.159254445	3.19288E-05	0.000123591
PGAM5	39.99811342	1.006245083	0.241969406	4.158563266	3.20256E-05	0.00012393
C6H4orf19	112.1564288	0.649488068	0.156230849	4.157233158	3.22125E-05	0.000124617
MCM8	10.3962221	-1.841708449	0.443089842	-4.156512456	3.23143E-05	0.000124975

PKD2L1	2.308286499	-4.860418753	1.169701599	-4.155263835	3.24912E-05	0.000125623
PCGF6	20.89304432	3.209199982	0.772379482	4.154952401	3.25355E-05	0.000125758
ETV6	272.5821478	0.626938197	0.150945605	4.153404768	3.27565E-05	0.000126555
TCF21	2.499684561	4.69875088	1.1313094	4.15337385	3.27609E-05	0.000126555
PRKAR1A	46.77934866	-0.844425401	0.203369324	-4.152176853	3.29328E-05	0.000127183
LOC105609854	11.24323728	-1.825920154	0.439769817	-4.151990615	3.29596E-05	0.000127249
HUWE1	214.2685219	0.748514959	0.180355122	4.150228452	3.32144E-05	0.000128196
ACADL	63.30586608	0.842348405	0.203000098	4.149497522	3.33206E-05	0.000128496
LOC121817058	2.36146822	4.572925434	1.102041624	4.149503372	3.33197E-05	0.000128496
RINL	2.361395818	4.572892985	1.102035761	4.149496002	3.33208E-05	0.000128496
SKAP2	48.37715875	0.887271509	0.213896224	4.148140116	3.35187E-05	0.000129222
GRSF1	94.88225481	-0.705996703	0.170252898	-4.146752929	3.37224E-05	0.000129969
FNBP1L	73.97988847	-0.831576919	0.200604006	-4.145365465	3.39272E-05	0.000130721
ZFHX4	3.278709466	4.452671766	1.074503068	4.143935834	3.41395E-05	0.000131501
RFK	71.01181893	1.128946427	0.272472794	4.143336342	3.42289E-05	0.000131808
RNF34	18.59197122	1.283036641	0.309712785	4.142666048	3.43292E-05	0.000132155
MAN2C1	11.24344994	-1.721736558	0.415700202	-4.141774651	3.44629E-05	0.000132632
FTO	155.4554752	0.651607454	0.157331087	4.141631924	3.44843E-05	0.000132676
MRPS22	21.79522068	-1.288550068	0.311162866	-4.141079183	3.45676E-05	0.000132958
LGALS3BP	44.09616894	1.04889429	0.25331367	4.140693587	3.46257E-05	0.000133144
SEMA4B	10.95540242	1.730348259	0.41790545	4.140525704	3.46511E-05	0.000133203
FAM199X	26.66783521	1.2384763	0.299121649	4.140376679	3.46736E-05	0.000133244
LOC101110855	7.125838497	2.518808266	0.608360402	4.14032251	3.46818E-05	0.000133244
GPR89A	36.57956972	-1.077428262	0.260254811	-4.139897586	3.47461E-05	0.000133453
SNX5	117.8774205	-0.812869478	0.196370553	-4.139467274	3.48113E-05	0.000133627
ZC3H13	213.3107833	0.940128745	0.227110373	4.13952358	3.48028E-05	0.000133627
TBC1D9	5.883859864	2.845506361	0.687487132	4.138995812	3.48829E-05	0.000133863
PPP2R3A	43.64686164	-0.892638367	0.215682275	-4.138672803	3.49321E-05	0.000134013
LOC101114145	7.800239886	-2.218154795	0.536012531	-4.138251754	3.49962E-05	0.000134221
DPAGT1	27.3385439	1.102184728	0.266351017	4.138090937	3.50208E-05	0.000134238
RPL28	293.6083207	-0.909103021	0.219688363	-4.138148271	3.5012E-05	0.000134238
LOC114111381	2.657796724	4.711699318	1.1386519	4.137962898	3.50403E-05	0.000134274
C1H21orf91	5.999140352	2.647207586	0.639796996	4.137574266	3.50997E-05	0.000134463
EXOC3	50.2221314	-0.987220215	0.238617186	-4.137255281	3.51485E-05	0.000134612
TNFRSF17	2.493803366	4.695888035	1.135094001	4.13700366	3.5187E-05	0.000134721
DARS2	12.04797154	1.783101061	0.431056864	4.136579672	3.52521E-05	0.000134931
SLC7A2	2.520753595	4.704871435	1.137437882	4.136376596	3.52833E-05	0.000135012
ARHGAP32	78.56596748	-0.854165639	0.20659017	-4.134589945	3.55589E-05	0.000136028
SDK1	5.417671499	3.025631711	0.732026942	4.133224528	3.57709E-05	0.000136799
AP3B1	107.2390915	0.82919323	0.200658221	4.132366099	3.59048E-05	0.000137236
LGALS12	2.548561925	4.687433828	1.134323627	4.132360216	3.59057E-05	0.000137236
ERI3	9.816487814	1.925305533	0.465984329	4.131695884	3.60097E-05	0.000137594

RFTN2	2.398613277	4.586973704	1.110359603	4.131070412	3.61078E-05	0.00013793
TMEM106C	32.46369874	1.078147205	0.261077816	4.129600986	3.63393E-05	0.000138775
CCL26	4.374608132	4.275679685	1.035497576	4.129106416	3.64176E-05	0.000139034
ITGB3	2.480516948	4.639123604	1.123628719	4.128697963	3.64823E-05	0.000139241
BRCA2	78.09321174	0.839679668	0.203457557	4.12705077	3.67445E-05	0.000140162
KLF15	4.436246659	3.855461859	0.934191984	4.127055172	3.67438E-05	0.000140162
MFSD11	54.61794936	-1.147716982	0.278137033	-4.126444335	3.68415E-05	0.000140492
LOC101118978	11.39951316	-1.674692315	0.405883095	-4.126045988	3.69054E-05	0.000140695
THNSL2	25.18922414	1.520854712	0.368627775	4.125719264	3.69578E-05	0.000140855
SOWAHA	26.17815321	1.284924612	0.311452853	4.125583052	3.69797E-05	0.000140898
CCNE2	14.70524896	-1.440911634	0.349308329	-4.125042308	3.70667E-05	0.000141189
ST6GALNAC1	4.117972996	4.155048446	1.007367306	4.124660808	3.71282E-05	0.000141383
TET1	21.6041894	1.380483209	0.334748328	4.123943554	3.7244E-05	0.000141784
PTPMT1	98.6667573	0.921568714	0.223481814	4.123685483	3.72858E-05	0.000141903
CCDC115	24.50426121	-1.349727669	0.327401059	-4.122551329	3.74699E-05	0.000142563
LOC105612674	4.862243681	3.517583946	0.853268434	4.122482219	3.74812E-05	0.000142565
PPP1R14B	39.6805013	-1.106245848	0.268392202	-4.121751082	3.76003E-05	0.000142978
STYX	37.77880064	0.994453841	0.241408283	4.119385747	3.79884E-05	0.000144412
RWDD4	131.4445507	-0.762174741	0.185054699	-4.118645692	3.81106E-05	0.000144835
SLC9A3	15.68643502	2.285716617	0.555002163	4.118392268	3.81525E-05	0.000144953
LRRN4	2.781400007	-4.471156058	1.085757108	-4.118007634	3.82162E-05	0.000145084
UBASH3A	2.402464737	4.59151502	1.114989689	4.117988771	3.82193E-05	0.000145084
ZFYVE21	20.03837322	-1.28467053	0.311957359	-4.118096573	3.82015E-05	0.000145084
THBS2	2.657030666	4.710213768	1.143917174	4.117617845	3.82809E-05	0.000145276
GKAP1	75.49605582	0.918843815	0.223164438	4.117339768	3.83271E-05	0.00014541
SIRT7	59.13888656	0.839671445	0.203976393	4.116512859	3.84648E-05	0.000145891
EZH2	165.7297091	-0.996647092	0.242241921	-4.114263487	3.88418E-05	0.000147237
ORMDL2	18.81669383	-1.28963595	0.313451058	-4.114313603	3.88333E-05	0.000147237
SCARF1	6.935425956	2.326804334	0.565630715	4.113645654	3.89459E-05	0.00014759
LOC132660073	19.60300008	2.216871069	0.538927643	4.113485545	3.89729E-05	0.000147651
KLRG2	5.120300645	-2.843144252	0.691190778	-4.113400152	3.89874E-05	0.000147664
LOC105605903	2.565367405	4.735680587	1.15142195	4.112897609	3.90724E-05	0.000147944
FBLN2	2.365600847	4.575372847	1.11318291	4.110171658	3.95365E-05	0.000149656
MYH10	23.78970541	1.358557285	0.330540329	4.110110519	3.9547E-05	0.000149656
ATRIP	8.615563539	-2.142737133	0.521633614	-4.107743585	3.99543E-05	0.000151155
SORT1	39.27886372	0.942609133	0.229484496	4.107506826	3.99953E-05	0.000151267
FAM3C	106.7929896	0.920415414	0.224196563	4.105394854	4.03625E-05	0.000152569
LOC121820181	2.923443337	4.883088162	1.189429754	4.105402731	4.03611E-05	0.000152569
LOC114113596	12.38820528	-1.617418763	0.393997662	-4.105148123	4.04056E-05	0.000152689
USHBP1	2.391602084	4.607665545	1.122571325	4.104563732	4.05079E-05	0.000153032
LOC121817234	44.80106257	0.955421695	0.232808425	4.103896566	4.06249E-05	0.000153431
STUM	2.496668055	4.627403354	1.127833129	4.102914902	4.07977E-05	0.00015404

NEFM	4.828250301	3.842882274	0.936733261	4.10242962	4.08834E-05	0.00015432
PXDC1	7.892799046	-2.108273513	0.514015367	-4.101576819	4.10344E-05	0.000154846
ZNF19	8.701611543	2.265668228	0.552425663	4.101308789	4.1082E-05	0.000154982
TRRAP	32.76880517	1.015017277	0.24753981	4.100420368	4.124E-05	0.000155535
WASHC3	13.06752662	1.541812123	0.376097564	4.09950042	4.14043E-05	0.00015611
RPS6KA1	27.76070555	1.274334111	0.310858648	4.099400548	4.14222E-05	0.000156133
TOR1AIP1	21.77883759	1.184976121	0.289071036	4.099255794	4.14481E-05	0.000156187
FXYD6	4.825405048	3.443244009	0.84010397	4.098592714	4.1567E-05	0.000156591
MYRFL	45.63031194	0.915869743	0.223471875	4.098366933	4.16075E-05	0.000156699
POLR3B	57.74347433	-0.860726465	0.21003567	-4.098001377	4.16733E-05	0.000156903
RAF1	8.314448299	-2.194112581	0.5357344	-4.095523044	4.21216E-05	0.000158546
CTNNA1	18.57565583	-1.357784905	0.331615669	-4.094453409	4.23165E-05	0.0001592
ZFX	67.28119203	0.760623842	0.185769976	4.094439037	4.23192E-05	0.0001592
TMEM186	16.66946439	-1.518184399	0.370825553	-4.094066299	4.23873E-05	0.000159412
FOXO1	8.716792625	2.047715022	0.500266937	4.093244765	4.25378E-05	0.000159933
POLR2B	65.42633529	-0.934263953	0.22825343	-4.093099289	4.25645E-05	0.000159988
LOC101113745	7.844867974	2.144942651	0.52417336	4.092048195	4.2758E-05	0.00016067
HSD17B10	18.50781117	1.311758996	0.320594692	4.091642901	4.28328E-05	0.000160906
LOC101120999	2.421175543	4.620459456	1.129497749	4.090720374	4.30035E-05	0.000161474
MICAL1	18.01112776	1.358464307	0.332086448	4.090694802	4.30083E-05	0.000161474
ARMCX2	2.500843071	4.68155296	1.144641108	4.089974515	4.31421E-05	0.000161931
LOC105612031	2.303220612	4.530203357	1.108160966	4.088037294	4.35038E-05	0.000163243
BNIP2	23.20753732	1.224200565	0.299489352	4.08762635	4.35809E-05	0.000163487
DNAJA4	6.644868032	2.370550657	0.580056568	4.086757722	4.37444E-05	0.000164054
ANGPTL1	2.364553623	4.575144997	1.119678665	4.086123223	4.38641E-05	0.000164456
B4GALNT2	27.0186984	1.363380048	0.333689058	4.08577991	4.3929E-05	0.000164608
OSTF1	81.28749474	0.804834191	0.196981728	4.085831713	4.39192E-05	0.000164608
CLDN18	17.2913606	-2.037384156	0.498709872	-4.085309457	4.40181E-05	0.000164895
ICE2	36.57498569	1.003708961	0.24570318	4.085046677	4.4068E-05	0.000165036
LOC105610887	5.562969056	2.751562112	0.673609989	4.084800043	4.41148E-05	0.000165165
FBXO48	2.452232568	4.610039288	1.128669066	4.084491572	4.41734E-05	0.000165338
NECAP2	54.1195424	0.896310444	0.219553598	4.082422024	4.45688E-05	0.000166771
DIABLO	19.19675945	-1.464022655	0.358655251	-4.081977478	4.46541E-05	0.000167044
GPR35	6.079597674	2.816177508	0.690076928	4.080961695	4.48497E-05	0.000167729
RPA3	15.53358169	-1.53678444	0.376609798	-4.080574764	4.49245E-05	0.000167961
BNIP3L	13.72694669	1.730118309	0.423998211	4.080484929	4.49418E-05	0.000167979
AP4M1	6.031029336	-2.593966268	0.635738806	-4.080238999	4.49894E-05	0.00016811
NMT2	28.70473794	1.459082749	0.35762764	4.079893687	4.50563E-05	0.000168313
RAB32	4.927591444	3.240762227	0.794509838	4.078945373	4.52405E-05	0.000168953
ALDH16A1	44.78329559	-1.062093671	0.260432749	-4.078187848	4.53881E-05	0.00016941
ATP6V0D1	44.53033147	1.095862718	0.268709746	4.078239567	4.5378E-05	0.00016941
HOXB2	2.288261534	4.546593272	1.115088237	4.077339461	4.5554E-05	0.000169982

LOC101116843	3.957032898	-4.352088438	1.067471771	-4.077005647	4.56194E-05	0.000170178
STARD4	19.53515922	1.280999341	0.31430783	4.075620195	4.58919E-05	0.000171147
SRSF12	7.172573652	2.53467121	0.622023994	4.074876911	4.60387E-05	0.000171647
MS4A10	3.42530366	5.100212197	1.251817546	4.074245654	4.61637E-05	0.000172065
PWP2	19.83059317	-1.329852271	0.326435009	-4.073865351	4.62392E-05	0.000172298
RASL11A	5.048061734	3.254126703	0.798969041	4.072907132	4.64299E-05	0.000172961
RPP38	13.57932768	-1.541333829	0.378458427	-4.07266352	4.64785E-05	0.000173093
RBM12B	101.8708572	0.881456359	0.216483155	4.071708763	4.66695E-05	0.000173756
RPF1	32.96236815	-1.108888002	0.272360478	-4.071398356	4.67318E-05	0.000173939
LOC121818384	9.81873278	-1.911824454	0.469702389	-4.07028897	4.69549E-05	0.000174721
TIA1	120.2049134	0.690278698	0.169607407	4.069861754	4.7041E-05	0.000174993
SEC24D	19.91687019	1.298012391	0.318938657	4.069786966	4.70561E-05	0.000175001
PLD3	8.125296178	-2.351198896	0.577774112	-4.069408524	4.71326E-05	0.000175236
HPGDS	2.463060202	4.634827035	1.139036165	4.06907803	4.71995E-05	0.000175414
PAQR7	2.473151432	4.654540315	1.143890696	4.0690429	4.72067E-05	0.000175414
HOMER1	45.70424335	0.902107957	0.221780763	4.067566302	4.75067E-05	0.00017648
ARHGAP31	2.410144937	4.593825012	1.1294594	4.067277682	4.75656E-05	0.000176649
ADCY7	3.743782782	4.060441281	0.998480186	4.066621788	4.76996E-05	0.000177098
RBM24	5.417432061	3.388852046	0.833644743	4.06510336	4.80112E-05	0.000178205
TEDC2	5.715205787	-2.632366005	0.647582275	-4.064913612	4.80503E-05	0.000178301
LOC101118202	6.922877128	-2.298241935	0.565442514	-4.06450148	4.81352E-05	0.000178567
FAM210B	22.9914795	-1.289041214	0.317184311	-4.064013153	4.82361E-05	0.000178891
FZD5	118.5133017	-0.899297292	0.221308808	-4.063540437	4.83339E-05	0.000179204
PAK2	165.8154941	0.726292025	0.178793271	4.062188814	4.86147E-05	0.000180195
CALM1	690.7422174	0.47695552	0.117467599	4.060315573	4.90064E-05	0.00018154
LOC101116641	2.67284827	4.786587895	1.17888715	4.060259623	4.90182E-05	0.00018154
NR1I2	14.08993464	1.488166712	0.366510049	4.060370834	4.89948E-05	0.00018154
LOC121819204	2.359056833	4.609422572	1.135351039	4.059909592	4.90917E-05	0.000181762
CASP6	99.86709003	-1.13264703	0.279020172	-4.059373281	4.92046E-05	0.000182086
LOC105616451	9.397978508	-2.215691599	0.545822394	-4.059363677	4.92066E-05	0.000182086
ATAD2B	69.02726314	0.806092105	0.198598034	4.058912816	4.93017E-05	0.000182388
LOC101113599	151.6978095	0.649253986	0.159969637	4.058607598	4.93662E-05	0.000182576
LOC121817654	3.119514757	4.336736061	1.068650555	4.058142337	4.94646E-05	0.000182889
PARP2	46.26321622	-0.890918359	0.219546322	-4.057997189	4.94954E-05	0.000182952
RPP40	4.623205662	-3.52594749	0.868949988	-4.057710498	4.95561E-05	0.000183126
TUBGCP3	16.33502514	-1.56696791	0.386176709	-4.057644791	4.95701E-05	0.000183127
ABCF1	130.7992601	0.756416947	0.186433836	4.057294329	4.96445E-05	0.000183301
ARHGEF19	26.50815251	-1.248714782	0.307767565	-4.057330676	4.96368E-05	0.000183301
SH3PXD2B	19.16340224	-1.371521757	0.338047951	-4.057181103	4.96686E-05	0.000183339
MN1	2.360936329	4.555070614	1.123011363	4.05612157	4.98943E-05	0.000184121
PRR13	46.16383447	1.146756984	0.282790213	4.055150886	5.0102E-05	0.000184837
TPX2	67.07613993	-0.816475452	0.201347314	-4.055060057	5.01214E-05	0.000184857

TNFSF8	2.334479108	4.584454912	1.130653127	4.054696176	5.01995E-05	0.000185094
TCEAL4	65.23369503	0.896819823	0.221198262	4.054371021	5.02694E-05	0.000185301
AHNAK2	8.389596849	-2.343283259	0.577987057	-4.054214073	5.03031E-05	0.000185334
BMF	14.7853666	-1.701351102	0.419651525	-4.054199732	5.03062E-05	0.000185334
YIPF2	26.45517486	1.163328739	0.286968923	4.053849194	5.03817E-05	0.000185561
NEDD8	98.40249099	-0.816768183	0.201505394	-4.053331615	5.04933E-05	0.000185921
TPH1	3.21130399	4.409873225	1.087994712	4.053212001	5.05192E-05	0.000185965
LOC101106871	39.88703404	-1.491009982	0.367942172	-4.05229434	5.07178E-05	0.000186645
SOX3	2.198383059	-4.659358649	1.149899195	-4.051971397	5.07879E-05	0.000186851
HOOK2	92.50010039	-0.791499675	0.195346155	-4.051780165	5.08294E-05	0.000186953
MRPS23	60.80884414	-0.918950645	0.226900749	-4.050011519	5.12151E-05	0.000188319
TMEM171	7.098447689	2.389907735	0.590134985	4.049764535	5.12692E-05	0.000188466
GATD3	30.34627946	-1.300396895	0.321114262	-4.049639175	5.12967E-05	0.000188516
FBN2	8.48160231	-2.175377729	0.53723065	-4.049243517	5.13835E-05	0.000188783
SURF6	146.647627	-0.734604414	0.181465499	-4.048176759	5.16182E-05	0.000189593
SETD7	52.23165828	1.124352104	0.277849139	4.046627996	5.19607E-05	0.000190799
AKTIP	21.90866029	-1.382482635	0.341674837	-4.046193886	5.20571E-05	0.0001911
PDE9A	12.2568281	1.643178555	0.406258873	4.044658877	5.23994E-05	0.000192304
SLITRK2	7.050080466	-2.313335342	0.571973194	-4.044482093	5.24389E-05	0.000192396
P2RY2	5.366935108	-2.790575046	0.690356275	-4.042224493	5.29465E-05	0.000194205
SRPK2	138.5909798	-0.795294324	0.196779309	-4.041554605	5.3098E-05	0.000194707
MYBPC1	5.136875324	3.242393011	0.802459664	4.040568215	5.33219E-05	0.000195474
ST3GAL5	4.600795286	3.519533087	0.871225481	4.039749945	5.35082E-05	0.000196104
TMEM117	3.212880424	4.435944211	1.098164763	4.039415905	5.35845E-05	0.00019633
WAS	3.186443015	4.378523883	1.084030905	4.039113519	5.36536E-05	0.000196529
CPXM2	2.292536282	4.568334042	1.13143998	4.037628263	5.39943E-05	0.000197723
CAMTA2	10.98369794	-1.761996909	0.436515015	-4.036509281	5.42524E-05	0.000198613
UPF1	56.53422167	-1.080239038	0.267688418	-4.03543435	5.45014E-05	0.00019947
LOC132659052	3.990296243	4.155093191	1.029695332	4.035264667	5.45408E-05	0.00019956
TFF1	36.51343467	3.666286542	0.908675701	4.034757987	5.46586E-05	0.000199936
MAL	2.468919073	4.68211067	1.160490955	4.03459471	5.46966E-05	0.000200021
PAFAH1B3	11.9715218	-1.762360808	0.436833154	-4.034402592	5.47414E-05	0.00020013
NOTCH2	29.29412776	1.091131514	0.270509514	4.033616046	5.4925E-05	0.000200705
WASF1	2.997984907	4.297903079	1.065525432	4.033599714	5.49289E-05	0.000200705
ERH	25.70944865	-1.763436992	0.437224848	-4.033249713	5.50108E-05	0.000200949
ATP2B4	38.77753996	1.16619262	0.289166594	4.032943795	5.50825E-05	0.000201156
WDR48	29.51728758	1.103602564	0.273768817	4.031147801	5.55051E-05	0.000202644
TRAM1	83.04381239	0.791774892	0.19643943	4.03063118	5.56273E-05	0.000203035
LOC121819090	24.91745175	-1.108835347	0.2751288	-4.030240911	5.57197E-05	0.000203317
COL8A1	2.268456931	4.539922756	1.126484031	4.030170541	5.57364E-05	0.000203322
CCNT1	37.26923837	0.998208043	0.247719374	4.029592151	5.58737E-05	0.000203768
SH2B1	51.139865	-0.976266953	0.242309684	-4.029005104	5.60134E-05	0.000204221

TDG	32.45400849	1.029945222	0.255734868	4.027394576	5.63983E-05	0.000205569
MAD2L1BP	10.54907367	-1.945005165	0.48299323	-4.026982249	5.64973E-05	0.000205873
NNT	128.7724088	0.639510776	0.158820094	4.026636414	5.65804E-05	0.00020612
C12H1orf21	4.966450278	3.004902479	0.746294305	4.026430941	5.66299E-05	0.000206244
ARPC5	34.95200201	1.020026108	0.253414928	4.025122429	5.69457E-05	0.000207338
GPR52	3.744862835	4.007565649	0.995880785	4.024141953	5.71834E-05	0.00020806
LOC132659058	8.684176199	1.988350471	0.494109181	4.024111569	5.71908E-05	0.00020806
MCMBP	26.53573415	-1.260574792	0.313253557	-4.024135605	5.7185E-05	0.00020806
ZDHHC12	4.92763738	-2.99764796	0.745203309	-4.022590782	5.75615E-05	0.000209351
LOC132658413	2.340510473	4.546634581	1.130649744	4.021258225	5.78881E-05	0.000210482
ADGRD1	3.126827421	4.344102641	1.08081079	4.019299846	5.83713E-05	0.000212182
CACNA1C	5.444661376	3.059683258	0.761703848	4.016893528	5.89704E-05	0.000214301
CAND1	62.41677092	-0.79195312	0.197159384	-4.016816761	5.89896E-05	0.000214312
RIPK4	21.11196721	-1.259021675	0.313510943	-4.015877927	5.92249E-05	0.000215109
PGRMC1	9.322086973	2.070086809	0.515502525	4.015667643	5.92777E-05	0.000215242
LOC114118019	6.585977695	-2.44268998	0.608326718	-4.015424452	5.93389E-05	0.000215406
CCT7	127.7777579	-0.608200443	0.151508796	-4.0142913	5.96247E-05	0.000216384
ABHD11	47.47745106	-0.917201676	0.228511603	-4.013807891	5.9747E-05	0.000216732
LOC114116836	2.526144464	4.660264301	1.161064902	4.013784495	5.97529E-05	0.000216732
GTPBP3	22.48042005	-1.324310267	0.33005766	-4.012360351	6.01146E-05	0.000217926
MARCHF2	5.999736176	2.790506969	0.695468536	4.012412963	6.01012E-05	0.000217926
LOC132657935	3.477153641	-4.247894081	1.059108231	-4.010821518	6.05078E-05	0.000219232
PID1	2.288820756	4.525090867	1.12820927	4.01086127	6.04977E-05	0.000219232
SFXN4	27.83586094	-1.178368526	0.293907909	-4.009312068	6.08959E-05	0.000220578
IL1R2	3.756878005	4.028954026	1.005034901	4.008770264	6.10358E-05	0.000221025
ARPC5L	95.24088904	-0.875737828	0.218577485	-4.006532646	6.16166E-05	0.000223068
XIAP	13.22505108	1.626119391	0.405890916	4.006296584	6.16782E-05	0.000223231
RRP1	8.408004126	-1.979837836	0.494215749	-4.006019312	6.17506E-05	0.000223432
DIRAS3	15.41667081	-1.817566678	0.453731868	-4.005816666	6.18036E-05	0.000223563
SPATA24	10.98190091	-1.785275779	0.445713709	-4.005431611	6.19043E-05	0.000223867
PES1	81.06413647	1.095649982	0.273572956	4.004964512	6.20268E-05	0.000224249
SAV1	12.19008997	1.842415678	0.460105953	4.004329144	6.21937E-05	0.000224792
IRF3	12.03495447	1.645765666	0.411020465	4.004096649	6.22549E-05	0.000224952
SIRPB2	2.228713293	4.502594459	1.125068258	4.002063364	6.27925E-05	0.000226834
AP2S1	29.11207376	1.059361615	0.264730384	4.001662373	6.2899E-05	0.000227157
ZNF16	6.502864457	-2.305506471	0.576182925	-4.001344664	6.29835E-05	0.000227401
TLR8	2.218941754	4.51728357	1.129058956	4.000927981	6.30946E-05	0.00022774
COMMD5	29.74717756	-1.031287574	0.257774521	-4.000735104	6.3146E-05	0.000227864
FAM3D	4.66486279	3.188911684	0.797406772	3.999102835	6.35831E-05	0.00022938
PSMA7	619.2885375	0.642992278	0.160814593	3.998345341	6.37868E-05	0.000230053
CD101	2.932516587	4.279546359	1.070435316	3.99794952	6.38936E-05	0.000230375
MZT1	14.55209646	1.565085447	0.391492558	3.997739968	6.39501E-05	0.000230517

LOC101109677	6.444252299	2.8958956	0.724938997	3.994674876	6.47831E-05	0.000233457
TMSB15B	4.046955512	-3.819557871	0.956219877	-3.994434715	6.48488E-05	0.00023363
GUCA2B	6.316057745	2.572724294	0.644163511	3.993899452	6.49954E-05	0.000234096
ZNF687	54.88194307	0.88762796	0.22225172	3.993795689	6.50239E-05	0.000234135
FBXL22	2.768293271	4.765014495	1.193135976	3.993689396	6.50531E-05	0.000234177
LAMP1	127.2819287	-0.903868427	0.226357061	-3.993109041	6.52126E-05	0.000234688
EAF2	2.986164631	4.315718487	1.080917173	3.992644946	6.53404E-05	0.000235085
SLC25A15	155.5130808	0.700120704	0.175371572	3.992213201	6.54595E-05	0.00023545
SLC38A3	12.71782801	1.999116399	0.500789675	3.991928145	6.55382E-05	0.00023567
TMEM176A	5.567919475	3.019317378	0.756441581	3.991474628	6.56637E-05	0.000236057
JPH1	21.90929062	-1.242029553	0.311262715	-3.990293388	6.59916E-05	0.000237172
CHL1	2.418381678	4.597052256	1.152111281	3.990111313	6.60423E-05	0.000237291
CACNA2D1	10.58327907	1.844061453	0.462200485	3.989743655	6.61447E-05	0.000237595
LOC105608537	2.906581058	4.276514853	1.072138049	3.988772582	6.64161E-05	0.000238505
CERT1	132.8071018	0.689272077	0.172819743	3.988387355	6.6524E-05	0.000238829
SLC5A1	3.84520771	4.08590236	1.024501341	3.988186442	6.65803E-05	0.000238967
PYGL	22.38568427	-1.16007325	0.29103762	-3.985990706	6.71991E-05	0.000241123
LOC105603808	3.264360568	4.378386838	1.098490192	3.985822421	6.72468E-05	0.000241229
HDAC9	3.173432838	4.393553339	1.102475437	3.985171179	6.74315E-05	0.000241827
LOC101105064	45.17447182	-0.985855237	0.247423983	-3.984477279	6.76288E-05	0.00024247
WDR6	11.25468599	-1.730715135	0.434391027	-3.984233163	6.76984E-05	0.000242654
CPEB2	13.65315298	1.461221466	0.36678821	3.983828886	6.78137E-05	0.000243002
TSNARE1	12.29882947	-2.021451514	0.507529728	-3.982922382	6.8073E-05	0.000243866
PIP5K1B	18.35037825	1.479911244	0.371663648	3.981856317	6.83791E-05	0.000244897
G3BP2	83.93077733	0.762018135	0.191508096	3.979038748	6.91945E-05	0.000247751
SKA3	3.971877185	-3.845553957	0.967268409	-3.975684435	7.01771E-05	0.000251202
CELSR1	7.345031303	-2.115914346	0.532231312	-3.97555405	7.02156E-05	0.000251246
WDR89	11.14847082	-1.658806749	0.417255799	-3.97551515	7.02271E-05	0.000251246
KNSTRN	27.58764986	-1.119754657	0.28173182	-3.974540951	7.05151E-05	0.000252209
WDR35	7.307205701	2.397333486	0.60323944	3.974099379	7.06461E-05	0.00025261
TSN	140.9639259	-0.70225083	0.176723973	-3.973715733	7.076E-05	0.00025295
WBP11	57.53658876	-0.7995516	0.201266917	-3.972593269	7.10944E-05	0.000254077
GLRX3	54.50700199	-0.973781476	0.245137425	-3.972390076	7.11551E-05	0.000254226
LOC101122262	52.3385865	-1.009271817	0.254084066	-3.972196421	7.1213E-05	0.000254342
ODR4	21.78047308	1.202115392	0.302635657	3.97215385	7.12257E-05	0.000254342
LUC7L3	1033.422364	0.960338777	0.241782101	3.971918396	7.12961E-05	0.000254526
FBXW5	10.79910439	1.693032488	0.426290143	3.971549698	7.14066E-05	0.000254852
CD80	2.240849248	4.526611362	1.139807837	3.971381152	7.14571E-05	0.000254964
GALNT12	31.60717253	1.357489695	0.342058779	3.968586036	7.23004E-05	0.000257904
DQX1	12.85374643	1.720998098	0.433662577	3.968518822	7.23207E-05	0.000257908
MED1	190.7970321	-0.494501809	0.124618818	-3.968115081	7.24434E-05	0.000258276
CEP128	37.85346275	1.156706299	0.291511431	3.967962056	7.24899E-05	0.000258373

CA13	4.070072252	-3.827765629	0.964691196	-3.96786624	7.2519E-05	0.000258408
TNK1	7.49226276	-2.017211267	0.508554355	-3.96655981	7.29175E-05	0.000259759
PAQR3	7.106007004	2.156644751	0.543751178	3.966234627	7.3017E-05	0.000260044
LOC101121599	23.37626511	-1.20688896	0.304304896	-3.966051733	7.3073E-05	0.000260174
CNOT4	126.8859374	0.754330425	0.190278386	3.964351615	7.35957E-05	0.000261965
ACSL1	67.47490165	0.897249861	0.226334524	3.964264242	7.36227E-05	0.000261992
NADSYN1	51.83198191	0.89185905	0.22503451	3.963210136	7.39486E-05	0.000263082
ANKRD16	13.8204735	-1.502339095	0.379169871	-3.962179518	7.42687E-05	0.00026408
DERL3	2.274206375	4.497054161	1.13497907	3.962235323	7.42513E-05	0.00026408
VGLL1	2.081582632	-4.642468732	1.171811568	-3.961787762	7.43907E-05	0.000264443
LOC101111528	7.949440634	2.152144355	0.543261606	3.961524857	7.44726E-05	0.000264523
NKRF	16.63786468	-1.315533175	0.332072275	-3.9615869	7.44533E-05	0.000264523
RCSD1	2.862532658	4.278423271	1.07998666	3.961551961	7.44642E-05	0.000264523
NOP10	17.34095755	-1.272747143	0.321411574	-3.959867178	7.49915E-05	0.000266295
LOC132657576	2.021946413	-4.573723514	1.155178873	-3.959320603	7.51633E-05	0.000266835
LOC132658418	2.261764606	4.493855705	1.135413086	3.957903746	7.56104E-05	0.000268351
STAU1	151.8855902	0.766805247	0.193769692	3.957302306	7.5801E-05	0.000268956
C1RL	8.182442663	2.197659254	0.555579392	3.955616937	7.63374E-05	0.000270657
LOC101116389	10.49204964	-1.680855545	0.424928367	-3.955620932	7.63361E-05	0.000270657
TMEM238L	6.930960468	-2.404423294	0.607852286	-3.955604591	7.63413E-05	0.000270657
ZC3H15	432.6392282	0.740873825	0.187329563	3.95492208	7.65596E-05	0.000271359
MKRN2OS	17.81467173	-1.273401596	0.322092035	-3.953533333	7.70055E-05	0.000272795
PLXND1	2.971898069	4.279724447	1.082502563	3.95354671	7.70012E-05	0.000272795
BIRC5	17.73062653	-1.379339087	0.348899474	-3.953399729	7.70486E-05	0.000272875
CLIP2	8.622676661	2.233562283	0.565213469	3.951714542	7.75933E-05	0.000274732
BATF3	2.67643781	4.729251418	1.197457042	3.949412174	7.83433E-05	0.000277314
KAT2A	10.82092733	-1.782323507	0.451362283	-3.948764825	7.85555E-05	0.000277991
CHI3L2	4.561793297	3.540356504	0.896701851	3.948198053	7.87417E-05	0.000278576
SEC23IP	40.51408281	0.982139152	0.248858132	3.94658251	7.92746E-05	0.000280388
FST	2.838081785	4.248233561	1.076684005	3.945664225	7.95791E-05	0.00028139
CRELD2	10.31789227	1.675101617	0.424768373	3.943564834	8.02793E-05	0.000283791
MISFA	6.604024374	-2.355705619	0.597611793	-3.941866019	8.08501E-05	0.000285582
PCSK1	6.716606309	2.221437543	0.563547645	3.941880626	8.08452E-05	0.000285582
TIMM22	36.21790702	-1.040574956	0.263979686	-3.941875126	8.08471E-05	0.000285582
RAB1A	94.49973821	-0.877842597	0.222723526	-3.941400411	8.10073E-05	0.000286047
TC2N	47.74883255	1.134264804	0.287785916	3.941349258	8.10245E-05	0.000286047
LOC101119975	42.35080665	1.103282329	0.280022685	3.939974821	8.14902E-05	0.000287608
STARD8	2.845701033	4.239803627	1.076114949	3.939917043	8.15098E-05	0.000287608
BICDL2	8.650711437	-1.947480749	0.494603423	-3.937459098	8.2349E-05	0.000290493
LOC105615523	17.78486448	-1.345828356	0.341841276	-3.936997815	8.25074E-05	0.000290974
KEF53_p06	2477.172899	1.494734839	0.379719243	3.936421099	8.27058E-05	0.000291597
NFU1	17.46675429	-1.225279912	0.311327728	-3.935659443	8.29686E-05	0.000292447

SERHL2	93.43253969	-0.885897014	0.225103015	-3.935518203	8.30174E-05	0.000292542
TOP1	205.3390706	0.550045116	0.139766758	3.93545019	8.30409E-05	0.000292547
SUSD4	19.10548577	-1.243854263	0.316084136	-3.935199907	8.31275E-05	0.000292775
ACBD6	11.25068393	-1.622655462	0.412407126	-3.934596085	8.33367E-05	0.000293435
PKP2	43.57378169	-0.937138377	0.238203304	-3.934195544	8.34758E-05	0.000293847
ARFGAP3	84.29051874	0.743377093	0.188991729	3.933384271	8.37582E-05	0.000294763
IGFBP4	5.871111889	2.781474506	0.707541725	3.931180887	8.45296E-05	0.0002974
PTTG1IP	164.9816068	0.746171564	0.189852367	3.930272643	8.48496E-05	0.000298369
TTLL6	2.380947767	4.587300951	1.167154029	3.930330391	8.48292E-05	0.000298369
CD300E	2.332307251	4.566354244	1.161997365	3.929745783	8.50357E-05	0.000298945
RASSF6	19.88091722	-1.261344503	0.321035419	-3.928988603	8.53039E-05	0.000299808
EIF1B	18.47253337	1.622017095	0.412874267	3.928598184	8.54425E-05	0.000300217
SHC1	55.45272821	0.838497198	0.213472282	3.927897299	8.56918E-05	0.000301014
LOC121817847	2.213648293	4.49564476	1.144680432	3.92742344	8.58607E-05	0.000301528
LOC105609801	15.57938968	-1.627684335	0.414526335	-3.926612615	8.61506E-05	0.000302466
AP5M1	15.37444231	-1.407387873	0.358435526	-3.926474281	8.62001E-05	0.000302556
ELL2	56.92143489	0.79985834	0.203712154	3.92641443	8.62215E-05	0.000302556
ANXA13	205.3433022	-0.751250326	0.191433218	-3.924346752	8.69654E-05	0.000305086
SAAL1	20.65962389	-1.162702851	0.296346337	-3.923459496	8.72864E-05	0.000306132
LOC101116862	2.131788512	4.436889961	1.130921379	3.923252352	8.73616E-05	0.000306315
CSAD	39.33207463	-1.316756199	0.335676837	-3.92269008	8.75658E-05	0.000306951
XBP1	73.69802248	-1.145792506	0.292169008	-3.921677094	8.79348E-05	0.000308164
FAM171A1	4.201004843	3.701886598	0.944034307	3.921347529	8.80552E-05	0.000308505
LOC101116543	2.071789653	4.414166719	1.125916669	3.920509252	8.8362E-05	0.000309499
C1H1orf122	17.72186069	-1.585966567	0.404575193	-3.920078629	8.85201E-05	0.000309971
VLDLR	23.92079143	-1.23465968	0.315045574	-3.918987542	8.89217E-05	0.000311296
PDXP	5.795655275	-2.362098246	0.602763031	-3.918784207	8.89967E-05	0.000311477
PLAA	53.36548764	0.868191721	0.221597211	3.917881979	8.93304E-05	0.000312563
ITGA8	2.175764509	4.434303906	1.131998062	3.917236307	8.95699E-05	0.000313319
PREB	21.35400529	1.969598982	0.502911172	3.916395363	8.98828E-05	0.000314331
NEXMIF	18.24415491	1.411548534	0.360525343	3.91525467	9.03088E-05	0.000315675
ROBO1	6.455695885	2.286966446	0.58411912	3.915239835	9.03144E-05	0.000315675
LOC101105867	4.734418578	3.455372606	0.882695035	3.914571249	9.0565E-05	0.000316468
PARP10	12.48312461	1.605012071	0.410051358	3.914173285	9.07145E-05	0.000316908
LOC132658270	4.880591	-3.194635567	0.816266411	-3.913716801	9.08862E-05	0.000317425
STON1	4.052501337	3.684309573	0.942197435	3.910337088	9.21674E-05	0.000321815
MED12	17.35574532	1.470063488	0.375960328	3.910155886	9.22366E-05	0.000321973
RNASEH1	43.28742832	-0.89268813	0.228309178	-3.909996683	9.22974E-05	0.000322101
PPARG	10.89562031	1.831235809	0.468616528	3.907749088	9.316E-05	0.000325026
COL4A4	5.985577165	2.481944421	0.635223495	3.907198714	9.33724E-05	0.000325682
MVP	19.40905073	1.358495973	0.347725967	3.906800475	9.35263E-05	0.000326134
LOC101103396	21.80990577	1.304957667	0.334038673	3.906606543	9.36014E-05	0.000326311

SHF	14.29460252	-1.609208922	0.411975858	-3.906075782	9.38071E-05	0.000326943
PDSS1	21.36299462	1.359002265	0.34795441	3.905690594	9.39567E-05	0.000327379
FHL3	4.343951454	3.3839892	0.86663932	3.904726131	9.43322E-05	0.000328601
PTPRR	2.796018803	4.218293856	1.080804981	3.902918591	9.50397E-05	0.00033098
LOC101105208	4.800250085	3.577007943	0.916538646	3.902735534	9.51116E-05	0.000331144
CENPF	305.0919785	-0.720204054	0.184546902	-3.902552937	9.51834E-05	0.000331221
HTATSF1	123.8826027	0.830391622	0.212778971	3.902601933	9.51641E-05	0.000331221
RAVER2	13.6816627	-1.433301314	0.367583001	-3.899258967	9.64875E-05	0.000335672
XRCC3	8.646009448	-1.926397648	0.49405258	-3.89917536	9.65209E-05	0.000335701
CDK5R1	10.70181969	-1.821283015	0.467106601	-3.8990736	9.65614E-05	0.000335754
ADCY9	11.60367697	1.63258522	0.418730992	3.898887949	9.66355E-05	0.000335844
FURIN	34.65790678	0.975617342	0.250229969	3.898882874	9.66375E-05	0.000335844
SBNO1	194.6237379	0.629583548	0.161508526	3.898144357	9.69326E-05	0.000336782
E2F4	20.02417874	-1.272953308	0.326596311	-3.897635294	9.71366E-05	0.000337315
LOC101103616	62.60667877	1.024645222	0.262886237	3.897675405	9.71205E-05	0.000337315
MTIF3	43.00872323	0.859958965	0.220645071	3.897476445	9.72003E-05	0.000337449
TRIB3	3.992219988	-3.382008983	0.867881558	-3.896855457	9.74497E-05	0.000338227
ITK	2.047818905	4.380386198	1.124371683	3.89585247	9.78539E-05	0.000339532
SLC7A9	4.110628832	3.68705966	0.946419916	3.895796779	9.78764E-05	0.000339532
GRAP	3.835577843	3.584662035	0.920223719	3.895424514	9.80269E-05	0.000339966
CACFD1	9.46035622	-1.770450734	0.454536808	-3.895065706	9.81721E-05	0.000340381
GPX8	4.539803746	3.75983421	0.965336265	3.89484405	9.82619E-05	0.000340604
STAB1	2.899142001	4.239908742	1.088930402	3.893645301	9.8749E-05	0.000342204
ADSS1	14.21705367	-1.475320211	0.379151749	-3.891107496	9.97877E-05	0.000345713
VASN	2.096601543	4.425475068	1.137362581	3.890997595	9.98329E-05	0.00034578
YIPF3	14.63795734	-1.522015296	0.391192324	-3.890708487	9.9952E-05	0.000346103
CC2D1B	57.8145859	-0.725818642	0.186571213	-3.890303502	0.000100119	0.000346591
ROR2	3.468845446	3.957106775	1.017826481	3.887800966	0.000101157	0.000350093
HIC1	2.068885256	4.412201596	1.135257827	3.886519423	0.000101692	0.000351854
TMEM259	79.88708209	-0.966671843	0.248745408	-3.886189711	0.00010183	0.000352241
BMP2K	26.7810664	1.22139665	0.314382272	3.885068465	0.000102301	0.000353688
LOC100101238	2.30275844	4.53630809	1.167615221	3.885105307	0.000102286	0.000353688
MAP3K1	36.83859922	0.909915505	0.234302971	3.88349964	0.000102964	0.000355886
JAKMIP3	2.616446639	4.70464391	1.211607556	3.882976702	0.000103185	0.000356561
ZRSR2	37.76441861	1.267956456	0.326568685	3.882663934	0.000103318	0.000356928
ABHD6	25.94457315	1.149124768	0.295990948	3.882296998	0.000103474	0.000357374
UFSP2	17.08799516	-1.569818869	0.404366868	-3.882164914	0.000103531	0.000357476
ACSS3	41.15164582	-1.047863303	0.270009256	-3.88084216	0.000104095	0.000359241
MAP4K4	32.2453721	-1.063874866	0.274134569	-3.880848997	0.000104092	0.000359241
ZNF532	5.704435055	2.448575803	0.631016858	3.880365113	0.0001043	0.000359853
PODXL	10.42341666	1.644417885	0.423793782	3.880231271	0.000104357	0.000359958
RILPL2	8.25051513	2.378310773	0.613067443	3.879362376	0.000104731	0.000361153

PEX11G	11.2870307	1.743550264	0.449472508	3.879103246	0.000104842	0.000361445
NUDT21	64.60773422	-0.882642855	0.2275624	-3.878684948	0.000105023	0.000361973
GIMAP8	3.739854553	3.969303821	1.023669375	3.877525222	0.000105524	0.000363609
OVAR	5.75360825	2.704759008	0.697801328	3.876116163	0.000106137	0.000365626
KPNA4	92.39733	0.697469267	0.179976934	3.875325868	0.000106482	0.00036672
PTK2	54.68632579	-0.985350531	0.254300019	-3.874756024	0.000106732	0.000367484
RAB8A	78.71796656	0.727753712	0.187829306	3.874548259	0.000106823	0.000367703
PDXDC1	89.55322269	1.180696217	0.304794617	3.873743667	0.000107176	0.000368825
RAB24	75.76105767	-0.895485705	0.231172427	-3.873670043	0.000107209	0.000368842
EXOSC6	67.71164011	-0.741240574	0.191369741	-3.873342615	0.000107353	0.000369243
MRI1	4.210716133	-3.362853233	0.868424985	-3.872358916	0.000107787	0.000370641
LAMA3	10.67480686	-1.689106516	0.436227841	-3.872074077	0.000107913	0.000370979
MCUB	46.70574875	-0.821816853	0.212256891	-3.871802939	0.000108033	0.000371297
AAK1	86.3598071	0.705577187	0.182268908	3.871078151	0.000108355	0.000372307
CHST4	5.635779224	2.673526576	0.690704928	3.870721733	0.000108514	0.000372756
SLC25A44	29.77360166	-0.951543745	0.245962855	-3.868648158	0.00010944	0.000375843
LOC132658352	1128.80121	-0.617073646	0.159547067	-3.867658992	0.000109885	0.000377208
MSANTD2	24.01572365	-1.13135105	0.292517258	-3.867638639	0.000109894	0.000377208
YIPF5	24.97516834	1.152013378	0.29790823	3.867007567	0.000110179	0.000378088
UBAC2	23.19970529	-1.133031566	0.293007804	-3.866898939	0.000110228	0.000378159
GATA5	3.395201833	3.911749449	1.011886683	3.865797936	0.000110727	0.000379772
MMGT1	25.67002172	-1.445989034	0.374055764	-3.865704453	0.000110769	0.00037982
EPN1	41.50566051	0.977289221	0.252839971	3.865248119	0.000110976	0.000380433
RCC1L	8.474433703	-1.857227776	0.48066594	-3.863863906	0.000111607	0.000382499
CNOT2	125.7392024	0.732964099	0.189718354	3.863432733	0.000111805	0.000383077
PSMC6	116.7088161	-0.747158946	0.19342645	-3.862754795	0.000112116	0.000384043
TRPM4	4.911881785	2.756415259	0.713693764	3.862182069	0.000112379	0.000384846
TMEM45B	26.60133115	1.123081234	0.290864807	3.861179516	0.000112841	0.00038633
FGD5	2.668315336	4.161998427	1.077988873	3.860891824	0.000112974	0.000386587
LOC121817139	2.224280163	-4.614881472	1.195282281	-3.860913481	0.000112964	0.000386587
WDR25	6.840832242	-2.213505581	0.573423293	-3.860159865	0.000113313	0.000387648
RASSF5	10.21344077	1.893007593	0.490551024	3.858941276	0.000113879	0.000389486
TIMM21	22.46002194	1.281136658	0.332043815	3.858336162	0.000114162	0.000390351
PPP1R9A	3.785442118	4.058496801	1.051902417	3.858244582	0.000114204	0.000390398
UGGT1	27.99774451	1.217098786	0.315487884	3.857830512	0.000114398	0.00039096
LOC132659109	2.663803308	-4.317047536	1.11916867	-3.857369897	0.000114614	0.000391597
GNG10	26.73544286	-1.501735352	0.389332798	-3.857202267	0.000114692	0.000391765
PCSK4	4.473514361	-2.931604062	0.760219588	-3.85625957	0.000115135	0.000393178
LOC105606460	2.878752346	4.233361204	1.097831652	3.85611145	0.000115205	0.000393315
LOC132658751	3.510164411	3.922743184	1.017326448	3.85593355	0.000115289	0.000393501
INTS9	5.296507268	-3.145026913	0.815764247	-3.855313497	0.000115581	0.000394373
WAC	122.3318124	0.560050596	0.14526894	3.85526731	0.000115603	0.000394373

SLC35C2	27.48960897	-1.040679355	0.270017062	-3.854124432	0.000116145	0.000396118
FH	51.15572221	-1.158107225	0.300495126	-3.853996706	0.000116205	0.000396224
CCS	13.52682012	-1.596681244	0.41439935	-3.853001321	0.000116679	0.000397737
LAT	2.689559331	4.143358835	1.075697183	3.851789239	0.000117258	0.00039961
UNC119	17.81571468	-1.27765175	0.331876195	-3.849784255	0.000118222	0.000402792
RFTN1	2.152710059	4.465537957	1.16009382	3.849290358	0.000118461	0.000403502
MRPL35	62.7672628	-0.814346914	0.211638647	-3.847817608	0.000119175	0.000405832
MYO1F	2.039266725	4.35008082	1.130803257	3.846894492	0.000119624	0.000407259
ADGRE3	5.298059689	5.746967978	1.494034116	3.846610941	0.000119763	0.000407627
LOC121818651	2.343453752	4.53475841	1.179358202	3.845106943	0.0001205	0.000410031
STEAP2	44.23061094	-0.785051684	0.204245328	-3.843670215	0.000121208	0.000412335
CSNK2A1	326.3325788	0.575392843	0.149714218	3.843274553	0.000121404	0.000412896
LEF1	5.61691718	-2.693922621	0.700971356	-3.843127964	0.000121476	0.000413037
ELMOD2	34.06845582	1.05296031	0.274082184	3.841768532	0.000122151	0.000415226
ANGPT4	2.264671685	4.478601669	1.165790916	3.841685165	0.000122193	0.000415262
LOC105609758	17.94116375	1.413203284	0.367891616	3.841357681	0.000122356	0.000415711
LOC121819805	1.85324656	-4.512304821	1.174804937	-3.840897054	0.000122586	0.000416386
UBR3	41.01916971	0.877238545	0.228549525	3.838286442	0.000123896	0.00042073
TRAPPC2	23.36562299	1.364855588	0.355658389	3.837546454	0.00012427	0.000421892
MFSD4A	13.63842905	7.117513816	1.855222588	3.83647432	0.000124813	0.00042363
PEX5	12.85942686	1.538798025	0.401134479	3.836115079	0.000124996	0.000424132
TCEANC2	22.62761141	1.091662607	0.284579241	3.836058469	0.000125025	0.000424132
MTR	21.82685474	-1.246009956	0.325023754	-3.833596591	0.000126283	0.000428293
GPATCH2	25.56159779	1.126760546	0.293925399	3.833491593	0.000126337	0.000428367
USP11	29.2867366	-1.028177538	0.268217451	-3.833373011	0.000126398	0.000428465
LOC132657179	12.27727748	2.013338601	0.525243294	3.83315432	0.000126511	0.000428737
GCA	15.02838829	-1.316309679	0.343491607	-3.832145098	0.000127031	0.000430391
MRPL51	46.4283699	0.932661823	0.243385138	3.832040976	0.000127085	0.000430464
CCDC51	26.79224441	1.296744583	0.33841137	3.831858793	0.000127179	0.000430674
LOC101115897	25.88884453	1.169329148	0.305264938	3.830538666	0.000127863	0.000432882
PDE2A	12.01902719	1.724788496	0.450306287	3.830256308	0.00012801	0.000433269
DUS2	4.769368711	-2.870924448	0.749584897	-3.830019066	0.000128133	0.000433577
EXOSC9	35.05579213	-0.914242336	0.238789321	-3.82865671	0.000128845	0.000435874
WASHC5	7.186922145	-2.024030875	0.528677738	-3.828477595	0.000128938	0.000436081
DSTN	450.6553834	-0.496821448	0.129775874	-3.828303628	0.00012903	0.000436278
USF1	25.31737564	1.077212726	0.281403865	3.82799549	0.000129191	0.000436714
PARP12	13.31578886	1.459964403	0.381440525	3.827502083	0.00012945	0.00043748
BTG3	23.6740883	-1.14207011	0.29846297	-3.826505208	0.000129975	0.000439143
CBX1	31.43636145	-1.154506094	0.301726945	-3.826327463	0.000130069	0.000439346
CDIN1	87.81491697	0.962114444	0.251449887	3.826267153	0.000130101	0.000439346
THSD7A	3.975127511	3.621756637	0.946615451	3.826006257	0.000130239	0.0004397
CYSLTR1	2.057356277	4.382342087	1.145688293	3.825073637	0.000130733	0.000441257

RSPO2	2.032012495	4.346914924	1.136931275	3.823375273	0.000131637	0.000444197
BAG5	53.52923934	-0.798889844	0.208985459	-3.822705413	0.000131995	0.000445293
CHKA	66.14677962	-0.714439119	0.186902406	-3.822524994	0.000132092	0.000445507
LOC121818309	13.5984145	1.592692578	0.416683118	3.822311269	0.000132207	0.000445781
FOXO6	4.56702681	-2.850787275	0.745998493	-3.821438383	0.000132676	0.000447249
ATP13A1	9.914597326	1.802471627	0.471775526	3.820612827	0.00013312	0.000448537
FIGNL1	11.46411809	-1.559297486	0.40812845	-3.820604724	0.000133125	0.000448537
UBR5	130.8690677	-0.652673466	0.170907583	-3.818867808	0.000134066	0.000451593
IFT122	22.81410043	-1.117270639	0.292576124	-3.818734846	0.000134138	0.000451648
SP3	28.5971606	1.355586015	0.354985028	3.818713209	0.00013415	0.000451648
NCOR1	453.0992548	0.617821599	0.161793726	3.818575744	0.000134224	0.000451786
ARV1	5.998563218	-2.438159122	0.638687799	-3.817450603	0.000134838	0.000453737
SBDS	67.94835347	0.881900267	0.231030344	3.817248644	0.000134948	0.000453994
PER1	53.23627857	0.766249182	0.200768811	3.816574793	0.000135317	0.00045512
RALGDS	23.01511082	-1.279339772	0.335300335	-3.815504003	0.000135905	0.000456984
BMP6	1.984600209	4.325756497	1.134109507	3.814231758	0.000136607	0.000459228
PALMD	11.38501743	-1.515325122	0.397299114	-3.814066199	0.000136699	0.000459421
SLC3A1	3.623063626	3.533659873	0.926666256	3.81330371	0.000137121	0.000460725
ZCRB1	13.81935965	-1.39427224	0.365642574	-3.813210875	0.000137173	0.000460783
TPRG1L	13.39548701	-1.627805177	0.426946519	-3.812667644	0.000137475	0.000461681
EP400	71.78483201	-0.783935014	0.205628017	-3.812393985	0.000137627	0.000461891
PPCS	18.64380546	1.204876088	0.316035497	3.812470744	0.000137585	0.000461891
TFB1M	27.15732511	-1.064606072	0.279250527	-3.81236907	0.000137641	0.000461891
LOC121818661	2.301852942	4.497606418	1.179778432	3.812246686	0.000137709	0.000462004
NID2	3.408907719	3.944019138	1.034632281	3.812000855	0.000137846	0.000462348
TSGA10	11.89060096	-1.621952445	0.425639542	-3.810624446	0.000138616	0.000464813
ARFGEF1	99.73581295	0.758751811	0.199145212	3.810042947	0.000138943	0.000465791
PAN3	42.26612529	0.883914118	0.232050273	3.809149234	0.000139446	0.000467292
TRIM14	10.57339233	1.810732971	0.475367371	3.809123391	0.00013946	0.000467292
RUSC1	50.39989874	0.77998868	0.20481754	3.808212332	0.000139975	0.000468899
CWC15	120.2780811	0.767675477	0.201634841	3.807256093	0.000140517	0.000470597
MCL1	107.8812447	0.882105417	0.231844776	3.804724145	0.000141962	0.000475318
ATP2C2	6.49625139	-2.185877667	0.574808555	-3.802792508	0.000143074	0.00047892
LEPROTL1	61.21467301	-0.793002588	0.208599613	-3.801553501	0.000143792	0.000481202
LOC105613870	3.263922126	4.404863888	1.158900276	3.800899853	0.000144172	0.000482352
C20H6orf62	35.59932469	0.939944898	0.247370201	3.799749911	0.000144842	0.000484475
FAM133B	192.9320021	0.767790489	0.202091948	3.799213666	0.000145156	0.000485403
CLEC1A	1.994308217	4.356345764	1.146679533	3.79909612	0.000145225	0.000485511
TSPAN2	1.972049837	4.322577079	1.137920703	3.798662831	0.000145479	0.000486239
LOC105611547	7.391779476	-1.987250325	0.523154962	-3.798588317	0.000145523	0.000486264
SOX18	2.164232849	4.432390245	1.166962737	3.798227745	0.000145734	0.00048685
DTX3	9.600713412	2.054580098	0.540984938	3.797850831	0.000145956	0.000487469

ZNF408	19.42099819	-1.280374352	0.337281518	-3.796159242	0.000146955	0.000490683
NOXO1	6.062426939	2.467253545	0.649997857	3.795787199	0.000147176	0.000491296
ZNF276	24.04783324	1.080772715	0.284751367	3.795496144	0.000147348	0.00049175
ELN	1.947382751	4.3099631	1.1360527	3.793805605	0.000148356	0.000494989
GTF2H3	11.00837809	1.892438221	0.498872498	3.793430641	0.00014858	0.000495613
NUMA1	92.06942995	-0.705058806	0.185868734	-3.7933158	0.000148649	0.000495719
SHLD1	25.78888682	1.02196108	0.269437766	3.792939251	0.000148875	0.000496348
PIK3R1	191.1596505	-0.551950985	0.145528522	-3.792734088	0.000148998	0.000496634
CNOT7	104.0928378	-0.777308376	0.20504149	-3.790980925	0.000150053	0.000500029
CLDN5	2.024449769	4.391117526	1.158392955	3.790697712	0.000150225	0.000500474
LMNTD2	3.388946455	-4.093300922	1.07994639	-3.790281591	0.000150477	0.000501189
ABCB7	125.8954601	0.601610161	0.158774962	3.789074497	0.00015121	0.000503505
DCAF13	10.63082637	-1.591826111	0.42035487	-3.786862539	0.000152561	0.00050788
PPOX	21.30560467	-1.187279694	0.313637336	-3.785517724	0.000153389	0.000510507
LRRC41	17.74976741	-1.187018014	0.313574257	-3.785444718	0.000153434	0.00051053
CLK4	21.19364075	1.090470423	0.288114949	3.784844993	0.000153804	0.000511508
POP5	9.853783209	-1.843719973	0.487124552	-3.784904633	0.000153768	0.000511508
DNAJB4	8.962525783	1.794468826	0.474333452	3.783137831	0.000154864	0.000514902
ATP2B1	587.2933137	0.773480931	0.204459984	3.783043096	0.000154923	0.00051497
LOC121818794	2.126386448	4.415800119	1.167386646	3.782637169	0.000155176	0.000515683
PISD	34.4826301	-1.358716551	0.359212296	-3.782488982	0.000155268	0.000515862
P2RY6	2.701501082	4.173461606	1.103399391	3.782367148	0.000155344	0.000515986
RHOQ	7.428337081	1.982261929	0.524126591	3.782028929	0.000155555	0.000516559
ST3GAL4	5.991928718	2.363897984	0.62523758	3.780799586	0.000156325	0.000518988
EME2	28.27402622	1.168253244	0.309029532	3.780393534	0.000156581	0.000519706
LOC101112774	14.15341746	1.435576221	0.379797665	3.779844774	0.000156926	0.000520724
GCN1	100.5171913	0.865500646	0.22906765	3.778362612	0.000157863	0.000523702
TMEM71	2.15908151	4.432997099	1.173449101	3.777749793	0.000158252	0.000524862
BEX5	7.399842792	2.123518781	0.562474727	3.775314125	0.000159806	0.000529886
GABARAPL2	53.43535468	-1.121348396	0.297034881	-3.775140456	0.000159918	0.000530123
IST1	91.8829308	-0.648923375	0.171999366	-3.772824216	0.00016141	0.000534938
MAP4K3	61.73971883	-0.687128769	0.182151839	-3.772285656	0.000161759	0.000535962
ADD1	71.32561097	0.790438045	0.209566681	3.771773452	0.000162091	0.00053693
ABCG5	2.512989797	4.649388568	1.232763021	3.771518524	0.000162257	0.000537346
LAPTM4B	17.87670806	-1.775964932	0.470912306	-3.771328351	0.000162381	0.000537489
PPP1R2	25.96551876	1.015457479	0.26925485	3.771361886	0.000162359	0.000537489
RBM23	31.13140065	-1.044643939	0.277055507	-3.770522203	0.000162906	0.000539095
EID1	18.4010245	1.208730029	0.320586314	3.770373147	0.000163004	0.000539284
KHNYN	8.841278684	1.739615841	0.461403751	3.770268093	0.000163072	0.000539377
RFX2	4.730684856	2.839661041	0.7533199	3.769528775	0.000163556	0.000540844
SON	581.6072174	0.605963969	0.160756069	3.769462467	0.0001636	0.000540854
ADGRA2	4.433811768	3.339649331	0.886305099	3.768058354	0.000164522	0.000543632

BEX3	28.78272385	1.055224136	0.280049008	3.767998124	0.000164562	0.000543632
NAIP	2.028122906	4.373029092	1.160536977	3.768108365	0.000164489	0.000543632
TMEM175	4.678216295	3.01423188	0.800256116	3.766583995	0.000165496	0.000546584
NOVA1	3.699968515	3.524833457	0.936280445	3.764719723	0.000166736	0.000550542
LOC105605507	14.2174361	1.379392576	0.366408664	3.764628709	0.000166797	0.000550606
DHRS1	38.00578215	-1.017277672	0.270225736	-3.76454769	0.000166851	0.000550649
LOC105602261	1.960557059	4.289581455	1.140007536	3.762765875	0.000168045	0.000554451
CLK2	43.04296191	0.859178016	0.228390083	3.761888449	0.000168635	0.000556263
LOC114116187	1.991924159	4.35432442	1.157897852	3.76054279	0.000169545	0.000559126
CKS2	26.22001078	-1.313934143	0.349485561	-3.759623545	0.000170169	0.000561046
LOC101103836	14.94689154	-1.334636884	0.35502396	-3.759286788	0.000170398	0.000561663
C1H1orf50	65.98931311	0.857949082	0.228229216	3.75915536	0.000170488	0.00056182
CREM	12.91454212	1.719688453	0.45756045	3.758385266	0.000171013	0.000563413
PDLIM4	2.011586639	4.337597804	1.15444618	3.757297552	0.000171758	0.000565727
ZNF366	2.744176448	4.172648675	1.110692502	3.756799175	0.0001721	0.000566715
LOC132659633	1.97215844	4.322805498	1.150793665	3.75636887	0.000172397	0.00056755
MTMR10	54.76558834	0.959424683	0.255446454	3.755873951	0.000172738	0.000568533
SLC12A6	15.62395005	1.475797182	0.393042345	3.754804537	0.000173477	0.000570825
WDR36	14.70209319	-1.281461267	0.341342762	-3.754177357	0.000173912	0.000572115
CEBPD	104.748882	-0.732762656	0.195226042	-3.753406305	0.000174448	0.000573737
DYNLL2	65.01576156	-0.995268058	0.265175792	-3.753238751	0.000174564	0.00057398
LOC101103412	5.090095166	-2.780185406	0.7408126	-3.752886231	0.00017481	0.000574647
CCDC127	67.85487753	-0.852842768	0.227257332	-3.752762386	0.000174897	0.000574789
PNO1	22.89056386	-1.294070176	0.344869404	-3.752348459	0.000175186	0.000575598
CKAP2	14.39813598	-1.396545881	0.37221101	-3.752027331	0.00017541	0.000576194
ARHGAP21	96.79571937	-0.679398298	0.181124958	-3.75099216	0.000176136	0.000578437
LOC101112460	2.615534505	4.143785529	1.104815954	3.750656853	0.000176372	0.000578926
SLC13A4	25.37859468	-1.224880211	0.326572237	-3.750717526	0.000176329	0.000578926
ATP5PF	79.28923766	-0.696347689	0.185685867	-3.750138334	0.000176737	0.000579982
CENPM	5.801945679	-2.513661956	0.670416378	-3.749404157	0.000177255	0.00058154
LOC114115049	2.068211242	4.368433678	1.165663996	3.747592526	0.00017854	0.000585611
COL13A1	2.008147669	4.338810077	1.157863163	3.74725634	0.000178779	0.000586252
C1H3orf70	2.003349899	4.33647897	1.157557023	3.746233561	0.000179509	0.000588502
LOC101112162	8.191358424	-1.831520551	0.489003793	-3.745411748	0.000180098	0.000590287
PNPLA2	4.522713086	-2.947210032	0.787060496	-3.74457878	0.000180697	0.000592104
RBP4	15.91211815	1.304336154	0.348404065	3.743745512	0.000181297	0.000593926
CDC40	62.57765	0.873226482	0.233270663	3.743404637	0.000181544	0.000594442
REPS1	10.91835791	-1.568979751	0.419126353	-3.743452878	0.000181509	0.000594442
BID	52.75710953	0.936605651	0.250242686	3.742789317	0.000181989	0.000595754
LOC121817827	8.597821539	-1.754983148	0.468932904	-3.742503747	0.000182196	0.000596285
MYRIP	13.27404824	1.42262896	0.380148663	3.742296369	0.000182346	0.000596632
EEF1AKMT2	31.97016296	-1.005152004	0.268635152	-3.741699464	0.00018278	0.000597758

POLD2	7.227526636	-2.037274573	0.544469886	-3.741758039	0.000182737	0.000597758
FAM169A	2.032118416	4.372028174	1.168482425	3.741629381	0.000182831	0.000597779
LOC132658130	2.659300984	4.140250876	1.106619368	3.741350456	0.000183034	0.000598296
AP5Z1	47.25126665	-0.864309064	0.231044167	-3.740882431	0.000183375	0.000599265
OGT	100.5331425	-0.666956774	0.178303078	-3.740579139	0.000183597	0.000599842
SLC2A12	6.473942599	-2.167491088	0.579530244	-3.740082783	0.00018396	0.000600881
PKN2	68.65708492	0.799473052	0.213776574	3.739759861	0.000184196	0.000601506
PSMD6	29.01870205	-1.063620395	0.284429447	-3.739487616	0.000184396	0.000602011
LOC105606593	5.526593093	2.739486573	0.732737344	3.738702002	0.000184973	0.000603748
ARFGAP2	13.089814	1.652689013	0.442086242	3.738385988	0.000185205	0.000604359
NCOA7	50.23275641	0.753499433	0.201594128	3.737705252	0.000185707	0.00060585
RRP12	22.69642011	-1.669371189	0.446718993	-3.736960404	0.000186258	0.000607498
IKBIP	27.1674361	1.132965149	0.303372804	3.734563993	0.000188041	0.000613162
FSIP1	6.055388664	2.427867316	0.65014161	3.734366913	0.000188188	0.000613343
SEC31A	32.23019823	0.994577959	0.26632997	3.734382421	0.000188176	0.000613343
TRIM15	6.45138259	2.631262608	0.70474556	3.733634883	0.000188736	0.00061498
LOC114113672	1.926352601	4.275058458	1.145033309	3.733566896	0.000188787	0.000614996
ARF6	50.04680863	-1.225693951	0.328320114	-3.733228327	0.000189041	0.000615673
MEX3D	49.62663434	-0.794037685	0.212698733	-3.73315663	0.000189095	0.000615699
ZAR1L	100.6402024	0.659152776	0.176609656	3.732257852	0.000189771	0.00061775
ARMC8	57.76819985	0.713508867	0.191188849	3.731958579	0.000189997	0.000618334
EIF6	46.44059298	0.836054036	0.224041187	3.73169794	0.000190193	0.000618823
NLRC4	2.141526532	4.403651258	1.180174639	3.731355607	0.000190452	0.000619514
LRWD1	8.7768249	-1.833646561	0.49143351	-3.73122004	0.000190555	0.000619697
PDAP1	242.2098238	-0.664872129	0.178201639	-3.731010182	0.000190714	0.000620062
S100A8	2.048236005	4.333169552	1.161416754	3.730934256	0.000190771	0.000620099
PAAF1	89.55528842	0.749522703	0.200905942	3.730714458	0.000190938	0.000620489
PRR16	2.101296074	4.384247673	1.175485525	3.729733443	0.000191682	0.000622758
PLXDC1	2.115782209	4.447469204	1.192852512	3.728431772	0.000192675	0.000625755
SYT17	9.008300927	1.935085587	0.519012148	3.728401337	0.000192698	0.000625755
NUDT12	38.74337781	0.906075895	0.243252556	3.724836077	0.000195442	0.000634511
PEAR1	1.9516575	4.335892759	1.164240366	3.724224728	0.000195916	0.000635896
FBH1	30.8499027	1.04084669	0.279489467	3.724099879	0.000196013	0.000636056
LOC114108823	1.880096143	4.253796888	1.142466045	3.723346445	0.000196599	0.000637608
MKNK1	6.344158981	2.216331489	0.59525983	3.723300944	0.000196635	0.000637608
RAB11A	88.86559362	-0.686321907	0.184330715	-3.7233182	0.000196621	0.000637608
MAPK8	87.02289057	0.616708633	0.165674007	3.722422397	0.000197321	0.000639677
RYR3	3.76806979	4.01313702	1.078151818	3.722237402	0.000197465	0.00063999
ENTPD6	13.32207308	-1.43469265	0.385552009	-3.72113909	0.000198326	0.000642625
FASTK	13.54958748	-1.601108908	0.430366825	-3.720335342	0.000198958	0.000644517
CD226	2.313073424	4.557145265	1.225039987	3.719997155	0.000199225	0.000645225
INTS11	80.49622207	-0.722945526	0.194374718	-3.719339286	0.000199745	0.000646663

RABL2B	8.71528221	-1.796419553	0.482997784	-3.719312203	0.000199766	0.000646663
SLC9A1	9.24574029	1.689361792	0.454301891	3.718588512	0.000200339	0.000648361
LOC132658648	1.916157774	4.269832061	1.148295818	3.718407742	0.000200482	0.000648668
LYST	9.666414112	1.667133635	0.448662773	3.715783287	0.000202575	0.000655281
KANSL1	52.45430596	0.753220346	0.202739049	3.715220871	0.000203026	0.000656581
BRMS1	48.809386	-0.95857181	0.258018917	-3.715122214	0.000203106	0.000656678
ZC3H12D	2.607939096	4.035914109	1.086419854	3.714875142	0.000203304	0.000657161
GPS1	26.47724082	0.990654702	0.266758218	3.713680168	0.000204267	0.000660114
SLITRK5	1.893376372	4.26087999	1.147442056	3.713372687	0.000204515	0.000660757
PMPCA	42.00781358	-1.023164608	0.275544573	-3.713245365	0.000204618	0.00066093
HACD3	30.19440857	-0.959033911	0.258346547	-3.71219945	0.000205466	0.000663508
ALDH1L2	8.654047198	-1.810287405	0.487809711	-3.71105241	0.000206399	0.000666361
POLE4	46.7347418	-0.794737295	0.214182167	-3.71056707	0.000206795	0.000667479
ARCN1	34.38996334	0.900003372	0.242577299	3.710171459	0.000207119	0.000668361
FARSA	40.11000609	-0.981877128	0.264670894	-3.709803952	0.00020742	0.00066917
GIPC1	88.49229552	-0.824517393	0.222320179	-3.708693447	0.000208331	0.000671949
CA3	1.862574683	4.24448527	1.14521331	3.706283566	0.000210323	0.000678045
FBXW9	10.83382764	-1.585147378	0.42768907	-3.706307902	0.000210303	0.000678045
ARHGAP20	1.988309547	4.306959326	1.163298818	3.702367147	0.000213597	0.000688425
CAD	4.468288546	-2.721956957	0.735205111	-3.702309623	0.000213646	0.000688425
DNMBP	39.64148961	-0.963586068	0.260274394	-3.702193112	0.000213744	0.000688576
NEUROD2	3.031649865	-3.992908112	1.078739236	-3.701458126	0.000214364	0.000690407
NAA16	41.78938917	1.005969198	0.271789259	3.701283863	0.000214511	0.000690715
RBM15B	18.55974641	-1.157610894	0.312944611	-3.699091961	0.000216372	0.000696539
LIMD2	6.435413482	2.359105081	0.637948286	3.697956609	0.000217342	0.000699493
STK11IP	8.350366526	-1.833783476	0.495981384	-3.697282869	0.000217919	0.000701182
ISLR	2.08145527	4.377199708	1.184175858	3.69641019	0.00021867	0.000703427
UBE2G2	42.06640448	1.095187382	0.296318461	3.695980937	0.000219039	0.000704447
CCDC82	58.7113703	-0.794111378	0.21487511	-3.695688062	0.000219292	0.00070509
GIT1	42.88449692	-0.912814395	0.247036382	-3.695060579	0.000219834	0.000706663
DOK5	7.373327801	-2.215543792	0.599661441	-3.69465775	0.000220183	0.000707614
JAM3	2.080137679	4.392406718	1.18953303	3.69254708	0.000222019	0.000713343
CCDC91	52.09237372	0.908168582	0.245950853	3.692479902	0.000222078	0.000713361
UBE2N	26.11278496	-1.168000737	0.316355644	-3.692049626	0.000222454	0.000714397
SAFB2	161.646481	0.617966507	0.167387624	3.691829147	0.000222647	0.000714845
DSG2	81.76278991	-0.714371358	0.193533199	-3.691208339	0.000223191	0.00071642
TSPAN17	31.2505695	-0.945410218	0.256133493	-3.69108393	0.0002233	0.000716599
PGR	2.587155714	4.129160216	1.11897604	3.690123889	0.000224145	0.000719136
SEC22C	9.724843551	1.633388108	0.442869411	3.688193556	0.000225852	0.000724439
CLCN2	8.693634015	-1.798554171	0.487693452	-3.68787845	0.000226132	0.000724988
LOC132659300	3.956492759	3.215809687	0.871982125	3.687930744	0.000226085	0.000724988
SNRNP27	9.833523954	-1.669990674	0.452908695	-3.687256818	0.000226685	0.000726587

MSH2	55.09709282	-0.710597357	0.192780201	-3.686049466	0.000227762	0.00072969
PA2G4	908.0013638	-0.679469973	0.184333437	-3.686091816	0.000227724	0.00072969
BAD	6.973884891	-2.035161844	0.552158948	-3.685826066	0.000227962	0.000730156
LOC121818696	3.745360773	3.437287564	0.932698324	3.685315472	0.00022842	0.000731446
LRCH1	21.66110737	1.206311948	0.327336146	3.685239046	0.000228488	0.00073149
GM2A	46.46246097	0.835671311	0.226780576	3.684933362	0.000228762	0.000732193
USP24	74.43016028	-0.675781072	0.183428412	-3.684167915	0.000229451	0.000734221
LSM14B	23.22855352	-1.0517641	0.285536527	-3.683466041	0.000230084	0.00073607
LOC101123627	1.845891684	4.238653655	1.150916261	3.68285148	0.00023064	0.000737671
CADPS2	59.42159101	0.78148689	0.212200921	3.6827686	0.000230715	0.000737735
ABCE1	98.60194385	-0.633543476	0.17203763	-3.68258663	0.000230879	0.000737852
ANKZF1	34.95198878	1.093895472	0.297053665	3.682484347	0.000230972	0.000737852
KCTD7	13.76650174	-1.340006628	0.363871674	-3.682635182	0.000230835	0.000737852
PDE12	42.26581297	0.782050646	0.21236985	3.682493744	0.000230964	0.000737852
BMAL1	6.679576351	2.094150468	0.568733964	3.682126616	0.000231296	0.000738712
CYLD	22.80294977	1.091346845	0.296403836	3.681959252	0.000231448	0.00073902
FAM234B	9.596519118	-1.734883954	0.471259179	-3.681379655	0.000231975	0.000740526
DPY19L4	29.4931155	1.162039839	0.315659336	3.681309906	0.000232039	0.000740552
TIPRL	70.66504351	-0.721240315	0.195927143	-3.681165893	0.00023217	0.000740793
PDE8A	78.84667887	0.730984689	0.198590524	3.68086389	0.000232445	0.000741494
GALNT10	22.32203537	1.459050283	0.396422731	3.680541427	0.000232739	0.000742255
ZNF574	16.45727135	-1.22049121	0.33164643	-3.680097539	0.000233145	0.000743371
ZNF174	5.510739317	-2.331197507	0.633569528	-3.679465953	0.000233723	0.000745037
WIZ	15.14710364	-1.533265862	0.41685286	-3.678194413	0.000234891	0.000748582
GALNT7	190.3594246	0.635722925	0.172858775	3.677701214	0.000235345	0.000749851
BLOC1S6	29.06699921	0.984670693	0.267756264	3.677488923	0.000235541	0.000750297
POLR2H	40.45630966	-0.851921334	0.231675692	-3.677215013	0.000235794	0.000750924
DAG1	93.45452644	0.747552912	0.203378869	3.675666581	0.000237229	0.000755077
PINX1	41.3987556	0.987777955	0.268737413	3.675624999	0.000237268	0.000755077
TARBP2	11.28666354	-1.491067751	0.405652902	-3.675723122	0.000237177	0.000755077
NDUFB8	5.70109667	-3.537616105	0.962579478	-3.675141832	0.000237717	0.000756192
SYTL3	1.939986399	4.280712931	1.164779719	3.675126601	0.000237732	0.000756192
VGLL3	8.337156363	1.740205503	0.473604997	3.674381633	0.000238426	0.000758221
RYK	47.80245502	0.832000139	0.226509437	3.673136764	0.000239591	0.000761745
DOK2	1.9005582	4.265156393	1.161201912	3.673053194	0.00023967	0.000761813
NR6A1	18.00560624	1.234826393	0.336193047	3.672968266	0.000239749	0.000761885
MARCKSL1	8.119762875	-2.061762136	0.561413597	-3.672447814	0.000240238	0.000763257
ENOX2	41.07457333	0.956669157	0.260506873	3.672337499	0.000240342	0.000763406
LOC105606855	8.320606777	-2.003408244	0.545588372	-3.672014193	0.000240646	0.000764191
PGAP1	15.44638187	-1.32919159	0.362016593	-3.671631678	0.000241007	0.000765154
PDGFRB	3.09944551	3.74155586	1.019149397	3.671253567	0.000241364	0.000766105
LETM1	52.39313801	0.853157751	0.232441207	3.670423854	0.000242149	0.000768414

ENPEP	2.186867517	4.468482847	1.217873683	3.669085644	0.00024342	0.000772263
GTSE1	12.37097428	-1.470000491	0.400743013	-3.66818745	0.000244276	0.000774797
NUDCD3	35.33338846	0.925016837	0.252202107	3.667760141	0.000244685	0.000775908
WWC2	54.19848281	-0.765917241	0.208838288	-3.667513506	0.000244921	0.000776473
RPSA	150.0945439	-1.018619579	0.277802707	-3.666701408	0.000245699	0.000778757
LOC132658472	11.37023491	-1.713555702	0.467509081	-3.665288596	0.00024706	0.000782883
PRPF4	13.35969763	-1.312365929	0.358092929	-3.664875301	0.000247459	0.000783963
GAR1	89.20789173	-0.749155623	0.204431369	-3.664582536	0.000247742	0.000784674
TTC14	151.0706719	-0.664985743	0.181523761	-3.663353703	0.000248934	0.000788263
RFX3	12.71154783	-1.378501253	0.376350439	-3.66281293	0.000249461	0.000789742
USP27X	7.634774211	-1.889704392	0.515964845	-3.662467336	0.000249798	0.000790622
SSH2	29.49471918	0.980481299	0.267719421	3.662346559	0.000249915	0.000790807
FGFR2	35.93971208	0.866366235	0.236699259	3.660198344	0.00025202	0.000797278
MAPK1IP1L	78.45649917	0.710026946	0.194013867	3.659671129	0.000252539	0.000798731
NOP9	17.61349218	-1.162297448	0.31774237	-3.657986971	0.000254204	0.000803807
DNM3	1.967223555	4.317555462	1.180508657	3.657368742	0.000254818	0.000805557
PTGER2	15.86560168	1.305872017	0.357140496	3.656465826	0.000255716	0.000808207
FBRS	25.49802943	-1.294441161	0.354157224	-3.65499014	0.000257192	0.000812678
SETX	140.8308343	0.565944924	0.154851345	3.654762721	0.00025742	0.000813206
RSRC1	111.867552	-0.6926226	0.189515555	-3.654700532	0.000257482	0.000813211
PHF3	132.3994033	0.572554269	0.156692816	3.653991826	0.000258194	0.000815268
DGKA	13.49625857	1.428153976	0.390952554	3.65301099	0.000259183	0.000818196
LOC101108528	38.76482501	1.036241778	0.283713274	3.652426143	0.000259774	0.000819869
F11R	31.67719527	-1.103506737	0.302161294	-3.652045316	0.00026016	0.000820892
MSTO1	3.493812117	-3.576462899	0.979480849	-3.651386246	0.000260829	0.000822808
TFIP11	11.23142544	-1.500930505	0.411079744	-3.65119062	0.000261027	0.000823046
ZKSCAN1	204.3250877	0.519048572	0.142158564	3.651194526	0.000261023	0.000823046
RNF113A	10.32742279	-1.585479887	0.434387143	-3.649923609	0.000262318	0.000826921
RAD21L1	9.622277946	1.772905936	0.485747674	3.649849564	0.000262394	0.000826965
FTH1	13.97749181	-2.097249764	0.574740836	-3.649035589	0.000263227	0.000829394
MEGF6	3.016688492	3.774153254	1.034807455	3.647203387	0.00026511	0.000835131
GNLY	1.95982452	4.313492393	1.182858599	3.646667824	0.000265663	0.000836676
UTP25	13.42578952	-1.363972446	0.374047436	-3.646522646	0.000265813	0.000836951
AAMP	161.5448382	-0.619724837	0.170007165	-3.645286583	0.000267094	0.000840786
PLCE1	5.860609971	2.246434593	0.6163332	3.644837878	0.00026756	0.000842056
ARL14EP	37.600814	-0.865599701	0.237518194	-3.644351136	0.000268067	0.000843453
HECW2	3.855212856	3.147423721	0.863807595	3.643662941	0.000268785	0.000845513
ANGPTL2	1.920287718	4.297605037	1.179948763	3.642196314	0.000270322	0.00085009
DPP4	29.5779924	1.440787377	0.395606475	3.641971172	0.000270558	0.00085009
LOC105603384	14.6899751	-1.286090371	0.353126847	-3.642006783	0.000270521	0.00085009
TIFA	1.786878018	4.180761804	1.147902184	3.642088901	0.000270435	0.00085009
TSPAN9	8.376000241	-1.738774708	0.477419005	-3.642030777	0.000270496	0.00085009

LOC132660160	2.469200543	4.027784806	1.106114187	3.641382465	0.000271178	0.000851836
BRD4	189.9198083	0.621611678	0.170725512	3.641000513	0.000271581	0.000852901
GNA15	2.439687933	3.995426885	1.097399958	3.640811954	0.00027178	0.000853325
RASGRP2	1.885283682	4.253779026	1.168456874	3.640510079	0.000272098	0.000854126
ABI3	1.935745169	4.279027846	1.175614139	3.639823395	0.000272825	0.000856206
HOMER2	65.21068666	-1.206652773	0.331560506	-3.639313944	0.000273365	0.0008577
CRKL	39.48364134	-0.812507601	0.223286089	-3.6388635	0.000273844	0.000859
STARD13	67.89248765	0.744148454	0.204508163	3.63872249	0.000273994	0.000859268
SCUBE2	3.089496277	3.712034396	1.020183425	3.638595085	0.000274129	0.000859492
ELAPOR1	59.4449001	0.852968059	0.234443658	3.638264589	0.000274481	0.000860393
ANKS4B	16.77960072	1.384266877	0.380528455	3.637748656	0.000275032	0.000861916
THADA	14.45074779	1.35099752	0.371399699	3.637583777	0.000275208	0.000862266
CCDC124	36.67810991	0.977380323	0.268699517	3.637447259	0.000275354	0.000862521
TFPI2	14.21339086	1.387477784	0.381510118	3.636804684	0.000276041	0.000864472
TPM4	73.56110866	0.670450875	0.184400053	3.635849688	0.000277066	0.000867478
SRPK1	60.92983854	-0.746471871	0.205354528	-3.635039743	0.000277938	0.000870004
UTP6	20.75719687	-1.033594247	0.284355062	-3.634871982	0.000278119	0.000870366
C3H12orf75	50.44013561	-0.870545741	0.239538127	-3.63426797	0.000278771	0.000872203
LOC132657851	32.1976091	0.920710892	0.253349588	3.634151926	0.000278896	0.000872392
PHF2	30.29560697	0.90917679	0.250210094	3.63365352	0.000279436	0.000873875
LOC106991845	4.993276356	-2.50142555	0.688459899	-3.633364201	0.00027975	0.000874651
PRKAB2	52.27218657	0.809693931	0.222927	3.63210348	0.00028112	0.000878731
EHF	134.9154594	0.623844126	0.171796018	3.631307254	0.000281989	0.000881241
LOC114117087	8.371679622	1.971475985	0.542931448	3.631169263	0.00028214	0.000881506
LOC132660247	13.11486261	1.575686735	0.433957155	3.630973053	0.000282355	0.000881971
ZFPM2	3.720629775	3.404559403	0.938018825	3.629521403	0.000283947	0.000886738
FZD4	10.03868542	1.592200138	0.438749026	3.628954237	0.000284572	0.000888481
TRMU	7.20157405	-2.176331168	0.599850072	-3.628125207	0.000285487	0.00089113
LOC105602786	3.055704125	-3.928703062	1.083751979	-3.625094245	0.000288856	0.000901273
SLC9A7	3.753810302	3.188409324	0.879541594	3.625080775	0.000288871	0.000901273
ANKRD40CL	1.945976198	4.285222842	1.182786481	3.622989365	0.000291218	0.000908382
GPD2	50.60625865	0.766880392	0.211725053	3.622057861	0.000292269	0.000911235
RAMP3	2.026719035	4.329214332	1.195233442	3.622065934	0.00029226	0.000911235
TUSC1	5.636705102	-2.325109184	0.642203522	-3.620517648	0.000294014	0.000916463
ETFA	39.4423758	-0.958898137	0.26490294	-3.61980934	0.00029482	0.000918761
DNAJC9	48.40154972	-0.830974819	0.229596283	-3.619286895	0.000295416	0.000920391
MTUS2	1.852452258	4.238730373	1.171169081	3.619230087	0.000295481	0.000920391
AGAP1	52.78783455	-0.761459784	0.210404153	-3.619034	0.000295705	0.000920874
C12H1orf174	3.900785235	-3.815033929	1.054232414	-3.618778819	0.000295996	0.000921567
LEAP2	12.87684852	-1.353974053	0.374224684	-3.618077882	0.000296799	0.000923636
RBM41	38.6578624	0.929545517	0.256915896	3.618092655	0.000296782	0.000923636
TMEM242	15.76734846	-1.341948536	0.371291062	-3.614276437	0.000301188	0.000937075

GNG12	149.6895885	0.63429319	0.17550761	3.614049499	0.000301451	0.000937678
LOC101116441	2.480984493	3.95179774	1.093567256	3.613675993	0.000301886	0.000938812
JMY	24.40171158	1.1018935	0.304950921	3.613347019	0.00030227	0.000939785
LOC101113195	4.060407025	-2.994494404	0.828824883	-3.612939798	0.000302745	0.000941044
SMIM19	128.1906175	-0.615215388	0.170301276	-3.612511921	0.000303245	0.00094238
FUT2	16.32342187	1.229771457	0.34043411	3.61236263	0.00030342	0.000942703
FGR	3.091133685	3.754193294	1.039580028	3.611259541	0.000304714	0.000946503
ALG12	18.1375957	1.206908936	0.334347665	3.609742376	0.000306501	0.000951835
SAP130	18.83077675	-1.216539836	0.337073949	-3.609118527	0.000307239	0.000953905
VSIG10	30.10951881	1.076359727	0.298289906	3.608434966	0.00030805	0.000956199
ZBTB22	8.349615813	-1.705878269	0.472766202	-3.608291501	0.00030822	0.000956506
MDK	5.933535136	-2.254784713	0.625037514	-3.607439014	0.000309234	0.00095943
FAM234A	16.57490902	1.211935725	0.336077544	3.606119323	0.00031081	0.000964096
IDH3B	19.8194563	-1.34850524	0.37397141	-3.605904632	0.000311067	0.000964463
UBE2O	22.38129833	-1.017450786	0.282162785	-3.605899995	0.000311073	0.000964463
CHD6	70.8716234	0.973621846	0.270114821	3.604473993	0.000312786	0.000969549
CORO1C	19.67742666	1.091234363	0.30275984	3.604290328	0.000313007	0.000969733
LOC101113001	63.39417661	-0.987312593	0.273935131	-3.604183911	0.000313135	0.000969733
MUSTN1	1.963500339	4.271242718	1.185074004	3.604199149	0.000313117	0.000969733
TOMM6	55.1469942	-0.779837633	0.21636555	-3.604259699	0.000313044	0.000969733
KCNK17	1.982667953	4.347251757	1.206220579	3.604027185	0.000313324	0.000970093
STX17	82.72905402	0.892177105	0.247588371	3.603469347	0.000313998	0.000971953
MFAP5	1.819403627	4.22345797	1.172173134	3.603100812	0.000314444	0.000973108
CFAP46	3.201028621	3.755766633	1.042491956	3.602681643	0.000314951	0.000974453
APH1B	3.369902672	3.385845889	0.939921164	3.602265826	0.000315456	0.000975788
BCKDK	9.151064626	-1.629581782	0.452401082	-3.602073136	0.00031569	0.00097606
STAR	1.756914789	4.167476471	1.156964722	3.602077395	0.000315684	0.00097606
TAGLN2	20.31125862	-1.270590011	0.352982127	-3.599587382	0.000318723	0.000985209
MLLT1	58.53150553	-0.874225883	0.242930506	-3.598666534	0.000319853	0.000988475
SNRPA1	8.326520669	-1.752918201	0.48727347	-3.597401271	0.000321412	0.000993065
CTSK	5.841433028	2.381062112	0.661929569	3.597153267	0.000321719	0.000993782
LOC114110099	9.444343937	-1.651424993	0.459114084	-3.596981778	0.000321931	0.000994208
FOXC1	2.24424206	-4.247061655	1.181002441	-3.596149769	0.000322962	0.00099693
TMCO3	10.08368419	-1.843872553	0.512727084	-3.596206661	0.000322891	0.00099693
GRHL2	23.25901199	-1.146851488	0.318988639	-3.59527377	0.000324051	0.00100006
RAP2B	50.10338142	0.898849299	0.250037222	3.594861958	0.000324564	0.001001412
MYO1E	20.35014181	-1.140991824	0.317411911	-3.594672361	0.0003248	0.00100191
LOC101122274	2.379031619	3.972314404	1.105117902	3.59447114	0.000325051	0.001002454
GMIP	20.8958025	1.157776938	0.322160141	3.593793237	0.000325898	0.001004835
ATP11C	50.40605929	0.970854896	0.270246344	3.592481153	0.000327544	0.001009677
CCDC71	29.93543983	-0.91944185	0.255992777	-3.591671068	0.000328564	0.001012587
MAP4K5	30.46469283	-1.052697611	0.293166909	-3.590779108	0.000329691	0.001015753

STK38L	42.55233785	0.818679767	0.227997654	3.590737674	0.000329743	0.001015753
RPS8	1205.876996	-0.488713385	0.136120265	-3.590305865	0.00033029	0.001016968
SOD1	25.57195222	-1.239484648	0.345228879	-3.590327233	0.000330263	0.001016968
BTBD8	28.04427837	0.954378083	0.265836618	3.590092635	0.00033056	0.001017566
ZFYVE1	21.68005875	-1.122851057	0.312805985	-3.589608605	0.000331175	0.001019222
PIKFYVE	16.01905605	1.184897613	0.330109152	3.58941158	0.000331425	0.001019758
ZNF292	344.793681	0.636026903	0.177237	3.588567295	0.0003325	0.00102283
ENTPD7	17.57057379	1.290914711	0.359869421	3.587175338	0.00033428	0.001028067
HDAC7	201.5756351	-0.684898213	0.190998827	-3.585876536	0.000335948	0.001032961
LOC121819155	43.23726018	1.154641615	0.322009789	3.585734514	0.000336131	0.001033285
PPP1R18	25.1035447	1.240775256	0.346037655	3.585665424	0.00033622	0.001033321
PARP9	21.05281299	1.150811441	0.320969372	3.585424472	0.00033653	0.001034038
PKN1	13.21166787	1.510425494	0.421533866	3.583165234	0.000339456	0.001042787
SYCE1L	4.738285244	2.977249327	0.830976836	3.582830709	0.000339891	0.001043884
FBLN5	1.76637976	4.169297165	1.164020548	3.581807188	0.000341226	0.001047501
TRAF1	44.91141066	0.850695233	0.237501351	3.581854288	0.000341164	0.001047501
FOSL2	149.8147797	0.694679731	0.193977256	3.581243209	0.000341963	0.001049284
LOC121816702	4.05418116	3.117246366	0.870425669	3.581289568	0.000341902	0.001049284
OBI1	13.90429114	-1.438594665	0.401785921	-3.580500435	0.000342937	0.00105203
ZNF784	9.300367669	-1.703066751	0.475767712	-3.579618177	0.000344097	0.001055345
BAP1	30.59888967	-0.928090067	0.259326431	-3.578848725	0.000345111	0.001058214
LOC101121216	72.52817354	2.217171637	0.619711917	3.577745687	0.00034657	0.001062445
OPA1	75.60545798	0.662233759	0.1851065	3.577582415	0.000346787	0.001062865
LOC105615175	2.313243848	3.930035102	1.098607351	3.577288189	0.000347177	0.001063818
DMXL2	5.431367351	2.369981235	0.66252401	3.577200522	0.000347294	0.00106393
RPL29	637.5548689	-0.66197915	0.185084155	-3.576638696	0.000348041	0.001065974
JAGN1	19.10230989	-1.344497916	0.375978193	-3.575999726	0.000348892	0.001068337
ZRANB2	108.633114	-0.640279142	0.179056232	-3.575855104	0.000349085	0.001068683
TAF15	35.52987959	-0.866782599	0.242410946	-3.575674343	0.000349326	0.001069177
MRPL12	47.50501987	-0.773492316	0.216362405	-3.574984837	0.000350248	0.001071753
C8H6orf120	32.2913543	-0.906329147	0.253563422	-3.57436865	0.000351074	0.001074034
GAA	57.85086906	-1.176586405	0.329404498	-3.571858956	0.000354456	0.001084133
G0S2	10.38123645	-1.586690836	0.44435146	-3.570801443	0.000355891	0.001088271
SLA2	1.826657857	4.227074648	1.18405002	3.570013577	0.000356963	0.0010913
FAM76B	28.52056844	-0.901096021	0.252423842	-3.569773819	0.00035729	0.00109205
ERCC8	54.21802654	0.678518105	0.190089863	3.569459694	0.000357718	0.00109286
TMBIM1	29.60739817	-1.118926733	0.313468567	-3.569502181	0.00035766	0.00109286
FFAR4	8.156590802	-1.872139433	0.524507984	-3.569324947	0.000357902	0.001093172
LOC114116113	2.170883697	-4.049579631	1.134645869	-3.569025139	0.000358312	0.001094174
GTPBP2	25.11910636	1.134484962	0.317919565	3.568465386	0.000359078	0.001096263
LAYN	1.782149968	4.205225844	1.178671452	3.567767622	0.000360036	0.001098935
INSM1	4.800587024	-3.048004809	0.854390403	-3.567461432	0.000360456	0.001099968

NPC2	44.00531982	-0.860787319	0.241334449	-3.566781792	0.000361392	0.001102572
CAV2	41.4105633	0.967086896	0.271191837	3.566061972	0.000362386	0.001105351
LOC121819267	4.789679493	2.70001081	0.757163718	3.565953764	0.000362535	0.001105555
GOLGA5	38.95578304	0.864486368	0.242460116	3.565478649	0.000363193	0.001107055
NEFL	2.438176453	3.978740374	1.11589638	3.565510602	0.000363148	0.001107055
ORC1	6.645463565	-2.35083218	0.659403852	-3.565087123	0.000363735	0.001108456
MAP7	173.3046789	-0.588530366	0.165154765	-3.563508241	0.000365931	0.001114893
LOC114115685	6.656573768	-2.159170587	0.605948986	-3.563287731	0.000366239	0.001115576
KDSR	46.51261044	-0.751689419	0.210978146	-3.562878107	0.000366811	0.001117065
NSG1	1.803135046	4.190270312	1.176186065	3.56259136	0.000367212	0.001118031
SLC50A1	22.43305242	1.017666245	0.285730404	3.561630934	0.000368558	0.001121874
CCDC43	34.77993242	1.233397636	0.346387234	3.560747959	0.0003698	0.001125397
SLC16A3	4.289194858	-2.931827009	0.823422964	-3.560535881	0.000370099	0.00112605
ARPP19	41.18987006	-0.861369205	0.241972445	-3.559782212	0.000371162	0.00112903
UPF3B	216.3701353	0.875257863	0.245885706	3.55961262	0.000371402	0.001129502
SPECC1	12.26563616	-1.326041622	0.372592691	-3.558957684	0.00037233	0.001132065
ELP5	18.11473749	-1.183003618	0.332410949	-3.558858767	0.00037247	0.001132233
ELP1	18.71577413	-1.129754585	0.317462437	-3.55870318	0.00037269	0.001132647
ZNF618	110.9944546	0.552925997	0.1553781	3.558583839	0.00037286	0.001132904
SLC25A46	42.74719931	0.846140872	0.237798978	3.558219124	0.000373378	0.00113422
MOGS	19.66550438	1.227171874	0.344899443	3.558056985	0.000373608	0.001134404
TMC6	9.498698226	-1.570876252	0.441493079	-3.558099379	0.000373548	0.001134404
CPEB1	2.451280726	3.984617221	1.120104629	3.557361623	0.000374598	0.001137152
RELA	23.2353462	-1.630832794	0.458467572	-3.557138812	0.000374916	0.001137858
BLTP3A	12.57066319	-1.32740688	0.373186141	-3.556956529	0.000375176	0.001138131
PCK1	2.065448214	4.385335227	1.23288316	3.556975526	0.000375149	0.001138131
PSMD11	150.728283	0.732917603	0.206074205	3.556571289	0.000375727	0.001139542
SYCP3	2.022027186	4.349532251	1.223085165	3.556197372	0.000376261	0.001140905
APOBEC3F	6.619999855	1.946483133	0.547719842	3.553793352	0.000379717	0.001151123
TERF2IP	207.1181375	0.632855787	0.178096686	3.553439434	0.000380229	0.001152412
TIMM17A	26.29689757	-0.953803343	0.268441122	-3.553119338	0.000380692	0.001153554
CHMP1A	63.50125275	0.951329788	0.267799188	3.552399823	0.000381734	0.001156451
RPS6KA5	33.38352479	1.154016789	0.324878111	3.552153101	0.000382092	0.001157273
RAMAC	28.27510879	-1.055051355	0.297111141	-3.551032631	0.000383723	0.001161948
NAA35	152.2555474	-0.594317583	0.167376074	-3.550791761	0.000384074	0.001162749
MTDH	655.9466909	0.456889875	0.128675951	3.550701363	0.000384206	0.001162885
NUAK1	5.300897091	2.264177905	0.637746024	3.550281489	0.000384819	0.001164477
S100B	1.89501902	4.238593878	1.194161229	3.549431832	0.000386063	0.001167977
ZAR1	1.785580068	4.15121425	1.169718255	3.548900972	0.000386843	0.00117007
IPMK	75.11817346	0.709018574	0.199794867	3.548732676	0.00038709	0.001170553
TAF13	82.55931462	0.70847449	0.199645688	3.54865911	0.000387198	0.001170615
RNF217	38.02748459	-0.80917441	0.228068836	-3.547939399	0.000388258	0.001173553

LOC105616742	597.1776245	-0.500963697	0.141204612	-3.547785644	0.000388484	0.001173973
SP6	2.382625815	-4.138028377	1.166459471	-3.54751149	0.000388889	0.001174929
REPIN1	16.92055134	-1.169165824	0.329607099	-3.547150009	0.000389423	0.001176277
ADGRE5	13.13632988	1.57262349	0.4433856	3.546852873	0.000389862	0.001177338
VPS26C	31.92844525	-0.895524575	0.252638834	-3.544682978	0.000393086	0.001186804
LOC101111805	1.907641795	4.243271738	1.197441184	3.543615999	0.00039468	0.001191079
MICAL2	22.03790028	1.102926981	0.311242412	3.543626896	0.000394663	0.001191079
LOC101116157	2.494757731	3.99970473	1.129083231	3.542435687	0.00039645	0.001196152
POLK	15.93812493	-1.216308115	0.343411586	-3.541837743	0.00039735	0.001198596
LOC101102587	4.929619689	2.72425001	0.769270442	3.541342371	0.000398097	0.001200578
FBXW11	15.95602291	-1.343857773	0.379530386	-3.540843688	0.00039885	0.001202578
WDR13	31.09538464	-1.035269438	0.292385245	-3.540771826	0.000398958	0.001202634
ITPRID2	52.37130335	0.823026455	0.232456324	3.540563841	0.000399273	0.001203311
LOC101102230	1.842987288	4.235937719	1.196475804	3.540345493	0.000399604	0.001204035
PRR5	6.757642511	1.962323163	0.554540202	3.538649055	0.00040218	0.001211526
LOC121818772	4.695236628	2.381046003	0.672943736	3.538254205	0.000402782	0.001213066
SLC2A6	3.885210527	3.07309327	0.868654813	3.537761173	0.000403535	0.001214831
ZNF420	13.39132471	-1.309185188	0.370061401	-3.537751258	0.00040355	0.001214831
LRRC69	6.683892037	-1.915399724	0.541467262	-3.537424808	0.000404049	0.00121606
SLC10A7	29.35161022	0.874990648	0.247359089	3.537329684	0.000404195	0.001216224
KDELR3	9.148029538	1.990238033	0.562678656	3.537077536	0.000404581	0.001217112
HRCT1	4.587367233	-2.500137011	0.706859605	-3.536964051	0.000404755	0.001217361
LRRC8C	4.688073413	2.57016657	0.727060233	3.535011894	0.000407757	0.001226114
TIMM17B	15.94605377	1.463521399	0.414113191	3.534109588	0.000409151	0.001230031
LPCAT2	1.798860297	4.159810879	1.177156209	3.53377984	0.000409662	0.00123129
GPRIN2	2.097809256	-3.998639748	1.131592019	-3.533640818	0.000409878	0.00123166
PRRX2	1.75141181	4.13453191	1.170146728	3.533344844	0.000410337	0.001232763
RAP1GAP2	18.92223653	1.166033819	0.330034683	3.533064495	0.000410772	0.001233794
LOC121820657	3.257185367	-3.478149865	0.984529516	-3.532804055	0.000411177	0.001234732
TPK1	15.06773602	-1.247496187	0.353137382	-3.5326087	0.000411481	0.001235367
ABTB2	7.671210946	-1.7812187	0.504253998	-3.532383892	0.000411831	0.001235863
BRWD1	129.3086738	0.570879282	0.161610742	3.532434005	0.000411753	0.001235863
TM6SF1	2.405830349	4.009037289	1.135059831	3.532005257	0.000412421	0.001237356
ZNF592	5.734813072	-2.089456147	0.591647766	-3.531587992	0.000413072	0.001239032
DEPDC1	17.80534426	-1.204927421	0.341203696	-3.531402018	0.000413363	0.001239625
TMEM158	6.900996458	1.978788112	0.560402096	3.531014829	0.000413969	0.001241163
ADRA1A	1.946397558	4.331012294	1.226766694	3.53042866	0.000414887	0.001243637
RNF167	34.32267638	0.84469567	0.239278957	3.530171146	0.000415291	0.001244569
SLC16A14	2.327682272	3.958559263	1.121463447	3.529815684	0.000415849	0.001245963
BNIP5	13.48680498	-1.311301773	0.371650163	-3.528322877	0.000418202	0.001252731
EFS	1.724957272	4.148378998	1.17592541	3.527756917	0.000419097	0.001255131
TMC8	6.744510026	-2.042188159	0.578994387	-3.52712946	0.000420091	0.001257827

CFTR	29.41078838	-0.953193282	0.270377608	-3.52541502	0.00042282	0.001265713
LOC101103602	1.74062156	4.157536557	1.179366816	3.525227689	0.000423119	0.001266325
NELFCD	144.9440924	-0.64760532	0.183727707	-3.524810322	0.000423786	0.001268038
TNKS1BP1	12.38996065	-1.402451429	0.39793907	-3.524286843	0.000424624	0.001270262
TAB3	13.96401113	1.23940764	0.351726736	3.523780005	0.000425437	0.001272409
ANKRD13C	10.91186029	1.555865398	0.441640998	3.522918851	0.000426822	0.001276265
TRHDE	1.997278827	4.296407231	1.219783328	3.522270826	0.000427867	0.001279103
VAV1	2.288395757	3.919179677	1.113065315	3.52106891	0.000429811	0.001284627
LOC121819072	2.343450114	3.96615014	1.126547539	3.520623855	0.000430533	0.001286497
MTMR8	27.88040621	1.070093539	0.303965766	3.520440974	0.00043083	0.001287097
SNX21	12.49582072	1.563927863	0.444360697	3.519500877	0.00043236	0.001291378
PCNT	64.47582762	0.86080589	0.244605935	3.519153735	0.000432926	0.001292781
TP53BP2	19.07233187	-1.069309784	0.303933294	-3.518238393	0.000434422	0.001296959
LDOC1	3.487611076	3.352458405	0.952922446	3.518081058	0.00043468	0.001297438
LOC101105495	9.449184643	1.736090481	0.493570125	3.517414031	0.000435773	0.001300413
ANXA6	4.852837803	2.620958651	0.745219876	3.517027302	0.000436409	0.001302018
LY6E	7.436429184	-2.061722076	0.586430377	-3.515715003	0.000438571	0.001307942
PTDSS1	10.21130237	-1.481959578	0.421525723	-3.515703783	0.00043859	0.001307942
DDX42	156.140281	0.68291388	0.194300882	3.514723523	0.000440212	0.001312486
SRSF9	18.63604651	-1.309516749	0.372617537	-3.514372298	0.000440795	0.00131393
GLRX2	18.1381655	-1.135872654	0.32322373	-3.514199449	0.000441081	0.001314199
VPS72	9.728197595	-1.591126039	0.452763043	-3.51425776	0.000440985	0.001314199
CRYBG1	42.52078171	0.864009817	0.245899647	3.513668391	0.000441964	0.001316535
ZNF444	12.36468528	1.340462223	0.381511665	3.513555014	0.000442153	0.001316804
SLC5A12	7.045409107	1.916327014	0.545433405	3.513402364	0.000442407	0.001317267
ZNF598	39.38631318	-0.90171757	0.256733745	-3.512267435	0.000444301	0.001322611
SLC25A19	36.65551646	-1.03531249	0.294776737	-3.512191982	0.000444427	0.001322692
LOC132657612	2.416973853	4.028428627	1.147168115	3.511628831	0.00044537	0.001325202
MRE11	32.43591577	0.945000752	0.269152434	3.511024355	0.000446383	0.001327924
PTGFRN	20.67068795	1.07326472	0.305734446	3.5104475	0.000447353	0.001330512
LOC101115536	34.60312473	0.963520146	0.274499785	3.510094356	0.000447948	0.001331984
LOC132659417	7.984952152	1.818372792	0.518149993	3.509356009	0.000449193	0.001335391
VIPR1	4.147781835	2.86730496	0.817426921	3.507720247	0.000451964	0.001343329
C15H11orf58	180.3537847	0.527818157	0.150491089	3.507305058	0.00045267	0.001345128
CD55	67.55656554	1.138700117	0.324671295	3.507239892	0.000452781	0.001345158
TOM1L1	30.92553262	-0.865561785	0.246803787	-3.507084705	0.000453045	0.001345644
GNPNAT1	23.5697083	0.989450553	0.282182607	3.506419353	0.000454179	0.001348713
RASAL2	22.5901937	1.089991377	0.310931546	3.505567032	0.000455636	0.001352738
C1H3orf85	4.268019231	2.709812994	0.773100086	3.505125717	0.000456392	0.001354682
EFCAB14	48.57763074	0.836672848	0.238724814	3.504758611	0.000457021	0.00135625
LOC101115808	1.743743163	4.159037574	1.187019743	3.503764447	0.000458731	0.001361021
CREBZF	83.29433232	-0.645339115	0.184205175	-3.503371258	0.000459409	0.001362729

LOC101104176	28.91589224	-0.996001373	0.284373922	-3.502435685	0.000461025	0.00136722
R3HDM4	81.57161619	0.684392629	0.195411842	3.50230888	0.000461245	0.001367568
PPP2R5C	233.152028	0.49265438	0.140689002	3.501726317	0.000462254	0.001370257
LOC121820701	13.01709657	-1.346826111	0.384674041	-3.501213936	0.000463144	0.00137259
FAM43A	3.246669237	3.377266333	0.964799255	3.500486051	0.000464411	0.001376039
PTPRJ	3.792702574	3.003668907	0.858330841	3.499430247	0.000466254	0.001381194
LURAP1L	13.48487499	-1.339241622	0.382832737	-3.498242165	0.000468336	0.001387054
MAP3K13	71.34327753	0.841813166	0.240643622	3.498173607	0.000468456	0.001387103
ZNF71	3.354028958	3.295858597	0.942199606	3.498047097	0.000468678	0.001387454
GFM2	8.968612771	-1.705779773	0.487647107	-3.49797989	0.000468796	0.001387496
LDB2	1.702142352	4.109222386	1.174929647	3.497419948	0.000469782	0.001390104
LRRC4B	1.838141764	4.211817281	1.204940413	3.495456901	0.000473251	0.00140006
GTF2H4	2.85550454	-3.967032585	1.134954605	-3.495322693	0.000473489	0.001400454
PCOLCE	1.870460538	4.225276529	1.209084114	3.49460925	0.000474756	0.001403892
PPIL6	9.600759665	-1.789909444	0.51227654	-3.494029696	0.000475788	0.001406632
EML6	2.887168142	3.642241839	1.042461729	3.493885424	0.000476045	0.001407081
NPTXR	4.919429244	2.459479956	0.704127775	3.49294552	0.000477724	0.001411731
TUBA4A	230.3223669	-0.630752518	0.180582987	-3.492867901	0.000477863	0.001411829
PSMG1	26.86708866	-1.026371886	0.29386369	-3.49268018	0.000478199	0.00141251
TAF5	28.85704863	1.104466334	0.316248092	3.492404738	0.000478692	0.001413655
FRMD4A	24.83681767	-1.053758127	0.301767729	-3.491951018	0.000479506	0.001415745
KEF53_r01	939.1087133	1.471857481	0.421665249	3.490582839	0.000481968	0.0014227
MYDGF	10.11887877	-1.56603466	0.448714225	-3.490049062	0.000482932	0.00142523
CD44	29.62836617	-0.889304726	0.254879901	-3.489112798	0.000484627	0.001429111
KIAA1143	29.87043787	-0.922476778	0.264387549	-3.489108248	0.000484635	0.001429111
SEC16B	2.931598579	3.659658035	1.048863557	3.48916502	0.000484532	0.001429111
SLC35F5	19.10281214	1.277566252	0.366160685	3.489086364	0.000484675	0.001429111
GEMIN8	7.898927473	-1.743325658	0.499659936	-3.489024302	0.000484787	0.001429127
LOC132659210	2.323381839	3.946453259	1.131616905	3.487446361	0.000487657	0.00143727
RB1	10.25116863	1.552353098	0.445164069	3.487148234	0.000488201	0.001438556
ATG12	59.83238873	0.656403336	0.188349503	3.485028234	0.000492085	0.001449363
MAGI2	1.660756066	4.088041355	1.173022326	3.485049914	0.000492045	0.001449363
LONP2	52.11952067	0.724737612	0.20796829	3.484846719	0.000492419	0.001450027
TEX9	32.55049038	0.993113514	0.285169084	3.482542704	0.000496676	0.00146224
THAP3	14.35699186	-1.407358928	0.404129994	-3.482441169	0.000496864	0.001462473
RABL3	14.98387312	-1.328348123	0.381461451	-3.482260447	0.0004972	0.001463138
RHBDL1	4.374098212	-2.613996664	0.75107777	-3.48032756	0.000500801	0.001473411
NOL6	11.55271492	1.367643857	0.39309232	3.479192509	0.000502927	0.001479341
SHFL	16.69624961	-1.25261346	0.36007951	-3.478713517	0.000503827	0.001481661
BRD10	64.45231946	0.744224833	0.213960202	3.478333014	0.000504543	0.00148344
TLK1	112.2826024	0.668087733	0.192175842	3.476439727	0.000508118	0.001493625
BABAM2	4.767564593	2.562648323	0.737332928	3.475564738	0.000509779	0.001498176

KAT5	73.80584929	-0.66987217	0.192844459	-3.473639707	0.00051345	0.001508633
SIK1	4.965709757	2.336897851	0.672848227	3.473142616	0.000514402	0.001511098
SOAT2	3.813591313	2.87443658	0.827834517	3.47223572	0.000516143	0.001515879
ATF2	27.36135796	0.996122243	0.286897104	3.472054021	0.000516492	0.001516572
SLC24A2	1.767254421	4.172578335	1.201948273	3.471512402	0.000517535	0.001519301
LOC105603087	165.0122787	-0.703124568	0.20259145	-3.470652719	0.000519195	0.001523838
DGAT1	10.44883529	1.502220725	0.432857057	3.470477608	0.000519534	0.001524498
SLC46A3	13.86194425	1.20971969	0.348590484	3.470317598	0.000519843	0.001525071
NFYC	18.2755402	1.198857504	0.345470286	3.47021887	0.000520034	0.001525297
C12H1orf74	12.23406908	1.333536273	0.384309025	3.46995825	0.000520539	0.001526443
KIAA1549	6.061344457	-2.009861703	0.579243825	-3.469802552	0.000520841	0.001526993
NDUFS3	61.85522666	-1.002518965	0.288959831	-3.469405981	0.000521611	0.001528914
NTS	2.360087726	3.972202799	1.145428346	3.467875413	0.00052459	0.001537311
EML1	2.319659617	3.931281623	1.134082134	3.466487571	0.000527306	0.001544592
PTPRCAP	1.791606068	4.157672208	1.19938323	3.46650854	0.000527265	0.001544592
NCKAP5	3.622556664	3.023523588	0.872526752	3.465250299	0.000529738	0.001551376
NSRP1	188.9014726	0.627736668	0.181159057	3.465113362	0.000530008	0.001551826
ACAT1	70.31306893	-0.757007544	0.218484625	-3.464809231	0.000530608	0.001553242
CACNG4	17.22655884	-1.316901372	0.380108945	-3.464536656	0.000531146	0.001554476
BOD1	42.34546131	-0.800561593	0.231089861	-3.464286959	0.000531639	0.001555239
SGPP1	2.906093033	3.648095307	1.053056248	3.464292922	0.000531627	0.001555239
SAMD10	4.908764741	-2.337727027	0.674949654	-3.463557633	0.000533082	0.00155912
ZBTB44	44.70876369	-0.811767189	0.234405406	-3.463090734	0.000534008	0.001561486
ENY2	55.12091369	-0.710534525	0.205186271	-3.462875563	0.000534435	0.001562394
LOC105603761	1.669689145	4.061565448	1.173057912	3.4623742	0.000535432	0.001564965
ZCCHC9	22.07275448	-0.964865416	0.278707342	-3.46193039	0.000536316	0.001567205
TARDBP	58.04647464	-0.864800381	0.249811982	-3.461805049	0.000536566	0.001567593
PTPRN2	13.8413602	-1.22600473	0.354325091	-3.460112646	0.000539949	0.001577134
AHRR	9.988080332	1.488069986	0.430085531	3.459939663	0.000540296	0.001577803
LOC101113264	27.40911773	-0.919689998	0.265961664	-3.457979566	0.000544243	0.001588979
SOCS7	60.29493466	-0.696835605	0.201588947	-3.456715336	0.000546802	0.001596103
SORCS2	4.430044179	-2.386192457	0.690347572	-3.456508799	0.000547221	0.001596978
BCL11B	76.890156	0.818842206	0.236948738	3.455777879	0.000548707	0.001600965
RP2	7.733783328	1.663257729	0.48136921	3.455264057	0.000549754	0.001603669
C9H8orf89	28.42391169	-0.909510201	0.263266867	-3.45470819	0.000550889	0.001606278
RASEF	44.20447214	-0.81317798	0.235379399	-3.454754246	0.000550795	0.001606278
MXD1	22.00205005	1.072601548	0.310580985	3.453532577	0.000553295	0.001612944
TNRC6A	181.596226	0.694650198	0.201181474	3.452853699	0.00055469	0.001616303
UBE2E3	49.33434419	-0.923407586	0.267429508	-3.452900889	0.000554593	0.001616303
PTPN13	7.95386633	1.680476854	0.48672912	3.452591565	0.000555229	0.001617522
LOC101123244	4.751243578	2.287375485	0.662730641	3.451440666	0.000557602	0.001624082
ZNF503	15.72297169	1.414430743	0.409832875	3.451237882	0.000558021	0.001624949

LENG8	15.78831867	1.32255455	0.383277138	3.450648156	0.000559242	0.001628149
CCDC85B	5.133953129	2.290907715	0.663931827	3.450516486	0.000559515	0.001628561
LOC105604543	5.936070498	2.464121931	0.714142553	3.450462265	0.000559627	0.001628561
LOC101114959	20.62285059	1.013066489	0.293646649	3.449950787	0.000560689	0.001631294
SUCNR1	1.839333793	4.21332818	1.221388249	3.449622333	0.000561371	0.001632925
PTGES	2.046617795	-3.981701696	1.154340591	-3.449330055	0.000561979	0.001634232
SNRPB	3.583463261	-3.319089984	0.962253458	-3.449288704	0.000562065	0.001634232
EBF3	1.759930471	4.1418517	1.201104407	3.448369414	0.000563982	0.001639449
PDLIM7	6.674509618	1.881858097	0.545773913	3.448054315	0.00056464	0.001641006
NAE1	12.11223131	-1.545806842	0.448346424	-3.447795634	0.000565182	0.001642221
NDUFA3	25.64985678	1.20634147	0.349939212	3.447288639	0.000566243	0.001644948
LOC121818464	4.306593808	-2.517851528	0.7304488	-3.446992491	0.000566864	0.001646395
SLITRK3	1.659458116	4.055555638	1.176644955	3.446711449	0.000567454	0.00164775
FAM107A	3.10330226	3.76998212	1.094028493	3.445963378	0.000569027	0.001651601
RNF135	11.19464288	-1.368484073	0.397121625	-3.44600744	0.000568935	0.001651601
CREBBP	78.98481945	0.701835646	0.203729044	3.444946438	0.000571173	0.001657467
MGAT2	19.18789042	1.203766277	0.349633287	3.44293957	0.000575428	0.001669453
NDC1	36.90472302	-0.855048358	0.24840193	-3.442196913	0.00057701	0.00167368
TUBB4A	11.54041068	1.598160364	0.464580039	3.440010825	0.000581691	0.001686891
DUSP18	7.816249604	-1.676981178	0.487608791	-3.439193899	0.000583449	0.001691623
ZNF609	43.3117769	0.888709813	0.258411862	3.439121594	0.000583605	0.001691708
HSPA2	5.992002206	1.988923647	0.578448655	3.438375439	0.000585216	0.001696009
CD37	1.913111255	4.250690078	1.236496021	3.437690057	0.000586699	0.001699939
QKI	70.84067404	0.734100915	0.21360088	3.436787882	0.000588656	0.001704885
TMEM25	18.74047023	-1.104449299	0.321361112	-3.436785773	0.000588661	0.001704885
TPD52	132.3670131	0.719085807	0.209240958	3.436639815	0.000588978	0.001705434
ACRBP	2.200640588	3.836867864	1.116694927	3.435914117	0.000590558	0.001709638
LDLRAD4	2.145448684	3.81426685	1.110229247	3.435566899	0.000591315	0.00171146
PDE10A	1.622168256	4.036231894	1.174985594	3.435133089	0.000592263	0.001713831
TTL	21.38530111	-1.090846876	0.317592122	-3.43474161	0.000593119	0.001715566
VMAC	7.508437621	1.716904508	0.499861467	3.434760672	0.000593077	0.001715566
TRPC1	8.868532732	1.672005615	0.486844641	3.434372023	0.000593928	0.001717535
NEK9	54.83096348	0.71094144	0.207077793	3.433209474	0.000596481	0.001724171
PTPDC1	4.11393093	2.742638592	0.798845621	3.433252334	0.000596387	0.001724171
ATP2C1	87.77337031	0.680050499	0.19811992	3.432519557	0.000598001	0.00172819
LOC106990930	1.611937227	4.029937137	1.174304739	3.431764349	0.000599668	0.001732635
IDS	29.54150668	-1.214491004	0.353928612	-3.431457543	0.000600347	0.001734221
LOC132657430	1.706794855	-4.321743438	1.260363648	-3.428965477	0.000605887	0.001749844
ARL4A	73.176475	0.592307195	0.172768566	3.428327328	0.000607313	0.001753584
LOC101117505	9.753452381	1.775977241	0.518060345	3.42812813	0.000607759	0.001754469
LOC114110475	3.503437632	-3.564503826	1.03979807	-3.428073132	0.000607882	0.001754469
HIF1A	72.19481729	0.625999661	0.182648307	3.42734993	0.000609503	0.001758769

CATSPER4	6.331627048	-1.954174236	0.570588017	-3.424842756	0.000615155	0.001774696
LIG1	55.35711461	-0.893869412	0.261010142	-3.424653936	0.000615583	0.001775546
RHOA	88.64660532	-0.660418445	0.192868582	-3.424188839	0.000616638	0.001778204
GNAZ	25.63378433	0.954577126	0.279174922	3.419279635	0.000627872	0.00181021
LOC101115172	7.009772431	1.869889742	0.547006423	3.418405457	0.000629892	0.001815643
SFPQ	639.2706911	0.587033253	0.17174128	3.418125515	0.00063054	0.00181712
GPR171	1.633663715	4.043737869	1.183269634	3.417427231	0.00063216	0.001821395
GJA5	1.797947507	4.191087621	1.22653754	3.417007212	0.000633136	0.001823814
TAOK2	39.66290962	0.794622887	0.232560038	3.416850517	0.000633501	0.001824471
SLC25A24	114.5941237	0.612686144	0.179319716	3.416724925	0.000633793	0.001824919
SMIM7	127.1878328	-0.545933006	0.159807247	-3.416196795	0.000635023	0.001828069
CGGBP1	59.80239612	0.655922905	0.192023009	3.41585578	0.000635819	0.001829965
SETD1B	35.465236	-0.891776519	0.26113321	-3.415025306	0.000637761	0.001835159
TSTD3	27.96999287	-0.94094209	0.275589365	-3.414290284	0.000639484	0.001839721
HMGB1	1484.207141	-0.507811579	0.148753678	-3.413774943	0.000640695	0.001842411
LOC101102223	42.80149233	0.962071791	0.281817969	3.413805711	0.000640622	0.001842411
PIK3C3	20.68205163	1.072111639	0.314068653	3.41362192	0.000641055	0.001843049
GRK4	78.97225382	0.693944295	0.203350112	3.41255919	0.000643559	0.001849455
TXNRD1	76.11871387	0.702144699	0.205752924	3.412562431	0.000643552	0.001849455
NMI	19.18070893	1.099435251	0.322381741	3.410352108	0.000648791	0.001864088
RAD51B	15.02036886	1.157803839	0.339602516	3.409291106	0.000651319	0.001870951
ZBTB7B	43.56163678	0.804187983	0.235912565	3.408839126	0.000652399	0.001873651
DUOXA2	2.455207341	4.053358257	1.189191549	3.408499045	0.000653213	0.001875585
CITED2	5.520675076	2.048406007	0.601038305	3.408112245	0.00065414	0.001877842
LOC121816116	1.694392432	4.079060229	1.196968069	3.407827105	0.000654824	0.001879402
PHAX	78.87771388	-0.623968884	0.183113112	-3.407559823	0.000655465	0.001880839
LOC101120236	16.26341877	-1.896661329	0.556644533	-3.407311517	0.000656062	0.001882147
LMAN1	133.2886314	0.54513041	0.160010285	3.406846071	0.000657182	0.001884955
TPI1	323.5323222	0.738058558	0.216671973	3.40634069	0.0006584	0.001888043
RPA2	31.35986707	-0.865976189	0.254282956	-3.405561278	0.000660282	0.001892889
SEPTIN1	1.798790577	4.137694468	1.214995063	3.405523689	0.000660373	0.001892889
UCHL5	105.9359332	-0.536352438	0.1575823	-3.403633767	0.000664958	0.001905625
OSR1	3.84770401	2.766420886	0.81290673	3.403122132	0.000666205	0.001908788
LOC121820608	4.05367387	2.983584894	0.876744217	3.403027745	0.000666435	0.001909038
CD2BP2	34.51504485	-0.804263879	0.236361547	-3.402684956	0.000667272	0.001911025
FAM177B	5.541762439	2.294586563	0.674731328	3.400741108	0.000672034	0.001924253
CHGA	325.5460329	0.6476876	0.190487172	3.40016387	0.000673455	0.001927907
FASTKD1	16.19565468	-1.163569575	0.342225511	-3.400008292	0.000673838	0.001928591
CDCA7	20.93325942	-1.205506643	0.354612154	-3.399507398	0.000675074	0.001931713
CERK	19.59925529	-1.13343628	0.333458827	-3.399029174	0.000676255	0.001934266
PTCH2	2.306940977	3.924858152	1.154685404	3.399071417	0.000676151	0.001934266
EFTUD2	9.990212253	-1.427823512	0.420088688	-3.398862077	0.000676668	0.001935034

QTRT1	13.5136751	-1.386425738	0.407927154	-3.398709115	0.000677047	0.001935702
AFG2A	18.76695802	1.088603715	0.320329001	3.39839263	0.000677831	0.001936106
LOC101107129	4.692256735	2.308581914	0.67928911	3.398526314	0.000677499	0.001936106
LOC114115623	13.29598777	-1.585182874	0.466455301	-3.398359649	0.000677912	0.001936106
PPP1R16A	15.3161921	1.154030833	0.339582189	3.398384461	0.000677851	0.001936106
RPS27L	282.4281562	-0.460562525	0.135524654	-3.398367096	0.000677894	0.001936106
LOC132660202	2.85354973	3.676399617	1.081838817	3.398287767	0.000678091	0.001936201
PLPPR3	5.089601897	-2.331276371	0.686092899	-3.39790191	0.000679048	0.00193852
ANKLE2	13.32773098	-1.328419678	0.391129618	-3.396366871	0.000682868	0.001949009
LOC101111247	142.7872149	0.621879274	0.183138231	3.395682442	0.000684577	0.001953472
ATP6V1E1	36.0884785	-0.872110118	0.256882952	-3.394970783	0.000686359	0.001958138
ZMAT1	1.622132055	4.036541472	1.189175821	3.394402579	0.000687785	0.001961787
MSI1	2.988666352	-3.376789908	0.995132485	-3.39330688	0.000690542	0.001969232
SERPINE2	77.08760851	0.737095272	0.217254789	3.392768813	0.0006919	0.001972683
TSPAN5	15.30404897	-1.181174037	0.348185498	-3.392369999	0.000692908	0.001975135
MID1	12.79137442	-1.677999483	0.494671263	-3.392150721	0.000693463	0.001976295
CLEC14A	1.648205694	4.082735269	1.203901741	3.391252898	0.000695739	0.001982358
GALNT6	16.70069973	1.557561355	0.459322658	3.390996125	0.000696391	0.001983794
LOC105612575	8.844649523	1.94245179	0.57294909	3.390269441	0.00069824	0.001988636
MRPL39	18.9931196	-1.131636568	0.33396365	-3.388502216	0.000702755	0.002001069
ATOSB	4.431674949	2.40576966	0.710281311	3.387065973	0.000706444	0.002011146
HEXIM1	21.94802191	1.077418132	0.31816943	3.38630312	0.000708411	0.002016316
MRAS	2.729804578	3.592270994	1.061181757	3.385160901	0.000711365	0.002024294
ST6GALNAC2	2.179272243	3.874985887	1.144770688	3.384945062	0.000711925	0.002025455
LSM7	27.93460511	-0.877826543	0.259419677	-3.383808635	0.000714878	0.002033424
PSRC1	7.016348373	-1.811922385	0.535569202	-3.383171358	0.000716539	0.002037715
RAPGEF6	36.11010293	0.850879172	0.251642809	3.381297379	0.000721444	0.002051228
OSMR	5.881319573	2.093446906	0.619141716	3.381207974	0.000721679	0.002051459
CRCP	34.03344277	-0.895979402	0.265188153	-3.378655467	0.000728412	0.002070159
ZNF507	41.10981908	0.781027112	0.231173974	3.378525262	0.000728757	0.0020707
LOC105611446	5.33048577	2.490312708	0.737308225	3.377573481	0.000731284	0.002077438
SFMBT1	39.72398594	1.143356322	0.338630853	3.376409182	0.000734386	0.002085806
PAMR1	2.252859829	3.860603291	1.143492228	3.376151754	0.000735074	0.002087316
H6PD	13.34580192	-1.205066152	0.35698236	-3.375702237	0.000736276	0.002090284
HR	4.028042166	2.763038433	0.818834367	3.374355722	0.000739887	0.002100091
HERC1	86.81873637	0.63189341	0.187310559	3.373506612	0.000742173	0.002106094
LOC114117367	4.391430941	2.659612754	0.788394781	3.373453018	0.000742317	0.002106094
STARD3NL	6.429688436	1.866602903	0.55337659	3.373115049	0.000743229	0.002108234
HYAL2	6.447987522	-1.95664448	0.580104136	-3.372919376	0.000743757	0.002109285
RSAD2	13.31836943	-1.223320358	0.362704332	-3.372775703	0.000744145	0.002109938
TCF7L1	1.61029458	4.059072303	1.203925408	3.37153139	0.000747515	0.002119043
IFT140	6.903626505	1.800065757	0.534376281	3.368536033	0.000755685	0.002141749

CCKBR	1.551868076	-4.333321846	1.28666201	-3.367878909	0.000757489	0.002146405
GPAT3	13.96442491	1.419760911	0.421625594	3.367349917	0.000758943	0.002150071
BANP	15.17689337	-1.264651154	0.375607815	-3.366945798	0.000760056	0.002152768
DESI2	37.56251327	-0.767684061	0.228051559	-3.366274124	0.00076191	0.00215756
EVI2A	1.56872997	4.006657515	1.19035211	3.365943137	0.000762824	0.002159658
LOC114108665	4.147320561	-2.800995496	0.832171041	-3.36588917	0.000762974	0.002159658
TUSC2	10.39753733	-1.64313514	0.488237143	-3.36544477	0.000764204	0.002162682
TMEM100	1.713500696	4.113552759	1.222348467	3.365286471	0.000764642	0.002163465
TMEM167B	83.27879897	-0.593296534	0.176312521	-3.365027802	0.000765359	0.002165036
EFCAB2	10.99257444	1.404777286	0.417484268	3.364862805	0.000765817	0.002165873
RPL7L1	53.73529439	-0.705280488	0.209623213	-3.364515199	0.000766783	0.002168144
EIF2B5	18.78166299	-1.170032162	0.347800583	-3.36408913	0.000767967	0.002171035
ALG3	7.740556385	1.637423592	0.486790964	3.363709917	0.000769023	0.00217356
SNRNP70	144.0457543	-1.004371054	0.298748459	-3.361928813	0.000774001	0.002187166
SWT1	10.03415179	1.473257169	0.438228045	3.361850494	0.00077422	0.002187324
TAL1	1.642179694	4.078627161	1.213343971	3.361476431	0.00077527	0.002189826
SLC9A8	38.92862959	-0.875109036	0.260446719	-3.360030947	0.000779337	0.00220085
LOC114117360	4.42892714	-2.336097227	0.695533687	-3.358711837	0.000783067	0.002210915
IGF2BP3	9.424529266	-1.519871834	0.452782677	-3.356735825	0.000788684	0.002226305
RNF215	20.90012963	-1.022758337	0.304715979	-3.35643159	0.000789553	0.002228197
SLC27A1	3.625612233	-2.91274209	0.86782143	-3.356384147	0.000789688	0.002228197
ENKD1	6.224958191	-1.870105474	0.557364976	-3.355261909	0.000792899	0.002236784
DNAH11	3.834765914	2.754065036	0.821042171	3.354352717	0.000795509	0.002243674
TMEM147	51.45566919	-1.093190602	0.326078446	-3.352538681	0.000800741	0.002257953
BTBD19	7.768401166	1.931739289	0.576268172	3.35215336	0.000801856	0.002260621
RIPOR1	13.35325922	-1.384892357	0.413162785	-3.351929092	0.000802506	0.002261976
LOC121818085	2.985674207	-3.449024047	1.029005103	-3.351804607	0.000802867	0.002262516
NPRL3	4.967838871	-2.251675054	0.67187763	-3.351317192	0.000804281	0.002266025
MAPRE1	25.53971402	-0.903461148	0.269628406	-3.350763973	0.00080589	0.002270078
HROB	4.10382135	-2.460623727	0.734494336	-3.350092176	0.000807847	0.002275112
LSM5	37.96697004	0.947259397	0.282761184	3.350033349	0.000808018	0.002275115
HPDL	10.27340567	-1.39627933	0.416847551	-3.349616248	0.000809236	0.002278064
SLC6A19	27.4640812	2.146318294	0.640838705	3.349233244	0.000810355	0.002280735
LOC101102428	24.79194927	1.071778463	0.320071555	3.348558932	0.00081233	0.002285811
TPM3	133.8330888	-0.616604039	0.184145407	-3.348462766	0.000812612	0.002286123
LOC132657463	60.1476014	-0.925008806	0.27627769	-3.348112568	0.00081364	0.002288532
LDB3	3.070599687	3.179770947	0.949770393	3.347936481	0.000814157	0.002289505
ZMIZ2	17.88306439	-1.01766936	0.304018405	-3.347393917	0.000815752	0.002293509
MADCAM1	1.694534554	4.107999677	1.227316973	3.347138324	0.000816505	0.002295142
SYNJ1	12.19737762	1.335793725	0.399163846	3.346479748	0.000818447	0.002300117
FMO5	6.702382045	1.958315727	0.585226413	3.346253151	0.000819116	0.002301514
KLHL23	4.072692905	2.678162104	0.800363683	3.346181442	0.000819328	0.002301625

PGM3	21.51232171	1.044600791	0.312212131	3.345804627	0.000820442	0.002304271
AAMDC	52.14890734	0.734375641	0.219619844	3.343849216	0.000826246	0.002320086
PPP1R3E	7.734009267	-1.781073639	0.53267804	-3.343621295	0.000826925	0.002321505
FOXJ3	57.35251202	0.674116854	0.201633546	3.343277288	0.000827951	0.002323897
TMEM106A	17.034548	-1.182264694	0.353692381	-3.342635464	0.000829868	0.002328789
LOC132660218	4.488334053	-2.258416381	0.675683436	-3.342417858	0.000830519	0.002330126
CYP2J	10.09322298	1.458036142	0.436274676	3.34201415	0.000831728	0.002332539
LOC121819811	23.84259867	0.960584848	0.287424919	3.342037469	0.000831658	0.002332539
GATA6	50.86229833	-0.673804968	0.201671033	-3.341109316	0.000834444	0.002339663
MEGF9	28.44468617	-0.905737815	0.271124225	-3.340674609	0.000835751	0.002342837
STXBP1	6.14776613	2.54959286	0.763310505	3.340177872	0.000837247	0.00234654
RIMS1	1.598231027	4.025014494	1.205335113	3.339332315	0.0008398	0.002353202
TRIM56	53.9276447	0.729660064	0.218548152	3.338669562	0.000841806	0.002358328
PDGFB	6.051417939	1.999626809	0.598944337	3.338585384	0.000842061	0.002358549
LOC106991020	2.342197742	3.898151896	1.167660511	3.338429157	0.000842535	0.002359381
TMEM119	1.583689048	3.98311591	1.193242085	3.338061874	0.00084365	0.002362007
CEP72	3.978170548	-2.506048549	0.750780826	-3.337922948	0.000844072	0.002362693
LOC114112619	2.092776456	3.795002239	1.136985948	3.33777409	0.000844524	0.002363464
DUSP6	243.1541619	0.554790578	0.16624465	3.337193564	0.00084629	0.002367911
SNX29	7.879263808	1.960347804	0.587634788	3.335996852	0.000849941	0.00237763
IFI44	5.099226487	2.116011022	0.634335206	3.335793128	0.000850564	0.00237788
KMT2E	514.3555229	0.500462544	0.150027436	3.33580682	0.000850522	0.00237788
LOC101117600	1.577663049	3.979257536	1.192860377	3.335895477	0.000850251	0.00237788
TTI2	4.751215356	-2.216381189	0.664538599	-3.335218136	0.000852325	0.002382304
AFAP1L1	4.030226403	2.734503103	0.82014052	3.334188514	0.000855487	0.002390141
NDUFA12	21.24447838	-1.17596081	0.352696499	-3.334200404	0.00085545	0.002390141
LOC101121923	20.37391162	1.11724859	0.335119416	3.333882007	0.00085643	0.002392276
HELZ	47.72285427	0.696321638	0.208924877	3.332880455	0.000859519	0.002400402
LOC101112832	1.601174307	3.995139751	1.198967027	3.332151477	0.000861773	0.002406197
DDX46	405.3764156	0.760377189	0.228236561	3.331531039	0.000863697	0.002411063
LOC114111217	3.30262069	-3.086188296	0.926482104	-3.331082471	0.00086509	0.002414447
USF2	52.19633459	-0.749923993	0.225142391	-3.33088758	0.000865696	0.002415634
TBC1D14	35.63499849	0.9038782	0.271408747	3.33032082	0.00086746	0.002420052
TOMM22	243.8191793	-0.433777814	0.130256844	-3.33017293	0.000867921	0.002420832
MAVS	32.51214775	0.80554212	0.241898974	3.330076634	0.000868221	0.002421164
NCBP2	34.43595643	-0.763552103	0.22932516	-3.329560973	0.00086983	0.002425146
TCF25	90.77562988	-0.703373467	0.211261494	-3.329397395	0.000870341	0.002426065
HNF1B	12.82987886	-1.336230989	0.401514818	-3.327974284	0.000874799	0.002437984
NLRP1	1.704765583	4.114563993	1.236476162	3.327653311	0.000875808	0.002440285
DCTN4	40.54468196	-0.832185697	0.250094713	-3.327482163	0.000876346	0.002440491
MBOAT2	11.106197	1.387580468	0.417009463	3.327455584	0.00087643	0.002440491
PPIG	690.5464705	0.649718802	0.195254185	3.327553786	0.000876121	0.002440491

PTPRF	219.5982422	-0.812471563	0.24422215	-3.32677263	0.00087858	0.00244597
KMT2D	30.52343833	0.858596068	0.258121735	3.326322236	0.000880001	0.002449416
PAIP2	60.30786489	0.997035086	0.29980411	3.325621808	0.000882215	0.002455068
ESRP1	62.9678777	-0.664633112	0.199913011	-3.324611584	0.000885418	0.002463467
ZDHHC2	2.07158186	3.805317786	1.144694668	3.324308126	0.000886382	0.002465636
LARGE1	85.57159108	-0.683723513	0.205688519	-3.324072317	0.000887132	0.002467209
SCAF4	34.87096081	-0.847688128	0.255125598	-3.322630638	0.000891729	0.002479478
USP8	188.4319522	0.629570899	0.189503201	3.322217762	0.00089305	0.002482634
PEX7	28.28154672	-1.014750478	0.305450125	-3.322147856	0.000893273	0.002482739
PSMB1	33.09732906	-1.021065359	0.307455537	-3.321017956	0.000896898	0.002492294
PABPN1	366.6273935	0.77679332	0.233937639	3.320514493	0.000898517	0.002496275
CENPX	6.038224297	-1.983261914	0.597294793	-3.32040717	0.000898862	0.002496716
DNAL1	18.76990003	1.167677665	0.351699128	3.320103951	0.000899839	0.00249891
ZFHX3	122.0340655	0.520079601	0.156661645	3.319763445	0.000900938	0.00250144
ETFB	107.4192342	0.65716113	0.19795982	3.319669271	0.000901241	0.002501764
CDK4	15.66142693	-1.19401966	0.359751383	-3.319013402	0.000903361	0.002507127
LOC132657607	9.108765572	-1.415449456	0.426511439	-3.318667045	0.000904482	0.002509717
LIMS1	53.27495892	-0.634147711	0.191116955	-3.318113312	0.000906277	0.002513654
LOC114117625	1.786075759	4.17817736	1.259197152	3.318128026	0.000906229	0.002513654
RAP2A	52.98891949	0.773566705	0.233162361	3.317716893	0.000907564	0.002516702
SPTSSA	29.77671207	0.913415153	0.275462987	3.315926987	0.000913397	0.002532351
MTREX	128.1414462	-0.647058508	0.195155744	-3.315600632	0.000914464	0.002534784
MAP4	85.61392057	-0.848491253	0.256038675	-3.313918316	0.000919984	0.002549555
POLR1G	110.6456388	-0.797480288	0.240662838	-3.313682724	0.000920759	0.002551175
RAB11FIP4	21.78477736	-1.303441289	0.393386104	-3.313389254	0.000921726	0.002553324
CLIP4	1.547736021	3.963740822	1.196300802	3.313331241	0.000921917	0.002553325
CCDC88B	1.943172716	4.291997468	1.295415141	3.313221633	0.000922279	0.002553797
PABIR1	14.37826823	1.307519464	0.394739653	3.312359057	0.000925127	0.002561154
THBS3	2.69903004	3.531728556	1.066700927	3.31088918	0.00093	0.002574111
FSD1L	21.75273189	1.23599014	0.373409858	3.310009397	0.000932928	0.002581681
ZNF333	16.13621349	1.103958566	0.333566182	3.309563815	0.000934415	0.002585259
C20H6orf136	11.45101148	-1.413298775	0.42707519	-3.309250472	0.000935461	0.002587619
CTR9	145.2534134	0.677389466	0.204700196	3.309178397	0.000935702	0.002587749
POLA1	55.05388263	-0.730506993	0.220775241	-3.308826611	0.000936878	0.002590467
LOC121820309	26.36348921	-0.901010172	0.272412519	-3.307521162	0.000941256	0.002602033
PTPN23	24.84167263	-1.264770787	0.382404663	-3.307414654	0.000941614	0.002602484
BTBD1	32.18641231	-0.912604818	0.275986559	-3.306700228	0.000944019	0.00260859
GAN	220.8257384	-0.469869472	0.142134061	-3.30581894	0.000946993	0.002616268
RAB28	31.54795182	-1.047317948	0.316891044	-3.304978057	0.000949838	0.002623587
LOC105603753	4.775860085	-2.291231224	0.693588432	-3.303444978	0.000955047	0.00263743
XPO6	6.032981627	1.824444816	0.552305297	3.303326664	0.00095545	0.002637998
KATNB1	4.071506188	-2.60803418	0.789571203	-3.303101949	0.000956216	0.002639567

OSBPL6	46.17691715	-0.773290836	0.234123442	-3.302919312	0.000956839	0.002640742
BRD2	146.8287462	-0.729954796	0.22111519	-3.301242194	0.000962578	0.00265603
FANCF	2.506586087	-3.700347122	1.1209163	-3.30118058	0.000962789	0.002656065
CCAR2	21.22153877	-1.279250252	0.387558181	-3.300795373	0.000964112	0.002659166
LOC121817713	4.835471342	2.153112575	0.652361905	3.3004879	0.000965169	0.002661532
KIF21A	70.00445194	0.73350684	0.222270765	3.300059904	0.000966642	0.002665045
NDUFB7	53.15857596	-0.700764309	0.212480265	-3.298020687	0.00097369	0.002683921
PROCR	7.267779095	1.64943668	0.500296686	3.296917065	0.000977524	0.002693934
SLMAP	168.1670002	0.535156846	0.162341126	3.296495837	0.000978991	0.002697421
ATG14	24.35751829	-0.918014043	0.278506802	-3.296199723	0.000980023	0.002699709
RDH13	8.875036969	-1.563901947	0.474509871	-3.295825949	0.000981328	0.002702747
PSMB4	93.59971801	-0.723543287	0.219542598	-3.295685176	0.00098182	0.002703544
ZNF41	2.023567962	3.737365483	1.134168491	3.295247145	0.000983352	0.002707204
RFX7	16.34702166	-1.21797921	0.369691729	-3.294580635	0.000985687	0.002713074
ALKBH6	7.338274999	-1.684792262	0.511759286	-3.292157674	0.000994219	0.002735994
STXBP5	56.40142937	0.644966381	0.19595008	3.291483129	0.000996606	0.002742
LOC101110922	2.906501984	3.647249683	1.10812901	3.291358362	0.000997048	0.002742652
MAP3K20	51.29818365	0.726214831	0.220656598	3.291153936	0.000997773	0.002744081
SARS2	14.39173867	-1.284666374	0.390400475	-3.290637322	0.000999607	0.002748559
FRYL	52.30151374	0.711427888	0.216203992	3.290540025	0.000999953	0.002748945
MS4A2	1.485742874	3.926783197	1.193444491	3.290293956	0.001000828	0.002750784
BCL2L15	70.8802637	0.887262516	0.269693659	3.289890164	0.001002265	0.002753602
BRD3OS	9.922677939	-1.433736747	0.435793201	-3.289947489	0.001002061	0.002753602
HMG20A	22.99080697	0.975344142	0.296515417	3.289353898	0.001004177	0.002758287
ASB5	2.945413941	3.51036548	1.067283946	3.289064257	0.001005211	0.00276056
AGPAT3	39.58357865	0.737227418	0.224186063	3.288462311	0.001007363	0.002765902
EPG5	16.45512974	1.210390296	0.368110089	3.288120405	0.001008587	0.002768694
TBC1D30	40.85085496	-0.74580178	0.226920804	-3.286617039	0.001013986	0.002782945
LBH	6.427339309	1.793175322	0.545640946	3.286365028	0.001014894	0.002784864
WDR11	12.45610072	-1.234236628	0.375603185	-3.286012147	0.001016166	0.002787783
RTRAF	70.82974957	-0.5654429	0.172106818	-3.285418361	0.00101831	0.002793092
SLC66A1	6.373494614	1.837250391	0.559316483	3.284813601	0.001020499	0.00279852
ALOX5	4.756485727	2.388841748	0.727299805	3.284535114	0.001021508	0.002800712
NDUFA7	47.27327495	-0.727251852	0.221442614	-3.284154926	0.001022887	0.002803918
CCDC25	66.5857633	0.686895396	0.209230764	3.282956011	0.001027247	0.002815293
ZFP37	1.642596794	4.022081085	1.225165132	3.28288896	0.001027491	0.002815385
CUEDC1	85.34429894	-0.722903477	0.220252703	-3.282154849	0.00103017	0.002822147
STPG1	2.475346363	-3.729615416	1.13662097	-3.281318499	0.00103323	0.002829949
PLEKHA1	33.80393314	-0.843148728	0.25708478	-3.279652442	0.00103935	0.002846129
SLC39A10	65.25292525	0.699405242	0.21328725	3.27917043	0.001041127	0.002850411
PCCB	9.514308648	-1.477773084	0.450688149	-3.278925988	0.00104203	0.002852297
EIF3K	52.26031928	-0.822972565	0.250994653	-3.278845005	0.001042329	0.002852532

NECTIN4	3.274299173	-3.068154029	0.935849364	-3.278469961	0.001043715	0.00285574
KCNJ2	6.30080421	1.848020708	0.563839744	3.277563753	0.001047071	0.002864337
CUBN	4.925465982	3.161371037	0.964669446	3.27715473	0.001048589	0.002867902
TIPARP	40.09261537	0.777663421	0.237306948	3.277036039	0.00104903	0.002868521
POLR2J	16.54897822	1.087492991	0.331892651	3.276640773	0.001050499	0.002871952
CHRAC1	47.23230191	-0.790677651	0.241320705	-3.276460056	0.001051172	0.002872921
CORO7	62.40051269	-0.715258998	0.218304373	-3.276430002	0.001051284	0.002872921
SIGMAR1	12.06892745	-1.460418623	0.445758236	-3.276257183	0.001051927	0.002874092
HERC3	16.89736333	1.189064467	0.362958075	3.276038057	0.001052744	0.002875735
CHAF1B	13.87417285	-1.295448542	0.395441392	-3.275955854	0.00105305	0.002875985
APOE	1.706274979	4.112860043	1.255669983	3.275430726	0.00105501	0.002880291
PRKRA	1.994767256	3.688501997	1.126136319	3.275360129	0.001055274	0.002880291
ZBTB47	5.529454833	2.128418292	0.6498172	3.275410827	0.001055084	0.002880291
CEP89	18.26917456	1.051775118	0.321201422	3.274503301	0.001058479	0.002887861
LOC132660076	1.586287631	4.017187723	1.2267934	3.274542986	0.00105833	0.002887861
UVSSA	16.45527244	-1.095182853	0.334483326	-3.274252465	0.001059419	0.002889836
LSAMP	3.612104607	2.989741963	0.913271426	3.273662	0.001061635	0.00289529
FZD3	21.56143005	-1.03920118	0.317493137	-3.273145336	0.001063578	0.002899996
CDH2	2.24433924	3.88538324	1.187138005	3.272899379	0.001064504	0.002901775
LRRC8E	15.59009158	-1.201866973	0.367222608	-3.272856705	0.001064664	0.002901775
KIAA0040	11.07458654	-1.686998686	0.515523157	-3.272401376	0.001066381	0.00290586
TRIM41	42.00004008	0.778682048	0.238042773	3.271185422	0.001070977	0.002917789
SPIRE2	43.74318334	-0.906868397	0.277243055	-3.27102295	0.001071592	0.002918871
LCMT1	18.12091271	-1.085520995	0.332014125	-3.269502451	0.001077368	0.002934006
SLC27A3	3.209458397	-2.959648176	0.905247183	-3.269436494	0.001077619	0.002934092
LOC101118606	13.82513938	-1.3022851	0.398359121	-3.269123339	0.001078813	0.002936743
RPUSD4	6.403891589	-1.791199569	0.548146488	-3.267738842	0.001084104	0.002950545
UBE2K	97.96991398	-0.632369124	0.19354716	-3.267261194	0.001085934	0.002954927
EBI3	1.530286964	3.952490155	1.209965783	3.266613164	0.001088423	0.002961095
TM6SF2	5.394408131	2.292359292	0.70178554	3.266466979	0.001088985	0.002962022
FRMD8	4.317368122	-2.522160951	0.77235873	-3.265530449	0.001092593	0.002971229
YPEL3	8.798704295	1.818207579	0.55680204	3.265447048	0.001092914	0.0029715
TTLL7	5.217798529	2.123078044	0.650331438	3.264609275	0.001096152	0.002979696
TRIP10	10.05923035	-1.360999169	0.416970527	-3.264017668	0.001098443	0.002985317
ARPC2	245.6392343	0.534623158	0.163815836	3.263562127	0.001100211	0.002989513
ESPN	10.71366226	1.465855011	0.44920827	3.263196851	0.00110163	0.002992761
DNAJB14	57.33214347	0.695863183	0.213269475	3.262835348	0.001103036	0.002995972
GMDS	14.7279904	-1.641262075	0.50310271	-3.26228033	0.001105198	0.003001234
CDC42EP3	38.71197565	0.804760953	0.246728689	3.261724264	0.001107368	0.003006517
P4HA3	2.406755129	-3.623923392	1.111261218	-3.261090492	0.001109846	0.003012633
IL17RC	47.16756427	0.689822597	0.211627137	3.259613135	0.001115643	0.003027752
FABP3	40.6208296	0.926661149	0.284310092	3.259332588	0.001116747	0.003030133

LOC101107495	22.09922715	-0.955093292	0.293104691	-3.25853977	0.001119872	0.003037996
CEACAM1	49.85609633	0.79086149	0.242716463	3.258375973	0.001120518	0.003039133
LOC114113196	4.40330185	2.774585498	0.851591404	3.258118255	0.001121537	0.003040044
POLG	54.35399532	0.857723477	0.263255446	3.25814144	0.001121445	0.003040044
PRKG2	30.50230136	1.063219685	0.326318896	3.258222852	0.001121123	0.003040044
KHDRBS3	3.605953804	2.805516208	0.861254317	3.257477093	0.001124073	0.003046302
ANKRD52	13.25264511	-1.550856593	0.476197522	-3.256750656	0.001126954	0.00305349
NECTIN3	276.8078678	-0.438704804	0.134713838	-3.256568213	0.001127678	0.003054833
BCAS2	18.66185864	-1.319907392	0.405396373	-3.255844105	0.001130558	0.003062014
CDHR5	25.21670559	1.03530839	0.318093971	3.254724972	0.001135022	0.003073482
TROAP	3.7334716	-2.55170929	0.784075451	-3.254418038	0.001136249	0.003076182
MTX3	8.52069348	1.590857177	0.4888669	3.254172408	0.001137232	0.00307822
LOC121816730	3.061889295	-3.314107865	1.018638429	-3.253468327	0.001140054	0.003085233
ENTPD5	44.33015779	0.830931043	0.255431264	3.253051441	0.001141728	0.003089138
SLC45A3	20.35096119	1.494164169	0.45934413	3.252820865	0.001142655	0.00309102
TEK	1.831243785	4.187956808	1.287509682	3.252757525	0.00114291	0.003091083
SNTB1	56.27416653	-0.915023751	0.281403436	-3.25164385	0.001147397	0.003102591
ITGA10	4.651908406	2.262209807	0.695740561	3.251513472	0.001147923	0.003103387
FETUB	1.551904849	3.967592805	1.2213916	3.248419923	0.001160479	0.003136695
LOC105602976	1.60044445	3.993688296	1.229599769	3.247957909	0.001162365	0.003141158
RGP1	50.14230388	-1.033888216	0.318339619	-3.247752252	0.001163205	0.003142793
SNX30	80.83790778	-0.638244225	0.196545251	-3.247314405	0.001164996	0.003146997
LYSMD3	30.80034133	0.8380127	0.258096819	3.24689279	0.001166723	0.003151025
TRAPPC3	20.95403492	-1.159512105	0.357164546	-3.246436742	0.001168594	0.00315544
JADE1	53.28244623	0.677359529	0.208676953	3.245971917	0.001170504	0.003159959
CCDC190	1.940617067	3.672830429	1.131545257	3.245853763	0.00117099	0.003160632
LOC121817871	1.866706957	-3.797994167	1.170132923	-3.245780108	0.001171293	0.003160811
CSNK1A1	421.6610093	-0.470862554	0.145093301	-3.245239793	0.001173518	0.003166026
TLR3	10.1385955	1.424296592	0.438893886	3.245195796	0.001173699	0.003166026
YWHAG	145.7564875	0.797442816	0.245769717	3.244674831	0.001175848	0.003171184
BRD9	185.2056077	0.69326529	0.213693413	3.244205244	0.001177788	0.003175135
PAX5	1.995319784	3.698459998	1.140014675	3.244221394	0.001177722	0.003175135
PLA2R1	3.360681772	2.924619841	0.901551635	3.243984844	0.0011787	0.003176952
MALT1	35.45243768	0.849489228	0.262105386	3.24102164	0.001191021	0.003209515
NLE1	5.670584877	-2.000129377	0.617184191	-3.240733328	0.001192227	0.003212115
LOC101122597	78.94744226	-0.82533506	0.25471987	-3.24016756	0.001194595	0.003217846
CAB39	57.4422345	0.646431493	0.199533824	3.239708837	0.001196518	0.003222378
LOC132658444	5.798428604	-1.816705768	0.560828449	-3.239325273	0.001198129	0.003226065
EPB41L2	37.51908905	0.947323532	0.292545792	3.238205973	0.001202839	0.003238097
FBXL6	4.901209052	-2.488144496	0.768422582	-3.237989817	0.001203751	0.003239246
LOC100144429	1.526081935	3.949713875	1.219803128	3.237992905	0.001203738	0.003239246
TDRKH	27.58287614	-0.838681525	0.259048384	-3.2375478	0.001205617	0.003243616

PPM1D	23.10029243	0.917133582	0.283361681	3.236618229	0.001209551	0.003253544
MTCL1	3.861763532	2.680655271	0.828277696	3.236420929	0.001210388	0.003255139
LOC105609560	1.464748925	3.880469442	1.199055308	3.236272269	0.001211018	0.00325618
RHEBL1	9.680802577	-1.457793097	0.450499891	-3.235945506	0.001212405	0.003259254
LOC114116979	1.882739517	-3.80795872	1.176902947	-3.235575821	0.001213976	0.003262821
GPSM3	3.722934804	2.55223076	0.788933123	3.235040699	0.001216254	0.003268285
FUT8	70.27998109	-0.598271911	0.1849565	-3.234662801	0.001217865	0.003271956
GJA4	1.504979383	3.904642979	1.20767115	3.233200511	0.001224116	0.00328809
AFG3L2	67.0752368	-0.686099345	0.212243455	-3.232605431	0.001226669	0.003294284
MAMDC2	1.946500629	3.702581053	1.145425194	3.23249486	0.001227143	0.003294897
EEFSEC	12.50789034	-1.206442902	0.373246505	-3.232295243	0.001228001	0.003296538
MPZL2	78.73840638	-0.655525013	0.202845717	-3.231643347	0.001230806	0.003303403
NOPCHAP1	28.08440999	0.896542343	0.277456762	3.23128669	0.001232343	0.003306865
EIF5B	808.4857096	0.592368442	0.183343361	3.230923874	0.001233908	0.0033104
KBTBD7	6.406332076	1.853069329	0.573904037	3.228883593	0.001242745	0.003333439
EIF5	261.6634226	0.483729786	0.149816241	3.22882075	0.001243018	0.003333502
EPPK1	13.33385715	1.188161242	0.368017621	3.228544437	0.001244219	0.003336055
LOC101107475	1.491699153	3.895424508	1.20678306	3.227940992	0.001246847	0.00334243
HM13	53.62843934	0.753787219	0.233524778	3.227868261	0.001247164	0.003342609
C2CD2	38.54555618	0.779754318	0.24157884	3.22774262	0.001247712	0.003343407
MED12L	2.58880296	3.456881478	1.071195294	3.227125342	0.001250407	0.003349957
ATG101	36.72165949	-0.82826435	0.256791979	-3.225429212	0.001257839	0.003369195
CISH	8.874490805	1.443487633	0.447673	3.224424154	0.001262263	0.003380366
LTBP4	5.801195174	2.323396936	0.72065306	3.224015917	0.001264064	0.00338451
TOGARAM1	35.26843839	-0.81310886	0.252319514	-3.222536562	0.001270609	0.003401355
SEC16A	42.2134086	-0.943755936	0.292918323	-3.221908162	0.001273399	0.003408141
TMEM254	28.16313767	-0.981034197	0.304495352	-3.221836357	0.001273719	0.003408313
HDAC6	14.44731694	1.226577437	0.380764084	3.221358022	0.001275847	0.003412768
SPINK1	455.0513717	-0.506986932	0.157383505	-3.221347311	0.001275894	0.003412768
RCL1	43.54473984	-0.789239028	0.245048851	-3.220741598	0.001278594	0.003419304
APP	81.9568809	-0.624486581	0.193934243	-3.220094459	0.001281484	0.003426339
TLDC2	1.738061861	4.1086681	1.275968891	3.220037831	0.001281737	0.003426339
ZBTB4	37.59697161	0.734401523	0.228122686	3.219327002	0.001284919	0.003434158
MAP2	44.88357208	0.690956724	0.214637499	3.219179909	0.001285578	0.003435233
PPP2R5E	168.5260125	-0.464446318	0.144324894	-3.218061027	0.001290604	0.003447973
GYG1	11.18897998	1.350087876	0.419613616	3.217454882	0.001293334	0.003454576
LOC105604950	4.367608063	-2.299074146	0.714702315	-3.216827617	0.001296165	0.003461446
DUSP9	1.708843736	-3.7936679	1.179389354	-3.216637397	0.001297024	0.003463049
GCHFR	6.391301114	1.998527801	0.621400318	3.21616797	0.001299148	0.003468026
HOXB5	1.92277419	4.248641063	1.321352113	3.215373873	0.001302747	0.003476941
SLC39A9	26.04031268	0.842040127	0.261904005	3.215071597	0.00130412	0.00347991
MIER3	23.90736853	0.949396443	0.295325552	3.214745343	0.001305603	0.003483171

INPP1	3.99739891	2.486834763	0.773708625	3.214174799	0.0013082	0.003489404
PIGL	16.07840407	-1.174078067	0.365306496	-3.213953429	0.001309209	0.003491398
NKG7	1.531965813	3.920826323	1.220219834	3.213213073	0.001312589	0.003499713
ITGB1	269.0100826	0.386616943	0.120349286	3.212457314	0.001316047	0.003508234
TNFRSF13B	4.082799311	2.759872034	0.85928211	3.211834626	0.001318903	0.003515145
ALS2	14.81414275	-1.11549917	0.347348321	-3.211471321	0.001320572	0.003518891
LAMA2	1.522464641	3.91816719	1.220504029	3.210286157	0.001326029	0.003532729
SDHC	93.90475481	-1.011600098	0.315127815	-3.210126339	0.001326766	0.00353399
TMEM238	17.41305282	1.086018099	0.338351154	3.209736646	0.001328566	0.003538079
IL2RA	1.469059874	3.918401353	1.221103894	3.208900874	0.001332434	0.003546966
OSGIN1	7.438243577	1.74794275	0.544713691	3.208920172	0.001332345	0.003546966
CD69	1.468990154	3.884114888	1.210975454	3.207426605	0.001339282	0.003563776
PLP1	1.468990154	3.884114888	1.210975454	3.207426605	0.001339282	0.003563776
COMMD4	11.08038784	1.350716449	0.421300374	3.206065158	0.001345635	0.003579256
LOC105604846	24.42386279	-1.063311058	0.331653318	-3.20609202	0.00134551	0.003579256
ST6GALNAC6	8.405974992	1.500969176	0.468196145	3.205855475	0.001346616	0.003581152
HNRNPUL1	81.2277486	0.566665082	0.176820537	3.204746978	0.001351813	0.003593542
MPPED2	1.695374943	4.082172259	1.273774993	3.204782857	0.001351644	0.003593542
LOC114113889	3.770793713	-2.630072254	0.820803288	-3.204266225	0.001354072	0.003598832
LOC101123281	16.09651799	-1.141763694	0.356642249	-3.201425792	0.001367493	0.003633779
DESI1	55.65648805	0.636732566	0.198911236	3.201088988	0.001369092	0.003637307
ADSL	43.83785726	-0.773670549	0.241745765	-3.200347887	0.001372618	0.003645948
COMMD6	19.86081417	-1.071422537	0.334811132	-3.200080385	0.001373893	0.003648609
ATP5F1C	44.71956763	-0.798834725	0.249661901	-3.199666116	0.001375869	0.003652405
IDE	112.9890601	0.503770121	0.157441925	3.199720278	0.00137561	0.003652405
DTNBP1	19.58297765	-1.049080288	0.327941638	-3.198984719	0.001379125	0.003660322
LOC101104401	17.30150408	1.104111844	0.345169678	3.198750972	0.001380243	0.003662564
FLT4	1.417300438	3.852090184	1.204306916	3.198595085	0.00138099	0.003663817
PPP1R13B	22.43479571	-0.987886422	0.308866551	-3.198424754	0.001381806	0.003665255
LOC132659218	2.768474666	3.13781623	0.98136851	3.197388338	0.001386781	0.003677721
GTF3C3	69.85334668	0.780110375	0.244000011	3.197173523	0.001387814	0.003679731
TAF1B	16.74366566	-1.09366466	0.342121998	-3.196709554	0.001390048	0.003684923
FOXO4	9.548696558	-1.389640015	0.434869654	-3.195532275	0.001395732	0.003699256
URB2	7.008367451	-1.730155385	0.541444321	-3.195444699	0.001396155	0.003699646
HDAC2	126.9977935	-0.487072821	0.152486454	-3.194203866	0.001402171	0.003714849
ARMC10	82.30271997	0.579837221	0.181551503	3.193789164	0.001404186	0.003719452
NIPSNAP1	14.04877368	-1.40316469	0.439398243	-3.193378016	0.001406187	0.003723756
SMAD9	11.90518409	1.215021537	0.380486013	3.193340876	0.001406368	0.003723756
GCC2	233.0383174	0.527813061	0.165379724	3.191522203	0.001415252	0.003746536
RIN3	13.99042433	1.256720271	0.393786679	3.191373242	0.001415982	0.003747727
METTL2A	10.83966344	-1.290251064	0.404384689	-3.190652607	0.001419519	0.003756342
TNFRSF13C	1.502072304	3.936912001	1.234168158	3.189931594	0.001423065	0.003764981

CYRIA	4.690648725	2.331615645	0.730960724	3.18979607	0.001423732	0.003765256
GNG2	5.082352618	1.978561074	0.620267951	3.18984895	0.001423472	0.003765256
N4BP1	72.78935033	0.700590092	0.219715846	3.18861886	0.001429542	0.003779874
OSGIN2	10.6011683	-1.265736347	0.39707146	-3.18767898	0.001434197	0.003791431
EIF2S2	31.39276911	-0.954219428	0.29938482	-3.187267243	0.00143624	0.003796082
LOC114118103	2.522386547	-3.57396992	1.121515395	-3.186732822	0.001438896	0.00380235
LOC114114247	1.461805645	3.913006213	1.228205621	3.185953675	0.001442777	0.003811852
LOC121818848	175.2421845	2.003033465	0.628725079	3.185865382	0.001443217	0.003812261
DYNC1I1	1.996962431	3.672661749	1.153025727	3.185238337	0.001446348	0.003819023
PAM	67.62962589	-0.73387308	0.230396496	-3.185261468	0.001446233	0.003819023
LOC105603042	2.35214826	3.347365124	1.050953628	3.185074045	0.00144717	0.003819682
NELFE	80.39577693	0.772130425	0.242419693	3.185097778	0.001447051	0.003819682
CD109	14.15118332	-1.190713803	0.373863523	-3.184888954	0.001448096	0.003821371
LOC101119987	7.788870304	-1.632482357	0.512815132	-3.183373998	0.001455695	0.003840666
POLR3E	13.91763983	-1.120708561	0.352066019	-3.183234111	0.001456398	0.003841006
RASA2	29.41773841	0.826139438	0.259525598	3.183267637	0.001456229	0.003841006
GJB3	1.34083315	-4.129841046	1.297514888	-3.182885286	0.001458154	0.003844877
MIGA2	12.64297889	1.23050326	0.386662402	3.182371115	0.001460745	0.003850951
LOC101106767	1.536170842	3.925472558	1.233651412	3.181994946	0.001462644	0.003855195
AACS	27.0494978	-0.856277664	0.269216719	-3.180625881	0.001469573	0.003872381
MRPL45	42.18473058	-0.723926952	0.227607592	-3.180592295	0.001469743	0.003872381
ADGRG5	1.467238903	3.916869458	1.231538686	3.180468061	0.001470373	0.003873177
CACHD1	28.10490108	0.795319337	0.250067512	3.180418481	0.001470625	0.003873177
ZNF697	10.62979334	-1.491558262	0.469009071	-3.180233291	0.001471565	0.00387489
ABHD14B	53.20873483	-0.725997459	0.228318432	-3.17975843	0.001473979	0.003880481
CEP83	81.92569488	0.717678249	0.225717098	3.179547571	0.001475052	0.00388254
XKR8	3.816597057	-2.364896026	0.743826159	-3.179366571	0.001475973	0.003884201
MYO7B	9.512200537	1.427398956	0.44899619	3.179089239	0.001477386	0.003887154
MED11	7.89874062	-1.542295596	0.485297974	-3.178038395	0.001482751	0.003900502
SMTNL1	1.522500842	3.919498749	1.233583757	3.177326813	0.001486394	0.003909317
YARS1	50.63369427	-0.629618748	0.198163732	-3.177265289	0.00148671	0.003909377
PRRG4	110.8053459	-0.644352903	0.202911994	-3.175528902	0.001495636	0.003932076
COPS2	78.85606844	0.571727184	0.180057396	3.175249643	0.001497076	0.003934573
SRBD1	32.47778848	-0.822420032	0.259011121	-3.175230577	0.001497175	0.003934573
RTN4RL2	1.488722355	3.894894127	1.226705468	3.175084997	0.001497926	0.003935774
TBX18	2.933771783	-2.975680794	0.937594638	-3.173739132	0.001504889	0.003953291
GPAT4	114.9724677	0.61611713	0.194227732	3.172137791	0.001513212	0.003974374
TRADD	6.954963782	1.886791209	0.594866737	3.171788053	0.001515035	0.003978382
SQOR	15.42864089	1.288984378	0.406499406	3.170937917	0.001519476	0.003989259
THRA	85.20774395	0.666780645	0.210330629	3.170154758	0.001523578	0.003999242
WDHD1	44.91318813	0.961940552	0.303443992	3.170076118	0.00152399	0.003999539
BCLAF1	488.5700722	0.489797098	0.154529138	3.169609976	0.001526437	0.004004388

LOC101111965	5.19411995	5.728166237	1.807207265	3.16962329	0.001526367	0.004004388
RBM26	200.5616918	0.608808889	0.192110878	3.169049529	0.001529383	0.00401133
AKR1E2	70.78938073	0.767066242	0.242062261	3.168879939	0.001530276	0.004012239
LOC132657869	1.97172962	3.661354309	1.15543415	3.168812614	0.00153063	0.004012239
YME1L1	18.72616165	-1.033034595	0.325999029	-3.168827211	0.001530554	0.004012239
KAT6A	268.8240643	0.623309156	0.196714872	3.16859193	0.001531793	0.004014499
COQ7	32.85239086	0.82407981	0.260229394	3.16674376	0.001541561	0.004038515
VPS26B	30.07918272	-0.952226105	0.30069314	-3.1667703	0.00154142	0.004038515
PDIA2	6.145057299	5.376375914	1.697863388	3.166553888	0.001542568	0.00404036
LOC132658791	1.414323639	3.849961834	1.215954967	3.16620429	0.001544423	0.004044427
ANKHD1	137.0495095	0.55013388	0.173773737	3.165805666	0.001546541	0.00404918
EEF2KMT	5.230375339	-1.963718901	0.620594682	-3.164253511	0.001554813	0.004070041
RNASEK	45.76271998	0.723005024	0.228496936	3.164178198	0.001555215	0.004070297
CFAP54	2.769032764	3.034544363	0.95907158	3.164043672	0.001555934	0.004070585
TMEM203	29.99055926	0.789590986	0.249546769	3.164100223	0.001555632	0.004070585
EPHA3	1.536207043	3.926330265	1.241089645	3.16361536	0.001558226	0.004075782
MCRS1	3.788281958	-2.551877594	0.806778624	-3.16304562	0.001561279	0.004082968
LOC105601843	3.046392389	-3.036154783	0.959920787	-3.162922217	0.001561941	0.0040839
AATK	2.107251919	3.665901912	1.159186033	3.162479367	0.001564318	0.004089317
LOC101119634	1.613965034	4.059469767	1.283804953	3.162061151	0.001566567	0.004094394
HOXB3	1.854340625	4.224358614	1.335976266	3.162001244	0.001566889	0.004094436
GON4L	191.0621621	0.860668969	0.272260067	3.161201637	0.001571197	0.00410489
MRPL28	15.19730295	1.184814539	0.374833034	3.16091281	0.001572756	0.00410816
LOC101114747	1.576365099	3.952729595	1.250684128	3.160453951	0.001575235	0.004113832
NHLRC1	9.538599233	1.340935614	0.42438996	3.159677985	0.001579436	0.004123997
METTL9	53.23495921	-0.642455888	0.203379941	-3.15889505	0.001583685	0.004134284
S100A16	13.28464745	-1.262932747	0.399812106	-3.158815675	0.001584116	0.004134603
EIF2B1	9.144464928	-1.39711992	0.442364393	-3.158301038	0.001586916	0.004140416
NCAPG	80.50659056	-0.700251296	0.221718329	-3.158292318	0.001586963	0.004140416
CNPPD1	13.61483184	-1.603258048	0.507659488	-3.158136676	0.001587811	0.004141819
IQCE	19.28809112	0.993733613	0.31469901	3.157727164	0.001590043	0.004146832
CASP9	13.27960325	-1.367635281	0.433210016	-3.156979824	0.001594124	0.004156664
DHRS7	33.81397341	1.009012527	0.319630131	3.156812924	0.001595036	0.004158232
TCHP	12.19529765	1.283706823	0.406667239	3.156651682	0.001595919	0.004159721
SCFD2	3.944439874	2.466221973	0.781426437	3.156051363	0.001599207	0.004167479
GMPR2	20.0750429	-0.990174689	0.313785531	-3.155577902	0.001601805	0.004173435
ZNF239	19.46951353	-0.960212911	0.304413878	-3.154300708	0.001608832	0.004190928
FAM91A1	58.11408525	-0.642243127	0.203657607	-3.153543512	0.001613012	0.004200997
DRG1	55.75444217	-0.628238409	0.199231164	-3.153313955	0.001614281	0.004203483
CUL5	42.00897011	0.754452316	0.239284295	3.152953758	0.001616274	0.004207853
SLC7A10	2.109358541	3.797236458	1.204375604	3.152867299	0.001616753	0.00420828
LOC121817434	2.558854741	3.432455695	1.088700733	3.152800022	0.001617125	0.004208431

GMEB2	19.32961757	-1.011486261	0.320950315	-3.151535338	0.001624145	0.004225876
FBXO33	14.54124962	1.271878623	0.403587904	3.151428994	0.001624737	0.004226592
SAMD4B	35.40865608	0.758855661	0.240819195	3.151142754	0.00162633	0.004229913
ISCU	34.72718096	0.975707259	0.30974531	3.150030773	0.001632533	0.00424522
RUFY1	7.679781734	1.697887081	0.539105539	3.14945212	0.001635769	0.004252809
POLD4	6.479846249	1.67984101	0.533524908	3.148570918	0.001640709	0.004264823
SKP2	3.869391279	-2.4495625	0.778025389	-3.148435174	0.001641471	0.004265974
PLD2	2.91532779	-3.281681757	1.042371216	-3.14828509	0.001642314	0.004267335
NFXL1	26.51557162	0.876294951	0.278405844	3.1475451	0.001646477	0.00427732
RNF114	20.485758	-1.053595021	0.334807604	-3.146867062	0.0016503	0.004286418
FBXL20	88.31712091	0.755569602	0.240112807	3.146727618	0.001651087	0.004287629
SARM1	8.058942586	1.536568685	0.488495865	3.145510116	0.001657975	0.004304679
DPF2	10.11428954	-1.759743309	0.559617089	-3.144548911	0.001663431	0.004318007
ZFAND3	23.13003093	-0.918011309	0.292014094	-3.143722606	0.001668135	0.004329377
GYS1	36.96294781	-1.046610499	0.333093096	-3.142096041	0.00167743	0.004351811
OSBPL1A	123.7663555	0.530106385	0.16870834	3.142146893	0.001677139	0.004351811
EHD2	3.7233184	2.467833818	0.785499798	3.14173705	0.001679488	0.004356304
NOP14	55.39357799	-0.685175893	0.218173599	-3.140507812	0.001686552	0.004373779
CEP126	1.827630541	3.592352512	1.144079923	3.139948915	0.001689773	0.004381281
LOC114110116	1.523692871	3.921965704	1.249132582	3.139751344	0.001690913	0.004383387
SF3B2	165.7876317	0.661767437	0.21078458	3.139543877	0.001692111	0.004385641
AIFM1	46.89566641	-0.716451794	0.228254632	-3.138826965	0.001696256	0.004395532
CDK9	69.38085952	-0.613707234	0.19556461	-3.13813033	0.001700293	0.004405139
TEX30	25.40501418	-0.867833975	0.276556923	-3.137994041	0.001701083	0.004406333
LOC121817543	1.396838381	3.837373981	1.223022128	3.13761615	0.001703278	0.004411163
RNF150	7.402798828	1.793051476	0.571750068	3.136075667	0.00171225	0.004433541
PPAT	7.297945256	-1.542370774	0.491873408	-3.135706765	0.001714405	0.004438261
SPIB	3.988541083	2.359157105	0.752507347	3.135061889	0.001718179	0.004447168
THEMIS2	1.848590971	3.600493355	1.148485846	3.13499149	0.001718591	0.004447374
ASPA	1.754569615	4.143665241	1.321855267	3.134734449	0.001720097	0.004450411
RAB25	6.355844093	-1.798817215	0.574063729	-3.133480004	0.001727467	0.004468613
IGDCC4	3.174321745	2.868615408	0.915584205	3.1330984	0.001729714	0.004473561
ENOPH1	40.86770861	-0.660854775	0.210934517	-3.132985462	0.00173038	0.004474417
GDPD3	7.439487872	1.717699618	0.548629161	3.13089376	0.001742752	0.004504813
JPT2	65.24714453	0.682427635	0.217966433	3.130884085	0.001742809	0.004504813
TOB2	264.3458677	0.644679013	0.205938583	3.130443086	0.001745428	0.00451071
CNOT10	12.00624272	-1.238643075	0.395756781	-3.129808848	0.001749201	0.004519586
METTL22	11.44577877	-1.296331659	0.414197964	-3.129739332	0.001749615	0.004519782
DISC1	1.377069979	3.827462107	1.223395061	3.128557756	0.001756665	0.004537117
ZNF436	10.16558617	-1.335167079	0.426778866	-3.128475151	0.001757159	0.004537516
TRAF6	13.20826346	-1.145093208	0.36609108	-3.127891582	0.001760651	0.004545656
NR1I3	3.418165806	2.629751189	0.840923458	3.127218255	0.001764689	0.004555201

ACTN2	1.492501413	3.902904835	1.248105925	3.127062179	0.001765626	0.00455674
FAM221A	5.188909614	-2.140537267	0.684561298	-3.126874503	0.001766753	0.004558769
PRRC1	56.84225373	0.664233345	0.212467312	3.126284886	0.0017703	0.004567038
TBRG1	93.51705377	0.676889783	0.216554025	3.125731713	0.001773633	0.004574754
GLRX	16.76734629	-1.247784681	0.399484087	-3.123490326	0.001787197	0.004608852
SLC19A1	11.15956835	-1.307219335	0.418528754	-3.123368046	0.00178794	0.004609878
RBBP9	7.763419649	-1.482266221	0.474834948	-3.121645171	0.001798436	0.004636044
ATAD2	72.26140822	0.854841359	0.273855908	3.121500519	0.001799319	0.004637428
MTFMT	7.839906483	1.53228537	0.490985322	3.12083743	0.001803376	0.004646091
NDUFAF5	13.1185164	1.182529631	0.378907926	3.120889142	0.001803059	0.004646091
MICOS10	22.2002635	-2.856873157	0.915524469	-3.120477119	0.001805583	0.004650882
ITGB3BP	8.769002986	-1.497328913	0.479921339	-3.119946524	0.001808839	0.00465837
TP53I3	3.1902564	2.860410362	0.916846944	3.119834101	0.001809529	0.00465925
SLC35G1	37.93754802	0.947789313	0.303809656	3.119681334	0.001810468	0.004659872
TREH	1.465938271	3.914743812	1.254847357	3.119697219	0.00181037	0.004659872
TFAP4	106.0698743	-0.624335695	0.200225694	-3.118159726	0.001819841	0.004683096
RGMB	58.09305906	-0.638580209	0.204837185	-3.117501382	0.001823911	0.004692665
DHCR7	53.70007529	-0.690992339	0.221653545	-3.117443217	0.001824271	0.004692687
BOC	1.470738723	3.886830329	1.246856339	3.117304061	0.001825132	0.004694
APOA1	5.632399651	2.159334051	0.692959632	3.116103669	0.001832578	0.004712244
LOC132657546	25.6688127	-0.929041641	0.298157694	-3.115940519	0.001833593	0.004712732
PLCXD2	23.09249139	0.970341474	0.311415819	3.115902971	0.001833826	0.004712732
SCUBE1	1.612597364	4.010212552	1.286984837	3.11597498	0.001833378	0.004712732
DNAJB2	6.1471591	1.728659351	0.554977281	3.11482904	0.001840516	0.004729014
AP2B1	96.68674091	-0.540049564	0.173408479	-3.114320397	0.001843692	0.004735354
TBL1XR1	305.5939514	0.413630146	0.132815	3.114333053	0.001843613	0.004735354
ZNF622	10.0251949	-1.537094828	0.493617586	-3.113938545	0.001846079	0.004740576
ADM2	4.120342245	-2.450064024	0.787178965	-3.11246125	0.001855344	0.004762536
MRS2	24.63780677	-0.919244199	0.295340558	-3.112488865	0.00185517	0.004762536
GPN2	9.714833672	-1.359044414	0.43665869	-3.112372303	0.001855903	0.004763056
S100A13	111.1889987	0.615184504	0.197761989	3.110731781	0.001866244	0.004788676
ZNFX1	29.76166306	0.861972504	0.277103154	3.110655694	0.001866725	0.004788991
LOC114108601	6.952455481	-1.910694283	0.614290498	-3.110408333	0.001868289	0.004792084
MRPL9	31.71869969	-0.830740286	0.267090327	-3.110334596	0.001868755	0.00479236
SEMA4C	8.538171468	-1.484770692	0.477392342	-3.110168641	0.001869806	0.004794134
VWA2	2.935860684	-2.968292592	0.954839243	-3.108683072	0.001879232	0.004817379
LANCL1	27.92571398	-0.840658899	0.270433015	-3.108566085	0.001879976	0.004818362
TRAPPC6A	8.129938758	-1.504272591	0.483953189	-3.108301848	0.001881658	0.004821748
FYB2	7.79867458	-1.486018363	0.478200336	-3.107522624	0.001886625	0.004833551
SH2D1A	1.427459064	3.859207588	1.242202205	3.10674669	0.001891584	0.004845326
NXPE3	3.874068576	2.403022301	0.773658407	3.106050784	0.001896041	0.004855813
MRPL16	9.658630492	-1.710030925	0.550596155	-3.105780726	0.001897774	0.004859319

NAA30	60.04087334	0.655505917	0.211207723	3.103607713	0.001911766	0.00489421
LOC101119619	7.993213285	-1.70468342	0.54936874	-3.102985838	0.001915788	0.004903566
TMEM165	56.89524267	0.734131465	0.236621037	3.102562117	0.001918533	0.004909651
TUSC3	5.418911282	-1.829720101	0.590002734	-3.10120614	0.001927341	0.004931247
C23H18orf32	67.92135983	-0.628178292	0.202587905	-3.100768984	0.001930188	0.004937588
LOC101107401	6.637546247	-1.8619608	0.600502867	-3.10066929	0.001930838	0.004938305
LDB1	135.8477876	-0.557978915	0.179972974	-3.100348361	0.001932932	0.004942714
PRADC1	35.62998364	0.802978594	0.259028312	3.099964584	0.001935438	0.004948176
DHX36	100.7357321	-0.512901334	0.165497812	-3.099142695	0.001940815	0.004960095
ZFYVE16	39.38670094	0.757061762	0.244281353	3.099138561	0.001940842	0.004960095
TATDN1	9.160354629	-1.327649848	0.428674686	-3.097103447	0.001954217	0.004993321
PSMG2	17.41904354	-1.046776437	0.338007278	-3.096905018	0.001955525	0.004995709
PHLPP2	8.674373499	1.542034839	0.497980003	3.09657984	0.001957671	0.005000236
LOC132659438	14.48624805	1.06597166	0.344265898	3.096361467	0.001959114	0.005002964
ANGPT2	1.901062311	3.64447828	1.177160689	3.095990474	0.001961567	0.005008271
SMTN	16.10222621	1.307282346	0.422375583	3.095070829	0.001967659	0.005022867
ZCCHC14	18.26665122	0.953547738	0.308219805	3.093726371	0.001976597	0.005044658
ZNF48	4.894164672	-2.013241403	0.650760821	-3.093673338	0.00197695	0.005044658
PIERCE1	27.37426126	-0.878953164	0.284132538	-3.093461836	0.00197836	0.005047291
CDH19	1.621636364	4.01740227	1.298936563	3.092839468	0.001982513	0.005056923
TPRA1	11.37686558	-1.280297357	0.414053198	-3.092108365	0.001987403	0.005068427
SLC30A10	3.277944889	2.618008891	0.84676708	3.091769807	0.001989671	0.005073243
TEX261	85.22490723	0.529476428	0.171264066	3.091579229	0.001990948	0.005075532
EIF5A	146.7858961	-0.565964033	0.183071745	-3.091487625	0.001991563	0.005076131
MOK	4.202253492	-2.367370454	0.766480749	-3.088623499	0.002010861	0.005124341
PSMD14	30.85689616	-0.824458246	0.267001494	-3.087841327	0.002016161	0.005135888
SAPCD2	121.1725786	0.538736845	0.174469311	3.087860225	0.002016033	0.005135888
HYCC1	39.91872403	0.717509411	0.232379614	3.087660747	0.002017386	0.005137052
SNRPG	6.840140266	-2.323685524	0.752563514	-3.087693572	0.002017164	0.005137052
MIF4GD	13.54020487	-1.235756464	0.400322097	-3.086905455	0.002022519	0.005149141
GABBR1	44.95103159	-0.859648836	0.278540777	-3.086258482	0.002026925	0.005159376
KPNA1	41.86877557	0.748880899	0.242731503	3.085223342	0.002033994	0.005176382
COL23A1	1.372795231	3.786853095	1.227542922	3.084904836	0.002036173	0.00517897
GRK6	19.46487722	-0.966945895	0.313440851	-3.084938968	0.002035939	0.00517897
RNF121	26.15565884	-0.874209545	0.283381341	-3.084922748	0.00203605	0.00517897
CACNB3	6.967644978	-1.620420895	0.525319956	-3.084636089	0.002038013	0.005182665
MCU	148.7162256	0.561889789	0.182187747	3.084125013	0.002041518	0.005190181
XG	1.819114562	3.556251668	1.153095252	3.084091848	0.002041746	0.005190181
DNAJC10	141.9890665	0.626121607	0.20307817	3.083155643	0.00204818	0.005205514
ENTREP1	5.940929351	1.709516084	0.554479436	3.083100966	0.002048556	0.005205514
LOC105603793	2.845710337	-2.858843307	0.927298315	-3.082981237	0.002049381	0.005206619
FLOT2	29.99775314	0.849306957	0.275503699	3.082742485	0.002051026	0.005209808

CCNG1	83.16664012	-0.917495398	0.297659243	-3.082368241	0.002053606	0.005214381
MYBPC2	9.460564671	1.546769767	0.501811897	3.082369659	0.002053596	0.005214381
TBCA	205.9323018	0.527058622	0.171005589	3.082113434	0.002055365	0.005217855
FCHO2	26.78842831	0.922937948	0.29947576	3.081845249	0.002057218	0.005221387
STEAP4	11.31263728	1.294491649	0.420044153	3.081798995	0.002057537	0.005221387
CNRIP1	2.495236777	3.375603307	1.095811215	3.080460631	0.002066807	0.005243914
EPS8L1	3.21370353	-2.625296707	0.852295579	-3.080265546	0.002068161	0.005246354
NLK	41.11136068	0.66740697	0.216686349	3.08006006	0.002069589	0.005248979
SIRPA	13.35260286	1.298603173	0.421695822	3.079478393	0.002073634	0.005258242
SANBR	7.227139596	1.639052112	0.532546568	3.07776298	0.002085607	0.0052876
XRCC1	7.203147732	-1.597315343	0.519017836	-3.077573127	0.002086936	0.005289966
LOC101105864	32.98157658	2.580643352	0.838574914	3.077415397	0.002088041	0.005291763
ASXL1	42.29117797	0.837405233	0.272119731	3.077341105	0.002088562	0.005292078
PPP6R3	12.59808427	1.19613043	0.388746844	3.076887819	0.00209174	0.005299128
LOC101107738	1.379977058	3.79251521	1.23266209	3.076686822	0.002093151	0.005301697
LOC101120381	47.14795224	-0.910565365	0.296042525	-3.075792452	0.002099439	0.005316617
LOC105614569	2.217881852	3.268337561	1.062798315	3.075218991	0.00210348	0.005325842
GIGYF2	110.8056309	0.561957596	0.182763562	3.074779191	0.002106585	0.005332691
C5AR2	1.555374656	4.017355293	1.306836267	3.074107594	0.002111333	0.005343699
GNB1	77.05011994	-0.557033615	0.181208098	-3.073999575	0.002112097	0.005344622
CD96	1.589787451	3.994193632	1.299592106	3.073420971	0.002116197	0.005353983
CD79A	1.542798453	3.985019027	1.296704947	3.073188727	0.002117845	0.005357138
PSAP	197.2081684	-1.272824997	0.41423561	-3.072707815	0.002121261	0.005364763
TMEM97	149.140576	0.504081828	0.16407887	3.072192223	0.002124928	0.005373022
AIF1L	4.79373542	2.234068317	0.727538798	3.070720522	0.002135429	0.005398553
WDR90	12.01558337	-1.183520338	0.385428813	-3.070658704	0.002135871	0.005398649
RALBP1	31.83644749	0.735815163	0.239643427	3.070458355	0.002137305	0.005401251
ATXN10	37.09267854	0.844173205	0.274987047	3.069865343	0.002141553	0.005410964
MYB	116.5375206	0.630623492	0.205428913	3.069789371	0.002142098	0.005411317
DHX29	84.25821177	0.747936914	0.243653495	3.069674471	0.002142922	0.005412376
MAP2K3	49.09307429	0.748205455	0.243749802	3.069563334	0.002143719	0.005413368
ANKRD39	8.66410042	-1.386789474	0.452111544	-3.067361344	0.002159576	0.005452069
FAHD1	26.82512904	-0.846626977	0.276015039	-3.067321915	0.002159861	0.005452069
HYPK	71.07887854	-0.822668266	0.268212635	-3.067224125	0.002160568	0.005452823
GPR34	1.415479467	3.850587509	1.255522988	3.06691916	0.002162773	0.005457359
KIF13B	85.51063288	0.614533168	0.20042178	3.066199536	0.002167986	0.005469479
PLA2G4B	5.229684481	-2.01214996	0.656299553	-3.065901768	0.002170146	0.005473895
OSBPL11	8.52325211	1.75074582	0.571221092	3.064918025	0.002177297	0.005490896
LRRC23	1.752645778	3.515971419	1.147962296	3.062793466	0.002192813	0.005528984
DSC3	1.449330537	-4.101628673	1.339416181	-3.062251099	0.002196791	0.005536924
ZNF143	18.58237062	1.087493284	0.355124557	3.062286916	0.002196528	0.005536924
FAM193B	46.58790435	-0.653007545	0.213256863	-3.062070475	0.002198117	0.005539222

LOC121817632	4.647381704	-2.051774062	0.670126048	-3.061773331	0.0022003	0.005542633
TPT1	149.7866477	-0.49036249	0.160155745	-3.061785203	0.002200213	0.005542633
LOC105606693	38.34857341	-0.891714781	0.291289763	-3.061263714	0.002204049	0.00555103
APLN	5.570535726	-1.907195658	0.623063265	-3.060998403	0.002206003	0.005553858
PNRC2	229.8799715	-0.477981732	0.156150383	-3.061034646	0.002205736	0.005553858
TADA2A	99.42291559	-0.598102976	0.195413675	-3.060701747	0.002208189	0.005558316
PI15	1.457461176	3.844796973	1.256425734	3.060106833	0.002212581	0.005568321
ZNF74	21.47903153	-1.01831399	0.332783584	-3.059988649	0.002213454	0.00556947
MGAT3	1.318056313	3.750474838	1.225781851	3.059659299	0.002215889	0.005574548
TUBB6	13.87580008	1.131776429	0.370018381	3.058703263	0.002222972	0.005591315
GPX1	27.70688646	-1.059034714	0.346333954	-3.057842589	0.002229366	0.005605288
TIMD4	1.426197315	3.824305758	1.250653217	3.057846656	0.002229336	0.005605288
CHST11	2.613588209	3.019112887	0.98765814	3.056839979	0.002236837	0.005621955
SOS2	27.91566484	0.829173697	0.271247481	3.056889944	0.002236464	0.005621955
ABCD4	6.572331628	1.773544113	0.580230828	3.056618208	0.002238492	0.005624708
PLCG2	9.293908514	1.399184088	0.457761241	3.056580511	0.002238773	0.005624708
CUL7	33.05220214	-0.850490009	0.278282543	-3.056210421	0.002241539	0.005630597
ABTB3	1.415549187	3.881092522	1.270500141	3.054775358	0.002252292	0.005656544
RMDN3	16.87759488	1.079082393	0.353262521	3.054618956	0.002253466	0.005658431
LOC101109901	7.496629676	-1.449910143	0.474707594	-3.054322622	0.002255694	0.00566296
ZNF84	13.61294722	-1.209333818	0.396098481	-3.053114001	0.002264799	0.005684751
NFYB	42.79467794	-0.630747108	0.20672508	-3.051139749	0.002279744	0.005721191
DIS3L	37.44542041	-1.047688478	0.343428562	-3.05067369	0.002283286	0.005729003
PTCH1	12.38868456	1.252075218	0.410554108	3.049720351	0.002290545	0.005746139
ABLIM3	6.084701981	-1.67502116	0.549317141	-3.049278889	0.002293914	0.005753511
BCCIP	20.65113646	-1.13863209	0.373518249	-3.04839748	0.002300654	0.00576825
LOC101113583	250.0394965	0.456613103	0.149786182	3.048432748	0.002300384	0.00576825
FZD8	1.755409047	3.523483972	1.156259574	3.047312258	0.002308977	0.005788032
MSH3	19.74596897	0.903318034	0.296453937	3.047077206	0.002310783	0.005791474
CDC42SE2	25.76628505	1.017395736	0.33400184	3.046078235	0.002318475	0.005809662
LOC121817754	4.414451081	-1.977224281	0.64914292	-3.045899783	0.002319851	0.005812021
COG2	35.20757387	0.799309704	0.26257012	3.044176184	0.002333185	0.00584433
ARHGAP4	4.638468345	2.027898318	0.666225188	3.043863179	0.002335613	0.005849318
ZNF821	6.27677722	-1.779620308	0.584708313	-3.043603567	0.00233763	0.00585327
MLH3	9.03875795	1.494581491	0.49108733	3.043412854	0.002339112	0.005855885
PPP2R3C	22.72944811	-0.871520848	0.28637884	-3.043244564	0.00234042	0.005858064
ETV1	17.23490298	1.159926759	0.381207039	3.042773714	0.002344085	0.005866138
PHF20	64.68734981	0.694791855	0.228371281	3.04237841	0.002347166	0.005872749
FRMD5	3.363456586	2.595867176	0.853337311	3.042017668	0.002349981	0.005878691
LOC101106042	21.54936505	-0.877293517	0.288397945	-3.041954809	0.002350472	0.005878818
LOC121819684	2.328739433	3.265423948	1.07353323	3.041753955	0.002352041	0.005881642
SALL1	1.375699627	3.789797885	1.246046796	3.041457108	0.002354361	0.005886343

CLCN4	7.524512175	-1.48843469	0.489501641	-3.040714401	0.002360176	0.005899543
KDM5C	28.53816435	0.782709547	0.257413506	3.040670083	0.002360523	0.005899543
LOC114116085	8.450489894	-1.521641229	0.500467748	-3.040438142	0.002362342	0.005902985
KDM6A	10.42548548	1.248941101	0.410977838	3.038949998	0.002374043	0.005931113
CEBPE	1.403524517	3.811606631	1.255171698	3.036721301	0.002391665	0.005974022
UTP3	65.81145509	-0.646201613	0.21282477	-3.036308293	0.002394944	0.005981094
CEMIP	5.86565777	-1.910857616	0.629446472	-3.035774607	0.002399186	0.005990287
HGF	6.755767586	1.579898287	0.52043395	3.035732559	0.002399521	0.005990287
LOC105603226	2.226850432	-3.416382146	1.125590974	-3.035189713	0.002403844	0.005999959
RNF213	47.46569589	0.844372296	0.27824276	3.034660441	0.002408066	0.006009375
MTAP	8.527639005	-1.618840989	0.533515862	-3.034288396	0.002411038	0.006015669
APRT	78.13400882	0.648928123	0.213908299	3.033674362	0.002415951	0.0060268
LOC101109345	26.37288816	-0.982546802	0.323905029	-3.033441025	0.00241782	0.006030337
LOC132659427	3.454991736	2.553001118	0.84180893	3.032756041	0.002423314	0.006042914
NDUFB3	40.71498826	-1.052343353	0.347112536	-3.031706565	0.002431755	0.00606283
LRRC8A	18.48192512	1.054465812	0.347853203	3.031352887	0.002434605	0.006068805
THEMIS	1.397495835	3.840693665	1.267101763	3.031085409	0.002436763	0.006073052
IVD	45.39385608	0.777062342	0.25637959	3.030905632	0.002438214	0.006075536
SSR1	192.912414	0.486647328	0.160619663	3.029811664	0.002447063	0.006096448
THUMPD2	7.165758818	1.605701196	0.530067621	3.029238406	0.002451711	0.00610689
RSPRY1	10.21417726	1.263462988	0.417117296	3.029035237	0.002453361	0.00610986
NOXA1	28.39604199	-0.762372059	0.251719068	-3.028662331	0.002456391	0.006116266
LOC121816195	2.573209125	2.959090142	0.977139248	3.02831981	0.002459177	0.006120923
ZNF512	35.2968389	0.802435653	0.264975754	3.028336138	0.002459044	0.006120923
LOC121815980	13.23873351	-3.276832872	1.08223667	-3.027833895	0.002463134	0.006129632
SART3	69.81461664	0.739774119	0.244351782	3.027496308	0.002465887	0.006135341
PRR3	10.60630888	-1.303375072	0.43069915	-3.026184453	0.002476612	0.006159731
RHOBTB3	217.6781679	0.455401419	0.150484651	3.026231693	0.002476225	0.006159731
THY1	1.44134627	3.86608104	1.277877088	3.025393504	0.002483098	0.006174715
ZBTB8B	2.923057855	-2.891057167	0.955638146	-3.025263463	0.002484166	0.006176221
CLEC4E	1.378857432	3.792506894	1.253879207	3.024619017	0.002489465	0.006188244
ENTPD2	6.113798896	-1.744191245	0.576710472	-3.024379357	0.002491438	0.006191997
LOC132657378	4.992383151	2.15483018	0.712525081	3.024216603	0.002492779	0.006194178
MGAM	4.17789203	3.301330083	1.091694916	3.024040905	0.002494227	0.006195673
ZYG11B	92.75102565	0.572540836	0.189330337	3.024031141	0.002494308	0.006195673
PHACTR4	44.69741426	0.640903414	0.212072789	3.022091702	0.002510345	0.006233436
WDR73	9.287377626	-1.408051496	0.465921346	-3.022079817	0.002510443	0.006233436
KLF1	1.830859753	3.558336335	1.177568917	3.021764826	0.002513057	0.006238767
FRZB	2.381933166	3.334262537	1.103499859	3.021534176	0.002514972	0.006242363
CRY2	13.99900002	-1.168492238	0.386758887	-3.021242118	0.0025174	0.006247227
CFAP58	1.345845002	3.771423635	1.248412975	3.020974396	0.002519627	0.006250512
MCEMP1	1.376961376	3.825289186	1.266245107	3.020970558	0.002519659	0.006250512

HOXA2	1.617434017	3.992377421	1.321961485	3.020040648	0.002527408	0.006268571
TMEM247	1.406537517	3.814407838	1.263388792	3.01918765	0.002534535	0.006285082
LOC101113072	15.35593921	-1.406591028	0.465932473	-3.018873139	0.002537167	0.006290443
DENND2A	4.024740599	2.314374503	0.766684866	3.018677695	0.002538805	0.006293335
LOC114112783	3.489674989	-2.549206018	0.8444982	-3.018604442	0.002539419	0.00629369
AHR	19.25733995	1.120634117	0.371256879	3.018487148	0.002540402	0.006294959
ARMC12	5.804747946	1.798897639	0.596001257	3.018278266	0.002542154	0.006298133
NDUFB6	2.840167543	-3.74813607	1.242013697	-3.017789642	0.002546256	0.006307127
DCTN6	9.51974914	-1.301847121	0.431526362	-3.016842621	0.002554224	0.006325691
ZNF106	131.7199095	-0.475763318	0.157717842	-3.016547217	0.002556714	0.006330685
LOC101119303	2.141142441	-3.386136856	1.12254595	-3.01647951	0.002557285	0.006330926
ANAPC1	48.69508524	-0.641010234	0.212510168	-3.016374426	0.002558172	0.006331948
LOC132659507	19.05621723	-0.9373804	0.310779985	-3.016218696	0.002559486	0.006334028
PTPN2	43.02228725	-0.655517539	0.217338041	-3.016119669	0.002560322	0.006334925
RAB11B	34.30890863	-0.901366577	0.298897263	-3.015640118	0.002564374	0.006343777
LOC121817439	4.766098144	2.118175116	0.70242582	3.015514314	0.002565438	0.006345235
LOC105607154	1.703025583	-3.589434582	1.19124308	-3.013183996	0.002585221	0.006392982
ARHGEF5	40.63090688	-0.759940752	0.252244962	-3.012709338	0.002589268	0.006401804
HS3ST1	63.90775156	-0.748603917	0.248491244	-3.012596758	0.002590228	0.006401811
SCRIB	35.07490607	-0.744435374	0.247103444	-3.012646687	0.002589802	0.006401811
FAT3	1.948430519	3.654657874	1.2132155	3.012373212	0.002592137	0.006405343
WDR41	13.57344043	-1.197050478	0.397440789	-3.011896391	0.002596212	0.006414227
LTF	43.58922505	2.191226498	0.727654194	3.011356929	0.002600829	0.006424447
LOC101110758	4.038073496	-2.942083562	0.977032189	-3.011245273	0.002601786	0.006425623
YY1	47.03615644	-0.659406831	0.219023906	-3.01066145	0.002606793	0.0064368
ERBB3	59.68488374	-0.69737503	0.23166877	-3.010224595	0.002610546	0.006444875
PAK1IP1	55.83464339	0.720198993	0.239355133	3.00891393	0.002621834	0.006471168
PEX14	18.46894108	-0.986878606	0.327989162	-3.008875659	0.002622164	0.006471168
TBRG4	55.93151589	-0.65411964	0.217442008	-3.00824871	0.00262758	0.006483337
MYRF	12.56222081	-1.174528583	0.390474559	-3.007951624	0.00263015	0.00648848
KLF7	19.37580037	0.926446436	0.308034266	3.007608371	0.002633122	0.006494614
CPNE8	1.793775829	3.513616646	1.168290247	3.007486072	0.002634182	0.006496029
ASB1	60.39075953	-0.645198843	0.214583543	-3.006748949	0.002640577	0.006510599
ACTR3B	3.315457565	-2.450786875	0.815165614	-3.006489518	0.002642832	0.006514955
CC2D2A	12.37891255	1.42877471	0.4752546	3.006335362	0.002644172	0.006517057
ING4	27.69925455	0.864830768	0.287700641	3.006009186	0.00264701	0.00652285
FAM89A	3.066070244	-2.655156402	0.883478639	-3.005343068	0.002652814	0.006535948
CABLES2	7.370826245	-1.527482857	0.508308987	-3.005028231	0.002655562	0.006541512
FDX1	67.09859128	-0.722657704	0.240522528	-3.004532295	0.002659895	0.006550978
MAFB	2.8666322	2.809874851	0.935520956	3.003540253	0.002668582	0.006571163
TSTD2	21.57213248	-0.859362691	0.28613036	-3.003395693	0.002669851	0.006573075
PFDN6	22.48040547	-0.998372496	0.332483014	-3.002777446	0.00267528	0.006585229

CCDC138	31.72779463	-0.80598067	0.268479207	-3.002022683	0.002681922	0.006600362
TSPAN31	30.84126034	-0.816980338	0.272157674	-3.001864049	0.00268332	0.006602587
ENTR1	27.66617758	-0.95109817	0.316879797	-3.001447804	0.002686991	0.006609187
MTA1	42.35997885	-1.001793519	0.333768291	-3.001464028	0.002686848	0.006609187
NAA10	10.08898463	-1.474159767	0.491248448	-3.00084361	0.002692328	0.006621096
DNAJB9	9.47668522	1.375978989	0.458638708	3.000137066	0.002698581	0.006635254
MEF2A	125.6415055	0.509475686	0.169834965	2.999828008	0.002701321	0.006640768
SFTA2	2.130385543	-3.365495508	1.122453556	-2.99833832	0.002714561	0.006672091
EPHA4	1.759614075	3.526066496	1.176601637	2.9968227	0.002728093	0.006704117
APBB3	2.516438192	-3.043990375	1.016125021	-2.995684892	0.002738292	0.006726711
ST14	189.5599446	-0.574080283	0.191635748	-2.995684725	0.002738294	0.006726711
LDLRAP1	36.92988766	-0.703290329	0.23477947	-2.995535884	0.002739631	0.006728758
KIF3A	112.7511919	0.671678902	0.224265786	2.995012805	0.002744333	0.00673907
MAGED2	15.57509129	-1.086076722	0.362797498	-2.993616902	0.002756919	0.006768732
LOC101119153	1.297026163	3.69816017	1.23549135	2.993270791	0.002760047	0.006775169
ARRB1	34.82066978	0.758138793	0.253292358	2.993137252	0.002761255	0.006776889
ACYP1	7.563091626	1.597093871	0.533816034	2.9918432	0.002772987	0.006804431
TRIM10	7.928394631	1.610880729	0.538504832	2.991395126	0.002777059	0.006813174
BVES	1.308591342	3.747097149	1.252692589	2.991234388	0.002778521	0.00681551
NUP160	19.82003578	-0.930793329	0.311190768	-2.991069869	0.002780019	0.006817932
CHAT	1.694148438	3.461358429	1.157513137	2.990340515	0.002786666	0.006832981
ZBTB7A	552.324444	0.489184937	0.163595982	2.99020141	0.002787936	0.00683484
LOC114113859	1.951334915	3.657165818	1.223493744	2.989116892	0.002797851	0.00685789
HSF5	5.558088729	1.787548284	0.598179207	2.988315644	0.002805197	0.006874635
LOC114117363	1.741153001	3.475772939	1.163170769	2.988188004	0.002806369	0.006876246
HLCS	23.32914823	-0.877657426	0.293719019	-2.988085101	0.002807314	0.006877301
SI	2.164480815	3.727153822	1.247379046	2.98798816	0.002808204	0.006878222
IL2RG	2.294549968	3.238162323	1.084408181	2.986110193	0.002825508	0.00691926
SLC16A12	2.671719902	2.903702583	0.972420158	2.986057579	0.002825994	0.00691926
LOC121818601	2.620266139	-3.163345742	1.05948098	-2.985750384	0.002828834	0.006924945
LOC132658132	1.801951438	3.541170218	1.186058933	2.985661267	0.002829659	0.006925695
ALKBH8	7.444393795	-1.574278248	0.527556028	-2.984096789	0.002844168	0.006958658
MEPCE	59.82949552	-0.609023498	0.204087921	-2.984123186	0.002843923	0.006958658
ADIRF	7.493054912	1.485937052	0.498048093	2.983521218	0.002849523	0.006970483
PTGS2	4.020927306	2.491119692	0.835188887	2.982702152	0.002857159	0.006987884
FOXP4	7.562048112	-1.54280019	0.517391696	-2.981880461	0.002864838	0.007005384
FUCA1	10.57398646	-1.177934445	0.395054734	-2.981699351	0.002866534	0.007008247
ALKBH4	23.04634758	-0.946061637	0.317353015	-2.981101779	0.002872133	0.007020653
SPAG16	1.288020682	3.73127049	1.251842517	2.980622914	0.002876628	0.007030354
ASNS	58.89865352	-0.593343414	0.199094911	-2.980203813	0.002880567	0.007038693
CIC	17.1455342	-1.207707738	0.405450174	-2.978683485	0.002894896	0.007072416
U2SURP	300.5251054	0.445488244	0.149572543	2.978409235	0.002897488	0.007077454

SNX8	11.27309585	1.258102273	0.422501974	2.977742947	0.002903794	0.007091561
LOC106990841	5.303861619	1.845012146	0.619833696	2.976624467	0.002914408	0.00711618
RBIS	11.90903126	-1.539626104	0.517258036	-2.976514615	0.002915452	0.00711743
TMEM101	15.89056024	-1.012427802	0.340194053	-2.97603028	0.00292006	0.007127379
DBN1	12.8093656	-1.225048699	0.411648	-2.975961739	0.002920713	0.00712767
RHBG	8.065843074	1.448772033	0.486856187	2.975769998	0.00292254	0.007130826
RGMA	6.433496486	1.612453982	0.542016229	2.974918268	0.002930666	0.007149349
ADARB1	5.285534599	1.868078415	0.628040856	2.974453649	0.002935108	0.007158878
LOC101114861	1.688119756	3.490531491	1.173720151	2.973904374	0.002940367	0.007170397
MED21	14.43510326	-1.16108695	0.39046383	-2.973609491	0.002943194	0.007175981
LOC106990546	29.76012725	-0.723104165	0.243257559	-2.972586627	0.002953019	0.007198623
RNGTT	37.4306671	0.704684676	0.237119425	2.971855543	0.002960059	0.00721447
NAP1L1	176.8463234	-0.551929025	0.185812982	-2.970346945	0.002974636	0.00724797
ORMDL3	16.39574193	-1.212394407	0.408169512	-2.970320834	0.002974889	0.00724797
LOC101104046	4.153267001	2.178431579	0.733485312	2.969973009	0.002978259	0.007254859
ACP2	24.8034397	0.84564798	0.284746959	2.969822687	0.002979717	0.007257087
FZR1	21.23321894	0.894716984	0.301658065	2.965997227	0.003017032	0.00734663
NCF2	3.441145103	2.340399114	0.789212269	2.965487493	0.003022036	0.007357475
LOC132657203	2.257125359	3.241007867	1.093176346	2.964762161	0.00302917	0.0073735
UTP14A	4.135135939	-2.059967605	0.694847036	-2.964634657	0.003030426	0.007375213
PFKP	29.01678223	-0.839317231	0.2831413	-2.964305211	0.003033672	0.00738177
USP6NL	47.39174917	-0.616404881	0.207947056	-2.964239519	0.00303432	0.007382003
ATL2	33.54439502	0.797486214	0.269050656	2.964074597	0.003035947	0.007384616
SIGLEC11	1.832252669	3.53869464	1.193884079	2.96401862	0.003036499	0.007384616
ZNF668	4.903710981	-2.037993344	0.687719869	-2.96340623	0.003042547	0.007397979
SCRN3	14.44803593	1.060862637	0.358056291	2.962837583	0.003048174	0.007410311
RSPH1	14.94023804	1.150841694	0.388463566	2.962547311	0.003051049	0.007415953
NFIL3	13.80246245	-1.150280767	0.388294848	-2.962389977	0.003052609	0.007418395
LEO1	66.26360291	0.560150851	0.189096758	2.962244603	0.003054051	0.007420549
C21H11orf54	42.5405812	0.643263373	0.217170922	2.962014279	0.003056336	0.007422055
NKAPD1	38.54853692	0.762670047	0.257480978	2.962044235	0.003056039	0.007422055
NSD3	210.7054827	0.498818289	0.168399285	2.962116429	0.003055322	0.007422055
EIF2AK4	54.70295163	0.737571665	0.249040531	2.961653123	0.003059923	0.007429415
ZRANB1	34.50018535	0.780565996	0.263572855	2.961480973	0.003061634	0.00743222
VPS33A	20.13169336	-0.92275396	0.311606297	-2.961281494	0.003063618	0.007435685
IPP	14.03924826	-1.057651994	0.357167625	-2.961220223	0.003064228	0.007435814
ADRB1	2.189672242	3.258358988	1.100561669	2.960632812	0.003070077	0.007448657
MBD1	55.54555057	0.648987886	0.219307051	2.959265935	0.003083729	0.00748042
LOC114113042	14.64102269	-1.161079184	0.392398619	-2.958927803	0.003087114	0.007487274
VPS13A	44.10444534	0.710644882	0.240198661	2.958571369	0.003090687	0.007494579
RNASEH2B	35.67606032	-0.713585299	0.241197333	-2.95851239	0.003091278	0.007494653
GTF3C5	5.908291917	-1.651288926	0.558187109	-2.958307167	0.003093337	0.007498285

CNTFR	1.60299796	3.984064302	1.346806899	2.958155549	0.003094859	0.007500614
CTNNA3	1.38477751	3.762993145	1.272249541	2.957747693	0.003098957	0.007509183
UTP15	14.45040046	-1.040863802	0.35212074	-2.955985503	0.003116717	0.007550851
NOC4L	11.35649072	-1.198729868	0.405550479	-2.955809274	0.003118499	0.007553797
WARS1	69.66019469	0.599544447	0.20289287	2.954980365	0.00312689	0.00757275
PWWP3B	1.35477808	3.742414148	1.266731461	2.954386358	0.003132915	0.007585968
LOC114118133	1.582351194	-3.628766412	1.228477192	-2.953873655	0.003138125	0.007597206
ZNF214	14.19116867	-1.118563629	0.378689487	-2.953775235	0.003139125	0.007598253
LOC101106751	31.28822584	-0.750926466	0.254262577	-2.953350332	0.00314345	0.007607342
LOC106990119	5.046047101	2.176979097	0.737176054	2.953133222	0.003145662	0.007611317
LOC132658174	10.94995709	-1.145458419	0.387931372	-2.952734689	0.003149725	0.00761977
SRPRA	56.18908686	0.723637883	0.245098619	2.95243558	0.003152778	0.007625776
LOC114114156	1.627628845	4.004011994	1.356264934	2.952234401	0.003154833	0.007629366
FAM167B	1.676590778	3.450985117	1.16898186	2.952128886	0.003155912	0.007630593
CYTH4	1.249613877	3.665774528	1.241985908	2.951542771	0.003161908	0.007643707
LOC105606221	16.74620079	-1.018954662	0.345244077	-2.951403746	0.003163331	0.007645766
UBN1	202.2984742	-0.479880313	0.162602095	-2.951255411	0.003164851	0.007648056
MRPL24	101.0098529	0.522825427	0.177195988	2.950548902	0.003172098	0.007664184
PLSCR3	3.660958053	-2.318598093	0.78598426	-2.949929423	0.003178465	0.007677517
TRPC4	1.246564676	3.663425625	1.241881194	2.949900234	0.003178766	0.007677517
TESPA1	1.345184865	3.79964894	1.288245031	2.949476886	0.003183124	0.007686655
ROM1	24.85859928	0.971567243	0.329435267	2.949190147	0.003186079	0.007692401
KYNU	1.737959989	3.512739756	1.191135373	2.949068457	0.003187334	0.00769404
CWC27	140.6641569	0.564704997	0.191501757	2.948824103	0.003189855	0.007698736
LOC101113488	9.958848412	-1.23307706	0.418281068	-2.947962872	0.003198755	0.007718822
NTAQ1	14.14315832	-1.271373528	0.431298345	-2.947782067	0.003200626	0.007721071
PRRT4	1.847833717	-3.750585778	1.272350601	-2.947761233	0.003200842	0.007721071
CERS4	1.339158865	3.795095733	1.288088497	2.946300462	0.003215998	0.007754832
LOC132659288	2.06596395	-3.405069686	1.155704009	-2.946316409	0.003215833	0.007754832
TRAPPC6B	6.787538602	-1.825548506	0.619706964	-2.945825383	0.003220942	0.007765351
DNAJC19	14.05513739	-1.081852006	0.367271089	-2.945649788	0.003222771	0.007768359
PRXL2C	8.940571914	1.464524114	0.497220967	2.94541906	0.003225175	0.007772754
HARBI1	11.32165858	1.209315702	0.410646934	2.944903764	0.003230551	0.007784307
YTHDC1	339.9287893	0.544693633	0.184976285	2.944667377	0.00323302	0.007788852
ZNF473	18.58486343	-0.959011096	0.325698507	-2.94447495	0.003235031	0.007792292
PEX16	34.81142564	0.707411019	0.240263366	2.94431495	0.003236704	0.007794917
USP16	158.3846081	-0.471663061	0.160216588	-2.943909037	0.003240952	0.007803742
MAN2B1	15.86486499	-1.064479948	0.361615422	-2.943679622	0.003243356	0.007808121
AP2A1	17.95306568	-0.969832466	0.329544108	-2.942951926	0.003250989	0.007825089
LOC105611345	3.630866398	2.531848866	0.860603366	2.941946273	0.003261565	0.007849132
LOC121816067	11.10497693	1.384590415	0.47065925	2.941810696	0.003262993	0.007851155
MYOF	19.05420852	0.925499287	0.31477835	2.940161818	0.003280409	0.007891639

PELI1	42.89589126	0.688848624	0.234294307	2.940099708	0.003281066	0.0078918
SLC28A1	1.68714394	3.450800662	1.174061371	2.939199558	0.003290611	0.007913334
IER2	147.059424	-0.583996108	0.198710321	-2.938931928	0.003293454	0.007918746
NAGPA	6.14244346	-1.678488111	0.57128735	-2.938080302	0.003302515	0.007939103
LOC132658008	2.208324838	3.311762925	1.127352635	2.93764597	0.003307144	0.007948803
ZNF623	8.621587041	-1.498742493	0.510237153	-2.937344889	0.003310357	0.007955094
APBA2	1.675394151	-3.671558271	1.250339652	-2.936448721	0.003319937	0.007976681
AP5B1	5.85918584	-1.634974233	0.556813178	-2.936306641	0.003321458	0.007978902
IFT80	17.08450116	-1.050250875	0.357736111	-2.935825715	0.003326612	0.007989846
ARHGAP35	63.22028591	-0.677902274	0.230979465	-2.934902786	0.003336522	0.008009437
INSIG1	32.60622399	0.72715575	0.247756244	2.934964374	0.00333586	0.008009437
LOC121818687	2.848189774	3.047166551	1.038252732	2.934898658	0.003336566	0.008009437
PTTG1	38.91799807	-0.779563379	0.265791913	-2.932983819	0.003357214	0.008057554
ZBP1	3.684076175	2.245041481	0.765478104	2.932861787	0.003358534	0.008059275
C3H2orf49	31.68444308	-0.827818389	0.282293807	-2.932470956	0.003362764	0.008067977
PER2	5.878443475	-1.610964575	0.549405202	-2.932197529	0.003365726	0.008072901
TFEB	21.2419483	-0.87105821	0.297069473	-2.932170044	0.003366024	0.008072901
LRAT	5.274476729	2.592561623	0.884713392	2.930397173	0.00338529	0.008117651
LOC114117868	3.55634607	-2.388610798	0.815306158	-2.929710236	0.003392782	0.008133745
LOC132659671	2.970451192	2.716792414	0.927337268	2.929670259	0.003393219	0.008133745
NOP16	68.71764675	-0.564839626	0.192838533	-2.929080712	0.003399661	0.008147727
LOC132658362	1.905777163	3.635212358	1.241226046	2.928727101	0.003403531	0.008154162
TPPP	39.44251206	0.8101803	0.276632534	2.928723851	0.003403567	0.008154162
LOC121817368	5.127235564	1.845511796	0.630351783	2.92774899	0.003414256	0.008178305
MEIS1	4.649943104	1.893200391	0.646757075	2.927220224	0.003420066	0.008190755
POMT2	32.61423487	-0.735614851	0.251351204	-2.926641444	0.003426437	0.008204542
MPRIP	22.21787988	0.940467136	0.321409273	2.926073437	0.003432699	0.008218065
HSPD1	80.95142591	-0.553461097	0.189153811	-2.925984382	0.003433682	0.008218945
NCOR2	36.00145	-0.739491702	0.252774638	-2.925498018	0.003439054	0.00823033
NCSTN	87.66020498	-0.625555795	0.213869375	-2.924943291	0.00344519	0.008243539
PKD2	3.518805503	2.235762604	0.764428948	2.924748741	0.003447344	0.008247218
GNG5	35.34200985	-0.743019614	0.254070984	-2.924456786	0.00345058	0.008252004
ZNF593	75.40367984	-0.595382039	0.203584483	-2.924496168	0.003450143	0.008252004
KIF20A	10.40267276	-1.548993388	0.529717593	-2.924187166	0.00345357	0.008257678
SMIM29	13.77133126	1.081904346	0.37007888	2.923442557	0.003461841	0.008275973
SLC26A2	135.9907672	0.516684312	0.176841745	2.921732718	0.003480901	0.008320051
DPCD	7.400687464	-1.501992313	0.514144997	-2.921339935	0.003485293	0.008329059
PLCB4	25.67077968	1.014928014	0.347426399	2.921274891	0.003486021	0.008329309
WASHC1	26.23966455	-0.919574706	0.314802365	-2.921117527	0.003487782	0.008332028
CSGALNACT1	4.044439987	2.084075695	0.71346669	2.921055354	0.003488478	0.008332202
NSUN6	22.64974457	-0.907326674	0.310656507	-2.920674938	0.00349274	0.008340891
ACTN1	185.6191006	0.4833784	0.165508825	2.920559671	0.003494033	0.008342487

LOC101111547	133.8241723	0.602807642	0.206409369	2.920447094	0.003495295	0.008343278
VPS37A	12.50252945	-1.153065709	0.394828885	-2.920418826	0.003495612	0.008343278
KDF1	9.43755424	-1.36319502	0.466875185	-2.919827536	0.003502251	0.008357631
ICE1	25.95727587	0.776343273	0.265936983	2.919275333	0.003508462	0.008370957
RBBP7	99.49906229	-0.584315175	0.200168461	-2.919117092	0.003510243	0.008373713
IFFO1	2.192576954	3.210645861	1.099983936	2.918811589	0.003513685	0.00838016
MPHOSPH10	72.27386027	0.527623608	0.180769415	2.918765915	0.0035142	0.00838016
RPS17	734.4784287	-0.461968687	0.158343303	-2.917513263	0.003528346	0.008412394
OTUB1	24.41478465	-0.915208669	0.3138073	-2.91646711	0.0035402	0.008439151
HP1BP3	101.8275158	0.599587205	0.205669212	2.915298787	0.003553482	0.0084693
UBASH3B	2.649776326	2.89856122	0.994512982	2.914553426	0.003561978	0.008488038
AFF2	1.483913032	3.873585783	1.329128129	2.914381013	0.003563946	0.008491213
KANK4	5.446178657	2.09280543	0.718623813	2.91224058	0.003588461	0.008548097
TMED7	124.7948423	0.717849712	0.246541174	2.911682869	0.003594874	0.008561847
FAM222B	38.46331745	0.668395025	0.229574459	2.911452034	0.003597531	0.00856665
PPP4R1	29.30553051	-0.738274122	0.25361498	-2.911003607	0.003602699	0.008577426
SLC37A4	18.62290595	0.974434733	0.334758948	2.910854932	0.003604413	0.00857998
LOC105605100	6.843828064	-1.538643219	0.52861388	-2.910712863	0.003606052	0.008582353
TENT4B	73.54714621	0.646113304	0.222035601	2.909953633	0.003614824	0.008601698
LOC114113958	6.385733476	1.590697474	0.546797265	2.909117467	0.003624506	0.008623203
PHETA1	10.49098981	-1.226469142	0.42171388	-2.908296833	0.003634032	0.008644327
MVB12B	2.686716357	2.876444654	0.989243073	2.907722815	0.003640709	0.008658668
DPM3	13.61084583	-1.099005433	0.377972534	-2.907633051	0.003641754	0.008659613
B4GALT5	225.448759	0.450136994	0.154859079	2.906752354	0.003652022	0.008682484
ATRN	95.79259908	0.495619891	0.170563194	2.905784531	0.003663336	0.008707834
LOC121818589	2.220985137	3.148694683	1.08371258	2.905470272	0.003667017	0.008715033
CDC25B	35.52097766	-0.677152864	0.233084254	-2.905184935	0.003670362	0.008721432
NTAN1	17.52494703	-0.972896193	0.334998645	-2.904179486	0.00368217	0.008747935
NES	8.414113904	1.333957984	0.459443679	2.903420038	0.003691112	0.008767621
LOC101117683	13.71334995	-1.226206329	0.422354199	-2.903265393	0.003692935	0.008770394
GGA1	102.1465617	0.574782309	0.19799192	2.903059416	0.003695365	0.008774605
CEP135	22.10176742	0.918794361	0.316620162	2.901882035	0.003709282	0.008806086
STRBP	137.093548	-0.494929486	0.170623782	-2.900706332	0.003723226	0.008837621
SCN7A	1.270112135	3.682694944	1.269749446	2.900331994	0.003727676	0.008846612
OPTN	29.29652734	0.777778282	0.268225853	2.899714074	0.003735032	0.008861536
THAP9	11.03151132	1.16677048	0.402377331	2.899692378	0.00373529	0.008861536
RNASET2	24.1737016	-0.884784566	0.305139272	-2.899608955	0.003736285	0.008862321
GPR132	1.269346077	3.677404075	1.268689963	2.898583722	0.003748522	0.008889771
LOC121819248	6.872951438	-1.557233705	0.537297184	-2.898272595	0.003752243	0.008897016
MSRB3	7.808262323	1.782127279	0.614946065	2.898022086	0.003755241	0.00889939
QDPR	31.46987774	-0.739130932	0.255037645	-2.898124826	0.003754011	0.00889939
ZMYND11	202.8191582	0.415404341	0.143338883	2.898057617	0.003754816	0.00889939

TMEM107	29.81292914	-0.905045775	0.312412603	-2.896956677	0.003768018	0.008928085
LOC105609874	9.611004646	1.305141401	0.450607685	2.89640289	0.003774674	0.008942272
MGA	143.4289252	0.544906388	0.188199666	2.895363199	0.003787201	0.008968767
ZFP1	6.859187561	-1.630286495	0.563063457	-2.895386788	0.003786916	0.008968767
PRKCA	57.91415738	0.750441846	0.259241563	2.894758995	0.003794497	0.008984456
ANK3	143.2810322	-0.482729794	0.166808338	-2.893918859	0.003804665	0.009006934
ARID3B	8.901617087	-1.276080525	0.441081542	-2.893071699	0.003814942	0.009029664
CDT1	10.86657183	-1.239854048	0.428568774	-2.893010701	0.003815683	0.009029819
ZNF536	1.236297446	3.655073302	1.263518467	2.892773947	0.00381856	0.009035028
PKP3	142.2393283	-0.635146426	0.219581947	-2.892525708	0.003821579	0.009040571
LPGAT1	99.25931483	0.510733684	0.176596545	2.892093306	0.003826843	0.009051421
CNOT3	6.453885946	1.824735872	0.631195601	2.89091982	0.003841161	0.00908368
LOC101106416	18.76027261	1.088746801	0.376621958	2.890821363	0.003842365	0.009084919
TIPIN	45.65931937	-0.725143915	0.250882267	-2.890375333	0.003847821	0.009096211
SREBF1	20.15743339	0.974081519	0.337093533	2.889647602	0.003856739	0.009115681
WBP1L	18.30952434	0.942494924	0.326178142	2.889509758	0.00385843	0.009118066
PCF11	136.2940796	0.540450845	0.187043016	2.889446797	0.003859203	0.00911828
RASGRP1	2.155225961	3.197306515	1.106585711	2.889343756	0.003860468	0.009119656
IFT52	6.979239969	1.50010273	0.519231984	2.889079979	0.003863708	0.009125697
RAI2	1.642805786	3.398091468	1.176646713	2.887945406	0.003877672	0.00915706
DCAF1	48.64669747	0.615601207	0.21320303	2.887394273	0.003884471	0.009171497
LOC121816724	2.167730708	-3.432006623	1.189412412	-2.885463938	0.003908372	0.009226299
TTF1	122.0492698	0.603966101	0.209335841	2.885153817	0.003912225	0.009233762
TBC1D23	28.73851165	0.797643863	0.276650519	2.883218385	0.003936344	0.00928905
AMIGO2	4.074339306	2.437535312	0.845462751	2.883078301	0.003938095	0.009291542
SLC27A6	2.112424668	3.133684115	1.087007452	2.882854308	0.003940897	0.00929651
KIN	36.44010043	0.719886105	0.249809262	2.881743058	0.003954821	0.00932771
UBAP2L	52.63364263	0.597608329	0.207383249	2.881661527	0.003955844	0.009328478
LOC132657775	8.964740905	1.337523693	0.464379793	2.880236634	0.003973768	0.009369092
LTBR	54.47123262	-0.775704485	0.269349707	-2.879915835	0.003977813	0.00937651
SMIM1	2.618173013	2.881324735	1.000503084	2.879875916	0.003978317	0.00937651
PMPCB	30.5188057	0.805910162	0.279920864	2.879064285	0.00398857	0.009399018
LOC114110036	8.362414841	-1.776440593	0.617135581	-2.878525637	0.003995388	0.009413424
INSIG2	9.165511058	1.309088448	0.454788901	2.878452937	0.003996309	0.009413935
CEP57	22.47968756	-0.868354204	0.301739819	-2.877824369	0.00400428	0.00943105
RRM2	6.209097929	-1.516156561	0.526983624	-2.877046821	0.00401416	0.009452654
ZFAND2B	25.55400299	0.833794934	0.289817544	2.876965015	0.004015201	0.00945344
LOC132659679	19.73114009	-0.942825892	0.327768157	-2.876502411	0.004021091	0.00946564
LOC101103771	1.205105988	3.634165237	1.263593903	2.876054742	0.004026799	0.009477406
DCLK3	2.644527653	-3.313015743	1.151954089	-2.875996339	0.004027544	0.009477491
C22H10orf143	18.06214193	-0.905480311	0.314920488	-2.87526644	0.004036867	0.009497758
BUD31	89.35148416	0.46246494	0.160851219	2.875109955	0.004038868	0.009500794

LOC121816971	2.457992531	2.894368993	1.006775786	2.874889356	0.004041691	0.009503448
MTM1	9.975492523	1.347751114	0.46879077	2.874952325	0.004040885	0.009503448
ZNF286A	8.177578774	-1.411066434	0.490830452	-2.874855113	0.004042129	0.009503448
UBE3C	118.2337954	0.55334041	0.19249774	2.874529381	0.004046301	0.009511583
LOC132657771	9.695818795	1.237434619	0.430573534	2.873921694	0.004054095	0.009528227
LOC114115299	1.131000031	-3.874531695	1.348206558	-2.873841305	0.004055127	0.009528977
MRPS35	94.46927727	-0.471116648	0.163943773	-2.873647709	0.004057613	0.009533144
ZNF142	12.62186656	-1.143951862	0.398207579	-2.872752607	0.004069126	0.009558513
TMEFF2	1.199116189	3.62941121	1.263461852	2.872592634	0.004071187	0.009561674
LOC101102143	30.5083262	1.88347053	0.655690729	2.872498338	0.004072402	0.009562848
LOC132658307	22.85935958	0.951862264	0.33154788	2.870964713	0.004092212	0.009604484
SPRYD7	32.46250977	0.758824299	0.264310416	2.870958744	0.004092289	0.009604484
UBE2V2	74.97716066	-0.586488217	0.204279176	-2.871013232	0.004091584	0.009604484
PDZK1IP1	13.4563312	-1.246921298	0.434368962	-2.870650085	0.004096287	0.009610492
RHOB	15.3448969	1.029643198	0.358674319	2.870691166	0.004095755	0.009610492
LOC114111371	1.625599766	3.425319989	1.193272334	2.870526611	0.004097887	0.009612558
SLC38A1	54.52201118	-0.62640654	0.218238668	-2.870282085	0.004101058	0.009618308
LOC101120470	22.29777264	-0.897519348	0.312705413	-2.870175281	0.004102443	0.009619536
LOC121817001	1.700988772	3.361559273	1.171221645	2.870130762	0.004103021	0.009619536
IKBKE	19.69633342	0.909122842	0.316790056	2.869796018	0.004107367	0.009628036
ATP11B	37.48087945	0.690776468	0.24082834	2.868335465	0.004126378	0.009670188
SGK3	7.774952985	1.426833376	0.497448554	2.868303396	0.004126796	0.009670188
SDCBP2	2.156841278	3.219562337	1.12267574	2.867758001	0.004133916	0.009685175
HEXA	3.124985525	-2.517587083	0.878126245	-2.86699902	0.004143843	0.009706731
TWF2	28.56959867	0.825706292	0.288058708	2.866451418	0.004151019	0.009721836
DNAJC11	26.06780563	0.755021257	0.263433797	2.866075899	0.004155946	0.00973167
ZBTB12	14.57215953	-1.047833476	0.365637464	-2.86577164	0.004159942	0.009739321
NPTN	183.0373789	0.41898593	0.146226378	2.865323867	0.004165829	0.009751397
MRPL1	31.22199562	-0.701857445	0.24503577	-2.86430608	0.00417924	0.009781075
SLC34A2	3.787517214	4.620753123	1.613447603	2.86390033	0.004184597	0.009791897
DYNLT2B	23.64632982	-0.787639081	0.275110691	-2.862989726	0.004196642	0.009818364
ITPKB	6.15378941	1.606692407	0.561263164	2.862636479	0.004201323	0.009827595
ALS2CL	48.2291182	0.77914364	0.272193129	2.862466234	0.00420358	0.009831156
RPS25	458.5100136	-0.466587779	0.16303983	-2.861802408	0.004212394	0.009850046
TSPAN33	2.970506874	2.462242122	0.860926127	2.859992334	0.004236512	0.00990471
GLOD4	5.26851472	-1.667203251	0.582990241	-2.8597447	0.004239822	0.009910713
LOC105614854	13.58646555	-1.55183631	0.542713556	-2.859402152	0.004244403	0.009919688
SHROOM3	47.21049236	0.637662994	0.223104561	2.858135177	0.004261388	0.009957642
LOC101102694	26.37798364	-1.009535116	0.353255774	-2.857802168	0.004265863	0.009964614
SBNO2	26.36097901	0.800954821	0.280264647	2.857851786	0.004265196	0.009964614
C9orf72	13.15185675	1.053236185	0.368590304	2.857471217	0.004270314	0.009973268
HS3ST3A1	1.211098468	3.595881832	1.258517562	2.857236118	0.004273478	0.009978915

GLIS2	12.02223361	-1.155026048	0.404402368	-2.856130772	0.004288385	0.010011974
HBP1	3.581572557	-2.231092377	0.781354484	-2.855416361	0.004298044	0.010031021
WASL	105.5587626	0.500739841	0.175364389	2.855424889	0.004297929	0.010031021
PIGB	9.540235835	-1.357381622	0.475403563	-2.855219709	0.004300707	0.010035407
RPP21	5.19025902	-1.722099633	0.603152056	-2.855166648	0.004301425	0.010035407
BLCAP	52.73099632	0.579874788	0.203149228	2.854427723	0.004311444	0.010057026
CCDC7	97.26979312	0.648844904	0.22733455	2.854141186	0.004315335	0.010064346
LOC121819617	1.76552765	3.476329713	1.218128128	2.853829275	0.004319574	0.010072474
WT1	1.301905206	3.766897952	1.320248432	2.853173584	0.004328497	0.010091521
FAR2	2.426689618	-3.043334889	1.066676847	-2.85309923	0.00432951	0.010092123
ARHGAP28	6.125459796	1.566057929	0.549155011	2.851759336	0.0043478	0.010132991
BEND7	88.45834524	-0.650633137	0.22821024	-2.851025171	0.004357852	0.010154646
N4BP2L2	50.25311102	0.713267285	0.250200537	2.850782395	0.00436118	0.010160631
TEN1	23.65992554	0.793285551	0.278456282	2.848869288	0.00438749	0.010220145
E2F2	7.971064434	-1.33802272	0.469766717	-2.848270583	0.004395753	0.010237609
ECE1	60.05097324	-0.717613919	0.252097357	-2.846574547	0.004419238	0.010290512
ABCA10	1.348894203	3.774521672	1.326087552	2.846359327	0.004422226	0.010295676
PDIA5	6.692169942	1.718625031	0.603817796	2.846264291	0.004423546	0.010296956
LOC114110273	2.922222598	-2.771292297	0.973689488	-2.846176661	0.004424764	0.010297997
KLHL17	11.97766537	1.237521082	0.435021401	2.844736095	0.004444823	0.010342881
TXN	64.10353406	0.990242715	0.348176733	2.844080666	0.004453977	0.010362377
FRMD4B	15.24846375	1.057362611	0.372105971	2.841563137	0.004489296	0.010439912
LOC114111327	2.721000548	2.83239398	0.996781928	2.841538255	0.004489647	0.010439912
TRAF5	2.784664221	2.605824195	0.917028234	2.841596472	0.004488827	0.010439912
TLR2	3.765845879	2.516647528	0.885778731	2.841169514	0.004494841	0.010450174
USP7	94.8223637	-0.50721463	0.178570336	-2.840419317	0.004505427	0.010472963
TRDMT1	9.850487396	1.240310271	0.436690851	2.840247894	0.004507849	0.010476771
LOC106990150	1.583105625	-3.481807287	1.226085591	-2.839775063	0.004514536	0.010490487
BHLHE41	13.4037152	-1.029378645	0.362514887	-2.839548616	0.004517741	0.010496111
PHLDA2	1.120719763	-3.862528877	1.361040512	-2.837923518	0.004540806	0.010547865
UTP4	11.99029417	-1.195279628	0.421194612	-2.83783219	0.004542106	0.01054905
KCTD6	14.87664319	1.005265511	0.354286192	2.83743915	0.004547701	0.010560211
MTUS1	152.2362075	0.425566861	0.150075688	2.835681555	0.004572802	0.010616652
ATOSA	16.17607604	1.065327322	0.37603158	2.833079396	0.004610193	0.010701606
CFAP97	69.48345474	-0.550230932	0.194268464	-2.832322446	0.004621122	0.010725112
LOC121819401	5.691724541	1.692692475	0.597674296	2.832131959	0.004623876	0.010729641
HECA	23.46261784	0.822840923	0.290592483	2.831597413	0.004631612	0.010744224
SLC48A1	5.477627833	-1.650635731	0.58293669	-2.831586618	0.004631769	0.010744224
EREG	5.326894239	-1.769730341	0.625104645	-2.831094532	0.004638901	0.010755815
PPP4R3A	113.3606357	-0.475858354	0.168083962	-2.831075311	0.00463918	0.010755815
TMED1	17.74996671	-0.947834123	0.334790067	-2.831129761	0.00463839	0.010755815
ATG10	22.76929936	-0.751737293	0.265580687	-2.830542015	0.004646921	0.010771894

PEX2	22.57214272	1.098852699	0.388296114	2.829934836	0.004655749	0.010790486
GRB7	4.728517443	-1.777209113	0.628037849	-2.829780268	0.004657998	0.010793829
LOC105607931	5.555097384	1.80603788	0.638266508	2.829598387	0.004660647	0.010798094
SGPP2	7.87027382	-1.455791883	0.514511609	-2.829463624	0.00466261	0.01080077
STYK1	3.67778327	2.285662432	0.80786528	2.829261869	0.004665551	0.01080571
RTCA	84.15605171	-0.581112582	0.205472293	-2.828179773	0.004681351	0.010840426
XPC	119.9809965	0.70984273	0.251070607	2.827263362	0.00469477	0.010869616
ASS1	1.71847403	3.372793011	1.193309345	2.826419674	0.004707155	0.010896403
RNF130	3.367674253	2.38656907	0.844604596	2.825664319	0.004718268	0.010920237
MRGPRF	1.226796275	3.651386773	1.292559267	2.824927929	0.004729125	0.010943471
L2HGDH	16.13062303	1.134601639	0.401704251	2.824470084	0.004735887	0.010957221
LOC121820472	2.649888394	-2.664954902	0.943663152	-2.824053154	0.004742052	0.010969586
ATF7IP2	15.46481867	0.97305582	0.344587382	2.82382894	0.00474537	0.010975363
FCAMR	2.221224048	3.075105205	1.089018539	2.823739996	0.004746687	0.010976509
PLEKHA2	20.12373007	0.902865008	0.319790849	2.823298449	0.00475323	0.010989738
LOC101121639	11.60634613	1.161419968	0.411566677	2.821948504	0.004773284	0.011034195
NUP210L	4.604421839	1.891197395	0.670594276	2.820181239	0.004799653	0.011093233
CCAR1	287.3204637	0.49764196	0.176463273	2.820088005	0.004801048	0.011094538
GORASP1	9.340097426	1.247019994	0.442234174	2.819818247	0.004805086	0.011101949
ELL3	2.281988965	3.101366603	1.100378354	2.818454755	0.004825541	0.011147284
ABCC11	7.486608035	1.491526053	0.529301691	2.817912879	0.004833692	0.011164184
ADGRL2	32.98687882	0.733413534	0.26029863	2.817585069	0.00483863	0.011173656
SLC26A7	3.34965187	-2.153026189	0.764657018	-2.815675706	0.004867477	0.011238331
NUSAP1	84.23123569	-0.786735927	0.279449729	-2.815303959	0.004873112	0.011249398
CLDN12	32.50248644	0.836453712	0.297133639	2.815075782	0.004876573	0.011255444
B3GNT2	87.76047636	0.563020614	0.200012954	2.814920755	0.004878926	0.011258931
HDC	1.221365698	3.606277895	1.281448326	2.814220302	0.004889571	0.011281547
ARID2	36.82769378	0.707256738	0.251421791	2.813028802	0.004907726	0.011319527
CMSS1	5.996288948	-1.528146334	0.543232905	-2.813059223	0.004907261	0.011319527
SIRT3	7.368532902	-1.400704685	0.49796161	-2.812876851	0.004910045	0.011322923
LOC114113418	4.66721508	1.975431952	0.702671034	2.811318322	0.004933895	0.011375959
CDH4	1.531286555	-3.578315941	1.273077194	-2.810761168	0.004942446	0.01139371
DHX38	19.5161914	0.947875356	0.337321545	2.810005381	0.004954067	0.011418531
CPSF6	110.6512152	-0.452598992	0.161076139	-2.809845056	0.004956536	0.011422251
LOC101102642	4.709312288	1.842578199	0.655932741	2.809096244	0.004968079	0.011446879
LYPD5	7.238644136	-1.502701338	0.535012543	-2.8087217	0.004973862	0.011458229
NOL8	83.30044826	-0.591573422	0.21070893	-2.807538449	0.004992172	0.011498427
HAPLN3	2.870916732	2.610429311	0.929875309	2.807289629	0.00499603	0.011504406
LOC105604976	14.01050393	-1.044668746	0.372131095	-2.807260024	0.004996489	0.011504406
PNPLA6	2.799555947	2.58458934	0.920746763	2.807057754	0.004999628	0.011508568
SPRN	1.320999593	3.719364368	1.325016471	2.807032554	0.005000019	0.011508568
SLC22A15	18.80020336	-0.919743121	0.327688431	-2.806761041	0.005004235	0.011516288

TRAF7	22.6904506	0.791368397	0.282003485	2.806236231	0.005012393	0.011533077
MRPL55	84.02517146	-0.553084532	0.197096178	-2.806165687	0.005013491	0.011533617
DBF4	63.1818292	-0.729557951	0.260009785	-2.805886522	0.005017836	0.011539641
KIF5B	369.5504645	0.362431486	0.129167563	2.805901697	0.0050176	0.011539641
MAPKAPK2	29.27973813	-0.729557802	0.260062008	-2.805322502	0.005026626	0.011557867
RRM2B	9.192207134	1.322122947	0.471401331	2.804665283	0.005036886	0.011577924
UBE2M	16.44158486	0.938498658	0.33462205	2.804652768	0.005037081	0.011577924
CLK1	132.7074752	0.527371605	0.18807168	2.80409898	0.005045741	0.011595835
A4GNT	12.00222724	3.169612443	1.13051642	2.803685454	0.005052217	0.011606725
ZNF250	7.158581484	-1.566601297	0.558754304	-2.803739114	0.005051376	0.011606725
GTPBP4	127.9860453	0.562908926	0.200822265	2.803020506	0.005062645	0.011628684
LOC114116894	6.290276437	-1.535674361	0.547901013	-2.802831759	0.005065609	0.011633492
EFNA3	3.080661606	-2.471598486	0.881912992	-2.802542324	0.005070156	0.011641936
ARL4D	3.87345879	-2.186523608	0.78035512	-2.801959712	0.005079322	0.011660978
NAPG	21.69516594	-0.818244859	0.292064103	-2.801593384	0.005085092	0.011672221
SEPTIN2	33.33936189	-1.081461381	0.386100991	-2.800980592	0.005094758	0.0116924
CPD	243.8493444	0.684311512	0.244322566	2.800852677	0.005096778	0.011695028
RAD51	12.73938995	-1.08931782	0.389003247	-2.800279507	0.005105838	0.011713804
TNS3	23.06071783	0.771516925	0.275543925	2.799977991	0.005110609	0.011722739
LOC132659150	29.6894863	0.762394434	0.272314806	2.799680437	0.005115322	0.011731536
CCZ1	101.6883233	-0.518591451	0.185237087	-2.799609194	0.005116451	0.011732112
RABAC1	4.771871761	-1.999699893	0.714638357	-2.798198382	0.005138854	0.011781462
RPL32	1058.452974	-0.435683844	0.155710462	-2.798038352	0.005141401	0.011785279
ADAMTS15	9.073628658	-1.308716424	0.467759898	-2.797838011	0.005144591	0.011790569
METTL25	13.89404212	1.007096577	0.360074715	2.796910017	0.00515939	0.011822185
WDR91	10.30702309	-1.283430758	0.45888238	-2.796862143	0.005160155	0.011822185
LOC132659271	2.695324524	2.65115986	0.948214401	2.795949795	0.005174744	0.011853578
FBLN7	1.190564009	3.579454766	1.280401042	2.795573143	0.005180778	0.011865366
TRPM7	79.81220431	-0.704551967	0.25205917	-2.795184828	0.005187005	0.011876176
ZPR1	76.08055442	0.484686838	0.173401683	2.795168023	0.005187275	0.011876176
SVOPL	2.917632679	-2.478011897	0.886563932	-2.795074115	0.005188782	0.011877592
LOC132658189	3.557645092	-2.203133537	0.788355812	-2.794592876	0.005196511	0.011893248
CIZ1	15.63042984	-1.006063674	0.360053336	-2.794207339	0.005202711	0.011904486
LOC105607736	4.867174766	-1.76837798	0.632879777	-2.794176784	0.005203203	0.011904486
TRPC4AP	6.218720983	-1.602248693	0.573624716	-2.793200238	0.005218937	0.011938442
AP3M2	2.954912318	-2.465270897	0.882784563	-2.792607619	0.005228507	0.011958287
PRX	3.950511246	-2.078953764	0.744485482	-2.792470523	0.005230723	0.011961308
LOC132659910	3.271039786	-2.281572681	0.817211466	-2.791899988	0.005239955	0.011980369
SRD5A1	14.55558385	-1.126661206	0.40356577	-2.791766025	0.005242125	0.011981231
SYNGR2	128.8806915	0.457183001	0.163759717	2.791791582	0.005241711	0.011981231
SHC3	3.752078837	-2.072923667	0.742652829	-2.791241863	0.005250622	0.011998601
CDC42EP4	22.54786837	0.808716029	0.289795875	2.790640236	0.005260391	0.012018869

LOC132659237	1.081983629	-3.80039807	1.362092534	-2.790117393	0.005268893	0.012036238
CHMP4A	37.12820598	-0.839153091	0.300792438	-2.789807804	0.005273934	0.012045694
GPR15	1.17812224	3.573776482	1.281132035	2.789545795	0.005278203	0.012053385
MAK16	78.36243784	-0.497010744	0.17828495	-2.787732464	0.005307836	0.012118984
BICRAL	116.9785649	0.578830544	0.207669122	2.787273033	0.005315367	0.012134108
PLEKHG6	4.251482061	1.862150822	0.668298839	2.786404395	0.005329634	0.012164598
PGLYRP4	1.297134766	3.703123827	1.329289435	2.785791965	0.005339713	0.012185522
PRKAR1B	28.09251455	0.775548565	0.27840099	2.785724885	0.005340818	0.012185963
ANKRD45	10.36065791	-1.106236281	0.397134986	-2.78554225	0.005343828	0.012190749
ASAH1	46.97129256	-0.776428638	0.278858081	-2.784314643	0.005364097	0.012234901
KRR1	41.01477538	0.839804726	0.301719413	2.783396396	0.005379304	0.012267493
ARHGAP42	17.65836634	0.898056755	0.322735144	2.782643203	0.005391806	0.012293907
RAB7B	6.386904404	1.494248725	0.537174891	2.781680141	0.005407831	0.012328342
LOC106991442	2.020817578	-3.31803726	1.192856516	-2.781589584	0.00540934	0.012329679
KLHL9	2.540432827	2.596249559	0.93353925	2.781082382	0.005417799	0.012346854
SIGLEC1	1.552183876	3.312087898	1.191067377	2.780772911	0.005422966	0.012356522
FADS3	2.970899043	2.425723138	0.872429476	2.780423181	0.00542881	0.012367731
GCFC2	14.65821559	1.013831419	0.364658307	2.780223021	0.005432158	0.012373248
FGFR1OP2	23.27657016	0.817933662	0.294221917	2.779988892	0.005436076	0.012380063
DAP3	61.18058466	-0.552699461	0.19882418	-2.779840265	0.005438564	0.01238362
MOGAT1	12.92464754	1.050867789	0.378163726	2.778869881	0.005454837	0.012418558
LOC132658243	23.25395372	0.797163106	0.286877787	2.778755073	0.005456765	0.012420832
UCHL3	15.5335216	-0.954862648	0.343678662	-2.778358835	0.005463425	0.012433873
TMEM9B	39.0375244	0.799653117	0.28787048	2.777822576	0.005472449	0.01245229
SLC6A6	9.810211975	-1.372826716	0.49432786	-2.777158291	0.005483647	0.012475646
ACAD11	17.19191214	0.867078363	0.312229617	2.77705354	0.005485414	0.012477544
LOC114113944	6.138304895	-1.4597332	0.525696418	-2.776760789	0.005490357	0.012486662
MRPL58	39.46594584	-0.738843108	0.26612461	-2.776305082	0.005498059	0.012502051
NCAPH2	8.099066864	-1.395780021	0.502776076	-2.776146453	0.005500742	0.012506025
TSPAN11	1.156359551	3.554595651	1.280512551	2.77591629	0.005504638	0.012512753
WNK3	1.561225243	3.353259104	1.208307026	2.775171402	0.005517262	0.012539318
BEND5	10.3025612	1.158926445	0.417614746	2.775109014	0.005518321	0.012539591
IRF8	23.27031145	-0.856626144	0.308717371	-2.774790876	0.005523721	0.01254973
LOC132657528	2.700933739	2.659467066	0.958744901	2.773904783	0.005538789	0.012581824
PSD4	9.363781356	1.174478758	0.423423654	2.773767473	0.005541127	0.012584996
LOC101115890	3.639275657	2.436061716	0.878464918	2.773089361	0.005552687	0.012609109
SLC17A9	10.66818197	1.538153945	0.554719315	2.772850886	0.005556758	0.012616209
FGFR1	65.47824026	-0.56618677	0.204224592	-2.772373122	0.005564921	0.012632597
USP35	3.988457459	-1.84324028	0.664997945	-2.771798461	0.005574754	0.01265277
MED10	13.9481174	-1.065685	0.384662719	-2.77044004	0.005598061	0.012703511
POLL	15.87551322	0.926734212	0.334548291	2.770105947	0.005603806	0.01271439
CXCL11	2.882182666	2.536845521	0.916058387	2.76930549	0.005617594	0.012743509

PLK2	19.09172397	-0.948396123	0.342477454	-2.769222066	0.005619032	0.01274461
EAPP	58.72301577	-0.529128093	0.191088015	-2.769028158	0.005622378	0.012750034
UROS	3.775640055	2.067581564	0.746903237	2.768205385	0.005636592	0.0127801
LOC132660063	4.524078743	1.839011378	0.664411856	2.767878632	0.005642246	0.01279075
BCKDHA	21.05064835	-0.84262374	0.304461137	-2.767590462	0.005647237	0.012799893
JUN	156.6681216	-0.443261319	0.160167292	-2.76748962	0.005648984	0.012801683
ZNF10	7.569666776	-1.366253169	0.49375326	-2.767076758	0.005656144	0.012815734
AGFG2	5.788098333	1.579079033	0.570694135	2.76694456	0.005658438	0.012818759
PPFIA1	105.3651702	0.602193692	0.217652104	2.766771744	0.005661438	0.012823382
ZDHHC8	5.135525437	-1.665544145	0.602008203	-2.766646928	0.005663606	0.012826119
RGL2	16.11725593	-0.920921464	0.332891446	-2.766431743	0.005667345	0.012832412
FAM131B	5.301580513	1.676073165	0.605930618	2.766113999	0.00567287	0.012842747
LOC132657211	8.062017295	-1.258243098	0.454994282	-2.765404196	0.00568523	0.01286855
ACAP2	80.76299555	0.626380573	0.226530727	2.765102028	0.005690499	0.012878296
LOC101112543	17.29734409	1.044877265	0.378016003	2.764108546	0.005707855	0.012915386
ATP10D	2.358045008	2.796534477	1.012199623	2.762829004	0.005730278	0.012958113
CHAMP1	39.7036499	-0.807655667	0.29231399	-2.762973021	0.00572775	0.012958113
NUP88	78.21489311	0.518602602	0.187706547	2.762837049	0.005730137	0.012958113
POLR2G	80.07372839	-0.812387115	0.294043813	-2.762809761	0.005730616	0.012958113
KBTBD6	10.09761404	-1.162141168	0.420682259	-2.762515277	0.005735788	0.012967615
TNC	1.297700177	3.759757388	1.361569372	2.761341042	0.005756453	0.013012135
TMEM59L	2.356558791	2.754692735	0.997699938	2.761043305	0.005761704	0.013021801
LOC114116876	2.926058189	2.481109171	0.898686017	2.760818711	0.005765667	0.013028556
C1H3orf33	29.61636773	-0.674943604	0.244480332	-2.760727615	0.005767275	0.013029987
ACTR10	48.3926939	-0.650683828	0.235698024	-2.760667309	0.00576834	0.013030191
DEXI	14.39963139	-1.054592688	0.382167382	-2.759504703	0.005788905	0.013074436
STIM2	36.02570136	-0.671046485	0.243199488	-2.759243005	0.005793544	0.0130827
NIPA1	3.888640426	2.034920955	0.737793299	2.758117969	0.005813521	0.013125596
NDUFB5	25.23022909	-0.774931119	0.280974175	-2.758015465	0.005815345	0.013127495
PTEN	66.04502833	0.494322893	0.179254618	2.757657778	0.005821711	0.013139647
CADM3	8.372516819	1.503156251	0.545378105	2.756172714	0.005848211	0.013195001
CDK20	4.274587797	-1.865670111	0.676904441	-2.756179452	0.00584809	0.013195001
CD46	132.111879	-0.553641944	0.200933965	-2.755342753	0.005863068	0.013224058
UNC5B	5.459388393	-1.556365573	0.56484998	-2.755360941	0.005862742	0.013224058
CENPQ	22.59387591	-0.816075935	0.296217741	-2.75498669	0.005869452	0.013236224
TOMM20	138.315323	-0.599636491	0.217680743	-2.754660252	0.005875311	0.013247201
EMC6	57.78058168	-0.602586232	0.218788843	-2.754190858	0.005883745	0.013263979
KIF26B	1.677884906	3.427029211	1.24438919	2.753985038	0.005887446	0.013270085
CCDC171	7.504165984	1.373359372	0.498792798	2.753366481	0.005898583	0.013292945
NCK1	62.57283094	-0.537256815	0.195204637	-2.752274858	0.005918283	0.013335092
CNTRL	83.78034668	0.619295531	0.225040779	2.751925819	0.005924594	0.013347063
RPS26	34.08573411	-1.204239986	0.437879843	-2.750160816	0.005956602	0.01341691

NFKBIZ	71.07404459	0.50593262	0.183975639	2.749997888	0.005959565	0.013421322
SGSM2	12.17299536	1.183070284	0.430256327	2.749687127	0.005965219	0.013431792
CEP41	19.62047598	-0.810760941	0.294900102	-2.749273175	0.005972759	0.013446503
BRIX1	37.25711495	0.745886724	0.271311434	2.74919016	0.005974272	0.013447645
CD7	1.212326698	3.600315448	1.309704997	2.748951449	0.005978624	0.013455176
SNRPA	68.31303436	-0.540311099	0.19658811	-2.74844241	0.005987915	0.013473818
EPOP	11.58324523	-1.086845549	0.395501181	-2.748020996	0.005995617	0.013488877
SCN9A	1.944010009	3.075819512	1.119958402	2.746369423	0.006025887	0.013554696
LOC132660129	5.241841934	1.908356363	0.695100941	2.745437749	0.006043023	0.013590955
EMD	10.19601968	-1.192542013	0.434425421	-2.745101817	0.006049213	0.013602587
CTNNB1	274.043671	-0.324777502	0.118369406	-2.743762201	0.006073952	0.01365592
CPPED1	13.26471859	-0.99397447	0.362283939	-2.743633829	0.006076327	0.013658963
RAB39A	1.259736302	3.675017973	1.339943936	2.742665477	0.006094274	0.01369585
TNFRSF11B	14.9645141	1.035896343	0.377700742	2.742637828	0.006094787	0.01369585
QTRT2	5.790107467	-1.606144194	0.585751477	-2.742023291	0.006106201	0.013716888
RNF122	2.243648473	2.754690057	1.004608854	2.742052338	0.006105661	0.013716888
LOC132658745	4.057605576	1.973231695	0.71980343	2.741348002	0.006118766	0.013742805
SDK2	1.574435753	3.328221208	1.214191085	2.741101668	0.006123356	0.013750802
ZFYVE27	33.67263577	0.801676604	0.29248832	2.740884162	0.006127411	0.013757596
IMPA1	14.10732058	-0.944959878	0.34483579	-2.740318451	0.006137968	0.013778986
PKLR	4.417660365	2.055636524	0.75037765	2.739469285	0.006153846	0.013812311
DNAJA2	118.859057	-0.450173125	0.16439264	-2.73840195	0.006173857	0.013854897
LOC121816456	28.65491986	-0.702969659	0.256726642	-2.738202994	0.006177593	0.013860955
CMYA5	3.155659963	2.297338253	0.839030073	2.738088093	0.006179752	0.013863472
NBL1	5.867380291	1.637442611	0.598403385	2.736352522	0.006212443	0.013934472
LOC121819673	1.309855773	3.745393777	1.368875184	2.736110509	0.006217014	0.013942385
RIOX2	18.13874305	-0.930902064	0.340291262	-2.735603781	0.006226595	0.013961528
DAAM1	120.7148842	0.505504448	0.184817448	2.735155432	0.006235083	0.013973527
MAZ	16.39433126	-0.95738977	0.350023967	-2.735212041	0.00623401	0.013973527
NUP50	79.97196555	-0.482728709	0.176490102	-2.73516023	0.006234992	0.013973527
STYXL1	2.562987462	2.989464801	1.093034263	2.735014721	0.006237749	0.013977158
CEP63	23.88488742	-0.805260481	0.294465963	-2.734647062	0.006244719	0.013990432
UNC93B1	25.80492889	0.969461997	0.354523331	2.734550624	0.006246549	0.013992186
CHID1	118.8669054	-0.618943141	0.226378033	-2.734113076	0.006254856	0.014008446
JAML	1.744023879	3.457809506	1.265223168	2.732964107	0.006276717	0.014055051
DNAJC1	121.6316308	0.543147532	0.198794235	2.732209666	0.006291109	0.01408256
TSPAN15	23.31656828	0.783207415	0.286652005	2.732258636	0.006290174	0.01408256
HOXB7	1.516194648	3.292562025	1.20519232	2.731980589	0.006295485	0.014089995
LOC114115362	5.044430462	-1.716763398	0.628517756	-2.731447729	0.006305674	0.014110437
TSPO	8.026045078	-1.335896473	0.489120311	-2.731222654	0.006309983	0.014117715
TCF20	43.10044238	-0.709805834	0.259959673	-2.730445937	0.006324871	0.014148656
GFER	29.45540264	-0.752581023	0.275791804	-2.728801259	0.0063565	0.014217031

RERE	397.4566471	-0.342663503	0.125590761	-2.728413309	0.006363982	0.014231383
PACC1	3.990725615	-1.839036474	0.674299224	-2.727329956	0.006384916	0.014275809
LMBRD2	41.74455517	0.585984674	0.214937989	2.726296432	0.006404945	0.014318196
LOC132659608	4.36682695	1.992689699	0.731171006	2.725340149	0.006423527	0.014357335
SEPHS2	49.80817892	-0.885804942	0.325038146	-2.725233798	0.006425597	0.01435956
SERBP1	933.606801	-0.334446624	0.12274312	-2.724768797	0.006434653	0.014377394
LOC132659423	1.547814161	3.337164525	1.224834731	2.724583522	0.006438264	0.014383059
TMEM82	2.486028553	2.921758299	1.072652303	2.723863354	0.00645232	0.01441205
BRF2	3.637251457	-2.169288069	0.796569852	-2.72328668	0.006463594	0.014434821
C15H11orf52	11.94339501	-1.155744551	0.424500315	-2.722599982	0.006477043	0.014462439
TTBK2	9.642630612	1.165620775	0.428314806	2.721411353	0.006500382	0.014512127
MCF2L	5.186476993	-1.629501599	0.598857959	-2.721015183	0.006508177	0.014527104
APOBR	2.67533968	2.527295909	0.928910817	2.72070888	0.00651421	0.014538142
PSMC3IP	6.175172179	-1.498897332	0.551092019	-2.719867609	0.006530806	0.014572746
ZNF516	8.656267778	1.333289125	0.490293679	2.719368372	0.006540672	0.014592326
EGFR	16.78423112	0.871409203	0.320472978	2.719134727	0.006545294	0.014600201
LOC101106919	4.294282691	1.989182243	0.731759273	2.718356046	0.00656072	0.014632168
LOC132657304	36.02873055	-0.753107168	0.277080409	-2.718009443	0.006567596	0.014645061
LOC121818983	5.635191875	-1.704288842	0.627088118	-2.717782066	0.006572111	0.014652683
ZNF202	9.03535495	-1.219273393	0.448654856	-2.71761996	0.006575331	0.014657418
CLEC5A	1.16491173	3.60089652	1.325094577	2.717463782	0.006578435	0.014659448
RND3	18.13818111	1.236140788	0.454886072	2.717473371	0.006578245	0.014659448
MTMR4	24.66955068	-0.892982153	0.328628028	-2.717303691	0.006581619	0.014664096
TLCD4	40.74730388	-0.622368582	0.229081037	-2.716805325	0.006591537	0.014683746
FBXL18	10.35268855	-1.182171873	0.435332823	-2.715558786	0.006616403	0.014736684
CCNB2	18.31283223	-0.881075822	0.32449464	-2.715224573	0.006623084	0.014749107
RNF38	23.64833872	0.81632977	0.300661325	2.715113987	0.006625296	0.014751576
MDP1	6.575785404	-1.47265271	0.542593957	-2.714097143	0.006645668	0.014794469
NUS1	103.2568567	0.500908084	0.184660895	2.712583433	0.006676097	0.014859736
DUSP14	4.480604368	-1.847879307	0.68129367	-2.712309518	0.006681617	0.014869546
FGF14	2.282729292	2.847008696	1.04996552	2.711525895	0.006697431	0.014902258
HELB	17.75736397	0.883324196	0.325880571	2.710576438	0.006716637	0.014942504
MCM5	14.89218149	-1.02901116	0.379641614	-2.710480419	0.006718582	0.014944344
PTGDR	1.52674781	3.292416503	1.214941918	2.709937367	0.006729591	0.014966343
SPPL2B	27.60140132	-0.784632131	0.289600154	-2.709363648	0.006741241	0.014989756
NFKBIE	6.823221164	1.400652766	0.51714845	2.708415286	0.006760537	0.015030162
ADI1	131.4465276	0.418879246	0.154725508	2.707241043	0.006784498	0.015080924
CEP112	18.77193699	0.899227978	0.332267361	2.706338576	0.006802965	0.015119459
SAR1B	57.53652489	0.606210801	0.2240241	2.706007079	0.006809759	0.015132044
LOC132658200	15.81798655	-0.925053788	0.341870235	-2.705862322	0.006812728	0.015136125
LOC132659922	2.472906955	2.825429732	1.044289087	2.705601129	0.006818089	0.015145516
LOC121816891	4.506785431	-1.790929173	0.662134842	-2.704780145	0.006834961	0.015180473

S1PR5	1.852545502	2.995073394	1.107432234	2.704520695	0.006840301	0.015189809
ADAT2	2.653408221	-2.691620991	0.995417836	-2.704011214	0.006850798	0.015210592
ABCC1	4.30359228	-1.933673451	0.715261209	-2.703450747	0.006862362	0.015233736
TNFSF13	18.51197122	0.850199397	0.314560563	2.702816236	0.006875475	0.015260311
PIP5K1C	25.83320791	-0.821845665	0.304142874	-2.702169721	0.006888859	0.015287479
C21H11orf24	18.1764282	0.857096686	0.317226753	2.701842384	0.006895644	0.015299997
POLD3	28.43541632	0.785518614	0.290815924	2.701085286	0.006911361	0.015332325
NKD1	8.966212685	-1.160308621	0.429628614	-2.700724728	0.006918858	0.015346408
SCYL3	17.52337701	-0.925553804	0.342786671	-2.700086916	0.006932136	0.015373309
NCF4	2.468228575	2.709651379	1.003590319	2.699957669	0.00693483	0.015376731
NFATC2IP	10.27686993	-1.092469948	0.404715683	-2.699351653	0.006947472	0.015402208
MUL1	17.30369633	0.857701647	0.3177571	2.699236767	0.006949871	0.015404972
ANKRD28	40.4346808	0.599605393	0.222167752	2.698885811	0.006957204	0.015416113
RAD51AP1	8.049847464	-1.27044013	0.470721547	-2.698920706	0.006956475	0.015416113
SNX4	58.05365243	0.636340566	0.235864574	2.6979065	0.006977704	0.015458973
BST-2B	1.963005848	3.051442434	1.131300408	2.697287488	0.006990689	0.015482609
LOC101119114	1.899550065	2.98570907	1.106920191	2.697311959	0.006990175	0.015482609
DAB1	24.83835931	0.763936722	0.283304327	2.696523311	0.007006749	0.015510469
MAPK7	7.823114309	1.262243058	0.46809408	2.696558475	0.00700601	0.015510469
SF1	161.9107265	-0.453166522	0.168055637	-2.696526751	0.007006677	0.015510469
DFFB	3.937073589	-2.178479948	0.80811784	-2.695745397	0.007023132	0.015544161
LOC101105614	11.06567564	-1.251614794	0.464517931	-2.694438062	0.007050743	0.015602687
SRPX	5.660697504	1.801316422	0.668560496	2.694320757	0.007053225	0.015605596
XAF1	4.055367364	1.977666005	0.734106657	2.693976395	0.007060516	0.015619143
KEF53_p13	49.0228789	0.790042978	0.293300219	2.693632414	0.007067806	0.015632683
DDX17	385.8041654	-0.413782397	0.153620538	-2.693535659	0.007069858	0.015634622
TAB2	71.59687872	0.546287547	0.202818433	2.693480755	0.007071023	0.015634622
EVPL	1.940424475	2.993415092	1.111379737	2.693422412	0.00707226	0.015634772
F8	4.730175064	1.704048987	0.632695662	2.693315428	0.00707453	0.015637204
DECR1	28.84428732	0.674052807	0.250290963	2.693076881	0.007079594	0.01564581
EEF1D	266.0873976	-0.56982583	0.211657544	-2.692206573	0.007098097	0.015684106
LOC132659970	3.151807301	2.417266784	0.897940599	2.692011907	0.007102241	0.01569067
EIF4G1	215.3310518	-0.398456065	0.148027929	-2.691762755	0.007107549	0.015699801
GFRA2	1.102885064	3.511665635	1.30477503	2.691395493	0.007115379	0.015709308
LOC101114275	21.83826428	-1.204087115	0.447374563	-2.691451891	0.007114176	0.015709308
LOC132658101	1.132388803	3.493553863	1.298038943	2.69140913	0.007115088	0.015709308
RPL5	260.9353396	-0.515250389	0.191480402	-2.690877945	0.007126426	0.0157311
TMEM161A	17.65408765	-0.917098599	0.340964741	-2.689716822	0.007151267	0.015783329
GNG7	1.479226921	3.263240113	1.213268668	2.689626955	0.007153193	0.015784973
LOC101102503	1.129303401	3.49075288	1.297979475	2.689374482	0.007158606	0.01579431
POGLUT1	24.95192272	0.738168551	0.274515688	2.688984941	0.007166965	0.015810143
LOC105604827	16.83728305	1.347772711	0.501252434	2.688810308	0.007170715	0.015815806

LOC114108786	2.419002724	2.498927398	0.929535471	2.688361527	0.007180361	0.015834467
MFSD6	19.26132842	0.904324363	0.336409577	2.688164737	0.007184594	0.015841189
LOC105611550	42.75233171	0.699678847	0.260315806	2.687807777	0.007192279	0.015855517
LOC114118624	3.869836324	1.981248068	0.73721668	2.687470483	0.007199547	0.015868921
PLAGL2	18.77976327	-0.90772311	0.33779892	-2.687169955	0.007206028	0.015880588
MAD2L1	17.87238578	-0.885518433	0.329585646	-2.686762737	0.007214818	0.015897339
CTH	16.11162624	-0.941089123	0.350277597	-2.686695156	0.007216278	0.015897935
LOC101118053	5.296290697	-1.575021279	0.586379762	-2.686008932	0.007231116	0.015927998
TRMO	10.66899089	-1.087009771	0.404795957	-2.685327635	0.007245874	0.015957876
ADGRL3	2.280297838	2.748141482	1.023419743	2.685253533	0.007247481	0.015958785
NUBPL	7.756923198	-1.354405399	0.504465164	-2.684834348	0.007256576	0.015976181
SLC4A2	3.000713236	-2.527954838	0.941790325	-2.684201324	0.007270331	0.016003828
DDX10	102.6631086	0.482130579	0.17963649	2.6839234	0.007276378	0.016014501
LOC114112928	1.161826328	3.597068383	1.340544163	2.683289728	0.00729018	0.016042237
LOC101123328	7.808387455	1.276605744	0.475788071	2.683139452	0.007293457	0.016046806
NME8	1.119108573	3.481247542	1.297747237	2.682531267	0.007306732	0.016073368
LOC105615318	7.784361708	-1.287783038	0.480145822	-2.682066527	0.007316891	0.016093066
DNAJC12	9.257369707	1.356037611	0.505616057	2.681951243	0.007319413	0.01609355
DZIP1L	9.766328884	1.135579841	0.42341631	2.68194638	0.007319519	0.01609355
LOC121820049	1.711172448	3.378460158	1.259838217	2.681661908	0.007325746	0.016104591
INTS7	34.56453961	-0.653976935	0.24393315	-2.680967858	0.007340957	0.016135377
BMPER	1.102921265	3.51140738	1.309925191	2.680616728	0.007348663	0.01614966
GIN1	38.95156789	0.608219001	0.2269589	2.679864071	0.007365206	0.016183355
LOC105605908	77.53498158	-0.450955728	0.168281689	-2.679767063	0.007367341	0.016185384
LRRC17	1.971379705	3.058786602	1.141587366	2.679415253	0.007375087	0.016199739
ZBTB46	1.437937968	3.20512551	1.19629091	2.679219145	0.007379408	0.016206567
FRRS1L	1.960649988	3.106737981	1.159615543	2.679110332	0.007381807	0.016209172
SEMA3A	6.959314387	1.389224428	0.518613555	2.678727571	0.007390249	0.016225045
LOC101121538	156.4253862	-0.512686257	0.191426745	-2.67823734	0.007401075	0.016246145
SLC30A5	21.72764206	0.930157574	0.34732458	2.678064343	0.007404899	0.016251869
ATOH1	5.535114492	1.731037137	0.646580253	2.677219308	0.007423603	0.016290243
AP1B1	68.57091275	-0.763755834	0.285336863	-2.67668126	0.007435533	0.016313746
DEGS1	1.862640896	-3.186316442	1.191281435	-2.674696632	0.00747969	0.016407933
REXO2	27.98691743	0.689211729	0.257770272	2.673744044	0.007500968	0.016451909
LOC101120904	2.469369724	2.674175763	1.000246127	2.673517739	0.007506031	0.016459509
NUMBL	5.917877157	-1.471528315	0.550416992	-2.673479082	0.007506896	0.016459509
SNAP29	5.397758939	-1.631577593	0.610372707	-2.673084126	0.00751574	0.016476198
TM4SF19	2.825241833	-2.371979826	0.887418843	-2.672897747	0.007519917	0.016482651
LOC114117067	1.967904533	3.060749831	1.145160658	2.672768933	0.007522805	0.016486278
JADE3	9.958713969	-1.157737938	0.43319734	-2.672541659	0.007527903	0.016494745
KIF27	10.51875982	1.117962715	0.418351512	2.67230471	0.007533221	0.016502367
MBNL3	21.06312503	-0.779905246	0.291850486	-2.672276672	0.00753385	0.016502367

MAPK1	61.90849819	-0.536940397	0.20096303	-2.671836693	0.007543735	0.016521311
F5	6.211020496	-1.533236756	0.573956025	-2.671348831	0.007554709	0.016542635
GNPDA2	23.95777895	-0.758392137	0.283927879	-2.671073162	0.007560917	0.016553515
PDHX	15.48436866	-0.98790366	0.36989239	-2.670786658	0.007567373	0.016564937
SYNC	3.596269384	2.065205429	0.773567413	2.669716169	0.007591539	0.016615115
YAF2	44.31460268	0.589696471	0.221011325	2.668173092	0.007626496	0.01668889
ZSWIM3	5.987336503	-1.51964826	0.569570527	-2.66805986	0.007629066	0.016691783
ORMDL1	41.33782934	-0.638561287	0.239370901	-2.667664628	0.007638046	0.016708694
CPSF2	75.86747888	0.519333615	0.194683259	2.667582292	0.007639918	0.016710054
NTM	1.69691308	3.465392054	1.299194989	2.667337915	0.007645476	0.016719475
BCL7A	4.017727328	-1.863241031	0.698564489	-2.667242696	0.007647642	0.016721477
CCDC141	1.916178615	3.032038796	1.137171162	2.666299409	0.007669136	0.016765729
PC	9.056902102	1.202429944	0.451276237	2.664509773	0.007710063	0.016852444
ZNF260	18.90073564	-0.901994734	0.3385577	-2.664227501	0.007716536	0.016863835
WDR3	90.850592	0.534530073	0.200669986	2.663727069	0.007728024	0.01688618
SIK3	19.98614535	0.827248457	0.310626246	2.663163427	0.007740981	0.016911728
BBIP1	5.319451778	1.524036032	0.57234794	2.662778922	0.007749831	0.016925221
DUSP2	2.501404731	2.872816868	1.078899015	2.662730087	0.007750956	0.016925221
RTN1	1.699000657	3.452676006	1.296618492	2.66283107	0.00774863	0.016925221
EMC4	28.46442184	0.763548623	0.286784434	2.662447932	0.007757457	0.01693665
LOC132659800	9.330787525	-1.532218257	0.575514844	-2.662343594	0.007759862	0.016939134
RIGI	8.185695114	1.235277186	0.464049675	2.661950329	0.007768934	0.016956168
LCOR	120.1714897	0.446490347	0.167820431	2.66052438	0.007801908	0.017025355
STUB1	56.62206891	-0.577341646	0.217033975	-2.660144095	0.007810723	0.017041809
THAP11	19.29635705	-0.843807207	0.317220128	-2.660005256	0.007813943	0.017046053
FGD3	7.172380993	1.423512389	0.535235173	2.659601727	0.00782331	0.017063701
LOC101115653	15.41964301	-0.942407572	0.354361004	-2.659456208	0.00782669	0.017068289
ARID1B	59.89834778	0.750664724	0.282286537	2.65922963	0.007831956	0.017076987
SDR39U1	7.988883717	-1.231224829	0.463143595	-2.658408413	0.007851069	0.017115868
ZC3H6	21.47102228	0.952514619	0.358341818	2.658117394	0.007857852	0.017127861
HTT	11.75574626	-1.186113442	0.446320707	-2.657536217	0.007871413	0.017154624
ID1	112.0751462	0.522581669	0.196674591	2.657087861	0.00788189	0.017174656
RAP1GAP	12.2512428	1.153707395	0.434235582	2.656869781	0.00788699	0.017182968
OSBPL8	33.53063161	0.662854986	0.249539239	2.656315647	0.007899963	0.017208427
MKNK2	187.4311072	0.455211333	0.171376415	2.656207584	0.007902495	0.017211138
CALML4	60.29652414	-0.572086784	0.215517383	-2.654480934	0.007943053	0.017296651
ATG16L2	6.641158366	-1.387526687	0.522774288	-2.654160156	0.007950608	0.017310283
DYRK2	74.26692232	0.506762941	0.190946524	2.653952169	0.00795551	0.017315315
NT5C2	99.07061563	-0.528786103	0.199242745	-2.653979215	0.007954873	0.017315315
ZNF576	16.06173475	-0.906196101	0.341459266	-2.653892257	0.007956923	0.01731557
OIP5	5.495535577	-1.45735845	0.549231038	-2.653452464	0.007967299	0.017335328
SPTAN1	16.16494067	-0.930350602	0.350633764	-2.65334003	0.007969954	0.017338282

EPHX3	2.552168688	-2.352192203	0.886537129	-2.653235972	0.007972411	0.017340727
MUS81	20.09090416	-0.767249555	0.289180835	-2.653182579	0.007973673	0.017340727
TYSND1	57.77956613	-0.68016692	0.256426293	-2.652485092	0.007990166	0.017373768
LOC105602040	5.716187436	-1.480010394	0.55801884	-2.652258826	0.007995522	0.017382588
ZGRF1	11.35087159	-1.075958958	0.405842055	-2.651176593	0.008021189	0.017435553
CEP295NL	11.16918074	1.194724254	0.450708678	2.650768252	0.008030893	0.017453806
BAIAP2	5.035253356	1.609471946	0.607346756	2.650005011	0.008049058	0.017490441
ADAMTS19	3.123721323	2.191136786	0.82688586	2.649866073	0.008052368	0.017494791
PIPOX	4.701339279	1.798142298	0.679140008	2.64767541	0.00810473	0.017605692
LOC121816362	3.395378354	2.182269395	0.824348114	2.647266802	0.00811453	0.017624117
BET1L	40.88240868	0.844519341	0.319070432	2.646811664	0.008125459	0.017644986
UFC1	106.7648539	0.482208459	0.182225042	2.646224989	0.008139566	0.017672748
LOC132657209	1.317306235	-3.277427449	1.23869121	-2.645879314	0.008147888	0.017687944
CTSL	6.897743084	-1.49482494	0.564976009	-2.645820205	0.008149312	0.017688162
LOC121817804	1.135401803	3.498116853	1.322213796	2.645651454	0.008153378	0.017694114
EBNA1BP2	131.745262	-0.496405882	0.187677998	-2.644987089	0.008169403	0.017726014
UNC119B	30.23646711	0.742987676	0.280977307	2.644297807	0.00818606	0.017759272
SBSPON	1.575129409	3.336328445	1.261786651	2.644130403	0.00819011	0.017765175
BCAR1	19.73143406	-1.049057806	0.396811024	-2.643721423	0.008200011	0.017783766
AFF4	80.14455492	0.487574392	0.184439624	2.643544713	0.008204293	0.017790165
PMS2	22.4409665	0.735764771	0.278347278	2.643333807	0.008209406	0.017798364
NFKB2	6.366090487	1.42144932	0.537828479	2.642941711	0.008218918	0.017816098
LCP1	5.492036475	1.7130165	0.64820863	2.64269314	0.008224954	0.017826291
SAC3D1	10.6638286	1.109191221	0.419776774	2.642335857	0.008233636	0.017842215
CIAO1	6.076088434	-1.617649449	0.612220478	-2.642266156	0.008235331	0.017842995
LOC121820186	2.033734478	-3.271572575	1.238588369	-2.641371949	0.008257101	0.017887264
LOC105616708	4.393016875	1.787661004	0.677116904	2.640106889	0.008287988	0.017951264
AKR1A1	18.82463253	-1.161698936	0.440038499	-2.639993861	0.008290753	0.017951434
PROS1	22.16156731	0.834587406	0.316131357	2.640001971	0.008290555	0.017951434
ACSBG2	1.126362803	3.489713208	1.322098454	2.639525972	0.008302206	0.017972497
LOC132657995	1.282556587	3.661728448	1.387288125	2.639486623	0.00830317	0.017972497
LOC132657725	1.182324586	3.613705647	1.369495066	2.638713886	0.008322118	0.018010595
SPIRE1	13.17134139	-0.966724427	0.366410845	-2.63836194	0.008330761	0.018026381
CRYZ	13.25558619	-1.207841744	0.457845274	-2.638100276	0.008337192	0.018034458
LOC121820452	1.970719568	3.086599644	1.170002961	2.638112678	0.008336888	0.018034458
CALCOCO2	129.0185183	0.416667747	0.157965448	2.637714461	0.008346683	0.018052065
GPNMB	1.420105645	3.232476305	1.225710198	2.637227225	0.008358682	0.018075092
PPP1R14A	3.004122109	2.273128681	0.861998311	2.6370454	0.008363164	0.018081858
LOC101123603	11.28614108	1.133184372	0.429849239	2.636236774	0.008383121	0.018122077
PHC2	15.10793671	-1.235515243	0.468837512	-2.635273866	0.008406942	0.018170632
LOC105607801	12.8175325	1.15378112	0.437863344	2.6350256	0.008413094	0.018180988
H1-5	2.574773219	2.574166251	0.977011512	2.634734821	0.008420304	0.018193628

PARD3B	82.52596241	0.627606359	0.238211336	2.634662022	0.00842211	0.018194589
EIF2B4	31.44948495	0.793340385	0.301350941	2.632612934	0.008473085	0.018301754
MEAK7	10.25983622	1.153126525	0.438131397	2.631919407	0.0084904	0.018336191
LOC132659389	1.829935477	-3.08168107	1.171056865	-2.631538366	0.008499927	0.018350836
NT5C3B	13.39745828	-1.135041132	0.431314282	-2.631587171	0.008498706	0.018350836
METTL25B	3.633162326	-1.96279028	0.746061972	-2.630867612	0.008516721	0.018384122
LOC105616076	13.95456429	-0.983523895	0.373955689	-2.630054638	0.008537115	0.018425169
AMMECR1L	24.0170126	0.743291555	0.2826466	2.629755874	0.00854462	0.018438391
TBC1D2B	16.53062801	0.855192261	0.325318297	2.628786238	0.008569021	0.01848806
CPSF1	18.1343201	-0.870196783	0.331072554	-2.628417162	0.008578325	0.018505146
MARCKS	186.4289176	-0.416180606	0.158346192	-2.62829565	0.00858139	0.018508771
CDK5RAP2	71.57855867	0.678544797	0.258177937	2.628205978	0.008583652	0.018510664
PREP	23.63099818	-0.72768073	0.276887996	-2.628068891	0.008587112	0.018515138
CCDC88C	24.00034867	0.759312837	0.289056787	2.626863891	0.00861758	0.018575749
CDK5RAP1	6.257436757	-1.440893972	0.548525994	-2.626847202	0.008618002	0.018575749
SEC63	179.4732347	0.358688943	0.136581436	2.626191034	0.008634634	0.018605597
TRIM8	20.43889052	-0.785886452	0.299247668	-2.626207437	0.008634218	0.018605597
FAM241B	32.15480641	-0.681179713	0.259419805	-2.625781451	0.00864503	0.018624996
NAB1	53.83435317	0.567205352	0.216054478	2.62528857	0.008657556	0.018648975
NDUFAF2	21.57162254	0.783915945	0.298634029	2.62500542	0.008664759	0.018661483
KIAA1328	22.3591028	-0.748521916	0.285212376	-2.624437011	0.008679234	0.018687648
LOC132658253	3.799197547	1.872804722	0.713607469	2.62441861	0.008679703	0.018687648
DNAJC22	9.098818335	-1.168206961	0.445192871	-2.624046874	0.008689183	0.018702031
TMEM37	1.649134898	-3.08228767	1.174616296	-2.62408046	0.008688326	0.018702031
BMT2	69.7847528	-0.562879399	0.214607465	-2.622832334	0.008720217	0.01876376
RNFT1	12.60273444	-1.076632899	0.410487602	-2.622814653	0.00872067	0.01876376
SEC24C	9.653654683	-1.152283944	0.439548507	-2.621517141	0.008753936	0.018832305
OSER1	10.12600913	-1.038648578	0.396334097	-2.620638963	0.008776515	0.018877841
LOC101117395	2.661421179	-2.283309162	0.871472913	-2.620057523	0.008791494	0.018907016
FREM1	2.932012309	-2.381631714	0.909170618	-2.619565202	0.008804194	0.018931283
LOC132657667	3.364240939	2.046471137	0.781269151	2.619418844	0.008807973	0.018936362
SRRM2	620.0152658	0.480200762	0.183344018	2.619124242	0.008815583	0.018949676
BBX	365.6759953	0.382394116	0.146023174	2.618722127	0.008825981	0.018968976
SCGN	7.922902634	-1.206391786	0.460705258	-2.618576117	0.008829759	0.018974045
GVQW3	6.128684327	-1.397979538	0.533890471	-2.618476286	0.008832343	0.018976547
MRPS14	18.57671651	-0.947108288	0.361723587	-2.618320512	0.008836376	0.018982161
RUFY2	44.65607179	0.67811859	0.258999602	2.618222517	0.008838915	0.018984563
LOC101110239	1.862264527	-3.110574084	1.188229876	-2.617821809	0.0088493	0.019003815
HAS3	11.73852332	0.986805014	0.377007977	2.617464547	0.008858569	0.019020664
TOP3B	10.95958724	-1.043618783	0.398804525	-2.61686796	0.008874066	0.019050878
RAB13	107.2453538	0.55341702	0.211514023	2.616455464	0.008884795	0.019070849
SCD	2.863258416	2.291793153	0.876060405	2.616021841	0.008896087	0.019092019

ALDH1A1	88.87010501	-0.527200258	0.201550211	-2.615726656	0.00890378	0.019105463
MCFD2	45.16137822	-0.57506421	0.219874486	-2.615420369	0.00891177	0.019119537
LOC105603715	2.308571969	-2.5172552	0.962629235	-2.614978964	0.008923295	0.019141191
LOC121816631	1.437698513	3.199423948	1.223806568	2.614321602	0.008940484	0.019173691
RNPC3	228.456223	-0.359765499	0.137615	-2.614289847	0.008941315	0.019173691
GNAI3	111.5436887	0.39986974	0.153035593	2.612919851	0.008977235	0.01924763
KRT8	615.4318452	-0.506848019	0.194001183	-2.612602723	0.008985568	0.019262407
DAPK2	2.341720627	2.778640236	1.063770016	2.612068582	0.00899962	0.019289435
STMN2	2.338471921	2.692814129	1.030958778	2.611951308	0.009002708	0.01929296
GPR160	11.21338228	-1.014563647	0.388502775	-2.611470785	0.009015369	0.019316996
LOC114117093	2.23493829	2.765504811	1.059009434	2.611407153	0.009017047	0.019317495
AGAP2	2.671626851	2.413272942	0.92438938	2.610667101	0.009036581	0.019356241
TCF7	4.725504437	1.723867266	0.660334161	2.610598344	0.009038398	0.019357031
ZNF410	6.103920369	1.439218229	0.55131311	2.610527854	0.009040261	0.019357919
EMC8	8.952068552	-1.211191352	0.463991772	-2.610372479	0.009044368	0.019363612
HOXC5	19.77171489	2.038544535	0.780957762	2.610313429	0.00904593	0.019363853
CD320	9.948773574	1.375132361	0.527041117	2.609155751	0.009076592	0.019423267
LOC114116623	4.75810713	-1.665535982	0.6383343	-2.609190798	0.009075662	0.019423267
TSPAN18	3.528546182	1.960454602	0.751497336	2.608731272	0.009087858	0.019444263
C18H15orf40	93.7851133	0.550272899	0.210984701	2.608117533	0.009104169	0.019465246
CENPC	59.30963884	-0.55022365	0.210958639	-2.608206294	0.009101808	0.019465246
LOC114116239	4.995597125	2.093406567	0.802659394	2.60808829	0.009104947	0.019465246
LOC132659061	31.73665822	0.695332174	0.266595387	2.608192818	0.009102167	0.019465246
NONO	112.4916285	0.591435462	0.226766445	2.608126005	0.009103944	0.019465246
FBXW8	6.30826628	-1.392595591	0.533966279	-2.6080216	0.009106721	0.019465925
AUP1	87.24767808	0.480415349	0.184250484	2.60740346	0.009123179	0.019497988
KCNJ14	7.759135681	-1.221824976	0.468620478	-2.607280377	0.00912646	0.019498763
TGFBR2	129.7336883	0.471995922	0.18102969	2.607284595	0.009126347	0.019498763
ABCC3	8.659167028	-1.130348594	0.433558857	-2.607139895	0.009130205	0.019503647
MRPS11	28.53093012	-0.771105574	0.295802722	-2.606823794	0.009138637	0.019518541
LOC114118111	8.332826082	-1.358089674	0.520987965	-2.606758245	0.009140387	0.019519158
ESYT2	39.62440968	0.582835449	0.223620581	2.606358717	0.009151057	0.019538822
MTCH2	296.944165	0.33657911	0.12915783	2.605952048	0.009161929	0.019558911
PLXNB2	12.38283246	1.059106665	0.406427331	2.605894297	0.009163474	0.019559085
TNIK	77.34048038	0.457404349	0.175606287	2.604715112	0.009195068	0.019623388
SCHIP1	3.776472433	2.030442412	0.779772367	2.603891211	0.009217201	0.019667482
SLC2A10	3.315154644	2.159436947	0.829361776	2.603733387	0.009221446	0.019673399
RBPMS	21.49741834	0.899072671	0.345445321	2.602648285	0.00925068	0.019732619
E2F1	4.030226834	-1.796825888	0.69042893	-2.60247769	0.009255284	0.019739289
LOC114108824	3.25047025	-2.094441476	0.804934611	-2.602002009	0.009268131	0.019763535
RPS6KA3	54.5414097	-0.58208675	0.223746734	-2.601542998	0.009280543	0.019786846
SPCS2	163.2041025	0.477072753	0.1834394	2.600710386	0.009303096	0.019831766

ARFIP2	31.20591915	0.766740424	0.294914324	2.599875154	0.009325768	0.019876928
LOC114117229	4.781828739	1.717547331	0.660792095	2.599224997	0.009343451	0.019911442
SCAF11	207.105072	0.38261888	0.147218295	2.598990016	0.009349849	0.019921901
LOC101112480	30.97547586	0.636320619	0.244839746	2.598926969	0.009351567	0.019922384
MMP23B	3.038503978	2.305974487	0.88747918	2.598342067	0.009367513	0.019953175
LOC105604869	2.978377108	2.240752336	0.862568388	2.597767744	0.009383194	0.019983392
COX15	19.40411388	0.803041149	0.309149847	2.597578991	0.009388352	0.019991193
SUPT6H	43.22026159	0.549630389	0.21161534	2.597308819	0.009395741	0.020003739
DPY19L1	61.40730762	0.519279336	0.199943932	2.597124764	0.009400777	0.020011274
TMEM131L	47.8771039	-0.559695408	0.215517938	-2.596978305	0.009404786	0.02001662
HMGN3	84.54346923	0.498197153	0.191846787	2.59684909	0.009408325	0.020020963
LOC101116298	12.64005382	1.046638859	0.403075028	2.596635329	0.009414181	0.020030237
AKAP6	3.773814674	1.95604499	0.753385637	2.596339634	0.009422288	0.020044294
IBTK	164.6848445	0.385186639	0.148365444	2.596201846	0.009426067	0.020049143
KNOP1	302.4741494	0.588651168	0.226747664	2.596062767	0.009429884	0.020050879
LOC105613872	19.59437128	-0.903142036	0.347884412	-2.596098025	0.009428916	0.020050879
OGG1	12.95414195	-0.932946758	0.359394189	-2.595887157	0.009434704	0.020057939
GFI1	2.555135125	2.457222811	0.946718836	2.595514864	0.009444932	0.020076488
NDUFS5	83.94651425	-0.474520246	0.182885815	-2.594625752	0.009469397	0.020125291
LOC132659977	7.513094523	-1.330109535	0.512654988	-2.594551046	0.009471455	0.020126465
HSPA13	39.00454543	0.877276549	0.338241552	2.593639199	0.009496609	0.020176708
OAZ1	250.7891784	-0.399193741	0.153929682	-2.593351302	0.009504563	0.020190399
MARS1	21.92161519	-0.797394206	0.307496495	-2.593181447	0.009509259	0.020197164
LOC132658585	5.38532535	1.514598407	0.584131619	2.592906045	0.009516877	0.020210132
SYT13	2.548953003	2.492509001	0.961415712	2.592540324	0.009527001	0.020228419
BRPF3	18.77386419	0.846285062	0.326472666	2.592208012	0.009536209	0.020241539
TYMS	9.288612353	-1.35984213	0.524585675	-2.592221242	0.009535843	0.020241539
LOC101120834	7.524087691	-1.214932305	0.468741161	-2.591904455	0.009544628	0.02025217
LOC105610483	5.845425124	1.676217058	0.646722732	2.591863521	0.009545763	0.02025217
POGLUT3	31.47241425	0.660196069	0.254713066	2.591920705	0.009544177	0.02025217
DIPK1A	2.19638713	2.646362536	1.021208536	2.591402679	0.009558557	0.020276095
ANO6	81.096224	-0.492301505	0.190033685	-2.590601264	0.009580843	0.020320144
SMAP2	41.70444952	0.629432156	0.243073155	2.589476222	0.009612206	0.020383428
GOSR1	50.98351159	0.532446953	0.205669316	2.588849725	0.009629711	0.020417309
LHFPL2	12.71576774	1.001529609	0.386980661	2.588061134	0.009651785	0.020460866
TCEAL1	5.055283583	1.683051927	0.65034285	2.587945615	0.009655022	0.020464483
NKAPL	2.91918053	2.526308395	0.976427107	2.587298507	0.009673175	0.020499708
NKIRAS2	19.74488704	-0.859821432	0.332339995	-2.587174113	0.009676668	0.02050386
BPNT2	42.73362322	0.557432154	0.215480407	2.586927326	0.009683601	0.020515298
LOC101109351	1.412213602	3.151411456	1.218288032	2.586754013	0.009688473	0.020521492
PROSER2	4.335947354	-2.013304844	0.778329343	-2.586700426	0.00968998	0.020521492
RGS3	13.41947923	0.933539989	0.360905636	2.586659489	0.009691131	0.020521492

SFXN3	2.165492708	2.699590324	1.043752891	2.586426678	0.00969768	0.020532107
CADPS	7.851608728	-1.188856227	0.459700111	-2.586156058	0.009705298	0.020544981
SPRYD4	23.77061653	-0.735642216	0.284564738	-2.585148887	0.009733696	0.020601833
FOXK2	33.77304119	-0.649209826	0.25120243	-2.584409019	0.009754604	0.020642817
GTF2B	14.53855861	1.01823658	0.394396503	2.581758642	0.00982983	0.02079872
CEP95	28.18431596	0.867819462	0.336160778	2.581560727	0.009835469	0.020799231
IGLON5	1.187019117	3.547906115	1.374341506	2.581531665	0.009836297	0.020799231
LOC101102356	44.32889861	0.572030844	0.221582041	2.581575845	0.009835038	0.020799231
LOC114110196	1.749309879	2.879813619	1.115529535	2.581566449	0.009835306	0.020799231
DEDD	19.23710968	-0.814343282	0.315525219	-2.580913456	0.009853928	0.020830257
IFIT3	6.759577632	-1.397928486	0.541642137	-2.580907927	0.009854086	0.020830257
ACAA1	28.20186956	0.733155857	0.284141016	2.580253522	0.009872781	0.020866475
CEP19	22.99212462	0.806199208	0.312496976	2.579862433	0.009883968	0.020883538
MST1	1.834214735	2.842945284	1.101975696	2.579862054	0.009883979	0.020883538
NSMCE1	50.786628	0.57523743	0.223000649	2.579532538	0.009893414	0.020900169
PLPP5	7.140446374	-1.236333835	0.479337755	-2.579254029	0.009901394	0.020913723
IFIH1	63.26330901	0.487530661	0.189040796	2.578970638	0.009909521	0.02092758
LOC105604550	1.674081136	-3.043000028	1.180004734	-2.578803236	0.009914324	0.020934416
LOC132658084	2.438588666	2.483314424	0.963027539	2.578653594	0.009918619	0.020940178
HACD2	29.57671896	0.626231656	0.242863206	2.578536559	0.00992198	0.020943965
TNFSF15	3.602643725	1.906118431	0.739405753	2.577905869	0.009940107	0.020978916
PANK4	13.46124826	1.045237456	0.405705448	2.576345623	0.009985078	0.021070501
AUTS2	31.74836457	-0.680780548	0.26427438	-2.576036877	0.009993999	0.021085996
ATP5PD	301.2581346	0.312564848	0.121447604	2.573660067	0.010062909	0.021228036
MTSS1	30.22550122	-0.62627834	0.243365775	-2.57340351	0.010070372	0.021240429
LRRC14	26.78168257	-0.82246169	0.319703716	-2.572574694	0.010094517	0.021287995
RHOT2	128.3502892	0.588584817	0.228966799	2.570612065	0.010151898	0.021405626
MELK	3.908205137	-1.983040828	0.771446051	-2.570550236	0.01015371	0.02140607
TBC1D9B	19.43453732	-0.943904091	0.367306601	-2.569798875	0.010175757	0.021449168
MINDY4	3.114225504	-1.972485602	0.767634734	-2.56956273	0.010182695	0.021460408
BSDC1	12.27109312	0.997738922	0.38835423	2.569146529	0.010194934	0.021479427
LYPLAL1	24.62196302	-0.778056871	0.302845124	-2.569157665	0.010194606	0.021479427
CCDC9B	2.349014974	2.473849881	0.962997298	2.568906358	0.010202002	0.021490932
NUDT22	5.461492527	1.524016314	0.593280694	2.568794717	0.010205289	0.021494468
SOCS6	23.27257676	-0.921641881	0.358848545	-2.568331111	0.010218949	0.021519848
GLUD1	4.414764771	-1.61175923	0.627642249	-2.56795847	0.010229941	0.021539602
TRMT1L	131.4478365	0.469043649	0.182736606	2.566774446	0.010264936	0.021609881
CNST	6.174963799	1.435017177	0.559206903	2.566164989	0.01028299	0.02164448
LOC101112800	7.812859096	1.448668939	0.564541455	2.566098425	0.010284964	0.021645226
TAF4B	11.63526273	1.001696162	0.390391898	2.565873339	0.01029164	0.021655866
MRPL14	40.52407187	0.575757625	0.224437215	2.565339379	0.010307493	0.02168581
ZSCAN25	24.91817066	-0.681429108	0.265682166	-2.564828184	0.010322691	0.021714366

TPR	215.0075322	0.591664949	0.230754195	2.564048508	0.010345908	0.021759782
MFAP1	75.02779765	0.58140888	0.226766464	2.563910331	0.010350028	0.021765021
ADAM11	3.473528364	-2.12746338	0.829808322	-2.56380097	0.010353289	0.021768455
LOC132657509	4.843634963	2.144356565	0.83655494	2.563318275	0.010367696	0.021795317
TDRD12	2.98272658	-2.268799851	0.88518246	-2.56308722	0.010374598	0.021806397
LOC101121062	7.460375159	-1.224489181	0.477857793	-2.562455188	0.0103935	0.021842692
XRCC5	56.60931991	-0.702724799	0.274276334	-2.562105115	0.010403982	0.021861284
SLC35D2	11.1821661	-1.015535667	0.396409925	-2.561832091	0.010412164	0.021875038
SLC25A16	38.38438457	-0.588800105	0.229853627	-2.561630695	0.010418203	0.021880846
STAMBPL1	13.4319366	-0.925859206	0.361429312	-2.561660542	0.010417308	0.021880846
DBF4B	1.251774368	-3.211747246	1.253818291	-2.561573132	0.01041993	0.021881035
PPME1	76.36912691	-0.479010408	0.187030493	-2.561135355	0.01043307	0.021905186
HRAS	3.541938639	-1.827888384	0.713787021	-2.560831634	0.010442195	0.021918748
SRSF7	139.4026301	-0.785511654	0.306743292	-2.560811189	0.010442809	0.021918748
WNK4	3.317939434	-1.942842454	0.758800352	-2.560413222	0.010454777	0.021940422
SLC4A7	118.366464	-0.50830321	0.198535373	-2.56026522	0.010459231	0.021946323
LOC121816326	1.524122892	3.351075683	1.309274747	2.559490046	0.010482586	0.021991876
SELENOM	5.361373404	1.750723366	0.684176298	2.558877545	0.010501072	0.022027202
TRIM7	1.838581893	2.924105918	1.143174401	2.557882608	0.010531164	0.022086856
SPSB3	17.14256963	-1.05479363	0.412381644	-2.557809363	0.010533382	0.022088043
LOC132657922	55.8353285	-0.516212823	0.201846555	-2.557451737	0.010544219	0.022103831
MPND	16.82239329	-0.92910583	0.363293176	-2.557454675	0.01054413	0.022103831
LOC132658556	3.650013755	1.844634464	0.72129641	2.557387559	0.010546165	0.022104444
AMOTL1	38.26652736	0.678691194	0.265397394	2.557263975	0.010549912	0.022108832
CFAP298	55.27928459	-0.533416131	0.208611348	-2.556985211	0.010558371	0.022123089
ABCD3	42.05775765	0.775895779	0.303512359	2.556389403	0.010576469	0.022157536
DNAJC30	10.75749861	-1.043825467	0.408384599	-2.555986369	0.010588727	0.02217974
LOC132657158	1.149071802	3.51032396	1.37373425	2.555315164	0.010609169	0.022219078
ATG3	24.56859773	-0.713478733	0.279285606	-2.5546563	0.01062927	0.022257687
LOC101105383	8.981818981	-1.240957844	0.485781377	-2.554560352	0.0106322	0.022260335
MLXIPL	44.0681048	-0.727403975	0.284762627	-2.554422193	0.01063642	0.022265683
SYMPK	2.592510984	-2.543403153	0.995814694	-2.554092812	0.010646487	0.022283268
SMUG1	18.11448363	-0.871001971	0.341072497	-2.553715054	0.010658044	0.022303963
TRAFD1	7.827145856	-1.422798067	0.557203224	-2.553463452	0.010665747	0.02231659
LOC121820538	3.284594901	2.007143333	0.786068621	2.55339455	0.010667858	0.022317512
MORC2	69.00070617	-0.457595835	0.179241492	-2.552957081	0.010681266	0.022342065
SAT1	201.0093807	-0.444797188	0.174258559	-2.552512719	0.0106949	0.022367085
SRF	39.00337299	-0.901445044	0.353226189	-2.552033433	0.010709624	0.022394374
TELO2	8.009372239	-1.240939451	0.486269544	-2.551957996	0.010711943	0.022395719
TRIM34	3.634145173	1.817957073	0.712562592	2.551294574	0.010732357	0.02243489
NNAT	3.530103481	-1.972622353	0.773290084	-2.55094743	0.010743053	0.022453737
ZUP1	11.88381691	1.010330206	0.396161493	2.550298866	0.010763061	0.022492037

ZMYM6	42.72227241	-0.54987843	0.215635013	-2.550042414	0.010770981	0.022505071
CAMK2D	221.4159365	-0.463697007	0.181962994	-2.548303901	0.010824813	0.022610477
RHPN2	28.0922079	-0.866136013	0.339882312	-2.548340944	0.010823663	0.022610477
TLCD3A	2.580169121	-2.346281343	0.920783159	-2.548136683	0.010830003	0.022617784
IKZF5	13.1888192	0.952417786	0.373902421	2.547236213	0.010857991	0.022672693
TBCB	12.06727747	-0.948525284	0.372446326	-2.546743563	0.01087333	0.022701177
LOC132658851	1.62831037	-3.067011775	1.20432289	-2.546669003	0.010875654	0.022702481
TF	44.19748654	0.62118126	0.243932291	2.546531488	0.01087994	0.022707881
AATF	6.935424152	-1.281607183	0.503392544	-2.54593994	0.010898394	0.022742846
SGF29	4.146823588	-1.706466223	0.670314287	-2.545770329	0.01090369	0.022750347
GRB2	45.70070578	0.506346067	0.198915771	2.545530022	0.010911198	0.022758907
LOC105603234	13.21293905	-0.948118689	0.372462399	-2.545542026	0.010910823	0.022758907
GPATCH2L	59.58887875	0.515632937	0.202584415	2.545274454	0.010919188	0.022768466
TMEM69	8.546403154	1.292276734	0.507713559	2.545287023	0.010918795	0.022768466
TOP3A	20.98207784	0.840281102	0.330255678	2.544335065	0.0109486	0.022826235
EPC1	161.1476063	-0.373401034	0.146768988	-2.544141229	0.010954678	0.022835345
FASTKD2	17.96961292	-1.000916183	0.39344374	-2.543988078	0.010959483	0.022841797
HRH1	19.96160593	0.864678507	0.339907947	2.543860817	0.010963476	0.022846558
KLF16	41.32509825	-0.638611554	0.251067248	-2.543587662	0.010972052	0.022860865
LOC105608645	13.10336051	-0.934839588	0.367542038	-2.543490248	0.010975112	0.022863676
USP4	85.08909082	0.526303645	0.206945575	2.543198352	0.010984285	0.022879221
HMGN2	17.87485366	-0.81952243	0.322272263	-2.542950555	0.010992078	0.022891885
LOC121816507	11.10207584	1.131296906	0.444912442	2.542740544	0.010998687	0.022902079
FEZ2	21.55800684	-0.737814382	0.290215401	-2.542299204	0.011012586	0.022927449
C3H2orf68	10.13867423	-1.058452804	0.416356389	-2.542179805	0.011016349	0.022931711
POLDIP2	94.13523137	-0.492069013	0.193674816	-2.540696947	0.011063177	0.023025602
PIGW	10.24988602	-1.195929526	0.470807832	-2.540164891	0.011080022	0.023057072
PLA2G4A	10.20283052	-1.123238	0.442288261	-2.539606177	0.011097735	0.023090338
ZNF451	79.14587404	-0.568737312	0.224089423	-2.53799266	0.011149032	0.023193458
GOLT1B	27.3140285	1.040011081	0.410040982	2.536358869	0.011201188	0.023298332
TAF1C	17.10452543	-0.815616203	0.32170549	-2.53528842	0.011235478	0.023366018
ITPKC	17.23952424	-0.89294391	0.35236451	-2.534148262	0.011272103	0.023438539
C2CD2L	39.72913628	-0.64706536	0.255415817	-2.533380142	0.011296837	0.023482662
VPS36	114.861239	0.526229349	0.207714397	2.53342742	0.011295313	0.023482662
HEATR5A	9.566773567	1.177013179	0.464639749	2.533173672	0.011303493	0.023492243
LOC101118647	34.38177927	0.739975706	0.292119331	2.533128168	0.011304961	0.023492243
SERPINF2	29.13594656	-0.825449994	0.326007601	-2.531996159	0.011341523	0.023564558
LOC101115044	1.469929319	3.198728583	1.263517821	2.531605435	0.011354168	0.023587163
HSPA12A	1.344336577	3.145796312	1.242743059	2.531332836	0.011362997	0.023601836
LIN7C	82.41141961	0.502679589	0.198626256	2.53078117	0.011380883	0.023635314
ARID5A	2.785444872	2.357300438	0.931505481	2.53063507	0.011385624	0.023641486
HMGXB3	17.35374992	-0.899904518	0.355650645	-2.530304753	0.011396349	0.023660082

MON1B	3.495375017	1.855002007	0.733173456	2.530099789	0.011403009	0.023670231
TIGD3	3.21013898	-1.950781057	0.771482379	-2.528613888	0.011451393	0.023766974
HECTD4	6.905041661	1.266394157	0.500844577	2.528517261	0.011454545	0.023769827
IFT57	14.96271145	0.929487701	0.36763551	2.528285965	0.011462095	0.023781801
GGNBP2	222.6155563	0.408114326	0.161461748	2.527622367	0.01148378	0.023823095
DEK	482.5044849	0.365896868	0.144765999	2.527505569	0.0114876	0.023823625
MEMO1	112.0340417	-0.546337218	0.21615477	-2.52752793	0.011486869	0.023823625
PRC1	46.11608345	-0.617085912	0.244170879	-2.527270717	0.011495286	0.023835865
ENPP4	24.30925112	0.933950753	0.369678117	2.526389066	0.011524178	0.023892068
LOC132659243	3.216444099	2.075177777	0.821674377	2.525547633	0.011551812	0.023945645
C1H1orf43	12.25653441	-0.991381177	0.392824835	-2.523723271	0.01161193	0.02406653
PHRF1	14.87227037	-1.016793998	0.402977634	-2.523202064	0.011629156	0.024098299
RSRP1	60.03585567	0.679814084	0.26943066	2.523150421	0.011630864	0.024098299
SYT1	1.099412574	3.470791945	1.375611695	2.523089879	0.011632866	0.024098712
ASB7	49.17045022	-0.52861781	0.209558319	-2.522533166	0.011651296	0.024133151
LOC114117498	15.81247711	-0.984649888	0.390472947	-2.521685291	0.011679415	0.024181244
LRP12	12.59645564	0.975180631	0.386720256	2.521669386	0.011679943	0.024181244
POF1B	32.69518828	0.629497285	0.249635057	2.521670208	0.011679915	0.024181244
LOC101116620	5.055465619	-1.529672427	0.606634067	-2.521573563	0.011683124	0.024184086
BTD	40.45981849	-0.819212235	0.324915717	-2.521306892	0.011691983	0.024198675
NRDE2	13.0600982	0.934052948	0.370518923	2.520931835	0.011704452	0.024220732
UBE4A	46.19169582	0.55591145	0.220535301	2.520736808	0.011710941	0.024230408
LOC101117163	1.429698861	3.170144135	1.257721955	2.520544482	0.011717343	0.024239901
MKI67	44.00300567	-0.731547518	0.290276474	-2.520175021	0.011729649	0.024261606
LOC101105704	90.05128654	0.631740421	0.250687592	2.52003067	0.011734461	0.024267802
FBP2	10.41754535	-1.116556143	0.443102941	-2.519857213	0.011740245	0.024276007
SNX6	180.905859	-0.452553353	0.179606054	-2.519699884	0.011745493	0.024283103
ANO10	48.79704951	0.573214612	0.227550285	2.519067878	0.011766597	0.024322972
MTMR3	5.687581321	1.424892147	0.56572779	2.518688621	0.011779277	0.024345419
CENPH	23.62401807	-0.778897355	0.309415978	-2.517314589	0.011825319	0.024436799
NUP98	5.637680614	1.45612013	0.578800684	2.515753989	0.011877806	0.024541468
ASAH2	2.085093721	3.151816486	1.252916078	2.515584675	0.011883513	0.024549464
CNOT8	16.61152798	0.831847198	0.330749968	2.515033343	0.011902113	0.024584089
MYH9	148.4889099	0.435985502	0.173382432	2.514588693	0.011917132	0.024611309
CHKB	8.983335323	-1.071123258	0.42605437	-2.514052975	0.01193525	0.024644918
CUX1	33.26132615	0.663858238	0.264074057	2.513909336	0.011940112	0.02465115
EDIL3	1.329724564	3.133168231	1.246474637	2.513623733	0.011949785	0.024667309
RRP9	1.686715063	-3.048770007	1.213056435	-2.513296099	0.01196089	0.02468642
TTC39C	4.807300167	-1.489090818	0.592532736	-2.513094598	0.011967724	0.024696711
ANKMY2	35.96384513	0.630580919	0.2509648	2.512626944	0.011983598	0.024725652
LOC121818684	2.381220365	-2.464276691	0.98095952	-2.512108441	0.01200122	0.02475819
CNP	17.66664634	-0.922600742	0.36728852	-2.511923713	0.012007504	0.024767331

LOC101104530	58.79700668	-0.771693315	0.307246481	-2.511642487	0.012017076	0.02478325
NECTIN2	25.4039995	-0.690530204	0.275040822	-2.510646229	0.01205104	0.024845627
RNF10	151.4264815	-0.448472048	0.17862696	-2.510662712	0.012050477	0.024845627
LOC101106534	56.67932764	-0.557246036	0.222002304	-2.510091222	0.012069998	0.024880874
LOC121818064	26.18903738	0.701393748	0.279445966	2.509944082	0.012075028	0.024887406
KANK3	1.854084596	2.97186671	1.18410275	2.509804754	0.012079793	0.024893388
C17H4orf33	15.58568919	0.877760215	0.34976074	2.509601893	0.012086734	0.024903852
EVL	124.5046305	0.510263183	0.203347864	2.509311738	0.012096668	0.024920478
DMD	82.20442803	0.588195205	0.234421223	2.509138032	0.012102618	0.024928894
ANLN	22.35448689	-0.76561287	0.305141386	-2.509043036	0.012105873	0.024931757
FBXL3	14.43501072	0.867498807	0.345804796	2.508637291	0.012119786	0.024956564
GIMD1	2.339981034	2.666269528	1.062913243	2.508454518	0.012126058	0.024965633
MAGEH1	38.29832947	-0.671950155	0.267957897	-2.507670653	0.012152988	0.025017225
KNG1	20.72090086	-0.823518269	0.328414795	-2.507555326	0.012156955	0.025021537
FAM110C	27.34344384	-0.647807973	0.258502916	-2.505998707	0.012210607	0.025128094
BSCL2	3.42666394	1.981243115	0.790630417	2.505902976	0.012213913	0.025131029
PHETA2	2.508036385	2.381270045	0.950530512	2.505201058	0.01223818	0.025177085
RTN3	252.5089766	0.348884623	0.13931913	2.504211894	0.012272451	0.025243703
CDC27	74.36338685	-0.512486764	0.204684984	-2.503782899	0.01228734	0.025270441
LMF1	26.32338927	0.749407804	0.299350662	2.503444614	0.012299093	0.025290719
PRPF19	76.56286245	-0.527114919	0.210620026	-2.502681861	0.012325628	0.025337599
SPMIP6	8.061035019	1.13555447	0.453735327	2.50268031	0.012325682	0.025337599
PLEKHM2	19.80301295	-0.844236092	0.337362963	-2.502456356	0.012333483	0.025349736
OPA3	10.90764219	1.024862238	0.409582665	2.502210973	0.012342035	0.025363414
LOC101117099	22.55188082	-0.770253505	0.307948484	-2.501241421	0.012375878	0.025425144
STT3B	129.9652147	0.395847994	0.158258625	2.501272804	0.012374781	0.025425144
PRPH	1.438906736	3.13757638	1.254783612	2.500491997	0.012402094	0.025467258
RELB	22.13757016	-0.788535099	0.315348233	-2.500521702	0.012401053	0.025467258
SYT7	10.18755585	-1.147227272	0.458782264	-2.500592028	0.012398592	0.025467258
USP3	175.6116796	0.541561624	0.216629169	2.499947846	0.012421159	0.02550249
DCAF7	59.57645903	0.551078026	0.22065413	2.497474332	0.012508152	0.025677156
TAF12	16.30572388	-0.828069885	0.331625807	-2.497000742	0.012524869	0.025707525
HIP1R	14.58895929	0.934101039	0.374159847	2.49652935	0.012541529	0.025737767
EDC4	27.93149608	-0.866333417	0.347032387	-2.49640509	0.012545924	0.025742833
YDJC	6.556170737	-1.262524896	0.505760969	-2.496287719	0.012550076	0.025747401
RAB20	18.32923392	-0.752962908	0.301667453	-2.496003133	0.012560149	0.025762783
SH3D19	169.0179385	0.42093836	0.168647406	2.495966998	0.012561429	0.025762783
FXYD1	2.061288766	2.621992444	1.050582038	2.49575221	0.012569037	0.025774432
NOL11	79.81069508	-0.514347497	0.20623366	-2.49400363	0.012631125	0.025897779
PAIP1	49.1335629	-0.75748222	0.303736348	-2.493880715	0.0126355	0.025902775
KHDRBS1	150.8270457	-0.372106291	0.149216563	-2.493733159	0.012640753	0.025909571
COMMD1	7.85448547	-1.186152898	0.475706112	-2.493457344	0.012650578	0.025925733

LOC114114864	5.391485862	1.41823188	0.568993386	2.492527881	0.012683737	0.025989703
LOC132659929	1.645371538	2.791441226	1.120055446	2.492234858	0.012694207	0.026007169
ZC3H12C	6.171774843	1.433826424	0.57542616	2.491764407	0.012711032	0.026037648
PRDX2	74.97749403	-0.408722441	0.164033964	-2.491693983	0.012713552	0.02603882
KANTR	4.258729694	-1.669920664	0.67032778	-2.491200146	0.012731238	0.026071047
LOC121819719	2.561065208	-2.249905232	0.903256661	-2.490881418	0.012742664	0.026090449
ARL6IP4	51.07228527	0.693289289	0.27834801	2.490728388	0.012748153	0.02609452
INVS	9.631896217	1.112735708	0.446753142	2.490717136	0.012748557	0.02609452
IKZF2	11.87276756	1.164860496	0.467718696	2.490515143	0.012755806	0.02610536
KBTBD11	5.745545343	1.467215385	0.589265449	2.489905676	0.0127777	0.026146164
HMGCS2	7.232278558	1.655891273	0.665150393	2.489499053	0.012792326	0.026172085
FBXO22	17.31857221	-1.076520622	0.432560708	-2.488715694	0.012820544	0.026225803
MXI1	26.58629113	0.628669088	0.252677673	2.488027848	0.012845367	0.026264522
TLR1	1.055400376	3.465390035	1.392801572	2.488071599	0.012843787	0.026264522
UBE3A	198.6473782	0.38076963	0.153036934	2.488089766	0.012843131	0.026264522
RNF128	34.40472057	-0.901391102	0.362302094	-2.487954435	0.012848019	0.026265926
AGK	15.06928202	-0.846008913	0.340194272	-2.486840558	0.012888315	0.026344275
B3GNT8	2.781870199	-2.068696414	0.831904606	-2.486699074	0.012893441	0.026350724
PARL	13.13964069	-0.883066765	0.355201483	-2.486101009	0.01291513	0.026391015
ADGRL1	12.37817659	1.12863487	0.454002672	2.48596526	0.012920058	0.026397049
ZC4H2	18.29212244	-0.76100876	0.306197731	-2.485350747	0.012942385	0.026438623
LOC132657529	4.281445359	1.970055718	0.792873996	2.484702146	0.012965987	0.026482791
MRTFB	40.89163717	0.553169124	0.222651977	2.484456374	0.012974941	0.026497029
E2F7	15.39413718	-1.023892407	0.412189471	-2.484033382	0.012990363	0.026524472
NUP214	37.65918115	-0.812435261	0.32707059	-2.483975283	0.012992483	0.026524747
MED17	51.6319361	-0.626362423	0.252213673	-2.483459427	0.013011316	0.026559139
SPC25	25.6736857	-0.717308967	0.288852481	-2.48330554	0.013016938	0.026562504
STK32C	10.91245597	-1.127023717	0.453833507	-2.483341799	0.013015613	0.026562504
GLYR1	70.79808747	0.489117102	0.19701675	2.48261684	0.013042129	0.026609845
FAM83B	4.839173436	1.604038082	0.646180521	2.482337411	0.013052362	0.026626659
RBFA	21.78600732	0.706091645	0.284561244	2.481334543	0.013089146	0.026697624
USP36	96.18925567	0.536715823	0.216335049	2.480947147	0.01310338	0.026722578
CEP78	11.56456611	-1.01417952	0.408817618	-2.480762758	0.01311016	0.026732326
LOC101106447	15.89578749	0.807061926	0.325388617	2.480301656	0.013127127	0.026762841
ZNF775	15.39922069	-0.899930221	0.362913124	-2.479740082	0.013147818	0.026800937
LOC101118736	3.651685606	-1.748037449	0.7049636	-2.47961377	0.013152476	0.026806343
N4BP2	64.85026528	0.547145232	0.220854023	2.477406678	0.013234101	0.026968592
FBXL15	5.194262533	-1.722487729	0.695312358	-2.47728623	0.013238569	0.026973584
SRXN1	51.45520906	-0.630920695	0.25500844	-2.474116912	0.013356598	0.027209921
LOC132660093	2.220623078	2.367917916	0.957481857	2.473068183	0.013395858	0.027285742
DISP2	4.775719737	-1.697365391	0.686606209	-2.472109002	0.013431855	0.027354896
SARAF	87.21155307	0.714120858	0.288957628	2.471368774	0.013459694	0.027407415

LYAR	92.99338233	-0.579235341	0.234425676	-2.470869872	0.013478485	0.027441498
COPZ1	186.2263178	0.39409914	0.159511682	2.47066005	0.013486395	0.027453421
PELO	21.53170913	0.782745961	0.316826464	2.470582639	0.013489315	0.027455182
AGO4	11.4202781	1.023399866	0.414299225	2.470194978	0.013503943	0.02748077
MCM3AP	74.60398852	0.475840817	0.19265888	2.469861839	0.013516524	0.027502187
TTK	20.36037494	-0.812586896	0.32902462	-2.469684172	0.013523239	0.02751166
CIR1	70.24557692	0.468484065	0.189858299	2.467545881	0.013604279	0.027672316
RPL13A	349.4423257	-0.549239739	0.222603773	-2.467342449	0.013612011	0.027683831
PAGR1	82.38633636	0.579827886	0.235038112	2.466952625	0.013626839	0.027705556
RBM19	20.38869146	0.815483161	0.330562055	2.466959374	0.013626582	0.027705556
CBX7	11.50743785	1.014854704	0.411544415	2.465966412	0.013664415	0.027777729
FUT4	19.25903658	-0.815577086	0.330913266	-2.464624929	0.013715674	0.027877692
GANC	4.238314453	1.634071258	0.663146991	2.46411622	0.013735157	0.027913046
BSG	86.37478898	-0.566702037	0.23005316	-2.463352549	0.01376445	0.027968324
SCML1	7.241493703	1.302169931	0.528630804	2.463288028	0.013766927	0.027969106
NCMAP	1.652426995	-3.13261718	1.271765041	-2.463204349	0.013770141	0.027971383
EFNB2	51.0836784	0.515184945	0.209164312	2.463063315	0.013775559	0.027976993
SLC11A2	11.8776752	0.947889396	0.38484789	2.463023501	0.013777089	0.027976993
STIMATE	6.625444465	-1.229222442	0.499552641	-2.460646469	0.013868695	0.028158739
LIAT1	3.661538696	1.903947171	0.774103358	2.459551625	0.013911069	0.028240484
LCK	1.308347349	3.120249194	1.269180486	2.458475551	0.013952828	0.028320956
FKBP3	17.15781935	-0.792498874	0.32236374	-2.458399554	0.013955781	0.028322649
L1CAM	5.215563765	1.499003195	0.609781877	2.45826131	0.013961155	0.028327344
ORC2	26.5078024	-0.641174128	0.260827452	-2.458231006	0.013962333	0.028327344
VAV3	15.05140149	0.828045551	0.3368661	2.458085132	0.013968006	0.028334552
LOC132659062	31.90082427	-0.643577809	0.261924796	-2.457109132	0.014006013	0.02840734
TLE4	59.22272859	-0.503740045	0.20503962	-2.456793693	0.014018317	0.02842798
TMEM63B	11.42983166	-0.962948861	0.392023357	-2.456355836	0.01403541	0.028458328
LOC105604727	6.068423176	-1.347029688	0.54846433	-2.456002359	0.014049224	0.028482014
ADAL	26.14604167	0.637821513	0.259744128	2.455576254	0.014065891	0.028511479
CARMIL2	3.927319032	1.872495758	0.762806488	2.454745455	0.014098438	0.028573119
LOC105603125	38.99487589	-0.880404233	0.358775398	-2.453914724	0.014131049	0.028634869
BOLA2B	21.42523395	-0.737658902	0.30062025	-2.453789795	0.014135959	0.028640477
AKNA	8.569346188	1.199428785	0.488822227	2.453711633	0.014139031	0.028641738
GPR82	1.004443198	3.384850379	1.379522167	2.453639716	0.014141859	0.028641738
LOC101123370	1.004406997	3.384812086	1.379523017	2.453610446	0.01414301	0.028641738
LOC114109329	1.37565184	3.05220028	1.244037376	2.453463488	0.01414879	0.028649103
LOC101104855	30.82819209	0.651236377	0.26544581	2.453368459	0.014152529	0.028652333
LOC101111058	154.1186939	-0.344684384	0.14050989	-2.45309696	0.014163216	0.028665285
MORN4	4.703757872	-1.598267378	0.651529289	-2.45310135	0.014163043	0.028665285
IQSEC1	3.813012429	-1.747430441	0.712386221	-2.45292566	0.014169962	0.028674597
LIFR	5.863965971	1.515272656	0.617766147	2.452825659	0.014173902	0.028678227

MNAT1	151.7852488	0.410854848	0.167533918	2.452368175	0.014191937	0.028710372
DCUN1D3	12.22517694	0.951748003	0.388247824	2.451393015	0.014230448	0.028783924
EIF1AD	6.936559632	1.218446683	0.497269054	2.450276511	0.014274655	0.028868972
RGS14	8.977439805	1.188214403	0.484991842	2.449967816	0.014286898	0.028889362
GLT8D1	16.60961641	-0.926973177	0.378419815	-2.449589426	0.014301919	0.028915361
IL34	14.07606642	0.885696996	0.361647783	2.44905966	0.014322972	0.028953546
ALDH4A1	18.2484625	-0.779700255	0.318426855	-2.448600808	0.014341229	0.028986068
DDX39A	8.449233695	-1.047606025	0.428082093	-2.44720824	0.014396763	0.029093913
MRPL46	66.09950573	-0.621701008	0.254061461	-2.44704964	0.0144031	0.029102318
PHF5A	76.42609924	-0.518589083	0.211998099	-2.446196848	0.014437215	0.029166841
PHLPP1	3.411805752	-1.916634356	0.783570302	-2.446027309	0.014444006	0.02917615
RBP1	23.907358	-0.867913028	0.354886611	-2.445606573	0.01446087	0.029205801
LOC132658039	25.46068456	-0.680606742	0.278311355	-2.445486788	0.014465675	0.029211091
MFN1	78.93645474	-0.419585065	0.171605993	-2.445049023	0.014483245	0.029242154
DCAF11	21.22092121	0.992389174	0.406200906	2.443099363	0.014561727	0.029396171
SYS1	218.5358021	0.394645132	0.161551705	2.442841026	0.014572155	0.029412779
LOC101109513	5.616130635	1.51991886	0.622282124	2.442491599	0.014586269	0.029436822
NMUR1	23.31365772	-0.7197049	0.294750662	-2.441741419	0.014616612	0.029493604
LOC132660210	2.18861581	-2.437160625	0.998718951	-2.440286752	0.014675608	0.029608177
HAT1	30.56104633	-0.746949197	0.306155542	-2.439770292	0.014696604	0.029646062
MTFP1	14.42960713	0.869146314	0.356269091	2.43957822	0.014704419	0.029657351
LOC101118703	2.971043533	-2.052818031	0.841644519	-2.439055902	0.01472569	0.029695771
RPUSD2	7.911917402	-1.260576336	0.516861309	-2.438906365	0.014731785	0.029703581
JOSD1	9.026022617	1.056370827	0.433165239	2.438724834	0.014739187	0.029714022
LOC121819426	2.310628961	2.385755768	0.978427097	2.438358234	0.014754145	0.029739692
RUNX1	14.27814845	0.912148717	0.374102591	2.43823149	0.014759319	0.029745636
RELL1	21.78298338	0.71643495	0.293940585	2.437346139	0.014795509	0.029814077
GPR107	51.94747455	0.525478861	0.215611327	2.437157955	0.014803212	0.029825102
HSPA4	126.340734	-0.347669939	0.142672685	-2.436836025	0.014816396	0.029847167
PCBP3	1.558657565	-2.894787958	1.18842052	-2.435827983	0.014857748	0.029925959
KCNB1	1.385299575	3.141253599	1.289642727	2.435754907	0.01486075	0.029927495
LY6G5B	2.431557781	2.378901083	0.976826844	2.435335491	0.014877988	0.029957697
HSPBP1	35.37978115	-0.623688918	0.256174198	-2.434628169	0.014907099	0.030011793
SLC39A4	5.833818915	1.422753206	0.584695192	2.433324621	0.014960881	0.030115533
DOHH	26.60076288	-0.626952532	0.257666486	-2.43319394	0.014966282	0.030121868
LOC101111513	1.060897166	3.395643652	1.395921314	2.432546604	0.014993062	0.030171223
UBR7	22.00897209	0.771856711	0.317425645	2.431614221	0.015031708	0.030244439
LITAF	18.10023912	0.790850132	0.325319762	2.430993211	0.015057497	0.030291767
ZNF8	11.47129989	-1.197550718	0.492708644	-2.43054538	0.015076118	0.030324664
BST1	16.32481922	-1.085073899	0.446465801	-2.430362856	0.015083713	0.030334984
PIR	3.639507435	1.928094204	0.793352208	2.430313024	0.015085788	0.030334984
LOC114113024	3.738610306	-1.738878045	0.715589824	-2.429992693	0.015099127	0.03035724

ZFP90	11.84362692	-0.918538344	0.378156427	-2.428990433	0.015140932	0.030436712
SLC25A14	3.402770595	-1.812016239	0.746052021	-2.428806823	0.015148601	0.03044755
MBTD1	10.26639839	-0.966795751	0.398157233	-2.428175784	0.015174986	0.030495995
CEP250	16.41499308	0.975564729	0.401830366	2.427802405	0.015190617	0.030522818
LOC101106791	60.99332303	-0.546102055	0.224993551	-2.427189807	0.015216293	0.030569813
DNAI7	25.0208546	0.81794922	0.337074599	2.426611861	0.015240551	0.030613948
MVB12A	38.70636671	-0.578265529	0.23832607	-2.426362881	0.015251012	0.030630358
BCORL1	29.56346562	0.660659181	0.272338639	2.425873843	0.015271578	0.030667054
PPARD	17.14173628	0.887947463	0.366051952	2.42574164	0.015277142	0.030673618
CLTA	287.2455764	-0.377830024	0.155765177	-2.425638584	0.01528148	0.03067772
UBAP1	16.37463063	0.915767308	0.377595283	2.425261518	0.015297363	0.030704993
SIN3B	4.503871501	-1.46752229	0.605121439	-2.425169883	0.015301225	0.030708134
SH3GLB2	8.261716724	-1.160945726	0.478843842	-2.424476675	0.015330468	0.030762203
SUGP1	6.551223082	1.248618739	0.515248414	2.423333494	0.015378801	0.030854557
PEX13	14.07223585	0.872807787	0.360213362	2.423030014	0.015391655	0.030875711
ID2	28.00545921	-0.643638572	0.265653762	-2.422847574	0.015399387	0.03088521
TGFB1I1	2.009750537	2.635699017	1.087868984	2.42280923	0.015401012	0.03088521
SLC6A18	1.375911302	3.127702897	1.29100542	2.422687658	0.015406166	0.030890912
SYNPO	2.286791748	2.341535891	0.967175215	2.421004855	0.015477671	0.03102963
PRPS1	2.742889063	-2.042460025	0.84389668	-2.420272614	0.015508875	0.031087527
CDKN1B	22.60415698	-0.742036292	0.306601532	-2.420197602	0.015512075	0.031089278
LOC101122545	1.661655865	2.827135657	1.168237003	2.420001807	0.01552043	0.031101359
SLC3A2	16.53768542	0.777470405	0.321312458	2.419670904	0.015534559	0.031125006
SMIM5	20.93734554	0.72925021	0.301421028	2.419374043	0.015547244	0.031145753
MBNL2	104.903124	0.373642612	0.154462485	2.418986149	0.015563833	0.031174313
ZDHHC23	21.77769969	-0.764218128	0.315945605	-2.418828168	0.015570594	0.031183181
PRAF2	8.603540472	1.155766768	0.477866844	2.418595853	0.01558054	0.031198427
LOC105602911	15.79299474	-0.882240041	0.364800586	-2.418417284	0.015588189	0.031209068
PCBD1	24.9196513	0.697705266	0.288576763	2.417745834	0.015616981	0.031262029
RARB	1.176262386	3.602582471	1.490156279	2.417587015	0.015623798	0.031270992
SNX9	90.74265151	0.383907868	0.158822862	2.417207839	0.015640084	0.031298901
CLIC1	134.7595888	-0.524546433	0.217018693	-2.417056456	0.01564659	0.031307234
CDC23	11.7165528	-0.993024153	0.410900552	-2.41670192	0.015661837	0.031333051
LOC114113766	1.508568951	-2.998492541	1.24079402	-2.41659171	0.015666579	0.031337847
ABHD5	9.749389263	1.042087351	0.431334668	2.41596011	0.015693781	0.031387561
HABP2	3.053743073	-2.073637992	0.858374193	-2.415773923	0.015701807	0.031398916
SYNE2	264.2331302	-0.373263036	0.154520991	-2.415613788	0.015708713	0.031408027
DDA1	33.69324347	-0.750270327	0.310600378	-2.415548659	0.015711523	0.031408947
TYW5	18.63565709	0.779787683	0.322830888	2.41546801	0.015715003	0.031411205
SAMD15	3.397670632	2.024261732	0.838220394	2.414951659	0.015737298	0.031451066
SUPT3H	10.59668681	1.143945831	0.473726742	2.414779935	0.015744719	0.031461192
CBFB	15.14560274	-0.808675375	0.334897195	-2.414697364	0.015748288	0.031463621

RASSF8	12.10462205	0.983709759	0.407402149	2.414591481	0.015752867	0.031468064
EFL1	21.07929446	-0.705372003	0.292145291	-2.414456177	0.015758719	0.03147505
LOC105611265	10.75904264	-1.041989529	0.431645158	-2.413995641	0.015778651	0.031510153
NR1H2	12.98307039	0.983937517	0.407617818	2.413872688	0.015783976	0.031516078
PPFIBP1	90.28113815	0.438660177	0.18173475	2.413738574	0.015789787	0.031522971
CCDC57	13.14238025	-0.960454593	0.397925264	-2.413655727	0.015793377	0.03152543
PLEKHH2	4.058564464	1.80966801	0.749800937	2.413531274	0.015798772	0.03153149
ZBTB21	32.31825355	-0.594606521	0.246400824	-2.413167748	0.015814539	0.031558246
KCTD15	22.08172927	-0.673024089	0.278919595	-2.412968115	0.015823204	0.031570823
LOC101108778	35.68753115	-0.725442586	0.300720034	-2.412352032	0.01584997	0.031619506
NCK2	25.91083683	-0.758973955	0.314681194	-2.41188215	0.01587041	0.03165556
AKAP1	52.27738139	-0.534749418	0.221748785	-2.411510031	0.015886615	0.031682151
ARHGAP39	17.77985564	-0.781480982	0.324068681	-2.411467162	0.015888483	0.031682151
EHHADH	39.61930217	0.662062644	0.274579886	2.41118406	0.015900822	0.031702026
YAP1	57.67769269	-0.460758417	0.191110447	-2.410953582	0.015910873	0.031717335
VKORC1L1	15.93894183	0.855041542	0.354725755	2.410429834	0.015933736	0.031758174
LOC101117955	1.924121139	-2.733697875	1.13431048	-2.410008479	0.01595215	0.031785396
TMEM132A	3.168155826	-1.793022584	0.743979495	-2.410043012	0.01595064	0.031785396
UBE2Z	156.0297154	0.364205618	0.151210153	2.408605572	0.016013594	0.03190307
PRDM16	12.06428226	-0.916539297	0.380702879	-2.407492424	0.016062495	0.031995725
SSTR1	2.430249017	-2.323240484	0.965119532	-2.407204918	0.016075147	0.032016155
DOK3	1.618554844	2.768062712	1.150492694	2.405980262	0.016129136	0.032118897
RCAN3	67.59375655	0.58522432	0.243293309	2.405427103	0.016153574	0.03216277
STYXL2	1.262757722	2.995777136	1.246699794	2.402965934	0.016262702	0.032375228
HBS1L	101.4023118	0.48029277	0.199884835	2.402847466	0.016267971	0.032380895
GEMIN5	21.57588379	-0.678621286	0.2825939	-2.40140104	0.016332426	0.032504349
MAGED1	36.08033501	-0.638066582	0.265716906	-2.40130217	0.01633684	0.032508293
SPTBN1	260.5432358	0.395288874	0.164626574	2.401124335	0.016344782	0.032519255
CIT	8.931769347	-1.089958332	0.453979314	-2.400898673	0.016354865	0.032534472
BTRC	17.89050615	-0.747610631	0.3114331	-2.40054969	0.016370468	0.032560666
JADE2	15.16517631	0.845736555	0.3523801	2.400069004	0.016391981	0.032598605
FAM32A	41.23356297	0.555884786	0.231666675	2.399502585	0.016417364	0.032644226
SLC49A4	16.6268107	0.750736147	0.312910092	2.399207203	0.016430614	0.032665713
LRATD1	34.39280073	-0.642927157	0.268007728	-2.398912754	0.016443832	0.03268713
CCDC15	32.3984406	0.661001922	0.275638708	2.398073644	0.016481551	0.032757236
METTL24	2.030742119	2.652218559	1.106468344	2.397012598	0.016529355	0.032847363
EXPH5	53.542454	0.461416266	0.192536278	2.396515974	0.016551772	0.03288702
NOC3L	66.57746384	-0.475511415	0.198525385	-2.395217196	0.016610523	0.032998847
COG1	8.068371706	-1.15892151	0.483866258	-2.395127769	0.016614574	0.033001992
DND1	5.244215546	-1.366918279	0.570732265	-2.395025415	0.016619213	0.033006301
CSRP2	3.880683998	1.657313311	0.692006141	2.394940177	0.016623077	0.03300907
LOC132660231	9.218337623	1.092986289	0.456468601	2.394439151	0.016645805	0.033049292

NAPB	2.913368087	-2.114601326	0.883205557	-2.394234626	0.016655091	0.033062817
ASPSCR1	23.64905656	0.824139282	0.344279845	2.393806357	0.01667455	0.033096529
TIMM8A	43.62437358	-0.516244337	0.215689264	-2.39346329	0.016690151	0.033122578
AAAS	5.023447623	1.457539434	0.609001448	2.393326714	0.016696366	0.033129993
LOC114117038	2.12304121	2.432113646	1.016473085	2.392698521	0.016724977	0.033175228
LOC121819027	1.578646519	2.712997423	1.133882098	2.392662718	0.016726609	0.033175228
LOC132658202	2.56498606	2.34659535	0.980710265	2.392750881	0.016722591	0.033175228
ABHD18	6.137596372	-1.208016691	0.504902109	-2.392576047	0.016730561	0.033178141
IPO13	6.787958391	1.239839603	0.518377846	2.391768112	0.016767432	0.033246329
VAMP4	15.27732744	0.868145867	0.363077248	2.391077579	0.016799003	0.033303986
TTC17	31.18015853	0.626523987	0.262032507	2.391016265	0.016801809	0.033304608
MDH1	35.19318609	-0.57429454	0.24028874	-2.390018527	0.016847523	0.033386418
SYNJ2	46.71857287	0.495317279	0.207245163	2.390006461	0.016848076	0.033386418
IL11RA	24.9541279	0.769365618	0.32201892	2.389193833	0.016885391	0.033455401
IFNAR1	18.95773374	1.016779478	0.425708411	2.388441133	0.016920019	0.033519041
ABHD17B	14.48397713	-0.838571897	0.351233693	-2.38750414	0.016963212	0.033599628
CCDC181	5.309654222	-1.454971858	0.609530802	-2.387035819	0.016984836	0.033637476
MLF2	21.93851436	-0.659508316	0.276298942	-2.38693754	0.016989377	0.033641484
CDKAL1	55.46950613	0.471041947	0.197365327	2.38664995	0.017002672	0.033662822
OXNAD1	7.543996066	-1.174971661	0.492386378	-2.386279787	0.017019797	0.033691737
NDOR1	10.09426143	1.0657906	0.446685861	2.385995829	0.017032944	0.03371277
RBM33	41.71517364	0.578103547	0.242348557	2.385421864	0.017059546	0.033755424
RCAN1	170.070473	-1.203115583	0.504350726	-2.385474079	0.017057124	0.033755424
ZYX	10.57164588	0.995071991	0.417158801	2.38535538	0.01706263	0.033756529
EPS15	27.41295923	0.632600639	0.265239671	2.385015167	0.017078417	0.033782762
SPTBN2	7.705531513	-1.317125425	0.552290764	-2.384840578	0.017086524	0.033793797
CTSO	10.52330518	1.05867689	0.443955156	2.384648256	0.017095458	0.033806464
EPHA1	11.19855459	-1.243198297	0.522041189	-2.381418026	0.017246129	0.034099372
LOC132659409	5.730542801	1.34165292	0.563439601	2.381183216	0.017257126	0.03411607
NECTIN1	23.04971035	0.704555665	0.295919668	2.380901782	0.017270316	0.034132048
PRRC2A	79.88475315	0.551601658	0.231675194	2.380926718	0.017269147	0.034132048
C19H3orf62	3.40869764	-1.917240763	0.805321323	-2.380715259	0.017279062	0.034144286
PRPF3	20.9433223	0.692113648	0.290732148	2.380588639	0.017285002	0.034150974
HABP4	6.047519276	-1.214336428	0.510221671	-2.380017348	0.017311823	0.03419891
SYTL4	26.37546566	-0.667751295	0.280605791	-2.379677528	0.017327794	0.034225402
PARS2	11.7833275	-0.916428022	0.385120421	-2.379588231	0.017331993	0.034228638
DNAAF9	17.40140964	0.767306544	0.322471375	2.379456297	0.017338199	0.034235835
IPO5	86.27524646	-0.458483156	0.192691465	-2.379364112	0.017342536	0.034239341
PROB1	1.904770482	2.521297294	1.059956111	2.378680841	0.017374712	0.0342978
LOC121820716	1.969703766	-2.616330072	1.100130689	-2.378199335	0.017397418	0.034337551
BPTF	371.0498536	0.386612098	0.162612865	2.377500078	0.017430439	0.034397645
DKKL1	6.395246868	-1.260693864	0.530389242	-2.37692201	0.017457779	0.034446512

USP37	28.47830773	0.613395573	0.258111959	2.376470949	0.017479138	0.034483565
LOC132658160	45.20209	-0.494951276	0.208298094	-2.376168052	0.017493494	0.034506794
PCED1B	2.605964243	2.113794883	0.889755577	2.375702877	0.017515561	0.034545224
TRAPPC8	20.37383706	0.672479097	0.283073325	2.375635701	0.017518749	0.034546415
KIF3B	29.25185953	-0.671525436	0.282706194	-2.375347452	0.017532438	0.034568309
HNRNPH3	95.31972123	-0.546165779	0.230007395	-2.374557477	0.01757	0.034632153
LOC105615270	30.0840124	0.617556919	0.260066506	2.374611516	0.017567429	0.034632153
LOC132657978	1.049437907	3.385045547	1.42563501	2.374412471	0.017576903	0.03464065
MICAL3	20.2433946	0.755262932	0.318105377	2.374253905	0.017584454	0.034650422
SLC15A4	18.99099768	0.697231013	0.293791046	2.373220773	0.01763372	0.03474238
GRHL1	12.42669061	-1.03024558	0.434163021	-2.372946404	0.017646824	0.034763074
CAMKK2	44.25138854	-0.665716759	0.280561577	-2.372800885	0.017653778	0.034767199
HPN	7.340936033	-1.223263139	0.515537079	-2.37279371	0.017654121	0.034767199
RETREG2	226.9572425	0.409798691	0.172730736	2.372471161	0.017669542	0.034792443
UTP20	44.57174451	-0.504064074	0.212534086	-2.3716858	0.017707141	0.034861342
LTO1	15.03403067	-0.794939475	0.335253935	-2.371156288	0.017732531	0.034906187
LOC121816358	30.58487709	-0.625272433	0.263721602	-2.370956449	0.017742122	0.034919923
ZNF280D	22.19085064	0.723048773	0.305055667	2.37021912	0.017777546	0.034984493
PITPNM2	22.64915048	0.68285187	0.288120969	2.370017953	0.017787222	0.034998381
PITPNC1	6.383359744	1.253292569	0.529088686	2.368775976	0.01784706	0.035110951
ZNRF3	10.28380079	-1.119853173	0.472833934	-2.368385793	0.017865895	0.035142834
LOC132657958	1.918866584	-2.711858846	1.145215168	-2.36799068	0.017884986	0.03517521
OTUD4	88.84152988	0.426274724	0.180047366	2.3675699	0.017905337	0.035210054
CDK5R2	2.856821613	-2.212214755	0.934625146	-2.36695403	0.01793516	0.035263512
LOC101122127	1.941890247	-2.632398154	1.112670414	-2.365838187	0.017989305	0.035364767
ARSA	29.61860688	0.705206648	0.298100423	2.365668055	0.017997573	0.035368053
DENND10	19.1992315	-0.72589469	0.306856181	-2.365586017	0.018001561	0.035368053
LOC132659056	48.17546586	0.503712536	0.212924169	2.365689804	0.017996516	0.035368053
MARK2	65.42934036	-0.508198724	0.214826203	-2.365627266	0.017999556	0.035368053
OSBPL3	11.30296871	-0.93201515	0.394019107	-2.365405978	0.018010316	0.035380053
LOC105609229	2.256357893	2.275348636	0.961977923	2.365281553	0.018016368	0.035386742
SELENBP1	3.668533681	1.733037013	0.732735676	2.365159867	0.018022289	0.035393171
RNF4	17.4250983	0.763877557	0.323050742	2.364574533	0.018050795	0.035443943
RNF14	19.20979933	-0.764640591	0.323425413	-2.364194524	0.018069322	0.035475112
EXOSC7	18.82591449	-0.791388331	0.334858835	-2.363349111	0.0181106	0.03555093
SYN3	2.184150303	2.586349077	1.094508523	2.363023241	0.018126533	0.035576981
NQO2	46.58610277	0.599470365	0.253701603	2.362895455	0.018132784	0.035584025
RAB3IP	45.78713018	0.567699311	0.240346182	2.362006784	0.01817631	0.035664203
MANBAL	34.94927615	0.579718908	0.245440909	2.361948998	0.018179143	0.035664527
KLHL8	14.29279359	0.884188981	0.374387087	2.361697325	0.018191488	0.035683508
WWP1	34.37195518	0.576774073	0.244262644	2.361286456	0.018211657	0.03571783
ACOT6	1.180818844	2.94679525	1.248041603	2.361135432	0.018219076	0.035727137

HAGH	12.86296332	-1.038122469	0.439901636	-2.359896815	0.018280019	0.035841386
LOC132659816	1.803804803	2.433275735	1.031317977	2.359384583	0.018305274	0.035880377
SERPINE1	1.821785752	2.476998656	1.049832433	2.359422873	0.018303385	0.035880377
COLEC10	2.143808159	2.279813805	0.966623703	2.3585329	0.018347333	0.035957545
RBM48	5.899371123	1.316998635	0.558467802	2.358235568	0.018362037	0.035981085
TXNRD3	3.29126412	-1.937350056	0.821565276	-2.358120666	0.018367721	0.035986949
DKK3	1.736721861	2.707988844	1.148430871	2.35799029	0.018374174	0.035994314
KCNMB4	8.271455087	1.241227602	0.526604415	2.35703987	0.018421269	0.036081284
KPNA3	37.6504602	0.606965793	0.257645283	2.355819546	0.018481893	0.036194724
H1-6	3.526628975	1.748667212	0.742427878	2.355336139	0.018505957	0.03623654
NIPAL2	5.019903198	1.40321198	0.596067559	2.354115667	0.018566833	0.036350415
XPO7	66.83894698	0.52855131	0.224539199	2.35393781	0.018575719	0.036362486
SF3B1	254.223047	0.35718967	0.151808394	2.352898027	0.018627742	0.036458983
EXTL3	14.1245153	0.944361266	0.401415006	2.352580879	0.018643635	0.036484747
KCTD21	2.729092397	1.945166487	0.8270126	2.352039723	0.018670781	0.036532522
STEEP1	10.70917612	0.977899727	0.415937957	2.351071139	0.018719455	0.036622399
ZNF75D	126.5832252	-0.420386267	0.178835838	-2.350682455	0.018739018	0.036655307
LOC105607817	5.231065923	1.399611205	0.595440087	2.350549177	0.01874573	0.036663072
RUSC2	3.64587625	1.888990358	0.803761515	2.350187616	0.01876395	0.036693338
MXRA8	1.303662195	3.097582119	1.318137928	2.349968128	0.018775019	0.036709611
LOC114116464	2.274477025	2.26699964	0.964753451	2.349822783	0.018782351	0.036718576
DUSP23	13.8603936	1.089576665	0.463719123	2.349647905	0.018791177	0.036730458
POLD1	8.17513737	-1.151171618	0.489970946	-2.349469141	0.018800202	0.036742726
FYTTD1	18.80472707	-0.816045468	0.347436656	-2.348760424	0.018836022	0.036807349
ZBTB49	6.025887903	-1.23722379	0.526776844	-2.348667761	0.01884071	0.036811128
COMMD9	28.67060777	-0.782714749	0.333301914	-2.348365602	0.018856003	0.036835623
PPL	10.51529785	-0.931548823	0.396883976	-2.347156546	0.018917305	0.036949977
PLEKHA6	50.84607558	0.467745408	0.199288558	2.347076078	0.018921391	0.036952559
KRT27	1.015623219	3.34795312	1.426544073	2.346897782	0.018930447	0.036964844
LOC101105179	207.903485	-0.489441704	0.208590907	-2.34641917	0.018954777	0.037006946
PM20D2	5.856517381	1.240884986	0.528865153	2.346316405	0.018960004	0.037011745
CLDN15	23.46799083	-0.803648177	0.342660506	-2.345318948	0.019010809	0.037105502
DCAF16	20.97579108	-0.702658105	0.299697172	-2.344560348	0.019049527	0.037172489
SSH1	20.02047951	0.762282799	0.32513141	2.344537548	0.019050692	0.037172489
ABHD12	9.643555847	-1.029767281	0.43929702	-2.344125349	0.019071761	0.037208167
SYNRG	27.64937022	0.632238363	0.269818135	2.343201892	0.019119034	0.037294952
LOC121818311	3.393969139	1.730196099	0.738495226	2.342867005	0.019136203	0.037322996
SNX11	10.45709831	0.927947295	0.396085482	2.342795524	0.019139869	0.0373247
ACP5	36.1354798	0.612881377	0.261610218	2.342727215	0.019143374	0.037326088
RANBP9	13.83084064	0.902204148	0.385158674	2.342421991	0.019159039	0.037351183
APPBP2	76.12847616	0.496878542	0.212184263	2.34173135	0.019194526	0.037409453
NTPCR	34.93142276	-0.785290541	0.335341298	-2.341765075	0.019192791	0.037409453

LOC101116336	2.604525587	2.143569268	0.915487965	2.34144997	0.019209	0.037432205
EGFL7	2.042873589	2.466627233	1.053590155	2.341163896	0.019223726	0.037449981
RAB2A	150.6958865	0.399065902	0.170454358	2.341189205	0.019222423	0.037449981
LOC105608474	3.963063625	-1.625936798	0.694515711	-2.341108736	0.019226567	0.037450057
MANEA	15.25160374	0.955528191	0.40818433	2.340923254	0.019236121	0.037463207
ENC1	9.818538419	-0.993608366	0.424553023	-2.340363424	0.019264983	0.037513952
LOC132658845	6.036759353	1.364181505	0.582925903	2.340231404	0.019271795	0.03752175
LOC105606506	1.218394637	2.963378077	1.266587005	2.339656151	0.019301501	0.037574113
VASH2	1.608768243	-2.964507624	1.26721462	-2.339388748	0.019315323	0.037595546
LOC101114378	11.38673299	-0.95941104	0.410170322	-2.339055236	0.019332575	0.037623646
MYOZ3	1.445406044	3.146780038	1.346412525	2.337158917	0.019430922	0.037809537
SH3YL1	16.05631454	-0.817805511	0.349926494	-2.337078002	0.019435129	0.037812217
COPG2	20.76819481	-0.661708508	0.283203766	-2.336510269	0.019464663	0.037864166
ZNF219	33.75448773	-0.637134715	0.272703238	-2.336366521	0.019472147	0.037873213
TPP1	15.8816329	-0.824454384	0.352888382	-2.336303562	0.019475426	0.037874079
TMEM223	14.76867653	0.923388143	0.395486736	2.33481444	0.019553117	0.038019633
ALPK1	4.808759925	1.387563785	0.594359543	2.334552883	0.01956679	0.038040687
MTCP1	5.624426659	1.321055847	0.566209633	2.333156784	0.019639918	0.038177304
ALKBH5	31.24040341	-0.579701969	0.248510582	-2.332705369	0.019663614	0.038217808
PADI1	1.886350814	-3.084193025	1.322377531	-2.332309007	0.019684441	0.038247164
TMEM127	4.337095889	1.509918294	0.647382937	2.332341813	0.019682717	0.038247164
MTERF1	5.004720959	1.442083783	0.618415153	2.331902404	0.019705826	0.03828315
JDP2	18.02475892	-0.746233253	0.320038625	-2.331697468	0.019716612	0.038298538
OSGEPL1	16.79135314	0.823568787	0.353245682	2.331433416	0.019730517	0.038319979
BICDL1	3.377480322	1.795465538	0.770265623	2.330969322	0.019754977	0.038359212
GPRC5A	9.696711127	-0.998301541	0.428282584	-2.330941249	0.019756458	0.038359212
LOC132659203	1.873831437	2.442557063	1.048212255	2.330212275	0.019794935	0.038428336
NCAPD3	10.84829154	-0.906770787	0.389369508	-2.328818174	0.0198687	0.038565938
GSK3A	43.46032888	-0.573639901	0.246339522	-2.328655577	0.019877319	0.038577065
EEF1AKMT3	4.488601621	-1.474590752	0.633330643	-2.328311077	0.019895592	0.038606922
TMCO1	247.0116956	0.38660203	0.166123449	2.327197226	0.019954771	0.038716136
NDUFA6	38.45964057	-0.561014309	0.241136891	-2.326538702	0.01998983	0.03877853
LOC121817573	14.00859168	0.874360451	0.37592964	2.325861964	0.020025916	0.038842895
LOC114114086	4.801870289	1.531027545	0.658371786	2.325475634	0.020046541	0.038877259
CAPRIN2	1.939566558	2.413559095	1.038027772	2.325139231	0.020064517	0.038906474
STOML1	2.404117095	-2.201021525	0.946829982	-2.324621702	0.020092197	0.038954498
PDE3B	23.09011465	0.717420672	0.308630883	2.324526521	0.020097292	0.038958724
PTK2B	4.228118337	1.539977978	0.662506944	2.324470699	0.02010028	0.038958867
LOC105605573	9.464512959	-1.075111979	0.462657055	-2.323777337	0.020137431	0.039025214
PRR15L	55.68342061	-0.452694776	0.194875686	-2.322992602	0.02017955	0.039101169
WDR74	17.70670046	-0.786980726	0.338804105	-2.322819337	0.02018886	0.039113538
KLHL5	20.41533766	0.72331854	0.311468853	2.322282097	0.020217751	0.039163835

CENPO	11.31651217	-0.948133219	0.408392369	-2.32162325	0.020253231	0.039225905
IQGAP3	1.569565832	-2.939052623	1.265971885	-2.321578115	0.020255663	0.039225905
OXR1	45.34613553	0.491572934	0.21175043	2.321473131	0.020261323	0.03923118
OFD1	36.39754246	0.584834729	0.251938431	2.321339888	0.020268507	0.039239407
KARS1	166.5592555	-0.497016056	0.21416254	-2.320742261	0.020300758	0.039296153
GPSM2	14.00691197	-0.979703504	0.422318474	-2.319821564	0.020350532	0.039386796
ARMC2	4.243377308	1.502165768	0.647614864	2.319535656	0.02036601	0.03940534
DCTN2	89.29193672	-0.552811748	0.238326772	-2.319553707	0.020365033	0.03940534
ADAP1	5.90631214	-1.406649923	0.606544351	-2.3191213	0.02038846	0.039443068
CFAP68	2.098894572	-2.359708764	1.017826462	-2.318380247	0.020428665	0.039515128
CLASP1	10.07148283	1.008580239	0.43518344	2.317597926	0.020471183	0.039585913
WDFY2	24.18760103	0.62783749	0.270896017	2.31763278	0.020469287	0.039585913
CBX4	17.44344106	-0.819919197	0.354017609	-2.316040719	0.020556046	0.039744265
SDE2	49.69353213	0.490447718	0.211863145	2.314927013	0.020616927	0.039856211
NUDT9	7.701561728	1.227152362	0.53051069	2.313152938	0.020714232	0.040038529
LOC101117269	279.9770626	-0.373811982	0.161632525	-2.312727483	0.020737627	0.040077953
GPR108	66.26937712	0.48379945	0.20921262	2.312477375	0.020751391	0.040098755
TARS2	36.4196594	-0.791661698	0.342391128	-2.312155993	0.020769088	0.040127152
LOC121816000	13.01748151	-0.803960296	0.347800001	-2.311559211	0.020801987	0.040184904
LTV1	27.71932518	-0.616882559	0.266875385	-2.311500399	0.020805231	0.040185364
PCMT1	42.24366965	0.532759206	0.230496595	2.311353905	0.020813315	0.040195169
WSCD1	2.578752137	2.08712715	0.90312566	2.311004152	0.020832626	0.04022665
PSMA1	89.34185442	0.482556739	0.20882047	2.310868947	0.020840095	0.04023526
FIBIN	0.908212073	3.245968612	1.404846305	2.310550699	0.020857685	0.040263405
NUDC	30.50109319	-0.667795441	0.289060398	-2.310228053	0.020875532	0.040292037
ATMIN	6.011181995	-1.276077669	0.552397786	-2.310070209	0.020884267	0.040303078
BORA	3.656706735	-1.618834444	0.700818079	-2.309921065	0.020892525	0.040307374
DLG1	55.67962872	-0.464759248	0.201199249	-2.309945249	0.020891186	0.040307374
SLC2A13	5.489881068	1.366906507	0.591876012	2.309447382	0.020918768	0.04035218
IL7	4.651696286	1.612750783	0.698353136	2.309362841	0.020923455	0.040355397
CAMLG	50.11641968	-0.563335253	0.244032072	-2.308447608	0.020974254	0.040447536
MNT	3.772367484	1.625143963	0.704222266	2.307714542	0.021015019	0.040520303
VCPKMT	32.46766749	-0.579993539	0.251482836	-2.306294729	0.021094169	0.040667052
SERPINC1	3.506540271	-1.701001323	0.737685616	-2.305862126	0.021118337	0.040707773
PLEKHM1	7.649986425	1.137384212	0.493504279	2.304709928	0.021182824	0.040820304
SH3GL1	67.84406038	0.508825991	0.220776343	2.304712477	0.021182682	0.040820304
ANXA5	66.8840746	0.65195109	0.28295608	2.304071674	0.02121862	0.040883391
LOC101106452	98.70522772	-0.494263936	0.2145247	-2.303995467	0.021222898	0.040885739
CCN1	34.67598178	-1.446434556	0.627918304	-2.303539404	0.021248513	0.040917393
FUNDC1	47.82980057	-0.525905911	0.228294012	-2.303634277	0.021243182	0.040917393
LRIG3	2.853881032	2.055919714	0.892484587	2.303591281	0.021245598	0.040917393
FLVCR1	18.76533724	0.731370794	0.317579118	2.302956186	0.021281308	0.040974642

WDFY3	27.8170476	0.600159186	0.260671154	2.302361332	0.021314804	0.041033223
SH3GLB1	52.60442499	-0.487952601	0.211954056	-2.302162136	0.02132603	0.041048923
FTCDNL1	12.63793434	0.869178941	0.377655043	2.301515518	0.021362509	0.041113218
COTL1	180.5754235	0.386714949	0.168037969	2.301354573	0.021371597	0.041124787
CFD	21.77730951	0.695065445	0.302033809	2.301283583	0.021375607	0.041126582
SFXN2	17.33874882	-0.884632376	0.384574895	-2.300286337	0.021432003	0.041229153
LOC105607506	17.17014003	-0.81030277	0.352336212	-2.299799859	0.021459561	0.041276227
DYNC1H1	187.7953718	0.411470678	0.178930792	2.299607982	0.021470439	0.041291208
CLN5	33.3393429	0.563643547	0.245121297	2.299447474	0.021479543	0.041302772
DOLPP1	25.62931746	-0.882400722	0.383788496	-2.299184919	0.021494441	0.041325475
ZNF331	14.50394367	0.833665788	0.362735198	2.298276517	0.021546056	0.041418752
GLE1	5.707759909	-1.207871021	0.525715796	-2.297574148	0.021586038	0.041489645
CEP164	63.25410612	1.338212495	0.582547357	2.297173748	0.02160886	0.041527538
RFC2	30.53085202	-0.616310706	0.2684375	-2.295918814	0.021680524	0.04165927
LOC132658420	1.242003229	2.946791567	1.28474197	2.293683584	0.02180868	0.0418995
LOC101102155	7.852360412	1.088761668	0.474716274	2.293499774	0.021819248	0.041907756
LOC132657591	1.259778241	3.032914342	1.322369465	2.293545353	0.021816627	0.041907756
METAP2	422.8959818	0.530738963	0.231509897	2.292510899	0.021876179	0.042011065
SMARCC2	161.172736	-0.344937749	0.150486488	-2.292150962	0.021896933	0.042041406
VIPAS39	17.52634497	-0.868596319	0.378947595	-2.292127806	0.021898269	0.042041406
LOC105601929	2.464748853	-2.280876117	0.995209923	-2.291854277	0.021914053	0.042065667
NOD2	1.335714677	3.066323684	1.338025287	2.291678425	0.021924206	0.042079113
CLCN7	15.64851927	0.92537614	0.403816623	2.291575152	0.021930171	0.042084517
CBLN3	2.101954934	2.291699962	1.000364709	2.290864462	0.021971254	0.042157304
MAP1LC3C	0.944237503	3.278958606	1.432162009	2.289516539	0.022049358	0.042301094
CTSH	15.28013787	0.771101241	0.336821682	2.289345619	0.022059279	0.042314054
EFNA4	7.925093216	-1.118681489	0.488688612	-2.289149902	0.022070644	0.042329779
TANC2	4.037796311	1.535995689	0.671133207	2.288659947	0.022099118	0.042378308
GSPT2	5.609744178	1.303346265	0.569524786	2.288480319	0.022109565	0.04239226
LOC101116755	87.00591048	-0.509992191	0.223026854	-2.286685135	0.022214208	0.042586791
COMP	2.677108744	2.006723385	0.877740371	2.286237995	0.022240339	0.042630772
LOC132660064	12.28579101	0.972855751	0.425774005	2.284911102	0.022318041	0.042773578
GLTP	9.198979384	-1.040153474	0.45542681	-2.283909184	0.022376869	0.042880176
SIT1	2.561698658	1.967864173	0.861675283	2.28376537	0.022385324	0.042890229
METTL8	13.81797688	0.812906944	0.356025428	2.283283387	0.022413681	0.042936996
RBM7	5.461678776	1.4734804	0.645345889	2.283241322	0.022416157	0.042936996
LOC114110261	2.656719166	-1.996783134	0.874637392	-2.282983958	0.022431313	0.04295987
PPIL1	89.17620398	0.464171805	0.203360737	2.282504542	0.02245957	0.043007823
LOC132659835	3.559809608	1.660601511	0.727698935	2.281989748	0.022489946	0.043059821
ADORA2B	1.226108357	3.004022933	1.3165092	2.281809298	0.022500602	0.043074053
FAM135A	45.68473138	0.536386979	0.235098471	2.281541762	0.022516408	0.04309814
FBXO25	59.09895516	0.551508652	0.241759005	2.28123313	0.022534655	0.04312689

PAK3	7.962087022	1.134325816	0.497592551	2.27962781	0.022629772	0.043302725
MIS12	11.21250307	1.076627763	0.472295516	2.279563803	0.022633572	0.043303796
TOMM40	41.44761135	-0.569264108	0.249731306	-2.279506392	0.02263698	0.043304119
NEK5	2.645073656	2.434944596	1.068345568	2.279173209	0.022656771	0.043335776
LOC114111468	1.882517151	2.478950299	1.087854466	2.278751779	0.022681825	0.043377489
SPG21	7.852170888	1.146149462	0.503248495	2.277502019	0.022756264	0.043513623
LOC101116273	28.36759859	0.620475412	0.272568346	2.276403043	0.022821898	0.043632883
ARL5A	88.62999215	0.433715527	0.19060099	2.275515609	0.022875017	0.043728187
PPP1R35	36.2838075	-0.514447679	0.226117721	-2.27513207	0.022898008	0.043765877
CHRM1	1.862913436	2.467779045	1.084774951	2.274922593	0.022910574	0.043783633
TMEM9	6.888385439	-1.110240299	0.488174733	-2.274268259	0.022949862	0.043852446
ITGA7	11.24905224	-0.925359882	0.407280528	-2.272045478	0.023083764	0.044098951
LOC114110614	7.631670461	-1.190052702	0.523786823	-2.27201726	0.023085468	0.044098951
KIFC2	5.889857758	-1.314317473	0.578590139	-2.271586369	0.023111505	0.04414238
LOC114108678	1.436002339	3.127005118	1.377080881	2.270749061	0.023162173	0.044232834
C6	1.981396437	2.578862584	1.135838138	2.270449017	0.023180353	0.044261229
LOC132659050	4.037056338	-1.527145766	0.672671753	-2.270268907	0.023191272	0.044275753
CYRIB	4.702882614	1.437327434	0.633215072	2.269888222	0.023214366	0.044313513
NECAP1	31.55815565	0.618141055	0.272411903	2.269141135	0.023259745	0.044393795
ZNF207	192.992082	-0.316315547	0.139431952	-2.268601581	0.023292565	0.04445009
TERF1	8.429412461	-1.034951216	0.456324228	-2.268017239	0.023328156	0.044511653
TNFAIP8L1	8.524465102	-1.034932501	0.456405747	-2.267571141	0.023355358	0.044557197
ADAM12	1.954159731	2.437646107	1.075275173	2.266997479	0.02339038	0.044617642
UBA52	225.9916821	0.373232132	0.164702302	2.266101493	0.02344517	0.044715773
LOC114118729	8.021594059	-1.046180658	0.461734078	-2.265764449	0.023465809	0.044748752
CDC25A	29.08872379	0.6533316	0.288401819	2.265351869	0.023491095	0.044790582
RPL14	302.7518899	-0.419618796	0.18526284	-2.264991713	0.023513188	0.044826312
TICAM2	3.124264397	1.770838796	0.781952639	2.264636893	0.023534971	0.044861441
RARS1	97.45150921	-0.373625204	0.164991644	-2.264509861	0.023542774	0.044869915
FKBP5	110.8834316	0.364376909	0.160987249	2.263389872	0.023611667	0.044994801
CHST14	3.541013853	-1.567262605	0.692686159	-2.262586864	0.023661169	0.045072238
CNNM2	2.628589276	2.025622003	0.895276188	2.262566602	0.023662419	0.045072238
PRKD3	11.21836171	-0.986735613	0.436110111	-2.262583664	0.023661366	0.045072238
SH3BP2	22.18384508	-0.7782921	0.344061508	-2.262072571	0.02369292	0.045123905
NCOA6	117.5802459	0.42047497	0.1859006	2.261826865	0.023708102	0.045146388
GTF2H5	14.21883422	-0.862839621	0.381536573	-2.261486006	0.023729178	0.045180085
BLZF1	10.84901311	1.178274239	0.521045749	2.261364269	0.023736709	0.045187987
SOCS1	2.067586105	2.219958924	0.981803882	2.261102206	0.023752929	0.045212425
POLQ	20.36004359	0.74366657	0.328987233	2.260472431	0.023791946	0.045280243
PIGC	54.08573974	-0.477403524	0.211309446	-2.259262582	0.023867056	0.045410563
ZBTB33	55.67041444	-0.497302965	0.220117632	-2.259260017	0.023867215	0.045410563
ZKSCAN8	22.12137752	-0.646869451	0.286340572	-2.259091147	0.023877716	0.045424075

PXYLP1	9.563626384	-0.935624865	0.414229551	-2.258711053	0.023901364	0.045462592
LOC100037663	50.93010288	-0.508177141	0.225077841	-2.257783971	0.023959131	0.045565985
ADRM1	29.06357531	-0.886319802	0.392738719	-2.256767055	0.024022634	0.045680258
CNOT6L	136.8792328	0.356391523	0.157957339	2.256251749	0.024054869	0.045735047
TRAPPC9	137.7419169	0.361236454	0.160110574	2.256168627	0.024060073	0.045738434
LOC105615393	17.85177663	-0.815377224	0.361429523	-2.255978473	0.024071979	0.045754561
CLGN	1.749618413	2.44249742	1.083172949	2.254946842	0.024136666	0.04587099
MDM4	94.26775961	-0.40931533	0.18156501	-2.254373413	0.024172686	0.045932915
CASKIN2	4.302180967	-1.537503418	0.68210643	-2.25405208	0.024192892	0.045964775
LOC132657201	1.608776257	2.63056326	1.167184392	2.253768365	0.024210744	0.045992155
PSMG4	108.1516592	-0.428649963	0.19020366	-2.253636774	0.024219028	0.046001354
FMR1	117.559051	0.418617783	0.185782076	2.25327325	0.024241925	0.046038303
SH3TC1	6.349325503	1.218113071	0.540633054	2.253123557	0.02425136	0.046049676
CEPT1	101.504986	0.370974558	0.164715922	2.252208251	0.024309115	0.04614684
TSPAN3	253.1752242	-0.612226189	0.27183435	-2.25220319	0.024309435	0.04614684
DNAJB6	151.7509917	0.346583331	0.153893908	2.252092597	0.024316421	0.046153548
CDC6	13.11940968	0.781569925	0.347067898	2.251922258	0.024327185	0.046167407
PSMB3	24.10952714	0.838373452	0.372301373	2.25186774	0.024330631	0.046167407
ZNRF2	37.8526394	0.524190635	0.232846294	2.251230314	0.024370954	0.046237355
LIPE	5.040130852	-1.305310133	0.579841302	-2.251150663	0.024375997	0.046240359
MAPK3	23.82606308	-0.691511176	0.307246736	-2.250670539	0.024406412	0.046291486
VPS52	5.406367747	-1.404823778	0.624270559	-2.250344436	0.024427089	0.046324131
CCDC50	57.44430144	-0.417402203	0.185521156	-2.249890047	0.024455926	0.046366798
MCC	3.008480404	1.791907597	0.796445642	2.249880597	0.024456526	0.046366798
ARPC1A	56.80744685	-0.538526358	0.239370417	-2.249761543	0.024464086	0.046374554
VPS26A	73.29452814	-0.425944762	0.189338494	-2.249646928	0.024471367	0.046381777
SMARCD1	5.122233465	1.305753806	0.580451881	2.249547033	0.024477714	0.046387229
FAM184B	2.813283652	2.118802822	0.941986814	2.249291382	0.024493964	0.046411443
MED23	24.04383063	0.609584071	0.27102969	2.249141306	0.024503507	0.046422945
POLR1H	4.992016922	-1.304472619	0.580006594	-2.249065154	0.024508351	0.046425542
HIPK1	22.5406676	0.738677174	0.328454912	2.248945429	0.024515968	0.04643339
SRP14	90.05586221	0.371947038	0.165396623	2.24881882	0.024524025	0.046442069
CCDC112	16.98341545	-0.726979956	0.323301395	-2.24861373	0.024537082	0.046460212
LOC132659174	2.112558531	-2.278991875	1.013992718	-2.247542644	0.024605368	0.046582911
KLHL32	2.071427289	2.283102826	1.016005713	2.247135815	0.024631349	0.046625493
CSRNP3	1.486760178	2.620670059	1.166332217	2.246932752	0.024644325	0.046636847
PCGF2	38.10393933	0.597792768	0.266043358	2.246974976	0.024641626	0.046636847
BAG4	42.32019793	0.490049912	0.218119777	2.246700958	0.024659145	0.046658286
MALSU1	8.509164675	-0.999071606	0.444912018	-2.245548704	0.024732929	0.046791271
WDR31	4.493669437	-1.370076777	0.610249093	-2.245110713	0.024761026	0.046837797
GINS2	2.490574832	-1.912479799	0.852136138	-2.244335984	0.024810792	0.046925293
ATF6	90.20438982	0.413637446	0.184437422	2.242698054	0.024916292	0.047118161

GATM	42.61664304	-0.580485998	0.258879825	-2.242299101	0.024942048	0.047160194
DDX19A	28.19766602	-0.61389615	0.273819515	-2.241973699	0.024963072	0.047186596
PFAS	6.272027196	-1.277571362	0.569835912	-2.241998678	0.024961458	0.047186596
FANCL	14.28156091	-0.792667041	0.353617605	-2.241593831	0.024987635	0.047219671
MTRR	15.12649321	-0.741437054	0.330758969	-2.24162343	0.02498572	0.047219671
BRD8	75.98456006	0.417690557	0.186395927	2.240878132	0.02503397	0.04729699
LTBP2	4.466617628	-1.447142164	0.645799817	-2.240852544	0.025035628	0.04729699
METRNL	4.103887242	-1.413205042	0.630670595	-2.240797418	0.0250392	0.047297054
COPS4	98.54488566	-0.392879168	0.175358307	-2.240436592	0.025062593	0.047334551
LOC101113728	720.188376	-0.954201242	0.425962829	-2.240104481	0.025084141	0.047368554
LOC114109646	5.449799913	1.404911358	0.627247933	2.239802293	0.025103761	0.047398908
PMM2	24.87449891	-0.619753091	0.276731595	-2.239545832	0.025120423	0.047423668
DAB2	20.19007869	-0.653818247	0.291954966	-2.239448967	0.025126718	0.047428854
PIK3C2A	26.82842136	-0.61627759	0.275202555	-2.239359989	0.025132503	0.047433074
ZFPL1	4.926836694	-1.389074879	0.620472172	-2.238738402	0.025172942	0.047502689
NUDT5	13.5288548	-0.809507416	0.361708248	-2.238012045	0.02522027	0.04758528
RRAGD	12.53518512	0.913108585	0.40807081	2.23762289	0.025245658	0.047626459
UROD	8.190342734	-1.023350385	0.457357761	-2.237527099	0.02525191	0.047631531
NUDCD2	31.64488526	0.537052103	0.24003402	2.237399944	0.025260212	0.047640468
ZNF169	66.99602581	-0.441587431	0.197381385	-2.237229365	0.025271353	0.047654755
CPQ	0.951422013	3.24208121	1.449685347	2.23640338	0.02532536	0.047749861
MMS22L	4.888185355	-1.300656503	0.581612258	-2.23629486	0.025332463	0.047756516
B4GALT4	19.21953926	-0.813850169	0.363942158	-2.236207463	0.025338185	0.047760566
COIL	83.36969473	0.445791378	0.199395397	2.235715494	0.025370413	0.047803782
GEMIN7	14.16354784	0.766763922	0.342959554	2.23572696	0.025369662	0.047803782
PRELID2	262.3530641	0.336310074	0.150427616	2.235693712	0.025371841	0.047803782
KIF15	31.64940919	-0.617661175	0.276425957	-2.234454327	0.025453195	0.047950303
TRERF1	4.703021244	1.365062637	0.611097336	2.233789212	0.025496946	0.048025955
SCLY	25.42048341	-0.75134575	0.336403252	-2.233467557	0.025518127	0.04805908
ARAP2	65.69422641	0.417760582	0.187114125	2.232651227	0.025571953	0.048148149
ARHGEF3	10.91529563	0.900289776	0.403239821	2.232641046	0.025572625	0.048148149
TTC4	98.12234613	-0.420281051	0.188291298	-2.232078988	0.025609743	0.048211243
SGMS1	108.2685249	0.381678725	0.171003815	2.231989532	0.025615655	0.048215581
NIT1	3.401046354	-1.5706336	0.703725419	-2.231884137	0.025622622	0.048221904
LOC132659917	2.846189132	-1.963252693	0.880002375	-2.230962948	0.025683583	0.048329829
FOXN3	82.39081439	0.381024531	0.170826223	2.230480332	0.025715572	0.048369594
FOXP2	2.77891652	1.84498969	0.827147346	2.230545379	0.025711258	0.048369594
ZHX3	30.86217841	-0.655031694	0.29367094	-2.230495444	0.02571457	0.048369594
ITGB4	20.44587532	-0.725523549	0.325361531	-2.229899605	0.025754109	0.048435263
SPTLC1	174.8292795	-0.358443121	0.160753039	-2.22977509	0.025762378	0.048443999
BRCC3	54.65627174	-0.473345189	0.212310176	-2.229498361	0.025780764	0.048471753
CCN3	7.147837722	-1.117185995	0.501292008	-2.228613218	0.025839651	0.048558106

CPSF4	8.38701766	-1.161940836	0.52137946	-2.228589588	0.025841224	0.048558106
PDCD5	125.3468736	-0.34172044	0.153331102	-2.228643988	0.025837602	0.048558106
USE1	4.866966512	1.331089562	0.597274077	2.228607624	0.025840023	0.048558106
LOC105605606	2.69811124	-2.126151479	0.954134083	-2.228357121	0.02585671	0.048580375
CRELD1	7.798828444	1.0672909	0.47906981	2.227840031	0.025891184	0.048638309
NGLY1	9.028805674	0.964928816	0.433265049	2.227109753	0.02593994	0.048723051
PCNP	27.64254465	-0.645579295	0.289892584	-2.226960369	0.025949923	0.048734954
HEPACAM2	2.639236957	1.999753796	0.898056464	2.22675731	0.025963498	0.048753598
POLR1B	41.62548488	-0.559729129	0.251423342	-2.226241708	0.025997996	0.04881152
VSTM5	2.104209656	2.138663919	0.960784637	2.225955575	0.026017157	0.048840635
SEMA4G	11.68445733	0.958508873	0.430787609	2.225014958	0.026080234	0.048952171
ALAD	21.7994287	-0.647949075	0.291276735	-2.224513659	0.026113905	0.049001607
MTFR1	40.17011803	0.530471226	0.238462477	2.224548003	0.026111597	0.049001607
PSMD5	8.135821882	-1.17842242	0.529944859	-2.223669877	0.026170663	0.049101219
LOC132659551	2.15515395	2.155865467	0.969626026	2.223398927	0.026188912	0.049128561
HEBP2	38.94792112	-0.485442063	0.218350891	-2.223219973	0.026200971	0.049144285
MAP3K15	5.999270907	-1.331970109	0.599230556	-2.222800717	0.026229241	0.049190407
LOC121816191	1.935244991	-2.266755767	1.019918915	-2.222486253	0.026250462	0.049216394
SLC46A2	1.135051573	2.905825832	1.307455423	2.222504707	0.026249216	0.049216394
MSL2	54.3424442	-0.442673235	0.199223539	-2.221992628	0.026283804	0.049271995
GET1	8.923105238	-0.995101818	0.447988904	-2.221264429	0.026333057	0.049357402
CAB39L	13.43587832	-0.826416487	0.372246593	-2.220078037	0.026413471	0.049501185
UBTD2	35.19482655	0.555328761	0.25020849	2.219464102	0.026455167	0.049572376
AURKAIP1	19.30039168	0.671310784	0.302595379	2.218509704	0.026520099	0.049687081
ENO4	5.402703165	1.352919839	0.610039288	2.217758538	0.026571301	0.049776034
ARHGAP24	42.56439302	0.567093602	0.255764779	2.217246658	0.026606241	0.049834503
NAPEPLD	34.76349441	0.591195104	0.266686027	2.216820693	0.026635347	0.04988203
GATB	5.017005094	-1.291875114	0.582785759	-2.216723889	0.026641965	0.049887435
TADA3	5.981319836	1.184391245	0.53450942	2.21584728	0.026701963	0.049992778
LOC101116807_1	11.89922085	0.886441644	0.400115723	2.215463162	0.02672829	0.05003506
FAR1	25.89612915	-0.612842585	0.276657044	-2.215170725	0.026748348	0.050065597
DMAP1	14.42506668	0.864048364	0.390102085	2.214928853	0.026764948	0.050089653
WDR24	6.139642223	1.185808567	0.535406963	2.214779875	0.026775177	0.050101781
CENPU	9.06547873	-0.950169134	0.429023481	-2.214725245	0.026778929	0.050101787
AIMP2	23.73990381	-0.642326109	0.290044058	-2.214581166	0.026788826	0.050110952
LOC132659459	7.265399587	1.06982071	0.483088318	2.214544776	0.026791326	0.050110952
RAB23	12.87503199	-0.848744683	0.383311105	-2.214244963	0.026811932	0.050142478
ACSM3	56.90784035	-0.615419074	0.277950501	-2.214131912	0.026819706	0.050149999
LOC105602361	2.419422749	2.185195146	0.986966882	2.214051136	0.026825261	0.05015337
LOC121817420	6.725559496	1.095466695	0.494796696	2.213973343	0.026830613	0.050156359
USP42	34.28777304	0.54912566	0.248040892	2.213851336	0.026839007	0.050165036
WNK2	6.989297174	-1.08612162	0.490829613	-2.212828224	0.026909491	0.050289744

LOC114114990	1.011733629	3.385454153	1.530147412	2.212501964	0.026932001	0.050324776
CACYBP	11.19282907	-0.893569597	0.403906253	-2.212319293	0.026944611	0.050341301
CEBPB	7.45966206	1.087298578	0.491526648	2.2120847	0.026960813	0.050364532
RIC1	72.57837566	0.402822591	0.182158532	2.211384706	0.027009208	0.050447886
BRK1	11.71646497	-0.883968215	0.399777405	-2.211151015	0.027025381	0.050471042
NLGN1	8.56721301	-1.262226401	0.570983942	-2.210616284	0.02706242	0.050533153
TMED10	406.7523533	0.3621269	0.163898605	2.209456884	0.027142878	0.050676311
DCUN1D5	41.64416498	0.531864957	0.240790939	2.208824636	0.027186841	0.050751301
RARS2	4.566671777	-1.370822954	0.620682739	-2.208572702	0.027204376	0.050776943
ZFP2	16.82875779	-0.697291953	0.315737572	-2.208454159	0.02721263	0.050785257
SETD3	31.03932998	-0.550760005	0.24941019	-2.208249813	0.027226864	0.050804727
DCAF8	63.92777259	-0.435510815	0.197278729	-2.207591346	0.027272773	0.050883289
CLMN	91.22308442	0.397967142	0.180367932	2.206418504	0.027354711	0.051016477
PRDX3	48.90822209	-0.629690341	0.28539192	-2.206405637	0.027355611	0.051016477
SRM	5.508331192	-1.34754064	0.610737844	-2.206414181	0.027355013	0.051016477
IGSF8	1.108847082	2.827296969	1.281634604	2.206008609	0.027383397	0.051061172
LOC101102490	5.924068759	-1.233908191	0.559431797	-2.205645439	0.027408835	0.051101477
STAM2	47.07254151	0.478616614	0.217063801	2.204958227	0.027457026	0.051184185
DENND2B	95.54815068	0.407960268	0.185107861	2.203905692	0.027530977	0.051314885
VPS18	40.64968737	0.599605438	0.272120305	2.20345717	0.027562543	0.051366557
ZNF771	2.500799949	-2.051950399	0.931381514	-2.203125538	0.027585902	0.051402922
PDHA1	114.7594816	0.357488756	0.1623358	2.202155999	0.027654291	0.051515993
USP39	43.81050179	0.491942819	0.223389088	2.202179274	0.027652647	0.051515993
RPL18	846.611452	-0.309638843	0.140665342	-2.20124473	0.027718703	0.051628789
ENTPD8	1.291981717	3.095237968	1.406185629	2.201158869	0.027724779	0.05163291
ANKS6	2.540298636	2.312061994	1.050903437	2.200070826	0.02780187	0.051769267
BBS10	11.18335491	-0.869455672	0.395204786	-2.200013011	0.027805972	0.051769692
LOC121819231	6.892409484	1.171105399	0.53233346	2.199947003	0.027810655	0.051771201
DSN1	6.184027581	-1.240298905	0.564075073	-2.198818852	0.027890805	0.051913174
CARM1	42.72706207	-0.566896017	0.25783766	-2.198654829	0.027902475	0.051927664
INTS4	16.85278622	0.759290607	0.345397135	2.198311827	0.027926892	0.05196587
LOC121820570	3.641455632	1.576123849	0.717047549	2.198074382	0.027943806	0.051990104
LOC101117358	13.62652195	-0.757740265	0.344787115	-2.197704707	0.027970156	0.052031886
CDKL5	5.965878822	1.223439457	0.556739536	2.197507772	0.027984202	0.052043529
KCMF1	31.37390567	0.687020218	0.312631788	2.197537948	0.027982049	0.052043529
NRN1	4.062438886	-1.466488777	0.667418967	-2.197253675	0.028002334	0.052070005
RET	8.921188058	-1.052023203	0.47881822	-2.19712442	0.028011562	0.052079918
SLC35B1	6.326164317	1.146103746	0.52170338	2.19684938	0.028031205	0.052101945
YTHDF2	97.90863478	-0.393834729	0.179271509	-2.19686179	0.028030318	0.052101945
SMARCAD1	32.19725035	0.564183787	0.25683508	2.196677286	0.028043502	0.052117554
LOC101109111	5.846733611	1.338777812	0.609740425	2.195652046	0.028116858	0.052246618
LOC132657415	1.440850785	2.542998207	1.158257561	2.195537756	0.028125046	0.052253688

MTRF1L	12.16429317	-0.806010958	0.367121249	-2.195489802	0.028128482	0.052253688
LOC101116576	21.95646407	0.720896264	0.328371363	2.195368854	0.028137149	0.052262526
MYBBP1A	52.54214901	0.474868139	0.216360498	2.194800544	0.028177908	0.052330961
INAFM2	2.091276165	2.327001307	1.060316109	2.194629778	0.028190166	0.052346451
DIXDC1	7.614872181	0.996347263	0.454145089	2.193896372	0.02824286	0.052437015
CDK17	11.26499779	1.060932596	0.483596929	2.193836502	0.028247166	0.052437724
CHURC1	63.11826533	-0.407838859	0.185956686	-2.193192767	0.028293493	0.052509141
LOC132660220	3.594223615	1.829828077	0.834318564	2.193200721	0.028292921	0.052509141
LOC101113523	45.22848389	-0.562184468	0.256401933	-2.192590597	0.028336889	0.052582377
RNF170	58.71291879	0.475520604	0.21689101	2.192440357	0.028347725	0.052595183
LOC114110432	7.781534136	1.011819204	0.461568304	2.192133199	0.02836989	0.052621698
TPST1	5.37884698	-1.315599004	0.600131938	-2.192182953	0.028366299	0.052621698
VPS50	29.84091815	-0.598695946	0.273168015	-2.191676602	0.028402866	0.052675553
SPHK1	3.370372802	1.60357274	0.732032528	2.190575799	0.028482504	0.05281592
ANKRD13D	3.947500809	1.575048909	0.719423796	2.189320005	0.028573589	0.052977472
SINHCAF	7.24918323	-1.277240352	0.583460072	-2.189079277	0.028591078	0.053002546
SSB	403.4140205	-0.360038045	0.164481222	-2.188930993	0.028601856	0.053015172
MZF1	9.287460146	0.945123781	0.431820325	2.18869684	0.028618882	0.053039375
TNIP2	6.141808547	-1.136177709	0.519187895	-2.188374807	0.028642312	0.053075439
SLC25A17	23.06031012	-0.641509454	0.293184727	-2.188072549	0.028664318	0.053108855
NELFA	34.19445925	-0.5116105	0.23384952	-2.187776569	0.028685881	0.05314144
ATP6V0E2	20.38989395	0.814902926	0.372616209	2.186976591	0.028744233	0.053242159
LOC105611576	3.081248298	-1.707137308	0.780919194	-2.186061404	0.028811113	0.053358645
ADAM9	23.10642834	-0.642674829	0.294029574	-2.185748937	0.028833978	0.053393593
MRGBP	22.03341325	-0.615956613	0.281825763	-2.185593708	0.028845343	0.053407239
GXYLT2	3.049927493	1.85003569	0.846656887	2.185106763	0.02888102	0.053465887
GBA1	16.11219633	0.813110065	0.372254816	2.184283537	0.02894142	0.053570283
TBC1D7	5.530732862	1.286602294	0.589053095	2.184187309	0.028948487	0.053575946
ZNF672	10.91667184	0.851348624	0.389812966	2.183992579	0.028962793	0.053595002
FKRP	4.982730211	-1.385060243	0.634374457	-2.183348066	0.029010187	0.053675272
KANSL2	5.616730135	-1.253793972	0.574620496	-2.181951361	0.029113123	0.05385827
ZBTB10	3.102896281	2.018133737	0.925183296	2.181333955	0.029158725	0.053935167
ADK	15.04821823	-0.755996416	0.346669583	-2.180740549	0.029202612	0.054008871
KREMEN1	2.469149374	2.057526131	0.943581517	2.180549422	0.02921676	0.054027561
ZFR	88.72343909	-0.35810249	0.164316775	-2.179342253	0.029306253	0.054185554
XRN1	139.1811453	0.416241654	0.191027034	2.178967262	0.0293341	0.054229541
LOC101110974	1.473518517	2.641624809	1.212437189	2.178772504	0.029348573	0.054248793
LOC121817886	10.59830374	0.893697937	0.410260297	2.178368087	0.029378644	0.054296869
POU2F1	64.27173893	-0.446848915	0.205166689	-2.177979852	0.029407537	0.054342754
SNURF	2.410702983	-1.997513732	0.91778851	-2.176442297	0.029522204	0.054547108
PSEN1	25.7560714	-0.643300485	0.295602682	-2.176233589	0.029537799	0.054568378
PLCD1	2.072354177	2.257234828	1.037540484	2.175563135	0.029587942	0.05465346

IQCC	13.37520831	-0.88846769	0.408456843	-2.175181311	0.029616532	0.054698711
DENND2C	2.035850333	2.548847336	1.172000099	2.174784232	0.029646289	0.054746104
LOC132658201	10.21104303	-0.893934981	0.411085774	-2.174570459	0.02966232	0.054768141
HMOX1	9.383608101	-0.947657808	0.436197023	-2.172545334	0.029814554	0.05504162
ELOVL1	53.30527682	-0.517246108	0.238150909	-2.171925822	0.029861258	0.055120229
TM7SF3	12.14659776	0.852242413	0.392407665	2.171829167	0.029868551	0.055126077
VAMP3	35.94361698	-0.499135046	0.229874072	-2.171341215	0.029905389	0.055186446
TMEM123	74.98062722	-0.423381512	0.195040726	-2.170733884	0.029951294	0.055263528
MYO1C	5.681098621	-1.257894867	0.579641704	-2.17012485	0.029997389	0.055340939
TSPAN1	83.4884346	-1.220054721	0.562579735	-2.168678759	0.030107081	0.05553564
PRPSAP1	16.25987373	-0.782058338	0.360668079	-2.168360285	0.030131285	0.055572617
UXS1	4.970185647	-1.391277283	0.641673905	-2.168199878	0.030143482	0.055587442
CHMP2B	44.72875573	0.523705294	0.241546825	2.168131556	0.030148679	0.055589355
HGS	29.28356963	-0.592219916	0.273161372	-2.168022188	0.030156998	0.055597026
GPR155	10.25700879	1.231551951	0.568532012	2.166196318	0.030296188	0.055845932
VPS35L	25.86720128	-0.575572724	0.265790148	-2.165515641	0.030348219	0.055934128
CEP44	25.72020906	0.698881872	0.323022062	2.163573184	0.030497121	0.056193069
PTPRH	3.167823785	1.725933906	0.797713065	2.163602404	0.030494877	0.056193069
EVI5L	2.78670178	1.871726067	0.865140765	2.16349309	0.030503275	0.05619666
ZBTB18	17.80435898	-0.697546514	0.32248498	-2.163035668	0.030538436	0.056253685
IMPDH1	8.307410943	-1.059037538	0.489723534	-2.16252123	0.030578023	0.056318844
LOC121816084	25.7056465	-0.797436518	0.368768746	-2.16242978	0.030585064	0.056324052
MFSD8	3.578921231	1.527552777	0.706514499	2.162096855	0.030610711	0.056363517
MFSD10	7.764270258	-1.104941498	0.511133841	-2.161745924	0.030637766	0.056405562
ASCC2	13.73056215	0.815957562	0.377549903	2.161191293	0.030680566	0.056476579
LOC105606290	15.22839646	-0.825479817	0.382031068	-2.160766198	0.030713404	0.056529243
RALB	98.88236868	0.393810181	0.182314113	2.160064155	0.030767703	0.056621386
SSBP2	13.90649696	0.746193767	0.34546662	2.159959091	0.030775837	0.056628556
AK3	32.55319194	0.611025134	0.282902961	2.15984001	0.030785057	0.056637725
LOC114114829	6.775403686	-1.051753999	0.48702821	-2.159534044	0.030808759	0.056673531
PDZK1	6.999600675	-1.316077313	0.609573186	-2.159014444	0.030849046	0.056739831
CSNK1D	31.08708632	-0.557401913	0.258186903	-2.158908554	0.030857262	0.056747133
LOC132659523	1.193956951	2.914231968	1.350176151	2.158408713	0.030896069	0.056810683
DCXR	5.158666991	1.221521578	0.566009682	2.158128414	0.030917849	0.056842912
NAP1L5	2.735260289	1.805489548	0.836875122	2.157418115	0.030973101	0.056936662
TMEM67	1.825402996	2.29499765	1.063958136	2.15703755	0.031002739	0.056983307
LOC121819061	3.510022091	-1.641587744	0.761180454	-2.156634128	0.031034183	0.057033259
CTTNBP2	2.807028859	1.711497584	0.793654259	2.156477539	0.031046396	0.057047859
ZMPSTE24	21.25252984	-0.647974797	0.300628849	-2.155397922	0.031130709	0.057194922
TRNT1	57.69179356	0.417335148	0.193638897	2.155223746	0.03114433	0.057212082
RPGR	12.45480744	-0.837654602	0.388683067	-2.15510958	0.03115326	0.057220623
LOC132660177	1.538096294	2.75192467	1.277050197	2.154907205	0.031169096	0.057241844

LYRM1	11.30572951	0.832371478	0.386298064	2.154738936	0.031182269	0.057258169
ABCF2	6.078090035	1.171263299	0.543648947	2.154447838	0.031205068	0.057292163
CTSC	46.58606782	-0.627558487	0.291402844	-2.153577084	0.031273353	0.057409646
BCL7B	12.00512903	0.835742737	0.388178119	2.152987752	0.031319641	0.057484868
BMI1	28.56875766	0.56523074	0.262540855	2.15292489	0.031324582	0.057484868
LOC105606856	3.672963464	1.496319974	0.69502815	2.15289118	0.031327232	0.057484868
SLC43A3	12.21093318	-0.838396855	0.38945374	-2.152750814	0.031338268	0.057497225
C1H3orf52	34.97134824	0.660306301	0.306747269	2.152606944	0.031349582	0.057503675
CEP120	57.33916302	0.444433008	0.206466607	2.152566048	0.031352799	0.057503675
WDR46	46.96080091	0.546344789	0.253813763	2.152542017	0.03135469	0.057503675
SEPHS1	53.57746422	0.453555315	0.210714376	2.152464983	0.03136075	0.057506899
C1GALT1C1	7.590729304	1.198190922	0.556911806	2.15149133	0.031437439	0.057639618
PIAS1	22.55246839	0.633897775	0.294669623	2.151215212	0.031459217	0.057671635
LOC114110349	3.732965439	1.517620763	0.705495248	2.151142431	0.031464959	0.057674252
LZTS1	2.879559683	-1.76094119	0.818779346	-2.15069078	0.031500615	0.05773169
TNPO1	127.9202101	-0.30192462	0.140442268	-2.149813048	0.031570006	0.057850932
RNF11	35.12929025	0.527319118	0.245311415	2.149590625	0.031587611	0.057875258
CDCA8	37.37872581	-0.56059399	0.260836761	-2.14921389	0.031617449	0.057921987
CCDC183	5.904003297	-1.166765336	0.543065501	-2.148479941	0.031675648	0.058020654
LOC101118455	11.93298513	-0.833841971	0.388224287	-2.14783567	0.031726812	0.058106409
CDK7	33.05218107	0.570224342	0.265502017	2.147721323	0.0317359	0.05811509
MBOAT1	48.67141296	0.443049456	0.206471106	2.145818195	0.031887486	0.05837668
MDN1	69.83991771	-0.463416065	0.215957724	-2.145864737	0.031883772	0.05837668
B4GAT1	19.1642333	-0.66608066	0.310443815	-2.145575552	0.031906858	0.058404144
KIAA1586	12.36786956	0.774985645	0.36129291	2.145034191	0.031950113	0.058475314
CBL	42.57252588	-0.518705443	0.241847843	-2.144759432	0.031972086	0.058507517
LOC114118052	5.444188418	1.298543228	0.605654323	2.144033615	0.032030193	0.058605827
SUOX	4.993480072	-1.221200748	0.569828814	-2.143101081	0.032104983	0.058734629
UBR2	40.3622718	0.484205985	0.225993044	2.142570305	0.032147618	0.058804579
TMEM64	10.67173935	0.972645007	0.454149806	2.141683192	0.032218984	0.058927058
LNP1	19.6370783	0.664233534	0.310170685	2.141509713	0.032232956	0.058944546
LOC105611784	5.602374211	-1.128895675	0.527408117	-2.140459423	0.032317657	0.059091354
NDUFB2	49.09502564	-0.515079618	0.240702047	-2.139905429	0.03236241	0.05916509
PUSL1	3.015761402	-1.762679801	0.823765405	-2.139783718	0.03237225	0.059174985
TMC4	7.825660693	1.051548204	0.491515508	2.139399847	0.032403299	0.059223643
FBXO15	10.97633609	-0.87283239	0.408013785	-2.1392228	0.032417629	0.059241731
DRAM1	9.135596153	0.964559295	0.451003462	2.138695989	0.032460298	0.059301255
PMVK	12.68189407	0.838553298	0.392073848	2.138763659	0.032454814	0.059301255
SORBS2	48.03900573	-0.485978127	0.227238062	-2.138629959	0.032465649	0.059301255
TKFC	11.55393398	0.998313919	0.466806869	2.138601603	0.032467948	0.059301255
DHRS12	3.512965601	-1.466163403	0.685590317	-2.138541584	0.032472813	0.059302038
DDX51	9.142930945	-1.066566593	0.498788862	-2.138312772	0.032491367	0.059327815

STAC	0.839697235	3.124667973	1.462243179	2.136900358	0.032606097	0.059529175
EPB41L4B	103.1380298	-0.410051962	0.191976056	-2.135953676	0.03268319	0.059661775
LOC121816364	1.422414121	-2.663662189	1.247255484	-2.135618743	0.032710502	0.059703479
KIZ	18.22084835	0.740077851	0.346557282	2.135513781	0.032719066	0.059710955
PKDCC	9.241720876	-1.19220721	0.558301098	-2.135419784	0.032726736	0.059716799
EMC10	827.7914625	0.375839458	0.176008124	2.135352899	0.032732195	0.059718607
LOC101122488	4.046920057	-1.525911733	0.714757315	-2.134866899	0.032771884	0.059782858
TMEM120B	10.05516946	-0.902655141	0.422864602	-2.134619773	0.032792081	0.059811539
PRR11	8.998983732	-0.925331346	0.43367068	-2.133718944	0.032865794	0.05993781
POLR2I	22.56249128	-0.749268726	0.351182128	-2.133561668	0.032878679	0.059953127
RFC5	13.04203841	-0.78316129	0.367100253	-2.133371695	0.032894247	0.059973333
CLASRP	34.51819923	-0.583589406	0.273746562	-2.13186022	0.033018339	0.06019137
POP7	19.54301604	-0.659492873	0.309369121	-2.131734647	0.033028667	0.060201986
NT5C	7.352661946	-1.082033921	0.507825723	-2.130719009	0.033112298	0.060346193
PGBD2	3.931922456	1.494349627	0.701388479	2.13055913	0.03312548	0.060361985
SKIC3	33.93224231	-0.57772369	0.271170049	-2.130484885	0.033131602	0.060364912
HIGD2A	16.32393775	0.741365461	0.348023437	2.130217057	0.033153698	0.060396936
KIAA1217	87.00977589	-0.472673952	0.221909674	-2.130028597	0.033169252	0.060417039
DOP1A	14.62917855	0.72511261	0.34049346	2.129593354	0.0332052	0.060474275
GALNT2	17.75530978	-0.846101814	0.397320408	-2.129520151	0.03321125	0.060477052
CFI	176.5146459	0.48214241	0.226438596	2.129241304	0.033234301	0.060509448
RAVER1	10.40032628	-0.862464081	0.405065719	-2.129195438	0.033238094	0.060509448
LOC114109042	1.405134531	2.596820316	1.219889576	2.128733918	0.033276281	0.060570716
VPS53	58.03596698	-0.497942184	0.233924401	-2.128645756	0.03328358	0.060575753
TNNI3	1.857138208	-2.383565233	1.119858094	-2.128452923	0.033299549	0.060596566
HAUS8	5.855732313	-1.162754491	0.546364667	-2.128165601	0.033323356	0.060631633
AXIN2	16.96915702	-0.897861436	0.42193765	-2.127948136	0.033341384	0.060656178
IPO7	191.671458	-0.308142081	0.144937468	-2.126034668	0.033500373	0.060934187
XYLT1	6.880010279	1.056514092	0.496949406	2.125999308	0.033503317	0.060934187
TEX10	18.51153521	-0.722482374	0.340126292	-2.124159145	0.033656838	0.061205075
GUCA1A	2.066548364	-2.277135354	1.072079741	-2.12403543	0.033667181	0.061215555
FAT1	115.3037402	0.378769916	0.178371925	2.123483931	0.033713321	0.061282775
TESK2	1.912181937	2.141160555	1.00830132	2.123532432	0.033709261	0.061282775
UBXN6	16.61566785	-0.764961165	0.360264172	-2.123333997	0.033725874	0.061297257
RTF2	12.37931247	0.792082426	0.373098144	2.122986779	0.03375496	0.06134178
RASSF4	15.02352454	-0.747109972	0.3519885	-2.122540856	0.033792346	0.061401372
SOCS4	33.59989538	0.528650194	0.249184073	2.121524815	0.033877662	0.061548026
TNFRSF18	3.83772294	1.579377159	0.74455616	2.121233083	0.033902192	0.061584222
LOC101120521	2.434256486	1.991291362	0.938967233	2.120725082	0.033944944	0.061653503
TRIM33	68.89356327	-0.437127015	0.206143069	-2.120503093	0.033963641	0.061672159
TTC39A	9.342898267	-0.928194728	0.437725794	-2.12049356	0.033964444	0.061672159
KDM5B	138.4707452	0.350125622	0.165120438	2.120425714	0.03397016	0.061674159

LOC105605336	7.139530659	1.046726928	0.493761403	2.119904312	0.034014116	0.061745575
PIK3R2	38.07640688	0.56636402	0.267187311	2.119726492	0.034029118	0.061764419
MAP3K7	14.70639057	-0.885144159	0.417712016	-2.119029678	0.034087959	0.061862819
LOC101113476	2.061166209	2.064531063	0.974354843	2.118869813	0.034101471	0.061878938
SLAIN2	54.62644639	0.425695317	0.200927406	2.11865233	0.03411986	0.061903902
LACTB2	13.91160937	-0.773032386	0.36504561	-2.117632333	0.034206218	0.062052159
CHCHD7	24.72369914	-0.618655535	0.292210924	-2.117154027	0.034246778	0.062117306
LOC132657887	1.205072034	2.813931236	1.330076461	2.115616145	0.034377468	0.062337435
SLC44A3	13.11502959	-0.828278987	0.391505335	-2.115626309	0.034376602	0.062337435
PRORP	10.32896971	0.973574487	0.460216835	2.115469085	0.034389987	0.062351678
CSGALNACT2	6.731691388	1.131729628	0.535020083	2.115303077	0.034404124	0.062368849
LOC114112704	3.639056623	1.572469599	0.743402205	2.115233972	0.034410011	0.062371061
PSMC5	73.08671677	0.489117673	0.231244478	2.115153964	0.034416827	0.062374958
ARHGAP12	115.3369764	-0.31173803	0.147403049	-2.114868264	0.034441177	0.062410625
EHD4	39.76485172	0.565457362	0.267382434	2.11478874	0.034447957	0.062414449
TMEM62	20.8824829	-0.693725308	0.328228604	-2.113543117	0.034554309	0.062598657
DDB1	11.61592465	-0.857188455	0.405718199	-2.112768067	0.034620624	0.062710294
CHN1	1.123229613	2.709216	1.282474237	2.112491558	0.034644309	0.062744693
SSH3	18.73509404	-0.788968646	0.373500118	-2.112365184	0.034655139	0.062755804
LOC114115866	1.696318888	2.351043709	1.11312011	2.112120416	0.034676122	0.062785295
FRY	1.641655055	2.278667775	1.079124895	2.111588552	0.034721755	0.062859404
WDR1	36.03526519	-0.557756856	0.264376124	-2.109709634	0.034883374	0.063143442
LOC114114080	5.642700517	1.133093215	0.537337165	2.108719235	0.034968824	0.063280977
LOC132657880	2.790000729	1.769150582	0.838947566	2.108773722	0.034964118	0.063280977
GALC	5.680697275	-1.228573308	0.5826961	-2.108428919	0.034993905	0.063309223
GPT	3.25317419	1.756683631	0.833166846	2.108441591	0.03499281	0.063309223
RCN1	69.01652428	-0.385545949	0.182932044	-2.107591102	0.035066374	0.063431445
YTHDC2	40.95840118	0.54135129	0.256864284	2.107538192	0.035070955	0.063431445
SLC1A4	9.926973071	-0.944861542	0.448409869	-2.107138151	0.035105606	0.063485527
LOC101122402	4.421812011	1.32394645	0.62853824	2.106389661	0.035170519	0.063594312
TIMELESS	58.25225751	0.699087869	0.332108865	2.104996113	0.035291646	0.063796071
TMEM231	5.442448009	-1.193793007	0.567109973	-2.105046753	0.035287239	0.063796071
ECHS1	102.3592464	-0.475283601	0.225818789	-2.104712378	0.035316352	0.063832098
TMEM143	2.86431417	1.730441024	0.82223832	2.104549231	0.035330565	0.063849152
MAL2	194.3339437	-0.348860049	0.165804726	-2.104041646	0.035374814	0.063920477
KIF1A	2.925923831	-1.761700305	0.837652953	-2.103138655	0.035453651	0.064054271
CRY1	12.16167846	-0.862088963	0.409963675	-2.102842313	0.035479556	0.06409241
LOC101110881	1.826622452	-2.450017672	1.165289502	-2.102496991	0.035509763	0.06413831
NANP	32.35408256	0.513119464	0.244131016	2.101820047	0.035569042	0.0642367
PIGH	4.597076723	-1.354226065	0.644378896	-2.101599033	0.035588415	0.064263003
CCDC90B	18.0711118	0.66760129	0.317693187	2.1014026	0.03560564	0.06427674
NUDT16	11.25352785	-0.80195164	0.38162657	-2.101404106	0.035605508	0.06427674

RABGGTB	37.89580468	0.513964643	0.244664778	2.100689142	0.035668263	0.064381095
LOC101114805	3.027407338	1.602300218	0.763014087	2.099961515	0.035732227	0.06448784
CBLC	5.105659165	1.192384225	0.568019758	2.09919498	0.035799716	0.064600919
TBC1D15	32.95534799	0.505415942	0.240809879	2.098817305	0.035833009	0.064652267
P4HA2	27.63358983	0.673596275	0.321198374	2.097134759	0.035981648	0.064911689
SNAI1	1.03359434	2.728536485	1.301170866	2.096985535	0.035994856	0.064926753
AAR2	5.35668471	-1.244521366	0.593509689	-2.096884667	0.036003786	0.064934099
CRACR2B	17.36612814	-0.653859872	0.311870632	-2.096574047	0.036031299	0.064974951
LOC105610122	24.03447234	0.617279284	0.294479172	2.096172982	0.036066849	0.065030285
TEKT2	4.140746173	-1.436075059	0.685223287	-2.095776787	0.036101996	0.065084878
LOC105607091	2.799165965	-1.701229771	0.812121901	-2.094796076	0.036189124	0.065233153
EIF3B	51.91010326	-0.435431741	0.207926388	-2.094162962	0.036245466	0.065317096
PSD3	87.79355658	-0.345436738	0.164950754	-2.094181014	0.036243858	0.065317096
CERS6	21.09301042	-0.623800333	0.297886733	-2.094085648	0.036252351	0.065320697
MGMT	6.630119152	-1.050609877	0.501722694	-2.094005096	0.036259526	0.065324818
PRKCI	37.43115792	0.505776109	0.241565704	2.093741378	0.036283025	0.065358344
SLC16A13	4.554253307	-1.354697999	0.647089273	-2.093525662	0.036302256	0.065384173
DNMT1	161.3986653	0.481832604	0.230227384	2.092855313	0.036362072	0.065483085
MARCHF9	5.191589816	-1.288575999	0.615850613	-2.092351573	0.036407077	0.06553395
MNS1	23.66099758	0.796495616	0.380675873	2.09231967	0.036409929	0.06553395
NTNG2	2.923668515	1.683592058	0.804608104	2.092437363	0.036399409	0.06553395
TBC1D1	8.727857354	1.044450363	0.499178277	2.092339375	0.036408168	0.06553395
MIPEP	3.036186409	-1.668720948	0.797605982	-2.092162028	0.036424024	0.065550492
GBA2	18.85605307	0.732723235	0.350402339	2.091091165	0.036519893	0.065714174
DENND4A	44.47659658	0.477861517	0.228534805	2.090979173	0.036529931	0.065723389
LOC121818789	1.121381889	2.80668129	1.342563218	2.09053939	0.036569374	0.065785498
ACP7	10.39641496	0.915151894	0.437805648	2.090315414	0.036589476	0.065812802
HDGFL2	2.721940912	1.769179325	0.846560079	2.089844971	0.036631728	0.065871072
PDHB	48.20155381	0.459740962	0.219983173	2.089891487	0.036627548	0.065871072
SCYL2	76.3761208	0.40305977	0.192896255	2.089515791	0.036661318	0.065915412
DLAT	102.9823472	-0.372366721	0.17824413	-2.089082663	0.036700283	0.065976594
LOC114108643	10.62914955	0.858998205	0.41120994	2.088952919	0.036711962	0.065988714
CCDC158	11.61473115	-0.825016997	0.395073656	-2.088261224	0.036774277	0.066091837
KCNAB3	5.389933705	1.14272707	0.547546069	2.086997122	0.036888395	0.066288019
MFSD14B	5.414807608	-1.168122411	0.55979776	-2.086686468	0.036916485	0.066329163
MRPS10	17.87635693	-0.790613	0.378893919	-2.086634171	0.036921216	0.066329163
KCNH6	3.619337847	-1.432576271	0.686767785	-2.085968944	0.036981436	0.066428421
RNF43	55.27611477	-0.404724705	0.194104791	-2.085083545	0.037061718	0.066563682
NAALADL2	15.31622857	-0.761530099	0.365282671	-2.084769304	0.037090246	0.06660597
LOC121817446	3.589922349	-1.398264841	0.67096226	-2.083969432	0.037162948	0.066727561
TRPM5	1.732306993	2.231088621	1.071135572	2.082918988	0.037258608	0.06688889
WDR75	39.40516567	-0.50531329	0.242603992	-2.082872941	0.037262807	0.06688889

HSF2	26.0738918	0.626006665	0.300755716	2.081445611	0.037393138	0.067113828
UFL1	68.98118211	0.372848793	0.179184438	2.080810126	0.037451289	0.067209174
YPEL1	3.28777579	-1.475980592	0.709927849	-2.07905718	0.037612095	0.067488692
TBC1D5	58.29406516	0.411595788	0.198008586	2.078676472	0.037647097	0.067542429
TSNAX	12.46890539	-0.910044361	0.438151311	-2.077009332	0.037800698	0.067808902
DDX47	21.07507821	-0.704786166	0.339431293	-2.076373573	0.037859413	0.067905115
LOC105605027	12.58762347	0.733413262	0.353360783	2.075536666	0.037936824	0.068034831
LOC101116756	0.958567639	3.257185273	1.569639754	2.07511645	0.037975744	0.068095491
CNIH4	19.66256945	0.679768765	0.327593925	2.075034712	0.037983318	0.068099936
LOC132660261	6.133213201	1.225838083	0.590824949	2.074790656	0.038005941	0.068131358
ALCAM	48.78268867	0.476835633	0.229830842	2.0747243	0.038012094	0.06813325
RAC3	5.144770721	-1.280673937	0.617312524	-2.074595747	0.038024017	0.068145482
USP15	57.68209509	0.406665108	0.196056951	2.074219278	0.038058951	0.068198945
CDK1	6.17387335	1.166788839	0.562763598	2.073319672	0.038142539	0.068339568
CLASP2	11.97519504	0.777190243	0.374941633	2.072829938	0.038188109	0.068402877
SOCS5	12.50648435	0.801837916	0.386830893	2.072838365	0.038187325	0.068402877
NEK3	4.470406933	-1.229932662	0.593684359	-2.071694569	0.038293934	0.06858324
LOC105608012	17.46569512	0.758048086	0.365941665	2.071499798	0.038312113	0.068606606
KLHDC10	52.2192486	0.453177398	0.218855252	2.070671798	0.038389477	0.068735935
MRPL2	18.70276522	-0.648304897	0.313130201	-2.070400412	0.038414863	0.068772176
NDUFC1	127.7115171	-0.403354707	0.194831138	-2.070278451	0.038426276	0.068783395
LOC114112673	6.777441385	-1.083940699	0.523866105	-2.069117833	0.038535031	0.068950364
MRPL42	14.37778563	-0.775958175	0.375017556	-2.069124931	0.038534365	0.068950364
RHBDF2	4.260955213	1.397456597	0.675382245	2.069134342	0.038533482	0.068950364
STON2	18.97113171	0.688030899	0.332532895	2.069061167	0.038540347	0.068950647
TMEM68	35.46963996	0.48815044	0.235940143	2.068958819	0.038549951	0.068958599
PCSK7	4.961574253	-1.243662353	0.601174029	-2.068722688	0.038572117	0.068979785
ZFHX2	4.264726909	1.317882694	0.637038584	2.068764324	0.038568208	0.068979785
CASC3	72.60571737	0.378714756	0.183095074	2.068404939	0.038601961	0.069023921
LOC121818997	9.122580465	-0.929464926	0.449507657	-2.067739918	0.038664486	0.069126474
TRMT9B	4.099895701	-1.43615414	0.694591264	-2.067624822	0.038675316	0.069136589
DTX2	36.74906211	-0.588800393	0.284812895	-2.067323512	0.03870368	0.069178041
LOC114115667	8.199052926	-0.952396891	0.460833315	-2.066684113	0.038763929	0.069276464
ACP6	26.26190423	-0.543269109	0.262906694	-2.066395117	0.038791187	0.069315909
RXYLT1	18.40539751	-0.641682745	0.310625624	-2.065775313	0.0388497	0.069411187
AMDHD2	22.79289201	0.721174705	0.349174824	2.065368567	0.03888814	0.069470581
TBC1D20	22.42807295	-0.627605843	0.303969777	-2.064698171	0.038951568	0.06957459
KDM3A	25.73372051	-0.556366572	0.269608902	-2.063606087	0.03905508	0.06975016
DOCK4	4.971852877	1.25629824	0.608853347	2.063383977	0.039076161	0.069778486
CPTP	24.36099534	-0.632108286	0.306391253	-2.063075499	0.039105456	0.069815421
MTHFR	1.761165274	2.293071059	1.111492304	2.063056173	0.039107291	0.069815421
BCAR3	13.02375321	1.050482381	0.509298798	2.062605261	0.039150147	0.069882595

PRICKLE3	3.111718763	-1.618243582	0.784737116	-2.062147373	0.039193707	0.069951007
LOC114117362	1.665513378	2.251021121	1.091770866	2.061807281	0.039226088	0.069990106
PTCD1	9.252093013	-0.905567746	0.439210392	-2.061808561	0.039225966	0.069990106
TRIM32	4.347086429	-1.293800737	0.627719022	-2.061114433	0.039292124	0.070098576
PIP4K2B	7.363851198	1.073568139	0.520949264	2.06079212	0.039322877	0.070144077
SLU7	77.24005328	0.393716142	0.191084932	2.060424849	0.039357943	0.070197261
LOC132659332	5.365720276	-1.180868258	0.573306299	-2.059751061	0.039422345	0.070302744
LAMTOR4	36.52788173	-0.651846586	0.316525013	-2.059384121	0.039457456	0.07035597
ZNF793	12.16453702	0.778187496	0.377940663	2.059020295	0.039492294	0.070408698
FOXA1	27.79190989	-0.707819442	0.343798349	-2.058821526	0.039511339	0.070433256
PFDN5	132.6007136	-0.384568635	0.186795881	-2.058764003	0.039516852	0.07043369
LOC132658501	1.144317165	-2.997161632	1.455865979	-2.058679628	0.039524939	0.070438711
URAD	14.44309285	-0.775772693	0.376908611	-2.058251447	0.039566002	0.070502491
SDHA	36.09118341	-0.786928856	0.382363514	-2.058064711	0.039583922	0.07052502
ADNP2	23.53806315	0.768977131	0.373700447	2.057736714	0.039615414	0.070566683
DIP2C	7.806249798	1.024565695	0.497915208	2.057711189	0.039617866	0.070566683
LOC106990437	4.127602772	-1.325736857	0.644668208	-2.056463837	0.039737829	0.070770928
MRPL33	35.78498952	0.643327841	0.312871281	2.056206111	0.039762653	0.070796274
PXDN	3.256330315	1.527365917	0.742802514	2.056220715	0.039761246	0.070796274
STRADB	4.903518236	-1.196713784	0.582380978	-2.054864136	0.039892129	0.071017342
C12H1orf53	4.983779121	-1.341008115	0.652647129	-2.05472154	0.039905908	0.071030476
PALS2	9.100322326	0.991381246	0.482499615	2.054677796	0.039910136	0.071030476
METTL3	2.32880694	-1.993554242	0.970340348	-2.054489691	0.03992832	0.071053379
MAD1L1	10.31679929	0.812342462	0.395438122	2.054284647	0.03994815	0.071079203
B3GALNT1	4.981334444	1.303922417	0.635064881	2.053211343	0.040052085	0.071252454
LRIF1	54.87288203	-0.434295674	0.211524555	-2.053169073	0.040056183	0.071252454
HSBP1L1	2.401237829	1.889164717	0.920182655	2.053032305	0.040069445	0.07126656
LOC101108931	833.7464364	-0.251284015	0.122415038	-2.052721786	0.040099568	0.071310647
ITGAE	13.15080044	0.725412094	0.353499832	2.052086105	0.040161295	0.071410918
RBM28	39.30775723	0.589559621	0.287414974	2.051248802	0.040242724	0.071546188
POLR2M	16.99298709	-0.673817915	0.328536809	-2.050966273	0.040270231	0.071585571
GNE	15.79173911	0.722518264	0.352336232	2.050649912	0.040301052	0.071621308
TAAR1	4.341321149	-1.314946193	0.64122602	-2.050675038	0.040298603	0.071621308
TRMT10A	5.450792815	1.135649256	0.553823657	2.050561114	0.040309706	0.071627165
METAP1	60.25741747	-0.398347006	0.19429997	-2.050165036	0.040348329	0.071676736
UBE2L3	28.22263858	-0.667081848	0.325371362	-2.050216845	0.040343275	0.071676736
LOC121820658	8.807524075	-0.938187081	0.457649323	-2.050013042	0.040363158	0.071693551
THYN1	30.52597711	-0.545994077	0.266358076	-2.049849907	0.04037908	0.071712301
CIAO2A	1.514635974	-2.297975503	1.121178561	-2.049607069	0.04040279	0.071744877
RAD50	105.0270682	0.367037652	0.179119807	2.049118173	0.040450561	0.071820164
BACE2	65.92396341	-0.42710206	0.208451887	-2.048923928	0.040469554	0.071844343
ST3GAL3	1.344692062	2.431334613	1.18691701	2.048445336	0.040516383	0.071917925

ZDHHC6	34.41771909	0.470504064	0.229694622	2.048389557	0.040521844	0.071918068
EIF3M	64.42103446	-0.541683125	0.264502731	-2.047930176	0.040566842	0.07198837
VAPA	56.4170423	-0.543995039	0.265776703	-2.046812358	0.040676512	0.072173405
PIP5K1A	64.05612346	-0.447535024	0.218662201	-2.046695876	0.040687954	0.072184125
LOC114115613	1.620791247	2.429951864	1.187331161	2.046566235	0.040700693	0.072197142
DUSP22	1.492094028	-2.295702838	1.121880828	-2.046298306	0.04072703	0.072234274
NRBF2	21.16728277	-0.586586917	0.286676929	-2.046160178	0.040740614	0.072248779
BORCS7	17.82197096	-0.645588944	0.315559808	-2.045852889	0.040770847	0.072283212
CREB3L2	13.05315264	0.791042375	0.386649985	2.045887508	0.04076744	0.072283212
NCDN	1.487468591	2.411379321	1.178769388	2.045675215	0.040788336	0.072304628
BIN3	17.01910912	-0.751962883	0.367749236	-2.044770756	0.040877464	0.072453015
EIF2S3	19.76956255	-0.678693416	0.332139242	-2.043400269	0.041012831	0.072670187
LOC121819944	2.006765294	2.031904814	0.994344073	2.043462489	0.041006677	0.072670187
TLN2	27.82457242	-0.568324247	0.278131512	-2.04336518	0.041016302	0.072670187
TIMMDC1	7.224988476	-1.034544567	0.50640663	-2.042912761	0.041061075	0.072739871
WASHC4	22.62392951	0.598563769	0.293018061	2.042753841	0.041076812	0.072758106
DPP7	5.692155377	-1.181191193	0.578320205	-2.042451885	0.041106728	0.072801447
LOC132660229	59.40386345	0.45819654	0.224350685	2.042322899	0.041119512	0.072814441
ANPEP	6.046839295	-1.056258221	0.517232898	-2.042132713	0.041138369	0.072838182
WDR83OS	28.08766819	-0.522512093	0.255908736	-2.041790762	0.041172291	0.072888588
LOC132658405	2.287300794	-2.001292764	0.980263642	-2.041586242	0.041192591	0.072914869
COMMD7	89.57221359	0.458953771	0.224833856	2.041301867	0.041220832	0.072955195
BMX	2.929426884	1.64236977	0.804906302	2.040448392	0.041305686	0.073095697
ZBTB41	45.05287284	-0.545059242	0.267148599	-2.040284858	0.041321962	0.073114819
PDPK1	34.35213112	0.522658213	0.256275454	2.039439227	0.041406211	0.073254191
NMRAL1	5.878654722	1.103818682	0.541278488	2.039280529	0.041422038	0.073272493
INO80E	55.88733619	-0.553999735	0.271688521	-2.039098791	0.041440169	0.073287259
UTP11	33.43577737	-0.465051208	0.228068357	-2.03908694	0.041441351	0.073287259
MST1R	25.37595362	-0.630998685	0.309562713	-2.038354941	0.041514449	0.073406817
DHDH	3.826419334	-1.381745589	0.67794756	-2.038130484	0.041536885	0.073436774
LOC132660138	3.231532953	1.513775547	0.742952998	2.037511862	0.041598774	0.073526853
WDR55	15.62024211	-0.740953777	0.363656291	-2.037511233	0.041598837	0.073526853
GYPC	5.541808431	1.132022998	0.555694635	2.037131413	0.041636875	0.073584354
PABIR2	19.42174239	-0.647369955	0.317831289	-2.036835193	0.04166656	0.073627082
MRPL48	12.2188228	-0.766503171	0.376516808	-2.035774111	0.041773043	0.073805485
TM2D2	28.56709846	-0.563491976	0.276816652	-2.035614448	0.041789086	0.073824071
RNF185	8.611395328	0.946251047	0.464874132	2.035499467	0.041800642	0.073834727
RNASE13	2.616935158	-1.854441798	0.911261312	-2.035027465	0.041848109	0.073908804
ZCCHC2	26.28063117	0.563429429	0.276970283	2.034259499	0.041925438	0.074035594
SCTR	6.361809031	1.211187451	0.595471195	2.033998388	0.041951758	0.074072285
ZFP3	18.45471162	-0.648862907	0.319068666	-2.033615257	0.041990402	0.074130725
MRPS18A	9.440340912	-0.866587611	0.426145241	-2.033549897	0.041996998	0.074132577

ARIH2	29.02326279	0.570561696	0.280628704	2.033155158	0.04203685	0.074193125
NUP62	21.04371159	-0.583240525	0.286873595	-2.0330924	0.042043189	0.074194515
UBA2	40.48515613	-0.451601272	0.222173269	-2.032653494	0.042087543	0.074262983
CFAP45	83.84027853	-0.455765218	0.224229422	-2.032584369	0.042094532	0.074265511
NSMCE2	71.70735793	-0.409277296	0.201367594	-2.032488387	0.042104238	0.074272832
LYRM4	11.83725624	-0.795400911	0.391378611	-2.032305517	0.042122736	0.074285855
MAP3K10	3.093299642	-1.586749034	0.780748512	-2.032343335	0.04211891	0.074285855
RAD23A	76.80675941	-0.400577998	0.197127142	-2.032079366	0.042145622	0.074316409
AFTPH	65.3351845	0.447983228	0.220587753	2.030861735	0.042269022	0.074524173
PRPF31	6.750912196	-1.058539415	0.521467516	-2.02992398	0.042364267	0.074682247
LGALS2	134.628686	-0.366118419	0.180387811	-2.029618399	0.042395343	0.074727174
RNF181	21.10364965	0.633827053	0.312385308	2.028991238	0.042459183	0.074829831
LOC114117582	62.51427032	0.405759317	0.199995752	2.028839681	0.042474622	0.074847172
CAGE1	4.982468691	1.187254805	0.585270819	2.028556297	0.042503504	0.074888193
MSANTD3	4.589095042	-1.265682542	0.624000132	-2.028336978	0.042525867	0.07491772
NDUFAF3	3.413145893	-1.468217371	0.724291635	-2.027108006	0.042651368	0.075128913
LOC101105270	2.135696067	-2.002293789	0.987900784	-2.02681668	0.042681164	0.07517149
EFCAB10	1.848048637	-2.160051466	1.065907315	-2.026490893	0.042714505	0.075200484
RAP1B	28.78645754	0.513254098	0.253258885	2.026598586	0.042703481	0.075200484
SGO2	79.90701944	0.369175012	0.18217087	2.026531532	0.042710345	0.075200484
RNF19B	10.34891052	-0.881423813	0.434975457	-2.02637597	0.042726272	0.075211293
LOC121817277	5.627246615	-1.179241939	0.582226462	-2.025400796	0.042826226	0.075377315
ANP32B	448.4361609	0.465710263	0.229941354	2.02534366	0.042832088	0.075377708
LOC121820573	12.97690064	-0.747019968	0.368898358	-2.025002147	0.042867144	0.075429469
PPIE	10.41572158	-0.825009021	0.407535628	-2.024385019	0.042930552	0.075531099
LOC105610458	7.476631994	0.95325093	0.471034776	2.023737903	0.042997126	0.075638272
IGSF5	26.97922479	0.668676606	0.330463729	2.023449317	0.043026844	0.075680589
LOC101112671	2.298736946	-1.931202901	0.954733493	-2.022766474	0.04309723	0.075794417
TNFSF9	19.66233692	-0.862738834	0.426555601	-2.022570638	0.043117434	0.075819974
DHX9	208.056764	0.309023092	0.152813068	2.022229496	0.043152648	0.075871915
LOC106990117	23.48325332	-0.5715728	0.282753012	-2.021456101	0.043232572	0.076002441
MGAT1	59.91560069	-0.490533748	0.242684505	-2.0212817	0.043250612	0.076024156
TMEM53	19.22104621	0.610012633	0.301808775	2.021189192	0.043260184	0.076030982
DBR1	57.81620009	0.555319168	0.274778539	2.020969944	0.043282877	0.076050865
TBC1D32	10.13901307	0.822679226	0.407066246	2.020995926	0.043280187	0.076050865
GAS2L1	33.46333399	0.623976221	0.308905328	2.019959397	0.043387599	0.076224849
ACTR1B	5.61170272	-1.259082803	0.623501637	-2.019373694	0.043448394	0.076315093
RAI14	24.48524359	0.608747832	0.301456643	2.019354512	0.043450386	0.076315093
THOC7	54.1354335	0.393990394	0.195132232	2.019094392	0.04347741	0.076352524
NME7	14.96280205	0.662236993	0.328044084	2.0187439	0.043513845	0.07640647
LOC132659479	1.71144595	-2.341350077	1.160472034	-2.017584231	0.043634581	0.076608407
PORCN	1.762595909	-2.057176074	1.019935569	-2.016966695	0.043698989	0.076703859

RAB12	80.90376225	-0.377876069	0.187349969	-2.016952934	0.043700425	0.076703859
FLT3LG	2.825125822	1.749148715	0.867348511	2.016661921	0.043730806	0.076747106
SH3BP4	6.872875007	1.077190812	0.534191876	2.016486697	0.043749108	0.076769145
PGLS	46.69888594	-0.404891088	0.200832907	-2.016059492	0.043793755	0.076817235
RBAK	22.5697045	-0.604083259	0.29962941	-2.016101354	0.043789378	0.076817235
SCAMP2	45.2466187	-0.41931468	0.207978388	-2.016145444	0.043784769	0.076817235
LOC121820216	4.495431366	-1.303337659	0.647096655	-2.01413135	0.043995745	0.077161413
PAIP2B	20.43519684	-0.681145796	0.338297672	-2.013451029	0.044067202	0.077269166
SQLE	279.551469	0.308508872	0.153225044	2.013436343	0.044068746	0.077269166
LOC121817228	5.149873673	1.238348635	0.615102391	2.013239832	0.044089405	0.077285113
SIKE1	117.3302888	0.375459251	0.186494263	2.013248266	0.044088518	0.077285113
NUP37	8.644470492	-0.914209692	0.454129169	-2.013104981	0.044103586	0.077299834
CPSF3	10.25474071	-0.850095359	0.422429308	-2.012396732	0.044178133	0.077416109
SEBOX	2.720103645	-1.589387857	0.789811057	-2.012364658	0.044181511	0.077416109
CHEK2	7.945670211	-0.899617883	0.4471681	-2.011811404	0.044239821	0.077508121
ZMAT2	210.766727	-0.309054343	0.153672455	-2.011123868	0.044312375	0.077625059
L3MBTL2	15.34804348	-0.665719651	0.331312628	-2.009339803	0.044501109	0.077945463
STXBP5L	2.706874554	-1.81844261	0.905076419	-2.009159195	0.044520254	0.077968777
CCDC6	32.88459062	-0.559395098	0.278590472	-2.007947706	0.044648849	0.078183744
LOC132658700	4.136756762	-1.216306195	0.60616191	-2.006569821	0.044795487	0.078430244
KIF5C	8.037251113	-0.890102049	0.443814075	-2.00557418	0.044901699	0.078605908
SLC36A1	40.09161235	-0.462209218	0.23049004	-2.005332713	0.04492749	0.078640758
PLEKHA7	42.99918663	0.483538698	0.241173835	2.004938463	0.044969626	0.078704206
PPM1F	17.27598026	0.654977906	0.326738922	2.00459101	0.045006788	0.078758934
LMLN	4.321252733	-1.409556609	0.7035265	-2.003558657	0.045117358	0.078942088
PSMD2	163.0985779	0.291394132	0.145443474	2.003487158	0.045125025	0.078945167
CRPPA	4.278407315	1.27339868	0.635675607	2.003220929	0.04515358	0.078984786
E2F5	10.96473258	-0.853221436	0.425992787	-2.002901133	0.045187901	0.079034479
LETM2	2.05431495	-1.854640009	0.926109814	-2.002613493	0.04521879	0.079078157
MAP3K8	2.553429246	1.676573499	0.837526875	2.001814568	0.045304679	0.079217992
NOSIP	28.44881307	-0.528895983	0.264234127	-2.001618755	0.04532575	0.079244471
LOC101111633	5.731550733	-1.073957628	0.536600715	-2.001409237	0.045348306	0.079273536
TRIM4	30.46866788	-0.50461537	0.252141887	-2.001315116	0.045358442	0.079280886
MLPH	21.34155214	-0.752010595	0.375994679	-2.000056481	0.045494165	0.079507716
DGKZ	14.3053146	-0.712900587	0.356475777	-1.999857024	0.045515705	0.07953496
ATP6V1F	76.49351988	-0.38067843	0.190463356	-1.998696429	0.04564121	0.079743844
LOC132658663	17.41388085	0.734777996	0.367685571	1.998386812	0.04567474	0.079791998
FBXO16	18.64499811	-0.619062827	0.309873172	-1.997794203	0.045738976	0.079893774
SIN3A	30.46144249	0.485358113	0.242988987	1.997449019	0.045776427	0.079948743
GATD1	47.14239517	0.487857302	0.244417974	1.995996015	0.045934357	0.080214085
MED19	76.74706732	0.580683738	0.290984692	1.995581737	0.045979469	0.080282375
SMIM32	4.098047138	1.333342025	0.668176479	1.995493805	0.045989049	0.080288614

LOC105608245	3.108503485	-1.477923315	0.740742413	-1.99519197	0.046021946	0.080335554
QSOX1	548.2304102	-0.251284087	0.125997221	-1.994362128	0.046112494	0.080483102
SFT2D3	4.502932882	-1.208340935	0.605913068	-1.99424802	0.046124957	0.080494343
YIPF6	123.2866874	0.363376516	0.182285636	1.993445691	0.046212665	0.080636877
GCC1	19.08347996	-0.702664812	0.352557042	-1.993052833	0.046255662	0.080701368
LOC114109488	6.767850862	-0.989642673	0.496714357	-1.992377832	0.046329618	0.080819847
LOC105611038	4.664112656	1.286406322	0.645793379	1.991978183	0.046373452	0.080885757
ABHD16A	20.25519973	-0.62607739	0.314327023	-1.99180263	0.046392718	0.080908803
POLH	23.1870642	-0.596013778	0.299255736	-1.991653647	0.046409073	0.080926767
LTN1	31.37953198	0.55182732	0.277257136	1.990308808	0.046556928	0.081174003
NPC1L1	25.5686847	-0.606985111	0.305000905	-1.990109212	0.046578906	0.08119114
RBX1	55.54628483	-0.384126102	0.193014545	-1.990140697	0.046575439	0.08119114
LRRC59	213.5884759	-0.339352868	0.170531824	-1.989967974	0.046594464	0.081207667
RPL8	1373.382228	-0.267270994	0.134387124	-1.988814001	0.046721737	0.08141887
CDK16	78.22235794	-0.403157685	0.202885206	-1.987122143	0.046908863	0.081726088
JARID2	149.8985909	-0.321984683	0.162036707	-1.987109526	0.046910261	0.081726088
RTN4IP1	20.24702385	0.608184086	0.306178496	1.986371001	0.046992144	0.081858074
RUVBL2	14.25790548	0.877297044	0.441689068	1.986232188	0.047007548	0.081874236
FKBP4	12.08975862	0.836540269	0.421408383	1.985105903	0.04713269	0.082071622
WDR77	16.29563779	-0.735479584	0.370499698	-1.98510171	0.047133157	0.082071622
INIP	72.50985221	-0.377211949	0.190049281	-1.984811243	0.047165476	0.082109641
OS9	28.53522295	0.527427061	0.265733763	1.984795062	0.047167277	0.082109641
MISP	305.6171349	-0.357726014	0.180248405	-1.9846279	0.047185886	0.082131339
LOC114113094	5.976240682	1.083508314	0.546025225	1.984355785	0.047216191	0.082173387
DMWD	7.875254058	-0.909060189	0.458142077	-1.984232041	0.047229978	0.08218668
TTC1	53.02540266	-0.397067309	0.200119636	-1.984149665	0.047239158	0.082191953
MIB1	51.17343346	-0.384478488	0.19379218	-1.98397318	0.04725883	0.082215478
SGTB	2.875522682	1.656645409	0.835135436	1.983684725	0.047290997	0.082260733
SNRNP40	17.36900704	-0.794477204	0.400685173	-1.982796613	0.047390153	0.082422483
USP48	140.6873611	-0.321531224	0.162252799	-1.981668266	0.047516381	0.082631271
MNX1	33.68022031	0.659689784	0.333041363	1.980804357	0.047613218	0.0827889
TSFM	23.08395953	-0.596835934	0.301341768	-1.980594784	0.047636734	0.082819016
LOC101110932	24.10095275	0.594758957	0.300369687	1.980089812	0.047693438	0.082906813
THNSL1	5.005111981	1.20223777	0.607462381	1.979114769	0.047803086	0.083086612
STOX1	2.702437711	1.605151029	0.811301715	1.978488396	0.047873636	0.083198417
LOC105614839	18.02030345	-0.665992951	0.336864444	-1.977035458	0.048037622	0.083472551
PYGB	8.184104263	1.051552339	0.532121769	1.976149822	0.048137811	0.083630078
RAB11FIP3	20.52067674	-0.664589697	0.336309799	-1.976123501	0.048140791	0.083630078
RAB11FIP5	9.663343269	-1.05863958	0.535731036	-1.976065431	0.048147367	0.083630631
NANOS1	2.439864208	1.938828211	0.981368451	1.975637397	0.04819586	0.08369311
TUBGCP5	19.48951426	-0.621990924	0.314827686	-1.975655103	0.048193853	0.08369311
FAAP100	5.681404537	-1.126529729	0.570315463	-1.975274744	0.048236978	0.083735782

RNF141	23.41055603	0.588253763	0.297811569	1.975254906	0.048239228	0.083735782
SNW1	148.7780453	0.342336494	0.173309601	1.97528868	0.048235397	0.083735782
LRRC2	6.606030682	-1.042386589	0.527785132	-1.975020754	0.048265793	0.083771015
BAHCC1	5.394252199	1.140040029	0.577354056	1.974594301	0.048314206	0.08383327
LOC105616368	4.872839428	-1.153783355	0.584297796	-1.974649506	0.048307937	0.08383327
LBR	151.6184179	-0.261879758	0.132659212	-1.974078948	0.048372767	0.083922292
LOC114117875	14.46679225	-0.715474271	0.362443038	-1.974032321	0.048378068	0.083922292
SLC35E1	31.04553023	0.537244599	0.272191565	1.973773867	0.048407462	0.083962384
CFAP20	38.96892593	-0.442152724	0.224084456	-1.973152147	0.048478231	0.084074222
WNT11	1.648471332	2.448760602	1.241274656	1.972779022	0.048520744	0.084137035
PACSIN2	41.23625348	-0.465228555	0.23584198	-1.972628261	0.048537931	0.084145004
PAPOLG	17.2691776	0.621561782	0.315090993	1.972642174	0.048536344	0.084145004
TVP23C	25.9984404	0.575497225	0.29178968	1.972301503	0.048575198	0.08419869
SUSD1	9.453910023	-0.833543431	0.42295168	-1.970776972	0.048749392	0.084489675
FIBP	7.373637871	-0.975886224	0.495213426	-1.970637656	0.048765336	0.084495396
TRIM40	12.73580791	0.783971913	0.397825393	1.970643222	0.048764699	0.084495396
BNIP1	15.21216199	0.632467994	0.321036966	1.970078404	0.048829386	0.084595407
PARP4	19.86940129	-0.606108943	0.307722775	-1.969659029	0.048877461	0.084667723
DPM1	89.85975043	-0.406691249	0.206540525	-1.969062722	0.048945889	0.084775269
MTHFSD	12.96130768	-0.734237425	0.372923518	-1.968868652	0.048968176	0.084802882
MAP1LC3B	6.057888922	1.085767175	0.551614395	1.968344527	0.04902841	0.084896195
LOC106990866	7.67855364	0.940539297	0.477854655	1.96825392	0.049038829	0.084903237
IDH3A	22.43671843	-0.541513384	0.275202555	-1.967690248	0.049103689	0.08500452
NRTN	1.775719642	2.087000406	1.060665262	1.967633411	0.049110233	0.085004839
TM9SF3	517.9619766	0.291138782	0.14798195	1.967393875	0.04913782	0.085041577
ADORA2A	1.875262486	1.919330915	0.975614862	1.967303892	0.049148187	0.085048506
HORMAD1	49.77745714	0.447696716	0.227584707	1.9671652	0.049164169	0.085065149
KLHDC9	6.959625489	-0.980829952	0.498807821	-1.966348382	0.049258382	0.085217128
CRK	75.14608243	-0.400429849	0.203687889	-1.965899154	0.049310261	0.085295839
ZMYM2	67.14511499	-0.424701974	0.216064928	-1.965621991	0.049342292	0.085340201
UCP2	6.923366192	-1.12379399	0.571829115	-1.965261931	0.049383929	0.085401164
COL7A1	2.824980558	-1.537283281	0.782325061	-1.96501858	0.049412087	0.085438804
CSTF2T	28.00284233	0.523306601	0.266450061	1.963995048	0.049530666	0.085632762
LOC101119762	20.68452932	-0.656612175	0.334393003	-1.963594241	0.049577166	0.085702069
SHCBP1	8.8822006	0.88006704	0.448286149	1.963181423	0.049625097	0.085773833
ASAP2	30.0656699	-0.536325398	0.273204664	-1.963090199	0.049635694	0.085780877
WDR44	14.93530592	0.662682831	0.337580606	1.96303585	0.049642009	0.085780877
ACY1	14.16799789	0.814995188	0.415272315	1.962556032	0.049697784	0.085866155
TMEM267	62.5169396	-0.449227594	0.228930879	-1.962284843	0.049729331	0.085905435
ZNF324	12.41752855	-0.897375271	0.457319516	-1.962250111	0.049733372	0.085905435
NAV1	18.31956064	0.696399446	0.355040323	1.961465787	0.049824712	0.086052087
TMEM19	24.16055601	0.558336905	0.284673495	1.961323817	0.049841261	0.086069547

PARN	29.16972778	-0.624490787	0.31842118	-1.961209955	0.049854536	0.08608135
SIDT2	15.67637388	0.720370695	0.367412909	1.960657007	0.049919048	0.086181605
PPP3CC	8.326999616	0.962082763	0.490807139	1.960205315	0.049971798	0.086261532
INTS12	3.522149002	-1.429413702	0.729405794	-1.959696118	0.050031319	0.086353127
TMEM177	6.85470099	-0.956135156	0.487956733	-1.959467084	0.050058111	0.086388213
TRMT11	39.08785824	-0.492434162	0.251351797	-1.959143192	0.05009602	0.086442474
SUGP2	14.82861885	-0.660245601	0.337120562	-1.95848511	0.050173117	0.086564332
THTPA	11.52180448	0.777613233	0.397176816	1.95785152	0.050247438	0.08668137
DCDC2	41.96894799	0.428404928	0.218823503	1.957764693	0.05025763	0.086687764
CDX1	4.665901946	-1.250553196	0.639067703	-1.956839925	0.050366291	0.086852774
LOC101109994	2.730552671	1.602029433	0.818675121	1.956856135	0.050364385	0.086852774
SUMF2	40.57041335	0.529107185	0.270489497	1.956109907	0.050452208	0.086989708
ETNK2	1.770863425	-2.120055249	1.084186664	-1.9554338	0.05053189	0.087115857
POLR2K	23.43738726	-0.685242481	0.350563835	-1.954686744	0.050620056	0.087256598
INO80D	75.59382422	0.402259839	0.205830301	1.954327602	0.050662487	0.087318478
RALA	161.33153	0.359796127	0.184107825	1.954268519	0.05066947	0.087319254
UHRF2	11.56168039	-0.749488769	0.38358827	-1.953888658	0.050714386	0.087385392
ITPK1	47.12965633	0.499509117	0.255702808	1.953475293	0.050763302	0.087458404
LEPROT	23.3789629	-0.766649745	0.392524569	-1.953125498	0.050804726	0.087518491
PTK7	12.04988584	-0.748442014	0.383264526	-1.95280795	0.050842356	0.087572027
HSPA1L	1.284799439	2.345808453	1.201411828	1.952543165	0.050873751	0.087614813
KLHL29	2.114279512	-1.867464997	0.956611865	-1.952165832	0.050918519	0.087673995
LOC105604257	7.14238319	-1.068540008	0.547376435	-1.952111818	0.05092493	0.087673995
VPS35	100.596764	-0.308425211	0.158000786	-1.95204859	0.050932436	0.087673995
ZNF3	26.2171284	-0.595704072	0.305171196	-1.952032435	0.050934354	0.087673995
IKBKG	13.41133028	0.706595826	0.361994657	1.951950983	0.050944024	0.08767935
PTRH1	9.152997205	-0.863345589	0.442421725	-1.95140867	0.051008451	0.087778931
TCF12	78.72095599	-0.428286196	0.219572531	-1.950545419	0.051111145	0.087944332
LOC105608827	13.23009315	-0.720508544	0.369404972	-1.950457083	0.051121664	0.087951108
SYK	7.555024519	0.929186606	0.476457075	1.950200037	0.051152281	0.087992457
IFI44L	4.248700796	1.228775396	0.630328351	1.949421114	0.051245155	0.088140875
TRIM13	71.4366069	0.388405246	0.199294034	1.948905537	0.051306707	0.088235389
GAK	33.62548311	-0.520544396	0.267206559	-1.948097372	0.051403313	0.088390156
LOC132657227	4.651099012	1.164590545	0.598163538	1.946943388	0.051541522	0.088616412
SKA1	7.114050288	-1.0806121	0.555080685	-1.946765809	0.051562818	0.088641623
ETV2	7.998300219	-0.91805938	0.471678324	-1.946367542	0.051610606	0.088712366
ACLY	53.72920231	-0.463178907	0.238035497	-1.945839647	0.051674004	0.088809919
RCOR1	63.45152092	0.450114165	0.231346513	1.94562762	0.051699487	0.088842291
CCNJL	1.596224328	-2.290735444	1.177529768	-1.945373702	0.051730018	0.088862192
EXOG	102.2221083	-0.366980164	0.188643305	-1.945365426	0.051731013	0.088862192
PAX6	2.051570378	-2.234750399	1.14870867	-1.945445749	0.051721353	0.088862192
GTF2H1	20.48341682	0.636520572	0.327296811	1.944780854	0.051801359	0.088971597

ADISSP	18.79349825	0.655447408	0.337082219	1.944473397	0.05183839	0.08902376
NEDD4L	66.72654346	-0.412126528	0.211967122	-1.944294584	0.051859937	0.089049322
LOC114111379	4.838808971	1.127494095	0.579965951	1.944069463	0.051887075	0.089084476
NDUFV2	131.3193798	-0.405692177	0.208734451	-1.943580343	0.051946078	0.089174323
TTC31	12.623168	0.784177132	0.403547547	1.943208771	0.051990939	0.089239873
XPO4	22.56549579	-0.634564963	0.326616175	-1.942846103	0.052034756	0.089303614
FAM83D	28.29693436	-0.626311597	0.322532783	-1.941854069	0.052154769	0.089498093
ARAF	12.01926528	0.842748785	0.434065312	1.941525297	0.052194594	0.089554935
KLC2	21.53684541	-0.634209657	0.326789371	-1.940729144	0.052291139	0.089709071
PPT2	3.450524548	-1.53265966	0.790009069	-1.940053246	0.052373219	0.089838353
LOC114110524	4.005679788	1.565356638	0.807082778	1.939524273	0.052437532	0.08993713
LINS1	4.564140075	-1.275539989	0.657709624	-1.939366465	0.052456731	0.089958515
GAB3	0.818043149	3.089696623	1.593871628	1.938485238	0.05256405	0.090119432
VIT	0.818043149	3.089696623	1.593871628	1.938485238	0.05256405	0.090119432
TBC1D4	34.17161664	0.495646574	0.255726566	1.938189611	0.052600094	0.090169662
LOC121818517	53.27050245	-0.487895176	0.25183871	-1.937331939	0.05270478	0.090337535
PLA2G6	3.582326351	1.337748987	0.690613735	1.937043704	0.052740001	0.090386313
LOC132658327	3.617241544	-1.336855085	0.69023399	-1.936814334	0.052768043	0.090422778
TMEM220	4.214328471	1.277723525	0.659724525	1.936753111	0.05277553	0.090424014
MAPK11	1.518643122	2.236395848	1.154798066	1.936612048	0.052792784	0.090441983
STMP1	128.4858122	-0.36808568	0.190083669	-1.936440319	0.052813795	0.090466383
B3GALT2	10.63956296	0.909347499	0.469754294	1.935793907	0.052892947	0.090590356
LOC101114408	28.40774124	0.575680398	0.29745644	1.935343532	0.052948154	0.090673289
NT5DC3	6.183437057	-0.979516193	0.506305586	-1.934634381	0.053035178	0.090810682
RIN2	9.802724328	-0.91222762	0.471742798	-1.933739367	0.053145181	0.090987382
LOC101117545	7.91994105	-0.942760488	0.487717555	-1.933005031	0.053235578	0.091130473
ZC3H7A	89.32227349	0.308295701	0.159617192	1.931469275	0.053425045	0.091443099
LOC105607312	2.125027742	-1.809077222	0.936690341	-1.931350354	0.05343974	0.091456539
ARIH1	64.72941954	0.407580967	0.211097614	1.930770124	0.053511486	0.091556387
LOC121817275	2.502580672	1.631816612	0.845164641	1.930767727	0.053511783	0.091556387
CDC37L1	42.16535759	-0.409353979	0.212078771	-1.930197811	0.053582333	0.09166536
NBEA	43.08906824	0.457746024	0.237198383	1.929802462	0.053631318	0.09173742
SDHAF4	46.36419113	0.476036431	0.246702572	1.929596544	0.053656847	0.091769344
LOC101104336	6.25174001	-0.991477648	0.513874972	-1.92941416	0.053679467	0.091789474
ZKSCAN5	19.0479242	-0.619764751	0.321223009	-1.929390905	0.053682352	0.091789474
ZC2HC1B	16.4957641	-0.794653636	0.411912626	-1.929180089	0.053708509	0.091822452
CENPP	6.829416078	-0.921433861	0.477760796	-1.92865105	0.053774197	0.091922997
APPL1	100.7990731	-0.360172228	0.186800125	-1.92811556	0.053840754	0.092025002
KCTD3	35.67721439	0.483912824	0.251060741	1.927473095	0.053920698	0.092149859
LRCH4	52.50238503	0.464285895	0.240912905	1.927193958	0.053955463	0.092185698
PLCD3	2.356102299	-1.686824095	0.875273423	-1.927196748	0.053955115	0.092185698
MAP2K1	28.27112969	0.553131837	0.287179508	1.926083936	0.054093894	0.092410402

MRPS30	14.63502318	-0.772091068	0.400878395	-1.925998202	0.054104598	0.092416877
CD2AP	267.1375607	0.295610062	0.15350555	1.92572883	0.054138242	0.092462528
PGAP3	8.09297694	-0.910126045	0.472650022	-1.925581303	0.054156675	0.092482192
UXT	48.44325154	0.410541992	0.213280043	1.924896426	0.054242317	0.092616608
PAQR6	1.718718319	-2.081329605	1.081623771	-1.924263926	0.05432151	0.09273998
LOC105602706	3.90568604	-1.290183469	0.670600136	-1.923923661	0.054364153	0.092789332
SOWAHB	9.832992392	0.793701475	0.41255392	1.923873307	0.054370466	0.092789332
ZNF365	1.483035204	2.413230577	1.254364493	1.923867098	0.054371244	0.092789332
TIMP2	195.3263436	0.355979746	0.185047255	1.923723467	0.054389255	0.092808219
LOC132659303	6.543347826	1.017185576	0.528959417	1.922993604	0.054480853	0.092952653
MTCH1	87.27986753	-0.505008276	0.262725894	-1.922186919	0.054582242	0.093113752
GOLGA2	150.3775234	0.341062047	0.177453931	1.921975163	0.054608883	0.093147311
PSME3IP1	49.50129762	-0.412887737	0.214832306	-1.921907111	0.054617447	0.093150031
ACSS2	54.42476065	0.439482116	0.228745015	1.921275167	0.054697026	0.093261953
VWA7	3.172756978	-1.441947781	0.750495383	-1.921327984	0.054690371	0.093261953
DDX59	7.193589468	0.983026212	0.511673774	1.921197179	0.054706854	0.093266812
ARL16	4.061382533	1.252823923	0.652338385	1.920512348	0.054793215	0.093402131
MEX3C	37.38789151	-0.54853527	0.285636524	-1.920396108	0.054807885	0.093415224
PEPD	12.00130074	0.701575502	0.365375776	1.920147826	0.05483923	0.093456731
MARK1	38.82159963	0.414245111	0.215746413	1.920055615	0.054850875	0.09346466
HEATR6	118.9061051	0.347278721	0.181099204	1.917615939	0.055159728	0.093978957
NUDT8	2.008588536	1.867332815	0.973978912	1.917220992	0.055209862	0.094052386
SURF2	4.393942574	-1.31862045	0.688130368	-1.916236386	0.055335014	0.094253574
BEND3	2.019814718	1.850142068	0.965767119	1.915722777	0.055400392	0.09435291
PLOD3	41.15992498	0.519970295	0.271456469	1.915483163	0.055430914	0.094392865
ANXA7	41.98953441	0.520041498	0.271585483	1.914835403	0.055513498	0.094504069
IL33	2.306554289	1.659793562	0.866821381	1.914804593	0.055517429	0.094504069
ZNHIT3	40.09195633	-0.476863532	0.249038776	-1.914816398	0.055515922	0.094504069
INHBB	1.904772729	1.932440465	1.009557548	1.914145923	0.055601513	0.094614513
LOC121818995	4.576966008	-1.11568805	0.582856913	-1.91417143	0.055598255	0.094614513
TNFSF12	4.706155514	1.108334382	0.579027745	1.914130008	0.055603546	0.094614513
LOC132658033	34.95146969	-0.550617971	0.28769069	-1.913923493	0.055629932	0.094647361
ZC3H4	11.05584709	-0.80221097	0.419178444	-1.913769616	0.055649599	0.094668773
PDCD2	22.73531318	-0.550737398	0.287920297	-1.912811993	0.055772125	0.094860763
RNF187	37.28960393	-0.488514701	0.25539558	-1.912776645	0.055776652	0.094860763
UBE2C	1.473968356	-2.319558324	1.212919228	-1.91237658	0.05582791	0.094935858
FDXACB1	3.071744149	-1.438943894	0.752545422	-1.912102381	0.055863064	0.094971471
NUDT19	141.3756557	-0.290638522	0.151996107	-1.912144511	0.055857661	0.094971471
QRICH1	8.789450716	-0.868948004	0.454475675	-1.911979125	0.055878872	0.094986264
LOC105610214	5.218528314	1.111290373	0.581541548	1.910938911	0.056012432	0.095201191
TTI1	4.215600029	1.278733009	0.669235852	1.910735961	0.056038521	0.095233423
MB21D2	36.12690376	0.447900019	0.234473716	1.910235513	0.056102897	0.095318586

SLC22A18	9.206466646	0.910742851	0.476763209	1.910262442	0.056099431	0.095318586
M6PR	72.39104108	-0.416668445	0.218173453	-1.909803595	0.056158506	0.09540094
TMEM106B	46.96052523	0.46946723	0.245953908	1.908761015	0.056292928	0.09561714
LOC132657217	4.016156384	-1.244666338	0.652181835	-1.908465204	0.056331116	0.095669848
PFKFB4	5.470574073	-1.039829837	0.544874484	-1.908384164	0.056341582	0.095675465
EWSR1	29.1998008	-0.756674468	0.39651357	-1.908319222	0.05634997	0.095677553
LAMC1	41.73446612	0.422406626	0.221374539	1.908108439	0.056377202	0.095711633
MPC1	25.9402634	0.550381023	0.288461207	1.907989741	0.056392542	0.095725517
TMEM87A	140.3080482	-0.37594391	0.197135621	-1.907031865	0.056516461	0.095923685
KHDC4	59.11519446	0.401635602	0.210616203	1.906954905	0.056526427	0.095925537
MIS18BP1	62.13512896	-0.475805914	0.249516372	-1.906912601	0.056531906	0.095925537
IFT74	42.43719241	-0.424360556	0.222546789	-1.906837468	0.056541638	0.095929872
PALM	1.850416747	1.974567109	1.035565902	1.906751763	0.05655274	0.095936531
CCKAR	2.097107041	-1.910315221	1.001955172	-1.906587514	0.056574023	0.095960456
CDK8	13.35185941	0.757836783	0.397839683	1.904879818	0.056795695	0.096324231
LOC132659849	1.688460876	1.945035325	1.021481292	1.904132106	0.056892981	0.096476984
E4F1	31.23271309	-0.444584703	0.233492997	-1.904060121	0.056902355	0.096480638
EGR2	1.202718123	2.373353368	1.246896914	1.903407845	0.056987348	0.09660024
TRIM37	45.24913585	-0.428327027	0.225025152	-1.90346289	0.056980172	0.09660024
ACTG1	505.1741054	0.299513427	0.157367702	1.903271273	0.057005157	0.096618175
LOC114112048	31.87634121	-0.509276329	0.267624872	-1.902948429	0.057047275	0.096677301
PYROXD1	15.32926947	-0.653881267	0.34364881	-1.902760167	0.057071847	0.096706682
LOC121817891	1.514016951	-2.35103707	1.235756806	-1.902507887	0.057104789	0.096737973
TMEM265	12.57727343	-0.689485007	0.362399234	-1.902556467	0.057098444	0.096737973
PERP	28.16396048	-0.676361316	0.355532841	-1.902387735	0.057120483	0.096752297
CLIC4	61.86648635	0.389596743	0.204850624	1.901857729	0.057189757	0.09685736
LAMC2	1.726375061	-2.051302527	1.078794023	-1.901477469	0.057239501	0.096929326
LSM1	17.08170104	0.634871664	0.333971613	1.900974929	0.057305297	0.097016161
TJAP1	9.567615478	-0.836236335	0.439891382	-1.901006407	0.057301173	0.097016161
CLPX	39.55309269	0.46654718	0.245518386	1.900253532	0.057399856	0.097156257
CYB5A	523.7870631	-0.362360109	0.190692489	-1.900232725	0.057402586	0.097156257
LOC121816955	7.415276765	0.911956322	0.479969031	1.90003159	0.057428974	0.097188613
CPNE1	3.917569686	1.243543231	0.654564565	1.899802245	0.057459076	0.097213346
RNF112	3.510688222	-1.348757902	0.709933196	-1.899837773	0.057454412	0.097213346
TNK2	4.194210272	-1.317325683	0.693419081	-1.899754015	0.057465408	0.097213346
KCTD5	13.22931154	-0.792237907	0.417131833	-1.899250656	0.057531527	0.097312882
ZWILCH	85.67201426	0.37668836	0.198356894	1.899043449	0.057558763	0.097346632
DPH3	9.224226777	0.832536824	0.438558048	1.898350349	0.057649946	0.097488509
LRRC45	2.347924948	-1.589933673	0.83830644	-1.896602004	0.057880486	0.097856006
SNAPC2	4.596311021	-1.131530146	0.596612556	-1.896591238	0.057881908	0.097856006
ENTREP3	18.59638482	-0.736747658	0.388536232	-1.896213523	0.057931816	0.097915611
PPM1H	37.11025654	0.459022302	0.242066384	1.896266199	0.057924853	0.097915611

LOC101111481	6.550274109	-0.938948822	0.49520147	-1.896094578	0.057947539	0.097929803
BDH2	12.39950082	0.731164653	0.3857477	1.89544786	0.058033093	0.098049592
ZNF787	3.011158046	1.547304286	0.816315542	1.895473264	0.05802973	0.098049592
CAMK2G	10.68957935	0.768384379	0.405505957	1.894878151	0.058108546	0.098164665
LOC101103150	1.958721121	-2.037051137	1.075599917	-1.89387439	0.058241685	0.098377146
ASB11	9.65379828	-0.876184354	0.462739162	-1.893473531	0.058294925	0.098454633
CHST2	1.608419838	2.039955587	1.077695095	1.892887513	0.05837283	0.098573752
LOC101108930	2.125869464	1.845009091	0.975057554	1.892205319	0.05846363	0.098714613
TADA1	22.19104175	-0.552321244	0.291921519	-1.892019626	0.058488366	0.098743905
TRPV4	1.59682121	2.226580703	1.177053189	1.89165683	0.058536719	0.098813057
NAA25	34.98584916	-0.488353395	0.258401172	-1.889903949	0.058770807	0.099195683
SUCLG1	106.8542603	0.443769637	0.234823961	1.889797083	0.058785104	0.099207285
GSS	9.538650988	0.776154349	0.410749193	1.889606509	0.058810606	0.099237793
FMO4	10.15297035	0.817836862	0.4328456	1.889442477	0.058832564	0.099262313
ELF4	7.395655117	0.885434386	0.468826961	1.888616613	0.058943219	0.09943646
CIP2A	44.36431924	0.475026373	0.251529163	1.888553862	0.058951634	0.099438105
LOC121819013	1.955483364	-1.804998374	0.955814963	-1.888439126	0.058967023	0.099451511
CST3	29.53099999	-1.197490516	0.634261334	-1.888008067	0.059024867	0.09953651
SYBU	29.4147519	-0.489279389	0.259161457	-1.887932698	0.059034986	0.099541015
KTI12	12.05855481	-0.750206998	0.397464134	-1.88748351	0.059095322	0.099630181
IL17RB	1.905645182	1.884373105	0.998515632	1.887174365	0.059136877	0.099687665
SHPK	5.115377484	1.090084435	0.577702883	1.886929194	0.05916985	0.09973067
LOC101120528	3.851821227	1.244730895	0.659839864	1.886413602	0.059239241	0.099835039
LOC132658860	2.233760313	-1.713536688	0.908567536	-1.885976132	0.059298171	0.099921754
LOC121818811	66.47378166	0.378646959	0.200847095	1.885249868	0.059396111	0.100074174
METTL15	51.00997119	-0.417274513	0.221343795	-1.885187309	0.059404554	0.100075783
PRKAA2	3.161604864	-1.408264676	0.747459514	-1.884068168	0.059555755	0.100317861
CCDC168	2.489311618	-1.592375052	0.845478979	-1.883399933	0.059646189	0.100406923
CDK2AP1	21.87458267	0.585225679	0.310704841	1.883542194	0.059626927	0.100406923
LOC132660000	9.377278285	0.799815579	0.42466532	1.883402158	0.059645888	0.100406923
SFN	13.5922389	0.779282811	0.413755115	1.883439701	0.059640804	0.100406923
STRIP2	1.426900591	2.05948241	1.093474197	1.883430277	0.05964208	0.100406923
HSD17B3	100.0642109	0.447713987	0.237725448	1.88332377	0.059656504	0.10041164
SLC7A4	2.126430737	1.827218161	0.970261414	1.883222537	0.059670216	0.100422073
MORF4L2	176.9646431	-0.378816065	0.201181124	-1.882960278	0.059705751	0.100469227
KLHL26	1.719149741	-1.938861814	1.029732284	-1.882879505	0.059716699	0.100475
DUSP5	9.036081828	0.961039997	0.510871025	1.881179301	0.059947532	0.100850688
ANXA11	43.52630663	0.455860284	0.242543627	1.879498094	0.060176513	0.101201968
LOC114111533	1.848206336	1.86198351	0.990691063	1.879479466	0.060179055	0.101201968
SNUPN	36.85084276	0.542064178	0.288408642	1.879500466	0.06017619	0.101201968
ZNF792	37.55699821	0.466840146	0.248477099	1.878805522	0.060271053	0.101343929
TTC22	6.758925707	1.072621847	0.571060106	1.878299387	0.06034022	0.10144747

WIPF2	35.24095389	-0.47035793	0.250427334	-1.878221206	0.06035091	0.101452681
CNOT11	37.84034373	-0.632038218	0.336624741	-1.877575062	0.06043932	0.101588525
HSBP1	69.01877606	0.339151925	0.180658417	1.877310402	0.060475563	0.101636663
KIAA0930	54.86002159	-0.459946285	0.245066489	-1.876822439	0.060542434	0.101736255
MYO16	1.722731975	2.005371521	1.068547753	1.876726159	0.060555635	0.101745647
ATG4C	39.52790382	0.463487234	0.246978732	1.876628119	0.060569081	0.101755446
LOC114114485	16.7569051	0.823820977	0.439048365	1.876378649	0.060603304	0.101800146
WHAMM	32.16057013	0.442732642	0.235966931	1.876248678	0.060621141	0.101817312
EIF3J	338.2682924	0.453324042	0.241696895	1.875589018	0.060711735	0.10195666
PEX6	3.965005002	1.209855066	0.645145065	1.875322515	0.060748368	0.102005362
LOC105604785	8.403715851	1.014503003	0.541009286	1.875204415	0.060764607	0.102019814
SERPINB1	465.305638	-0.336397309	0.179426458	-1.874847852	0.060813658	0.102089343
ANKRD42	29.21383243	-0.528196267	0.28179208	-1.874418428	0.060872776	0.102175753
LOC101117325	2.569182445	1.539842953	0.821596645	1.874207936	0.060901771	0.10219153
LOC132660158	2.221228404	-1.681668275	0.897280393	-1.874183687	0.060905112	0.10219153
PSMC3	86.79359342	-0.484629574	0.258574599	-1.874235044	0.060898036	0.10219153
YBX3	210.7291924	-0.266401557	0.142159018	-1.873968748	0.060934733	0.102228397
LOC114113389	1.707599486	-1.995487489	1.066008316	-1.87192488	0.061216997	0.102689054
ZNF428	19.22005879	-0.623986052	0.333371804	-1.871742133	0.061242288	0.102718586
LOC114118112	1.459205837	-2.146686895	1.147649763	-1.870506982	0.061413448	0.102992742
TRIM52	8.004786936	0.91997926	0.49192123	1.870175962	0.061459387	0.103056851
ZSWIM8	10.88947311	-0.778944319	0.416599634	-1.86976717	0.061516157	0.103139106
TRAF3IP1	22.96447355	-0.536091832	0.286829211	-1.869028018	0.061618917	0.103298437
CNOT6	40.63194191	0.452099709	0.241965898	1.868443916	0.061700221	0.103421764
VXN	1.841914089	1.969849858	1.054342549	1.868320557	0.061717403	0.103437593
LOC121820630	5.303655335	1.066437168	0.570877503	1.86806655	0.061752795	0.103483935
LOC114110260	4.379165104	1.266545212	0.678460606	1.866792559	0.061930562	0.103768822
OTUD7B	38.66300351	0.497923479	0.26687998	1.865720605	0.062080465	0.104006957
OPLAH	8.818219504	0.848346545	0.454914296	1.864849166	0.06220255	0.104198431
DUSP15	1.628632164	2.089970399	1.120834046	1.864656419	0.062229579	0.104230646
SEPTIN8	36.7020214	0.636685931	0.341472816	1.864528894	0.062247468	0.104247545
EXO1	11.29211651	0.797804766	0.427979158	1.864120603	0.06230477	0.104317369
SPPL2A	219.1818311	0.326611313	0.175205244	1.864164023	0.062298674	0.104317369
TM7SF2	24.1278597	-0.572137971	0.307099385	-1.863038479	0.062456853	0.104558905
ME1	3.884130231	-1.300908218	0.698452697	-1.86255737	0.062524567	0.104659156
BBS2	8.058561995	-0.840675366	0.451453237	-1.86215381	0.062581413	0.104741193
S100A10	99.28196715	0.360522251	0.193660051	1.861624263	0.062656071	0.104853017
RAB30	8.736832897	0.854637056	0.459113238	1.861495128	0.062674288	0.104870372
RNF168	41.37200903	0.433319439	0.232843988	1.860986163	0.062746131	0.104977442
UBR1	54.79732345	0.380091239	0.204303217	1.860427086	0.062825125	0.105096449
LOC114118018	36.15815892	0.455099657	0.244719061	1.8596821	0.062930516	0.105259577
SYT11	1.553909279	1.998293044	1.074662327	1.859461334	0.062961775	0.105298684

LOC132657159	1.638178124	2.133613568	1.147632036	1.859144308	0.063006686	0.105360612
ZDHHC9	84.68811622	-0.34560254	0.18592294	-1.858848291	0.063048645	0.105417587
UBE2A	27.08439916	-0.520291001	0.279931771	-1.858635052	0.063078885	0.105454957
PPP2CB	43.75753127	0.448978398	0.241601993	1.858338966	0.063120894	0.105511989
LOC121816400	2.307122098	-1.624865214	0.874487418	-1.858077292	0.063158039	0.105560879
IAH1	4.600926156	-1.257567009	0.677017988	-1.857509005	0.063238771	0.105682598
LGR5	5.030170528	-1.213314766	0.653277818	-1.857272255	0.06327243	0.105725628
MMS19	81.83614746	0.376414421	0.202696589	1.857033824	0.063306342	0.105769071
BCL2L11	20.18916965	0.574706402	0.30953822	1.856657322	0.063359923	0.105845361
LRRC28	9.990985782	0.7365801	0.396736889	1.856595946	0.063368661	0.105846729
LSM4	33.08633265	-0.48447326	0.261085569	-1.855611023	0.063509021	0.106067922
GUF1	49.08667653	0.485131581	0.261622482	1.85431916	0.063693513	0.106362755
RNASEH2A	6.466421097	-1.015889233	0.548145395	-1.853320747	0.063836399	0.106588046
DBT	8.035389129	-0.923657591	0.498473615	-1.852971879	0.063886389	0.106658191
DOK4	5.469039358	1.000167296	0.539789428	1.852884189	0.06389896	0.106665855
B3GALT4	4.672846367	-1.085896473	0.586098048	-1.852755656	0.063917389	0.106683294
PPP1CC	26.67092192	-0.501064128	0.270565306	-1.851915659	0.064037935	0.10687115
FAM114A2	57.75104507	0.378727342	0.204579359	1.851249044	0.064133733	0.106996818
KIFAP3	10.73266529	0.71236221	0.384805939	1.851224575	0.064137252	0.106996818
PLA2G5	5.113775044	1.193079482	0.644451771	1.851309184	0.064125086	0.106996818
LMCD1	1.307001611	2.333117661	1.26040382	1.851087424	0.064156977	0.107016368
C1H3orf38	26.96079989	-0.521927248	0.281986143	-1.850896792	0.064184402	0.107037478
LOC132658196	37.96941599	0.658534678	0.355793885	1.850888127	0.064185649	0.107037478
RARRES2	21.35659236	-0.598349459	0.323311826	-1.850688442	0.064214388	0.107072044
LOC101114349	1.429496806	2.066657781	1.116891121	1.850366381	0.064260761	0.107122641
LOC106991529	2.963557128	1.377920968	0.744655588	1.850413789	0.064253933	0.107122641
LFNG	115.189947	0.339580069	0.183578956	1.849776664	0.064345746	0.107250933
LOC101107122	2.29220488	1.699802977	0.919489331	1.848638065	0.064510092	0.107511458
EEF1E1	9.404858915	0.80713687	0.436679276	1.848351671	0.064551485	0.107550935
MOB1B	141.332747	-0.264779819	0.143251194	-1.848360292	0.064550239	0.107550935
SEMA3C	35.17379814	0.450487169	0.243729601	1.848307168	0.064557919	0.107550935
ZNF524	9.501387534	0.76812377	0.415717318	1.847706931	0.064644751	0.107682171
LOC121817115	1.436246854	2.338113185	1.265477984	1.847612691	0.064658392	0.107691473
RAB11FIP2	86.25126111	-0.310101961	0.167856809	-1.84741962	0.064686348	0.107724611
RNPEP	16.4676036	-0.746167277	0.403918124	-1.84732309	0.064700328	0.10773447
ERCC6L	11.12050477	-0.701017973	0.379554103	-1.846951374	0.064754188	0.107810722
EIF2B3	22.56357436	0.624948556	0.338397125	1.846790382	0.064777526	0.107836146
MECR	14.77167728	0.631366637	0.34191668	1.846551142	0.064812221	0.107880466
TFAM	112.0975929	-0.312905983	0.169601256	-1.844950858	0.065044688	0.108253928
SS18	19.05865191	-0.636247467	0.344890745	-1.844779765	0.065069582	0.108281877
PUS7L	11.16712681	0.756978192	0.410381985	1.844569744	0.065100152	0.108319261
HID1	9.439996319	-0.850314803	0.461021038	-1.844416489	0.065122466	0.108342903

G6PC3	3.215869942	1.385229629	0.751138189	1.844174147	0.065157765	0.10837465
ICMT	11.59370188	-0.741449948	0.402049614	-1.844175254	0.065157604	0.10837465
FMNL3	6.011767686	0.989977867	0.536908095	1.843849769	0.065205037	0.108439782
DNAJA3	25.39177505	-0.542330607	0.294289394	-1.842847953	0.065351213	0.10866936
THOC6	2.157232765	-1.777138101	0.965126591	-1.841352334	0.065569943	0.109019512
SERINC5	16.88033141	0.595242479	0.323462733	1.840219653	0.065735996	0.109282005
AMMECR1	80.64542327	0.336633358	0.182962424	1.839904339	0.065782283	0.109345354
ACSF2	16.64486467	-0.714634631	0.388472628	-1.839600991	0.065826839	0.10940581
LOC101103238	34.84455078	0.447160373	0.243141998	1.839091463	0.065901734	0.109516287
LOC101111082	2.909386647	-1.384924124	0.753070157	-1.839037321	0.065909697	0.109516287
LOC114118742	3.232682347	1.357378133	0.738148473	1.83889581	0.065930512	0.109537256
ITFG2	3.7708934	1.419992608	0.77250745	1.838160406	0.066038772	0.109689851
STK11	31.47651799	0.473486982	0.257587197	1.838161937	0.066038547	0.109689851
RNF212B	1.94759099	-1.752162708	0.953568583	-1.837479484	0.066139142	0.109842915
DDIAS	3.903397663	-1.366129451	0.743839021	-1.836592882	0.066270019	0.110046599
NAGK	11.02991007	-0.797061626	0.434068395	-1.836258145	0.066319487	0.110115063
FAU	1269.437922	-0.299771512	0.163263509	-1.836120724	0.066339804	0.110135116
CIBAR1	15.18725208	0.745734099	0.406196038	1.835897026	0.066372888	0.110176356
ACAT2	42.39564385	-0.427377268	0.23281357	-1.835706004	0.06640115	0.110209582
SPCS1	155.3269929	0.287686478	0.15674927	1.835328981	0.06645696	0.110288518
PRDM5	29.84972915	0.530813741	0.289273276	1.834990594	0.066507083	0.110357998
LOC101110128	4.416902922	-1.269462989	0.69192327	-1.834687522	0.066552002	0.110418826
RACGAP1	8.278142109	-0.918268882	0.500688667	-1.834011719	0.066652255	0.110571433
PCDH1	2.805132766	1.465306755	0.799023368	1.833872216	0.066672965	0.110592063
PLCL2	11.25797713	-0.789792287	0.430698161	-1.833748917	0.066691274	0.110608706
HPS4	7.419334873	0.873952285	0.476779538	1.833032284	0.06679777	0.110771588
BEX4	2.195730313	-1.779518917	0.970950895	-1.832758923	0.06683843	0.110812761
LOC101116495	2.989057418	1.531989193	0.835894664	1.832753884	0.06683918	0.110812761
SLC9A5	2.486765461	1.720605981	0.938990231	1.8324003	0.066891803	0.110886251
ILKAP	103.553961	-0.344515473	0.188022722	-1.832307652	0.066905598	0.110891447
TCEA1	37.03919334	-0.431419279	0.235456453	-1.832267809	0.066911531	0.110891447
ARHGEF37	18.24032854	0.561078391	0.306295737	1.83181913	0.066978372	0.11098846
LOC114117957	21.18705425	-0.538305436	0.293906261	-1.831554841	0.06701777	0.11103998
SLC31A1	67.4891595	-0.363573832	0.198610902	-1.830583464	0.067162739	0.111266381
RDH14	37.61404367	0.441096749	0.241045971	1.829927904	0.06726072	0.111414895
NAA40	9.13240657	0.847273199	0.463175942	1.829268582	0.067359383	0.1115645
PFDN4	93.92159552	0.320945555	0.175639054	1.827301778	0.067654408	0.112039254
UNC45A	8.988287966	-0.768107948	0.420368015	-1.827227382	0.067665588	0.112043887
EFNA5	11.63926501	0.777016476	0.425382465	1.826630244	0.067755383	0.112178675
PPIP5K1	18.49175505	0.740257618	0.405400866	1.825989238	0.067851883	0.112324532
MARK4	3.150786947	-1.306421994	0.71559094	-1.825654744	0.067902284	0.112394047
RHBDL2	104.8004081	-0.314472332	0.172311946	-1.82501759	0.067998375	0.112539163

ETV5	2.948839958	-1.391745463	0.762651447	-1.824877497	0.068019518	0.112560217
LOC121819614	2.659661942	-1.433647295	0.785639071	-1.824816699	0.068028695	0.112561468
HSD11B1L	10.45502346	-0.759836382	0.416513576	-1.824277589	0.068110117	0.11268224
UBA3	36.93247915	-0.507036643	0.277986676	-1.823960235	0.068158084	0.112747643
ANKRD50	23.54709383	0.524919817	0.287817307	1.823795179	0.068183043	0.112768447
PRMT5	15.65449577	-0.685192111	0.375701876	-1.823765479	0.068187535	0.112768447
ARVCF	2.67247051	-1.549680418	0.849784536	-1.823615697	0.068210192	0.112791961
LOC114110262	2.627504988	-1.578171728	0.86556824	-1.82327823	0.068261262	0.112862447
SET	101.8235378	-0.362489168	0.198840879	-1.823011291	0.068301681	0.112915308
TFG	19.98671626	-0.69276017	0.380076229	-1.822687447	0.068350743	0.112982442
PDZRN4	14.27732916	0.738188947	0.405107197	1.822206451	0.068423666	0.113088997
MAML1	10.35322845	-1.175723141	0.645375091	-1.821767151	0.068490324	0.11317118
PRKAG1	48.27174131	-0.445195043	0.244371786	-1.821793954	0.068486256	0.11317118
LOC121820746	4.909498099	1.13150431	0.621182011	1.821534254	0.068525685	0.113215612
RAB9B	1.471924807	2.138691936	1.174156505	1.821470926	0.068535302	0.113217507
LOC105605893	8.639614262	0.858626404	0.47153332	1.820924137	0.06861839	0.113340756
EARS2	3.062932127	-1.374335745	0.75542047	-1.819299053	0.068865818	0.113735391
PRKAA1	35.005397	0.421986856	0.231981392	1.819054765	0.068903075	0.113782865
OCRL	23.61069763	0.542931835	0.298539627	1.818625693	0.068968555	0.113876925
TTLL10	65.45581493	0.439876113	0.24190416	1.81839003	0.069004541	0.11392227
HNRNPA3	312.0967456	-0.271647942	0.149394866	-1.818321804	0.069014962	0.113925402
LOC121816399	26.33398034	-0.508426297	0.279671686	-1.817939829	0.06907333	0.114007672
PRCP	57.47711633	0.408122841	0.224524116	1.817723848	0.069106351	0.11404809
CARD19	17.24642762	-0.691400278	0.380573045	-1.816734756	0.069257737	0.114283817
MRPS15	18.14458477	0.579027161	0.318752338	1.816542477	0.069287198	0.114318318
LOC101119459	52.46307794	0.404061536	0.222538773	1.815690505	0.069417861	0.114519765
SLC25A10	11.68446973	-0.758341771	0.417887283	-1.814704111	0.069569393	0.114755585
TPGS2	23.51446221	-0.519384172	0.286228121	-1.814581218	0.069588291	0.114772593
LOC105611518	2.58696879	1.483922957	0.817893101	1.814323846	0.069627882	0.114823723
TAP1	25.89708456	0.560678675	0.309046579	1.814220616	0.069643767	0.114835751
SERTAD1	62.9470444	-0.395454652	0.217986242	-1.814126657	0.069658228	0.114845428
NOLC1	190.2532225	0.382297138	0.210839663	1.813212618	0.069799034	0.115063381
NHEJ1	4.643448327	-1.13409783	0.625500036	-1.81310594	0.069815483	0.115074768
XPNPEP1	15.61474648	-0.632855323	0.349054452	-1.813056155	0.069823161	0.115074768
CDKN2C	9.441625214	0.791247107	0.436565476	1.81243628	0.069918811	0.115144815
CNTROB	5.51939915	-1.132317407	0.624734279	-1.812478434	0.069912303	0.115144815
KIFC1	55.03766026	-0.413415161	0.228080278	-1.812586181	0.069895671	0.115144815
LOC101117613	3.91443622	1.189267351	0.656150954	1.81249047	0.069910445	0.115144815
LOC114116634	24.90123992	-0.485449989	0.267850741	-1.812389942	0.069925966	0.115144815
SAR1A	56.15122762	0.379851332	0.209553204	1.81267251	0.069882347	0.115144815
SNX14	22.3930212	-0.599669499	0.330834822	-1.812594863	0.069894331	0.115144815
PRKCSH	10.67856574	0.71155347	0.392620073	1.812320662	0.069936664	0.115148244

CDK2AP2	9.415976293	-0.750847177	0.414335705	-1.812171067	0.069959768	0.115172098
INTS5	25.74702256	-0.562448767	0.310462186	-1.811649832	0.07004032	0.115290507
SYF2	30.12316753	0.466243372	0.257415437	1.811248688	0.070102365	0.115378427
CCDC97	22.6781632	-0.641407223	0.354275137	-1.810477662	0.070221745	0.115560681
RPAIN	3.268342513	-1.482458809	0.818982225	-1.810123301	0.070276668	0.115625305
TUT4	22.60604797	0.513535989	0.283703881	1.81011267	0.070278317	0.115625305
STN1	20.3165021	-0.53553659	0.295886805	-1.809937384	0.070305498	0.115655789
NT5E	5.612453526	-1.021727938	0.564564611	-1.809762636	0.070332605	0.115686142
DSP	110.2477196	-0.32375926	0.178902262	-1.809699082	0.070342465	0.115688124
LOC121819243	23.7577342	0.646570542	0.357297523	1.80961384	0.070355692	0.115695643
CCDC14	13.46905418	-0.632094145	0.349353042	-1.809327722	0.070400105	0.115754434
LOC132657823	2.160213677	1.888294891	1.044276899	1.808231985	0.070570402	0.116020172
IRGQ	14.2876924	0.603896387	0.334000826	1.808068544	0.070595833	0.116047707
SLC12A8	6.21412154	0.965402317	0.534205083	1.807175461	0.070734926	0.116262053
COQ2	34.97849971	0.551499732	0.305294822	1.806449679	0.070848128	0.116433797
KLHL28	46.14650466	-0.404430759	0.223904904	-1.806261285	0.070877536	0.116467808
LOC105605937	1.832752983	-1.723060591	0.954485069	-1.805225296	0.071039434	0.116705147
LOC114118339	8.963559292	0.842441289	0.466654212	1.805279515	0.071030953	0.116705147
HAUS2	20.04104264	0.585339819	0.324265497	1.805125197	0.071055093	0.116716526
LOC132658031	4.61210097	1.099769264	0.609330248	1.804882109	0.071093132	0.11676466
DENND5A	102.0360104	0.357987389	0.198355184	1.804779593	0.071109179	0.116776666
BROX	17.59697582	-0.604430704	0.334933675	-1.804628049	0.071132906	0.11680128
PDP2	18.61879691	-0.612274267	0.339411841	-1.803927242	0.071242714	0.116967217
DTWD1	8.278323145	0.863714882	0.478997044	1.803173721	0.071360936	0.117146926
CHIC2	26.23375586	0.52206264	0.289552848	1.802996044	0.071388836	0.117178335
ZNF318	80.08574186	0.347737221	0.1929143	1.802547668	0.071459282	0.117279563
PGGT1B	20.58563555	0.510791682	0.283516095	1.801632043	0.071603316	0.117501526
LOC114117237	27.60883149	0.516596932	0.286799109	1.801250126	0.071663465	0.117585793
LOC132660038	1.991926198	1.804000599	1.001976052	1.800442831	0.071790743	0.117780173
UBE3B	42.54576721	-0.427104911	0.237244589	-1.800272504	0.07181762	0.117809808
LOC101106394	2.786488954	1.554139122	0.863760696	1.799270481	0.071975905	0.11805497
SLC25A26	4.053820219	-1.2474085	0.694012418	-1.797386426	0.072274294	0.118529842
LOC121817852	3.759291466	-1.193504285	0.664216785	-1.796859566	0.072357917	0.118652425
ORC5	3.149980144	-1.389749941	0.773634792	-1.796390178	0.072432485	0.118760132
LOC121816229	4.796005883	-1.037377583	0.577505362	-1.796308141	0.072445524	0.118766941
GRTP1	7.708657703	-0.8686943	0.484016378	-1.7947622	0.072691596	0.119149333
RNASEH2C	11.40805221	-0.689292962	0.384064824	-1.794730781	0.072696604	0.119149333
SLBP	25.76468351	-0.669338333	0.37303951	-1.794282681	0.072768061	0.119251827
VPS29	18.99380692	-0.623680514	0.347686463	-1.793801542	0.072844851	0.119363035
SP4	7.623849352	0.895064216	0.499000203	1.793715137	0.072858648	0.119371009
PRKDC	20.79925575	-0.516440448	0.287936885	-1.793589065	0.072878783	0.119389364
LOC121817423	25.47147117	0.53975053	0.300954177	1.793464159	0.072898737	0.119407416

DDX28	13.76209919	-0.658240787	0.367196607	-1.7926113	0.073035098	0.119602544
MRPL52	56.21121708	0.437803595	0.244227292	1.792607167	0.07303576	0.119602544
LOC132658665	8.584225748	0.873726806	0.487432745	1.792507408	0.073051724	0.119614031
LOC132659766	4.399634951	1.089179438	0.607683287	1.792347199	0.073077367	0.119641363
ZNF182	3.899693946	1.172338882	0.654323076	1.791682007	0.073183918	0.119801133
PHF23	14.59746511	-0.602150662	0.336096342	-1.791601355	0.073196846	0.119807622
HMBOX1	39.19477887	-0.664674823	0.371136795	-1.790915993	0.073306777	0.119972864
LOC132658769	3.076925925	-1.40839917	0.786476153	-1.790771614	0.073329952	0.1199961
LOC132659776	1.5478261	2.228209636	1.244429609	1.790546946	0.073366027	0.120025743
VPS45	45.00992236	0.397392781	0.221939181	1.790548107	0.073365841	0.120025743
PSKH1	11.33374976	-0.725760135	0.405387167	-1.790288876	0.073407484	0.120078868
TMEM51	146.4617201	0.323424036	0.180787332	1.788975107	0.073618825	0.12040984
PEMT	8.192521811	-0.783011843	0.437707888	-1.788891324	0.073632319	0.120417176
SPTLC3	26.34411887	-0.468981019	0.262448131	-1.786947452	0.073945982	0.120915341
WDR70	54.37277481	0.372574568	0.208504654	1.786888502	0.073955511	0.12091613
SSNA1	13.7472357	0.612293473	0.342715236	1.786595428	0.074002901	0.120978813
CARD6	28.63798334	0.490153042	0.274410193	1.786205672	0.074065963	0.121067098
LOC114108902	1.999328209	1.788262094	1.001204892	1.786110024	0.074081445	0.121077598
SMYD5	3.639634462	-1.22556329	0.686221941	-1.7859576	0.074106124	0.121103124
RSAD1	6.788688558	-1.013144565	0.567384215	-1.785641085	0.07415739	0.121172088
TNPO2	190.7501525	-0.356850368	0.19989923	-1.785151288	0.074236781	0.121286983
LOC105615359	3.512295955	1.195501604	0.669809548	1.784838104	0.074287581	0.121340316
SMCO4	30.51896355	-0.558824879	0.313093004	-1.784852654	0.07428522	0.121340316
LOC101106637	17.5382931	0.663820947	0.371970932	1.784604362	0.074325513	0.12138744
C16H5orf22	9.750152871	-0.848377118	0.475416448	-1.784492566	0.074343662	0.121402246
LIG3	36.25819511	0.411691555	0.230729919	1.784300698	0.074374817	0.121438285
TSC22D1	74.42730541	0.304034716	0.170440346	1.783818937	0.074453091	0.121551242
MAP3K21	20.61509406	0.556764109	0.31213389	1.783734885	0.074466755	0.121558701
LOC121816008	75.64743809	-0.337329448	0.189127131	-1.783612151	0.07448671	0.121576427
NUDT16L1	9.528246374	-0.823401214	0.461689285	-1.783453158	0.074512567	0.121594389
SPATA1	1.1996289	2.271633884	1.273742486	1.783432608	0.074515909	0.121594389
PLA2G12A	6.494467192	-1.009845295	0.566333209	-1.783129224	0.07456527	0.121660083
DEPDC1B	3.154369421	-1.414632978	0.793841644	-1.782009031	0.074747759	0.121942945
GSTZ1	4.138666501	-1.287933482	0.722828197	-1.781797511	0.074782258	0.121984338
PHB1	37.79557538	-0.407566762	0.228749744	-1.781714612	0.074795783	0.121991511
HIVEP3	9.458704396	0.758457191	0.425723335	1.781572982	0.074818894	0.122014316
UBFD1	7.732881947	0.808104534	0.453651876	1.781331844	0.074858255	0.122063613
ZBTB24	5.13132912	-0.99369453	0.557950492	-1.780972585	0.07491693	0.122144386
NSMCE3	7.569226828	-0.8567475	0.481232432	-1.780319535	0.075023682	0.122303516
PPFIBP2	55.97596878	-0.381509474	0.21434878	-1.779853721	0.075099903	0.122412842
CDKL2	5.912101573	-0.975569402	0.548249263	-1.779426746	0.075169825	0.12249609
PHLDA3	18.95845566	-0.578574545	0.325151686	-1.779398876	0.075174391	0.12249609

SLC6A9	2.405374606	-1.48522324	0.834688637	-1.779373978	0.07517847	0.12249609
EFHD2	30.98596451	-0.44866205	0.252154884	-1.779311364	0.075188729	0.122497873
TJP1	35.46602158	-0.423642517	0.238191187	-1.778581829	0.075308345	0.122677799
ZMYM1	48.6105307	-0.366692694	0.206187361	-1.778444094	0.075330946	0.122699662
CHRNE	2.284126006	-1.68432981	0.947429104	-1.777789814	0.075438382	0.122859683
CMPK2	24.73169003	-0.523252732	0.294368984	-1.777540296	0.075479387	0.122896857
LOC121817397	3.175756262	1.386988624	0.780285895	1.777539018	0.075479597	0.122896857
NAMPT	25.48292994	-0.47407377	0.266779489	-1.777024807	0.075564158	0.123019554
LOC101104234	2.80864984	-1.373559742	0.773020239	-1.776874231	0.075588935	0.123044904
PLRG1	46.33320253	0.392093995	0.220683186	1.776727996	0.075613004	0.123069095
TMPRSS4	12.10974187	-0.712397984	0.401076381	-1.77621525	0.075697446	0.123191533
ATP6V0A2	136.2731398	0.323416889	0.182106277	1.775978813	0.075736409	0.123224943
SUV39H1	9.184892074	-0.782612623	0.440665525	-1.775978785	0.075736414	0.123224943
AKR7A2	23.64349082	0.538664045	0.303319165	1.775898484	0.075749651	0.123231479
CCDC62	10.48912623	-0.69192695	0.389636749	-1.775825695	0.075761651	0.123233673
MAPK6	85.75973105	0.299600958	0.168715279	1.775778461	0.075769439	0.123233673
MLST8	13.85702704	-0.654504007	0.36861318	-1.775584927	0.075801356	0.123270584
LOC132657186	1.722338931	1.995006894	1.124094984	1.774767189	0.075936336	0.123475069
LOC121817909	2.344216644	1.537342667	0.866636	1.773919693	0.076076435	0.123687827
LOC132660094	5.308344702	0.957479334	0.539853315	1.773591655	0.076130719	0.12376103
GORASP2	43.75317071	-0.466091493	0.26304145	-1.771931736	0.07640589	0.124193253
DTWD2	14.82931903	0.580665157	0.32783836	1.771193451	0.076528538	0.124377485
MIER2	3.301891543	-1.398401343	0.789599622	-1.771025851	0.076556403	0.124407645
LOC132658382	5.080063379	1.021657577	0.576936371	1.770832327	0.076588588	0.12443753
MUC20	8.873297493	-0.9241383	0.521875178	-1.770803325	0.076593413	0.12443753
SGTA	32.59350584	-0.549834056	0.310581374	-1.770338154	0.076670826	0.12454816
LY96	4.370190226	-1.167685375	0.659736896	-1.769925834	0.076739497	0.124644563
LOC101119869	8.236923069	-0.807266368	0.456203003	-1.769533218	0.076804932	0.12473569
UBXN7	17.11523454	0.64128188	0.362417017	1.769458525	0.076817386	0.124740759
LOC101114892	3.509285177	-1.212945792	0.68555694	-1.769285263	0.076846281	0.124772522
MED25	13.66481856	0.602184135	0.340431222	1.768886329	0.076912846	0.124865432
IRF5	2.691661935	1.438642035	0.813617825	1.768203684	0.077026858	0.12503534
MDM2	17.09570357	0.585030561	0.330893118	1.768034839	0.07705508	0.125065962
EXO5	3.339954544	1.239542634	0.701162	1.767840576	0.077087559	0.125088299
WDR7	24.07666477	-0.520885127	0.294636422	-1.767891167	0.0770791	0.125088299
BOLA3	6.002677298	-0.902438348	0.51052649	-1.76766214	0.077117403	0.125092768
KCNJ15	2.519085057	-1.428969564	0.808397886	-1.767656236	0.07711839	0.125092768
SLC38A9	8.242291947	-0.808738613	0.457516063	-1.767672609	0.077115652	0.125092768
UAP1	58.88001499	0.64230853	0.36350414	1.766990962	0.077229742	0.125258188
BRD7	134.5049635	-0.317102353	0.17951201	-1.76646873	0.077317243	0.125384891
ASB6	6.68173658	-0.868457668	0.491693897	-1.766256756	0.077352783	0.125427308
LOC105615222	11.70100697	0.679072	0.384490378	1.766161234	0.077368803	0.125438066

IWS1	138.0553242	0.31530094	0.178532758	1.766067721	0.077384488	0.12544828
LOC121816538	1.861257796	1.762140984	0.998137957	1.765428287	0.077491813	0.12560703
AAGAB	63.54431813	0.327878639	0.185889347	1.763837705	0.077759307	0.126025331
TGFB3	2.092193092	1.730327749	0.981898108	1.7622274	0.078030885	0.126434817
UBE2V1	21.52170901	-0.553679471	0.314186127	-1.762265811	0.078024397	0.126434817
LOC101111669	3.05382782	1.463076813	0.830335822	1.762030224	0.078064191	0.126473452
COQ6	2.475417663	-1.400331783	0.794769702	-1.76193403	0.078080444	0.126484453
TMDD1	2.828891067	1.753875928	0.995489126	1.76182329	0.078099158	0.126499437
NEURL1	13.99604104	0.66014463	0.374713363	1.761732287	0.07811454	0.12650902
AP1AR	27.80754467	0.457683532	0.259890876	1.761060411	0.078228179	0.126677712
LOC121819931	1.312998364	-2.131506723	1.210651409	-1.760627962	0.078301393	0.126765554
TIAM1	21.86105331	-0.499326331	0.283604779	-1.760641459	0.078299107	0.126765554
ADAM8	3.591109824	-1.185630594	0.673671981	-1.759952362	0.078415884	0.126935534
LOC114114091	11.28357452	-0.66859816	0.3799493	-1.759703624	0.078458071	0.126988445
SLC23A1	2.27260688	-1.596489423	0.90752045	-1.759177353	0.07854739	0.127117618
SLC39A8	2.158226772	1.600950942	0.910603794	1.758120219	0.078727056	0.127392958
TTC29	1.379136695	-2.10244998	1.195892535	-1.75805929	0.078737422	0.127394308
FNTB	8.073899728	-0.813631532	0.462907932	-1.757653035	0.078806563	0.127490744
ACVR2A	2.893412198	1.449272561	0.82508747	1.756507782	0.079001744	0.12777557
ANP32A	66.71699505	-0.427371703	0.243304051	-1.756533441	0.078997367	0.12777557
C21H11orf98	27.14022138	-0.489171951	0.278512439	-1.756373801	0.079024603	0.127797078
RNF123	3.476668429	-1.276456638	0.726965864	-1.755868742	0.079110823	0.127921034
TIMM44	20.95101315	-0.533734417	0.304065048	-1.755329726	0.079202925	0.128054468
GOT1	24.49969487	0.464670419	0.26478305	1.754909991	0.079274705	0.128139147
NUDT13	8.923099739	-0.88499899	0.504314507	-1.75485531	0.07928406	0.128139147
TRABD2B	3.512849995	-1.327965467	0.756728599	-1.754876806	0.079280382	0.128139147
LOC132657622	6.986312066	-0.866070398	0.493858545	-1.753681104	0.079485165	0.128448642
DHFR	88.79608514	0.344962816	0.196735071	1.753438336	0.079526795	0.128500381
LOC132658296	1.658127413	2.130461509	1.215153901	1.753244184	0.079560102	0.128529684
LYSMD2	3.396684185	1.225192764	0.69882411	1.753220512	0.079564163	0.128529684
LOC132658647	1.750003926	1.792629012	1.024051836	1.750525656	0.080027651	0.129262789
RRAS	4.021395785	1.1515159	0.657859916	1.750396811	0.080049866	0.129283048
TM9SF1	36.87218172	0.5277534	0.301594093	1.749879763	0.080139063	0.129411468
MAST4	21.11945318	0.5034545	0.287764962	1.749533704	0.080198808	0.129492301
LOC121816721	1.027745609	-2.307487002	1.319470763	-1.748797371	0.080326051	0.129682088
MRPL53	72.42086504	-0.302727636	0.173122548	-1.748632053	0.080354642	0.129712578
EID3	1.597608572	1.791564816	1.024798699	1.748211446	0.08042742	0.129814382
BSPRY	16.38746255	-0.578812681	0.331118478	-1.748053097	0.080454834	0.129842949
LOC101120890	2.553529703	-1.40427341	0.803546786	-1.747593836	0.080534384	0.129939953
LOC121817253	3.837771704	-1.150480215	0.658301703	-1.74764885	0.080524851	0.129939953
LOC101110741	37.82771452	0.480060999	0.274729167	1.747397277	0.08056845	0.129979227
SPATA7	14.38905262	-0.608830924	0.34854456	-1.746780739	0.080675379	0.130136026

SLC25A1	11.57993203	0.708127293	0.405477835	1.746401978	0.080741127	0.130226366
OXSM	3.779235974	-1.215494014	0.696032028	-1.746319084	0.080755521	0.130233868
CLN3	16.42818383	0.598291785	0.34289014	1.744849777	0.081011022	0.130615969
RPP30	18.24503581	-0.53175203	0.304756174	-1.74484416	0.081012	0.130615969
TMEM30A	41.79323901	0.408823764	0.234345795	1.744532104	0.081066348	0.130687832
TXLNG	19.41539334	-0.550791899	0.315905594	-1.743533227	0.081240516	0.130952814
KSR2	2.74816445	-1.400804247	0.803462392	-1.743459633	0.08125336	0.130957724
PDIK1L	3.641851235	1.206197751	0.692002141	1.743054941	0.081324018	0.131055803
RUFY3	61.73839505	0.341562835	0.195980087	1.742844595	0.081360764	0.131099213
CTTNBP2NL	40.8383055	0.387064205	0.222162603	1.742256346	0.081463598	0.131249091
GLS	48.48246983	0.37216224	0.213643452	1.74197822	0.081512255	0.131305504
SIX4	7.281716565	-1.024844314	0.588333708	-1.741943901	0.081518261	0.131305504
AP5S1	19.80202895	-0.594160306	0.341183682	-1.741467537	0.081601659	0.131424
RHOBTB2	9.999784861	-0.710144036	0.407858723	-1.741152014	0.081656936	0.131497182
ATOH8	8.4476507	-0.826397719	0.4749269	-1.740052457	0.081849807	0.131791896
XRCC6	2.45627731	-1.482904799	0.852636836	-1.739198609	0.081999834	0.13201756
MRPS33	25.38425775	0.482529023	0.277456699	1.739114699	0.08201459	0.132025413
PKD1	6.853875092	-0.902061022	0.519057342	-1.737883177	0.082231401	0.13235849
ELOVL5	7.149243026	-0.850264726	0.489333873	-1.737596297	0.082281973	0.132423943
G3BP1	102.5054778	0.291752652	0.167938295	1.737261004	0.082341111	0.132503166
BPGM	11.21642999	0.698785357	0.402352385	1.736749633	0.082431373	0.132620976
LOC105609232	19.43946841	-0.524886015	0.302225943	-1.736733813	0.082434167	0.132620976
ERN1	22.58651629	0.487903871	0.280967287	1.736514866	0.082472838	0.132667223
LOC114109672	4.5729095	-1.097398134	0.632186418	-1.735877429	0.082585509	0.132832481
ATAT1	7.203876724	-0.831820523	0.479325444	-1.735398223	0.082670295	0.132952852
DLG4	2.703617909	1.543333041	0.889454096	1.735146364	0.082714884	0.132995586
RELCH	93.6073359	-0.326067778	0.187920617	-1.73513573	0.082716767	0.132995586
LOC132660040	42.82498625	0.424317175	0.244578426	1.734892083	0.082759922	0.1330393
MAU2	9.915259251	-0.818297185	0.471676414	-1.73486984	0.082763862	0.1330393
POLDIP3	70.55624176	-0.362010157	0.208728235	-1.734361219	0.082854012	0.133168196
PGAP2	3.338876429	-1.284256423	0.740771401	-1.733674411	0.082975869	0.133348018
DDX1	77.226441	-0.33120963	0.191094379	-1.733225393	0.083055615	0.133460129
SPINT2	170.285675	-0.304652893	0.175813918	-1.732814422	0.083128659	0.133561444
NR2F6	91.95224609	0.404013373	0.233167259	1.73271914	0.083145601	0.133572608
DUSP12	4.245892019	-1.504676589	0.86920459	-1.731096	0.083434644	0.134020845
DDX49	22.38687175	0.494851783	0.28588536	1.730944823	0.083461606	0.134048045
NFAT5	26.37507954	0.512712503	0.296239915	1.730734029	0.083499213	0.134092332
TMEM258	16.36813529	-0.605212201	0.349761151	-1.730358558	0.083566233	0.134183839
MAB21L3	17.32684259	0.597641558	0.34556028	1.729485685	0.083722206	0.134401995
RAPH1	14.71000194	0.591149855	0.341805234	1.729493281	0.083720848	0.134401995
LOC101106641	14.41640058	-0.597794522	0.345728394	-1.729087147	0.083793499	0.134500289
MTARC2	32.8004901	0.421194868	0.243639699	1.728761242	0.083851835	0.134577766

TOX	30.46143192	0.473857614	0.27412108	1.728643466	0.083872925	0.134595452
SPHKAP	2.442860279	-1.487309838	0.860657505	-1.728108834	0.083968714	0.134732993
TMEM94	10.5034506	-0.661450137	0.382782466	-1.728005318	0.083987271	0.134746593
C11H17orf49	1.493283333	-2.136449834	1.236474568	-1.727855865	0.084014069	0.134773409
C14H16orf87	16.30415556	0.663147974	0.38390963	1.727354365	0.084104041	0.13490155
EIF3H	97.17477999	0.367659511	0.212941319	1.726576658	0.084243721	0.13510938
C23H18orf54	2.697281639	1.587357627	0.919958546	1.725466472	0.084443441	0.135413441
STARD10	4.197475549	-1.084988556	0.628843201	-1.725372165	0.084460424	0.135424428
HNRNPM	370.4118584	-0.260433589	0.151041944	-1.7242468	0.084663299	0.135733436
TMEM60	5.544063493	-0.970435937	0.562967752	-1.723786015	0.08474648	0.135850498
SLC25A39	58.06505602	-0.343019968	0.199012726	-1.723608206	0.084778596	0.135885684
LOC101122242	7.787732005	-0.795895243	0.462032341	-1.722596389	0.084961538	0.136162581
CRB3	27.8015834	0.466726555	0.27105828	1.721867914	0.085093449	0.136357635
ANTKMT	20.38222748	-0.565611294	0.328508573	-1.721755049	0.0851139	0.136374058
FRAT1	4.200631631	1.146897834	0.666344502	1.721178505	0.085218437	0.136525186
LOC101102687	2.092803509	1.684729863	0.978926425	1.720997431	0.08525129	0.136561451
TMEM39B	13.05615594	-0.667227605	0.38772105	-1.720896003	0.085269697	0.136574568
TRMT44	2.071861727	1.832914995	1.065137113	1.720825397	0.085282512	0.136578728
ECD	13.75338538	0.636022181	0.369716171	1.720298517	0.085378193	0.136715578
LOC132659636	9.940622088	-0.793670255	0.461372717	-1.720236645	0.085389435	0.1367172
HIP1	5.840181827	1.020229914	0.593213895	1.719834822	0.085462471	0.136817749
USP28	21.29442347	-0.521860516	0.303465398	-1.719670578	0.085492339	0.136849174
MMACHC	8.750918186	0.750043083	0.436196576	1.71950704	0.085522087	0.136880399
SLC25A35	3.304231901	1.26451153	0.736169412	1.717690939	0.085853003	0.137393587
PTGES3	12.85827153	-0.675964083	0.393650938	-1.717166195	0.08594881	0.137530444
DNTTIP1	14.17098059	-0.616037709	0.358777548	-1.717046431	0.085970689	0.137548986
CNNM4	9.179207618	-0.792892482	0.461832554	-1.716839739	0.086008458	0.137592944
GARNL3	2.341428674	1.586155069	0.923985684	1.716644636	0.086044122	0.137633524
FHOD1	29.2853681	0.561321012	0.327013286	1.716508278	0.086069054	0.137656932
NF2	13.60029892	-0.641147247	0.373564998	-1.716293684	0.086108304	0.137703229
FAM161A	54.15050634	0.447254392	0.26060706	1.716202135	0.086125053	0.137713537
ERMP1	41.96367123	0.416555093	0.242728173	1.716138211	0.086136749	0.137715764
LOC101115083	23.36293688	0.496371133	0.289356134	1.715433248	0.086265824	0.137905634
PSMD4	19.22145553	-0.542579183	0.316307946	-1.715351098	0.086280875	0.1379132
INO80B	41.30436466	-0.370742652	0.216160907	-1.715123503	0.086322586	0.137963373
BAIAP2L2	4.147562369	1.088251563	0.634581156	1.71491314	0.086361153	0.137969777
RNF149	39.7194358	0.367329384	0.214180991	1.715041948	0.086337536	0.137969777
SLC25A27	7.046813681	0.794075354	0.463024609	1.714974405	0.08634992	0.137969777
SPATA17	1.677885923	-1.879260545	1.095857696	-1.71487644	0.086367883	0.137969777
CAPZA1	76.85335165	-0.340201958	0.19851593	-1.713726241	0.086579015	0.138290526
ITPR2	174.957484	0.328219802	0.191573101	1.71328752	0.086659657	0.138389633
MVK	57.76337025	0.358319181	0.209142704	1.713276024	0.086661771	0.138389633

NDUFS8	89.60006979	-0.385252111	0.224879893	-1.713146093	0.086685666	0.138411255
POMT1	2.576718828	-1.373229479	0.801900879	-1.712467856	0.086810483	0.138593994
GUSB	4.48005918	1.204388723	0.703539523	1.711899167	0.086915252	0.138701441
NRBP1	64.88383136	-0.34305261	0.200395566	-1.711877245	0.086919293	0.138701441
SPRY2	73.47709604	0.367972382	0.214931532	1.712044664	0.086888438	0.138701441
XXYLT1	10.64964607	-0.769826283	0.449683706	-1.711928344	0.086909874	0.138701441
BLVRB	72.43844946	0.357926935	0.209110275	1.711665934	0.08695825	0.138743577
TBL3	3.446836329	1.217839964	0.71151246	1.711621416	0.086966459	0.138743577
LOC132658760	46.34815091	-0.412426655	0.24101115	-1.711234748	0.087037786	0.138840799
LOC121817075	5.03097866	1.052550555	0.61528712	1.710665671	0.087142848	0.138991803
SEMA3F	7.984235161	-0.824988485	0.482397857	-1.710182734	0.087232087	0.139117537
UBE3D	13.49221533	-0.610162858	0.356875243	-1.70973714	0.087314492	0.139232343
DISP1	1.571834952	-1.895631963	1.108781022	-1.709654049	0.087329865	0.139238582
PIGA	2.662452614	1.356803224	0.793636253	1.709603385	0.087339239	0.139238582
CDKN1C	23.77586501	0.493103204	0.288549538	1.708903115	0.087468898	0.13941203
KRAS	141.6913046	0.308698321	0.180639808	1.708916348	0.087466446	0.13941203
ANKS3	2.069212945	-1.551559252	0.908294172	-1.708212273	0.087596962	0.139599498
LARS1	26.28673521	-0.479909963	0.280976739	-1.708006025	0.087635225	0.139643826
LOC101116975	14.37584455	-0.641671593	0.375847482	-1.707265909	0.08777264	0.13984612
EIF3E	73.54624623	-0.442689456	0.25937231	-1.706772231	0.087864396	0.139975627
NRBP2	2.763968528	-1.577656399	0.924414741	-1.706654307	0.087886325	0.139993876
SPSB1	1.65879946	1.830957484	1.07300195	1.706387843	0.087935892	0.140056141
OXLD1	5.914009184	-0.888368633	0.520715364	-1.7060542	0.087997989	0.140138344
ZNF362	23.08163281	0.520760537	0.305295714	1.705757772	0.088053188	0.140209545
LOC132658597	2.307900942	-1.46132111	0.856742029	-1.705672257	0.088069118	0.140218205
SLC7A7	4.043255721	1.138600459	0.667635102	1.7054233	0.088115506	0.140275352
OSTC	145.3951769	0.297583202	0.174508674	1.705263097	0.088145367	0.140306178
PPP2R2C	3.107163244	-1.361890438	0.798780507	-1.704962035	0.088201505	0.140378819
DUSP7	6.13326597	0.968768321	0.56842249	1.704310329	0.088323126	0.14055565
GALNT1	119.740223	0.281584044	0.165260944	1.703875323	0.088404382	0.140668211
LOC132659361	3.133913031	1.232995683	0.723692705	1.703755854	0.088426709	0.140686988
CEBPZ	233.4813584	0.283105216	0.166198412	1.703417101	0.08849004	0.140754239
PHC1	5.24278274	0.943451317	0.55384595	1.703454395	0.088483066	0.140754239
FBXO34	21.96814329	-0.484773304	0.284676641	-1.702891049	0.088588459	0.140894021
ANXA1	7.918490182	-1.083292318	0.636261218	-1.702590519	0.088644726	0.140966734
PADI2	2.990790047	1.323727466	0.777824026	1.701834118	0.088786469	0.141175344
MINK1	8.254668206	0.837221294	0.491974226	1.701758444	0.088800659	0.141181113
ALG14	15.91761728	0.651624084	0.383369326	1.699729322	0.089181851	0.141770293
L3MBTL3	9.704637841	0.761114608	0.447934048	1.699166677	0.089287783	0.141921811
RBM6	90.88781054	0.448997698	0.264414285	1.698084116	0.089491887	0.142229318
GNL2	76.80435924	-0.343474443	0.202293377	-1.697902563	0.089526154	0.142266861
ALPK3	2.643693695	1.40810504	0.82981766	1.69688488	0.089718427	0.142555456

CDCA3	5.834541456	-0.901551277	0.531574577	-1.696001495	0.089885597	0.142804099
GMCL1	22.35028979	0.518789072	0.305901451	1.695935313	0.089898131	0.142807038
PPP1R10	84.80791286	-0.367506714	0.216731347	-1.695678634	0.089946757	0.142867303
AMT	5.24393882	-1.006921129	0.593903634	-1.695428469	0.089994169	0.142921795
SH2D3A	2.576755874	1.522062665	0.897768269	1.695384787	0.09000245	0.142921795
STK24	20.6214844	-0.51382782	0.303091728	-1.695288168	0.090020768	0.142933902
OCLN	25.30472911	-0.486938749	0.287263262	-1.695095803	0.090057248	0.14297484
NDST1	22.896848	-0.56936147	0.335898835	-1.695038536	0.090068111	0.142975103
HSD17B7	68.74428378	0.314561207	0.185584512	1.69497553	0.090080063	0.142977095
LOC114117887	3.635506131	1.179068135	0.695719544	1.694746317	0.090123555	0.143029142
DMTF1	15.97029635	-0.571152696	0.337243546	-1.69359118	0.090342995	0.143360379
SIVA1	12.93466464	-0.593964729	0.350857469	-1.692894639	0.090475524	0.143553639
LOC114112955	3.09768005	-1.28074937	0.756639406	-1.692681296	0.090516147	0.143601048
SERTAD4	32.05311329	0.420891149	0.24868513	1.692466091	0.09055714	0.143649031
ZNF740	20.04915103	-0.517403238	0.305759122	-1.692192323	0.09060931	0.143714731
LOC105604916	2.537168326	-1.431616777	0.846444798	-1.691329169	0.090773952	0.143941706
SEH1L	30.22666737	-0.419659771	0.248119196	-1.691363575	0.090767385	0.143941706
CRAMP1	12.47323614	-0.688579989	0.407251641	-1.690797332	0.090875517	0.144085665
FAM133A	6.524925329	0.93743431	0.554855523	1.68951064	0.091121615	0.144458724
MPP2	2.648223952	1.399510971	0.828500253	1.689210071	0.09117918	0.144532841
AP1G2	23.88112276	0.474317715	0.280843622	1.688903281	0.091237966	0.144608877
WDR12	26.24071064	-0.490176014	0.290244493	-1.688838296	0.091250423	0.144611471
SELENOT	98.98472787	0.379228315	0.224581822	1.688597551	0.09129658	0.144667468
PURB	62.47640579	0.349952595	0.207313559	1.688035245	0.091404464	0.144821249
SRSF4	115.0837007	0.343568828	0.203550016	1.687884065	0.091433486	0.144850062
MLLT10	88.11405356	-0.284949415	0.168848877	-1.687600296	0.091487982	0.144919219
ANXA4	47.23372278	-0.488450213	0.289489429	-1.687281689	0.0915492	0.144999006
KLHL3	2.071874101	1.629963485	0.966242783	1.686908833	0.091620883	0.145095347
CIB1	18.21564114	-0.601564483	0.35666623	-1.686631456	0.091674239	0.145162645
APEX2	2.721078507	-1.291692319	0.766236969	-1.685760895	0.091841863	0.145410843
UNKL	3.04634737	1.312199187	0.77842826	1.685703428	0.091852936	0.145411151
DNAJC18	6.297935201	-0.856937393	0.508490297	-1.685258102	0.091938786	0.145529821
BAG2	35.77560332	0.391438204	0.23233578	1.684795189	0.092028093	0.145653937
CACUL1	63.22773098	-0.318184491	0.18886358	-1.68473186	0.092040317	0.145656035
FBXO28	7.86334625	0.751032926	0.445809864	1.684648517	0.092056405	0.145664249
JKAMP	17.72562223	0.548899104	0.325846269	1.684533956	0.092078523	0.145682
RNF5	13.68649019	-0.693956314	0.412057911	-1.684123263	0.092157849	0.145790249
SFSWAP	48.44777272	0.402730041	0.23918546	1.683756364	0.092228763	0.145885166
MAOB	20.30137778	0.500771539	0.297502467	1.683251718	0.092326373	0.146022281
BUB1B	49.17750097	-0.432075506	0.256775096	-1.682700205	0.092433142	0.14617385
SHISA5	104.7546428	0.28872318	0.171603794	1.682498815	0.092472155	0.146218244
IL18	23.88441789	0.502020265	0.298575373	1.681385372	0.092688085	0.14654234

TSPYL2	18.80727229	0.706589206	0.42030391	1.68113879	0.092735959	0.14660069
CHCHD5	16.91527417	0.550996983	0.327815286	1.680815405	0.092798775	0.146682643
MAGIX	5.038850202	0.971655635	0.578221285	1.68042177	0.092875283	0.146786217
AP1M1	3.070529396	-1.352805645	0.805072307	-1.680352975	0.09288866	0.146790001
RUNDC1	23.50995087	-0.459320247	0.273361405	-1.680267362	0.092905308	0.146798954
MRTFA	98.23567025	0.288516246	0.171742475	1.679935297	0.092969905	0.146883659
MACIR	10.68516773	0.668140159	0.397757254	1.67976863	0.093002341	0.146911658
RNF24	23.97548958	0.547207647	0.325770945	1.679731283	0.09300961	0.146911658
LOC121820768	2.827205195	1.32252739	0.787501574	1.679396503	0.093074794	0.146997247
LOC101112076	151.3270966	-0.282009055	0.167935725	-1.679267801	0.093099863	0.147019468
MORN2	1.783497852	-1.720312008	1.024529366	-1.679124157	0.093127849	0.147046288
LOC114118094	15.25050891	0.665345794	0.396412676	1.678417051	0.093265711	0.147229184
SESN2	31.18468117	-0.493783213	0.294186597	-1.678469442	0.093255491	0.147229184
DGKD	19.29136867	0.57136174	0.340452491	1.678242207	0.093299825	0.147265643
FICD	5.896313399	0.871468365	0.519810875	1.676510453	0.09363825	0.147782366
TNFRSF19	3.982466038	-1.175967205	0.701737184	-1.675794345	0.093778482	0.147986209
LOC105602274	18.48396567	0.556063204	0.33190292	1.675379065	0.093859881	0.148097176
EIF2AK1	10.94167361	-0.641639117	0.383107374	-1.674828417	0.093967902	0.148250115
RWDD1	93.639094	-0.313194333	0.187024294	-1.674618445	0.094009118	0.148297636
MRPL18	13.58510679	-0.597132621	0.356602904	-1.674502967	0.094031792	0.148315899
LOC105605201	310.5199352	0.294255671	0.175768574	1.674108545	0.094109269	0.148420588
ACTR3	118.5805298	0.286942049	0.17146586	1.67346461	0.094235868	0.148602715
ACTL6A	11.26672841	-0.678562369	0.405596887	-1.672996983	0.09432789	0.148713397
DOP1B	16.55278965	0.607631406	0.363199806	1.672994853	0.094328309	0.148713397
MAP3K6	8.381128704	-0.81375978	0.48653389	-1.672565462	0.094412871	0.148829158
RAD23B	124.6263394	-0.293250458	0.17534184	-1.672449991	0.094435621	0.148847467
EOGT	21.42777388	-0.507255178	0.303332197	-1.672276084	0.094469893	0.148883928
LOC101110828	33.41850223	-0.420916457	0.251761268	-1.671887264	0.094546555	0.148987179
PWP1	141.2659397	-0.260971874	0.156109128	-1.671727187	0.09457813	0.149019367
NSUN5	5.260772976	-0.942023048	0.563608332	-1.67141434	0.094639865	0.149099062
LEMD2	16.34056469	-0.570270938	0.341205371	-1.671342208	0.094654104	0.149103919
RBBP8	48.38689895	-0.367033199	0.219676984	-1.670785863	0.094763982	0.149259413
LOC114117845	2.367390545	1.389387051	0.832091166	1.669753397	0.094968164	0.149563389
FN1	352.8690639	0.337599363	0.202198202	1.669645724	0.094989478	0.149579332
RTCB	23.7214095	0.509428459	0.305244166	1.668921196	0.095132998	0.149787685
PUS10	3.068596217	1.220371087	0.731260435	1.668859723	0.095145183	0.149789225
CARD14	25.3442715	-0.532288282	0.318980817	-1.668715653	0.095173746	0.149816545
CMPK1	67.09909464	0.6102255	0.365777766	1.668295773	0.095257027	0.149929983
CSNK1G3	73.44353032	0.33652231	0.201758689	1.667944569	0.095326731	0.150018243
PBX3	39.79303071	-0.390072528	0.233870432	-1.667900152	0.095335549	0.150018243
SLC9A6	6.816436054	0.853342998	0.512017562	1.666628378	0.095588322	0.150398295
LOC105616575	2.365486897	-1.615692423	0.969545379	-1.666443322	0.095625148	0.150438528

RER1	46.22719909	0.40372077	0.242295193	1.666235159	0.095666586	0.150486005
NHLRC2	36.67710062	0.388597586	0.233296651	1.665680084	0.095777151	0.150642198
KALRN	15.14247924	0.579162266	0.34787367	1.664863757	0.09593994	0.150871755
LOC114110804	2.504743158	-1.408639089	0.846142073	-1.66477845	0.095956965	0.150871755
TSHZ1	13.30787973	0.614864157	0.36933077	1.664806203	0.095951426	0.150871755
DGCR2	14.61736716	0.58395551	0.351003029	1.663676556	0.096177084	0.151187352
FAAP24	17.86642678	-0.562956482	0.338384251	-1.66366041	0.096180312	0.151187352
FAM3B	86.09549097	-0.378307971	0.227405529	-1.663582999	0.096195792	0.151193903
USP45	24.89744158	0.486426822	0.292540036	1.662770088	0.096358466	0.151431777
CRBN	19.98298154	0.484839138	0.291711237	1.662051631	0.096502423	0.151640181
EXOC2	14.13266204	0.585745427	0.352460827	1.661873839	0.096538073	0.151678369
FKBP15	16.42920667	0.603609678	0.363241056	1.661733077	0.096566306	0.151704895
CDK13	22.49773892	0.461633354	0.277939474	1.660913249	0.096730871	0.151945566
FLOT1	19.70300341	0.546833634	0.329335264	1.660416283	0.096830737	0.152084562
FAF1	23.15248379	0.470770847	0.283602286	1.659968447	0.0969208	0.152208132
CDCA2	12.21757292	-0.662618572	0.399219464	-1.659785234	0.096957665	0.152248138
LOC105606443	1.527417159	-2.002105212	1.206363393	-1.65962033	0.096990856	0.152282366
TRIP12	116.0531008	-0.288697955	0.173961034	-1.659555293	0.097003948	0.152285033
AP4S1	8.685339091	0.759892279	0.45794546	1.659351048	0.097045074	0.152331704
EXOC7	21.90473368	0.483985794	0.2917712	1.658785354	0.097159053	0.152492708
MON2	15.53309049	-0.580078631	0.34972306	-1.658679958	0.0971803	0.152508147
CNKSR1	2.602203695	-1.396700787	0.842196603	-1.658402304	0.097236292	0.152578102
USP18	9.304492911	-0.704597306	0.425050823	-1.657677786	0.09738252	0.152789619
ITPRIPL2	44.19061224	0.386667886	0.233409224	1.656609281	0.097598495	0.153110502
SACM1L	107.9048139	-0.264360906	0.159586075	-1.656541191	0.097612271	0.153114143
GPR137B	22.98696518	-0.467789792	0.282550512	-1.655597042	0.09780345	0.153378025
PPIL3	21.70008823	-0.534430024	0.322796694	-1.655624219	0.097797942	0.153378025
GOLPH3	73.69768126	0.374303651	0.226142617	1.655166356	0.097890758	0.153496934
SETD1A	25.36602548	0.564058018	0.340810583	1.655048423	0.097914676	0.153516429
UBL7	2.713622183	1.290112653	0.780025914	1.653935632	0.098140592	0.153852586
METTL26	2.143481043	-1.484316009	0.898012688	-1.652889797	0.098353294	0.154167952
RHOD	2.563196578	1.376299751	0.832967935	1.652284192	0.09847663	0.15434318
PAK4	4.926394509	-1.032563855	0.625050222	-1.651969423	0.098540784	0.154425621
GNG13	3.598536641	1.310098143	0.793239663	1.651579219	0.098620359	0.154532207
CCT2	69.33092758	0.368466785	0.223165674	1.651090776	0.098720041	0.154670269
SLC37A1	3.968441471	1.199543625	0.726644749	1.6507979	0.098779849	0.154745835
RNF2	61.01077528	0.303077967	0.183602757	1.650726664	0.098794401	0.154750493
ST3GAL2	2.16582686	1.512833619	0.916707753	1.650289979	0.098883641	0.154872128
GPC4	9.807728636	-0.698498132	0.423321669	-1.650041054	0.09893454	0.154933691
VWCE	3.331194348	-1.142085145	0.692239772	-1.649840403	0.098975583	0.154979808
ZDHHC15	14.67083482	-0.566866505	0.343698864	-1.649311545	0.099083826	0.155131125
LOC780510	16.428428	-0.650221673	0.394379234	-1.648721882	0.099204625	0.155302063

DNAJC2	152.1876588	0.306551859	0.185975151	1.64834849	0.09928118	0.155403706
BCL7C	20.74074996	0.539596313	0.327388499	1.64818347	0.099315028	0.155438485
F3	38.8077998	-0.630524764	0.382637384	-1.647838896	0.099385735	0.155530937
COQ10A	6.221921257	0.832114831	0.505031813	1.647648346	0.099424853	0.155564998
LOC101117010	152.2738199	-0.271356469	0.164701744	-1.647562813	0.099442417	0.155564998
LOC121817907	1.821373444	-1.641329673	0.996214032	-1.64756731	0.099441493	0.155564998
LOC132657946	2.165231436	1.550317745	0.941175758	1.647213852	0.099514098	0.155658915
LOC132657669	3.394715627	-1.176532012	0.714284764	-1.647147009	0.099527833	0.155662183
IL15	4.788243133	1.00136856	0.607983984	1.647031149	0.099551643	0.155681206
COBLL1	38.21060462	0.411812085	0.250078266	1.64673281	0.099612977	0.155758897
YWHAB	235.1825475	-0.269692682	0.163802427	-1.646451078	0.099670924	0.155831276
UNC13B	11.35839354	0.626400755	0.380821391	1.644867565	0.099997125	0.156322991
WFDC3	4.549634717	1.117649358	0.679543532	1.64470605	0.100030445	0.156356791
TMX3	17.84125489	-0.621747639	0.378062379	-1.644563633	0.100059832	0.156384438
SHROOM4	9.790109206	-0.831017423	0.505537983	-1.643827864	0.100211764	0.156603582
LOC121817829	17.04215712	-0.576675507	0.350964098	-1.64311823	0.100358474	0.156814515
LXN	4.929086804	-0.924840963	0.5629131	-1.642955125	0.100392218	0.156848906
NEPRO	14.1533315	-0.581626356	0.35420094	-1.642080218	0.100573381	0.157113582
DCTN1	149.9821904	0.305356985	0.185971619	1.641954757	0.100599381	0.157135833
DPM2	18.64258111	0.553628838	0.337236987	1.641661087	0.10066026	0.157212554
LOC101110095	103.0206408	-0.327661428	0.199609785	-1.641509849	0.100691624	0.157243165
LOC101115589	2.295503087	-1.532410545	0.933687125	-1.641246305	0.100746297	0.157310164
NIPAL3	19.74158382	-0.499905278	0.30464811	-1.640926896	0.100812591	0.15738645
UNC5CL	10.53511269	1.012708399	0.617167389	1.640897458	0.100818703	0.15738645
ORC4	38.90080427	-0.405000971	0.246988745	-1.639754764	0.101056167	0.157738728
CAPG	3.326570929	-1.246140999	0.76011566	-1.639409716	0.101127959	0.157832357
LOC132658205	2.190676153	1.502435377	0.916609792	1.639122111	0.101187831	0.157907361
HAUS4	19.32699102	-0.522592131	0.319250369	-1.636935086	0.101644034	0.158600766
WTAP	20.57777073	-0.517418178	0.316194916	-1.636389934	0.101758005	0.158760066
RAD54B	4.375444827	-1.026605254	0.627738765	-1.635402036	0.101964796	0.15906413
WDR53	15.05194814	-0.611376075	0.373955409	-1.63489031	0.102072044	0.159212853
MARVELD2	46.12747886	-0.353131411	0.216032162	-1.634624248	0.102127841	0.159281298
TMEM185B	14.18475787	-0.601729705	0.368143088	-1.634499533	0.102154004	0.159303514
LOC121818625	1.906879733	-1.661113745	1.016331624	-1.634420996	0.102170483	0.159310624
RAB3C	17.88126981	0.550501768	0.336903505	1.634004275	0.102257953	0.159428415
DDX39B	11.71989183	-0.668168023	0.408988979	-1.633706669	0.102320457	0.159507258
CRTC3	5.747531427	-0.930589439	0.569783881	-1.633232303	0.102420149	0.159644047
PNPT1	11.7795173	-0.694764221	0.425409659	-1.63316513	0.102434272	0.159647443
LOC121820079	2.688882695	1.28071299	0.784461779	1.632600878	0.102552966	0.159813798
IDUA	2.571567515	-1.319157464	0.808092183	-1.632434383	0.102588011	0.159849773
LOC132657190	8.809909631	-0.821395289	0.503251054	-1.63217798	0.102641998	0.159915252
LOC105615213	10.03193909	0.664979115	0.40750003	1.631850468	0.102710991	0.160004091

SCAMP1	21.58285569	0.517610698	0.317288404	1.631357124	0.102814986	0.160147431
TXLNA	21.27204998	-0.54791131	0.336035295	-1.630517147	0.102992244	0.16040484
TRIM2	40.9851324	0.385648934	0.236527653	1.630460245	0.103004261	0.160404864
REXO1	12.39476344	-0.641889164	0.393746657	-1.630208542	0.103057429	0.160468965
MSS51	5.050111187	-0.978637732	0.60038011	-1.630030236	0.103095107	0.160508934
NEK1	57.67064283	0.454127324	0.278684043	1.629541894	0.103198354	0.160632257
TUBG1	5.853768822	-0.900017099	0.552304687	-1.629566289	0.103193194	0.160632257
RCBTB2	7.249643208	0.783764504	0.48101076	1.629411581	0.103225919	0.160656454
AASDH	15.53695455	-0.6212865	0.38136431	-1.629115481	0.103288575	0.160735253
ZNF575	2.403031231	-1.463988063	0.898705217	-1.628996956	0.103313663	0.160755579
SIRT5	22.53566605	-0.499205851	0.306531473	-1.628563117	0.103405538	0.160861082
SLC41A3	2.869817765	-1.215861612	0.746584353	-1.628565622	0.103405007	0.160861082
AKAP10	29.59167418	0.428217632	0.263047356	1.6279108	0.103543801	0.161057424
MAST2	7.335098384	0.827722759	0.50849982	1.627773948	0.103572827	0.161083826
KICS2	3.744952964	-1.044011034	0.641410455	-1.6276801	0.103592735	0.161096044
LYPD6	1.638515577	-1.701917998	1.045717321	-1.627512487	0.103628299	0.161101447
NDRG3	43.15823447	-0.36602452	0.224885818	-1.627601611	0.103609387	0.161101447
ZNF511	20.31623347	-0.492002124	0.302306696	-1.627493306	0.103632369	0.161101447
NIPSNAP3A	10.92495541	-0.648867971	0.399303805	-1.624998215	0.104162939	0.161907413
PIP4P2	1.858569693	1.804498517	1.110671495	1.624691481	0.104228313	0.16199019
ZCCHC3	19.64913865	0.512271981	0.315334408	1.62453563	0.104261543	0.162022995
ICOSLG	5.656819806	0.860131626	0.529507971	1.624397882	0.104290919	0.162030968
UCK1	60.63808559	-0.402944396	0.248049931	-1.624448731	0.104280074	0.162030968
ORAI3	21.24091576	0.505855663	0.311461337	1.624136296	0.104346723	0.162098826
SUPV3L1	16.33994457	0.580484987	0.357434381	1.62403232	0.104368911	0.162114453
LOC105604469	8.642627717	-0.714140591	0.439815317	-1.623728334	0.104433801	0.162196396
LOC114117296	1.601112077	-1.769971014	1.090232665	-1.623480079	0.104486818	0.162259883
LOC101121590	3.969093286	-1.040001438	0.640646315	-1.623362866	0.104511858	0.162279913
VPS37C	23.0542808	0.492376179	0.303364972	1.623048882	0.104578956	0.162365237
ACTRT3	4.722662238	-0.960309818	0.591949497	-1.622283357	0.10474269	0.162586048
ATP5MF	78.00192041	-0.330953033	0.204006111	-1.622270193	0.104745508	0.162586048
ALOX12	4.269939281	1.120120506	0.690548117	1.622074521	0.104787393	0.162632175
CCDC92	3.233930875	-1.1779321	0.726296295	-1.621834103	0.104838874	0.162693184
SLC36A4	42.0507249	-0.492949346	0.303964616	-1.621732662	0.104860602	0.162708011
VWA5A	7.80417569	0.768556417	0.474236031	1.620620044	0.105099151	0.163059228
ANKFY1	62.79456152	-0.282924472	0.174683035	-1.619644818	0.105308596	0.163365216
SMPX	3.58683006	1.083557491	0.669065464	1.619508927	0.105337807	0.163391567
PICALM	93.94521795	-0.421284551	0.26014283	-1.619435565	0.10535358	0.16339707
HYCC2	33.57326327	0.431623409	0.266564129	1.619210399	0.105402001	0.163453202
FANCG	17.81091567	-0.749498031	0.46309266	-1.618462342	0.105562996	0.163683876
ASB8	10.10245703	0.66269393	0.409553475	1.618088896	0.105643441	0.163789611
PKP4	180.0025494	0.295810226	0.182869455	1.617603259	0.105748126	0.163932899

MPZL3	18.182476	-0.5112617	0.316159534	-1.617100371	0.105856617	0.164082053
ELOB	43.73532496	0.3368721	0.208362609	1.616758891	0.105930336	0.164177281
FANCA	4.280991424	0.995013958	0.615832043	1.615722937	0.10615423	0.16450521
TCAIM	5.511301373	0.852573648	0.527699656	1.615641848	0.106171771	0.164513319
SH2D6	2.988822801	1.278107165	0.791208574	1.615385888	0.106227154	0.164580056
SALL2	2.337384257	-1.369931442	0.848504579	-1.614524513	0.106413704	0.164849973
KIF19	1.582865737	-1.905217443	1.180265383	-1.614228012	0.106477978	0.164930427
ZMYM4	29.65173406	0.432597993	0.268017679	1.614065144	0.106513297	0.164966017
FA2H	5.182960811	0.967268188	0.599937571	1.6122814	0.106900719	0.165546867
TUT1	8.990771321	0.687869935	0.426932408	1.611191659	0.107137955	0.165895031
RIOK1	39.42829446	-0.365658612	0.226957443	-1.61113294	0.10715075	0.165895625
GDI1	10.3829696	0.69210846	0.429774039	1.610400809	0.107310384	0.166123534
SRC	29.96006002	0.4946753	0.307213166	1.610202148	0.107353733	0.166171394
LOC101105166	18.79153892	0.515726052	0.320488832	1.609185719	0.107575738	0.166487883
RIPK3	2.084345023	1.423171457	0.884423262	1.609151996	0.10758311	0.166487883
TSEN15	5.260656434	0.9477051	0.589285189	1.60822827	0.107785194	0.166781304
LOC105603376	1.736480827	1.627322861	1.011927999	1.608140957	0.107804311	0.166791575
NDUFA13	161.8324767	0.330594641	0.205735006	1.606895427	0.10807731	0.167194597
NET1	142.845406	0.262207691	0.163219136	1.606476402	0.108169276	0.167317503
ABR	24.16267289	-0.481456494	0.29979028	-1.605977667	0.108278817	0.167428814
C9H8orf76	6.734491251	-0.851323905	0.5300661	-1.606071215	0.108258264	0.167428814
LCA5	2.777127939	1.416863277	0.882216784	1.606026209	0.108268151	0.167428814
L3MBTL1	1.838498655	1.692487353	1.05399355	1.605785304	0.108321091	0.167474806
COPS8	14.00078116	-0.614276754	0.382671478	-1.605232659	0.108442613	0.167643299
TMTC3	27.56267094	0.432983174	0.269806198	1.604793284	0.108539305	0.167773372
MTURN	2.733283076	1.383157181	0.861942579	1.604697592	0.108560372	0.167786533
VAMP5	1.416473915	1.846542825	1.151047886	1.604227632	0.108663886	0.167927102
GPD1L	4.491233412	1.035465629	0.645508264	1.604109021	0.108690024	0.167948076
LLPH	161.1880436	-0.360519202	0.224771507	-1.603936403	0.108728072	0.167987447
CDC14A	20.27767715	0.490242988	0.305737729	1.603475597	0.108829692	0.16810559
ERLEC1	93.19798333	0.30295289	0.188934401	1.603481888	0.108828305	0.16810559
HMGCL	16.94114309	-0.642426152	0.400684927	-1.603319986	0.108864026	0.168139193
SLC25A25	29.05344031	0.451204229	0.281431087	1.603249425	0.108879598	0.168143813
TM9SF2	278.7695388	-0.291584951	0.181936212	-1.602676825	0.109006023	0.168319605
CIMIP3	9.853433094	0.645860115	0.403024756	1.602532117	0.109037991	0.16834952
HS6ST1	16.61604502	0.50055191	0.31237839	1.602389684	0.109069465	0.168378663
CDC42BPG	34.70099989	0.396660785	0.247633224	1.601807619	0.109198158	0.168538404
TCTN1	10.82459877	-0.676422313	0.422276743	-1.601846003	0.109189667	0.168538404
ANKIB1	25.26721787	0.478388202	0.298806816	1.600994943	0.109378039	0.168796545
DCAF10	6.645411665	0.814472315	0.508770015	1.600865402	0.109406734	0.168813188
SF3B4	7.936900048	-0.80035945	0.499964604	-1.600832225	0.109414084	0.168813188
DCPS	2.39519169	-1.377859813	0.860770693	-1.600728073	0.109437161	0.168829304

ATL3	31.472472	-0.39936969	0.249529059	-1.600493712	0.109489101	0.16887045
DYNLT1	7.875858157	-0.794919754	0.49665421	-1.600549713	0.109476688	0.16887045
LOC114117762	2.631086802	-1.258491299	0.786526459	-1.600062254	0.109584773	0.16899851
LOC121818631	1.935012887	-1.47407461	0.921347085	-1.599912382	0.109618022	0.169030282
FDPS	23.58451652	-0.498208464	0.311410274	-1.599845945	0.109632763	0.169033512
SAMD8	5.167041901	0.936549629	0.585491668	1.599595143	0.109688427	0.169099828
TYRO3	5.359863793	-0.923269641	0.577232945	-1.599474957	0.109715109	0.169121456
HGSNAT	16.50943478	-0.559881143	0.350275443	-1.5984025	0.109953429	0.169469272
MMUT	42.46606049	0.441417701	0.27624995	1.597892418	0.110066922	0.169624637
CLOCK	56.65781176	-0.331720902	0.207726152	-1.596914488	0.11028477	0.16994077
LOC114109597	3.339616285	-1.172595836	0.734748805	-1.595913906	0.110508016	0.170265147
TMEM135	14.97344847	0.544097093	0.341072084	1.595255427	0.110655129	0.170472159
SDHAF3	7.300547459	-0.771211293	0.483600142	-1.594729254	0.110772793	0.170633762
PLEKHA8	28.67006108	-0.399099188	0.250279025	-1.594616996	0.11079791	0.170652784
ASAP1	10.92220698	0.674632307	0.423245527	1.593950235	0.110947182	0.170853315
CLHC1	2.292282608	1.346516139	0.844782093	1.593921261	0.110953672	0.170853315
INTS15	5.420753849	-0.908523258	0.570101251	-1.593617374	0.111021761	0.17093847
EXT2	29.22302331	-0.427984931	0.268786513	-1.592285735	0.111320517	0.171378718
METAP1D	2.659668034	-1.292861047	0.812330777	-1.591545075	0.11148696	0.171615191
POLR3F	5.598863932	-0.892379905	0.560836573	-1.591158544	0.1115739	0.171729243
LOC121817339	4.910063093	-0.889418942	0.55903027	-1.591003188	0.111608858	0.17176327
GNA14	6.802912296	-0.788193481	0.495511694	-1.590665752	0.111684818	0.171845901
HNRNPL	62.52428673	-0.441357922	0.277470089	-1.590650447	0.111688265	0.171845901
RSL1D1	132.005087	-0.257574337	0.161995626	-1.590007969	0.111833009	0.172048803
BRIP1	5.108267942	-0.92455335	0.581547943	-1.589814495	0.111876626	0.172091191
PM20D1	7.695978099	0.878783471	0.552773434	1.589771536	0.111886312	0.172091191
ZNF35	27.86572256	0.489470509	0.307905923	1.589675522	0.111907964	0.172104688
DAP	35.39407532	-0.479639683	0.301753237	-1.589509652	0.111945377	0.172142419
DSCAML1	1.273206163	1.812539589	1.140376107	1.589422628	0.111965009	0.172152803
DYNLRB1	22.19827677	-0.466038256	0.293244359	-1.589248835	0.112004225	0.172193291
KLHL21	12.76561109	-0.57852033	0.364116179	-1.588834453	0.112097773	0.172317289
DRC1	3.868520345	-1.061847379	0.668531905	-1.588327155	0.11221238	0.172473628
LOC132659162	1.999376368	-1.535284327	0.967138787	-1.587449854	0.112410796	0.172758732
CMC1	18.96502945	-0.49149335	0.309630499	-1.587354449	0.11243239	0.172772054
BTBD7	69.85898449	-0.32052159	0.201974494	-1.586940921	0.112526026	0.172896064
GNPTG	11.44525973	-0.599939516	0.378085163	-1.586784075	0.112561557	0.172910903
ZNF24	233.5273636	-0.229069992	0.144357087	-1.586828856	0.112551411	0.172910903
INTS3	2.857737787	1.180833965	0.744471121	1.586138042	0.112707999	0.173115963
LPXN	6.976307334	0.77866092	0.490945946	1.586042062	0.112729768	0.173129505
EML3	6.619079605	-0.771306725	0.486441947	-1.58560899	0.112828036	0.173240612
LOC101105041	88.20579701	-0.325264309	0.205133839	-1.585619958	0.112825546	0.173240612
DHPS	3.185402989	-1.110660475	0.701084669	-1.584203054	0.113147518	0.173711203

DIAPH3	52.94711412	-0.4571339	0.288617562	-1.583874166	0.113222357	0.173806136
ATAD1	64.22890725	-0.333127068	0.210373419	-1.583503609	0.113306725	0.173915673
RALGAPB	25.88153905	-0.444320611	0.280653612	-1.583163699	0.113384158	0.174014543
FAM217B	2.415359407	1.303371865	0.823315416	1.583077201	0.113403869	0.174024813
LOC105610254	8.319527468	-0.777338671	0.491218649	-1.582469785	0.113542365	0.17421734
CBLL1	52.98397968	-0.344778654	0.217916143	-1.582162064	0.113612578	0.174270502
TOMM70	130.5654917	-0.250756601	0.158486751	-1.582192822	0.113605558	0.174270502
TP53INP1	16.74791212	0.50380948	0.31843415	1.582146513	0.113616127	0.174270502
IGSF9	82.43462394	0.401407758	0.253832999	1.58138524	0.11378998	0.17451714
FBLIM1	29.17064891	0.448313216	0.283600777	1.580789803	0.113926107	0.174705868
BCL2L2	13.35452931	0.571113312	0.36145784	1.580027456	0.114100579	0.174953348
MYO5C	45.9139354	-0.396569893	0.251271772	-1.578250873	0.114507987	0.175557898
NRDC	65.46857643	0.366572037	0.23234285	1.577720327	0.114629874	0.175724613
WDR59	2.791898015	-1.258120102	0.797490517	-1.577598824	0.114657803	0.17574727
CDK5	4.329636376	1.147197978	0.727327133	1.577279225	0.11473129	0.175839746
SP1	29.84400096	0.425597302	0.269879805	1.576988326	0.114798211	0.175922138
GREB1L	1.688115145	-1.653935763	1.049009537	-1.576664181	0.114872816	0.176016286
USP47	134.7300779	-0.262921446	0.166772571	-1.576526905	0.114904423	0.176044535
LOC106990837	2.161191741	1.426569391	0.905296057	1.575804268	0.115070918	0.176279415
CEP295	129.2083429	0.25214721	0.160025536	1.575668582	0.115102202	0.176286929
ZFTRAF1	16.03565216	0.493741119	0.313351284	1.575679256	0.11509974	0.176286929
ADPRH	2.601444252	1.278261836	0.81141777	1.575343656	0.115177142	0.176381495
CHST10	2.061573477	-1.595691085	1.013014096	-1.575191392	0.115212273	0.176396585
CUL9	8.784041462	-0.70222924	0.445823219	-1.575129358	0.115226589	0.176396585
TSPYL5	2.071424004	1.426971956	0.905925397	1.575153937	0.115220917	0.176396585
SRP9	37.29630852	0.357790888	0.227165016	1.57502636	0.11525036	0.176412771
KIF21B	17.24241036	-0.566525697	0.359720811	-1.574903868	0.115278636	0.176435846
SHANK2	52.18042214	0.351076589	0.222939839	1.574759314	0.115312011	0.176466721
ESCO1	73.98277858	0.325322869	0.206623399	1.574472541	0.115378245	0.176527658
PARP1	72.32737156	0.377811179	0.239954773	1.574509955	0.115369602	0.176527658
PCID2	3.204810222	-1.279050705	0.812572198	-1.574076381	0.115469792	0.176647504
ZNF783	8.198218376	-0.767676813	0.487785087	-1.573801319	0.115533389	0.176724568
LOC132657406	1.342762282	3.763920082	2.392159696	1.573440138	0.115616939	0.176832133
SPATA13	50.03212194	-0.329888586	0.209743645	-1.572818024	0.115760961	0.177032151
RAB14	86.45809756	-0.256739947	0.163248751	-1.572691643	0.115790236	0.177056663
GKN1	21.71134313	1.490130806	0.947619897	1.572498436	0.115835002	0.177104854
SLC35E3	28.52807881	-0.437417994	0.278188188	-1.572381621	0.115862074	0.177125984
LOC106990092	58.38535347	0.343667166	0.218595932	1.572157186	0.115914102	0.177171383
SPAG1	6.44129054	-0.79677571	0.506809912	-1.57213916	0.115918281	0.177171383
ETS2	166.9308879	0.264652441	0.168401177	1.571559326	0.116052786	0.177356681
ERN2	37.55250731	0.442717588	0.28174991	1.571314034	0.116109723	0.177389416
TCN1	3.37786941	3.536548419	2.250721374	1.571295523	0.116114021	0.177389416

VAMP7	63.58281887	0.351612312	0.223764491	1.571349905	0.116101395	0.177389416
STAM	24.14229267	0.468996459	0.298506589	1.571142736	0.116149498	0.177423336
ACAP1	1.899325789	1.583838763	1.008325246	1.570761784	0.116237992	0.177538224
ARHGEF7	11.14646308	0.657218249	0.41852236	1.570330076	0.116338341	0.177597917
LOC101109640	3.845485372	-1.073906093	0.683852545	-1.570376686	0.116327503	0.177597917
LOC101114909	3.482014678	-1.0862036	0.691662533	-1.570424229	0.116316449	0.177597917
LOC121820333	7.541467977	-0.79995811	0.509378873	-1.570457967	0.116308606	0.177597917
SDHD	3.068316882	-1.189559665	0.757532779	-1.570307844	0.11634351	0.177597917
LOC101107315	21.75291651	0.509078145	0.324349154	1.569537452	0.11652276	0.177851229
IARS2	35.33410872	-0.47527406	0.302876423	-1.569201247	0.116601054	0.177950409
LOC101110467	15.85467824	-1.641725924	1.046460916	-1.56883635	0.116686076	0.178039508
NPL	3.744370082	-1.054521015	0.672153438	-1.568869481	0.116678354	0.178039508
DVL3	10.12584278	-0.656981606	0.418995658	-1.567991441	0.11688313	0.178319817
TSEN2	18.91364201	-0.516392914	0.329424052	-1.567562874	0.116983182	0.17845209
PKIA	25.52131441	-0.445778252	0.284396719	-1.567452165	0.117009039	0.178471165
FADS6	2.160790568	-1.386249764	0.884633282	-1.567033246	0.117106921	0.17860008
CLP1	9.872587618	-0.639797002	0.408737991	-1.565298593	0.117512913	0.179194024
KIF18B	5.718670169	-0.92818339	0.592991886	-1.56525479	0.117523179	0.179194024
TMUB1	8.600916114	-0.75513067	0.482497736	-1.565045001	0.117572358	0.179248561
ODAD1	4.285864055	-0.941396883	0.601645682	-1.564703133	0.117652534	0.179350338
LOC132657521	1.226237802	1.821139739	1.164081462	1.564443554	0.117713439	0.17942272
GBGT1	6.203810163	-0.78982469	0.504935573	-1.564208848	0.11776853	0.179486223
CPT1B	2.978982589	-1.198743064	0.766650958	-1.563609948	0.117909197	0.17968012
BZW2	157.0387816	0.253489625	0.162132838	1.563468748	0.117942381	0.179710199
MRPS12	8.086372174	0.80973951	0.51796093	1.5633216	0.11797697	0.179726514
SH2B3	8.00519856	0.705995533	0.451603379	1.563308791	0.117979982	0.179726514
DRAM2	35.95818946	0.411767235	0.263415844	1.56318325	0.118009499	0.179750992
MEAF6	39.99091404	0.364234134	0.233040155	1.562967264	0.118060295	0.179807874
SEMA3B	2.762018802	1.226011235	0.784531411	1.562730589	0.118115976	0.179872182
BHLHE40	2.894090414	-1.238179426	0.792602662	-1.562169148	0.118248147	0.180032433
ZNF341	7.717072781	-0.715356508	0.457909288	-1.562223187	0.11823542	0.180032433
LOC101115117	3.669919617	-1.089640909	0.697814505	-1.561505101	0.118404621	0.180250133
LOC101102683	2.479238715	1.246243777	0.798324966	1.561073285	0.118506461	0.18038462
DEFB1	13.27850647	0.685798669	0.439334045	1.560995959	0.118524704	0.180391847
MTRFR	34.12603391	0.397827195	0.254890249	1.5607784	0.118576046	0.180449439
DCLRE1C	27.37413596	0.422015135	0.270666453	1.559170449	0.118956043	0.181007112
LRIG2	17.23164293	0.510677611	0.327810749	1.557842789	0.11927052	0.181464971
HOXB8	5.332182396	-0.899446791	0.577536868	-1.557384198	0.119379296	0.181609796
TASOR	70.30204515	-0.285246339	0.183236664	-1.556709951	0.119539365	0.181832611
C4H7orf25	4.400036304	-0.917249941	0.589752271	-1.555313962	0.119871314	0.18229605
SCAMP3	14.660422	-0.508667476	0.327044633	-1.555345736	0.11986375	0.18229605
CCM2	17.03957573	-0.481129834	0.30936465	-1.55521917	0.11989388	0.182309625

ZC2HC1A	52.00050843	0.356945985	0.229566339	1.554870745	0.119976856	0.182394297
ZER1	4.779440437	0.918279892	0.590582113	1.554872509	0.119976436	0.182394297
C3H9orf78	54.12200395	-0.304205901	0.195664708	-1.554730559	0.120010254	0.18242432
GPALPP1	21.87230165	-0.431177568	0.277410155	-1.554296265	0.120113764	0.182560902
ZBTB9	16.10197566	0.515431825	0.331646215	1.554161638	0.120145866	0.18258893
NT5DC1	37.16839798	0.455381632	0.293088229	1.553735657	0.120247484	0.182722586
LOC121817236	32.90749719	0.364797893	0.234836876	1.553409747	0.120325276	0.182820011
ATG4A	4.405904189	0.972322073	0.626054954	1.553093808	0.120400725	0.182913855
PSMD12	37.92544499	-0.36119566	0.232626401	-1.552685585	0.120498268	0.183041237
ORC6	4.200589993	-1.029868393	0.663416874	-1.552369912	0.120573739	0.183135067
TULP2	4.679894883	-0.884672328	0.570010634	-1.552027762	0.120655581	0.183238552
EEIG2	12.93637256	0.584244004	0.376460813	1.551938431	0.120676956	0.183250193
PIEZO1	3.484788381	-1.116197255	0.719677645	-1.550968356	0.120909267	0.183582105
TIAM2	2.121945107	1.555573145	1.003425985	1.55026197	0.121078651	0.183818407
FITM1	2.493617609	1.306815386	0.843296027	1.549652012	0.121225062	0.184019782
TMX1	53.33187966	-0.364472605	0.235253797	-1.54927406	0.121315853	0.18413669
POP4	8.889716829	0.716050131	0.462216455	1.549166247	0.121341761	0.184155102
SNPH	2.201622291	-1.448194477	0.935023614	-1.548831981	0.121422115	0.18425613
NEBL	4.564542504	0.947661845	0.611885327	1.548757264	0.121440082	0.184262475
KLF13	45.43890747	-0.317434662	0.205145801	-1.547361245	0.121776161	0.184751438
TNPO3	125.4742548	0.322529371	0.208503553	1.5468771	0.121892884	0.184907535
SNN	6.26698496	-0.850867445	0.550092306	-1.546772125	0.121918204	0.184924957
LOC105602957	3.677182662	-1.117022433	0.722643306	-1.545745214	0.122166113	0.185279957
PIGY	20.66274139	-0.577770591	0.373893984	-1.545279186	0.122278748	0.185429742
P3H1	4.08007113	0.995247896	0.64467481	1.543798331	0.122637196	0.185910033
PSMA5	48.50336284	0.317124679	0.205411509	1.543850588	0.122624533	0.185910033
TSPYL1	27.53203827	0.443663078	0.287363764	1.543907526	0.122610737	0.185910033
LYN	196.6480512	0.20893322	0.135365283	1.543477137	0.12271505	0.186006959
MRPL40	47.58065975	-0.338821672	0.219574241	-1.543084792	0.122810204	0.186130081
LOC114110324	11.36986082	0.636822698	0.41278424	1.542749543	0.122891556	0.186232261
DENND4C	26.20342224	0.399265839	0.258820487	1.542636149	0.122919082	0.186252857
LOC101111229	1.914018796	-1.587253731	1.029895349	-1.541179629	0.123273074	0.186768069
DCLRE1A	10.12823938	0.661771012	0.42943667	1.541021199	0.123311627	0.186805305
LRRIQ1	7.204392375	-0.853873401	0.5543385	-1.54034656	0.1234759	0.187032965
ZNF200	6.384789374	0.774935036	0.503166984	1.540115034	0.123532316	0.187097216
HARS1	14.73777569	-0.604057139	0.392317362	-1.539715539	0.123629707	0.187223507
HIRIP3	47.0599134	0.413559685	0.268651356	1.539391765	0.123708683	0.187307815
TSG101	31.22850229	0.426522316	0.277075459	1.539372408	0.123713406	0.187307815
LOC105609918	286.5042968	0.220788703	0.143435863	1.539285215	0.123734682	0.187311644
RNF125	6.916215994	-0.865158584	0.562066051	-1.539247179	0.123743964	0.187311644
LOC132657286	3.028600613	1.161863287	0.754856072	1.53918519	0.123759092	0.187313331
WDR26	45.83917189	-0.411633361	0.267452819	-1.539087763	0.123782872	0.18732811

IRAK2	1.655508208	-1.582582634	1.028322234	-1.538994862	0.123805551	0.187341219
SNX22	2.384676006	-1.252726776	0.815408291	-1.536318419	0.124460304	0.188310666
REEP6	18.59314327	-0.497759615	0.324010812	-1.53624384	0.124478587	0.188317012
BRF1	3.720526438	1.110741278	0.723205512	1.535858424	0.124573107	0.188438677
LOC101114249	38.33343717	-0.393765166	0.256502213	-1.535133605	0.124751013	0.188686437
JMJD4	8.155541476	0.667509181	0.434885111	1.534909255	0.12480612	0.188748427
YLPM1	171.5775327	0.236380227	0.154044417	1.534493961	0.124908178	0.188881401
CLN8	5.957166661	-0.825402973	0.5381102	-1.533892078	0.125056205	0.189083851
GPR161	3.774944304	-1.044345443	0.680925748	-1.533714133	0.125099995	0.189128666
LOC132657481	4.139838863	-0.957863814	0.624650325	-1.533440031	0.125167472	0.189209277
RNF40	21.09134964	-0.442957184	0.288954482	-1.532965264	0.125284414	0.189364636
POU2F3	5.388113619	-0.866109705	0.565104466	-1.532654149	0.125361092	0.189459109
AIRIM	11.28332213	-0.706391521	0.461079949	-1.532036954	0.125513316	0.18966772
SS18L2	20.70976591	0.576228399	0.376228603	1.531591153	0.125623357	0.189812547
RAC2	20.94281825	0.433935152	0.28338694	1.531246121	0.125708576	0.18991984
NAPRT	11.63823424	0.620397064	0.405271002	1.530820267	0.125813819	0.190057359
CHPF	9.189831035	-0.716081543	0.467867751	-1.530521269	0.125887753	0.190147554
PARD6B	5.507118857	-0.954051711	0.623600227	-1.529909179	0.126039211	0.190354813
ALKBH7	10.45418228	0.610680464	0.399340221	1.529223533	0.126209038	0.190589765
GLO1	24.29651831	-0.464840422	0.304082246	-1.52866676	0.126347077	0.190776664
DDX11	3.353567157	-1.062103806	0.694967498	-1.528278385	0.126443434	0.190900592
LOC114118232	3.722729997	-1.003359509	0.656956086	-1.527285506	0.126690032	0.191251295
STAT6	17.57936808	0.489797322	0.320794156	1.526827449	0.126803924	0.191401609
ANGEL1	9.892106652	-0.611879692	0.400981761	-1.525953926	0.127021339	0.191708133
RESF1	59.87883102	0.306564226	0.20093663	1.525676161	0.127090534	0.191790909
PLEKHB1	5.947810193	0.794355464	0.520708436	1.525528316	0.127127377	0.191824849
GSPT1	133.1822877	0.232991005	0.152813597	1.524674569	0.127340288	0.192124426
KANSL3	98.12791642	-0.310546991	0.20372418	-1.524350182	0.127421258	0.192224891
CLDN3	49.3564057	-0.331931924	0.217763725	-1.524275563	0.127439889	0.192231301
LOC121818850	13.13104558	0.682110892	0.447841312	1.523108461	0.127731572	0.192649537
LOC114115410	4.169824919	1.117213879	0.733695638	1.52272117	0.127828479	0.192773943
PSMF1	22.78611103	-0.426582969	0.280204615	-1.522398082	0.127909365	0.192874162
GLI4	16.97505929	-0.519960526	0.341650401	-1.521908139	0.1280321	0.193026994
LOC121816265	2.630426075	-1.186686728	0.779751447	-1.5218782	0.128039602	0.193026994
PMS1	17.68503395	0.564705667	0.371456677	1.520246375	0.128449066	0.193622445
CROCC	2.839239416	-1.127475654	0.741725995	-1.520070299	0.128493308	0.193667294
UBXN1	96.35114797	-0.305461518	0.200994537	-1.519750352	0.128573731	0.193766658
TOP2A	326.7954003	-0.292826479	0.192734482	-1.519325845	0.128680497	0.193905696
LOC101102122	6.413964319	-0.771328231	0.507791607	-1.518985782	0.128766075	0.194012778
GRAMD1A	8.713706893	-0.744381129	0.490088139	-1.518871954	0.12879473	0.19403408
LOC105607297	4.703809819	0.919275724	0.60537873	1.518513418	0.12888502	0.194148222
MYG1	7.170871119	-0.749904589	0.493903523	-1.518322009	0.128933242	0.194198976

SDF2	35.13527261	-0.415346446	0.273625044	-1.517940171	0.129029482	0.194322034
TMEM260	16.26715931	0.533812267	0.351686791	1.517862713	0.129049012	0.19432955
GNB1L	15.82040117	-0.579829352	0.382061628	-1.517633044	0.129106932	0.194394869
XK	10.92239713	-0.665748027	0.439097013	-1.516175257	0.129475043	0.194927171
BTBD3	15.71313028	-0.504838418	0.333042787	-1.515836515	0.129560696	0.195034156
ATG9A	15.36435153	0.491069564	0.324004636	1.515625116	0.129614173	0.195070716
PSMA2	63.08791752	-0.330843907	0.2182829	-1.515665707	0.129603903	0.195070716
LOC132658375	2.413230351	-1.319533764	0.871047242	-1.514881973	0.129802296	0.19533185
TMEM225B	1.911387333	1.399064747	0.92442967	1.513435572	0.130169055	0.195861712
HAGHL	5.304538663	0.912743883	0.603191622	1.513190584	0.130231255	0.195932771
LZTS2	6.509556107	-0.827854954	0.547112746	-1.513134103	0.130245599	0.195932771
CIAO2B	7.844180012	-0.674713	0.446180338	-1.512197968	0.130483508	0.196268576
POT1	10.55362346	0.642903007	0.425186705	1.512048705	0.130521473	0.19630359
MAFF	3.116620159	1.140029329	0.754052737	1.511869492	0.130567067	0.196350069
IFNAR2	3.452979597	-1.064526036	0.704221719	-1.51163477	0.130626801	0.1964178
CRTC1	24.48736528	0.423859773	0.280454188	1.511333369	0.130703536	0.196511076
IL27RA	22.00605233	0.438886448	0.290435214	1.511133729	0.130754383	0.19651643
MTRES1	10.27058665	-0.590192214	0.390574225	-1.511088486	0.130765908	0.19651643
RNF146	9.147367594	0.642810541	0.425389969	1.511108838	0.130760723	0.19651643
SIDT1	19.73132298	-0.528848397	0.34996604	-1.511142045	0.130752265	0.19651643
RPRD1A	21.11006109	-0.43485795	0.287979551	-1.510030657	0.131035602	0.19689959
ETFBKMT	29.61689085	0.377915554	0.250328319	1.509679589	0.131125202	0.197012078
RLIM	108.4182939	-0.257720001	0.170895282	-1.508058026	0.131539677	0.197612602
ABHD3	2.142598755	1.401561576	0.929578564	1.5077387	0.131621417	0.19769096
BET1	4.180138652	0.945258389	0.62693008	1.507757276	0.13161666	0.19769096
ABCB9	4.901396995	-0.868512583	0.576162532	-1.507408995	0.131705855	0.197773335
ISCA1	51.61055219	-0.326136485	0.216347961	-1.507462716	0.131692094	0.197773335
MED14	10.40100776	-0.612840962	0.406862721	-1.506259804	0.132000493	0.198193505
CSK	25.77430575	0.389321349	0.258502698	1.506062999	0.132051002	0.198247073
UBTD1	3.150064605	-1.259124371	0.83626326	-1.505655493	0.132155635	0.198381875
DPP3	8.316139418	-0.689839795	0.458238276	-1.505417227	0.132216843	0.198451468
NOTCH1	15.90228193	-0.555906529	0.36934894	-1.505098484	0.132298759	0.198552123
PRSS35	1.901915907	1.560007293	1.037085179	1.504222916	0.132523979	0.198867801
UNG	47.21776297	-0.515790785	0.342934906	-1.50404866	0.132568838	0.198912785
PXMP2	5.647122063	-0.85008705	0.565236733	-1.503948701	0.132594576	0.198929072
EMC3	60.55115648	-0.31539961	0.20976216	-1.503605848	0.132682885	0.199039218
OTUD1	4.22299802	0.955635061	0.635725645	1.503219302	0.132782502	0.199166302
LOC101102857	2.708045766	-1.189396732	0.791473768	-1.502762037	0.132900419	0.199320803
OARD1	98.13215076	-0.248112388	0.165115508	-1.502659505	0.13292687	0.199338107
NDFIP2	58.95263319	0.345760666	0.230115797	1.502550758	0.13295493	0.199357818
SEC61A2	7.653442429	-0.728268426	0.484772051	-1.502290458	0.133022112	0.19943618
CRLS1	11.39039957	0.594507565	0.395795839	1.502056127	0.133082614	0.199504511

LOC132659996	1.737401466	1.738961424	1.158065003	1.501609512	0.133197985	0.199655072
PXK	9.943434824	0.593030912	0.395034215	1.501214046	0.133300208	0.199785892
DMAC2L	40.75481649	-0.393075955	0.261985862	-1.500370867	0.133518362	0.200090416
PLOD2	2.548088885	1.267674679	0.845162218	1.499918776	0.133635444	0.200243424
FMO2	1.87497847	1.496606073	0.998157164	1.499369164	0.133777888	0.200434397
NCR3	5.119415392	-0.945901171	0.630961993	-1.499141282	0.133836984	0.200500463
HHEX	23.1911221	-0.491847334	0.328158661	-1.498809547	0.133923047	0.200606909
MOCS3	5.977497795	-0.83651437	0.558228067	-1.498517218	0.133998923	0.200698073
NCBP1	39.52118883	-0.350555411	0.234022717	-1.497954627	0.134145039	0.200894408
DNAJC8	177.0806799	-0.298648866	0.199545994	-1.496641753	0.1344865	0.201364548
NXT1	7.453262404	0.706748576	0.472226097	1.496631761	0.134489101	0.201364548
ARMC9	3.638909568	-1.059579965	0.708064061	-1.496446471	0.134537347	0.201414223
TRIM27	17.63086162	0.476236818	0.318266863	1.496344333	0.134563948	0.201431485
HEMK1	39.23795419	0.342389856	0.228904747	1.495774379	0.13471246	0.201631214
CSTF3	43.37696976	-0.335671223	0.224477465	-1.495344855	0.134824464	0.201776261
LOC101110178	14.12761958	-0.500636479	0.334949484	-1.494662636	0.13500251	0.202020102
KBTBD8	5.954102376	0.854820401	0.572260086	1.493762052	0.135237823	0.202349574
FOXP1	295.0524458	0.233730495	0.156496895	1.493515227	0.135302371	0.202411798
KCTD20	48.97393563	0.322775026	0.21612172	1.493487219	0.135309697	0.202411798
LONRF3	15.32179472	0.481350926	0.322428154	1.492893595	0.135465042	0.202621503
LOC114113906	9.846569981	-0.633553009	0.424482207	-1.492531367	0.135559901	0.2027407
GCNT3	201.9907556	0.359785855	0.241085482	1.492358031	0.135605311	0.202763238
ZNF567	8.469129147	-0.64047008	0.42915277	-1.492405793	0.135592797	0.202763238
MBTPS2	17.3846762	-0.490839449	0.328996103	-1.491930891	0.135717264	0.202907936
PELP1	7.070597424	-0.744572023	0.499376011	-1.491004788	0.135960238	0.203248466
RNF169	29.76703826	-0.378385521	0.253795614	-1.490906463	0.135986054	0.203264326
ZFAND5	36.85594478	-0.370945826	0.24885213	-1.490627488	0.136059323	0.203351103
TEPSIN	9.679296533	0.707752775	0.474906702	1.490298561	0.136145751	0.203457525
LOC105601988	31.6800105	0.366637054	0.246046328	1.4901139	0.13619429	0.203507309
VCPIP1	27.62680247	0.428549851	0.287607317	1.490051978	0.13621057	0.203508884
BCL10	9.768608214	0.603394211	0.405076152	1.489582162	0.136334135	0.203670733
ASPHD2	1.739586479	1.435000429	0.96372352	1.489016715	0.136482967	0.203870288
C12H1orf116	31.67617239	0.422538486	0.283923295	1.488213518	0.136694593	0.204163586
ERGIC2	74.59278824	-0.256378106	0.172295722	-1.488012019	0.136747724	0.20422012
LOC105602567	10.07135696	0.583430983	0.392224694	1.487491715	0.13688499	0.204402276
COQ9	11.6295972	0.665338261	0.447361317	1.487250318	0.136948711	0.204431729
PLXNB1	9.229857381	-0.630903787	0.424210244	-1.487243167	0.136950599	0.204431729
VSIG2	2.198361096	-1.439597894	0.967908838	-1.487327977	0.136928209	0.204431729
DVL1	52.59706297	-0.39557096	0.266049345	-1.486833052	0.13705891	0.204570562
MAGI3	34.75928663	0.351094294	0.236197514	1.486443648	0.137161812	0.204701293
LOC114111284	4.783647459	-0.892298207	0.60085477	-1.485048053	0.137531095	0.205206589
TMEM86A	2.25905047	1.285791789	0.865801841	1.485087843	0.137520556	0.205206589

DCK	2.109851623	1.358367485	0.914849053	1.484799575	0.137596925	0.205281895
PAK6	2.90808396	1.336815521	0.900676097	1.484235592	0.137746431	0.205482008
IFT43	2.625714884	1.212461361	0.81702877	1.483988576	0.137811951	0.205556806
STAMBP	20.76967619	0.43031574	0.290015312	1.483769033	0.137870205	0.20562075
MAP6D1	2.383911898	1.249683285	0.842322545	1.483616095	0.137910797	0.205658341
ZNHIT6	170.0404283	0.303919804	0.204991502	1.482597089	0.138181491	0.206039024
LGALS8	15.45880522	-0.522403518	0.352444507	-1.482229138	0.138279336	0.206161919
NTRK2	2.037382401	1.571271577	1.060180677	1.482079056	0.138319261	0.206198443
GET3	8.652094154	-0.722312674	0.487427673	-1.48188688	0.138370397	0.206205678
TMEM185A	3.896531667	-1.096240076	0.739712385	-1.481981507	0.138345216	0.206205678
UBE2W	25.63856622	0.376964893	0.254378114	1.481907728	0.138364849	0.206205678
TRIM44	19.86275196	0.43700789	0.295068114	1.481040716	0.138595724	0.206518445
ALG9	41.03956913	-0.343873336	0.232252	-1.480604416	0.138712018	0.206652884
USP34	109.9568701	0.263729524	0.17812507	1.480586222	0.138716869	0.206652884
TMEM199	20.12725078	-0.45081078	0.304528506	-1.480356587	0.138778109	0.206721075
PPP1R15A	14.76638925	0.550225391	0.371699381	1.480296766	0.138794066	0.206721806
EVC	4.826091595	0.859367575	0.580772095	1.479698461	0.138953736	0.206936562
CELF4	15.31331531	-0.484342241	0.327349795	-1.479586204	0.138983711	0.206958141
DSC1	3.932415415	0.97217446	0.657424062	1.478763125	0.139203634	0.207257978
LOC443128	12.6109856	0.57514307	0.388947469	1.478716577	0.13921608	0.207257978
ZNF395	77.70783858	-0.319845429	0.216370513	-1.478230211	0.13934617	0.207428546
DNM2	26.58367798	0.431028552	0.291597039	1.478165052	0.139363606	0.207431398
NUP210	34.72790449	0.368769177	0.249550832	1.477731712	0.139479603	0.207580934
NFE2L1	155.3043061	0.267706969	0.181226723	1.477193675	0.139623729	0.207772296
LOC105603811	2.620162832	1.17094665	0.793442135	1.475780776	0.140002753	0.208313125
CEP57L1	12.03323532	0.626418527	0.424621399	1.475240129	0.140147996	0.208506024
IER5	151.1126357	0.273717285	0.185577887	1.474945587	0.140227173	0.208579295
TMEM150A	2.434958021	-1.480373126	1.003683072	-1.474940813	0.140228456	0.208579295
OTUD3	25.09108584	-0.384081893	0.260469017	-1.474578042	0.140326022	0.208701192
THAP12	22.18516001	-0.471896798	0.32007725	-1.474321584	0.140395027	0.208780588
COPA	24.60009987	0.480224762	0.325887884	1.473588881	0.140592317	0.209050719
SOX13	1.947086618	-1.413912878	0.959853474	-1.473050748	0.140737352	0.209243098
MARVELD3	9.483147714	0.623105563	0.423031632	1.472952646	0.140763805	0.209249322
VHL	30.81519049	-0.422762728	0.287023724	-1.472919106	0.14077285	0.209249322
NDUFA9	72.67741406	-0.319581071	0.217003182	-1.472702233	0.140831344	0.20928972
SLC37A3	23.38606027	0.3896278	0.264557858	1.472750813	0.14081824	0.20928972
YPEL2	48.2178171	0.303496396	0.206090736	1.472634832	0.140849527	0.209293472
CHMP1B	23.72436558	-0.417594836	0.283626442	-1.472340989	0.14092882	0.209388018
HPRT1	20.49366185	-0.464790983	0.316032128	-1.470708012	0.141370098	0.210020311
EMC2	96.04162832	0.329095706	0.223848388	1.470172326	0.141515088	0.210212343
LOC105606001	3.93715863	-1.081484015	0.735816734	-1.469773606	0.14162308	0.21032601
MBD5	2.551771521	1.211043103	0.823937193	1.469824537	0.141609282	0.21032601

EPB41L1	18.65905823	0.436255598	0.296836239	1.469684426	0.141647243	0.210338523
CHDH	12.7600984	-0.551510949	0.375482899	-1.468804442	0.141885839	0.21066942
SRSF1	26.2790692	-0.439645819	0.299373107	-1.468554816	0.141953578	0.210746587
GRINA	23.79342043	-0.430939908	0.293531998	-1.468119014	0.142071898	0.210898821
ADAM17	32.39978482	-0.413456432	0.281800913	-1.467193377	0.142323458	0.211248789
RPS6KA4	8.351843488	-0.683335978	0.465864433	-1.466812939	0.142426949	0.211378925
SH3GL2	12.23082936	-0.518654711	0.353876509	-1.465637582	0.142747047	0.211830469
IFT172	2.503520178	1.19799736	0.817565286	1.465323175	0.142832767	0.211923262
LOC132657287	29.44137525	-0.378453972	0.258283724	-1.465264501	0.142848768	0.211923262
PLEKHG2	4.092388871	-0.922351126	0.629490757	-1.465233787	0.142857145	0.211923262
RHBDD2	16.1255175	-0.493381122	0.337018315	-1.463959375	0.143205049	0.212415789
SLC35A2	25.15849931	-0.429438027	0.293413631	-1.463592631	0.143305287	0.212540887
FAM219B	24.65399719	-0.390152613	0.266749841	-1.462616105	0.143572454	0.212913505
HDAC4	28.32973704	0.488566046	0.334166215	1.462045007	0.143728877	0.213121829
CUL3	34.94361546	-0.330343599	0.225974621	-1.4618615	0.143779167	0.213131653
DGCR8	27.06346289	-0.583930416	0.39942794	-1.461916801	0.14376401	0.213131653
SOD2	7.480416397	-0.650878515	0.445244158	-1.461846278	0.143783339	0.213131653
PIM2	8.295605508	-0.651581766	0.446128151	-1.460526006	0.144145566	0.213644891
PPP1R21	12.09776759	0.538136609	0.368548279	1.46015228	0.144248227	0.213773345
XYLT2	12.2169593	-0.594429173	0.407262549	-1.459572392	0.144407631	0.213985854
DOLK	7.115656171	0.750887247	0.514487765	1.459485139	0.144431628	0.213997688
ZNF706	21.78114729	0.503503055	0.345044163	1.459242348	0.144498417	0.214072915
UGT8	29.36750836	-0.452921035	0.310465725	-1.45884392	0.144608071	0.214211623
LRRC75A	4.846437821	-0.868459496	0.59568782	-1.457910447	0.144865229	0.214568777
ALDH18A1	22.97070844	-0.555166646	0.380938803	-1.457364391	0.145015821	0.214761827
VPS28	14.35262174	0.523166561	0.358991905	1.457321334	0.145027701	0.214761827
HOPX	18.28078262	0.639404437	0.438771892	1.457259337	0.145044807	0.214763364
ZSWIM7	23.78389324	0.419411783	0.287865168	1.45697302	0.145123828	0.214856567
SNX13	19.98606493	0.469166207	0.322184934	1.456201573	0.145336905	0.215148197
RFX5	6.348900839	0.716595595	0.492572015	1.45480371	0.145723611	0.215696764
LOC114118096	3.029644791	-1.013461953	0.69682961	-1.454389908	0.145838236	0.215842528
PKIG	12.13431899	0.610557327	0.419878569	1.45412834	0.145910728	0.215925907
TMLHE	2.256584948	1.329972849	0.914661028	1.454060913	0.145929419	0.21592966
SMARCAL1	12.82566957	0.561051722	0.386065179	1.453256477	0.146152556	0.216235894
CARF	39.311698	0.33958887	0.23371296	1.453016853	0.146219075	0.216310366
IFIT2	16.62389086	-0.503391213	0.346518765	-1.452709822	0.146304339	0.216412549
CAPNS1	39.9068815	0.34014519	0.234382654	1.45123875	0.14671339	0.21698953
LOC105602949	2.951663705	-1.166836798	0.804054977	-1.451190318	0.146726872	0.21698953
RBM46	4.006378062	0.971170383	0.669469125	1.450657464	0.146875265	0.217184954
LOC114116488	1.651774859	1.493188809	1.029617871	1.45023591	0.146992744	0.217334626
PARP6	10.25585888	-0.623176805	0.430135029	-1.448793433	0.147395278	0.217905684
KIF13A	100.5646275	0.290849309	0.200760918	1.448734701	0.147411686	0.217905839

ZNF81	7.730667288	0.702900963	0.485513731	1.447746825	0.147687869	0.218289954
LOC132658306	16.86587895	0.474845094	0.328091129	1.447296352	0.147813941	0.218452137
RAPGEFL1	8.433170975	0.655413825	0.452926546	1.447064279	0.147878921	0.218524009
TTC6	3.080017169	1.241468259	0.858007964	1.446919273	0.147919535	0.218559861
MINPP1	8.934608203	-0.636199742	0.439830469	-1.446465827	0.14804659	0.218723414
ARFRP1	28.95879098	0.386141053	0.266989367	1.446278766	0.148099029	0.218776705
UMPS	7.597213328	-0.654430935	0.45256091	-1.446061559	0.148159937	0.218842492
LOC106991294	3.278769646	1.150440546	0.795731259	1.445765179	0.148243076	0.2189411
SLC25A29	5.079846148	0.78923743	0.54602037	1.445435873	0.148335493	0.219053387
GID8	62.60864911	0.282495934	0.195527628	1.444787815	0.148517495	0.219297927
MPHOSPH8	299.063662	0.252756477	0.174986787	1.444431784	0.148617555	0.219421434
LOC132659437	5.356372315	0.902580345	0.624897615	1.444365162	0.148636285	0.219424849
PLOD1	9.506275043	0.780205437	0.540659224	1.443063214	0.149002665	0.219941427
PPM1L	22.07085371	-0.508504716	0.352410213	-1.442934107	0.149039034	0.219970818
GCH1	19.68008223	-0.450646604	0.312503389	-1.442053497	0.149287282	0.220312887
DGAT2	7.691821183	0.717577712	0.497860251	1.441323565	0.149493293	0.220592554
LIMD1	13.17425074	0.526377737	0.365321391	1.440862075	0.149623652	0.220760541
ATP6V1A	16.96680149	0.524855088	0.364283803	1.440786233	0.149645084	0.220767792
TMPO	51.60333499	0.318065234	0.220830614	1.440313135	0.149778827	0.220940713
ATP9B	22.91496848	-0.410543837	0.285457857	-1.438194209	0.150378958	0.221801495
FBXO4	2.813638963	-1.164919963	0.811218843	-1.436011963	0.150998937	0.22269136
SLC25A34	3.516603578	1.035729086	0.721512157	1.435497762	0.151145306	0.22288263
HHAT	19.91594506	-0.434902309	0.303090673	-1.434891761	0.151317944	0.223106924
SHLD3	5.550600871	-0.759901499	0.529626283	-1.434788119	0.151347485	0.223106924
SMPD4	23.92366084	-0.407102409	0.283733941	-1.434803351	0.151343143	0.223106924
LOC101102976	2.185659409	-1.279524531	0.891933606	-1.434551319	0.151414995	0.22315722
MAP2K5	8.585774827	-0.611259359	0.426093847	-1.434565092	0.151411068	0.22315722
RDH10	34.57158878	0.381665344	0.266075201	1.434426592	0.151450564	0.223185031
ITGB7	5.815976118	-0.791510922	0.552026481	-1.433827814	0.151621406	0.223387534
TK2	10.11227163	0.674377901	0.470323557	1.4338595	0.151612361	0.223387534
CDR2L	5.643557825	-0.808911418	0.564356789	-1.43333337	0.15176259	0.2235709
CCDC125	41.60743013	-0.329304689	0.229827826	-1.432832113	0.151905822	0.223757242
ATF3	5.330881712	0.805676835	0.562450701	1.432439918	0.152017961	0.223897749
LOC105607837	2.143694337	1.371898972	0.958016071	1.432020833	0.152137859	0.22404965
PRCC	6.045620211	0.748088303	0.52256883	1.431559365	0.152269966	0.224219496
TUB	1.425611061	1.531593391	1.070249773	1.431061635	0.152412551	0.224404733
AMN1	2.643969983	-1.068097698	0.746554629	-1.430702666	0.152515449	0.2245315
RUSF1	5.596846002	0.793400938	0.555246011	1.428917854	0.153027845	0.225261033
DNAAF3	2.037276708	1.37274591	0.960799336	1.428754016	0.153074947	0.225288748
PPID	41.40619	0.338520688	0.236937335	1.428735105	0.153080384	0.225288748
HPS3	3.587027141	1.002336709	0.701605991	1.428631913	0.153110057	0.22530761
ZNF311	19.26876715	-0.443666563	0.310611348	-1.428365594	0.153186657	0.225395515

FKBP9	41.25925314	-0.396576133	0.277836342	-1.427373146	0.153472367	0.225791047
TRAPPC2L	43.00368617	0.30729328	0.215334537	1.427050598	0.153565311	0.225902921
PARD6G	1.622101699	1.443916474	1.011939223	1.426880628	0.153614306	0.225950127
PACS1	56.71901165	0.27806562	0.194922927	1.42654137	0.153712134	0.226069144
C11H17orf80	27.9919348	0.383453496	0.268851045	1.426267459	0.153791154	0.226160476
CERS1	0.833070371	-2.041941827	1.432350951	-1.425587651	0.153987403	0.226424161
EIF4B	197.7357465	-0.230504491	0.161709051	-1.425427268	0.15403373	0.226442459
LOC101115420	4.386196884	0.913618308	0.640928091	1.425461484	0.154023846	0.226442459
YEATS2	9.168583389	-0.612203006	0.429603265	-1.425042721	0.154144852	0.226580897
EPRS1	157.8792102	-0.233161526	0.163691049	-1.424399976	0.154330721	0.226829164
SLC9B2	1.367976798	1.566118104	1.100049171	1.42368009	0.1545391	0.227110457
MSI2	8.238652765	0.619900509	0.435588648	1.423132837	0.154697651	0.22731847
GTF2A1	40.1398408	-0.421255026	0.296080351	-1.422772652	0.154802072	0.227446904
CFAP141	1.656306158	1.47011446	1.033516371	1.422439452	0.154898717	0.227563888
FAM185A	78.39463572	0.258811554	0.181961451	1.422342768	0.154926769	0.227580085
LOC101120606	3.550269613	1.044410107	0.734452834	1.422024749	0.155019066	0.227690642
MRPS9	8.471403975	-0.622489578	0.437814387	-1.421811608	0.155080949	0.227756506
COG7	5.58774476	-0.900405408	0.633330436	-1.421699253	0.155113577	0.227779397
AQP3	8.643963637	-0.693071977	0.487666288	-1.421201329	0.155258238	0.227966782
MRPL23	23.04035263	0.46427332	0.326701207	1.421094599	0.15528926	0.227987285
DUS4L	11.98364239	-0.552399658	0.388900712	-1.42041308	0.155487457	0.228253196
SORBS3	3.739680121	0.997176068	0.702208569	1.420056822	0.155591139	0.228380317
ZFP62	42.67686179	-0.399962415	0.281824558	-1.419189364	0.155843816	0.228726084
IQGAP1	127.3026702	0.273228668	0.192541293	1.419065302	0.155879979	0.228728927
KLHL13	12.64753138	-0.546394696	0.385032388	-1.419087623	0.155873472	0.228728927
TSR2	15.00974006	-0.475449905	0.335072798	-1.418945102	0.155915022	0.228730126
ZNF565	6.575342275	0.704727876	0.496648758	1.41896635	0.155908827	0.228730126
TAF11	32.17165295	-0.384123534	0.270777545	-1.418594491	0.156017273	0.228855012
SUMO2	15.71484216	-0.621080166	0.437837879	-1.418516294	0.156040085	0.228863357
APMAP	92.30445455	-0.30078538	0.21206545	-1.418361077	0.156085373	0.228904663
AREL1	20.23032947	0.433861537	0.305944631	1.418104756	0.156160183	0.228978013
LOC105606185	4.934633059	0.886883213	0.625414657	1.418072319	0.156169652	0.228978013
LOC101109732	6.270766487	0.745777657	0.526399878	1.416751198	0.156555683	0.229518839
NMD3	30.22230383	0.343915652	0.242842138	1.416210772	0.156713803	0.229725454
GNPAT	18.925948	-0.459339998	0.324399268	-1.415971131	0.156783957	0.229803089
KIAA1671	79.41265632	0.279120672	0.197146494	1.41580338	0.15683308	0.229849884
SLC26A6	2.616799357	1.164385786	0.822459514	1.415736296	0.156852727	0.229853476
LOC121819196	7.475796944	0.672296509	0.475070496	1.415151047	0.157024214	0.230079549
KGD4	81.17211034	-0.25776796	0.182184618	-1.414872251	0.157105955	0.230174087
TTC23L	2.573195198	1.147873498	0.811454601	1.414587454	0.157189489	0.230271231
LOC101109964	32.22107356	-0.362394147	0.256216449	-1.414406248	0.157242657	0.230323874
KLC1	20.48408568	-0.413594412	0.292477298	-1.414107747	0.157330269	0.230426953

PRKCZ	2.982131516	-1.024625107	0.724635088	-1.413987707	0.157365512	0.230453318
PDE4C	2.471493674	1.219752107	0.862823674	1.413674826	0.1574574	0.230562622
FAM120C	31.39521667	0.399340957	0.282529827	1.413447073	0.157524313	0.230635334
RBBP5	22.77902654	-0.376634656	0.266644765	-1.412495969	0.157803977	0.231019491
LOC121816682	84.62793665	0.375607469	0.266017327	1.411966177	0.15795992	0.231222461
SPR	4.670094607	-0.859012926	0.608603681	-1.411448786	0.158112327	0.23142021
METTL18	7.961076507	-0.660107616	0.467768838	-1.411183394	0.158190545	0.231509343
RPRD2	19.47088713	-0.445452442	0.315742084	-1.410811116	0.158300316	0.231644627
KBTBD3	7.450362742	0.723419471	0.512876752	1.410513284	0.158388177	0.231747824
PLXNA2	5.859633469	-0.724798039	0.513918764	-1.410335815	0.158440548	0.231799077
LOC101108092	4.923596242	-0.872323852	0.61858415	-1.410194316	0.158482314	0.231834804
DUSP1	6.01135829	0.834546436	0.591920437	1.409896302	0.158570305	0.231938137
ARL6IP5	52.99665144	0.334812093	0.237517083	1.409633733	0.158647861	0.232026186
LOC105612199	7.22844665	0.698115685	0.495333793	1.409384329	0.158721555	0.232083176
ZHX2	2.156464961	1.225508215	0.869514481	1.409416682	0.158711994	0.232083176
LOC101107232	4.064036579	-0.913725728	0.648564072	-1.408844195	0.158881244	0.232291261
DNTTIP2	149.6301114	-0.277851975	0.197251484	-1.40861792	0.158948177	0.232363703
LOC132658781	2.577670753	1.10540203	0.785077198	1.408016986	0.15912604	0.232598278
LOC132657616	4.255872954	0.867657928	0.616299932	1.407850112	0.159175458	0.232619634
ZBED5	41.246195	0.303579906	0.215626669	1.407895914	0.159161893	0.232619634
BAG3	69.16893602	0.333318265	0.236827851	1.407428494	0.159300367	0.232776725
ARMC6	3.283933156	-1.057728194	0.751609568	-1.407284099	0.159343162	0.232813807
LOC101111060	27.30966802	-0.386724281	0.274944371	-1.406554642	0.159559491	0.2331044
HKDC1	27.64517189	-0.441481245	0.313900088	-1.406438744	0.159593882	0.233129162
MTHFD2	2.688448087	-1.128161141	0.802278733	-1.406195994	0.159665934	0.233208924
LOC101122717	1.945436301	-1.29783026	0.923273228	-1.405683844	0.159818026	0.233405566
OMA1	11.38791832	0.553189163	0.393627969	1.405360408	0.159914134	0.23352041
AEBP2	40.81561766	0.31478006	0.224036539	1.405038935	0.160009701	0.23363444
PPP1R1A	2.166341956	1.264611663	0.900842724	1.403809599	0.160375555	0.234117482
SENP1	7.701961659	0.614083658	0.437427122	1.403853642	0.160362437	0.234117482
AS3MT	11.00925624	-0.562052388	0.400530633	-1.403269418	0.160536514	0.234296771
EVC2	3.592784927	-0.908188471	0.647217008	-1.403220958	0.16055096	0.234296771
RIDA	5.31722723	0.768089716	0.547342924	1.403306195	0.160525552	0.234296771
CTNNBIP1	15.67448654	-0.478119983	0.340810958	-1.402889114	0.160649907	0.234415575
LOC101120701	3.631200697	-0.950103143	0.677285247	-1.402810925	0.160673228	0.234424012
IFT88	15.54899532	0.456481168	0.325476947	1.402499231	0.16076622	0.234528777
RBM39	256.3544421	0.254832812	0.181705112	1.402452627	0.160780127	0.234528777
TIMM10B	61.85730765	-0.252331231	0.179953712	-1.402200753	0.160855307	0.234612836
PPA2	37.08216376	-0.362720701	0.258741241	-1.401866591	0.160955089	0.234732756
WDCP	2.884379675	-1.102012843	0.786148531	-1.401787066	0.160978842	0.234741785
SLC18B1	6.442632896	-0.79623017	0.568161612	-1.401414937	0.161090028	0.234857995
ZFP91	239.4908177	0.195675245	0.139628132	1.401402726	0.161093678	0.234857995

LOC114114914	1.909915794	1.421788442	1.014636528	1.401278588	0.161130781	0.234886468
LRRC8D	58.43082226	-0.325582599	0.232409715	-1.400899268	0.161244198	0.235026166
PLEKHA4	11.37247231	-0.593838683	0.423936279	-1.400773446	0.161281832	0.235055388
RYBP	42.60037636	-0.315776612	0.225547666	-1.400043804	0.161500201	0.235347982
LOC121819259	2.726979975	-1.057240127	0.756388423	-1.397747631	0.162188865	0.23632578
LOC101111868	5.70987405	0.82326808	0.589778306	1.395894138	0.162746376	0.237112281
FER1L6	2.797285952	1.097725785	0.786455792	1.39578829	0.162778258	0.237132882
FHDC1	8.028769235	0.708594273	0.508190645	1.394347338	0.163212742	0.237739921
IL13RA1	13.89800916	0.503277942	0.360962201	1.39426771	0.163236778	0.237749022
PAPOLA	194.9015855	-0.211735137	0.151903697	-1.393877445	0.163354616	0.237894727
POLR2D	18.03095194	-0.476631253	0.341987461	-1.393709731	0.163405276	0.237942578
NHLRC3	12.98269633	0.545120537	0.39132098	1.393026607	0.163611745	0.238217275
PHF1	10.1384215	0.634729264	0.455700077	1.392866265	0.163660235	0.238261922
EPCAM	465.567882	0.224569977	0.161366385	1.391677556	0.164020062	0.238759763
GALNT11	27.40155799	0.412198552	0.296277544	1.391258161	0.164147156	0.238918751
ATP7A	12.86317402	0.510525623	0.367043746	1.390912198	0.164252054	0.239045399
FSBP	11.26980475	-0.532790214	0.383068256	-1.390849295	0.164271131	0.239047136
AVIL	2.650363962	1.070416384	0.770195153	1.38979891	0.164589949	0.239478316
ZC3H8	46.30763745	-0.334643601	0.240793224	-1.389755061	0.164603268	0.239478316
GPAA1	35.65900302	0.346129808	0.249214126	1.388885189	0.164867665	0.239836875
PLEKHA5	168.2162209	0.241137598	0.173629829	1.388802825	0.164892716	0.239847213
VCL	21.78762271	0.440005015	0.31720334	1.387138656	0.165399487	0.240558165
CCT5	106.7387117	-0.250565892	0.1806584	-1.386959542	0.1654541	0.240611413
TRMT12	31.16165018	0.335861271	0.242273268	1.386291081	0.165658039	0.240881783
KATNA1	45.90324785	-0.301492136	0.217495183	-1.386201442	0.165685401	0.240895363
IL17D	6.708618134	-0.751461003	0.542319915	-1.385641542	0.165856387	0.241117736
PLEKHM3	4.398219905	0.862006544	0.622379292	1.385018036	0.166046953	0.241368523
FPGT	8.967510493	0.592463045	0.427801861	1.384900579	0.16608287	0.24139448
LOC101108113	5.155254425	0.879094182	0.634837508	1.384754635	0.166127507	0.241406855
NR2C1	10.37931936	-0.521571555	0.376647517	-1.384773642	0.166121693	0.241406855
ZSWIM9	12.18039046	-0.502459528	0.363073142	-1.38390718	0.166386878	0.241757475
FAM131A	3.722793262	-0.869830869	0.628616587	-1.383722426	0.166443464	0.241798471
PRAG1	31.86246359	-0.385887938	0.278881834	-1.383696929	0.166451274	0.241798471
TMEM170A	15.80504851	0.442331513	0.319769437	1.383282644	0.166578218	0.241956581
GFOD2	10.48974485	-0.788580294	0.570217923	-1.38294547	0.166681587	0.242080418
INPPL1	98.26927317	-0.655365319	0.474246366	-1.381908997	0.166999645	0.242515999
ARHGAP9	16.61915987	0.536641923	0.388352416	1.381842625	0.167020028	0.24251925
LOC132657800	4.964184444	-0.793800508	0.574740445	-1.381146071	0.167234052	0.242803644
LOC101117055	10.76860932	-0.531794281	0.385122376	-1.38084493	0.167326645	0.242911691
ABRAXAS1	38.2721107	-0.39312929	0.284727085	-1.380723192	0.167364088	0.242939661
CCDC146	2.81790873	-1.057800764	0.766262082	-1.380468626	0.167442403	0.243026948
AHDC1	2.706271792	-1.246543736	0.903693151	-1.379388274	0.167775073	0.243483346

LOC105601865	2.744968138	-1.028968719	0.746091752	-1.379145013	0.167850048	0.243565708
LIN9	6.50833058	-0.686404193	0.497795065	-1.378889108	0.167928947	0.243653746
DDX52	88.2879977	-0.238146586	0.172834676	-1.37788661	0.1682383	0.2440761
LOC121818754	11.9270166	-0.591827108	0.42987867	-1.376730573	0.168595563	0.244567863
LOC132657734	7.712369529	0.665971799	0.483782266	1.37659407	0.168637785	0.244602565
RNLS	8.928760629	-0.640324243	0.465215362	-1.37640391	0.168696618	0.24466135
GSE1	100.2397306	0.241275518	0.175412076	1.375478381	0.168983184	0.245023783
LOC101118849	7.409702861	-0.64010849	0.465360264	-1.375511705	0.168972859	0.245023783
LOC106990434	4.11963687	-0.931614662	0.677877973	-1.374310272	0.169345379	0.245522327
ZBTB38	170.1938857	-0.234561523	0.17069195	-1.374180349	0.1693857	0.24555415
LOC101116299	28.84252454	0.549183826	0.399885677	1.37335208	0.169642919	0.2458737
SIRT1	16.68248822	0.42617059	0.310312502	1.373359397	0.169640646	0.2458737
PRDM2	32.5334269	0.343958191	0.250562644	1.372743303	0.169832162	0.246121292
CEP192	29.38777404	-0.355821579	0.259304647	-1.372214434	0.169996693	0.24630632
MPV17L2	1.780942687	1.333178551	0.97153622	1.37223762	0.169989478	0.24630632
DDX27	150.7737897	0.262517352	0.191356341	1.37187694	0.17010175	0.246431822
TRABD	110.7020971	-0.258302576	0.188337319	-1.371489076	0.170222546	0.246580097
SRGAP1	62.74256352	0.345538515	0.2519551	1.371428937	0.170241282	0.246580513
SNX27	32.64194113	0.330963554	0.241372447	1.371173713	0.17032081	0.246668973
GALK1	2.866503789	-1.074987724	0.784057877	-1.371056597	0.170357314	0.246695109
SPINT1	29.65462121	-0.437787891	0.319365235	-1.370806349	0.170435331	0.24678135
COASY	23.82351857	0.380132183	0.27736132	1.370530623	0.170521323	0.246879116
SEMA4F	2.014033004	-1.235048844	0.901351435	-1.37021898	0.170618555	0.246993134
STXBP2	21.3758437	0.460866595	0.336490746	1.369626357	0.170803568	0.247234187
CTDNEP1	41.08052314	-0.302632279	0.221129856	-1.368572677	0.17113289	0.24768405
NUDT14	10.76645971	-0.583079371	0.426143726	-1.368269286	0.171227801	0.247794585
LOC105612547	4.02365993	-0.915812436	0.669601473	-1.367697761	0.171406701	0.248026628
FAM20C	9.668571672	0.564382942	0.412810585	1.367171684	0.171571499	0.248238217
SNAP47	11.64251275	-0.504016175	0.368849429	-1.366455077	0.171796172	0.248536382
UMAD1	17.15124014	0.532959802	0.390151975	1.36603128	0.171929146	0.248701836
LMBR1L	5.466514026	-0.822615504	0.602232961	-1.365942348	0.171957059	0.248715297
IRF7	3.731975572	-0.997935785	0.73153938	-1.364158665	0.172517634	0.249499102
SMC3	265.1416481	-0.266961455	0.195746669	-1.363810972	0.172627066	0.249630355
BBS9	2.708185553	-1.062388576	0.779176217	-1.363476648	0.172732339	0.249701541
LOC105601981	2.91455687	0.991556076	0.727171441	1.363579509	0.172699945	0.249701541
SHB	8.522059343	0.626407126	0.459407749	1.363510144	0.17272179	0.249701541
ZFAND1	6.698371983	-0.699541286	0.513202302	-1.363090703	0.172853926	0.249850284
NRF1	10.09668888	0.556460082	0.408371389	1.36263239	0.172998396	0.250032067
ERP29	60.68761821	-0.28734796	0.210913233	-1.362398916	0.173072026	0.250111439
LYRM7	6.046225	0.714561407	0.524680833	1.361897296	0.1732303	0.250313101
LRRC51	4.151264537	0.937595786	0.688497826	1.361799197	0.173261265	0.250330782
CELF6	2.114675654	1.311973519	0.96354459	1.361611629	0.173320483	0.250389276

ILF3	72.45436364	-0.35813067	0.263058098	-1.36141283	0.173383264	0.250452902
ARL6IP1	97.59880975	0.268436868	0.197263358	1.360804512	0.173575476	0.250703459
ABCA3	28.25933115	0.389457572	0.286219113	1.360697292	0.173609371	0.250725321
LOC101115820	2.371127617	-1.113525005	0.818946138	-1.359704813	0.173923355	0.251151638
NPEPL1	12.93578613	0.478230146	0.351903669	1.358980279	0.174152838	0.251430866
OXCT1	79.88077236	0.23041951	0.169553834	1.358975527	0.174154344	0.251430866
LOC121817857	5.090429206	-0.81212398	0.597720411	-1.358702103	0.174241006	0.251528813
CLUAP1	19.3797003	-0.387829369	0.285525261	-1.358301423	0.174368059	0.251685041
LOC132657742	8.865076785	0.604716952	0.445283952	1.358047937	0.174448474	0.251773923
CGNL1	17.21086927	-0.500733237	0.368906181	-1.357345751	0.174671377	0.252041198
LOC114110426	6.088761798	0.705163464	0.519506815	1.35737096	0.174663371	0.252041198
TESK1	4.701632943	0.773810367	0.570376608	1.356665678	0.174887463	0.252325758
MAPK15	1.335438792	-1.574344611	1.160673628	-1.356405947	0.174970042	0.252390415
SAP25	4.307547264	-0.811351243	0.598160241	-1.356411188	0.174968375	0.252390415
NDC80	33.36197394	-0.405899009	0.299307719	-1.356126095	0.175059052	0.25249156
ATPAF1	54.27913682	0.305954359	0.225671883	1.355748687	0.175179143	0.252637508
NXPH4	6.398903011	0.698460407	0.515421588	1.355124473	0.175377903	0.252896866
SLC7A6OS	19.28583301	-0.397983075	0.293786837	-1.354666122	0.175523956	0.253080173
APTX	7.829529287	-0.594099494	0.438604265	-1.354522839	0.175569632	0.253118725
CTCF	266.0804207	0.241364976	0.178267546	1.353947934	0.175752989	0.253355744
NUDT15	11.37133011	0.503356684	0.371942113	1.353319956	0.175953437	0.253617344
AUH	11.1865533	0.526927959	0.389466881	1.352946772	0.176072636	0.253761791
LOC106991640	1.396233879	1.625525092	1.201918555	1.352441965	0.176233973	0.25396693
LOC101120042	20.51149732	-0.419107575	0.310103598	-1.351508264	0.176532674	0.254369958
CMC2	11.99859465	0.526169872	0.389379377	1.351303903	0.176598102	0.254436804
SHLD2	7.510468302	-0.643409626	0.476164596	-1.351233653	0.176620598	0.254441788
TOR1B	32.25924602	0.344889562	0.255253323	1.351165804	0.176642326	0.254445665
LOC121820693	2.643185492	-1.108959764	0.820853624	-1.350983576	0.176700694	0.254502314
SOX12	13.42759419	0.500484057	0.370683352	1.350165995	0.176962743	0.254852282
LOC105609211	3.793015383	-0.907404714	0.672335247	-1.349631331	0.177134269	0.255071821
ZBTB2	17.56541079	-0.464786337	0.344498669	-1.349167295	0.177283237	0.255258833
METTL14	13.13658489	-0.487104199	0.361201521	-1.348566301	0.177476311	0.255509303
SCCPDH	14.29937863	-0.502034977	0.372332794	-1.348350148	0.17754579	0.255581802
GNB2	89.93913557	-0.286678186	0.212832828	-1.346964131	0.177991785	0.256170392
LOC105602913	6.704997624	0.722775245	0.536597264	1.346960362	0.177992999	0.256170392
HEATR3	22.82277121	0.42656166	0.31671784	1.346819173	0.178038478	0.256180676
LOC121819405	2.387858014	1.199412253	0.890536534	1.346842277	0.178031036	0.256180676
GTF3C6	15.50031466	-0.43553145	0.32339293	-1.346756252	0.178058749	0.256182264
NPRL2	10.40365097	-0.612388002	0.454804237	-1.346487019	0.178145504	0.256279497
CPT1A	27.34175673	-0.343288712	0.255032329	-1.34605959	0.178283299	0.256422531
RANBP3	16.12098226	-0.460993059	0.342474515	-1.346065296	0.17828146	0.256422531
DDX18	78.8318849	-0.251293358	0.186760422	-1.345538613	0.178451361	0.256636635

GINS4	8.892851107	-0.590287077	0.438950358	-1.344769552	0.178699666	0.256949559
GRWD1	7.208929206	0.655468759	0.487442895	1.344708819	0.178719286	0.256949559
NCBP2AS2	6.905353868	-0.636119773	0.473061907	-1.344686105	0.178726624	0.256949559
LOC121818690	3.095155023	-1.062767606	0.790382764	-1.344623965	0.1787467	0.256950785
CFAP90	2.856912647	-0.982664454	0.730940128	-1.34438433	0.178824139	0.257034459
USP14	57.86964995	0.275092344	0.204650815	1.344203513	0.178882586	0.257090822
RBL2	8.725599842	0.578400329	0.430314165	1.344134996	0.178904737	0.257095013
TGFBRAP1	4.318843848	-0.808277046	0.60140685	-1.343977118	0.178955786	0.257140727
CTSA	41.59107989	0.31136472	0.23168472	1.343915648	0.178975665	0.257141647
STX16	27.63559052	-0.363922486	0.270862557	-1.34356882	0.179087858	0.257275183
ZMAT3	26.0624129	-0.361255481	0.269024281	-1.34283597	0.179325093	0.257588307
RNF115	46.26003646	-0.297082445	0.221358254	-1.34208885	0.179567189	0.257908343
DPP8	53.31800917	-0.324084664	0.241589977	-1.341465688	0.179769303	0.258170893
UNC50	23.97438276	0.509727135	0.380062436	1.341166838	0.179866291	0.258282428
KCNJ3	17.71734829	-0.474522643	0.353839086	-1.341069038	0.179898039	0.258300267
PTX3	2.852820951	1.009859388	0.753203797	1.340751856	0.180001032	0.258420386
KIF20B	186.8880909	-0.284943935	0.212604841	-1.340251398	0.180163627	0.258626038
RETREG3	3.26381635	0.933513804	0.696661793	1.33998134	0.180251411	0.258724266
LOC132660161	5.358682824	0.721825107	0.538892967	1.339459135	0.180421249	0.258940236
LYSMD1	4.589371524	0.762759683	0.569558681	1.33921176	0.180501745	0.259000142
NLRX1	2.349559556	-1.167668576	0.871872954	-1.339264591	0.180484552	0.259000142
VPS37D	1.534758692	-1.487761407	1.111277154	-1.3387852	0.18064061	0.259171575
ALMS1	50.64881645	0.324141547	0.242140125	1.338652763	0.180683741	0.259205633
SCFD1	23.01058263	0.383048004	0.286326821	1.337799939	0.180961662	0.259576472
BRPF1	6.53313824	-0.678414473	0.507236495	-1.337471731	0.181068704	0.259702146
LOC121819040	2.216192465	1.231623292	0.921040815	1.337208158	0.1811547	0.259797609
DUSP3	5.888205747	-0.706977354	0.528852242	-1.336814517	0.181283189	0.259953986
PPP1R15B	46.86897613	0.461288713	0.345122339	1.336594772	0.181354947	0.260001096
VWA8	5.728294615	0.729999722	0.546139596	1.336654084	0.181335576	0.260001096
POLR2E	95.45266332	-0.2464333	0.184395838	-1.336436348	0.181406692	0.260047388
ZDHHC1	6.120324946	-0.723944261	0.541771209	-1.336254583	0.181466076	0.260104619
MAGI1	51.11352569	0.296054266	0.221574168	1.336140713	0.181503285	0.260130057
CCSER1	54.58695256	0.318791717	0.238629187	1.335929279	0.18157239	0.260201197
TRIM63	1.489140245	-1.447354459	1.08413856	-1.335027194	0.181867446	0.260596086
DYRK1B	23.816053	0.41472565	0.310762052	1.334544059	0.182025617	0.26071093
LOC114117862	19.23167377	0.441944019	0.331120182	1.334693696	0.181976618	0.26071093
MFSD1	21.12066955	-0.409886886	0.307131125	-1.334566421	0.182018294	0.26071093
STXBP3	6.400088388	0.662765249	0.49661724	1.334559486	0.182020566	0.26071093
AP1S2	17.78794671	0.405011276	0.303555289	1.334225725	0.182129891	0.260776446
GMFB	23.89339244	-0.47245636	0.354099294	-1.33424824	0.182122515	0.260776446
QPRT	33.62590921	-0.398789061	0.298872255	-1.334312749	0.182101381	0.260776446
ROGDI	3.168785426	-0.905999681	0.679109587	-1.334099384	0.182171288	0.26080778

PSENEN	13.72694789	-0.492524644	0.36924733	-1.333861083	0.182249388	0.260891648
BOP1	29.0151545	0.400347461	0.300174989	1.333713586	0.182297741	0.260932919
LOC121820168	3.162196383	-0.926672265	0.694901141	-1.333531074	0.182357585	0.260990629
KIF23	40.90707094	-0.301847998	0.226404768	-1.333222794	0.182458701	0.261107387
NSUN3	5.90204647	-0.664906572	0.499114799	-1.332171623	0.182803799	0.261573234
LOC121816870	1.373072053	1.593985902	1.196855989	1.331810942	0.182922322	0.261686798
MMAB	52.5834323	0.322607282	0.242222764	1.331861948	0.182905557	0.261686798
SLC44A4	54.99299551	-0.402604507	0.302317697	-1.331726561	0.182950058	0.261698467
LOC132658492	6.706009694	-0.661767499	0.497093758	-1.331273002	0.183099198	0.261883776
ACER3	9.21701822	-0.55904541	0.4200893	-1.330777551	0.183262217	0.262088892
SMPD2	31.73249336	-0.440100104	0.330796324	-1.330426221	0.18337788	0.262226248
DIAPH1	66.68283557	0.323455679	0.24318794	1.330064637	0.183496976	0.262354188
RHEB	4.456131461	0.782280924	0.588165489	1.330035403	0.183506607	0.262354188
URI1	97.24821781	0.320263348	0.240820788	1.329882486	0.183556993	0.262398157
ETNK1	170.6195049	-0.212033905	0.159479966	-1.32953317	0.18367213	0.262506597
SLC7A8	7.246583195	-0.669891669	0.503854376	-1.329534287	0.183671762	0.262506597
CWC22	109.8911547	-0.255071705	0.191880876	-1.329323231	0.183741353	0.262577454
TRIM11	7.53264208	-0.693039308	0.521577966	-1.328735784	0.183935155	0.262826307
NIPA2	12.95339878	0.499988514	0.37633455	1.328574573	0.183988366	0.262874238
FAM72A	7.213522649	-0.675246266	0.508323663	-1.328378582	0.184053071	0.26293858
IRAK3	9.391980631	0.613981256	0.462402637	1.327806562	0.184242018	0.26318038
RRAS2	27.12113214	-0.330284026	0.248764145	-1.327699477	0.184277406	0.263202801
B4GALT6	5.718221771	0.691720655	0.521275511	1.326977078	0.184516265	0.263515803
SH3GL3	1.47276981	1.462107112	1.102085063	1.326673558	0.184616692	0.263631058
ECPAS	143.4479526	-0.206008193	0.155292226	-1.326584072	0.184646308	0.263645182
GAB1	9.250039936	0.634659037	0.478740031	1.325686169	0.184943671	0.264037703
GSK3B	76.75128625	-0.234449032	0.176857946	-1.325634713	0.184960722	0.264037703
MAPKBP1	18.28611029	0.45756888	0.345198078	1.325525573	0.184996893	0.264061136
LOC114117238	2.124245899	1.154908679	0.871639266	1.324984686	0.185176231	0.264288895
SRSF6	30.81700165	-0.388381206	0.293192784	-1.324661545	0.185283433	0.264413663
PGAP4	10.5854036	-0.571255182	0.43148771	-1.323919937	0.185529636	0.264717413
PRPF18	45.28215229	0.372740253	0.281546901	1.323901104	0.185535891	0.264717413
PTGR3	25.65043648	-0.369421692	0.279085609	-1.323685924	0.185607375	0.264791138
IRS2	22.53897835	0.369260787	0.279001972	1.323506011	0.185667159	0.264848158
SMG7	31.13849971	0.498867128	0.376959448	1.323397333	0.185703278	0.264871414
MFAP3	6.864832671	0.665533233	0.50293916	1.323287758	0.185739701	0.264895097
RASSF10	36.03343945	0.31749964	0.239975628	1.323049518	0.185818911	0.264979789
HEATR1	9.198943483	-0.619736983	0.468470559	-1.322894196	0.185870566	0.264996905
LOC105609394	3.493008782	0.932000187	0.704491831	1.322939664	0.185855444	0.264996905
LOC105607169	140.3837878	0.216883925	0.163985783	1.322577609	0.185975884	0.265115672
PLEKHB2	6.618234817	-0.666600581	0.504036452	-1.322524549	0.18599354	0.265115672
RAD51C	5.017472476	-0.787197389	0.595327207	-1.322293655	0.186070385	0.265196925

LOC132657301	2.083964961	1.194477009	0.903395419	1.322208397	0.186098766	0.265209095
DACH1	19.77953796	-0.377150444	0.285502057	-1.321007799	0.186498764	0.265750797
MBD6	11.99548077	-0.57255616	0.433538279	-1.320658838	0.186615145	0.265871948
NEO1	37.99633792	0.307385391	0.232756	1.320633587	0.186623568	0.265871948
SEC11A	114.869246	-0.196992569	0.14917299	-1.320564592	0.186646586	0.2658764
CSTF1	16.5669143	-0.477509858	0.361616393	-1.320487308	0.186672371	0.265884794
NUFIP2	94.28327116	-0.24079976	0.182452402	-1.319794955	0.186903486	0.266185615
NEU3	8.8137136	0.561160707	0.425330705	1.319351506	0.187051626	0.266368211
RPS6KA6	5.079349414	0.738319938	0.560011493	1.318401401	0.187369312	0.266792183
SLC44A1	83.87058233	-0.309180769	0.234610187	-1.317848862	0.187554247	0.267027063
POLR1F	94.39820597	-0.298476966	0.226525576	-1.317630314	0.187627433	0.267102808
GPRASP3	1.758066794	1.284131975	0.97465715	1.317521731	0.187663802	0.267126131
RHBDF1	4.266044582	-0.841278763	0.638591102	-1.317398193	0.187705186	0.267156588
NPAS2	4.260592272	0.804101735	0.610590641	1.316924435	0.187863955	0.267354091
LOC114110620	53.99929005	-0.301061995	0.22862192	-1.316855334	0.187887121	0.267358592
TMEM245	12.9091187	-0.500092193	0.37992177	-1.316303071	0.18807234	0.267593666
SMNDC1	70.89875439	0.216787227	0.164724584	1.31605873	0.188154331	0.26768183
PDZD11	16.82212438	0.424240381	0.322374561	1.315985912	0.188178771	0.267688107
PLD4	15.48920502	-0.452648352	0.344078483	-1.315538095	0.188329123	0.267873476
ASB3	17.43950585	0.423396033	0.322014327	1.314836011	0.188565022	0.26815194
KIF24	2.663564128	-1.128983333	0.858648497	-1.314837604	0.188564486	0.26815194
LSM14A	43.36498436	-0.312452382	0.237717989	-1.31438257	0.188717493	0.268340214
LOC101118735	9.736173239	-0.650171031	0.494706585	-1.314255865	0.188760114	0.268372268
BLOC1S3	4.696021014	0.793519636	0.604044111	1.313678293	0.188954489	0.268620049
ATXN1L	7.766208943	-0.594309167	0.452465035	-1.313491921	0.189017242	0.268680682
DOK1	18.50881607	0.469993552	0.357866399	1.313321268	0.189074715	0.268733798
CILK1	29.11017884	0.357071411	0.272082343	1.312365248	0.189396926	0.269163139
PMAIP1	7.740962795	-0.622097417	0.474327007	-1.311536996	0.189676403	0.269531661
CDC42	4.692502079	-0.772491651	0.589515006	-1.310385049	0.190065609	0.270056014
GEN1	11.42730464	-0.530042966	0.40463667	-1.30992321	0.190221814	0.270249232
RAB21	4.696208719	-0.748810527	0.57185026	-1.309452106	0.190381251	0.270427134
YJU2	2.295211959	-1.130558572	0.863395083	-1.309433646	0.1903875	0.270427134
DCUN1D4	7.641117888	-0.600361087	0.458636335	-1.309013353	0.190529828	0.27060054
DLG3	51.25068374	-0.412110335	0.314923788	-1.308603385	0.190668734	0.27076905
SNX18	14.68967903	-0.455462501	0.348175796	-1.308139469	0.190826008	0.270963607
SLC25A53	5.657919613	-0.719064948	0.549845504	-1.307758165	0.190955348	0.27111846
SMG9	9.020236696	0.543746696	0.415835859	1.307599344	0.191009239	0.27116617
PJA1	9.536220482	0.522561122	0.399688738	1.307420182	0.191070047	0.271223688
INO80	46.47146288	0.300260811	0.2296817	1.307290961	0.191113913	0.271257148
MCCC1	4.724795126	-0.796172769	0.609180829	-1.306956377	0.191227527	0.271389587
RPS6KB2	15.6765388	-0.479828736	0.367278156	-1.30644507	0.191401247	0.271607291
FZD6	3.454396286	0.880076104	0.674180716	1.305400887	0.191756377	0.272082349

MECP2	29.76180461	0.33093663	0.2537045	1.30441766	0.192091217	0.272528522
AARS2	11.04474897	-0.550488816	0.422152154	-1.304005704	0.192231638	0.272698797
LOC105606707	2.8053612	0.973171374	0.746354251	1.303900088	0.19226765	0.27272094
LOC114109595	2.110847092	1.283685765	0.985304651	1.302831326	0.192632353	0.273209257
NICN1	18.25832418	-0.412137716	0.316446672	-1.302392322	0.192782306	0.273392923
ADPRS	72.71038439	-0.243299295	0.186916698	-1.301645588	0.193037568	0.273696842
NOX4	1.613357694	1.373111409	1.054858265	1.30170228	0.19301818	0.273696842
LOC114116173	8.152199149	0.606687487	0.4662751	1.301136363	0.193211783	0.273914796
FMNL2	6.573875121	0.642977171	0.49432586	1.300715222	0.19335595	0.274090108
SPRTN	8.546410192	0.566096889	0.435491768	1.299902618	0.193634348	0.274455641
GEMIN4	7.214577852	-0.632544301	0.486695653	-1.299671154	0.193713701	0.274539002
ZNF654	9.188522024	0.577699833	0.444723751	1.299008274	0.19394109	0.274832125
DAGLB	2.074192547	1.192465667	0.918271655	1.298597926	0.19408195	0.275002581
DCP2	28.07698462	0.343010757	0.26422745	1.298164733	0.194230734	0.275159406
MARS2	15.14559911	0.459020259	0.35359412	1.298155803	0.194233802	0.275159406
LOC106991883	7.263221734	0.710768023	0.5478096	1.297472742	0.194468578	0.275462803
SNRPN	96.50316219	0.229427186	0.176854888	1.297262338	0.194540938	0.275506906
STAG1	54.48561893	-0.324437335	0.250090787	-1.297278237	0.19453547	0.275506906
C20H6orf89	77.36322236	0.306042988	0.236020752	1.296678304	0.194741897	0.275708101
CDKN3	6.865771657	-0.649455172	0.500864033	-1.296669614	0.194744889	0.275708101
NEK2	1.901527221	-1.200674526	0.925930319	-1.296722336	0.194726741	0.275708101
PNN	480.4362999	0.213556712	0.16483877	1.295549045	0.195130894	0.276225326
HYKK	1.745660146	1.32878592	1.026096394	1.294991316	0.195323225	0.276439033
UBE2Q2	92.7962935	0.250396352	0.193349219	1.295047137	0.195303969	0.276439033
FBXW7	118.8002155	0.222419265	0.171835297	1.294374722	0.195536018	0.276710896
DIDO1	7.64000414	0.591556664	0.457051006	1.294290257	0.195565181	0.276722867
ATP8A1	20.52194471	0.383519528	0.296517531	1.293412657	0.195868375	0.277122546
UBAC1	24.12662615	-0.376821718	0.291372207	-1.293265827	0.195919136	0.277165026
KPNA6	14.56943844	0.434274376	0.335922742	1.292780515	0.196086981	0.277373118
DIPK2A	11.32609618	-0.553778029	0.428547944	-1.292219545	0.196281125	0.277618362
DUSP19	8.075738452	0.57790453	0.447290987	1.292010227	0.196353603	0.277691488
LYSET	9.094079234	-0.539783353	0.417954325	-1.291488858	0.196534216	0.277917513
HLTF	59.3345659	0.273827561	0.212065937	1.291237834	0.19662122	0.278011131
ANKRD33B	19.81080562	-0.420802474	0.326156481	-1.290185843	0.19698614	0.278497646
LOC105610032	3.834736538	-0.871452893	0.675522957	-1.290041862	0.197036124	0.27853885
PIP4P1	55.92772516	0.269726247	0.209319362	1.288587184	0.197541641	0.279209834
SIAE	16.14497354	0.462761156	0.359138812	1.288530063	0.197561511	0.279209834
UBQLN4	12.91576482	-0.455351783	0.353397966	-1.288495769	0.197573441	0.279209834
FBXO10	2.524052126	-1.02601678	0.796450102	-1.288237363	0.19766335	0.279307361
SNRNP35	13.42732881	0.482283741	0.374414024	1.288102768	0.197710192	0.279344019
DUS1L	11.79886751	-0.476513792	0.370634733	-1.285669554	0.198558411	0.280512813
PRMT6	7.070488042	-0.643473849	0.500573043	-1.285474432	0.198626546	0.280563332

ULK2	11.06435141	-0.580269174	0.451414339	-1.285446925	0.198636153	0.280563332
CHERP	26.31291113	-0.339690886	0.264275284	-1.28536759	0.198663861	0.28057282
NEIL3	4.350366135	-0.819299466	0.637798371	-1.284574411	0.198941045	0.280934602
YPEL5	6.412147384	-0.652706622	0.508677497	-1.283144283	0.199441532	0.281611612
LOC101107164	10.25290908	-0.541353662	0.422000456	-1.282827195	0.199552624	0.281738711
MIB2	2.499673403	1.036049761	0.808237108	1.28186364	0.199890485	0.282185913
LOC132657631	15.38337064	0.531807663	0.414896861	1.281782807	0.199918847	0.282196146
MED6	12.28409865	0.5047311	0.393879277	1.281436039	0.200040552	0.282338123
CTIF	8.873607901	0.594332659	0.463958689	1.281003404	0.200192471	0.282522707
FIZ1	3.441931102	0.859485056	0.671188769	1.280541474	0.200354768	0.282692055
ZNF280C	6.467990794	0.625734665	0.488641425	1.280560003	0.200348256	0.282692055
FBXO31	4.574122258	0.781058107	0.610048577	1.280321168	0.200432206	0.282771465
COL9A1	2.050960664	-1.265070832	0.988220358	-1.280150547	0.200492194	0.282810547
SIGIRR	15.22589677	-0.429623966	0.33561173	-1.280122021	0.200502225	0.282810547
LOC114112247	8.536712659	-0.556180531	0.434642225	-1.279628391	0.20067586	0.283025593
SLC35F6	8.221636064	-0.577741371	0.451825319	-1.278683038	0.201008695	0.283464647
SMS	18.7432327	-0.431664367	0.337600773	-1.278623751	0.201029582	0.283464647
LOC132657236	4.357012604	-0.742722572	0.581509301	-1.277232488	0.20152018	0.284099761
PPM1M	2.134994455	1.211594272	0.948613927	1.277225895	0.201522507	0.284099761
CDC25C	7.462028996	0.699901042	0.548176047	1.276781511	0.201679395	0.28429095
TMPPE	11.19511713	0.524129948	0.410832658	1.27577479	0.202035142	0.284762386
MTCL2	5.540933493	0.728600731	0.571487101	1.274920694	0.202337314	0.285158218
REV1	18.14072328	-0.391573859	0.307282776	-1.274311122	0.202553177	0.285432341
PUS1	6.650084341	-0.663297976	0.52058973	-1.274128046	0.20261804	0.285493646
LOC132657401	11.67860421	0.528210926	0.414748165	1.273570255	0.202815759	0.285711998
NUDCD1	38.52436787	-0.328436023	0.25787988	-1.273600808	0.202804926	0.285711998
CLCN6	1.023990678	-1.640661715	1.288365807	-1.273444007	0.20286053	0.285744951
SNRNP200	48.37077446	-0.294298777	0.231119906	-1.273359711	0.202890427	0.285756949
SMIM13	2.659900365	-0.984500704	0.773202527	-1.273276625	0.202919898	0.285768345
RANBP1	99.13057725	0.353729639	0.27795024	1.272636565	0.203147037	0.28605808
ATP5F1D	16.95533004	-0.396093728	0.311360226	-1.272139775	0.203323461	0.286276349
EXOSC5	35.21461122	-0.416239958	0.327243324	-1.271958594	0.203387831	0.286336819
ZNF461	2.601013199	0.99697874	0.784005071	1.27164833	0.203498097	0.286461881
ASIP	1.256230124	-1.530821107	1.204076439	-1.271365386	0.203598691	0.286573305
LOC132657507	2.201857106	-1.124020864	0.884400597	-1.270940871	0.203749685	0.286755638
FAM117A	1.272832369	-1.602361074	1.261230479	-1.27047443	0.203915686	0.28692884
LOC101122645	2.7890731	-1.002023874	0.788686968	-1.2704963	0.2039079	0.28692884
TIMM29	28.87197884	-0.331478423	0.261171409	-1.26919874	0.20437019	0.287538101
ABCF3	18.27040607	0.412971455	0.325400384	1.269117909	0.204399014	0.287548386
SH3RF3	1.338831709	1.46533831	1.15515513	1.2685208	0.204612028	0.28781776
NUDT2	20.28806592	-0.410231205	0.323458271	-1.26826624	0.204702889	0.28791527
MARCHF6	57.76208768	-0.332812507	0.262469086	-1.268006503	0.204795628	0.288015401

DTD1	10.41661622	-0.528968883	0.417274773	-1.267675203	0.204913964	0.288151503
LOC101117005	8.700309559	-0.557598982	0.439918222	-1.267505991	0.204974423	0.2882062
NCAPH	5.366949694	0.744362156	0.587521329	1.266953419	0.205171946	0.288453585
PTP4A2	705.3884995	-0.142782918	0.112725991	-1.266637065	0.205285093	0.288582305
EFCAB11	38.64991135	0.287197421	0.226783174	1.26639651	0.20537116	0.288658517
PIGQ	23.4454672	0.409361829	0.323257424	1.26636482	0.2053825	0.288658517
TUFM	103.523638	0.25694689	0.202912392	1.266294714	0.205407589	0.288663425
ELMOD3	15.60296804	0.454656079	0.359074183	1.266189829	0.205445128	0.288685827
LOC101119041	1.891137351	1.230420343	0.972035539	1.265818269	0.205578153	0.288842383
LOC101116852	3.959160891	0.781591014	0.617557982	1.265615596	0.20565074	0.288913999
BTNL2	155.258749	-0.204493557	0.161599842	-1.265431666	0.20571663	0.288918045
DCP1A	5.719599825	-0.718695541	0.567974344	-1.265366206	0.205740084	0.288918045
LOC132659757	44.39510319	0.298600365	0.235978683	1.265370082	0.205738695	0.288918045
TOR3A	19.29386907	-0.530415621	0.419177163	-1.265373373	0.205737516	0.288918045
ZNF581	2.429563209	-1.035340735	0.818432143	-1.265029415	0.205860784	0.289057173
HDAC5	3.516377123	-0.832243235	0.658017425	-1.264773855	0.205952406	0.289155448
LOC101103215	8.515261033	0.574189303	0.454009517	1.264707637	0.205976151	0.289158412
LOC101108416	19.46022835	-0.35632428	0.281788155	-1.264511206	0.206046601	0.289158602
NAA15	44.60609808	0.377032597	0.298170416	1.264486941	0.206055305	0.289158602
PIN4	48.86581928	0.255252082	0.201865519	1.264465985	0.206062822	0.289158602
ZNF605	76.13921065	0.237657356	0.187924335	1.264643857	0.205999024	0.289158602
HOMEZ	13.62335823	0.473273075	0.374375923	1.264165364	0.20617068	0.289279583
MLF1	3.037310976	0.92695332	0.733346757	1.264004117	0.206228549	0.289330407
CLTRN	15.76252977	-0.419720244	0.332082785	-1.263902448	0.206265043	0.289335802
PCM1	214.3811359	0.218360917	0.172771797	1.263868989	0.206277054	0.289335802
PLAGL1	13.94880036	-0.427893962	0.33857393	-1.263812493	0.206297336	0.289335802
FUCA2	30.70853032	0.334652482	0.264914868	1.263245376	0.20650101	0.289561288
LOC132660083	18.7982002	0.385342473	0.305047477	1.263221309	0.206509657	0.289561288
TPMT	31.84455882	0.390995829	0.309531994	1.263183893	0.2065231	0.289561288
SUFU	4.871299726	-0.745315278	0.590289765	-1.262626125	0.206723575	0.289811968
AGTPBP1	9.814095638	0.551768576	0.437260365	1.261876493	0.206993232	0.290159574
GART	61.24578062	0.249654993	0.197904474	1.261492412	0.207131492	0.290298756
UBAP2	95.82328619	0.307378851	0.243665258	1.261480005	0.207135959	0.290298756
DHX58	6.93190355	-0.588844566	0.466826689	-1.261377253	0.20717296	0.29032017
SETBP1	10.88861998	0.491038058	0.389337274	1.261215125	0.207231351	0.290371552
FHIP2A	19.94767067	0.435443312	0.345319087	1.260988254	0.207313079	0.29045562
TMEM181	17.17567399	0.460718735	0.365386359	1.260908414	0.207341846	0.290465477
MRPL50	17.32340714	0.422738605	0.335379012	1.260480202	0.207496186	0.290651228
LOC132658477	1.250900349	1.492804216	1.184490901	1.260291839	0.207564103	0.290715897
PIGX	9.329031616	0.53866401	0.427575515	1.259810235	0.207737827	0.290928729
STX10	2.003165112	-1.137781541	0.903238302	-1.25966928	0.207788692	0.290969477
UBE2T	9.342265791	-0.520696427	0.413441753	-1.259419067	0.207879006	0.291065451

ASCC1	9.440325771	-0.50272752	0.399202297	-1.259330228	0.207911079	0.291079867
ATP7B	4.287484714	-0.915925726	0.727388404	-1.25919759	0.207958972	0.291116424
TSEN34	14.64955324	0.48388942	0.384379249	1.258885388	0.208071732	0.291243771
GAREM1	6.905012716	0.632033023	0.502203909	1.258518725	0.20820422	0.291398701
VDAC1	66.06226719	-0.255809213	0.203324655	-1.258131793	0.208344097	0.291563941
CABYR	8.543933972	0.57295244	0.455449716	1.257992748	0.208394379	0.291603776
SRFBP1	45.87399384	-0.302335303	0.240354892	-1.257870395	0.208438632	0.291635168
PCGF5	146.0125756	-0.192723284	0.153236919	-1.257681801	0.208506857	0.291672089
STX5	2.804425226	1.083817441	0.861761494	1.257676803	0.208508665	0.291672089
LARP4	109.0464927	0.228137306	0.181427851	1.257454709	0.208589029	0.291704605
LOC114118079	4.820617688	0.701806233	0.558099321	1.257493436	0.208575015	0.291704605
LOC121818591	6.417777403	-0.704026351	0.559892352	-1.257431627	0.208597383	0.291704605
APEX1	407.6675956	-0.286419766	0.227824425	-1.257195168	0.208682973	0.291793766
FANCE	30.21259613	-0.367423346	0.292284834	-1.257072906	0.208727237	0.291825131
LOC443344	2.409827888	-1.252711598	0.996625728	-1.256952898	0.208770692	0.291855357
CCDC121	3.680013188	0.828614364	0.659749495	1.25595301	0.209133007	0.292331288
TMCC1	16.27421877	0.432033857	0.34407582	1.255635623	0.209248109	0.292461595
YWHAH	41.12947222	-0.271816196	0.216531283	-1.255320675	0.209362373	0.292590702
URGCP	14.67433534	0.431710008	0.343941623	1.255183959	0.209411987	0.292629443
TTLL3	2.874364979	0.987923416	0.7872801	1.25485633	0.20953092	0.292765029
MYBL1	5.766315405	0.644189015	0.513490015	1.254530754	0.209649155	0.292869001
TASP1	45.49034636	0.273788888	0.218232366	1.254575082	0.209633054	0.292869001
LOC101109820	23.94262797	-0.427605202	0.340877435	-1.254425077	0.209687542	0.292892015
SLC41A2	21.006801	0.4002175	0.31913159	1.254082995	0.20981184	0.293035012
ZNF830	39.57553744	-0.272329864	0.217246596	-1.25355181	0.210004956	0.293274083
CCDC191	26.68788004	0.377722747	0.301397285	1.253238717	0.210118844	0.293402472
LOC101121190	2.933451373	-0.941555816	0.751542198	-1.252831602	0.210266998	0.293578679
UBE2D1	15.30621213	0.447824913	0.357578767	1.252381164	0.210431006	0.293776983
STOX2	215.1771969	0.202370253	0.16161602	1.252167034	0.210509005	0.293855182
MITD1	13.82308852	-0.450930847	0.360162631	-1.252020085	0.210562546	0.293899226
LOC132657919	1.917215976	-1.226577315	0.979938973	-1.251687451	0.210683775	0.294037729
TOM1L2	3.685554954	-0.84998988	0.679548693	-1.250815268	0.211001883	0.294450947
SART1	6.578624451	-0.617644548	0.494110541	-1.250012897	0.211294836	0.294828978
COMMD8	32.74736668	0.314203605	0.251412414	1.249753741	0.211389519	0.294930304
ELL	8.174111538	0.563014239	0.450538319	1.249647845	0.211428217	0.294953507
AZI2	20.20311879	-0.386235062	0.309268417	-1.248866811	0.211713792	0.295321076
UBXN8	8.506157364	0.563073008	0.451149751	1.248084492	0.212000117	0.295689613
PEDS1	8.842265948	0.529817162	0.424552458	1.247942751	0.212052023	0.29573115
TMEM104	2.117400705	-1.078596077	0.864345506	-1.247876075	0.212076444	0.29573435
MYEF2	61.28750994	-0.248696171	0.199377315	-1.247364427	0.212263904	0.295910417
PHF19	5.741726441	-0.682783819	0.547387433	-1.247350191	0.212269121	0.295910417
PHF21A	18.42713761	-0.370772632	0.297242373	-1.247374753	0.212260119	0.295910417

GPBP1	58.42961864	0.247653537	0.198583165	1.247102376	0.212359962	0.296006179
LOC114108725	7.277418283	0.616021183	0.493990052	1.247031556	0.212385927	0.296011502
LOC114118738	31.39039297	0.321722253	0.258159759	1.246213795	0.212685916	0.296398702
PIDD1	8.180836546	0.561099154	0.45040764	1.245758518	0.212853063	0.296569795
SLC35F2	33.46082264	-0.306796443	0.246262796	-1.245809141	0.212834473	0.296569795
GGT7	2.495230294	-0.975828228	0.783361947	-1.245692661	0.212877249	0.296572578
F2R	100.877526	-0.217671456	0.174793164	-1.245308746	0.213018282	0.29673813
CRAT	13.88057649	-0.445893258	0.358123203	-1.245083406	0.213101093	0.296822552
NAV2	17.31687369	0.436978004	0.351025769	1.244860186	0.213183148	0.296905904
NFKBID	1.272144345	1.40740415	1.131380383	1.243970791	0.213510313	0.297330574
ZNF835	1.557646668	-1.358723753	1.092381163	-1.243818367	0.213566419	0.297377722
DENND4B	7.664480856	-0.569806153	0.458292262	-1.24332484	0.213748154	0.297599774
MAP4K2	7.361356176	-0.590158786	0.474968519	-1.242521898	0.214044066	0.29798073
RPIA	8.682900421	0.57497563	0.462934746	1.242023061	0.214228054	0.298205808
GTF2F1	12.38810623	-0.506069096	0.407600977	-1.241579693	0.214391678	0.298402496
MTMR12	12.14134535	-0.497816003	0.401034749	-1.241328848	0.214484292	0.298500317
LOC121817766	4.231472038	-0.769943228	0.62061415	-1.240615006	0.214748006	0.298836215
LOC101106881	4.8761193	-0.724642706	0.584213006	-1.240374143	0.21483704	0.298897874
LOC106991210	3.508787145	0.896531613	0.722761886	1.240424585	0.214818392	0.298897874
UBE2R2	18.13085474	-0.384140782	0.309835952	-1.23981991	0.215042012	0.299151908
CERS5	68.15633524	-0.231292496	0.186608841	-1.239450906	0.215178559	0.299310711
SWSAP1	6.248753941	0.632785413	0.510666779	1.239135655	0.215295265	0.299441884
WBP4	153.2991332	0.2262133	0.182699404	1.238172078	0.215652262	0.299907204
IL17RD	4.406220194	-0.761483695	0.615297029	-1.237587149	0.215869182	0.300177641
FAXC	23.45935381	0.364769468	0.294857992	1.237102192	0.216049146	0.300367069
LOC132659192	2.159922691	1.144299178	0.924985979	1.237098945	0.216050352	0.300367069
PPP1R36	2.859719434	-0.893933772	0.722691232	-1.236951179	0.216105208	0.300386345
TSPAN14	72.1460884	0.221051827	0.178708534	1.236940522	0.216109165	0.300386345
HELQ	9.5284405	0.537610874	0.434762739	1.236561522	0.216249913	0.300519477
RPS11	459.6994288	-0.230225396	0.186176776	-1.23659567	0.216237228	0.300519477
IPPK	10.44752394	0.488704314	0.395278739	1.236353656	0.216327135	0.300595538
MED24	69.67616101	-0.306792559	0.248308749	-1.235528592	0.216633842	0.300959145
MYLIP	6.474727894	0.748784219	0.606028107	1.235560216	0.21662208	0.300959145
LOC101112974	5.238821469	-0.679039683	0.549709819	-1.235269336	0.216730282	0.301061832
ELAC1	33.25312679	-0.29059756	0.235273581	-1.235147433	0.216775639	0.301093546
CCDC40	2.606458607	-1.042519691	0.844778753	-1.234074233	0.217175244	0.301617239
PLS3	8.649756084	-0.555638544	0.450353292	-1.233783684	0.217283521	0.301736264
LOC132659500	7.493661207	0.557945029	0.452250377	1.233708269	0.217311632	0.301743951
CRIP2	5.643361949	-0.643107111	0.521588557	-1.232977798	0.217584048	0.302090826
PRR5L	2.080268582	1.134867804	0.921210701	1.231930766	0.217974947	0.302602113
ATP6V1D	16.4165075	-0.452875956	0.367668281	-1.231751496	0.218041927	0.302663662
DOCK6	8.412006085	0.57199586	0.464402626	1.231680933	0.218068295	0.30266883

OSBPL5	2.663408477	-0.968216325	0.786154781	-1.231584858	0.2181042	0.302687233
NSMCE4A	45.6860139	0.305716439	0.248290746	1.231284065	0.21821664	0.302785901
SMAD3	79.52073702	-0.238495855	0.193698526	-1.231273463	0.218220603	0.302785901
HAUS1	13.11595121	-0.42618528	0.346193265	-1.231061731	0.218299777	0.302864316
NUP153	40.48461135	0.284780071	0.231449809	1.230418256	0.21854052	0.303166849
GPX7	1.421728497	1.351905189	1.099634996	1.229412663	0.218917125	0.303657772
MAML2	295.1513032	-0.188335493	0.15333915	-1.228228359	0.219361256	0.304242248
LOC114114011	5.072576238	0.708400993	0.576954164	1.227828894	0.219511207	0.304418633
UGCG	50.41554508	0.337170499	0.274637338	1.227693587	0.219562015	0.304457505
PDZD8	137.1639571	-0.206215111	0.168044591	-1.227145186	0.219768027	0.304711561
CHM	28.66397532	0.325922348	0.265666999	1.226807808	0.219894835	0.304855759
LOC105612738	4.060915476	-0.764994891	0.623654865	-1.226631804	0.21996101	0.304915874
SLC12A7	21.58811984	-0.37113208	0.302626946	-1.226368255	0.220060127	0.305021638
LOC132658093	1.992977941	-1.191278542	0.971533169	-1.226184118	0.220129396	0.305086013
CDK12	64.04208164	-0.232131176	0.189410604	-1.225544772	0.220370032	0.305334455
NAT10	67.28434964	0.287562477	0.234629255	1.225603673	0.220347855	0.305334455
PTPN11	350.9465765	-0.196689246	0.160493766	-1.225525767	0.220377188	0.305334455
CCNG2	19.45822894	0.403995079	0.329746743	1.225167763	0.220512018	0.305489597
LOC114109097	9.668571414	0.516705497	0.421879764	1.22476957	0.220662053	0.305665769
LOC114118101	2.169230819	1.26212409	1.031337864	1.223773638	0.221037632	0.306154299
CDK6	285.2795473	-0.290670499	0.237755786	-1.222559096	0.22149627	0.306757761
DBNDD2	19.34874436	0.440195601	0.360079164	1.222496732	0.221519839	0.306758617
BNIPL	1.602837979	-1.252335248	1.024494222	-1.222393666	0.221558793	0.306780776
NFIA	486.3380411	-0.178109352	0.145717952	-1.222288327	0.221598612	0.306804127
KATNBL1	14.14499285	0.405101242	0.331551225	1.221836056	0.22176963	0.307009102
CARNMT1	20.38754333	0.388689444	0.318178537	1.221607994	0.221855904	0.307072593
FBP1	22.48149111	0.359401281	0.29420697	1.221593358	0.221861442	0.307072593
LOC132657746	2.993869458	-0.864111004	0.707566526	-1.221243477	0.221993847	0.307196217
SSBP4	8.485169839	0.569984221	0.466727384	1.221235867	0.221996728	0.307196217
ADGRG7	54.88856995	0.269390353	0.220724851	1.220480396	0.222282817	0.307560262
ALG5	40.6874481	0.294367285	0.241287936	1.219983433	0.222471155	0.307629781
MED8	19.40658377	-0.36753691	0.301205658	-1.220219144	0.222381812	0.307629781
MGST2	63.97029204	0.38328274	0.314133657	1.220126309	0.222416997	0.307629781
NEDD9	31.58073318	-0.294109119	0.241068082	-1.220025133	0.222455348	0.307629781
RAPGEF3	3.864517783	0.826465158	0.677419523	1.220019692	0.22245741	0.307629781
VPS8	17.3480762	-0.376355585	0.308485958	-1.220008807	0.222461536	0.307629781
LOC121815981	27.32157088	0.393355058	0.322646028	1.219153572	0.22278591	0.308027349
RPGRIP1L	14.31045593	-0.443579028	0.363856622	-1.219103903	0.222804759	0.308027349
SCRN1	1.583812162	1.234420893	1.013273602	1.218250323	0.223128862	0.308443517
FADS1	16.64412808	-0.420683519	0.34539238	-1.217987262	0.223228814	0.308549775
LOC121819857	2.75803126	0.929099604	0.764375946	1.21550084	0.224175128	0.309825744
PPP1R1B	33.38170583	0.301880842	0.248459749	1.215009045	0.224362641	0.31005284

INAFM1	10.27709669	-0.537374459	0.442306291	-1.214937407	0.224389965	0.310058542
EXOSC4	9.650429657	-0.507778703	0.418067113	-1.214586575	0.224523812	0.310211419
FCHSD1	1.911584896	1.16948468	0.963132827	1.21425067	0.224652017	0.310356471
TMEM250	6.706835672	0.619035635	0.510395789	1.212854117	0.225185601	0.311061465
LOC105605990	41.99117709	0.273955954	0.225917506	1.212637122	0.225268589	0.311143946
MAP7D1	19.87222056	0.372462419	0.307287066	1.212099237	0.225474396	0.311363857
SMG6	20.57478546	-0.350000848	0.288744154	-1.212148688	0.225455469	0.311363857
LOC121819274	6.060247769	0.618390087	0.510443722	1.211475545	0.225713202	0.311661431
ONECUT3	5.942488421	0.631391216	0.521354344	1.211059663	0.225872539	0.311817017
RIC8A	9.685384755	-0.543133447	0.448474053	-1.211069944	0.225868599	0.311817017
NEDD4	6.797532386	-0.605062018	0.499966004	-1.21020632	0.226199734	0.31223646
SMARCC1	346.4136239	-0.203877142	0.168543149	-1.209643607	0.226415677	0.312502266
SLC41A1	4.662658045	0.713290536	0.589888834	1.209194844	0.226587998	0.312707815
SUN1	72.33356574	-0.375728191	0.310985545	-1.208185389	0.226975961	0.313210891
EPB41L5	23.06094219	0.353433804	0.292747599	1.207298727	0.227317121	0.313649286
LOC114113841	2.597724812	-1.00739222	0.834982115	-1.206483591	0.227631082	0.314050067
TSC22D2	25.47659629	0.36281011	0.300774483	1.206252961	0.227719969	0.314140273
TRMT10B	46.21508355	-0.295336495	0.244853319	-1.206177216	0.227749167	0.314148129
MAF1	17.18118558	0.431331035	0.357795867	1.205522687	0.228001585	0.314463852
C22H10orf95	4.087948225	0.773196666	0.641761842	1.204803114	0.228279318	0.31481442
GTF3C4	32.03284595	0.325940297	0.270649063	1.204291245	0.228477029	0.315054573
OLR1	1.463267591	1.426878251	1.184956755	1.204160611	0.228527507	0.315091671
LOC121817549	2.509725503	-0.976713302	0.811502997	-1.203585577	0.228749797	0.31536563
GJA10	10.6555414	0.50532272	0.420107893	1.202840338	0.229038112	0.315697989
QRSL1	17.80209008	0.405110159	0.336778127	1.202899256	0.229015308	0.315697989
G2E3	17.46060733	-0.38011037	0.316473981	-1.201079372	0.229720414	0.3166058
PI4K2B	11.55287321	0.502408387	0.418447779	1.200647757	0.229887867	0.316803922
SH3BGRL3	9.86860292	0.516584302	0.430720854	1.199348251	0.230392561	0.317466697
TXNL1	23.83597508	-0.327784467	0.273390958	-1.198958695	0.230544007	0.317642635
LOC101113241	2.032948248	-1.132667637	0.945003053	-1.198586221	0.230688879	0.317809478
SUPT16H	222.7542617	0.181498337	0.151439699	1.198485856	0.230727926	0.317830512
LOC105603225	9.042961284	-0.53222066	0.444285927	-1.197923741	0.230946706	0.318099101
LOC101122322	18.31661759	-0.363272534	0.303383504	-1.197403712	0.231149237	0.318345256
LIN52	23.08567718	0.385237658	0.321745672	1.197335943	0.23117564	0.318348816
KIFBP	79.74508098	-0.23960627	0.200141763	-1.19718277	0.231235324	0.318398201
CEP85	5.608901418	-0.665262697	0.55573521	-1.197085743	0.231273136	0.318417464
DECR2	8.01812708	-0.532072212	0.44456354	-1.196841765	0.231368236	0.318482785
DENND6A	3.393211189	0.800943173	0.669197917	1.196870392	0.231357076	0.318482785
LOC114110970	5.61342478	-0.645135101	0.539382557	-1.196062224	0.231672278	0.318868465
ESPL1	2.9489455	-0.856958927	0.716702453	-1.195696935	0.231814848	0.319003682
LOC114116932	2.629293281	-0.934052023	0.7811836	-1.195688214	0.231818252	0.319003682
SRPX2	1.468982048	1.283011009	1.073162706	1.195541927	0.231875365	0.319049427

GABPB1	12.81628271	0.453078988	0.379002295	1.19545183	0.231910546	0.319064988
PTCD3	30.47996384	0.288443901	0.241374123	1.195007556	0.232084078	0.319270871
CASP8AP2	61.09919977	0.234510656	0.196296978	1.194672776	0.232214903	0.319417968
ARRDC3	9.925163726	0.561808778	0.470363503	1.194414054	0.232316042	0.319491328
RECQL4	13.72346042	-0.485396152	0.406374911	-1.194454033	0.232300411	0.319491328
ALDH6A1	16.77322665	0.385509053	0.322790623	1.194300658	0.23236038	0.319519429
LOC132658197	5.862153899	-0.64226918	0.537917539	-1.193991891	0.23248114	0.319652599
SMC5	163.8142574	-0.190993383	0.159984628	-1.193823338	0.23254708	0.319710376
NUDT4	87.85060553	0.217750009	0.182479447	1.193285121	0.232757728	0.319967067
FCSK	6.823766531	0.65227163	0.546833772	1.192815191	0.23294176	0.32018712
GALNT3	30.90661047	0.334949285	0.280858875	1.192589288	0.233030263	0.320275835
C3H12orf4	13.02255262	0.431478278	0.361831439	1.192484212	0.233071438	0.32029949
RAB40C	6.410275709	-0.697973309	0.585605322	-1.191883482	0.233306936	0.320590162
SUPT20H	22.71750306	0.327152475	0.274617446	1.191302594	0.233534816	0.320870307
SCAMP5	2.723061786	-0.935888314	0.785932963	-1.190799162	0.233732438	0.321108826
USP5	16.87993083	-0.464525771	0.390162588	-1.190595373	0.233812469	0.321185762
INTS6	67.87533882	0.227800589	0.191438303	1.189942584	0.23406896	0.321505058
CARMIL1	49.14321344	-0.259007838	0.217700234	-1.18974534	0.234146499	0.321578515
MIOS	7.051694451	0.618312689	0.519814151	1.189487989	0.234247695	0.321651396
SAP18	43.36154038	-0.283390944	0.238237327	-1.189532082	0.234230354	0.321651396
PEX3	7.156475435	0.570560302	0.479733652	1.189327242	0.234310919	0.321705162
HDAC3	141.99477	0.188122949	0.1582176	1.18901405	0.234434138	0.321841279
EPHA7	2.432249267	0.943837887	0.794051953	1.188634928	0.234583357	0.322013058
MACO1	81.27550841	0.206915563	0.174168848	1.188017061	0.234826688	0.322313978
TMEM234	11.73562544	-0.466902402	0.393035971	-1.187938094	0.2348578	0.322323581
VRK2	10.59181655	0.453413571	0.382004371	1.186932939	0.235254074	0.322834286
LOC101109919	2.270601056	1.168610807	0.98491593	1.18650818	0.235421673	0.322975895
LOC101117965	135.1952142	0.192209738	0.161998202	1.186493028	0.235427653	0.322975895
LZTS3	55.77564625	-0.300544779	0.253306276	-1.186487696	0.235429758	0.322975895
LOC101116597	1.770610994	1.104125308	0.930879477	1.186109841	0.235578925	0.323147363
LSM8	43.85215447	-0.283076842	0.238845271	-1.18518923	0.235942637	0.323613062
RBM4	27.33336211	0.375580553	0.316986557	1.184846944	0.236077968	0.323765455
KLHL15	8.227132762	-0.509119109	0.430346892	-1.183043539	0.236791893	0.324711238
RBM43	13.88656503	-0.426912124	0.361101916	-1.182248292	0.237107196	0.325110257
ARMT1	3.524096966	-0.766664359	0.648825182	-1.181619304	0.23735679	0.325419106
ZXDC	20.50717079	-0.391694122	0.331662247	-1.181003039	0.237601516	0.325721217
TWSG1	10.3527918	0.493075205	0.417651155	1.180591024	0.237765231	0.325912222
PRIM2	2.063736319	-1.142353376	0.968056235	-1.18004857	0.237980898	0.326174393
LOC132658823	8.750143019	-0.561862404	0.476229912	-1.179813343	0.238074462	0.326269174
LOC132658575	2.425009426	-1.002688683	0.8500696	-1.179537161	0.238184348	0.326386303
PIM3	55.86312837	-0.272270415	0.230888786	-1.179227539	0.238307583	0.326521697
ZNF316	3.010940637	-0.861150114	0.73036252	-1.179072161	0.238369443	0.326572978

DUSP11	6.495775912	-0.596774507	0.506610124	-1.177975882	0.238806221	0.327137845
IMMT	119.0877317	0.255169627	0.216635628	1.177874704	0.238846561	0.327159575
BARD1	4.651660542	-0.661904152	0.562100401	-1.177555025	0.238974049	0.327300659
IQCB1	13.07535568	-0.407808384	0.346464043	-1.177058317	0.239172231	0.327538528
ATP6V1C1	44.94213414	0.278870261	0.237168953	1.175829543	0.239662998	0.328176994
REC8	2.451674454	-0.971654585	0.82643266	-1.175721425	0.239706214	0.328202547
WDPCP	19.30852214	0.358312344	0.30486872	1.175300451	0.239874534	0.328399368
TMT1A	14.49249239	0.440181454	0.374613296	1.175028914	0.239983148	0.328514416
IRAK1	37.92121877	-0.298123953	0.253747062	-1.174886325	0.240040197	0.328558861
DAZAP2	176.8673626	0.20898972	0.177974574	1.17426729	0.240287981	0.328864341
CABCOCO1	15.1519241	0.39097454	0.332978521	1.174173452	0.240325557	0.328882093
LOC101104224	18.78553984	0.458482582	0.390560997	1.173907752	0.240431977	0.328994042
JUNB	100.5966311	-0.251079018	0.213922091	-1.173693737	0.24051772	0.329077679
ZNF79	2.917788104	-0.864392023	0.736545382	-1.173576054	0.240564877	0.329108511
HNRNPH2	66.14087943	-0.263384947	0.224549327	-1.172949171	0.24081619	0.329418605
DDX21	340.313678	-0.21885326	0.18665662	-1.172491283	0.24099987	0.32963613
HSD17B12	133.7229803	-0.222645739	0.189954667	-1.172099338	0.241157176	0.32978584
ZNF304	5.699216953	-0.615218676	0.52488779	-1.172095613	0.241158671	0.32978584
LRP1	3.743433577	0.784244494	0.6691486	1.17200349	0.241195655	0.329802673
GNAI2	15.38366496	0.394519011	0.336644607	1.171915434	0.24123101	0.329817275
UBE2I	102.2316665	-0.241398162	0.206019216	-1.171726437	0.241306906	0.329887297
NMNAT3	2.226813343	1.051718005	0.897739295	1.171518292	0.24139051	0.329967841
EDRF1	3.924909309	0.743563371	0.634936034	1.171083906	0.241565052	0.330172665
LOC121817557	14.95462933	-0.417330306	0.356444476	-1.170814346	0.24167341	0.330286994
LRRC66	7.528320832	0.550340497	0.470425499	1.169878118	0.242050021	0.330767875
HDHD2	6.887675221	0.558228066	0.477271818	1.169622939	0.242152742	0.330806781
PDRG1	55.14603242	0.274465951	0.234650843	1.169678098	0.242130536	0.330806781
TBCCD1	4.406110743	0.858520669	0.733979748	1.169678961	0.242130188	0.330806781
NUFIP1	30.51987617	-0.316817349	0.270911341	-1.169450298	0.242222255	0.330867926
CABLES1	4.479511795	-0.91125876	0.779363901	-1.169233985	0.242309372	0.330953104
UBL5	28.77880969	0.315941955	0.270250242	1.169071868	0.242374677	0.331008474
LPAR2	13.55209523	0.483851859	0.414021254	1.168664301	0.24253891	0.331198925
ZNF568	9.573623713	0.524711704	0.449066297	1.168450421	0.242625127	0.331282812
LOC121818609	2.016825279	-1.0158485	0.871266172	-1.165945072	0.243636654	0.332629982
LOC132659242	1.265969405	1.411475071	1.210764139	1.165772115	0.243706594	0.332691486
TGFB1	4.926111317	0.673335517	0.577682672	1.165580256	0.243784195	0.332763434
HADH	17.62985337	0.514579394	0.441798429	1.164737944	0.244125085	0.333194719
IQSEC2	13.6246466	0.444645016	0.381904297	1.164283878	0.244308988	0.333411674
MXD4	10.45515695	-0.524097124	0.450470806	-1.16344304	0.244649796	0.333842692
EFNA1	20.32593857	0.357699147	0.307509414	1.163213648	0.24474283	0.333935552
ERI1	12.77818703	-0.510555273	0.439023295	-1.162934357	0.244856137	0.334022508
UNC13D	10.104499	0.483386615	0.415661492	1.162933359	0.244856541	0.334022508

FAM174B	48.86055396	0.384387958	0.330583863	1.162754753	0.24492902	0.334087282
BAIAP2L1	85.03122291	-0.305659029	0.2633232	-1.160775158	0.245733348	0.335116001
OTULIN	13.00525725	0.412437574	0.355310288	1.1607814	0.245730809	0.335116001
ABHD8	2.596404939	-0.931219891	0.80237938	-1.160573059	0.245815567	0.335193927
SLC38A5	2.534516944	0.939143214	0.810043347	1.159374023	0.246303761	0.335825366
MRM1	7.20293854	-0.580606026	0.500995792	-1.158903996	0.24649532	0.336052269
ARK2N	13.6993219	-0.392900521	0.339080713	-1.158722706	0.246569233	0.336118752
LZIC	45.92264332	0.23680857	0.204519355	1.157878533	0.246913609	0.336553875
NDUFAF8	38.14724987	-0.28609373	0.247246616	-1.157118893	0.247223789	0.336873602
PHF13	17.89447564	0.360223415	0.311300401	1.157156925	0.247208253	0.336873602
SLC12A9	2.113878721	1.004956853	0.868439185	1.157198881	0.247191115	0.336873602
GLTPD2	1.178557014	-1.378084944	1.191521494	-1.156575816	0.247445708	0.337141624
KCTD9	5.553030412	-0.643505252	0.557000219	-1.155305205	0.247965465	0.337815351
COPRS	17.28735932	0.35564698	0.307879103	1.155151408	0.248028429	0.337866693
POGK	7.725111194	-0.516003216	0.446809809	-1.154860983	0.248147359	0.337994253
NT5C3A	9.464550386	0.479090024	0.414944221	1.154588977	0.248258783	0.338111564
PCLO	6.867527615	-0.589954823	0.511074727	-1.15434161	0.248360144	0.338215148
MRPS28	22.11966328	0.325519	0.282048576	1.154123891	0.24844938	0.338289783
TRIM25	122.4189219	-0.231295148	0.200414414	-1.154084398	0.248465569	0.338289783
USP38	5.312918191	0.685444194	0.595091163	1.151830572	0.249390702	0.339514782
BECN1	116.2162122	-0.180731062	0.157011672	-1.151067686	0.249704391	0.339907209
CHD8	51.02183528	0.27685013	0.240534891	1.150977013	0.249741692	0.339923367
LOC105611838	26.22680027	0.303419545	0.263693597	1.150651925	0.249875462	0.340068612
LOC121819991	1.914710634	-1.143377418	0.993727924	-1.150594031	0.24989929	0.340068612
DSTYK	6.075532293	-0.598717429	0.520463384	-1.150354562	0.249997867	0.340168124
PTMA	4636.844592	-0.249769649	0.217175361	-1.150082808	0.250109767	0.340285743
GALNT18	3.488648545	0.803475172	0.698884932	1.149653019	0.250286812	0.340491962
ACVR2B	12.84078959	0.413665335	0.360121135	1.14868386	0.250686364	0.341000808
MBP	9.312323767	0.516228841	0.449440618	1.148602998	0.25071972	0.341003044
RRP7A	7.822740953	-0.523475495	0.455768296	-1.148556185	0.250739033	0.341003044
RPE	26.64761252	0.292778587	0.254962492	1.148320227	0.250836392	0.341100744
RMND5A	17.29615699	0.368802859	0.321217933	1.148139068	0.250911158	0.341167705
LOC132657329	1.647397245	1.113465532	0.971023397	1.146692794	0.25150861	0.341945283
RAB22A	66.72295867	0.25271347	0.220574515	1.145705661	0.251916961	0.342465633
ZNF570	32.34079604	0.30032346	0.262206086	1.145371813	0.252055169	0.342618671
AGMAT	2.930355498	0.860516072	0.751523025	1.14502955	0.252196917	0.342776488
LOC121818299	1.844450909	-1.104006991	0.96429216	-1.144888486	0.252255354	0.3427862
SKP1	13.91069124	-0.416374783	0.363679249	-1.144895632	0.252252394	0.3427862
ZNF445	43.97952032	-0.239050078	0.208886484	-1.144401848	0.252457022	0.343025369
NFATC2	6.268068109	0.564766278	0.493723971	1.143890738	0.252668952	0.343278432
LOC105611671	1.638620994	-1.099055421	0.960926449	-1.143745624	0.252729146	0.343325315
LOC101102969	3.39402412	-0.793087392	0.693604417	-1.143428981	0.252860526	0.34346888

MCAT	3.633676696	0.759130056	0.6641104	1.143078103	0.253006165	0.343631785
LOC132657992	2.438506194	-0.920502051	0.805645527	-1.14256459	0.253219414	0.343886476
ADGRG6	14.00293516	-0.407171045	0.356398374	-1.142460446	0.253262678	0.343910287
CAVIN3	4.197916162	-0.730884054	0.63989629	-1.142191423	0.25337446	0.344027127
ATP13A2	5.738976207	-0.58055416	0.508668132	-1.141322059	0.253735926	0.344482924
SNRPD2	71.42577278	-0.364412021	0.319323672	-1.141199518	0.253786905	0.344517141
ACVR1	3.988213424	0.806727273	0.707166548	1.140788228	0.253958061	0.344714475
MACROD1	3.709069024	-0.819943855	0.718984505	-1.140419368	0.254111628	0.344887898
EEF1AKMT1	8.917621848	-0.474646098	0.416366533	-1.139971781	0.254298058	0.345105883
HIPK2	347.842527	0.171306133	0.150283348	1.139887653	0.25433311	0.345118411
SH3BP5	13.33936836	0.411573412	0.361331544	1.139046449	0.254683781	0.345559174
PPP2R5D	74.52436126	0.26703173	0.234611574	1.138186517	0.255042607	0.346010911
BCL2L14	16.5475931	0.375262254	0.329831308	1.137739943	0.255229089	0.346193628
RPUSD3	9.248623823	-0.595538465	0.523419842	-1.13778351	0.255210892	0.346193628
CDC16	13.26538975	0.39429644	0.346643266	1.137470359	0.255341709	0.346276104
MOB3A	5.861683243	0.627816727	0.551930862	1.137491615	0.255332828	0.346276104
LOC105602066	1.585380914	-1.185929734	1.043500408	-1.136491874	0.255750765	0.34679565
AGL	12.02078726	0.441765622	0.388743207	1.136394446	0.255791519	0.346815728
CHCHD2	59.92213435	-0.277839191	0.244551733	-1.136116223	0.255907926	0.346938365
ADPGK	4.084494049	-0.745841084	0.65660184	-1.135910742	0.255993922	0.346949378
HTRA1	47.84644475	-0.283385663	0.249461509	-1.135989533	0.255960945	0.346949378
SS18L1	2.093266515	0.978336434	0.861233748	1.135970851	0.255968764	0.346949378
LOC132657142	2.74724799	-0.864673203	0.761379528	-1.135666473	0.256096177	0.347052774
PTPN14	29.14411018	-0.286629203	0.252429054	-1.135484203	0.256172497	0.347121005
NFKBIL1	6.526220576	-0.620705831	0.547040211	-1.134662166	0.256516895	0.34755244
ASH2L	15.53924636	-0.41192789	0.363201801	-1.13415707	0.256728668	0.347768864
MAP3K5	14.63186269	0.392905829	0.34641658	1.134200417	0.256710489	0.347768864
LOC101122142	62.62099872	-0.260407342	0.229718794	-1.133591801	0.256965814	0.34805483
URB1	10.19227174	-0.457624718	0.403812346	-1.133260837	0.257104733	0.348207707
UBQLN1	113.1791289	-0.190122298	0.167983794	-1.131789521	0.257722934	0.349009599
RING1	10.2005753	-0.465997966	0.411805247	-1.131597933	0.25780351	0.349083346
OVOL2	15.89967528	-0.424817548	0.375463385	-1.131448672	0.257866296	0.349132993
USP30	12.15952293	-0.440519277	0.389531272	-1.130895792	0.258098955	0.349412603
BUD23	7.771999809	-0.687021126	0.607560652	-1.130786076	0.258145142	0.349439737
LOC132658531	1.519225323	1.144025482	1.011774123	1.130712337	0.258176187	0.349446371
LOC114117925	3.165813559	-0.811492937	0.717849303	-1.130450269	0.258286542	0.34954006
RTF1	193.4537009	0.203235842	0.179787306	1.130423757	0.258297708	0.34954006
SERF1B	28.87719325	-0.308952679	0.273392535	-1.130069914	0.258446766	0.349706365
FAM193A	10.59871375	0.431865315	0.382371975	1.129437677	0.258713247	0.350031507
ARHGDIA	29.7164152	0.315795635	0.279660976	1.129208801	0.258809762	0.350126648
CUL4B	27.17289312	-0.287718219	0.254866304	-1.12889862	0.258940603	0.350268202
ELAVL4	5.185122841	0.679741773	0.602552591	1.128103643	0.259276151	0.350651121

RPS6KC1	12.57949404	-0.447029268	0.396250576	-1.128147932	0.259257449	0.350651121
MFSD5	6.421168511	-0.548295066	0.486072262	-1.128011427	0.259315093	0.350668306
MEA1	15.66817996	0.384660418	0.34133099	1.126942555	0.259766769	0.351208036
PRDX5	38.91469504	-0.297689565	0.264150077	-1.126971338	0.2597546	0.351208036
EXOC3L2	3.821198198	0.784172054	0.696044712	1.126611612	0.259906728	0.35136172
NCL	558.7780248	-0.230762802	0.204920968	-1.12610634	0.260120511	0.351615165
ITM2B	2.272499694	-0.999428813	0.88771692	-1.125841798	0.260232489	0.351704016
PRODH	3.009039713	0.813067626	0.722196059	1.125826728	0.260238868	0.351704016
KCTD10	21.0988782	0.395232021	0.351134898	1.125584566	0.260341404	0.351807017
FUZ	2.044200837	-0.990828154	0.880688459	-1.125060904	0.260563227	0.352071178
CGRRF1	16.62907082	0.403861606	0.359080284	1.124711168	0.260711447	0.352235844
SLC23A2	12.3246254	0.45614269	0.406008574	1.123480438	0.261233504	0.3529055
HTATIP2	15.43487699	0.412713735	0.367618099	1.122669792	0.261577762	0.353315681
LOC114109527	1.884083095	-1.079795745	0.961835318	-1.122640981	0.261590003	0.353315681
PNRC1	25.62948503	-0.294393311	0.262389368	-1.121971189	0.26187469	0.353664455
TIRAP	6.775161532	0.575701336	0.513177534	1.121836593	0.261931924	0.353706011
PPP2R1B	146.0759538	-0.161235843	0.143735621	-1.121752855	0.261967537	0.353718365
EIF2D	5.81570294	-0.766083697	0.683029551	-1.12159671	0.262033951	0.353772302
TTC21B	13.64478493	0.438497638	0.391024317	1.121407593	0.262114406	0.353845182
LOC132659851	1.58581687	1.108947104	0.988991742	1.12129056	0.262164202	0.353876664
ERLIN2	47.56975363	0.227092827	0.20255661	1.121132642	0.262231406	0.353931634
POGLUT2	4.578328958	0.660686446	0.58955844	1.120646235	0.262438476	0.354175351
TBC1D17	16.2298882	-0.380965287	0.34002599	-1.120400493	0.262543135	0.354280822
AMACR	11.87328989	-0.707115961	0.631267996	-1.120151766	0.262649094	0.354388027
EMC7	14.05931369	-0.40206611	0.35897447	-1.12004096	0.262696308	0.354415954
LOC121816294	2.542580887	-0.870984951	0.77826352	-1.119138863	0.263080902	0.354899005
TSR3	28.9128863	0.424575587	0.379496402	1.118786858	0.26323108	0.35506576
LOC121818078	14.45546178	-0.484942578	0.433575152	-1.1184741	0.263364562	0.355209963
GLCCI1	10.77302451	0.478881318	0.428238607	1.118258162	0.26345675	0.355298448
ZFP57	4.311501813	0.673952548	0.602757002	1.118116498	0.263517241	0.355344173
TUBE1	1.172188317	1.309326044	1.171086966	1.118043392	0.263548461	0.355350422
C2CD3	8.085597485	-0.488967049	0.438198021	-1.115858644	0.264482647	0.356574042
ABHD4	3.464305282	0.742310799	0.665375375	1.115627097	0.264581788	0.356635758
GAS2L3	4.238776761	-0.696512567	0.62430533	-1.115660132	0.264567642	0.356635758
CETN2	16.79820612	0.378872479	0.339724215	1.115235425	0.264749549	0.356825903
ZNF892	2.824934201	0.914605548	0.82072403	1.114388654	0.265112489	0.35727904
ADCY1	2.469494608	0.974664501	0.874805935	1.114149393	0.265215102	0.357381293
UBE2D4	16.68863412	0.348987427	0.313393877	1.11357449	0.265461775	0.35767763
ALG11	15.01344235	-0.387103934	0.347694537	-1.113344885	0.265560336	0.357774362
ABITRAM	3.16234054	-0.810381464	0.728129389	-1.112963543	0.265724087	0.357958895
LOC132659646	19.86170421	-0.342760153	0.308066955	-1.112615772	0.265873484	0.358124053
TMX2	20.66005282	0.32393522	0.291231454	1.112294757	0.266011437	0.358273768

FHIP2B	13.86366101	0.436917503	0.392976606	1.111815553	0.266217463	0.358492795
GPR19	8.748585956	0.522696688	0.470139028	1.111791742	0.266227703	0.358492795
SLX4	3.214719769	0.78350164	0.705734239	1.110193608	0.266915607	0.359382895
ACYP2	10.04004449	0.442171419	0.398396262	1.109878434	0.267051415	0.359529534
LOC132657231	1.693396113	-1.053695876	0.949487409	-1.109752341	0.267105761	0.359566483
CD9	5.432317144	-0.708043865	0.63820283	-1.109433917	0.267243038	0.359678828
LOC132658044	2.144504453	1.011420289	0.911630131	1.109463427	0.267230314	0.359678828
LOC105605067	16.25900492	0.488960647	0.440942311	1.108899361	0.2674736	0.359952893
MMP9	2.806485078	0.945466	0.852868802	1.108571445	0.267615102	0.360107063
LOC114110805	1.640172233	-1.094269644	0.9874869	-1.108135858	0.267803147	0.360323823
SAXO2	1.327121722	-1.224214794	1.105341484	-1.107544421	0.268058618	0.360631252
GOPC	54.96164707	0.219231966	0.197997144	1.107248122	0.268186667	0.360734954
LIPT1	8.780431135	0.484129781	0.437239691	1.107241155	0.268189678	0.360734954
HMCES	18.83113991	-0.35782172	0.32325538	-1.106931985	0.268323336	0.360878417
PLAT	6.065411166	0.627285452	0.567011456	1.106301195	0.268596175	0.361209022
CALM2	256.1639895	-0.243762689	0.220409279	-1.105954748	0.268746106	0.361320863
LOC132657416	1.783087871	-1.014193349	0.916987025	-1.106006215	0.26872383	0.361320863
PRPSAP2	16.01488343	0.360439795	0.325918024	1.105921638	0.268760439	0.361320863
DGUOK	4.814562858	-0.648594188	0.58651252	-1.105848837	0.268791953	0.361326887
IMPDH2	73.96179495	-0.194835334	0.176462797	-1.104115637	0.269542979	0.362300026
FGF11	1.695724302	-1.270306693	1.150901595	-1.103749181	0.269701955	0.362477258
NDUFAF7	10.02975442	-0.436795359	0.395764372	-1.103675291	0.269734018	0.362483901
GNL3L	4.144874145	-0.718169217	0.650844029	-1.103442892	0.269834879	0.362582989
EDN1	5.815582658	0.624532213	0.566344134	1.102743323	0.270138647	0.36295468
LOC132658526	1.68651458	1.231656371	1.117486875	1.102166297	0.270389381	0.363255047
ATM	23.6579702	0.345613667	0.313632975	1.101968524	0.270475356	0.363319325
MADD	10.51441156	-0.510556915	0.46332922	-1.101931182	0.270491591	0.363319325
LOC105602036	7.354182555	-0.526436734	0.47830032	-1.100640564	0.271053124	0.364036983
TNFAIP3	2.482913051	0.858377822	0.779994656	1.100491926	0.271117846	0.364087323
THOP1	4.615165517	-0.697549746	0.634090728	-1.100078768	0.271297804	0.36429239
HEXD	7.255889587	0.546797342	0.497150576	1.099862632	0.271391978	0.364382238
PIBF1	5.848177868	0.578213479	0.526232348	1.09877981	0.271864121	0.364942838
WIPI1	10.45338414	-0.457369057	0.416234778	-1.098824705	0.271844534	0.364942838
FXN	21.62251121	0.316644979	0.288270761	1.098429054	0.272017181	0.365038318
NEIL2	10.38875874	0.429103683	0.390628457	1.098495707	0.271988091	0.365038318
SMIM12	14.56901522	-0.41548016	0.378237456	-1.098463817	0.272002009	0.365038318
DAPK3	6.006074913	-0.602424862	0.548581736	-1.098149689	0.272139131	0.365165307
LOC101103232	54.71516091	-0.275523574	0.250935987	-1.097983504	0.272211693	0.365224661
MYO9B	13.25230822	0.500449462	0.455814623	1.097923228	0.272238014	0.365224661
KAT8	6.47284016	-0.60014763	0.546747969	-1.097667782	0.272349583	0.365337668
LOC114115364	2.164688958	-0.92815101	0.845645168	-1.097565557	0.27239424	0.365360904
DTNB	9.74446397	-0.438404113	0.399611057	-1.097077036	0.272607718	0.365610552

C25H1orf131	51.24140458	-0.227516093	0.207607118	-1.095897366	0.273123692	0.366265804
SMIM10	2.854789086	-0.810998657	0.740349888	-1.09542619	0.273329965	0.36650565
CFAP206	1.296952619	-1.243821844	1.135836474	-1.095071229	0.273485432	0.366677328
EIF4EBP2	59.35500496	-0.255555641	0.233552283	-1.094211699	0.273862142	0.367145575
AVL9	28.17752268	0.36860458	0.3369744	1.093865231	0.27401409	0.367312439
MAP3K3	11.24832378	0.416451172	0.380778614	1.093683197	0.274093946	0.36738264
BCL2	34.49074421	0.248456519	0.227423985	1.0924816	0.274621474	0.368052805
LOC101119317	29.9889155	-0.302065731	0.276526218	-1.09235838	0.274675609	0.368088449
MIPOL1	3.1324299	0.763578295	0.699495241	1.091613281	0.275003116	0.368490391
LENG1	12.33595768	-0.408907164	0.37485144	-1.090851256	0.275338338	0.368902589
DERL1	107.538675	-0.215181782	0.197279582	-1.090745326	0.27538496	0.368928073
FECH	6.209319099	0.5595196	0.513150579	1.090361431	0.275553965	0.369117488
PITX1	12.94674486	-0.441716839	0.405263808	-1.089948894	0.275735657	0.369323859
BOLA1	10.45367488	-0.417011051	0.382860759	-1.089197683	0.27606672	0.369730239
LOC101118245	32.16806332	0.315204882	0.289605132	1.08839536	0.276420609	0.370092943
LOC105611919	1.868977811	1.099098899	1.009738885	1.08849814	0.276375257	0.370092943
ZNF394	3.375423583	0.792875222	0.728455098	1.088433898	0.276403603	0.370092943
EIF2B2	14.58419002	0.358305856	0.329227424	1.08832324	0.276452435	0.370098482
GALNS	4.550695762	0.624793748	0.574528001	1.087490509	0.276820091	0.370553564
EIPR1	28.8391819	0.294629413	0.271030255	1.087072042	0.277004972	0.370763916
ZNHIT1	36.62160423	-0.303039506	0.278816381	-1.086878416	0.277090546	0.370841318
RTL5	25.31541943	0.322192211	0.29656784	1.086403064	0.277300706	0.371085427
RRS1	21.58997593	-0.359269142	0.330762546	-1.086184473	0.277397385	0.371161512
SCRN2	4.586841295	-0.622929763	0.57355464	-1.086086171	0.27744087	0.371161512
SMIM11	24.73366587	-0.317020231	0.291883321	-1.086119721	0.277426028	0.371161512
LOC105603102	5.824371683	-0.587021607	0.540618266	-1.085833838	0.277552513	0.371273708
BTG2	48.38254916	0.21548656	0.198621434	1.084910908	0.277961116	0.371779332
PPM1G	93.42872166	0.31746378	0.292632606	1.084854433	0.277986133	0.371779332
CORO1B	41.91436464	-0.320831365	0.295785647	-1.084675232	0.278065521	0.3718111
TMCO2	1.880636382	-1.00755391	0.92887339	-1.084705322	0.27805219	0.3718111
LOC114114843	8.508671811	0.476955362	0.439901223	1.084232863	0.278261564	0.372036018
ZDHHC17	4.836871275	0.670644427	0.618657286	1.084032213	0.278350516	0.372117724
HSDL1	13.21490862	-0.399743051	0.368982833	-1.083364902	0.278646488	0.372476143
VPS4A	39.99024727	0.286375934	0.264381257	1.083193029	0.278722753	0.372540832
DENND6B	4.216481367	0.688212459	0.635407541	1.083104014	0.278762258	0.372556378
MTOR	48.18738234	0.224620848	0.207633134	1.081816006	0.279334293	0.373261372
PRRC2B	39.81315475	0.263938441	0.243982947	1.081790528	0.279345616	0.373261372
CCDC30	10.9467817	-0.46000828	0.425425141	-1.081290774	0.27956779	0.373483566
IDNK	3.242824372	-0.780724954	0.72201535	-1.081313512	0.279557679	0.373483566
LOC101116334	39.26204153	0.267107005	0.247064501	1.081122558	0.2796426	0.373546172
TMC7	4.633175664	0.62079446	0.574329192	1.080903546	0.279740021	0.373638965
RNF225	3.238417579	-0.767783337	0.710563292	-1.080527724	0.279907248	0.373824968

PAQR5	3.561273229	-0.701120103	0.649023904	-1.080268536	0.280022617	0.373941682
LOC105604630	29.07378706	0.288689458	0.267257002	1.08019418	0.28005572	0.373948526
EEF1G	207.7258805	-0.191866573	0.177756044	-1.079381433	0.280417724	0.374394497
MLN	1.474095731	1.259321517	1.166899519	1.079203048	0.280497221	0.37446323
DIPK1C	32.77023311	0.303934968	0.281807673	1.078519136	0.280802146	0.374832866
CCDC71L	37.302301	-0.257887791	0.239142291	-1.078386388	0.280861359	0.374874468
BLTP3B	75.40569477	0.203814634	0.189048726	1.07810636	0.280986294	0.374993167
MBD3	23.83700543	0.342922496	0.318091834	1.078061298	0.281006402	0.374993167
CDCP1	40.87630685	-0.249004939	0.231041274	-1.077750891	0.281144941	0.375140589
LOC114114600	2.233080802	0.919118542	0.853001841	1.077510619	0.281252209	0.37524626
RIMS2	3.972939573	0.711635764	0.660871575	1.076813999	0.28156337	0.375578609
SHQ1	10.3468793	-0.469810831	0.436317271	-1.076764233	0.281585608	0.375578609
TRMT10C	93.2928716	-0.22869866	0.21238367	-1.076818479	0.281561368	0.375578609
CREB1	94.92502006	-0.195197004	0.181327644	-1.076487845	0.281709134	0.375705876
MAGEF1	34.29715566	-0.300816613	0.279516039	-1.076205191	0.281835499	0.375836902
LOC101111217	1.650595863	-1.092495217	1.015334514	-1.075995351	0.281929335	0.375924531
BMPR2	3.373895677	0.723084728	0.672116499	1.075832432	0.282002205	0.375984186
CDNF	2.468279847	0.940996517	0.874811327	1.075656531	0.282080895	0.376013079
CFAP210	3.594484356	-0.784800288	0.729609761	-1.0756439	0.282086546	0.376013079
KNTC1	2.810578356	-0.862087498	0.801497983	-1.075595343	0.282108271	0.376013079
FTSJ1	24.03037784	-0.319458943	0.297080554	-1.075327681	0.282228047	0.376135216
SLC38A10	12.39123583	-0.447092734	0.416140113	-1.074380286	0.282652274	0.376663041
PTGES2	7.150764246	0.537261247	0.500283312	1.07391399	0.282861233	0.376903921
ZNF25	8.813570506	0.474893631	0.4426473	1.072848814	0.283338955	0.377502838
INTS8	4.674179639	0.617039204	0.576217149	1.070844914	0.284239168	0.378664479
SERINC2	7.867264886	-0.538939737	0.503429834	-1.070535955	0.284378134	0.378811854
PARVB	3.038332619	0.759799038	0.709901411	1.070288108	0.284489646	0.378922631
ZBTB6	14.38942041	-0.399697206	0.373578355	-1.069915321	0.284657426	0.379108326
MAMSTR	1.850383651	1.168339026	1.09207627	1.069832811	0.284694571	0.379112363
VAC14	7.103107777	0.512162452	0.478753779	1.06978258	0.284717185	0.379112363
AARS1	33.2778001	-0.26432178	0.247111175	-1.069647214	0.284778135	0.379155747
HTRA2	2.917241915	-0.831608587	0.777523403	-1.069560845	0.284817028	0.37916976
SLC2A8	18.40376257	0.343603628	0.321380578	1.069148704	0.285002669	0.379379113
RIN1	1.952546614	1.044174257	0.97709262	1.068654328	0.285225459	0.379637869
CPNE3	67.9754623	-0.27364498	0.256148582	-1.068305662	0.285382655	0.379743268
DFFA	15.50961261	-0.333773766	0.312423227	-1.068338513	0.285367842	0.379743268
LOC101104050	17.95410199	-0.377470138	0.353340632	-1.068289643	0.285389879	0.379743268
ELOA	21.33265097	0.354632745	0.331991672	1.068197714	0.285431336	0.379760624
C2H2orf76	1.99713402	1.016618973	0.951923624	1.067962752	0.285537314	0.379840528
DLL4	5.145444232	0.644913011	0.603885877	1.067938556	0.285548229	0.379840528
DYNC2LI1	7.731925148	-0.511021727	0.4786255	-1.06768596	0.285662194	0.379954312
BCLAF3	15.37799697	-0.364117524	0.341503097	-1.066220269	0.286324082	0.380786252

CUTA	42.75682342	-0.255071107	0.239239492	-1.066174754	0.286344652	0.380786252
SENP8	2.638608792	0.871562368	0.817838957	1.065689475	0.286564037	0.381040083
MSRB1	34.48244595	-0.246354224	0.23123663	-1.065377161	0.286705288	0.38111416
PLBD1	5.37187066	0.567631606	0.53275911	1.065456404	0.286669444	0.38111416
SLF1	22.19577991	-0.30719426	0.288335564	-1.065405375	0.286692525	0.38111416
IFIT5	14.67375155	-0.397150945	0.373131225	-1.064373385	0.287159586	0.381680094
SLC25A40	8.929166072	-0.473292119	0.444728695	-1.064226628	0.287226047	0.38173047
RAPGEF4	3.375797806	0.755131887	0.709847062	1.063795186	0.287421493	0.381952244
PAN2	4.303170049	-0.725141428	0.681970603	-1.063303059	0.287644539	0.382210647
LOC121819876	9.87592124	-0.476492248	0.448203277	-1.063116386	0.287729174	0.382285104
LOC132659947	3.746291274	-0.71290133	0.670901634	-1.06260187	0.287962539	0.382557131
CCP110	22.44922744	-0.32656588	0.307584512	-1.061711065	0.288366875	0.383039962
KIAA0232	42.52920316	0.238767047	0.224896572	1.061674903	0.288383297	0.383039962
TTC19	45.96300347	-0.246512568	0.232397546	-1.060736535	0.288809652	0.383568144
CFAP91	6.10722983	0.532382322	0.502181047	1.060140212	0.289080816	0.383890133
XPNPEP3	2.498564354	-0.972440943	0.917818558	-1.059513271	0.289366089	0.384230791
ELK1	10.78788933	-0.401978085	0.379575383	-1.059020429	0.289590476	0.384452354
PSMD8	126.4912092	0.215111355	0.203111892	1.05907809	0.289564217	0.384452354
ETAA1	35.12984199	0.277412749	0.261996608	1.058840995	0.2896722	0.384522656
SFR1	6.86951511	0.551768107	0.521530022	1.057979567	0.290064758	0.385005517
P2RX4	34.29463993	0.288626033	0.272852547	1.057809564	0.290142272	0.385070162
LOC105606428	2.548957612	-0.952373636	0.900407924	-1.057713522	0.290186069	0.385090051
LOC101106610	52.672769	0.256594706	0.242703001	1.057237469	0.290403223	0.385339966
API5	38.24517914	0.301935722	0.285670353	1.056937549	0.29054009	0.385483308
LOC114109448	9.005326435	0.493120011	0.466650281	1.056722841	0.290638097	0.385575068
ZBED4	2.528678314	-0.923171868	0.874161853	-1.05606515	0.290938451	0.385935226
MAP3K12	2.060996729	0.924911461	0.876671456	1.05502632	0.291413287	0.386526744
RINT1	15.33424265	-0.359256981	0.34095606	-1.053675304	0.292031599	0.38730843
TMEM115	1.69014771	1.005766954	0.954683436	1.053508332	0.292108078	0.387371422
TECPR1	5.254570032	-0.629175195	0.597329665	-1.053313157	0.292197491	0.387451554
LOC114114229	5.900049755	-0.573962512	0.544946673	-1.053245282	0.29222859	0.387454353
CLDND1	4.730719575	0.615283457	0.584270985	1.05307892	0.292304823	0.387516988
LYPD6B	13.07612616	-0.416091618	0.395183525	-1.052907298	0.292383481	0.387582823
SLC38A6	9.094069153	-0.461567881	0.438739937	-1.052030694	0.292785467	0.388077207
NUBP1	30.50425501	0.283418095	0.26944484	1.051859426	0.292864049	0.388142874
NDE1	2.127706194	0.910817308	0.865974856	1.051782625	0.292899292	0.388151095
MATR3	654.7624119	0.136289472	0.129609121	1.051542295	0.293009595	0.388258773
PGPEP1	8.747903021	0.468544741	0.44572551	1.051195703	0.293168716	0.388416076
TMEM35B	1.350347047	-1.162802452	1.106211827	-1.051157132	0.293186428	0.388416076
LDAH	16.75119411	-0.350460032	0.333464629	-1.050966132	0.293274145	0.388493778
ISY1	19.51032517	-0.348119738	0.331264753	-1.050880705	0.293313383	0.388507252
TUBGCP2	6.33717758	0.522502902	0.497573599	1.05010174	0.293671339	0.388942837

HERC4	66.7180552	0.206295601	0.196638322	1.049111889	0.294126625	0.389448004
RBMS2	15.21943359	-0.364436465	0.347371421	-1.049126216	0.294120032	0.389448004
RO60	34.93477528	0.252448361	0.240637295	1.049082442	0.294140176	0.389448004
LMBRD1	31.60437215	-0.257374975	0.245348279	-1.04901887	0.294169434	0.389448163
CACNA2D2	9.568467997	-0.503975322	0.480457986	-1.048947746	0.294202169	0.389452926
RHOG	2.818850903	0.748760865	0.714137871	1.048482226	0.294416487	0.389698036
MASTL	3.016517523	0.796699216	0.760042221	1.04823021	0.294532555	0.389774468
PGM1	8.354556754	-0.482033171	0.459841035	-1.048260452	0.294518625	0.389774468
STIP1	85.21452447	0.180327637	0.172062566	1.04803527	0.294622357	0.38985471
DYNC2I1	25.1130556	-0.389553553	0.371919309	-1.047414166	0.294908601	0.390181725
STK26	13.26133087	-0.39512947	0.377257873	-1.047372361	0.294927874	0.390181725
SLC25A51	56.75442154	0.23667078	0.226141592	1.046560158	0.295302486	0.39063866
YRDC	37.95649623	-0.305398339	0.291850117	-1.046421849	0.29536631	0.390684423
WDR45B	25.37569154	-0.302702213	0.289311712	-1.046283993	0.295429933	0.390729912
FKBP1A	38.10812806	-0.232239841	0.222024271	-1.046011052	0.29555593	0.390857876
C2H9orf85	7.259418156	-0.57047227	0.54569313	-1.045408561	0.295834182	0.391187146
CPLX2	2.620721449	-0.801289521	0.76661702	-1.04522793	0.295917638	0.391258794
NADK2	72.15901162	-0.237878331	0.227662501	-1.044872694	0.296081812	0.391398427
RNF44	46.18998846	-0.220413156	0.210939029	-1.044914054	0.296062694	0.391398427
ERCC4	15.01810699	0.343282409	0.328563127	1.044798946	0.296115902	0.391404781
NCAPG2	30.40029492	-0.267314804	0.255976905	-1.044292666	0.296350005	0.391675484
SPECC1L	18.15227156	-0.311451919	0.298286534	-1.044136707	0.296422145	0.391703182
TMEM17	7.778624309	-0.482021312	0.461652888	-1.044120648	0.296429574	0.391703182
LOC114117337	4.315668934	0.651703683	0.62422654	1.044017902	0.296477107	0.391727265
DNAJB1	43.12989803	-0.271005135	0.259605065	-1.043913127	0.296525584	0.39175259
TXNDC12	25.82460838	0.300003105	0.287429036	1.04374669	0.296602602	0.391815614
SCNN1A	4.961853958	0.602814441	0.577630955	1.043597881	0.296671474	0.391867864
ABRAXAS2	11.3436211	0.417610128	0.400501881	1.042717021	0.297079373	0.392367875
PROCA1	20.95127977	-0.356191512	0.341730924	-1.042315714	0.297265329	0.392574685
SRP68	45.73566683	0.225390482	0.216363024	1.041723659	0.297539817	0.392898359
CHUK	29.24723218	0.290353819	0.278742764	1.04165509	0.297571618	0.392901535
CCDC149	10.70670799	-0.437440309	0.419990686	-1.041547643	0.297621454	0.392928521
FIG4	6.460916446	-0.512817255	0.49249514	-1.041263585	0.297753232	0.393063674
ADGRA3	17.57475236	-0.377064189	0.362224076	-1.040969425	0.297889739	0.393203708
NPDC1	21.04706496	0.363241854	0.348987515	1.040844841	0.297947565	0.393203708
STAU2	31.45688527	0.27995056	0.268961958	1.040855598	0.297942572	0.393203708
OGFOD2	5.809678626	-0.633987334	0.609223395	-1.040648373	0.298038772	0.393285243
LSM10	7.505060142	0.517448891	0.497526729	1.040042395	0.298320205	0.393617755
BICD1	4.677434612	-0.608931315	0.58567761	-1.039703934	0.298477472	0.393786388
LOC132657470	2.589630805	0.985859806	0.9487516	1.039112668	0.29875234	0.394110124
ATP6V0B	70.87085254	-0.348268845	0.335269522	-1.038772756	0.298910435	0.394279766
ALG1	4.455042359	0.632365909	0.60900542	1.038358425	0.299103218	0.394464739

DDX56	30.84202968	0.293205679	0.282395276	1.038281103	0.299139204	0.394464739
LOC132659164	2.278876167	0.866329358	0.83435458	1.03832277	0.299119811	0.394464739
TBCE	19.29463482	-0.318279064	0.306859725	-1.037213546	0.299636346	0.395081326
TIGD2	8.005219656	0.49350044	0.475920835	1.036938085	0.299764713	0.395211596
NUP54	17.33047646	0.340073484	0.32803604	1.036695493	0.299877793	0.395321687
LOC114110140	34.56727632	-0.345808583	0.333701158	-1.036282238	0.30007049	0.395536703
RTKN	1.918223408	-0.945889098	0.913826981	-1.035085544	0.300628963	0.396233776
FAM171B	1.41858646	1.225964045	1.184890233	1.034664656	0.300825548	0.396446705
THAP7	2.25942743	0.95328082	0.921389089	1.034612664	0.300849837	0.396446705
PARVA	20.04116504	0.347490849	0.336165949	1.033688422	0.301281848	0.396976851
FYCO1	42.54446148	-0.25548007	0.247185674	-1.033555326	0.301344093	0.397019729
HSPA4L	53.24255077	0.23141798	0.223923432	1.033469245	0.301384356	0.397033639
NGEF	5.029279861	-0.591657925	0.572595253	-1.033291704	0.301467408	0.397103911
LOC121816535	1.908558145	1.011353511	0.979136678	1.032903306	0.301649152	0.397304155
TRIM36	8.801166767	0.44145402	0.427955711	1.031541369	0.302287019	0.398105064
EHD1	167.5833098	-0.204744058	0.198506281	-1.031423572	0.302342232	0.398138549
ALDH3B1	3.348985105	-0.714034827	0.692353313	-1.03131568	0.302392808	0.398152175
GFOD1	17.44840446	-0.374753478	0.363388711	-1.031274407	0.302412157	0.398152175
PIGK	10.97060119	-0.458526948	0.444662655	-1.03117935	0.302456722	0.398171629
MAFK	6.389531775	-0.49761071	0.483029043	-1.030187971	0.302921775	0.398705313
NR2C2AP	3.969186444	-0.709312065	0.688500215	-1.030227805	0.30290308	0.398705313
MOB1A	67.2173171	-0.255834215	0.248381901	-1.030003452	0.303008385	0.398780039
POLR3C	5.602143425	0.533718962	0.518550845	1.029250973	0.303361755	0.399205791
FOXRED2	1.864745799	0.987388023	0.959398974	1.029173524	0.303398141	0.399214369
LOC105613472	2.115137456	0.873388249	0.849159483	1.028532644	0.303699343	0.399571358
CPLANE2	5.010420763	-0.566591059	0.551000414	-1.028295159	0.303811007	0.39967893
LOC105610542	2.437096363	0.910856073	0.885957442	1.028103642	0.303901078	0.399758075
FBRSL1	16.7055898	-0.320854535	0.312180858	-1.027784141	0.304051378	0.399900939
LOC114110621	3.815020661	0.671968104	0.653867744	1.02768199	0.304099442	0.399900939
SAP30L	46.10825408	0.212035028	0.206318623	1.027706684	0.304087823	0.399900939
IL22RA1	7.392358828	-0.502532234	0.489099092	-1.027465073	0.304201523	0.399995825
SESTD1	20.68932586	-0.300904102	0.292982557	-1.027037598	0.304402761	0.40022106
TXNDC9	11.64149308	-0.406819389	0.396303914	-1.026533868	0.304640008	0.400493592
LOC105605752	5.485742188	-0.550859893	0.536747289	-1.026292828	0.304753577	0.400603492
UFD1	128.8693769	0.168406253	0.164179082	1.02574732	0.305010703	0.400902061
SLC25A43	3.255852124	-0.70748579	0.689830616	-1.025593491	0.305083237	0.400957969
OST4	32.43892724	-0.32584681	0.31775147	-1.025476955	0.305138194	0.400990767
MIEF2	3.621378544	-0.715402325	0.697744822	-1.025306535	0.305218573	0.401017542
ST18	2.345757142	0.938792306	0.915597772	1.025332668	0.305206246	0.401017542
FAM184A	2.480346264	-0.823044395	0.803098777	-1.024835822	0.305440661	0.401230454
SUCLG2	348.9207697	0.161620147	0.157700551	1.024854675	0.305431764	0.401230454
ZNF134	5.462784087	-0.565429102	0.551929026	-1.024459805	0.305618147	0.40142415

CASP2	4.610311741	-0.654791529	0.639970623	-1.023158729	0.306232803	0.402191966
MED15	10.02097408	-0.421817877	0.412748617	-1.021972841	0.306793755	0.402889107
IGBP1	27.56095383	-0.271295044	0.265556401	-1.021609883	0.306965578	0.403075146
PDCD11	69.85046708	0.21429913	0.209781576	1.021534557	0.307001245	0.403082381
SHMT1	5.036828088	-0.68452195	0.670386165	-1.021086033	0.30721368	0.403321682
ADNP	33.51614423	-0.252860359	0.247676828	-1.020928605	0.307288265	0.403351753
POLRMT	2.021753831	0.898143815	0.879748023	1.020910297	0.30729694	0.403351753
MCCC2	30.4013996	0.276210931	0.270725186	1.020263151	0.307603675	0.403714721
CCDC137	37.86626942	-0.299708569	0.293887412	-1.019807438	0.307819795	0.403958702
CDC7	39.74230319	-0.244374373	0.239767652	-1.019213273	0.308101727	0.404288993
NOM1	20.48476679	-0.289093722	0.283746883	-1.018843692	0.308277179	0.404479511
LOC132659905	3.604739467	-0.687608992	0.675132658	-1.018479825	0.308449984	0.404666518
IL15RA	2.032298343	0.92011332	0.903927532	1.017906068	0.308722598	0.404984419
RHOT1	26.91083092	0.299857239	0.294622354	1.017768119	0.308788166	0.40503068
SHKBP1	5.914164911	-0.563911827	0.554111882	-1.017685859	0.30882727	0.405042222
HPCAL1	12.14400692	-0.406108254	0.399128587	-1.017487264	0.308921688	0.405126303
ST13	765.0712776	-0.149887451	0.147434201	-1.016639628	0.309324895	0.40561528
FBXO11	108.2239641	0.214149011	0.210688234	1.01642606	0.309426541	0.405708765
ATP5PB	37.77279093	-0.266190889	0.261955629	-1.016167852	0.309549462	0.405830124
ABT1	36.2739959	0.257291588	0.253229826	1.016039826	0.309610422	0.405863754
FBXO5	18.67782136	0.379146747	0.373180913	1.015986439	0.309635844	0.405863754
AP3S2	31.83264226	0.279440838	0.275161098	1.01555358	0.30984202	0.406094179
AGO2	118.9727868	0.179584305	0.176914511	1.015090872	0.310062512	0.406343322
TBL2	41.23762277	-0.214434871	0.211336634	-1.0146602	0.310267832	0.406572534
LOC101123619	24.82519861	0.301151759	0.296850878	1.014488355	0.310349783	0.406640055
MSLN	5.070987429	1.330791513	1.312126924	1.014224683	0.310475553	0.406725105
SNX2	48.69512299	0.224778126	0.221617811	1.014260204	0.310458608	0.406725105
TENT2	31.3407061	0.292612241	0.288561386	1.014038108	0.310564569	0.406801845
MTX2	22.44831618	-0.317684551	0.313343493	-1.013853992	0.310652428	0.406877055
CLRN3	7.091961701	0.486826666	0.48038221	1.013415268	0.31086185	0.407111453
ERGIC3	50.70710562	0.236147499	0.233056523	1.013262772	0.310934664	0.407166917
MYBL2	9.128257054	0.461988582	0.456159757	1.012778034	0.311166195	0.407430189
ENTPD4	48.825509	-0.231240089	0.228408805	-1.012395686	0.3113489	0.407629484
ACTMAP	3.129315011	-0.730276572	0.721807433	-1.01173324	0.311665618	0.408004177
NISCH	15.53379318	-0.325932416	0.322186866	-1.011625394	0.311717199	0.408031739
SLC17A5	27.34842527	0.313958501	0.310373789	1.011549661	0.311753425	0.408039197
FARS2	41.08573316	0.256349452	0.253444075	1.011463583	0.311794603	0.408053134
NOTCH3	5.459120839	-0.616714659	0.609828418	-1.011292095	0.311876648	0.408120548
ALG8	7.724521447	-0.520241716	0.514704319	-1.010758404	0.312132076	0.408414814
FAM83F	23.30547449	-0.295206887	0.292204525	-1.010274865	0.31236362	0.408677774
PBK	8.420566058	-0.432587563	0.428235627	-1.01016248	0.312417452	0.408708198
LOC101106404	11.38593502	0.37927184	0.375483383	1.010089545	0.312452391	0.408713902

SOS1	51.78596206	0.193672887	0.191912633	1.009172165	0.312892073	0.409248992
TAPT1	21.64920914	0.299678104	0.297219181	1.008273097	0.313323375	0.409773016
PML	24.17309517	-0.32557711	0.323150133	-1.00751037	0.313689577	0.410211807
CPOX	19.37865924	-0.32194677	0.319588407	-1.007379376	0.313752498	0.410253951
HIVEP1	18.03647633	0.307392088	0.305180129	1.007248046	0.31381559	0.410296309
NLGN2	4.809811439	-0.576562522	0.572494011	-1.007106644	0.313883529	0.410344997
CRYZL1	7.792271881	-0.441182805	0.438339149	-1.006487342	0.314181198	0.410693974
VPS4B	187.4038612	0.17864117	0.177612088	1.005793987	0.31451468	0.411089694
LOC132659149	4.459514084	-0.601420276	0.598120362	-1.00551714	0.3146479	0.411223608
CDC26	2.612647556	-0.970330027	0.965081405	-1.005438528	0.314685736	0.411232845
NAA60	9.448281401	-0.410315649	0.408310359	-1.004911191	0.314939615	0.411484154
PDIA6	139.2286577	-0.305506483	0.304004701	-1.004939997	0.314925744	0.411484154
HGH1	2.262129182	-0.825972575	0.822081125	-1.004733657	0.315025117	0.411525862
OSBPL10	3.577686886	-0.650715495	0.647660447	-1.004717051	0.315033115	0.411525862
PWWP2B	7.708226196	0.453244671	0.451344875	1.00420919	0.315277793	0.411805236
LOC101110185	13.76472902	-0.39529843	0.393825365	-1.003740401	0.315503758	0.412060116
ILF2	17.33348972	0.367034069	0.365777938	1.003434135	0.315651441	0.412212717
LOC114116364	2.318121906	0.833460234	0.830803747	1.00319749	0.315765584	0.412321492
LOC101109892	3.395174252	-0.729649567	0.727618736	-1.002791064	0.315961681	0.412456669
LOC114113953	3.703404155	0.646624177	0.644803164	1.002824138	0.31594572	0.412456669
SLK	86.36671002	-0.159379455	0.158932475	-1.002812386	0.315951391	0.412456669
MOB3C	9.137706035	0.436158768	0.435047291	1.002554843	0.316075693	0.412565206
SF3A3	142.9620015	-0.14652003	0.146183006	-1.002305492	0.316196071	0.412641738
SMAD6	10.19236609	-0.448085496	0.447052829	-1.002309945	0.316193921	0.412641738
CAAP1	39.83583652	0.224280911	0.2238209	1.002055266	0.316316901	0.412759124
DYNC2I2	24.54455656	0.353263345	0.352602576	1.001873978	0.316404462	0.412833077
SLC66A2	18.05070563	0.337561234	0.337202404	1.001064139	0.316795801	0.413303336
PARK7	48.65333134	-0.282351087	0.282145438	-1.000728876	0.316957903	0.413474461
ZDHHC13	7.370186847	-0.60351548	0.603174738	-1.000564914	0.3170372	0.413537544
RNASEL	60.5296413	-0.241660901	0.24176806	-0.999556769	0.317525053	0.414133476
LOC101111915	1.802325955	0.988611768	0.989193183	0.999412233	0.317595036	0.414184336
ZNF638	211.9643225	0.148360022	0.148593251	0.99843042	0.318070689	0.414764178
MAIP1	5.073097755	0.584655802	0.585703988	0.998210382	0.318177353	0.414862794
TRAPPC5	29.34273483	-0.269183996	0.269695644	-0.998102869	0.318229479	0.414890287
AIDA	30.98421505	0.237907184	0.238444095	0.997748273	0.318401439	0.415033512
RFWD3	14.35912738	-0.377373842	0.37821735	-0.997769779	0.318391008	0.415033512
PPP4C	7.643979669	0.561330652	0.562675804	0.997609366	0.318468817	0.41508086
RAI1	23.22040203	0.289455559	0.29033948	0.996955561	0.318786081	0.415413353
UBA6	24.74477353	0.28571392	0.286570753	0.997010048	0.318759633	0.415413353
GZF1	4.833737243	0.563415696	0.565201403	0.996840583	0.318841896	0.415445582
FAM117B	16.86499032	0.333478445	0.334620422	0.996587245	0.3189649	0.415565343
NFIC	160.3915525	0.174324778	0.174959203	0.996373871	0.319068524	0.415659833

FIRRM	2.046901088	-0.972856331	0.976507108	-0.996261393	0.319123157	0.41569049
GNAO1	4.895549964	-0.622475867	0.625188482	-0.995661124	0.319414825	0.416029872
MLLT6	16.35827135	-0.308255641	0.309685591	-0.995382573	0.319550231	0.416165682
SETDB2	48.95497434	0.228952098	0.230176259	0.994681637	0.319891128	0.416569059
PTDSS2	12.64760836	-0.354969112	0.357122448	-0.993970315	0.32023732	0.416979251
GJA9	4.070656211	0.629591165	0.633483026	0.993856409	0.320292779	0.41701084
GOLPH3L	19.64702688	-0.322667333	0.325028454	-0.992735649	0.320838797	0.417559042
LRRN2	4.732651713	0.571808073	0.575990238	0.992739173	0.320837079	0.417559042
MIIP	8.589865659	-0.446933449	0.450151097	-0.992852071	0.32078205	0.417559042
TECR	8.79797455	0.441183654	0.444352741	0.992868084	0.320774245	0.417559042
TRIQK	7.10852777	-0.518434296	0.522427119	-0.992357167	0.321023325	0.417758521
NFATC3	46.60725692	-0.231171438	0.233001721	-0.992144768	0.321126911	0.417852637
CTSF	7.24960918	-0.540113113	0.544498059	-0.99194681	0.321223473	0.417937597
FEM1A	14.21003846	0.357424675	0.360388343	0.991776458	0.321306584	0.418005042
CDIPT	9.591733352	-0.432993377	0.436616975	-0.991700741	0.321343529	0.41801242
HEPH	7.921728515	0.532862044	0.537586672	0.991211413	0.321582359	0.418282387
SIX5	2.197659509	-0.850752635	0.859032744	-0.990361126	0.321997639	0.418781789
GSTCD	3.013236574	-0.692924412	0.700027303	-0.989853408	0.322245776	0.419063732
UPF3A	14.89667364	0.367191535	0.37101721	0.989688685	0.322326307	0.41912768
LOC121816105	2.416056267	0.775746183	0.784016085	0.989451872	0.322442106	0.419219694
PRMT9	11.25707656	-0.373258789	0.377251737	-0.989415694	0.322459799	0.419219694
SLC35C1	35.78939585	0.257286652	0.260108703	0.989150494	0.322589516	0.419347546
RAB31	4.602116301	0.570159988	0.576533426	0.988945242	0.322689934	0.41943729
SETMAR	1.298007373	-1.21090747	1.224564383	-0.988847534	0.322737744	0.419458643
MYCBPAP	1.679660613	0.992726585	1.004107395	0.988665744	0.322826708	0.419533474
HPCAL4	4.415867449	0.603246277	0.610254692	0.98851559	0.322900203	0.419588189
CFAP96	2.151735911	-0.869009672	0.87959714	-0.987963277	0.323170634	0.419898774
SKA2	46.75727043	-0.235422564	0.238506103	-0.987071445	0.323607615	0.420425679
NCBP3	41.05146451	-0.291176319	0.295125184	-0.986619695	0.323829112	0.420672554
CCNDBP1	2.599061565	0.794660085	0.805690831	0.98630896	0.323981524	0.420829646
LOC105605852	5.405439507	0.565407747	0.573366768	0.986118795	0.324074822	0.420909928
LOC105603320	3.637023573	0.640558961	0.649639173	0.986022684	0.324121982	0.420930277
SPEN	74.58346604	-0.189974549	0.192769711	-0.985499989	0.324378537	0.421222532
NQO1	4.233876165	-0.65530101	0.665039939	-0.985355874	0.324449296	0.421273489
GCAT	6.307166539	0.542753162	0.550996097	0.985039939	0.324604453	0.421434009
RALGPS1	34.99391481	-0.269673525	0.273894858	-0.984587759	0.324826605	0.421681468
RAD51D	4.287792202	-0.604540116	0.614157567	-0.984340417	0.324948164	0.421798306
RBMX2	23.70911574	-0.295485877	0.300282038	-0.984027815	0.325101837	0.421956803
YJU2B	4.135552375	-0.678976933	0.69025368	-0.983662895	0.32528129	0.422148725
MAP2K7	26.29170758	-0.27639226	0.28101172	-0.983561325	0.325331249	0.42217257
NEUROD1	10.47170195	-0.394385095	0.401006229	-0.9834887	0.325366974	0.422177942
UBLCP1	67.20019409	0.184041806	0.187193958	0.983161038	0.325528187	0.422346122

KRT23	2.712972947	0.824746558	0.839208659	0.982766979	0.325722137	0.42255674
MFN2	18.44534587	-0.370573951	0.377271282	-0.98224797	0.3259777	0.422847238
CNPY2	49.73456965	-0.290799979	0.296312638	-0.981395803	0.326397595	0.423350825
ZNF462	34.26173412	0.25403718	0.25887756	0.981302435	0.326443622	0.423369441
CC2D1A	2.926848426	0.756855749	0.771336152	0.981226858	0.326480882	0.423376683
TMEM70	26.36381745	-0.291572626	0.297308229	-0.980708225	0.326736647	0.423667251
PI4K2A	4.182656647	0.608936955	0.621086641	0.980438017	0.326869951	0.423798988
PTPRK	17.82291743	-0.308937944	0.315125941	-0.980363416	0.326906762	0.423805604
LARP4B	45.51106045	0.22611803	0.23075575	0.979902043	0.327134475	0.424059683
LOC101118051	13.85181325	-0.411340125	0.419871734	-0.979680438	0.327243885	0.424160373
RAMP1	3.696855379	0.644773652	0.658452681	0.979225495	0.327468574	0.424410449
MICOS13	28.15502007	0.337754153	0.344993536	0.979015888	0.327572129	0.424462344
TMPRSS3	5.222853491	0.732483265	0.748174751	0.979026977	0.32756665	0.424462344
CSF1	3.896540317	0.661945345	0.676456543	0.978548218	0.327803256	0.424720659
SELENON	3.409627293	-0.688588376	0.703807008	-0.978376698	0.327888049	0.424789344
LOC101118895	3.462883956	-0.706453164	0.722145584	-0.978269728	0.327940938	0.424816687
FKTN	9.502558068	-0.43290146	0.442668613	-0.977935744	0.328106105	0.424908865
LOC105607546	10.11455	0.412132168	0.421407115	0.977990529	0.328079008	0.424908865
STAG3	2.79369411	-0.929621355	0.950598214	-0.977932992	0.328107466	0.424908865
ARHGEF17	2.336454741	0.916377516	0.937699285	0.977261613	0.328439652	0.425076564
GPAM	26.08462034	0.250723474	0.256544501	0.977309871	0.328415767	0.425076564
IZUMO4	9.734807664	0.428803418	0.438713194	0.97741172	0.328365363	0.425076564
MKLN1	31.37632894	0.240976544	0.246593614	0.97722135	0.328459581	0.425076564
RHOV	3.349634439	-0.686568993	0.702349636	-0.977531643	0.32830602	0.425076564
SETD2	103.7884643	0.178305793	0.182431273	0.977386117	0.328378034	0.425076564
ZNF329	32.59369296	0.274707735	0.281064012	0.977384948	0.328378612	0.425076564
ELOC	26.27920478	0.254411385	0.26066971	0.975991363	0.329068744	0.425823685
ASXL2	118.8642586	0.153939667	0.157770863	0.975716713	0.329204868	0.425876139
LOC101113531	1.488262864	0.956942751	0.98070988	0.975765382	0.329180743	0.425876139
ZC3HAV1L	7.201632447	-0.483419601	0.495405086	-0.975806698	0.329160265	0.425876139
LOC121817701	5.691074685	0.522544779	0.535684179	0.975471742	0.329326312	0.425950792
TMEM268	23.06811238	-0.296254577	0.303690863	-0.975513634	0.329305542	0.425950792
DCAF6	98.70136962	0.178760802	0.183306074	0.975203924	0.329459116	0.42608133
SLAMF8	1.313712325	1.080226239	1.108254418	0.974709616	0.329704322	0.426357195
LOC101110611	4.650377133	0.634207035	0.651053539	0.974124242	0.329994854	0.426691615
LOC121819716	1.112153256	1.282423455	1.316843664	0.973861583	0.330125271	0.426818956
DENND1B	22.27261458	0.269179858	0.276527037	0.97343052	0.330339376	0.427013163
EIF3I	43.15030243	-0.227859535	0.234070296	-0.973466261	0.330321621	0.427013163
LOC101111954	5.691741821	-0.597422923	0.613809502	-0.973303478	0.330402494	0.427053451
SMG5	2.666650536	0.769556621	0.79083703	0.973091285	0.330507935	0.42714843
NUP107	27.36077236	-0.251970389	0.259091499	-0.972515074	0.330794369	0.427477283
CTDSPL	56.68497941	-0.216362564	0.222512072	-0.972363262	0.330869862	0.427533505

TMEM179B	12.19378455	0.352783222	0.362880743	0.972173995	0.330963995	0.4276138
ESYT1	4.02956067	-0.661142119	0.680619269	-0.971383194	0.331357494	0.42808083
MRTO4	32.51311913	0.231449377	0.238371952	0.970958934	0.331568729	0.428312325
TGFBR1	24.06493835	0.272810747	0.281076958	0.970590934	0.331752022	0.428507686
RFT1	6.575321858	-0.489943331	0.504996804	-0.970190954	0.331951319	0.428723677
ESF1	173.9703837	0.157367034	0.162305066	0.96957561	0.332258075	0.429078399
TBXAS1	8.313169591	0.421546313	0.434880179	0.969338988	0.332376083	0.429189327
LOC105603426	2.455109734	-0.802976933	0.828486326	-0.969209639	0.332440603	0.429231172
SBF2	15.66202521	-0.33907598	0.349922066	-0.969004281	0.332543053	0.429319561
WDR19	4.556719591	0.572571318	0.590923231	0.968943659	0.332573301	0.429319561
ANXA2	131.6335991	-0.168563826	0.173998656	-0.968765101	0.332662403	0.429356889
TTC27	13.25527445	-0.339552023	0.350502794	-0.968756965	0.332666463	0.429356889
LOC101107920	2.501424945	-0.781125211	0.806388586	-0.968670966	0.332709383	0.429370823
PRR14L	51.05899162	0.196469082	0.202900744	0.968301435	0.332893848	0.429567404
PGM2L1	8.767151626	-0.43315767	0.447370603	-0.968230069	0.332929481	0.429571912
ZC3HC1	29.89045495	-0.260925655	0.269550367	-0.968003338	0.333042703	0.429645987
ZNF317	5.080105861	-0.573277204	0.592236859	-0.967986364	0.33305118	0.429645987
SPAG4	3.700299339	-0.632909556	0.65399708	-0.967755936	0.333166275	0.429752985
LOC132659897	8.064982761	-0.504430711	0.521492213	-0.967283303	0.333402428	0.430016101
ATP2A2	58.77372498	-0.201093326	0.207968269	-0.966942349	0.333572854	0.430194401
SOWAHD	4.50657364	-0.601292539	0.622952765	-0.965229746	0.334429751	0.431257892
NSD1	83.83319185	0.178526908	0.184978511	0.965122422	0.334483498	0.43128559
LAMTOR5	57.06570076	0.201989066	0.209510225	0.964101231	0.334995175	0.431903686
NUCKS1	847.619541	-0.163677001	0.169904925	-0.963344651	0.335374593	0.432351158
DUSP8	3.10626756	-0.658150808	0.68391848	-0.962323475	0.335887141	0.432970153
RAB7A	30.08699818	-0.239158666	0.248567931	-0.962146103	0.335976218	0.433043214
BCAM	8.1652734	0.474262386	0.493372667	0.961266032	0.336418423	0.433571366
FANK1	1.180239624	-1.260001483	1.311307693	-0.960874012	0.336615519	0.433783555
ZNF800	70.47459101	0.190698213	0.198478895	0.960798445	0.336653521	0.433790703
AGPS	26.58321517	-0.273740481	0.284990617	-0.96052454	0.336791286	0.433926386
GALK2	3.172478224	-0.706596875	0.735877179	-0.960210339	0.336949364	0.434088213
MAPKAPK3	13.0277325	-0.396153102	0.412727855	-0.959840963	0.337135262	0.434285844
WDR43	218.9054628	-0.174309537	0.181678905	-0.959437404	0.337338439	0.434505694
LOC132658027	3.860171889	-0.738060766	0.76974763	-0.958834736	0.337642007	0.434854796
SEC23A	32.65299512	0.28465452	0.296916854	0.958701116	0.337709336	0.434857706
SPOP	66.41397103	0.188021951	0.196118339	0.958716824	0.337701421	0.434857706
CEP97	17.52837452	0.295492243	0.308286443	0.958498985	0.337811203	0.434946974
MLYCD	12.21018494	-0.367263004	0.383691365	-0.957183397	0.338474696	0.435759274
EXOC6B	9.487072482	-0.388355532	0.405987402	-0.9565704	0.338784135	0.436115645
LOC101114342	60.63856922	0.25317068	0.264721291	0.956366899	0.338886902	0.436205926
DEAF1	10.71532258	-0.40477574	0.423388683	-0.956038166	0.339052953	0.436337892
LPIN3	27.87364839	-0.290980806	0.304362189	-0.956034674	0.339054717	0.436337892

TSTD1	194.8156668	-0.205551029	0.215152387	-0.955374151	0.339388521	0.436725424
LOC132658146	5.077645989	-0.631042573	0.660888311	-0.954839967	0.339658633	0.437030928
CCNL1	74.74560489	-0.192868313	0.202189308	-0.953899666	0.340134433	0.437558886
TPPP3	2.38711882	0.828225965	0.868249827	0.953902827	0.340132832	0.437558886
TCP11L1	9.096795676	-0.420198425	0.440688867	-0.953503608	0.340334969	0.437774728
C24H7orf50	56.43638639	0.195715031	0.205280003	0.953405242	0.340384786	0.437796676
FOXM1	2.084591057	-0.850040995	0.891698225	-0.953283265	0.340446568	0.437834007
TMEM50B	22.34677046	-0.377217086	0.395953006	-0.952681457	0.340751491	0.438183994
ITPRIP	2.311340371	0.81856271	0.859491324	0.952380422	0.340904085	0.438338047
CANT1	46.32875393	-0.237078534	0.249068091	-0.951862331	0.341166806	0.438591471
CTNNAL1	37.71583684	-0.254756108	0.267624626	-0.951915792	0.34113969	0.438591471
DIAPH2	183.56477	0.145491318	0.152896216	0.951569116	0.341315551	0.438740494
CCRL2	3.245215166	0.677978603	0.712683471	0.951303952	0.341450101	0.438871243
ZNF527	5.751964915	-0.490224981	0.515440908	-0.951078918	0.341564316	0.438975831
ELMO2	11.32325286	-0.394444604	0.41511841	-0.95019781	0.342011751	0.439508611
ARRDC5	2.478842783	0.759886793	0.800430571	0.949347539	0.342443883	0.440021624
BLMH	100.1444526	-0.159768533	0.168352629	-0.949011217	0.342614907	0.440146372
DNMT3B	1.760086984	0.901016962	0.949475978	0.948962358	0.342639757	0.440146372
PIH1D2	2.448773961	-0.779260672	0.821094832	-0.949050758	0.342594797	0.440146372
RBPJ	162.4527731	0.175997413	0.185508549	0.948729397	0.342758259	0.440256283
XRCC2	3.989325775	-0.617340664	0.650789333	-0.948602923	0.342822604	0.44029662
WDR5B	4.448631162	-0.594774322	0.627100646	-0.94845114	0.342899835	0.440311192
ZNF277	3.327844859	0.683633532	0.720746653	0.948507398	0.342871209	0.440311192
CDC73	45.57261039	0.206477653	0.217932432	0.947438851	0.343415202	0.440930604
TMX4	18.39565283	-0.291943666	0.308173298	-0.947336023	0.34346758	0.440955497
LOC132658430	10.06673665	0.381955455	0.403502458	0.94660007	0.343842608	0.441353028
MPZL1	32.01388761	0.240433483	0.253997246	0.946598782	0.343843265	0.441353028
COPE	23.96373657	-0.253144936	0.267554487	-0.94614349	0.344075404	0.44160859
LOC105605445	5.545148295	0.536192488	0.566774754	0.946041588	0.344127375	0.441632885
GMNN	31.77865749	-0.245999331	0.260221652	-0.945345361	0.344482587	0.4420463
AHI1	66.7304616	0.20503914	0.217071157	0.944571092	0.344877891	0.442489082
CCL24	13.45631713	0.388024992	0.410808497	0.944539838	0.344893854	0.442489082
APOBEC1	24.51890578	0.26379952	0.279603837	0.943476037	0.345437467	0.443143985
NUCB1	40.42219618	0.219299501	0.232466869	0.943358086	0.345497774	0.443178815
SLC25A38	8.855712996	0.438596734	0.465019257	0.943179724	0.345588983	0.443253272
CFLAR	74.23527592	0.160153352	0.169859488	0.942857856	0.345753614	0.443421877
NUF2	18.56680195	-0.288405506	0.305910435	-0.942777598	0.345794672	0.443431986
CSTF2	26.18077704	-0.239688326	0.254256161	-0.942704103	0.345832274	0.443437661
RPA4	27.97706297	0.251560803	0.267041137	0.942030154	0.346177202	0.44379479
UVRAG	11.82886754	0.40228339	0.427010226	0.942093106	0.346144974	0.44379479
CYSTM1	28.4384881	-0.371264929	0.39429521	-0.941591275	0.346401939	0.44404031
BATF2	4.316969806	0.596180392	0.633230081	0.941490953	0.346453323	0.44406359

HECTD3	14.63966665	0.33828214	0.359547881	0.940854218	0.346779572	0.444439137
STEAP1	2.292341495	-0.759344888	0.807391582	-0.94049146	0.346965528	0.444634828
CARD11	1.823831709	0.879084277	0.935034693	0.940162202	0.347134366	0.444808546
CACNA1A	12.85717679	-0.398613677	0.424527973	-0.938957391	0.347752621	0.44551534
LOC101112784	5.266521882	-0.504744556	0.537541173	-0.938987712	0.347737053	0.44551534
LOC121816808	6.408798088	-0.475420561	0.506425524	-0.938776855	0.347845325	0.445591394
ANKRD49	78.47627327	0.273165609	0.291148776	0.938233755	0.348124295	0.445828225
GMPR	2.476887016	-0.739979112	0.78872726	-0.938193911	0.348144767	0.445828225
NOP58	275.4842308	-0.193555722	0.206314806	-0.938157209	0.348163625	0.445828225
SERPINB5	2.446025694	-0.767275785	0.817703405	-0.938330182	0.348074753	0.445828225
ZNF639	11.88816132	-0.33568785	0.357891725	-0.937959237	0.348265359	0.445915776
RPS19BP1	70.32053243	0.195789661	0.208755576	0.937889492	0.348301204	0.445918955
TLR6	2.157238685	0.980618831	1.045671177	0.937788908	0.348352902	0.445942428
ZZZ3	91.69988466	0.18321974	0.195476579	0.937297659	0.348605467	0.44622301
SMPDL3A	2.764823889	0.707327838	0.754722663	0.93720233	0.348654491	0.446243027
LOC101110168	2.56481933	-0.790907558	0.844011895	-0.937081056	0.348716865	0.446280124
DYNLT3	58.02426014	0.202530495	0.216188253	0.9368247	0.348848738	0.446406149
DNA2	2.259829191	-0.741390434	0.791528764	-0.936656339	0.348935362	0.446433385
PDGFC	4.461871824	-0.569435606	0.607946915	-0.9366535	0.348936823	0.446433385
TPCN1	17.20810092	0.360662225	0.38534413	0.935948409	0.349299751	0.446854946
LOC101105780	38.55041769	0.225546408	0.241056528	0.93565775	0.349449431	0.44696087
ZNF579	19.47705997	-0.281657412	0.301015205	-0.935691646	0.349431973	0.44696087
EPHA2	4.355579889	0.546991927	0.584698005	0.935511875	0.349524567	0.447014195
LOC114110119	5.331585401	-0.493164073	0.527215571	-0.935412572	0.34957572	0.447036843
RPH3AL	1.718571032	0.919466765	0.983231604	0.935147691	0.349712192	0.447168579
DACT2	2.352429605	-0.823170067	0.880623266	-0.934758482	0.349912781	0.447351644
PLAG1	2.001192949	0.905128865	0.96832151	0.934740018	0.349922298	0.447351644
FOXO3	198.6928475	0.137897764	0.147610075	0.934202923	0.350199228	0.447662862
SRR	27.27404413	0.248143334	0.265757014	0.933722616	0.350446996	0.447936745
DCUN1D1	30.9914004	0.231463951	0.247984708	0.933379937	0.350623836	0.448119926
RABIF	28.41762399	-0.291941864	0.312936278	-0.932911534	0.350865646	0.448386101
LRP6	70.75934397	0.18822549	0.201884376	0.932343025	0.351159279	0.448718444
AKT1S1	28.637921	0.239931118	0.257439928	0.931988755	0.351342337	0.448850681
LOC132659450	3.040834005	-0.679276263	0.728878026	-0.931947786	0.35136351	0.448850681
SYT5	2.906639808	-0.728335616	0.781489374	-0.93198403	0.351344779	0.448850681
LOC121817260	6.386675098	-0.466471427	0.500600316	-0.931824077	0.35142745	0.448889458
LRIG1	23.82001914	0.26594256	0.285431525	0.93172105	0.351480705	0.448914582
CD40	2.612138466	0.734111866	0.788304754	0.931253887	0.351722249	0.449180163
DTD2	7.421414513	0.422344363	0.45355667	0.931183226	0.351758793	0.449183914
RCCD1	2.511885331	-0.768605742	0.825517754	-0.931059009	0.351823041	0.449223038
GOLGA3	44.932243	0.210697195	0.226345085	0.930867113	0.351922308	0.449266177
MED9	10.82028637	0.368804017	0.39619549	0.930863743	0.351924051	0.449266177

PCLAF	47.64005386	-0.248848778	0.267413909	-0.930575299	0.352073297	0.449413779
LOC114109996	13.77665857	-0.342179427	0.367833845	-0.930255418	0.352238855	0.449552862
PAPSS2	8.3487924	0.44072741	0.473780819	0.930234808	0.352249523	0.449552862
MIX23	16.14322942	-0.325556289	0.350053861	-0.930017706	0.352361917	0.449653369
NPM3	76.29601368	-0.216278137	0.23263048	-0.929706794	0.352522916	0.449815875
BTG1	306.3286946	-0.148963538	0.160254976	-0.929540796	0.352608893	0.449882634
GNA13	21.8365776	0.316737235	0.340770481	0.929473802	0.352643596	0.449883965
DDX23	123.4513015	0.166407348	0.179096994	0.929146517	0.35281316	0.45005733
LOC105605916	103.2047325	-0.174770822	0.188200037	-0.928643932	0.353073647	0.450346632
PLA2G7	4.09982791	0.606919937	0.653771671	0.928336244	0.353233179	0.450464142
TPH2	4.880917219	0.568171172	0.612022278	0.928350474	0.3532258	0.450464142
PSMB8	43.75040184	0.261330956	0.281621311	0.927951635	0.353432659	0.450675531
CCDC167	16.18407299	0.292517567	0.3154537	0.9272916	0.353775154	0.451069228
DDB2	2.192934358	-0.782176303	0.843604931	-0.92718318	0.353831434	0.451097954
LOC121820063	2.314578068	0.78017361	0.841574977	0.927039932	0.353905802	0.451149732
ZNF691	7.928741256	-0.434551247	0.468902731	-0.926740704	0.354061178	0.451304759
RPS6KA2	2.714639951	0.758212806	0.818346541	0.926518006	0.354176844	0.451409144
LOC105605761	4.984583959	0.569571427	0.615600782	0.925228564	0.354847029	0.452220193
C2H2orf72	16.85572172	0.376381699	0.407222189	0.924266183	0.355347745	0.452771969
PKNOX1	31.29021568	0.224026953	0.242381795	0.924273015	0.355344189	0.452771969
NEDD1	11.39963182	0.353091134	0.382248204	0.923722153	0.355630995	0.453089684
CNDP2	32.77922012	-0.222097773	0.240684133	-0.92277696	0.35612345	0.45367385
CAMSAP3	7.904250764	-0.463764241	0.50272057	-0.922508982	0.356263147	0.453765316
PRDX4	29.88046729	-0.262521019	0.284571774	-0.922512502	0.356261312	0.453765316
PSMD9	7.709839418	-0.427838373	0.464123495	-0.921820114	0.356622414	0.454179627
LOC114116860	1.659870534	0.938631749	1.01859197	0.921499262	0.356789826	0.454349544
ACOX3	4.633173724	0.578793439	0.628236692	0.921298368	0.356894673	0.454439763
PTAR1	30.076884	0.249464737	0.270804243	0.921199512	0.356946273	0.454462172
LOC105613604	6.478164407	-0.485738429	0.527436362	-0.920942249	0.357080579	0.454589868
MTSS2	2.3978367	0.794419522	0.862725587	0.92082527	0.35714166	0.454598783
TANGO2	33.60908705	-0.292028853	0.317147381	-0.920798563	0.357155605	0.454598783
PPP4R4	2.782854237	-0.681306805	0.740162321	-0.920482962	0.357320433	0.454765274
LOC105601979	31.15094735	-0.270929703	0.294382268	-0.920332958	0.357398791	0.454821693
LOC101109652	20.91403233	-0.318228286	0.345873618	-0.920071001	0.357535657	0.454952551
ARHGAP10	40.74911378	0.208986942	0.22727923	0.919516234	0.357825618	0.455278174
DMAC1	30.90338404	0.250931258	0.273095788	0.918839723	0.358179412	0.455661053
NUP155	27.24244179	-0.238394918	0.25946039	-0.918810452	0.358194724	0.455661053
FAM76A	9.75774775	0.396829274	0.431969593	0.918650944	0.358278176	0.45572384
PAF1	23.45778786	-0.259438952	0.282443079	-0.918553049	0.358329399	0.455745623
MTFR2	4.583659264	-0.531073763	0.578405148	-0.918169149	0.358530317	0.455957775
SLC38A2	111.5993259	0.157952083	0.172147561	0.917538899	0.358860319	0.456334033
LOC105605808	20.92980163	-0.259304682	0.282666978	-0.917350459	0.358959024	0.456416126

PCED1A	28.03088855	-0.242799378	0.264722152	-0.917185721	0.359045328	0.456482436
SLC35A5	26.1743224	0.254602129	0.277705951	0.916804729	0.359244974	0.456692821
MBD2	38.92157543	0.210687449	0.229897361	0.916441357	0.359435452	0.456891513
PPAN	6.394439563	-0.472915158	0.516217144	-0.916116722	0.359605678	0.457064425
LOC101102527	47.56936051	-0.240708199	0.263021468	-0.915165599	0.360104701	0.457655172
BTAF1	57.17119044	0.18645403	0.203824106	0.914779089	0.360307615	0.457869517
ASDURF	20.43491525	0.274508365	0.300275569	0.914188145	0.360617992	0.458220371
ZNF26	2.824710491	0.68053561	0.745043956	0.913416725	0.361023412	0.458691914
EIF4A2	41.64145353	0.319945312	0.350416636	0.913042589	0.361220143	0.458898245
CDR2	3.876857999	0.669233727	0.733489343	0.912397342	0.361559588	0.459285826
DYRK1A	117.7256496	-0.142597408	0.156336888	-0.9121162	0.361707551	0.459386463
LOC132657583	2.406110237	-0.740525399	0.811826504	-0.912171992	0.361678185	0.459386463
MAPK12	6.602087693	0.512198651	0.561765846	0.911765381	0.361892238	0.459577354
RNMT	14.1821265	0.322401195	0.353936234	0.91090192	0.362347054	0.460111222
LOC132657344	2.438263485	-0.820881094	0.901248723	-0.91082636	0.362386871	0.460118069
UBA5	21.70912957	-0.289974592	0.318397109	-0.910732491	0.362436341	0.46013717
LOC101110440	1.438927371	-0.96194339	1.056728611	-0.910303157	0.362662654	0.460380761
ARAP1	12.17067511	-0.365229919	0.401488705	-0.909689152	0.362986467	0.460529389
CDCA7L	24.23789292	0.299895353	0.329633232	0.90978495	0.362935933	0.460529389
DHRS7B	36.53589142	0.275684402	0.303010808	0.909817056	0.362918998	0.460529389
DLGAP4	9.379215871	-0.381603189	0.419367468	-0.909949431	0.362849179	0.460529389
ECH1	12.77097723	0.328629208	0.361238422	0.909729386	0.362965243	0.460529389
MYZAP	19.99649862	0.290540264	0.319335735	0.909826969	0.362913769	0.460529389
VRK1	91.4680647	0.241822501	0.265917487	0.909389239	0.3631447	0.460686414
LMF2	23.95448892	-0.32241367	0.354597801	-0.909237646	0.363224696	0.460715862
TIGD7	3.465590463	-0.69208567	0.761190657	-0.909214614	0.363236852	0.460715862
IRF2BPL	82.25821602	0.208565253	0.22941057	0.909135324	0.363278698	0.460725219
KDM4C	32.99302567	-0.222754361	0.24508148	-0.908899199	0.363403337	0.460839563
G6PD	5.893683591	0.486014097	0.534848449	0.908694972	0.363511159	0.460932563
CCNC	37.48217161	-0.257706304	0.283654392	-0.908522172	0.363602405	0.461004529
LOC105602268	2.490366982	0.781325184	0.860243426	0.90826057	0.363740569	0.461135962
LOC114110832	12.77760471	0.341428669	0.375962269	0.908146104	0.363801035	0.461168876
CDK19	98.97233962	0.216804443	0.238781232	0.907962664	0.363897948	0.461204245
ZNF827	116.514116	-0.166434525	0.183297979	-0.907999784	0.363878336	0.461204245
HSP90AB1	3340.530378	-0.150003446	0.165442624	-0.906679565	0.364576273	0.462020145
DNAAF2	16.75646639	0.305951737	0.33795967	0.905290673	0.365311416	0.462864005
DXO	7.151500675	0.416275363	0.459823402	0.905293992	0.365309658	0.462864005
NGRN	9.018615057	-0.381455088	0.42147903	-0.905039302	0.365444566	0.462988822
AGR3	17.09560753	0.329828045	0.364502342	0.904872225	0.365533083	0.463042607
STIL	23.46651256	0.26352025	0.291237821	0.904828394	0.365556306	0.463042607
CTNND1	50.02187708	-0.217645714	0.240702754	-0.904209488	0.36588433	0.463279926
FGFR3	37.94007977	0.272912688	0.301809469	0.904254891	0.36586026	0.463279926

PKMYT1	4.703368187	-0.535797955	0.592599881	-0.904147929	0.365916967	0.463279926
PPP1R11	8.420547771	0.425934745	0.471085241	0.90415642	0.365912465	0.463279926
TPPP2	5.104036398	-0.579091796	0.640440519	-0.904208553	0.365884826	0.463279926
ZBTB39	4.082248589	-0.588349891	0.65083488	-0.903992561	0.365999346	0.463340335
TBCC	22.34788822	-0.249239747	0.275857822	-0.90350799	0.36625635	0.463621779
PIGP	3.601725949	0.587018312	0.649963822	0.903155363	0.366443444	0.463814685
CCNYL1	42.48795086	-0.212352141	0.235146806	-0.903061984	0.366492999	0.463833484
SRSF10	64.83683032	-0.160768306	0.178084409	-0.902764635	0.366650824	0.463989294
AGBL5	11.80447179	-0.358109447	0.397046789	-0.901932609	0.367092668	0.464504459
PPWD1	19.64507947	0.264775618	0.293675615	0.90159211	0.367273584	0.46468939
GADD45GIP1	14.19900175	-0.318158864	0.352943648	-0.901443802	0.367352401	0.464701135
LOC121817399	2.073288199	-0.858773215	0.952638524	-0.901468074	0.367339501	0.464701135
LOC114114100	11.35722211	-0.337129726	0.374054226	-0.901285704	0.367436433	0.464763449
WDR18	4.921568805	-0.493419262	0.547943527	-0.900492911	0.367857996	0.465252647
PAFAH1B2	51.24338308	-0.234502706	0.260550646	-0.900027342	0.3681057	0.465521884
ABLIM2	1.522066964	-0.934956584	1.038938679	-0.899915079	0.368165445	0.46555339
FDX2	27.79061254	0.250355106	0.278438933	0.899138289	0.368579006	0.465988175
GSTA1-1	16.22909865	0.338397418	0.376339801	0.899180521	0.368556514	0.465988175
ZNF236	28.40685458	0.219030858	0.243718195	0.898705401	0.368809599	0.46623561
MPPE1	26.5799729	0.27823006	0.309618821	0.898621274	0.368854423	0.466248176
BCDIN3D	35.84887555	-0.249260766	0.277471603	-0.898328923	0.369010217	0.466400999
SERTAD2	24.28572279	-0.239441658	0.266599656	-0.898131908	0.36911523	0.466489615
SLC25A32	10.08942579	0.365441558	0.407169727	0.897516524	0.36944336	0.466860164
RAB43	25.43673223	0.290540633	0.323777832	0.897345664	0.369534497	0.466931186
CHRM3	9.993450719	-0.357425299	0.398417072	-0.897113411	0.369658403	0.467043597
EMP1	158.2106894	0.169636534	0.189198866	0.896604391	0.369930054	0.467342638
SUMO1	16.61319132	-0.313361372	0.349550525	-0.896469463	0.370002082	0.467389456
SMIM15	19.02218334	0.279775504	0.312206078	0.896124463	0.370186293	0.467577962
RHOF	26.85787619	0.294167	0.328386375	0.895795385	0.370362055	0.467755762
AK2	64.83284171	0.196091399	0.218983333	0.895462662	0.370539816	0.467936053
CMBL	40.22631885	0.20606518	0.230387148	0.894430017	0.371091858	0.468574854
ZNF165	2.076875734	-0.805803115	0.900957462	-0.894385305	0.371115772	0.468574854
CAPZA2	115.3423215	0.169364332	0.189397828	0.894225315	0.37120135	0.468638637
DNASE1	29.32529351	-0.239437757	0.268020694	-0.893355486	0.371666833	0.469181988
RPL26L1	38.71073818	-0.199795687	0.223700902	-0.893137603	0.371783488	0.469284928
CYTH1	13.84720951	0.329457534	0.369013622	0.892805887	0.371961133	0.469420499
LOC114116810	3.391298071	-0.627642232	0.702989714	-0.892818514	0.37195437	0.469420499
TIMM9	14.23819548	0.327618711	0.367107406	0.892432859	0.372160965	0.469628348
PJA2	32.65038507	0.232458478	0.260512051	0.892313723	0.372224801	0.46966456
PTPN12	33.84101334	-0.212580485	0.238266594	-0.892195931	0.372287923	0.469699866
LOC101103089	124.791727	-0.127544918	0.143051612	-0.891600708	0.37260699	0.470058049
SLF2	57.24879726	-0.167357683	0.187763289	-0.891322708	0.37275607	0.470157368

SMURF1	8.781373544	0.389680647	0.437177804	0.89135506	0.372738719	0.470157368
ULK4	4.627201571	-0.53764823	0.60328598	-0.891199608	0.372822094	0.470196274
SMAP1	87.68490038	-0.152707253	0.171371018	-0.891091472	0.3728801	0.47022506
LOC105608011	3.213668933	-0.645758729	0.724964989	-0.890744711	0.373066143	0.470415289
SPATA18	2.393349911	-0.713860566	0.80157801	-0.890569049	0.37316041	0.470489769
STRN	52.0072875	-0.192122165	0.215771265	-0.89039736	0.373252559	0.470561565
LOC101112330	1.900520704	-0.897220101	1.008209715	-0.889914159	0.37351198	0.470844207
ERICH3	2.644659955	-0.677610543	0.76189262	-0.889378011	0.373799958	0.471037996
LOC101104287	5.57281537	-0.530833449	0.5968572	-0.889380994	0.373798356	0.471037996
SMC6	178.8019844	0.149190057	0.167714125	0.889549742	0.373707703	0.471037996
STRADA	6.768077788	-0.424038183	0.476787304	-0.889365508	0.373806676	0.471037996
ITPRIPL1	16.78010629	0.275943518	0.310663762	0.888238513	0.374412466	0.471756884
ZC3H18	76.96426309	-0.165978217	0.186917724	-0.887974738	0.37455434	0.471891159
CBX8	2.394936887	-0.824935524	0.929109211	-0.887877888	0.37460644	0.471912316
TSPAN13	7.817542109	0.453577954	0.510964207	0.887690269	0.374707382	0.471994992
RFFL	30.35117015	0.239566597	0.270031189	0.887181212	0.374981346	0.472295577
SPAST	29.52857899	0.230889795	0.260360568	0.886807846	0.375182363	0.472504236
LOC105610484	2.606812689	-0.698915604	0.788245346	-0.886672667	0.375255158	0.472551389
MBLAC1	2.703177166	0.697013702	0.786262372	0.886489964	0.37535356	0.472630775
GPRC5B	10.44566068	-0.416477895	0.470544009	-0.885098708	0.376103396	0.473530331
SMKR1	1.109280011	1.302362539	1.473229198	0.884018957	0.37668598	0.474219158
LARP1	291.2683087	-0.131058388	0.148275925	-0.883881776	0.376760036	0.474267719
LOC101112526	3.005803917	-0.634367906	0.718065839	-0.883439751	0.376998722	0.474523487
OGFOD1	13.85866876	-0.313355437	0.354828896	-0.88311702	0.377173049	0.474698209
CHSY1	4.879049341	-0.527739087	0.597764176	-0.882854992	0.377314624	0.474812158
SEC22A	3.71537041	-0.585278794	0.66296657	-0.882817958	0.377334636	0.474812158
LOC105607217	12.52196041	0.369567994	0.418693954	0.882668569	0.377415369	0.474869041
LOC101103203	13.82109957	0.302748819	0.343236327	0.882041891	0.377754154	0.475228776
MPP7	16.00345037	-0.302971288	0.343501679	-0.882008172	0.377772388	0.475228776
RBSN	9.337319081	0.380175373	0.431302271	0.881459244	0.378069304	0.475557531
LOC114114502	2.503259208	-0.753745337	0.855200701	-0.881366603	0.378119428	0.475575823
LOC132659291	8.24045876	-0.391113184	0.444029707	-0.880826615	0.378411672	0.475898608
CPT1C	2.55854525	-0.731189562	0.830279248	-0.880654989	0.378504586	0.475925898
LOC105604230	8.449546017	0.376862308	0.427910915	0.880702722	0.378478743	0.475925898
MTMR1	5.138848126	0.493257638	0.560212822	0.880482593	0.378597931	0.47599849
NDUFS1	29.23598498	0.216752738	0.24625348	0.880201727	0.378750038	0.476144941
SELENOF	29.85787	0.299233289	0.339997679	0.880103916	0.378803018	0.476166758
CDH1	135.4626519	-0.208930544	0.23770021	-0.878966591	0.379419391	0.476896707
DNAJC24	6.672754661	0.454789274	0.517564311	0.878710655	0.37955818	0.477026294
CIPC	21.66903945	0.273852289	0.311864378	0.878113399	0.379882182	0.477388609
KAZALD1	2.720937434	0.651081765	0.741615694	0.877923391	0.379985294	0.477473295
GRK2	14.6981656	0.308623772	0.351723886	0.877460373	0.380236634	0.477744204

FAM149B1	2.782618724	0.666319657	0.759534564	0.877273647	0.380338022	0.477826676
CAMTA1	56.06310753	-0.204821419	0.233723687	-0.876340012	0.380845219	0.47841891
LYG2	1.756737338	0.85718103	0.978887165	0.875668882	0.381210067	0.478832229
HDHD3	5.68595858	-0.482615515	0.551301447	-0.875411297	0.381350155	0.478963181
USB1	8.93631959	0.387315218	0.442524799	0.875239576	0.381443564	0.479035485
ARSG	3.852704063	0.572451529	0.654281273	0.874931856	0.381610985	0.479141227
BTBD9	15.68292555	0.291784609	0.333511135	0.874887159	0.381635306	0.479141227
KCNN4	93.49820404	-0.196970988	0.225116727	-0.874972689	0.381588766	0.479141227
REEP5	73.56729077	0.215091233	0.245911587	0.874668963	0.381754052	0.479245296
LOC121817381	11.82471977	0.370595868	0.424038882	0.873966711	0.382136382	0.479635166
STX18	13.09501748	0.315198773	0.360638594	0.87400178	0.382117284	0.479635166
NCAPD2	69.44320509	0.199128736	0.227863449	0.87389503	0.382175421	0.479639125
LOC101108663	131.8586596	-0.15640592	0.17901306	-0.873712343	0.382274928	0.479718964
DNAAF4	10.03907803	0.376971781	0.431688249	0.87325004	0.382526805	0.479989981
SLC38A7	5.412920505	0.527269207	0.603997619	0.872965704	0.382681772	0.480139356
SPACDR	13.33056491	-0.310895558	0.356186778	-0.872844185	0.382748012	0.480177392
ARFGEF3	75.88032253	0.237864669	0.272775618	0.872015875	0.383199717	0.48069896
PCYT1A	37.98789964	0.213396319	0.244858867	0.871507417	0.383477158	0.481001848
APBB2	19.35911532	-0.255301552	0.292991937	-0.871360334	0.383557437	0.481057399
LOC101116026	8.052656568	0.384816499	0.441684458	0.871247542	0.383619006	0.481089476
ZNF212	18.84717371	0.258912977	0.29720378	0.871163137	0.383665085	0.481102122
TSNAXIP1	1.616405585	-0.893663327	1.026420195	-0.870660312	0.383939655	0.481401259
PPARGC1A	16.48267414	-0.318137922	0.365474494	-0.870479136	0.384038616	0.481480175
AVEN	46.89069144	0.190103749	0.218520108	0.86995998	0.384322275	0.481790614
PIGT	82.27924572	-0.162602486	0.187062026	-0.86924369	0.384713855	0.482236274
LOC114116599	10.59705158	-0.336245749	0.386884141	-0.869112258	0.384785733	0.482281142
SYNE4	2.117931164	0.741157364	0.852905898	0.868979059	0.384858585	0.482327224
ABCG8	4.965461396	0.551261266	0.634529093	0.868772246	0.384971716	0.482423773
DDX55	24.81957244	0.276227404	0.318207691	0.868072681	0.385354545	0.482858241
FNIP2	6.459003152	0.41650345	0.480013028	0.867691971	0.385562982	0.48307413
WEE1	10.75197785	-0.374251265	0.431583777	-0.867157863	0.38585552	0.48339534
LOC101105290	9.44718608	0.378709041	0.436823868	0.866960505	0.38596365	0.483485487
AGPAT1	29.84153417	0.235884695	0.27252153	0.865563521	0.386729568	0.484359133
SLC30A6	14.00848216	-0.324249833	0.374614395	-0.865556257	0.386733553	0.484359133
CDX2	59.76969088	0.230350991	0.266169249	0.865430518	0.386802537	0.484400141
LOC101121661	4.122714624	0.56777083	0.656134729	0.865326593	0.386859559	0.484426163
LOC121818785	5.969452674	-0.476041001	0.55055401	-0.864658131	0.387226456	0.48484017
CASP3	35.45112281	-0.211390204	0.244632893	-0.864111938	0.387526402	0.485124837
CNN3	59.61061498	-0.202647516	0.234503905	-0.864154124	0.38750323	0.485124837
GSR	2.699995471	-0.633323663	0.733100123	-0.863897909	0.387643976	0.485226576
PRSS53	4.078483631	-0.569623702	0.659628278	-0.863552581	0.387833723	0.485418629
NEMP1	1.698335544	0.858282605	0.994700423	0.862855374	0.388216989	0.485852835

CLPP	21.96481402	-0.260046891	0.30145384	-0.862642488	0.388334062	0.48595385
MRPL34	64.65820493	0.176954817	0.205276179	0.862032884	0.388669422	0.486327982
TMEM39A	41.0708621	0.207019265	0.240180872	0.861930691	0.388725659	0.486352818
FAH	3.578428654	-0.599942664	0.696177838	-0.861766392	0.388816082	0.486420419
DLL1	11.23639199	0.34059121	0.39536196	0.86146682	0.388980988	0.486581178
TXNL4B	5.47775659	0.47608173	0.552748243	0.861299399	0.389073166	0.48665094
VKORC1	5.664002935	-0.445699028	0.517722863	-0.860883419	0.389302255	0.486891919
ALDH5A1	130.2693754	-0.159682175	0.185534643	-0.860659618	0.38942554	0.48700054
C1QTNF1	2.706513919	-0.650963357	0.756830915	-0.860117292	0.38972439	0.487284681
QPCTL	15.33228143	0.291243436	0.338609887	0.860114979	0.389725665	0.487284681
LMBR1	13.89521675	-0.310826423	0.361513112	-0.859792945	0.389903189	0.487461044
RBM5	87.65402315	0.170519223	0.198477549	0.859136076	0.390265446	0.487868308
HNRNPU	497.4757413	0.096640396	0.112546392	0.858671647	0.390521697	0.488142991
MAD2L2	10.38688818	-0.349037931	0.406551936	-0.858532208	0.390598654	0.488193529
TBP	5.210207166	0.475158164	0.553676721	0.858187	0.390789213	0.488386033
AP2A2	48.81760605	-0.194049543	0.226242752	-0.857705018	0.391055368	0.488642804
ATRAID	17.4716872	-0.292461237	0.340990083	-0.857682529	0.39106779	0.488642804
E2F6	11.58125768	0.315869504	0.368481288	0.857219932	0.391323349	0.488916422
CNNM3	18.17134716	-0.265446908	0.309934456	-0.856461432	0.391742597	0.489394481
TCF19	2.270163792	-0.713045052	0.832617687	-0.856389509	0.391782366	0.489398421
VPS54	14.38857165	-0.30452041	0.355806712	-0.855859094	0.392075724	0.489719103
ERI2	15.20375924	0.313617278	0.3667551	0.855113611	0.392488255	0.490077318
LOC132657394	9.144986563	0.353772024	0.413699315	0.855142881	0.392472053	0.490077318
NUP205	16.07043367	-0.287842331	0.336627849	-0.855075812	0.392509179	0.490077318
RANGAP1	2.799707494	0.732305866	0.856333346	0.855164485	0.392460094	0.490077318
MTG2	4.870974122	-0.52024947	0.60855545	-0.854892468	0.392610681	0.490158263
RNF144B	16.73217177	0.280567454	0.32830301	0.854599093	0.39277313	0.490315275
DDX20	16.83679697	-0.276104459	0.323307239	-0.854000239	0.393104857	0.49064473
MID1IP1	32.92366114	0.243611547	0.285262722	0.853990126	0.393110461	0.49064473
ZBTB3	2.544416658	0.666348024	0.780538255	0.853703223	0.393269448	0.490797333
LOC101112013	19.13566239	-0.245165566	0.287326887	-0.853263571	0.393513157	0.49105563
LRRC27	3.513706259	-0.588008093	0.689242543	-0.853122169	0.393591558	0.491107615
NARS2	6.711359467	0.420993537	0.493679152	0.852767502	0.393788249	0.491307172
HSH2D	3.135212112	0.707905576	0.830573644	0.852309222	0.394042489	0.491578486
RGS7	2.385940264	-0.794320239	0.932264807	-0.852032848	0.394195861	0.491723927
CHIC1	3.734027451	0.528608901	0.620543801	0.851847846	0.394298547	0.491806119
JOSD2	6.9773814	-0.436770233	0.513218552	-0.851041395	0.394746358	0.492318729
FNDC3B	328.1551611	-0.183278604	0.215422616	-0.850786272	0.394888089	0.492449542
ECI2	32.14676267	-0.499818079	0.587549195	-0.850682944	0.3949455	0.492475189
DNAJA1	15.95459658	0.28558763	0.335864382	0.85030639	0.395154765	0.492658584
MKS1	9.44755288	-0.372779055	0.43841626	-0.850285649	0.395166293	0.492658584
REPS2	3.862669821	0.527998494	0.621035268	0.850190837	0.395218995	0.492678334

TLCD1	1.991078269	-0.899804449	1.058554267	-0.850031478	0.395307585	0.492696866
USPL1	15.64215505	0.293053696	0.344744813	0.850059767	0.395291858	0.492696866
IGHMBP2	2.201714657	0.701404839	0.825437807	0.849736749	0.395471462	0.492855157
SAP30BP	23.26330707	-0.246625505	0.290494965	-0.848983751	0.395890334	0.493331177
SIRT6	2.562271456	-0.658661036	0.776055612	-0.84872917	0.396032011	0.493461719
PPP1CB	89.69269937	0.242860841	0.286171803	0.848653987	0.396073857	0.493467857
DROSHA	19.6644742	0.294540374	0.347107672	0.84855622	0.396128277	0.493489659
ITGA6	122.748301	-0.147540731	0.173927846	-0.848286999	0.396278156	0.493630367
BCKDHB	3.186854953	0.592879574	0.699113547	0.84804475	0.396413049	0.493660376
BCL11A	52.91820703	-0.176852485	0.20852289	-0.848120245	0.396371008	0.493660376
SLC39A6	93.57104604	-0.143860487	0.169627383	-0.848097071	0.396383913	0.493660376
MAP3K11	6.070422261	0.488415518	0.576060921	0.847853934	0.396519322	0.493746717
TMEM116	21.89537934	0.242343747	0.285945604	0.847516956	0.39670704	0.493934448
SLC35B3	8.612843527	-0.389893627	0.460216072	-0.847196894	0.396885385	0.494110474
BIRC6	62.5907574	0.166137987	0.196145545	0.84701382	0.396987419	0.49418204
ITGAV	124.0501798	-0.149070913	0.176020564	-0.84689487	0.397053722	0.49418204
TEX264	6.109982752	0.454562442	0.53674018	0.846894753	0.397053787	0.49418204
BACE1	16.24943367	0.294801214	0.348388267	0.846185828	0.397449087	0.494581929
C23H18orf21	21.47025219	-0.257039733	0.303760334	-0.846192553	0.397445336	0.494581929
LOC132659121	2.103013683	0.766353403	0.905729252	0.846117537	0.397487179	0.494583283
CUTC	20.80781987	0.239083608	0.282654231	0.845851867	0.397635387	0.494713469
LRRC57	7.887463969	-0.363433986	0.429693954	-0.8457973	0.397665832	0.494713469
SPHK2	51.80917571	0.186360945	0.220433222	0.845430388	0.397870584	0.494922124
LOC106991840	22.80050415	-0.246896476	0.29216023	-0.845072158	0.398070552	0.495124791
HECTD1	371.2911966	-0.126741334	0.150013003	-0.844868989	0.39818399	0.495173945
LOC105601854	4.692499768	-0.535185427	0.633453983	-0.844868674	0.398184166	0.495173945
KLHL22	5.293135624	0.493093218	0.583714582	0.844750556	0.398250126	0.495209897
ATP5F1B	167.3282896	-0.168891763	0.199967086	-0.844597811	0.398335432	0.49526385
NDST2	28.65179535	-0.266178082	0.315175156	-0.844540176	0.398367623	0.49526385
ESRRA	131.2143449	-0.138984389	0.164640542	-0.84416868	0.398575154	0.495453098
SCAI	8.275700467	0.372189809	0.44091267	0.844134981	0.398593982	0.495453098
ZNF526	5.973242425	0.447473647	0.53022556	0.843930736	0.398708112	0.495548877
LOC132660174	2.318032577	-0.711009593	0.842578447	-0.843849727	0.398753384	0.495559063
NDEL1	31.91622323	0.203792882	0.241694296	0.843184494	0.399125269	0.495975116
NME6	5.706113498	-0.49929696	0.59243954	-0.84278129	0.399350774	0.496209207
LOC132659276	1.814605928	0.775265453	0.92052631	0.842198039	0.399677111	0.496568532
CALM3	87.10551021	-0.159527063	0.189460193	-0.842008342	0.399783284	0.496654278
LOC121817267	2.753184212	0.677659609	0.805855079	0.840919945	0.400392784	0.497365237
PLK3	11.18934988	-0.318690967	0.379083533	-0.840687974	0.400522759	0.497480457
TM2D3	17.83683467	-0.274163235	0.326212862	-0.840442749	0.400660189	0.497604914
C2H9orf43	2.463300762	-0.669760792	0.797551064	-0.839771674	0.401036418	0.497978517
GCLM	13.44011349	0.312129969	0.371713023	0.839706842	0.401072777	0.497978517

SCML4	10.57228696	-0.332765452	0.396274611	-0.839734475	0.401057279	0.497978517
STX12	17.01637863	0.302825056	0.360782124	0.839357152	0.401268921	0.498175775
LOC132659699	16.52510632	-0.311734208	0.371551216	-0.83900737	0.401465174	0.498373132
LDHAL6B	3.24100908	-0.586844115	0.700255068	-0.838043367	0.402006348	0.498998593
LGALSL	1.585856114	-0.824363741	0.98409096	-0.837690594	0.402204497	0.49919819
MARCHF5	29.27870304	-0.22237774	0.265515985	-0.837530518	0.40229443	0.499261472
SGO1	17.63116471	-0.290801966	0.34724029	-0.837466086	0.402330633	0.499261472
TTC23	7.683830674	-0.433859814	0.518103186	-0.837400397	0.402367543	0.499261472
LOC105609820	5.873595887	0.422670463	0.504842721	0.837231963	0.402462194	0.499332562
DET1	2.074699041	-0.758698725	0.906780452	-0.836695061	0.402763996	0.499660625
ST6GAL2	1.898536264	-0.748696113	0.895020096	-0.836513187	0.402866261	0.499741109
VPS9D1	1.509145397	0.911352181	1.089570275	0.836432676	0.402911536	0.49975089
LOC105607754	19.01480554	0.255452563	0.305483192	0.836224609	0.403028557	0.499771051
NR2C2	57.08960897	-0.177257215	0.211978351	-0.836204328	0.403039964	0.499771051
PCGF1	2.583909598	0.728116737	0.870695676	0.836247104	0.403015904	0.499771051
DYNC1LI1	30.39262112	-0.226241587	0.270601756	-0.836068436	0.403116404	0.499819467
TCIRG1	12.54813816	-0.340438695	0.407593648	-0.835240433	0.403582349	0.500350772
EIF4E	118.1708291	0.134297117	0.160873476	0.834799622	0.403830539	0.500612037
TARBP1	31.57770096	-0.216319605	0.259283358	-0.83429807	0.404113039	0.500915782
C25H1orf198	6.930352484	0.393429059	0.471633616	0.834183666	0.404177494	0.50094922
RHOBTB1	17.96219104	-0.260647241	0.312557748	-0.833917069	0.404327718	0.501088945
LOC114114095	3.073668939	-0.632158942	0.759256895	-0.832602175	0.405069132	0.501961248
SPA17	8.902157328	0.363261773	0.436434504	0.832339721	0.405217216	0.502098202
CREB3L1	36.85492984	0.209819192	0.252197253	0.831964619	0.405428916	0.50231395
OGFR	34.45994597	-0.233557686	0.281049998	-0.83101828	0.405963302	0.502929419
TIGD6	5.412280716	0.455909255	0.548869645	0.830633028	0.40618097	0.503152442
RAPGEF1	26.08392008	0.230064974	0.277134774	0.830155562	0.406450836	0.503440078
ETFDH	4.415510648	0.527578364	0.635662522	0.829966131	0.406557933	0.503526069
ARFIP1	21.94206329	0.25158574	0.303246029	0.829642325	0.406741039	0.503659508
FBXO42	10.05796585	-0.359111753	0.432837797	-0.829668193	0.40672641	0.503659508
HAPSTR1	10.76734282	0.3329111	0.40153882	0.829088206	0.407054497	0.504000964
ACAP3	2.544679176	0.633139867	0.763932003	0.828790867	0.407222757	0.504143343
SUMF1	42.59174516	0.184398641	0.222501675	0.828751698	0.407244925	0.504143343
LOC101113672	7.320525087	0.484546756	0.584888791	0.82844254	0.407419923	0.50431327
PLIN3	5.155599113	-0.467883964	0.565171276	-0.82786225	0.407748516	0.504673272
EGLN1	25.12667132	-0.269489068	0.325550375	-0.827795295	0.40778644	0.504673477
CDC42SE1	62.09082507	-0.162553301	0.196610703	-0.826777479	0.408363197	0.505311808
UBXN2A	44.02143441	-0.240612673	0.291033796	-0.826751657	0.408377836	0.505311808
MKRN1	6.38882814	0.426638989	0.516419992	0.826147314	0.408720531	0.505689032
LOC101108756	1.360991989	1.006349503	1.218763993	0.825713189	0.408966809	0.505946906
PTPRA	74.12644649	0.16512484	0.200007229	0.825594361	0.409034235	0.505983488
CENPB	52.01871686	-0.188674691	0.228642872	-0.825193849	0.409261545	0.506217824

RABGGTA	4.332021166	0.498851494	0.604728515	0.824918094	0.409418093	0.5063646
NAXE	28.06455893	0.247801901	0.300447144	0.824777023	0.409498194	0.506416808
ILVBL	13.3588273	0.339308845	0.411786215	0.823992724	0.409943692	0.506920843
C15H11orf71	1.832337383	-0.830783869	1.008330742	-0.823920004	0.409985013	0.50692504
LOC105609542	1.575806819	0.887861104	1.077810676	0.823763508	0.410073946	0.506988101
SPNS1	3.583816995	-0.575324829	0.698821692	-0.823278434	0.410349674	0.507282071
APC	84.9669704	-0.135245163	0.164315078	-0.823084313	0.410460049	0.507334877
TMEM43	8.024570243	0.447235056	0.543374401	0.823069795	0.410468304	0.507334877
MIP	2.156941436	-0.712261667	0.86618799	-0.822294555	0.410909275	0.507832951
ARHGEF10L	27.7854301	-0.267336601	0.325299037	-0.82181799	0.411180493	0.508121161
MOB2	2.498806782	0.714451406	0.869697738	0.821493922	0.411364985	0.508302153
VASP	161.2130791	0.154832246	0.188502817	0.82137895	0.41143045	0.50833605
OSBPL9	61.81245229	0.156816199	0.190936147	0.821301791	0.411474388	0.508343347
ITGA3	9.904220365	0.36588604	0.445573931	0.821156747	0.41155699	0.508398403
STRN3	241.5592646	-0.104015294	0.126714692	-0.820862152	0.411724791	0.508558688
CCDC68	18.3701752	0.268144197	0.326965516	0.82009932	0.41215949	0.509048581
TOX3	61.25055519	-0.179667661	0.219350959	-0.819087646	0.41273641	0.509714021
FEM1B	12.60155003	-0.320241934	0.391052597	-0.818922919	0.412830393	0.509752352
LOC106991039	2.787634163	0.675692741	0.825122858	0.818899555	0.412843724	0.509752352
ATP5ME	296.5805312	-0.175068735	0.21383797	-0.818698077	0.412958694	0.509847211
MTPAP	18.9100836	0.251527145	0.307677728	0.817501958	0.413641631	0.510572227
NR3C2	24.35759744	0.209689021	0.256497327	0.817509576	0.413637279	0.510572227
NXN	10.65471096	-0.322203914	0.394148236	-0.817468874	0.41366053	0.510572227
LGALS4	64.06602127	0.231131441	0.282850889	0.817149425	0.413843041	0.510750331
KBTBD2	40.27927504	0.247105905	0.302457877	0.816992794	0.413932546	0.510813629
MKKS	6.217376958	-0.432260414	0.529463754	-0.816411721	0.414264695	0.511176322
COG8	24.38068265	-0.229983632	0.28176599	-0.816222114	0.414373111	0.511262901
ALG6	7.870787278	0.375396357	0.460070261	0.815954407	0.414526213	0.511395763
ASB9	3.041534698	0.591602997	0.725211414	0.815766252	0.414633839	0.511395763
HMG20B	38.32256644	0.221267908	0.271205721	0.815867402	0.414575978	0.511395763
KIAA0586	42.92605756	0.20736076	0.254190059	0.815770534	0.414631389	0.511395763
GNG4	4.606154962	0.513230066	0.629207249	0.815677293	0.41468473	0.511411339
ZNF148	130.3491883	-0.121519385	0.148996405	-0.815586021	0.414736947	0.511428549
TSPAN12	6.756121064	0.433164384	0.531388503	0.815155732	0.414983174	0.511684972
RBKS	6.620792089	0.427013782	0.524497608	0.814138664	0.415565518	0.512355753
LOC101116548	49.63448282	0.248836645	0.305877558	0.813517167	0.415921608	0.512747483
LOC105613406	2.306845357	0.696875838	0.856947819	0.813206852	0.416099472	0.512872145
TMEM141	2.640103029	0.608233924	0.74791809	0.813236011	0.416082756	0.512872145
VPS51	4.478350659	-0.502042798	0.617477838	-0.813053954	0.416187125	0.512932882
ARHGEF9	3.963270396	0.558593273	0.687118394	0.812950545	0.416246413	0.512958654
ITGB8	9.930929452	-0.369444261	0.454599167	-0.812681342	0.416400781	0.51310158
LSM11	6.241706158	0.447738282	0.551281264	0.812177578	0.416689744	0.513390789

SGCB	4.278838365	0.479164546	0.590003666	0.812138253	0.416712305	0.513390789
ANXA3	20.92038856	0.248825395	0.306828971	0.810957954	0.417389817	0.514178091
LTC4S	2.583612681	-0.664772562	0.820025238	-0.810673295	0.417553313	0.514332097
LOC121817744	3.287583319	-0.541551242	0.668157085	-0.810514853	0.417644332	0.514396806
ADRA2A	5.489158735	0.496941395	0.613248395	0.810342757	0.417743208	0.51447118
DELE1	20.12792359	0.246134017	0.303788423	0.810215262	0.417816467	0.514513995
HINT2	113.0231633	0.229146231	0.283184483	0.809176506	0.418413627	0.515106985
IL4I1	6.528055158	-0.413426773	0.510912153	-0.809193462	0.418403875	0.515106985
LOC105603888	2.924212139	0.622271589	0.76894025	0.809258702	0.418366355	0.515106985
PPIB	132.0721614	-0.179674486	0.222213279	-0.808567728	0.418763834	0.515490643
TXNDC5	14.15846996	0.308955687	0.382165079	0.80843516	0.418840118	0.515537068
MCUR1	32.75454818	-0.197617431	0.244505485	-0.80823312	0.418956395	0.515632705
NOL4	2.67262672	0.720737207	0.891869772	0.808119335	0.419021888	0.515665828
POLR3G	9.680577041	0.392251725	0.485466104	0.807989934	0.419096377	0.515710014
SNAP25	1.286114007	1.098054893	1.359123501	0.807913992	0.419140096	0.515716333
ZBTB42	8.764179758	0.341272177	0.422473634	0.807795207	0.419208485	0.515753001
VIRMA	42.09822817	-0.206011342	0.255289763	-0.806970635	0.419683401	0.516289769
HYAL1	7.869677667	0.408400953	0.506445371	0.806406724	0.420008371	0.516641992
ARHGEF12	89.93576383	0.164860257	0.204507057	0.806134808	0.420165123	0.516787249
MACC1	175.2480584	-0.149410892	0.185391092	-0.805922713	0.420287414	0.516857088
MSRA	9.338716111	-0.33276915	0.412915054	-0.805902199	0.420299243	0.516857088
FIP1L1	75.45865156	-0.152527114	0.189293846	-0.805769003	0.420376054	0.516903987
FSCN1	8.976724796	0.347406581	0.431235146	0.805608225	0.420468782	0.516970448
C17H4orf46	11.53350365	-0.292606	0.363408981	-0.805169975	0.4207216	0.517233711
DLK2	19.33294123	0.268962263	0.334083239	0.805075596	0.420776057	0.517248677
LOC114108697	5.425834768	-0.480373187	0.596725949	-0.805014744	0.420811171	0.517248677
PUS3	24.09321228	-0.245719402	0.305333058	-0.804758592	0.420959001	0.517382805
COPG1	29.08098403	0.276542892	0.343798842	0.804374125	0.42118094	0.517607985
CFAP418	8.062300059	-0.369969095	0.460092832	-0.804118363	0.42132862	0.517741872
FUBP3	40.45767057	-0.194060325	0.241375245	-0.803977743	0.421409829	0.517794059
ARMCX5	3.01552809	-0.652510836	0.811750278	-0.803831983	0.421494016	0.517849896
ARHGEF38	8.552265066	0.369645503	0.459928111	0.803702784	0.421568646	0.517893982
LOC105603128	6.048445311	0.410403045	0.510858277	0.803359881	0.421766757	0.518089741
LOC106991387	58.95176444	0.166366024	0.207369435	0.802268781	0.422397497	0.518816848
MRPL4	37.87458624	-0.176127594	0.219786794	-0.801356581	0.422925244	0.519417331
DBNL	31.58620322	0.199684143	0.249416546	0.800605037	0.423360334	0.519903916
FBXL8	4.931753445	-0.454955354	0.568494439	-0.800281098	0.423547952	0.520059385
ZNF710	86.36608156	0.148541508	0.185618393	0.800252097	0.423564751	0.520059385
GASK1A	1.860057069	0.711650287	0.889376195	0.800167905	0.423613522	0.520071492
RPP14	2.778002551	0.594624396	0.743438246	0.799830248	0.423809155	0.520263884
KXD1	17.81050374	0.248658143	0.31109262	0.799305822	0.424113104	0.520589195
PEF1	8.116854202	0.368771475	0.46145357	0.799151852	0.424202366	0.520650948

KCNAB2	2.000923382	0.743357106	0.930506633	0.798873516	0.424363757	0.52080121
TNRC18	92.499866	0.143837448	0.180070374	0.798784634	0.424415302	0.520816648
POC1B	51.1509746	0.240641303	0.301316208	0.798633782	0.424502793	0.52087619
F2RL1	5.117841004	0.462507155	0.579349381	0.798321652	0.424683856	0.521050525
TUBGCP4	12.52262096	-0.278601435	0.349396729	-0.797378488	0.425231249	0.521674242
GEMIN2	17.99799603	0.260852924	0.327194008	0.797242361	0.425310288	0.521723322
TAF5L	6.542836469	-0.408476075	0.512428198	-0.797138168	0.425370792	0.521749656
GNMT	5.37946135	-0.419356626	0.526151903	-0.797025772	0.425436064	0.521781834
RAD1	6.526718732	0.418062197	0.52476076	0.796671987	0.425641557	0.521985967
PPTC7	21.98831452	0.260274243	0.326781575	0.796477717	0.425754422	0.522076478
FNTA	1.802599942	-0.767372823	0.963662326	-0.796308834	0.425852552	0.522080589
HGFAC	2.281545839	0.746467112	0.937454443	0.796270281	0.425874956	0.522080589
SOX5	3.289322016	0.552437653	0.693734498	0.79632432	0.425843554	0.522080589
KLF11	36.65908583	-0.249359253	0.314024594	-0.794075554	0.427151448	0.523597419
OTUD6B	44.11677712	-0.168582795	0.21242472	-0.793611947	0.427421375	0.523880247
EFR3A	301.2210369	-0.095640065	0.120610287	-0.792967727	0.427796626	0.524292106
HPS6	1.775441141	-0.730824744	0.922738882	-0.792016851	0.428350851	0.524923212
DTL	12.73813434	0.313527914	0.396052268	0.791632669	0.428574892	0.525149616
C12H1orf159	15.69970111	-0.258720397	0.326955893	-0.79130061	0.428768593	0.525290652
DNAJC25	55.96857936	-0.156279762	0.197491907	-0.79132236	0.428755904	0.525290652
LOC114110058	8.952129392	-0.37653393	0.47597197	-0.791084253	0.428894828	0.525397147
LOC106991327	1.787876633	-0.811698011	1.026324403	-0.790878604	0.429014835	0.52540387
PROX1	17.49119768	0.252427619	0.319156088	0.790922149	0.428989423	0.52540387
TM2D1	27.95696047	-0.225631794	0.285294683	-0.790872764	0.429018243	0.52540387
DNLZ	4.613664008	-0.499847833	0.632162311	-0.790695403	0.429121759	0.525482495
SDCBP	58.19742988	-0.226902162	0.287191695	-0.790072157	0.429485629	0.525879894
NUDT1	9.11998653	-0.365242512	0.462343459	-0.789980921	0.429538911	0.525896957
ZNF70	3.818837832	-0.524700967	0.664712981	-0.789364705	0.429898878	0.526289468
PWWP2A	84.83975784	0.160092987	0.203012756	0.788585855	0.430354101	0.526798509
ANKRD54	6.047173974	-0.442970123	0.562072218	-0.788101792	0.430637167	0.527096738
FBXO6	3.552080714	0.525957525	0.667919635	0.787456301	0.4310148	0.52751065
GOLGA1	13.09240425	0.293684836	0.373148614	0.787045229	0.43125539	0.527660156
HNRNPA2B1	509.7704402	-0.099274694	0.126135634	-0.787047171	0.431254253	0.527660156
SYVN1	10.61702126	0.32490165	0.412749755	0.787163762	0.431186007	0.527660156
MORC4	10.74136608	0.335634644	0.426890743	0.786230785	0.431732294	0.528146974
MYO10	80.59650138	0.168042784	0.213723801	0.786261442	0.431714337	0.528146974
UBE2Q1	7.829721305	-0.390894763	0.497228959	-0.786146413	0.431781716	0.528159093
BCO2	4.316356232	0.458948764	0.583854018	0.786067664	0.431827847	0.528167186
LNPEP	39.94324819	0.225047759	0.286533141	0.785416157	0.432209611	0.528585749
ST8SIA3	3.360919991	0.532485309	0.678847031	0.784396609	0.432807428	0.529268443
GATAD2B	62.4891649	-0.158121652	0.201646225	-0.784153796	0.432949873	0.529365778
PPFIA4	31.9252118	-0.19908409	0.253893013	-0.784125909	0.432966234	0.529365778

ZNF18	4.711077124	-0.475098127	0.605986741	-0.784007463	0.433035731	0.529402322
TMEM222	12.57764581	-0.303241603	0.386911119	-0.783750035	0.433186797	0.52953857
CAMK1	2.707994815	-0.627308134	0.800787968	-0.783363587	0.433413633	0.529736229
VPS33B	3.295391869	-0.549342529	0.701282788	-0.783339528	0.433427757	0.529736229
ZFAT	2.386956396	0.673967692	0.860780902	0.782972404	0.433643318	0.529951228
EI24	55.56379454	-0.171164213	0.218632756	-0.782884577	0.433694895	0.529965804
ECT2	32.4587242	-0.203581717	0.260217569	-0.782351931	0.434007775	0.530299655
TMEM33	35.18435179	0.222243777	0.284110108	0.782245229	0.434070468	0.530327777
NUAK2	9.074829796	0.36252796	0.463760981	0.781712941	0.434383295	0.530661468
NIPSNAP2	62.62980089	-0.154933011	0.198330302	-0.781186784	0.434692645	0.530990852
EYA3	46.118509	-0.166715996	0.213542518	-0.780715701	0.434969724	0.531280758
FAM120B	3.973666744	0.477017643	0.611294949	0.780339579	0.435191022	0.531502485
DPH7	9.872259266	0.352502847	0.451798047	0.780222159	0.435260122	0.531528288
PNPO	37.03888427	0.176956165	0.226817871	0.780168529	0.435291684	0.531528288
ATP5MC3	576.9892097	0.111901949	0.143450492	0.780073648	0.435347527	0.531547917
RARA	8.569451738	0.418435296	0.536571222	0.779831788	0.435489893	0.531673174
GPR180	28.16529784	0.217700507	0.279461112	0.779001077	0.43597908	0.532202848
LRRC61	5.306952266	-0.430379107	0.552504882	-0.778959826	0.43600338	0.532202848
UNC80	2.323292935	0.616357023	0.791826992	0.778398601	0.436334063	0.532557857
TMEM41A	4.510059874	0.482064711	0.619447997	0.778216595	0.436441335	0.532640147
LOC121818028	3.07393914	0.549483791	0.70627954	0.777997605	0.436570425	0.532718678
RAP2C	10.28703121	-0.34719436	0.446281184	-0.777972211	0.436585396	0.532718678
C19H3orf20	3.433230733	-0.562893998	0.723774563	-0.777720062	0.436734062	0.532840542
TLK2	26.64387601	0.201612107	0.259252292	0.777667598	0.436764999	0.532840542
LOC114116879	37.98093426	-0.189920456	0.24428428	-0.777456722	0.436889359	0.532943613
LNX1	13.47100428	0.276126671	0.355879458	0.775899436	0.437808366	0.534015935
STARD9	1.421900316	0.839066441	1.082100487	0.775405289	0.438100211	0.534323151
ZSWIM5	2.242327094	0.65816695	0.849180022	0.775061746	0.438303175	0.534521919
GFM1	14.59136785	-0.293692284	0.379176657	-0.77455265	0.438604046	0.53484004
RECQL	20.3304625	-0.240776944	0.311070803	-0.774026174	0.438915313	0.535170778
RABEP2	4.952992373	-0.457980993	0.591837204	-0.77382934	0.439031719	0.535263884
RBM27	152.7924974	0.134566098	0.173945126	0.773612351	0.439160066	0.535371529
LOC101111733	3.909399834	-0.505028494	0.654226822	-0.771947094	0.440145763	0.536475312
MAP10	3.37872415	-0.565538616	0.732572686	-0.771989766	0.440120489	0.536475312
AGGF1	135.1537859	-0.12769806	0.165469331	-0.771732494	0.440272881	0.536532397
KIAA0753	36.65560753	0.170560825	0.220992471	0.771794731	0.440236013	0.536532397
SLC39A3	1.387064863	-0.819368921	1.062277606	-0.771332199	0.440510052	0.536772482
AFG1L	13.37133325	0.286986529	0.372348675	0.770746743	0.44085706	0.537053391
FGD6	9.726253012	0.377407317	0.489668854	0.770739885	0.440861126	0.537053391
POLR3D	50.63921682	-0.16143255	0.209449851	-0.770745598	0.440857739	0.537053391
LAMB1	25.14943173	0.286582418	0.37193642	0.770514534	0.440994737	0.537167197
MINDY1	17.65468922	-0.243926529	0.31669744	-0.770219454	0.441169726	0.537331379

DHCR24	160.3126582	-0.217318845	0.282321919	-0.769755482	0.441444952	0.537617606
MRPS5	44.83118392	0.171981941	0.223455793	0.769646376	0.441509687	0.537647456
FAM229B	6.918356902	0.394022659	0.512078619	0.769457354	0.441621852	0.537727116
LPCAT1	8.063409452	0.376252525	0.489020351	0.769400546	0.441655565	0.537727116
MFSD12	9.139482558	-0.355782105	0.46317916	-0.768130641	0.442409575	0.538596081
MAPK14	20.16465652	0.221529844	0.288548214	0.76773944	0.442642	0.538782871
MFSD4B	3.411147947	0.548624899	0.714600327	0.767736702	0.442643627	0.538782871
FRMD1	8.138309364	0.364102908	0.474519038	0.767309378	0.442897594	0.53904291
ACACA	9.555907133	-0.329751844	0.429814445	-0.767195817	0.442965099	0.539075983
EEF2K	60.18350328	0.171643912	0.223815151	0.766900327	0.443140779	0.539240682
SLC26A11	14.9618518	0.256401847	0.334432983	0.766676314	0.443273989	0.539353677
RNF31	15.68248211	0.252531077	0.32943472	0.766558779	0.443343892	0.539389629
MCM9	13.03685222	0.289914638	0.378567121	0.765820967	0.443782836	0.539874524
TAF8	33.11921705	0.190134642	0.248298837	0.765749226	0.443825531	0.539877325
R3HCC1L	22.68386143	-0.204973374	0.267762691	-0.765503863	0.443971567	0.540005821
CHFR	8.17391857	0.333265971	0.435504373	0.765241389	0.444127818	0.540146716
DHX37	8.704234635	0.35400468	0.462768013	0.764972233	0.444288079	0.540292464
BLOC1S1	24.6617859	0.224467865	0.293543278	0.764684058	0.444459701	0.540451998
RNF180	1.750887528	-0.756336488	0.989336848	-0.764488344	0.44457628	0.540544579
LOC114118024	3.846295254	0.48744052	0.637721257	0.764347299	0.444660306	0.540596888
LOC132658508	9.675366866	0.307685539	0.402582035	0.764280349	0.444700193	0.540596888
KPNB1	329.7947845	0.101466632	0.132824408	0.763915559	0.444917565	0.540811947
CCER2	3.813319768	0.499979758	0.654687781	0.763691905	0.445050866	0.540924786
LIG4	9.78226473	0.309349281	0.405264393	0.763327069	0.445268363	0.541139929
POLR1A	19.49117969	0.242409529	0.31764177	0.763153814	0.445371669	0.541216268
NSUN4	8.825042786	0.346339725	0.454266481	0.762415321	0.445812165	0.541673607
SPATS2	50.53088333	-0.221615041	0.290685761	-0.762386987	0.44582907	0.541673607
RNF214	89.86417882	0.146842616	0.192691325	0.762061375	0.446023374	0.541860427
CLSPN	48.84395236	0.19518321	0.256233808	0.76173871	0.446215967	0.542045135
ITPKA	11.33164269	-0.308571063	0.405277131	-0.761382866	0.446428418	0.542224481
SLC5A6	20.73583113	0.285549388	0.375053936	0.76135553	0.446444741	0.542224481
SPRED3	1.462529946	-0.813748215	1.069247022	-0.761047913	0.44662845	0.542398315
LOC105606919	4.419759731	-0.49710099	0.65328241	-0.760928172	0.446699971	0.542435887
RGS17	1.159190197	-0.94446245	1.241391019	-0.760809798	0.446770681	0.542472468
LYPLA2	164.6462615	-0.132791749	0.174564141	-0.760704625	0.446833512	0.542499474
BFSP1	2.68685136	-0.560590858	0.737126228	-0.760508631	0.446950612	0.542543082
TMEM241	12.43803501	-0.304539393	0.400409317	-0.760570196	0.446913827	0.542543082
PAQR8	4.254717742	-0.441724168	0.580949453	-0.760348711	0.447046172	0.542609802
ASB13	4.314391476	-0.455963445	0.59991133	-0.760051398	0.447223863	0.542776187
TOMM40L	29.91020456	0.232528129	0.305975401	0.759956938	0.447280325	0.542795427
GATA4	26.45835008	-0.200848786	0.264337052	-0.759820781	0.447361719	0.542844915
ZCCHC10	31.21065839	0.196540373	0.258777522	0.759495538	0.447556182	0.543031584

NSMAF	25.22403967	0.219902902	0.289591493	0.759355532	0.447639907	0.543040783
RENBP	3.664615403	-0.504210038	0.664064208	-0.759279047	0.447685649	0.543040783
SLC35B4	21.15568771	0.228706611	0.301202064	0.759312895	0.447665406	0.543040783
MIEN1	3.323537674	0.535393339	0.705444679	0.75894447	0.447885775	0.543234235
PIAS2	22.56963925	0.213836425	0.281847872	0.758694482	0.448035339	0.543366331
CDPF1	72.09093313	-0.190316704	0.250907706	-0.758512789	0.44814406	0.543448875
HEY1	1.887879592	0.674748224	0.890087257	0.758069749	0.448409228	0.543671784
WDR27	3.535302867	-0.537828363	0.7094557	-0.7580859	0.44839956	0.543671784
ATG16L1	30.27733362	-0.205473227	0.27109749	-0.757931129	0.448492214	0.543723077
SLC22A5	4.23491027	0.478247827	0.631170453	0.757715804	0.448621136	0.543830047
WDR47	7.692632595	0.344425563	0.454641734	0.757575772	0.448704989	0.543882369
ENOSF1	11.28439824	0.289645138	0.382382588	0.757474704	0.448765515	0.543906409
FOCAD	34.40876488	0.205745385	0.271726816	0.757177329	0.44894363	0.543974306
GORAB	7.453386054	0.362308683	0.478486518	0.757197265	0.448931688	0.543974306
PTCD2	5.362440521	-0.415762885	0.549011517	-0.757293558	0.448874009	0.543974306
KTN1	398.8928301	0.110721296	0.146277692	0.756925368	0.449094576	0.544009254
LOC101109035	14.32178667	-0.261333608	0.345256239	-0.756926532	0.449093879	0.544009254
PPP6C	23.84961839	0.200126319	0.264361308	0.75701819	0.449038965	0.544009254
PLEKHF1	2.045900854	-0.680116934	0.899380386	-0.7562061	0.449525636	0.544482071
CTSB	41.75222029	-0.249417864	0.3299184	-0.755998648	0.449650006	0.544583362
SYTL2	18.94459339	0.227341845	0.300760212	0.755890693	0.449714734	0.544612407
LOC132657788	10.76622276	0.324651454	0.430344505	0.754398977	0.450609686	0.545646771
ACTB	1606.269364	0.146379722	0.194188843	0.753800889	0.45096879	0.545989987
IVNS1ABP	169.7315376	0.126189768	0.167421979	0.75372283	0.45101567	0.545989987
TMED2	25.49618079	-0.266028675	0.352942875	-0.753744285	0.451002785	0.545989987
SPN	11.29104762	-0.295693545	0.392494674	-0.753369571	0.451227863	0.546197393
ISL1	3.248298758	-0.498647253	0.662060917	-0.753174278	0.451345194	0.546255145
PWWP3A	40.10914919	-0.237140187	0.314862766	-0.753154111	0.451357311	0.546255145
ACSF3	1.829232355	-0.684114811	0.908496872	-0.753018345	0.45143889	0.54630441
DENND1A	7.356541913	-0.372130416	0.494327144	-0.752801907	0.451568961	0.546412342
LOC105607270	9.913283015	-0.324360936	0.430939732	-0.752682828	0.451640531	0.546449474
LOC101118372	2.243375643	0.626945207	0.834100668	0.751642135	0.452266298	0.547157072
EXOC8	26.28542535	-0.226481955	0.301402014	-0.751428141	0.452395032	0.547263282
FBXO38	24.49531907	-0.254124071	0.338326569	-0.751120646	0.452580051	0.54739459
LOC114113869	3.919976781	-0.517964779	0.689597618	-0.751111614	0.452585487	0.54739459
HDAC8	129.4436951	-0.141651597	0.188691288	-0.750705549	0.452829882	0.547632825
ZNF572	3.015922371	0.591670223	0.788212402	0.750648203	0.452864403	0.547632825
SEPTIN11	168.2831824	0.10544605	0.14048879	0.75056558	0.452914142	0.547643426
LOC121819675	2.129602331	-0.69292827	0.923396267	-0.750412683	0.453006195	0.547705183
GDPD1	8.550621667	-0.327710223	0.436826344	-0.750207095	0.453129986	0.547758554
TMEM42	5.459336272	-0.410743778	0.547509994	-0.750203252	0.453132301	0.547758554
GLMP	2.797727534	-0.542160687	0.72283627	-0.750046324	0.453226805	0.547823248

LOC101119765	6.274568526	0.390387972	0.520845365	0.749527592	0.453539274	0.548123185
LOC114116823	7.454518186	-0.363155298	0.484531231	-0.749498226	0.453556967	0.548123185
NAA20	15.6719839	-0.247978655	0.330906888	-0.749391034	0.453621553	0.548151675
ASAP3	3.0022975	-0.586116921	0.782388042	-0.749138394	0.453773796	0.548286075
ARL10	2.115820208	-0.655816253	0.875900141	-0.748734042	0.454017521	0.548481398
RBM45	72.89510811	0.125640241	0.167793197	0.748780304	0.453989633	0.548481398
TMEM200B	6.800010245	0.379173732	0.506610017	0.748452891	0.45418703	0.548636588
LOC132658423	6.168086223	-0.378472241	0.505923622	-0.748081774	0.454410835	0.54885733
COMMD3	14.83658968	-0.290265314	0.388585606	-0.74697907	0.455076195	0.549611315
LOC132657768	2.033190037	0.658460853	0.881975434	0.74657505	0.455320114	0.54985622
HAUS3	4.660328615	0.444704795	0.596125167	0.745992319	0.455672055	0.550231519
ARPC4	47.17708216	0.203939542	0.273463463	0.745765227	0.455809249	0.550347464
LOC105612743	1.425728774	0.80526473	1.079945642	0.74565302	0.455877046	0.550379604
C7H14orf119	140.2046362	0.126653334	0.169936918	0.745296171	0.456092695	0.550540501
CD164	38.59488258	-0.181556016	0.243585827	-0.745347207	0.45606185	0.550540501
FGG	3.824399049	-0.511078093	0.686255208	-0.744734739	0.456432094	0.550900431
GMPPB	38.15813948	0.195496417	0.262553005	0.744597904	0.456514835	0.55094342
LOC132659829	1.622225371	-0.732322415	0.983591081	-0.744539504	0.456550151	0.55094342
WDR20	11.28677492	0.297716142	0.399967803	0.744350269	0.456664597	0.551031777
LRBA	106.5267354	0.134422516	0.180627963	0.744195492	0.456758215	0.55109499
IFT27	4.993648372	-0.439899884	0.592412287	-0.742556988	0.457749937	0.552241688
ACOX2	2.14608143	0.731244898	0.985132888	0.742280465	0.457917425	0.55239389
LOC132659445	2.076197232	-0.661093518	0.890804333	-0.742131009	0.458007964	0.552405296
NTN4	10.49763305	-0.314942287	0.424377079	-0.74212841	0.458009538	0.552405296
PCYOX1L	2.111994125	0.624298846	0.842013575	0.741435607	0.458429362	0.552861757
C2CD5	11.9388148	-0.328071132	0.442589183	-0.741254292	0.45853927	0.55288638
LOC114111378_1	9.68870677	-0.292846026	0.395098663	-0.74119721	0.458573875	0.55288638
ZBTB17	5.857961542	0.397206496	0.535843247	0.741273682	0.458527516	0.55288638
LOC101105018	7.94170392	-0.369633067	0.498801923	-0.741041784	0.458668106	0.552900244
ZNF689	28.04632912	0.200022642	0.269900204	0.741098523	0.458633705	0.552900244
PEX19	32.22996975	0.203845223	0.275216262	0.740672884	0.458891804	0.553120016
LEKR1	2.253902383	0.624284422	0.84323475	0.740344753	0.459090833	0.553310015
STAT5B	4.164070077	0.502046686	0.678288448	0.740166942	0.459198704	0.553390125
GHITM	203.4774831	0.123767669	0.167319129	0.739710219	0.459475846	0.553674194
KLHL18	12.17267386	0.297798781	0.402881157	0.73917277	0.459802093	0.55396744
RPAP2	29.1892683	0.186812044	0.252725908	0.739188337	0.459792642	0.55396744
FBXL17	58.28614301	-0.163011063	0.220653612	-0.738764537	0.460049989	0.554216148
SARS1	403.8322186	0.125964877	0.170532983	0.738654042	0.460117099	0.55424704
ABI2	9.942985967	0.327512449	0.443553192	0.738383704	0.460281315	0.554394886
RMDN2	3.737407602	-0.47673097	0.645940845	-0.738041221	0.4604894	0.554595542
AGAP3	34.42495776	0.168232331	0.22798106	0.737922402	0.460561605	0.554632527
LOC105605744	2.554356573	-0.565018122	0.766405862	-0.73723095	0.460981915	0.555088674

DNPEP	27.31129903	0.216624802	0.29401771	0.736774672	0.461259388	0.555372758
CCHCR1	4.40976794	-0.431128461	0.585466526	-0.736384476	0.461496749	0.555608499
DPH2	5.73584237	-0.402426666	0.546602477	-0.736232789	0.461589041	0.555630805
TBPL1	27.34365498	0.194903715	0.26473664	0.736217378	0.461598418	0.555630805
ING3	36.50430348	-0.169803451	0.23077568	-0.73579439	0.461855836	0.555890599
ATR	5.116989979	-0.395896845	0.538633065	-0.735002862	0.462337751	0.556420528
SPACA9	1.745275632	0.706167803	0.96187145	0.734160269	0.462851065	0.556988146
CRACD	14.68555672	0.264965411	0.360947788	0.734082381	0.462898531	0.556995117
COQ5	5.522968643	-0.392116857	0.534366495	-0.733797609	0.463072098	0.557153809
CBR4	21.14438385	0.218149247	0.297362885	0.733612896	0.463184699	0.557188974
LOC101118645	6.924227537	-0.392338314	0.534789605	-0.733631153	0.463173569	0.557188974
HLF	8.592280904	0.339510835	0.46289751	0.733447099	0.463285782	0.557260418
MAP3K4	30.66910669	0.210387364	0.286914259	0.733276084	0.463390059	0.557285543
PACSIN1	2.600997986	-0.582966427	0.794998941	-0.733292079	0.463380305	0.557285543
LOC101118904	123.3590294	0.107772375	0.147111282	0.732590824	0.463808029	0.557738023
SIK2	34.41545918	0.165297752	0.225679208	0.732445642	0.463896609	0.557794357
TEF	10.38063547	-0.28381503	0.387837617	-0.731788299	0.464297792	0.558226526
LRRC1	73.46385544	0.221866288	0.303270557	0.731578725	0.464425737	0.558306282
RBCK1	15.95183096	-0.358079381	0.489485208	-0.731542804	0.464447669	0.558306282
SKI	30.41471253	0.181584645	0.248317387	0.73126029	0.464620181	0.558463429
LOC121819183	1.992876025	0.652751035	0.893108173	0.730875671	0.464855098	0.558695552
CENPN	8.058679476	-0.326088791	0.446204475	-0.730805739	0.464897817	0.558696658
GIGYF1	48.47803169	-0.148699002	0.203505478	-0.730687955	0.464969775	0.558732896
AFMID	6.604144318	-0.366536427	0.501806166	-0.730434282	0.46512477	0.558768441
CHD7	156.8878701	-0.140992735	0.19299248	-0.730560772	0.46504748	0.558768441
SSR2	100.8097606	-0.169088509	0.231483285	-0.730456668	0.465111091	0.558768441
GLA	15.12271889	0.249956799	0.34227487	0.730280896	0.465218503	0.558830818
LOC121817445	3.106718689	-0.582494749	0.798357626	-0.729616315	0.465624747	0.559168047
OSBPL7	35.5545145	-0.446057127	0.611312528	-0.729671168	0.465591209	0.559168047
SLC2A11	3.69699995	-0.498083783	0.682563559	-0.729725131	0.465558217	0.559168047
SSR3	75.31747261	-0.152919043	0.209703019	-0.729217173	0.465868828	0.559410902
DIS3L2	8.698451224	0.304211264	0.417316971	0.728969309	0.466020436	0.559542683
LOC101103726	32.22434009	0.187436799	0.257160895	0.728869757	0.466081336	0.559565538
DCAF17	19.8858717	0.255920629	0.351183276	0.728738087	0.46616189	0.559611983
LOC114112248	3.053368121	-0.537314321	0.737632337	-0.728431081	0.466349743	0.559787217
LOC132660133	3.485710222	0.536024811	0.736418083	0.72788111	0.466686368	0.560140983
LOC101121159	12.43953421	0.307275368	0.422256827	0.72769781	0.466798591	0.560218101
SMIM8	2.895833531	-0.525037767	0.721563282	-0.727639252	0.466834446	0.560218101
CROT	46.98732851	-0.190195812	0.261581823	-0.727098733	0.467165475	0.560514698
NPC1	20.20731153	0.275214972	0.378484104	0.727150675	0.467133658	0.560514698
RIC8B	15.214334	0.316600576	0.435688803	0.726666771	0.467430114	0.560781878
TBC1D22B	8.513366418	0.312550555	0.430711053	0.725661794	0.468046129	0.561470522

SORL1	56.00142407	0.148930868	0.205316183	0.725373254	0.468223076	0.561632383
INTU	4.523561431	0.483141575	0.66724044	0.724089168	0.469010994	0.562527005
BIVM	46.36602027	-0.147845846	0.204268317	-0.723782565	0.469199235	0.562694952
LOC105609508	7.31041436	0.338725473	0.468031309	0.723723962	0.469235219	0.562694952
TUBD1	6.348688067	0.390227294	0.539274419	0.723615437	0.469301862	0.562724382
HPS1	3.54846418	0.488917055	0.677170983	0.721999417	0.470294836	0.563823234
PABPC1L	10.7814165	0.294130415	0.407429065	0.721918097	0.470344834	0.563823234
SLC6A20	3.398762208	-0.507602648	0.703115435	-0.721933587	0.47033531	0.563823234
PPFIA3	3.418763731	0.509881227	0.706460807	0.721740288	0.470454167	0.563903722
SSU72	15.38219178	0.233054914	0.323238881	0.720999011	0.470910123	0.564399633
ARHGEF1	5.029901079	0.398342127	0.552693792	0.720728427	0.471076619	0.564447341
C13H20orf204	4.186420331	-0.436200322	0.605199316	-0.720754817	0.471060379	0.564447341
LOC101110567	16.70637192	0.233527601	0.32401109	0.720739531	0.471069786	0.564447341
CEP20	12.42251923	0.296049581	0.410972232	0.720363951	0.471300939	0.564665503
CUL2	15.22128907	0.268969591	0.373723631	0.719701856	0.471708583	0.564904813
DCP1B	7.804777728	0.329952655	0.458389517	0.719808466	0.471642932	0.564904813
DGKE	14.60994995	0.243002711	0.33754349	0.719915265	0.471577169	0.564904813
MYSM1	81.42999715	0.135241582	0.187914792	0.719696305	0.471712002	0.564904813
SMIM3	21.62701311	-0.23578894	0.327603999	-0.719737672	0.471686527	0.564904813
ZNF653	5.296829679	0.409075323	0.568661033	0.719365842	0.471915537	0.565097928
CMTR2	3.396853989	0.49366555	0.687860073	0.7176831	0.472952706	0.566289158
KIF18A	7.746556972	-0.333332439	0.464549377	-0.717539308	0.473041391	0.566344611
COQ8A	5.785530558	0.400206359	0.558988218	0.715947755	0.47402361	0.567469733
TNKS2	17.32009033	-0.261628182	0.365588723	-0.715635262	0.474216594	0.567649919
ADCK5	25.89333354	0.22949019	0.320773059	0.715428504	0.474344304	0.56765028
ARB2A	101.5059504	0.120565925	0.168495688	0.715543087	0.474273527	0.56765028
ZNF529	1.660387174	-0.657960102	0.919606192	-0.715480286	0.474312318	0.56765028
TTF2	34.19365675	-0.201989142	0.282552174	-0.714873786	0.474687036	0.568009573
MYO5B	18.38265372	-0.215561644	0.301731114	-0.714416358	0.47496976	0.568297002
LATS1	49.32236739	-0.156178815	0.21872383	-0.714045721	0.475198908	0.568520283
RNF25	14.38494721	-0.250749115	0.351263279	-0.713849497	0.475320249	0.56856367
TMEM18	11.16780186	-0.278708565	0.390429364	-0.713851445	0.475319045	0.56856367
PDLIM1	16.31575101	-0.255073809	0.357374525	-0.713743682	0.47538569	0.568591064
DPY19L3	14.90871313	0.242486013	0.339819796	0.713572358	0.475491655	0.568666917
CCDC93	43.99032264	0.167862852	0.235529616	0.712703797	0.476029066	0.569172216
IPO8	112.3991122	-0.113984838	0.159937631	-0.712683042	0.476041912	0.569172216
TMEM8B	6.999259822	0.366662751	0.514441864	0.712738945	0.476007312	0.569172216
SESN1	29.79696837	0.189362904	0.266139294	0.711518021	0.476763287	0.56998373
ANO9	6.495726337	0.376320208	0.529236676	0.711062225	0.477045677	0.570270327
PCGF3	43.9920165	0.155718783	0.219037636	0.710922498	0.477132264	0.570322825
KLHL11	22.73014956	0.203425006	0.286340652	0.710430056	0.47743749	0.570636635
DENND3	2.06300097	0.686465751	0.966943097	0.709933969	0.477745084	0.570903943

TMEM161B	105.4260305	0.148985909	0.209859531	0.70993158	0.477746566	0.570903943
CLNS1A	37.79925795	0.166694499	0.235112104	0.709000073	0.478324432	0.571543389
FNBP4	239.5489161	0.099382728	0.140359378	0.708059051	0.478908588	0.572139094
SCARB2	62.71924954	-0.165306528	0.233442007	-0.708126744	0.478866553	0.572139094
TRAF3	32.65989644	-0.194677277	0.275155121	-0.707518278	0.479244458	0.572471338
ZNF770	9.632155537	-0.288067985	0.407178553	-0.707473375	0.479272353	0.572471338
BUD13	18.67694392	0.218576563	0.3091644	0.706991371	0.47957184	0.572777877
TSEN54	12.2343069	0.275050037	0.389240679	0.706632303	0.479795009	0.572993218
MRM3	7.195388764	-0.333860113	0.472552533	-0.7065037	0.479874953	0.573037489
ADGRG1	57.96287209	-0.175519548	0.248709664	-0.705720658	0.480361871	0.573567694
SULT1C2	26.70675252	0.269573305	0.38231132	0.705114632	0.480738902	0.573966606
TGIF1	6.418818771	0.394045002	0.558932285	0.70499596	0.48081275	0.574003503
MDM1	9.088163353	-0.31994673	0.453907312	-0.704872386	0.480889656	0.574044042
IL20RA	10.12072755	-0.313380696	0.444882675	-0.704412002	0.481176234	0.57433484
ARRDC1	17.17874881	0.274539585	0.390066302	0.703827998	0.481539895	0.574666272
EPC2	37.38280153	0.164637238	0.233907187	0.703857114	0.481521761	0.574666272
PI4KB	3.872044772	0.48204081	0.685206979	0.703496643	0.481746298	0.574861264
CD47	77.62713074	-0.154932203	0.220307935	-0.703252943	0.481898131	0.57499111
KLHDC3	55.59554832	-0.144884683	0.206133254	-0.702869041	0.482137366	0.575225211
EIF3G	34.39268518	0.182126664	0.259145976	0.702795648	0.48218311	0.57522844
LOC101102169	2.744189187	0.55800767	0.794093571	0.70269763	0.482244205	0.575249982
MET	45.89708123	-0.160330673	0.228218013	-0.702532945	0.482346864	0.575321094
CDK10	43.30373893	-0.183514283	0.261253554	-0.70243746	0.482406391	0.575340753
LOC114111252	3.074496413	-0.511307594	0.729685681	-0.700723074	0.483475853	0.576564798
MRPS26	115.645594	-0.136336622	0.19480772	-0.699852253	0.484019579	0.577161717
CEP76	5.446171052	0.46641159	0.666526756	0.699764243	0.484074549	0.577170022
GAPVD1	64.78457961	-0.146482628	0.209370457	-0.699633703	0.484156089	0.577170022
HSCB	7.221355947	0.331075976	0.473204283	0.69964704	0.484147758	0.577170022
SPOPL	29.60551233	-0.193428978	0.276504058	-0.69955204	0.484207103	0.577179357
LOC132658276	1.790719102	-0.74418633	1.063934894	-0.699466043	0.484260827	0.577191922
NAA50	9.304894029	-0.335136735	0.479584126	-0.698806981	0.484672666	0.577631284
OPHN1	11.14174704	-0.260722275	0.373594904	-0.697874282	0.48525582	0.578274722
RNF138	15.73659469	-0.243771169	0.349586366	-0.697313145	0.485606843	0.578641443
LOC101102824	5.676564323	0.365913294	0.525206993	0.696703013	0.485988673	0.579044803
EIF4EBP1	17.79741958	0.256503713	0.368466664	0.696138181	0.486342297	0.579414488
LOC114113036	7.026103128	-0.365069537	0.524494464	-0.696040782	0.48640329	0.579435506
NACC1	36.18530529	-0.17939282	0.257797299	-0.695867725	0.486511671	0.579461326
VPS13D	50.46789855	0.159441611	0.229104832	0.695932993	0.486470794	0.579461326
PLK4	12.39265814	-0.291890214	0.419531353	-0.695753039	0.486583504	0.579495243
LOC101106362	2.71114661	0.540644919	0.77715986	0.695667579	0.486637034	0.579507359
NAT9	6.246939781	-0.372017339	0.535044438	-0.69530176	0.486866212	0.579728622
SEPTIN7	146.6266813	-0.105784162	0.152223958	-0.694924526	0.487102602	0.579958434

BMPR1B	4.294639691	0.469293712	0.675722158	0.694506917	0.487364365	0.580122513
PPP1R3D	8.067432879	0.339811497	0.489291569	0.694496939	0.48737062	0.580122513
ST7L	31.13234268	-0.187285944	0.269653661	-0.694542559	0.487342021	0.580122513
BBS1	2.010024389	-0.641460773	0.923989257	-0.694229687	0.487538178	0.580270284
STARD3	30.53040294	0.194705211	0.280603726	0.693879635	0.487757696	0.580479865
OXSR1	26.13254062	-0.186019818	0.268161441	-0.693685929	0.487879192	0.580572764
C7H14orf93	13.02447171	0.261383271	0.376889588	0.69352744	0.487978612	0.580639377
LOC101121643	3.109446278	0.471664665	0.680444896	0.693170994	0.488202249	0.58085377
LOC105602800	3.212903719	-0.498772783	0.719686514	-0.693041725	0.488283367	0.580898574
CACNB2	1.62799424	0.67416468	0.973976569	0.692177513	0.488825859	0.581492206
LTBP3	2.922374866	0.502721228	0.726839066	0.69165411	0.489154572	0.58183145
PEX10	5.885023535	-0.370729586	0.536077524	-0.691559653	0.489213907	0.581850247
PRPF4B	396.3281224	-0.142261785	0.205936022	-0.690805737	0.48968763	0.582361852
BRAF	64.38282567	-0.142011812	0.205614179	-0.690671299	0.48977213	0.582410524
NDUFS7	34.06797518	0.17176786	0.248808815	0.69036083	0.489967303	0.582590781
EXT1	27.33799742	0.203909155	0.295573832	0.689875533	0.490272463	0.582901774
LOC101112888	3.059009081	0.476574681	0.691073115	0.689615427	0.490436063	0.583044421
VMA21	58.00982977	0.139105459	0.201769076	0.689429033	0.490553318	0.583131951
CEP55	13.67414806	0.255187159	0.370239482	0.689248908	0.490666644	0.583214794
PPM1N	1.738707304	-0.695387764	1.00917357	-0.689066563	0.49078138	0.583247438
TRIR	11.58750564	0.288994916	0.419388084	0.689087095	0.490768461	0.583247438
CALU	16.78539684	-0.242938807	0.352857651	-0.688489556	0.491144545	0.583475768
CENPJ	24.84484243	0.201346052	0.2924183	0.688554894	0.491103414	0.583475768
KIAA0513	36.74997899	-0.158558381	0.230336294	-0.688377754	0.491214929	0.583475768
LIN7B	1.141300859	-0.915594108	1.329897147	-0.688469864	0.491156941	0.583475768
LOC101110131	4.379850215	-0.414679628	0.602316906	-0.688474164	0.491154234	0.583475768
TXNRD2	10.57260972	0.296336058	0.430505021	0.688345184	0.491235434	0.583475768
ATAD5	22.81534723	0.201356627	0.292736947	0.687841522	0.491552585	0.583800592
CDC42BPA	51.22349047	0.139374034	0.202835854	0.687127207	0.492002571	0.584283106
PLCH1	7.243671651	0.329966737	0.480927224	0.686105341	0.492646683	0.584996053
AXIN1	6.434519154	0.351990602	0.51311989	0.685981208	0.492724958	0.585037025
ERC1	22.78814101	0.204730545	0.299169662	0.68432923	0.493767293	0.586170498
LOC101109476	2.467319134	-0.535225142	0.782086042	-0.684355829	0.493750501	0.586170498
DHTKD1	3.094799106	0.528532231	0.772827274	0.683894382	0.494041862	0.586444363
FGGY	1.931007584	-0.599898775	0.878073826	-0.683198561	0.494481382	0.586913964
DDX50	31.08729038	0.16011048	0.23443997	0.682948732	0.494639239	0.586997076
MTBP	8.606369698	-0.34323163	0.502530153	-0.683007035	0.494602397	0.586997076
FREM2	1.569280687	0.691918729	1.014127005	0.682280154	0.49506182	0.587414524
OGA	179.5428734	-0.118063781	0.173049819	-0.682253133	0.495078903	0.587414524
DZANK1	2.484618412	-0.586413651	0.859937387	-0.681925986	0.495285753	0.587607791
SCPEP1	27.90476273	0.197347626	0.289548999	0.681569013	0.495511514	0.587823458
FBXO2	2.037204388	-0.688547619	1.01034777	-0.681495659	0.495557913	0.587826328

SCAND1	109.8014059	0.130856231	0.192278599	0.680555357	0.496152883	0.588479851
TTC7B	14.46920243	-0.257855833	0.379041929	-0.680283137	0.4963252	0.588631998
IGFLR1	12.86179521	-0.268454278	0.394749226	-0.680062835	0.496464675	0.588654322
LOC132659355	40.23847219	0.166435407	0.244722989	0.680097148	0.49644295	0.588654322
RIMKLB	43.60660531	-0.193117143	0.283977123	-0.680044718	0.496476147	0.588654322
MFHAS1	46.96882234	0.146319381	0.215402039	0.67928503	0.49695728	0.589172521
DLGAP1	2.626244847	-0.514180455	0.757375496	-0.678897664	0.497202706	0.589411208
PHYH	116.76344	0.159103042	0.234534369	0.678378368	0.497531821	0.589749053
LOC105609749	3.620927208	-0.457640871	0.67500796	-0.67797848	0.497785338	0.589944922
PAXBP1	61.12996891	-0.150105165	0.221395203	-0.677996465	0.497773935	0.589944922
FAM168A	9.112503336	-0.344268747	0.507910916	-0.677813247	0.497890111	0.590016777
COP1	57.33724175	0.15319681	0.226042511	0.67773451	0.497940042	0.590023634
LOC121817471	3.934671674	0.443447047	0.654596127	0.6774361	0.4981293	0.59019557
EIF4H	80.10633998	0.133794773	0.197766528	0.676528906	0.4987049	0.590825181
LOC114117985	5.08606539	0.38228426	0.565269874	0.676286278	0.498858903	0.590955251
CLEC16A	41.42560181	0.169697949	0.251022191	0.676027678	0.499023073	0.59109734
STRN4	11.13782369	0.268772622	0.397875845	0.675518822	0.499346197	0.591427672
ANKRD46	13.60320922	0.233821743	0.346345792	0.675110681	0.499605448	0.591682298
PTRH2	6.189413199	-0.363109769	0.538401661	-0.67442171	0.500043244	0.59209202
SNX16	32.63930848	0.178578872	0.264796541	0.67440032	0.500056839	0.59209202
ZNF667	1.81649292	-0.610218978	0.904889961	-0.674357109	0.500084304	0.59209202
LOC105601990	5.551887651	0.381948938	0.56657116	0.674141158	0.500221575	0.592149638
RECQL5	4.403797013	0.458619954	0.680284815	0.67415874	0.500210398	0.592149638
DOC2B	1.554008576	0.697157042	1.034429364	0.673953261	0.500341029	0.592158238
RRP15	59.63021099	-0.135938394	0.201708957	-0.673933356	0.500353684	0.592158238
TENT4A	24.80032793	-0.187367751	0.278026419	-0.673920671	0.50036175	0.592158238
NFYA	35.49024274	-0.162060882	0.240559845	-0.673682185	0.50051339	0.592285257
SETD6	7.480078233	-0.309603505	0.459651266	-0.673561737	0.500589986	0.592323456
RNF6	6.961418137	-0.309618554	0.459859662	-0.673289221	0.500763309	0.592476089
COPS9	214.9118457	-0.166202366	0.247305825	-0.672051968	0.501550613	0.593348996
PATL1	7.222167786	0.336474681	0.500713606	0.67199029	0.501589878	0.593348996
LOC132657331	6.95015292	-0.337035098	0.502098818	-0.671252522	0.502059675	0.593852178
SEMA4D	37.65575208	-0.147279942	0.219622577	-0.670604742	0.502472361	0.594287724
SPINDOC	5.140268564	-0.36287597	0.542261066	-0.66919053	0.503373946	0.595301377
PTPN21	16.16489294	0.240252261	0.359058306	0.669117681	0.503420412	0.595303656
TXNDC11	17.76629896	-0.212231361	0.317289101	-0.668889542	0.503565942	0.59542307
POFUT1	10.44057966	-0.272815651	0.408062318	-0.668563695	0.503773838	0.595616198
ILK	13.23513354	0.24747492	0.370404977	0.668119855	0.504057089	0.595898377
ADAMTS6	2.924353434	0.476108025	0.712877697	0.667867751	0.504218014	0.596035905
KEF53_p05	51.85795781	0.214251499	0.32097616	0.667499728	0.504452984	0.596260929
FAM118B	4.192440659	-0.41246803	0.618212316	-0.667194779	0.504647726	0.596391378
PHPT1	20.47632095	0.202671638	0.303770289	0.667187166	0.504652588	0.596391378

INPP4A	8.082852946	0.294795325	0.441925241	0.667070577	0.504727053	0.596426646
EDC3	3.182370858	0.485259733	0.727621403	0.666912395	0.504828093	0.596493308
PGS1	4.718383024	0.409795078	0.614586029	0.666782286	0.504911209	0.59653878
ZNRD2	3.347573775	0.454876103	0.682276958	0.666703013	0.504961854	0.596545885
PYCR2	16.19681415	0.211398868	0.317393143	0.666047369	0.505380823	0.596988077
ECHDC3	10.88947487	0.270551203	0.406448195	0.665647446	0.505636473	0.597237283
LOC105608991	3.639707325	-0.474261146	0.713388966	-0.664800226	0.506178278	0.597824412
TECPR2	26.81847126	0.197750441	0.297753829	0.664140716	0.506600253	0.598269922
LOC101108033	1.104604758	-0.824573748	1.242653556	-0.663558837	0.50697271	0.598656882
SEC61B	52.49425187	-0.199381528	0.30052549	-0.663442985	0.507046884	0.598691577
ACAD9	18.66929304	-0.198234919	0.298910059	-0.663192534	0.507207252	0.598828031
SMC1A	428.7485282	0.118247437	0.178411819	0.662778046	0.507472716	0.599088529
NFKBIB	4.265202326	-0.402193057	0.607024937	-0.662564307	0.507609636	0.599197244
PSMC1	102.7544014	-0.159389072	0.240604202	-0.6624534	0.50768069	0.599228197
INCENP	28.70593758	0.194146473	0.293197637	0.662169297	0.507862729	0.59927491
SMG8	12.35300253	-0.235199617	0.355195168	-0.662170092	0.507862219	0.59927491
SNX12	55.15032738	0.149506723	0.225802857	0.662111744	0.50789961	0.59927491
VPS41	22.68096285	0.192118138	0.290109598	0.662226064	0.507826353	0.59927491
BRAT1	2.374332543	0.527228377	0.796993477	0.661521571	0.508277885	0.599668304
DGKH	49.10760618	0.136430336	0.206309215	0.661290556	0.508425997	0.599790104
PAXIP1	7.257732563	0.333864715	0.505280303	0.660751494	0.508771694	0.600124537
PPP5C	5.985078178	-0.349658737	0.529217876	-0.660708477	0.508799286	0.600124537
LOC101111046	5.05169687	0.368997418	0.558844905	0.660285913	0.509070368	0.600384746
PNKD	3.884413803	-0.414751568	0.628197728	-0.660224559	0.509109734	0.600384746
LOC101113508	20.21072692	-0.196863329	0.298534504	-0.659432413	0.509618134	0.600890986
LOC114115665	1.813703406	-0.771906854	1.170592339	-0.659415603	0.509628925	0.600890986
CELSR2	9.14319599	-0.308006064	0.46738482	-0.658998862	0.509896499	0.601153445
PARPBP	11.77296184	-0.252588529	0.383791148	-0.658140581	0.510447802	0.601750337
TMEM160	3.5231064	-0.436860365	0.663974371	-0.657947633	0.510571781	0.60184341
OVCA2	32.48110677	-0.166724794	0.253499801	-0.657692011	0.510736057	0.601877811
PPP1R13L	11.87474994	0.285627593	0.434286169	0.657694428	0.510734504	0.601877811
SLC35A1	52.75891691	-0.211142652	0.321015214	-0.657734097	0.510709009	0.601877811
KRI1	9.980540426	-0.272755677	0.4164386	-0.654972131	0.512485698	0.603886435
DDX24	160.3998598	-0.130381287	0.199127074	-0.65476424	0.512619558	0.603960863
LOC114118386	5.929979972	0.350219311	0.534903456	0.654733686	0.512639233	0.603960863
SEC22B	40.0700719	0.145287383	0.222020904	0.654386054	0.512863117	0.604171375
APOM	5.023718835	-0.349325047	0.534126037	-0.654012392	0.513103822	0.604401664
TRAPPC14	2.808339684	-0.480400951	0.734931456	-0.653667695	0.51332592	0.604609998
CCNA2	32.00688894	-0.175523424	0.268882804	-0.652787836	0.513893067	0.605111039
CIAPIN1	19.41555048	0.194849771	0.298516566	0.652726828	0.513932404	0.605111039
LOC101118024	17.35427961	0.276444968	0.4234557	0.652830907	0.513865296	0.605111039
ZNF189	25.50992739	0.191861555	0.29392027	0.652767347	0.513906278	0.605111039

DNAJC27	3.795597963	-0.462191279	0.708227588	-0.652602761	0.514012405	0.605151925
FBXL14	24.20596055	-0.227994068	0.349522457	-0.652301628	0.51420661	0.605327246
PRPF38A	72.230381	0.116587633	0.178755505	0.652218418	0.51426028	0.605337111
LZTR1	2.96551929	0.488692839	0.74965202	0.651892913	0.514470257	0.605530949
SFT2D2	7.036528099	-0.3234266	0.496341348	-0.651621311	0.514645497	0.60568387
NDUFA11	67.29757571	0.160534779	0.246428727	0.651445068	0.514759226	0.605764379
ALAS1	24.3483617	0.218420395	0.33571587	0.650610872	0.51529771	0.606344678
HS1BP3	3.474901719	-0.424384759	0.652614526	-0.650283962	0.515508815	0.606526748
ZFYVE9	25.77316426	0.182644441	0.280891743	0.650230723	0.515543198	0.606526748
TMEM102	9.304903925	-0.287351152	0.44203463	-0.650064797	0.515650367	0.606599438
FAM111B	8.556231009	0.294018504	0.452702781	0.649473598	0.516032306	0.606995319
LOC105611310	5.823506479	0.353332706	0.544193844	0.649277293	0.51615916	0.607091106
ALDH7A1	36.98876368	-0.159251566	0.245343636	-0.649095975	0.516276343	0.607175504
HSF1	35.22981431	-0.169865862	0.261792971	-0.648855703	0.516431649	0.607304718
ADIPOR2	73.6454066	0.155055709	0.239373781	0.647755607	0.517143032	0.608087777
AMFR	26.59722914	-0.196480178	0.303531817	-0.647313285	0.517429205	0.608370756
PTPN4	27.14621297	-0.167572467	0.258934358	-0.647161962	0.517527127	0.608432367
CCDC32	31.57063519	0.162660039	0.251373261	0.647085686	0.517576489	0.608436882
NHERF4	6.027891089	0.402299973	0.621810624	0.646981504	0.517643914	0.608462629
ADAR	33.72387098	-0.162582292	0.251350605	-0.646834698	0.517738933	0.608520804
EME1	7.07870632	0.319153642	0.493651522	0.64651607	0.517945194	0.608572752
LOC105606886	2.072853957	0.578467994	0.894789398	0.646485079	0.517965257	0.608572752
LOC105611487	1.643124948	-0.631447814	0.976593162	-0.64658226	0.517902342	0.608572752
LOC132659360	6.809783595	0.323039927	0.499617698	0.646574228	0.517907542	0.608572752
FARSB	28.43900786	-0.172993956	0.267958268	-0.645600369	0.518538199	0.609192367
C5	78.61485339	-0.168386836	0.260883049	-0.645449512	0.518635927	0.60925363
ATP11A	77.49179981	-0.145128388	0.225313739	-0.644116906	0.519499629	0.610160987
PAPSS1	18.565809	0.197943827	0.307293582	0.644152166	0.519476766	0.610160987
NIPAL1	30.3094492	-0.168573717	0.261826687	-0.643837033	0.519681117	0.610320517
PLS1	113.1495283	-0.113621721	0.176924229	-0.642205545	0.520739733	0.611476509
SPEF1	4.229832283	0.376977344	0.587028398	0.64217906	0.520756928	0.611476509
LOC114118046	4.021923242	-0.433881407	0.675808017	-0.642018733	0.52086102	0.611522343
RRAGB	50.80168119	-0.173696646	0.270564794	-0.641978002	0.520887466	0.611522343
CTNND2	3.023627335	0.44413734	0.692736646	0.641134466	0.521435323	0.612099219
TOM1	8.882035892	0.269291223	0.420091673	0.641029661	0.521503412	0.612099219
ZRANB3	2.627341757	-0.511783849	0.798402366	-0.641009935	0.521516228	0.612099219
HPSE	2.053957108	-0.602846935	0.940639532	-0.640890495	0.521593832	0.612114986
PDCD4	147.6683957	-0.093302027	0.145607455	-0.640777812	0.521667051	0.612114986
SUZ12	92.54806345	-0.154661205	0.241341008	-0.640840968	0.521626013	0.612114986
PLD1	85.96227553	-0.133733722	0.208819802	-0.640426439	0.521895399	0.61232917
LOC101106243	62.54837858	0.141945648	0.221883063	0.639731787	0.522346986	0.612805216
FGF18	1.740358422	-0.583234522	0.91235973	-0.639259387	0.522654204	0.613111822

TRAPPC10	30.20830946	-0.15302996	0.239421393	-0.639165773	0.522715095	0.61312944
ARRDC4	26.57551148	-0.252282223	0.394982904	-0.638716815	0.523007172	0.613163877
EPS8	99.50836061	-0.104502778	0.16356792	-0.638895315	0.522891036	0.613163877
EXOSC1	11.11970982	-0.234094441	0.366491916	-0.638743805	0.522989611	0.613163877
LSM3	32.84395573	-0.166547462	0.260653631	-0.638960837	0.52284841	0.613163877
PRKRIP1	27.63853731	0.169687926	0.265586413	0.63891795	0.52287631	0.613163877
SUGT1	93.65802731	-0.132964573	0.208180807	-0.638697555	0.523019704	0.613163877
PEX11A	61.67738557	0.159430501	0.249727553	0.638417744	0.523201783	0.613313684
PSMB7	71.0914758	0.117087514	0.183419207	0.638360159	0.523239259	0.613313684
MMP15	30.84238498	-0.164881842	0.25839833	-0.638091749	0.523413957	0.613464657
CDC14B	11.01982636	0.263370794	0.413107275	0.637536082	0.523775715	0.613834827
PDX1	165.8702315	0.115888723	0.182085596	0.636451901	0.524481922	0.614608571
LOC101113786	4.654977599	0.396053226	0.622442562	0.636288792	0.524588208	0.614679232
KIF22	4.752729737	0.356272676	0.560617166	0.635500834	0.525101823	0.615227118
LOC105606262	2.154596953	0.56743383	0.893782592	0.634867847	0.525514609	0.615602828
UPRT	19.05456574	-0.218452153	0.344071482	-0.634903397	0.525491422	0.615602828
ST7	5.50337293	-0.335218157	0.528275467	-0.634551816	0.525720762	0.615790352
CNKSR3	20.75571683	-0.180486513	0.284517098	-0.634360868	0.525845341	0.615828339
GNL1	8.76520175	-0.293353964	0.462400988	-0.634414655	0.525810248	0.615828339
PRDX6	52.49481641	0.13032171	0.20579981	0.633245046	0.526573634	0.616627229
DNASE1L1	12.95849281	0.240519832	0.380054837	0.6328556	0.526827944	0.616838782
NAF1	28.11631968	-0.180209567	0.284769065	-0.632827049	0.526846591	0.616838782
DDX3X	115.3367181	-0.097584196	0.154259392	-0.632598081	0.52699614	0.616959833
FBXL5	20.54296553	0.180323195	0.285281656	0.632088294	0.527329184	0.617295662
AFDN	287.7327376	0.103344783	0.163536176	0.631938363	0.527427154	0.617356278
IRAK1BP1	2.526594382	-0.549051137	0.869255015	-0.631634133	0.527625978	0.617534923
TMEFF1	4.930296015	-0.366514276	0.580445074	-0.63143662	0.527755079	0.617631939
HNRNPF	195.8917289	-0.169664482	0.26920924	-0.630232758	0.52854231	0.618499082
LOC114114038	1.782496569	-0.650772382	1.034886263	-0.62883469	0.529457287	0.619515547
ZFP36L1	261.4681153	-0.093095376	0.148080773	-0.628679702	0.529558769	0.619580051
PRDM15	4.743765397	-0.374970034	0.596614405	-0.628496447	0.529678774	0.619666213
AKT2	9.540616128	0.265025409	0.421823042	0.628285757	0.52981676	0.619694572
LOC132658503	5.386576707	0.335045488	0.533214939	0.628349776	0.529774831	0.619694572
LOC132658785	2.66013632	0.504147957	0.80246767	0.628247063	0.529842104	0.619694572
AP1M2	28.66931654	0.16383456	0.261024596	0.627659471	0.530227041	0.620090527
LOC101108868	2.339598228	0.524151964	0.835700063	0.627201059	0.530527449	0.620387566
GOLGA7	28.02356742	-0.168105204	0.268150352	-0.626906518	0.530720515	0.620559041
INTS10	13.3868899	0.217297869	0.346718145	0.626727709	0.530837737	0.620587527
TMEM230	29.39821208	-0.179319289	0.286104703	-0.626761069	0.530815867	0.620587527
LOC105602080	5.734783846	0.330443627	0.52768554	0.626213156	0.531175139	0.620927664
PEX26	39.50616467	0.161312489	0.257661785	0.626062919	0.531273673	0.620988536
RAB5IF	23.95304917	0.178415334	0.285037192	0.625937033	0.531356243	0.62103074

CNIH1	34.11149453	0.161287638	0.257844625	0.625522592	0.531628125	0.621294179
GPRASP1	1.495942299	0.620567553	0.992296881	0.625384968	0.531718425	0.621345382
FAM8A1	21.09523222	-0.212483038	0.339958361	-0.625026658	0.531953562	0.621565811
FAM228B	33.94048152	-0.17523835	0.280759453	-0.624158324	0.532523614	0.622173336
TMEM41B	74.7221838	-0.120722147	0.19343619	-0.624092869	0.532566597	0.622173336
SMOX	3.306006091	-0.422379172	0.677113239	-0.623793994	0.532762884	0.622348253
FBXO46	12.42937932	-0.253230918	0.406212764	-0.623394782	0.533025126	0.622545774
MPV17	28.80807651	-0.209252155	0.335639375	-0.623443404	0.532993183	0.622545774
ANKRD9	2.453547616	-0.490117601	0.786417943	-0.623227897	0.533134772	0.622619429
LOC101120961	2.810061782	0.453118663	0.727164232	0.623131121	0.53319836	0.622639287
ILRUN	152.7291322	0.102984205	0.165381197	0.622708062	0.533476384	0.622909526
ERGIC1	66.22676492	0.145749968	0.234140841	0.622488446	0.533620739	0.623023654
SLC35A3	173.3050449	0.113187938	0.181954688	0.622066622	0.533898062	0.623292994
EXOC1	46.42740782	-0.142870433	0.229832493	-0.621628524	0.534186161	0.623574867
LDHD	1.797553262	-0.613462118	0.988176872	-0.620801939	0.534729947	0.624155137
EXOC5	70.84377771	0.136581572	0.220133469	0.620448914	0.534962277	0.624371795
CORO2B	4.414281834	-0.404653462	0.653478183	-0.619230255	0.535764683	0.625253712
GBE1	20.07407667	0.204324901	0.330094004	0.618990042	0.535922919	0.625383773
ZNF132	9.941354936	0.24526407	0.396379763	0.618760323	0.536074264	0.625505771
CCNB3	3.239049885	-0.494016837	0.798995628	-0.618297297	0.536379383	0.625774987
LOC105611964	2.573069561	-0.516403358	0.835337769	-0.618197066	0.536445444	0.625774987
LOC114113871	13.80402859	0.242523712	0.392276539	0.618246793	0.536412669	0.625774987
LOC132658690	2.681182173	-0.482757795	0.78115192	-0.618007564	0.536570353	0.625866073
PCYOX1	71.00377673	-0.134556242	0.217812114	-0.617762897	0.536731644	0.625999578
SELENOS	87.41458894	0.127278369	0.206090701	0.617584241	0.536849435	0.626082327
AKIRIN1	16.1043557	-0.20446551	0.331113582	-0.617508675	0.536899261	0.626085807
PDK2	18.37189427	0.196811257	0.319128973	0.616713848	0.537423486	0.626642442
PPP1R12C	29.53319009	0.166016535	0.269428852	0.616179499	0.537776057	0.626998847
LOC101106288	83.50961304	-0.115432391	0.187527721	-0.615548412	0.538192608	0.627429778
LOC101116076	26.5046378	0.22557792	0.366593245	0.615335725	0.538333029	0.627538746
DHDDS	7.975440098	0.272426075	0.443066845	0.614864502	0.538644208	0.627758563
LAPTM4A	93.75127837	0.112230064	0.182536346	0.614836807	0.538662499	0.627758563
ODF2	29.83803676	-0.154592163	0.251423038	-0.614868728	0.538641417	0.627758563
RAB10	203.6510786	-0.073653926	0.119840224	-0.614601033	0.538818232	0.627885308
ZNF330	41.3438405	-0.138022689	0.224806814	-0.613961322	0.539240886	0.628323048
TM9SF4	38.97897551	0.144988948	0.236327248	0.613509231	0.539539681	0.628616403
CDAN1	12.53542488	-0.213896913	0.349026068	-0.612839362	0.539982562	0.629077566
PUF60	96.15219383	-0.140041931	0.228841813	-0.611959543	0.540564525	0.629700666
NUDT3	42.28478177	-0.139260572	0.227706749	-0.611578587	0.540816609	0.629939416
PLEK2	11.32356329	-0.236393485	0.386596332	-0.611473689	0.540886032	0.62996538
GCNT1	20.94572634	-0.239140054	0.391192401	-0.611310581	0.540993987	0.630036215
LOC105609244	3.119981857	-0.443419224	0.725803728	-0.610935446	0.541242318	0.630270502

ADGRG2	2.001691389	-0.598103679	0.979284309	-0.61075591	0.541361186	0.630354004
RETREG1	20.45143225	-0.215434023	0.352797187	-0.610645523	0.541434278	0.630384195
LDLRAD3	27.53847575	-0.158898597	0.260308837	-0.610423367	0.541581393	0.630427401
PIGO	12.72855896	-0.242134341	0.39674035	-0.610309339	0.541656912	0.630427401
RTP4	1.698637444	-0.579566972	0.949635601	-0.610304596	0.541660053	0.630427401
ZNF32	5.59222557	0.321725204	0.527117248	0.610348466	0.541630998	0.630427401
FAAP20	19.99884345	0.176868235	0.289961988	0.609970418	0.541881404	0.630630113
GGCT	7.815275243	-0.277158891	0.454496819	-0.609814811	0.541984489	0.630695167
HK1	93.19100963	-0.15190377	0.249157627	-0.609669354	0.542080859	0.630708625
SH3D21	3.022335291	0.467206387	0.766345648	0.60965491	0.542090429	0.630708625
DTX3L	33.77178705	0.147657538	0.242593248	0.608663016	0.542747826	0.631198768
LOC121819422	1.832571891	-0.537028304	0.88222595	-0.608719686	0.542710256	0.631198768
NEK11	11.99360097	0.235267818	0.38642588	0.608830386	0.54263687	0.631198768
SOWAHC	1.775523901	-0.619338167	1.017491081	-0.608691495	0.542728946	0.631198768
THAP5	44.8371237	-0.131719429	0.216371491	-0.608765177	0.542680098	0.631198768
ZNF513	10.79292754	-0.248526324	0.408525558	-0.608349512	0.542955689	0.63138557
CITED4	2.264469655	0.479611726	0.789263783	0.60766975	0.54340653	0.631854865
METTL21A	16.22778191	0.191240019	0.314849398	0.607401569	0.543584448	0.632006761
KAT2B	37.4299625	0.187302828	0.308906856	0.606340792	0.544288476	0.632715235
VTA1	32.04327106	0.14800195	0.244067399	0.606397866	0.544250585	0.632715235
GTPBP1	3.690102714	0.455396834	0.751857561	0.605695623	0.544716891	0.633103127
LOC105609873	15.64938292	-0.21152827	0.349217897	-0.605720015	0.544700691	0.633103127
ALDH2	15.6577342	0.196435063	0.324456991	0.605427123	0.544895233	0.633255342
LOC121816291	1.33727638	-0.781087505	1.291784346	-0.604657819	0.545406381	0.633794269
CFAP44	3.672359356	-0.395980567	0.655366794	-0.60421213	0.545702617	0.634083385
NPHP3	5.92829017	0.367915779	0.609447027	0.603687871	0.546051179	0.634433246
CHAF1A	27.52608996	0.206416517	0.342108956	0.603364845	0.546266004	0.634550412
EIF5A2	3.414990337	-0.416923704	0.691046655	-0.603322079	0.546294448	0.634550412
LOC101117028	4.982481972	-0.352039296	0.583489453	-0.603334464	0.546286211	0.634550412
YIF1A	21.57969497	0.212167717	0.351864166	0.602981882	0.546520742	0.634758102
LOC101114495	5.67518374	-0.391234499	0.649131866	-0.602704196	0.54670549	0.634917506
ARHGAP29	53.9290554	0.126376206	0.209842536	0.602243036	0.547012373	0.635218712
MYL6	259.3664529	-0.125306493	0.208100236	-0.602144886	0.547077699	0.635239382
ME3	1.575042363	0.576412325	0.957597207	0.601936097	0.547216675	0.635345559
ESAM	8.054201997	0.271345062	0.450931989	0.601742765	0.547345379	0.635384604
LSS	21.71848198	0.163485751	0.271661523	0.601799433	0.547307653	0.635384604
ARF1	3.688756466	-0.374033843	0.621944667	-0.601394085	0.547577538	0.635598903
WDR81	5.46145457	-0.322772217	0.536820254	-0.601266839	0.547662273	0.635642058
WARS2	20.98497468	-0.205919916	0.342632261	-0.600993949	0.547844017	0.635797788
DPH6	3.943793962	-0.394426001	0.656883747	-0.600450235	0.548206217	0.636162901
CGAS	9.573408232	0.245582797	0.409466424	0.599762966	0.548664218	0.636639112
LOC101122372	51.43012901	-0.151833331	0.253713722	-0.598443512	0.54954404	0.637559443

MINDY3	20.42118212	0.176431961	0.294824518	0.598430422	0.549552772	0.637559443
LOC101117184	7.146649194	-0.285057581	0.476509662	-0.598219939	0.549693189	0.637666999
RAB29	2.257874515	0.496767334	0.830516512	0.598142634	0.549744765	0.637671486
LOC121820457	3.218386779	-0.421030431	0.704133761	-0.597940979	0.549879315	0.637772208
LOC101109655	20.47885825	-0.208794362	0.349320841	-0.59771516	0.550030008	0.637891634
GABPB2	6.732125823	-0.2937942	0.492077934	-0.597048109	0.550475261	0.638297245
PALLD	35.63711598	0.171395638	0.28705792	0.597076847	0.550456075	0.638297245
AP3M1	11.28571223	0.228314703	0.38246165	0.596961036	0.550533395	0.638309278
ARRB2	9.921029771	0.242727051	0.407012518	0.596362618	0.550933008	0.638717198
IL27	1.989122651	-0.526963282	0.884007806	-0.596107046	0.551103718	0.638804292
TTC5	27.48541297	-0.255126918	0.427948437	-0.596162752	0.551066506	0.638804292
ABCG1	4.206826079	-0.381637241	0.640911971	-0.595459686	0.551536239	0.639219059
AK8	3.100789982	-0.45611539	0.766029031	-0.595428334	0.55155719	0.639219059
CEP70	32.00344716	-0.1441818	0.242588795	-0.594346495	0.552280387	0.640001704
C3H12orf57	31.42673093	-0.199949639	0.336548307	-0.59411869	0.552432731	0.640109934
SCARB1	11.29626806	0.253921482	0.427431466	0.594063616	0.552469565	0.640109934
LOC114110433	5.18874022	-0.359624264	0.605550392	-0.593879994	0.55259238	0.6401696
MRPL47	16.90081498	-0.195359383	0.328974575	-0.59384341	0.552616852	0.6401696
DNM1L	22.46189113	-0.167707522	0.282542535	-0.593565576	0.552802711	0.64027392
ERMARD	6.565743738	0.313577797	0.528268511	0.593595474	0.552782709	0.64027392
TYK2	13.67221233	0.228906421	0.385924893	0.593137226	0.553089319	0.640550372
S1PR2	2.965508747	-0.408710905	0.689339229	-0.592902432	0.553246451	0.640676838
LOC132657358	2.224876	0.495807077	0.836472591	0.592735592	0.553358118	0.640750637
HAVCR2	5.310147254	-0.309890224	0.523816742	-0.591600457	0.554118167	0.641575141
EML4	409.4219297	-0.070605205	0.119428211	-0.591193692	0.554390649	0.641835029
PPP3CB	6.896925242	-0.288741977	0.488608596	-0.590947396	0.554555668	0.641970471
LMAN2L	11.66073965	-0.211634086	0.358435632	-0.590438191	0.554896913	0.642309876
SWI5	11.15988793	-0.232221331	0.393714739	-0.589821278	0.555310476	0.642732925
ULK3	25.66917475	-0.151446864	0.256935178	-0.589436078	0.55556878	0.642976216
SLC24A1	1.322565808	-0.63520365	1.077887647	-0.589304138	0.55565727	0.643022949
HDAC10	3.316208445	-0.411826404	0.699439066	-0.588795257	0.555998628	0.643306585
LOC105615690	11.30428971	-0.23981756	0.407272869	-0.588837554	0.555970251	0.643306585
HAX1	5.040561326	-0.322639234	0.548072125	-0.588680247	0.556075791	0.643340174
LOC101116886	82.06487676	0.136192174	0.231387002	0.588590424	0.556136059	0.643354212
ABHD17A	45.34846567	0.140059345	0.238088637	0.58826556	0.556354059	0.643550701
POLE2	1.723500238	0.565573118	0.962163753	0.587813786	0.55665729	0.643845737
BBS5	1.233284119	0.685923635	1.168060921	0.587232757	0.557047395	0.644241195
JMJD8	5.651782307	-0.324616607	0.553015704	-0.586993471	0.557208092	0.644371289
IDH3G	30.20597271	0.151699741	0.258510591	0.586822149	0.557323159	0.644448598
LIPG	26.3076178	-0.176343513	0.300779034	-0.586289246	0.557681156	0.644806777
UBA1	23.07009557	0.180245704	0.308037318	0.585142427	0.558451953	0.645642142
ANKRD40	10.67551132	-0.240197484	0.410561059	-0.585046922	0.558516167	0.645660534

ZNF496	2.299398643	-0.490656945	0.840206435	-0.583971896	0.55923922	0.646440493
WHRN	1.844427429	0.598382652	1.024822473	0.583889081	0.55929494	0.646448994
LOC101122752	2.184174424	-0.489924738	0.839272415	-0.583749363	0.559388951	0.646501748
MED31	9.555677253	0.242123943	0.414867773	0.583617139	0.559477927	0.646548674
NMNAT2	19.85333849	-0.17298768	0.29648958	-0.583452814	0.559588513	0.646620565
E2F8	14.16037814	-0.221171041	0.37912857	-0.583366852	0.559646367	0.646627127
METTL13	15.8328462	0.224169956	0.384312909	0.583300614	0.559690949	0.646627127
RAB26	43.37232323	0.15506978	0.265883208	0.583225173	0.559741727	0.646629898
GCNA	10.23344116	-0.248121555	0.425487482	-0.583146544	0.559794653	0.646632375
JAKMIP2	1.843569733	0.583634542	1.00095407	0.583078244	0.559840629	0.646632375
VAMP2	13.11804259	-0.214808114	0.368484636	-0.582949989	0.559926968	0.646676216
EHMT1	4.365481714	-0.343037027	0.589058604	-0.582347876	0.560332384	0.647049242
MRPS6	16.60544766	0.187355101	0.321735484	0.582326508	0.560346773	0.647049242
TAF4	41.50934618	0.125705929	0.215956505	0.582089107	0.560506662	0.647177958
HMGB2	106.4220257	0.123032	0.211713758	0.581124255	0.561156713	0.647872559
MICALL1	8.673143341	0.260943073	0.44938938	0.580661414	0.561468672	0.648149614
PITRM1	5.646087346	0.325754813	0.561042303	0.580624333	0.561493669	0.648149614
PDXK	66.39611378	-0.14156216	0.243880306	-0.58045753	0.561606119	0.648223435
KLHDC1	17.58996042	-0.194303883	0.335223631	-0.57962466	0.56216776	0.648759651
RCAN2	1.843603155	0.555861399	0.95895689	0.579652125	0.562149234	0.648759651
ACOT9	26.47554075	-0.205324295	0.35450009	-0.579193917	0.562458335	0.649038946
ERO1A	19.4787846	-0.175171946	0.302616457	-0.578857964	0.562685016	0.649244468
CELF1	252.2532148	-0.073302428	0.126742505	-0.578357105	0.563023048	0.649568205
LOC132657332	2.26669071	-0.48388606	0.836741378	-0.578298232	0.563062788	0.649568205
LOC132658567	20.58527978	0.199221677	0.344723524	0.577917268	0.563319977	0.649752745
LPAR6	4.903871495	-0.355291483	0.614729007	-0.577964403	0.563288153	0.649752745
MRPL49	11.91754144	0.226118241	0.391364309	0.577769195	0.563419956	0.649811988
LOC105607815	7.221543775	-0.257270586	0.445941277	-0.57691584	0.56399631	0.650420593
TAF1A	18.61859988	-0.200611837	0.347835212	-0.5767439	0.564112472	0.650498429
LOC101112109	4.706091124	0.354848327	0.615468298	0.576550129	0.564243397	0.650537155
LOC101113761	2.703810363	-0.51378589	0.891042149	-0.576612331	0.564201368	0.650537155
SAMD13	3.127609987	-0.418568872	0.726145918	-0.576425291	0.564327754	0.650578295
PRIMPOL	13.18947911	-0.212439543	0.368720366	-0.576153537	0.564511407	0.650733891
ZNF175	2.137376736	0.497812503	0.864335615	0.575948155	0.564650225	0.650837781
FKBPL	2.143262035	0.481278589	0.83580805	0.575824304	0.564733944	0.650878149
FAM151B	2.47621449	0.461229272	0.802408922	0.574805763	0.565422669	0.651615744
PDCL	7.79568084	0.303054132	0.528338156	0.573598799	0.566239325	0.652500631
TCTN3	6.829218863	-0.279111031	0.487462734	-0.572579217	0.566929635	0.653239785
SCG3	8.135446515	-0.265973509	0.46469736	-0.572358553	0.567079089	0.653355668
TMEM201	9.309685237	-0.243499851	0.425827292	-0.571827723	0.567438693	0.653713633
LOC121816504	2.791572762	0.459965783	0.805148344	0.571280791	0.56780932	0.653983925
MAPKAPK5	17.61740755	0.198188133	0.346842077	0.57140741	0.567723508	0.653983925

WNT5B	1.848999257	-0.554934703	0.971413991	-0.571264887	0.5678201	0.653983925
TMOD2	5.050975465	0.317711125	0.556234191	0.571182301	0.567876075	0.65399204
SMIM27	8.609223311	-0.260267127	0.455865618	-0.570929495	0.568047436	0.654133026
TST	12.13439095	0.222367807	0.389849953	0.570393315	0.568410962	0.654495255
UACA	87.2439644	0.139040053	0.243922569	0.570017174	0.56866605	0.654676178
USF3	148.0146354	0.081743221	0.143400939	0.570032676	0.568655536	0.654676178
UNK	15.18935182	0.220879683	0.388222201	0.568951704	0.569388916	0.65545192
SDC4	45.03663526	-0.130554956	0.229660393	-0.568469617	0.569716131	0.655772115
ARMH4	3.725108103	-0.406405702	0.715939952	-0.567653336	0.570270383	0.656353565
LOC132657534	4.852073754	-0.343969469	0.606101797	-0.567511052	0.57036702	0.656408265
CCDC61	9.317180744	-0.256394244	0.451964866	-0.567287999	0.570518529	0.656467626
NAGA	48.95123821	-0.164585494	0.290162575	-0.567218201	0.570565944	0.656467626
TRAF3IP2	7.411154336	-0.26239376	0.462500838	-0.567336832	0.570485357	0.656467626
TBC1D24	3.310306087	-0.378045047	0.666793384	-0.566959805	0.570741491	0.65661308
ABHD17C	144.7900079	0.127052138	0.22415734	0.56679892	0.570850805	0.656682318
TTLL12	34.95080012	-0.179103997	0.316044166	-0.566705595	0.570914219	0.656698748
CHCHD10	91.09729187	-0.105797481	0.186720435	-0.566609011	0.570979852	0.656717726
ZNF114	20.05116727	0.162954868	0.287691932	0.566421403	0.571107349	0.656807849
DNAJC16	14.46095357	0.18915504	0.334623553	0.565277125	0.571885285	0.657645937
LOC101118373	43.67912863	-0.139847038	0.24800432	-0.563889524	0.57282932	0.65867487
HACD4	2.356206746	-0.485943701	0.862845739	-0.56318723	0.573307398	0.659121644
SMARCA5	182.9753506	0.082457533	0.146415764	0.563173873	0.573316492	0.659121644
NADK	107.5848134	0.102677037	0.182403363	0.562911971	0.573494828	0.659269964
EFHC2	1.424173984	-0.564358542	1.003677091	-0.562290947	0.573917806	0.659699466
APBA3	3.742652177	-0.366073353	0.651456348	-0.561930748	0.574163203	0.659754588
CARS1	59.92499195	-0.136489278	0.242892438	-0.561933009	0.574161663	0.659754588
LOC101110699	3.756615871	0.368736614	0.656006339	0.562093065	0.574052613	0.659754588
SMDT1	10.72125379	0.213848645	0.380545044	0.561953567	0.574147655	0.659754588
KEAP1	115.054207	0.120748751	0.214930348	0.5618041	0.574249499	0.659797026
LOC105602881	10.34200071	0.241356634	0.429734467	0.561641321	0.574360421	0.659867749
MED30	20.67002582	0.251257738	0.447555464	0.561400224	0.574524731	0.65999979
SERTAD3	21.34645449	0.216870578	0.386359844	0.561317594	0.57458105	0.660007761
BUB3	33.32484744	-0.166513139	0.296816559	-0.560996797	0.57479972	0.660202205
YEATS4	28.2166202	-0.16840777	0.300612647	-0.560215186	0.57533267	0.660757558
TRIB2	31.51919196	-0.132513073	0.236580398	-0.560118563	0.575398569	0.660776465
CCNL2	77.38174514	-0.09580936	0.171092619	-0.55998535	0.57548943	0.660794858
LIN54	14.56926979	-0.241850994	0.431971159	-0.559877641	0.575562901	0.660794858
NFX1	24.94387319	0.151353167	0.270323427	0.559896597	0.57554997	0.660794858
YY2	8.956204043	0.266539758	0.476778054	0.55904368	0.57613191	0.661391317
AFG2B	3.918831489	-0.38134647	0.682589537	-0.558676113	0.576382784	0.661622492
SERAC1	7.200904821	0.252760469	0.452635934	0.558418919	0.576558357	0.661767196
LOC105603195	1.583125836	-0.556220008	0.997433262	-0.557651353	0.577082482	0.662311906

APEH	38.02167891	0.125102035	0.224395892	0.557505908	0.577181824	0.662369044
LOC121816527	6.724239008	0.274536677	0.492862058	0.557025383	0.577510086	0.662688858
LOC105611988	1.174605647	-0.694907963	1.24835444	-0.556659183	0.577760309	0.662735875
LOC114108594	2.071880482	-0.48539533	0.871995655	-0.556648795	0.577767409	0.662735875
NSF	15.53597462	-0.188836922	0.339237756	-0.556650662	0.577766132	0.662735875
TRIM45	2.033720859	-0.511452726	0.918883111	-0.556602597	0.577798979	0.662735875
TWF1	117.7211095	-0.098171115	0.176301533	-0.556836423	0.577639196	0.662735875
ODF2L	26.3330045	0.15391393	0.276657636	0.55633357	0.577982842	0.662889881
LOC106990852	6.164959067	-0.277114829	0.499304324	-0.555001861	0.578893388	0.663877222
GMEB1	22.05415498	0.165374003	0.298214286	0.554547554	0.57920417	0.6641614
TRAPPC1	2.965148082	-0.391015378	0.705174679	-0.554494354	0.579240569	0.6641614
LOC132660057	2.484984072	-0.44256821	0.798330557	-0.554367117	0.579327625	0.664204241
NDUFAF1	6.135394711	-0.304656155	0.550505399	-0.553411748	0.579981497	0.664871698
PSMD10	3.981245566	-0.359863444	0.650311155	-0.553371169	0.580009277	0.664871698
COPS7B	7.626865318	-0.269606942	0.487350173	-0.553209902	0.580119687	0.664941235
C22H10orf62	2.380592985	0.430582264	0.77933391	0.552500357	0.58060559	0.665441117
VPS39	18.00261034	0.170965144	0.309906881	0.551666175	0.581177088	0.666039009
TPP2	47.80869591	-0.116991447	0.212156531	-0.551439291	0.581332572	0.666160079
ABHD10	19.80310959	-0.165082783	0.300198269	-0.549912509	0.582379384	0.66730243
GRPEL2	19.05347813	0.187483893	0.341194262	0.549493101	0.582667098	0.667574869
RUBCN	8.455681678	-0.232321589	0.424542168	-0.547228536	0.584221734	0.669298679
ABCA5	12.31181336	0.211813033	0.38719193	0.547049192	0.584344937	0.66938245
TMEM88	2.528433993	0.468859658	0.858525069	0.546122268	0.584981894	0.670054674
LOC121819276	3.164434216	-0.42733091	0.783623314	-0.545326948	0.585528673	0.6706235
LARP7	204.3482536	-0.129152736	0.237096537	-0.544726371	0.585941725	0.671039079
ARHGEF18	11.89306413	0.205733137	0.377812548	0.544537598	0.586071583	0.671130293
STX6	11.47635632	0.211489984	0.388789107	0.54397096	0.586461456	0.671519218
SLAIN1	4.082330901	0.341069582	0.627128804	0.543858901	0.586538573	0.671549988
CRACDL	13.06474307	0.192489134	0.354167054	0.543498136	0.586786874	0.671776733
AQP7	2.879373866	-0.399458881	0.735109474	-0.543400534	0.586854058	0.671796106
LCMT2	1.319157631	-0.584384322	1.075822566	-0.54319768	0.586993704	0.671840884
LOC114117858	1.378695204	0.585579177	1.077999261	0.543209256	0.586985734	0.671840884
BAHD1	7.940858937	-0.260730515	0.480125029	-0.543047122	0.587097359	0.671901986
SNF8	52.24255936	0.139036781	0.256261974	0.542557209	0.587434708	0.672230506
LOC105607966	3.146249963	-0.381003027	0.702607435	-0.542270133	0.587632428	0.672399198
LOC105616911	5.018210185	0.324100649	0.598108366	0.541876135	0.587903838	0.672594599
TFDP1	11.61873651	0.22820757	0.421122669	0.541902839	0.587885441	0.672594599
PAXX	3.649806765	-0.379821875	0.7020694	-0.541003318	0.588505296	0.673225075
LOC114109259	5.399892394	-0.360677252	0.667488291	-0.54034993	0.588955731	0.673625047
ZNF829	3.132308099	-0.425274247	0.786938028	-0.540416439	0.588909873	0.673625047
SLC12A4	3.936780523	-0.391318727	0.724625903	-0.540028621	0.589177294	0.673820802
GATAD1	32.85568354	0.146992242	0.272508045	0.539405148	0.58960733	0.674226351

TOR1A	6.632923601	0.276320034	0.512303117	0.539368247	0.589632786	0.674226351
GRAMD2A	2.807547513	0.384036516	0.712845663	0.538737255	0.590068164	0.674666473
ARHGAP27	15.95377358	-0.192099673	0.356626146	-0.53865841	0.590122576	0.674670974
PSMB2	37.88792354	0.125029892	0.232296566	0.538233922	0.590415564	0.674948206
LOC132659713	2.5883665	-0.505317125	0.94136276	-0.536793196	0.591410472	0.67602774
ASPM	60.06876188	-0.129868397	0.242501722	-0.535535978	0.592279286	0.676962967
LOC101104041	7.325692473	0.25039322	0.467694553	0.535377669	0.592388729	0.677030162
LOC101121621	53.00417558	-0.129518696	0.242189225	-0.534783065	0.592799875	0.677442127
YTHDF1	9.952345806	0.223089896	0.417506445	0.534338808	0.593107147	0.677735328
KIF14	24.37014981	-0.159863345	0.299243151	-0.534225577	0.593185475	0.677749224
PCBD2	9.181429389	0.247949063	0.464235994	0.534101331	0.593271429	0.677749224
ZBTB14	11.68052606	-0.20468863	0.383217361	-0.534131933	0.593250258	0.677749224
CLTB	5.691912312	-0.291468179	0.546058619	-0.533767199	0.59350261	0.677955379
SNX17	21.46218777	-0.151507003	0.283928801	-0.533609138	0.593611985	0.678022371
CDC42BPB	101.9680998	-0.107097702	0.200759665	-0.533462246	0.593713639	0.678080535
RTN4	117.1387216	-0.089490573	0.167896135	-0.533011512	0.594025612	0.678378873
PDS5A	101.4355796	0.100526202	0.188822249	0.532385367	0.594459118	0.678815939
PRDX1	848.2474254	0.093500333	0.17568057	0.532217839	0.594575129	0.678890413
CACTIN	51.55420941	-0.107293984	0.201794285	-0.531699812	0.594933922	0.679035394
CDADC1	21.43103843	0.15372497	0.289137276	0.531667768	0.59495612	0.679035394
CEP15	2.420659783	-0.419979189	0.789783614	-0.53176488	0.594888849	0.679035394
EED	17.69829097	0.190781785	0.358760283	0.531780674	0.594877909	0.679035394
YAE1	4.549523364	-0.31337111	0.589145259	-0.531908056	0.594789677	0.679035394
RAB6A	163.2747495	0.098364401	0.185212604	0.531089134	0.595357014	0.679434926
ARID4B	307.2897725	-0.073750929	0.13899966	-0.530583521	0.595707418	0.679776773
DPP9	6.019573956	0.281426652	0.530903385	0.530090145	0.596049432	0.680108989
PHTF1	10.19298189	-0.208538014	0.393775646	-0.529585861	0.596399099	0.680449882
ARF4	1.799694863	-0.482946073	0.912122014	-0.529475295	0.596475778	0.680459893
GLIS3	12.33721165	0.261344397	0.493636887	0.529426393	0.596509693	0.680459893
SLC1A1	2.234841759	0.423129513	0.799799491	0.52904449	0.596774588	0.680703971
CASD1	22.25457956	0.171009512	0.323382043	0.528815732	0.596933283	0.680826885
ZNF326	190.1441398	0.080647504	0.152556145	0.528641464	0.597054191	0.680906682
TSSC4	2.573003708	-0.412075029	0.780593147	-0.527899881	0.597568827	0.681435453
LOC101109989	3.602605427	-0.347044091	0.657879845	-0.527518959	0.597833254	0.681678833
JRK	14.71815993	0.189369937	0.359249299	0.52712681	0.59810553	0.68193112
DSCC1	24.86504947	-0.165688219	0.314533452	-0.526774554	0.598350155	0.68215184
RNF139	37.52221171	0.114883876	0.21837493	0.52608546	0.598828831	0.682639331
SUB1	32.77714733	-0.129870692	0.247000918	-0.525790321	0.599033901	0.682814866
WSB2	32.37168115	0.130193977	0.247858232	0.525275983	0.599391351	0.683164048
LOC101120446	10.65189848	-0.212226211	0.404340804	-0.524869636	0.599673819	0.683427717
FRS2	20.8433943	-0.177065077	0.33760294	-0.524477295	0.599946608	0.683622026
MAP2K6	5.379368509	0.282286055	0.538219203	0.524481575	0.599943632	0.683622026

POLR1E	28.4994753	-0.147354499	0.281193544	-0.524032299	0.600256075	0.683909364
ZNF319	1.783473063	-0.495413988	0.945505098	-0.523967548	0.600301111	0.683909364
HERC6	4.103125511	0.314608082	0.601086452	0.523399057	0.60069658	0.684235864
NDUFAF6	2.933716818	-0.397339512	0.759141096	-0.523406669	0.600691284	0.684235864
STC1	14.58950349	-0.173154981	0.33086845	-0.523334821	0.600741273	0.684235864
SH2D4A	22.88987654	0.148929922	0.284727979	0.523060369	0.600932243	0.684395055
FASTKD5	10.69267393	-0.219874842	0.421671833	-0.521435925	0.602063131	0.685590157
SLC10A3	5.678191253	-0.269723174	0.51730002	-0.521405691	0.602084188	0.685590157
PRDM11	2.865312469	0.41027279	0.787042177	0.521284375	0.602168684	0.68562796
FUT10	13.49920275	0.182768049	0.350936839	0.520800409	0.602505818	0.685953387
ATPAF2	8.659077487	-0.270316528	0.519453891	-0.520385992	0.602794572	0.686106808
GARRE1	23.98443746	-0.180207938	0.346259574	-0.520441749	0.602755718	0.686106808
MED28	29.18536211	0.137813837	0.264760074	0.520523489	0.602698761	0.686106808
LOC114110979	1.848599351	0.493930976	0.951417389	0.519152773	0.60365421	0.686992913
RAD17	33.75437208	0.127982849	0.246537275	0.519121701	0.603675876	0.686992913
TRIM38	21.53294981	0.154545108	0.29794045	0.518711401	0.60396201	0.687260023
JPT1	74.71442313	0.096444161	0.18599363	0.518534754	0.604085219	0.687341707
DCAKD	2.464086057	0.433882722	0.83781563	0.51787375	0.604546358	0.68780785
CAPN7	27.72044162	-0.145658203	0.281458417	-0.517512338	0.604798558	0.688036218
LOC132659222	12.17436626	-0.197664463	0.382091408	-0.517322449	0.604931086	0.688128416
LOC132659908	5.956134339	0.259792781	0.502469915	0.517031515	0.605134159	0.68830084
GLB1L	4.433171818	-0.323565249	0.626195277	-0.516716207	0.605354281	0.688434044
LOC114114483	9.486737249	-0.266928085	0.516570313	-0.51673137	0.605343694	0.688434044
UBQLN2	12.99670502	0.19008182	0.368108364	0.516374629	0.605592782	0.688646684
FAM228A	11.12110324	0.197916915	0.383454185	0.516142275	0.605755044	0.6887726
LOC121819403	2.426633317	-0.402206853	0.782690535	-0.513877242	0.60733782	0.690396096
MPLKIP	84.40038791	0.096757076	0.188267477	0.513934098	0.607298067	0.690396096
TCTA	10.78055369	-0.222524275	0.432989346	-0.513925522	0.607304063	0.690396096
LOC106990496	1.932642951	0.465297352	0.90617919	0.513471681	0.607621415	0.690659736
ALDH3A2	10.68663757	-0.218555619	0.426173326	-0.512832704	0.608068351	0.690906101
ANXA10	27.10645329	-0.423795407	0.82644651	-0.5127923	0.608096616	0.690906101
FANCI	29.43774505	0.164298117	0.320303827	0.512944595	0.607990078	0.690906101
PDK3	11.7186681	-0.201554492	0.393038064	-0.512811634	0.608083091	0.690906101
SYTL1	2.178397361	-0.428109717	0.834404931	-0.513071892	0.607901032	0.690906101
TTC13	3.708804382	-0.337886929	0.659040326	-0.512695378	0.608164423	0.69092441
LOC101111335	38.7731633	0.123627169	0.241260794	0.512421295	0.608356191	0.690966081
RASIP1	7.206316672	0.252327793	0.49231071	0.512537688	0.608274751	0.690966081
TFB2M	4.470923843	0.298974359	0.583392055	0.512475883	0.608317995	0.690966081
ITGB1BP2	6.581116253	0.28993407	0.565980843	0.512268346	0.608463216	0.691028918
PAQR4	1.868123265	0.448185332	0.875036631	0.512190366	0.608517785	0.691032176
LOC114115633	1.974500311	0.46930529	0.916912983	0.511831874	0.608768681	0.691258363
PGRMC2	132.1546838	0.095748729	0.187117993	0.511702414	0.608859296	0.691302528

GDPD5	49.83299634	-0.117634386	0.229956355	-0.511550924	0.60896534	0.691305481
PHAF1	6.619558384	-0.25303143	0.494634324	-0.511552511	0.608964229	0.691305481
LOC105607070	1.853650595	0.470374469	0.919948145	0.511305416	0.609137213	0.691441868
LOC114113403	3.266781164	-0.339164561	0.663622526	-0.511080543	0.60929466	0.691561856
PRKD2	15.40251301	0.175506636	0.343492171	0.510947993	0.609387474	0.691608472
ECI1	29.02712734	-0.141075446	0.276304197	-0.510580179	0.609645058	0.691748685
LOC101118415	15.64167075	-0.191797967	0.375669402	-0.510549877	0.609666281	0.691748685
NHS	2.79472065	-0.433166312	0.848340881	-0.510604076	0.609628321	0.691748685
ANAPC13	15.43006703	-0.172397483	0.337736667	-0.510449411	0.609736648	0.691767484
PDSS2	43.52141688	-0.12749945	0.249813551	-0.510378437	0.60978636	0.691767484
CSNK1G1	113.0824347	-0.092728976	0.181987619	-0.509534531	0.610377601	0.69232069
DSC2	67.73506867	-0.110791406	0.217436269	-0.509535079	0.610377217	0.69232069
UBALD1	3.98167839	0.327133894	0.642390855	0.509244319	0.610580981	0.692492609
SETDB1	11.92039417	-0.188325756	0.369924472	-0.509092451	0.610687423	0.692554564
IREB2	91.97038337	0.100834906	0.198152435	0.508875429	0.610839544	0.692668307
C18H15orf39	20.71594699	0.155808525	0.306327145	0.508634403	0.61100851	0.692801132
BPHL	7.697990071	0.276569573	0.5439669	0.508430886	0.611151197	0.692904139
FHL2	72.65647591	0.094071572	0.18513507	0.508123998	0.611366387	0.693056719
USP53	32.18987839	0.134734524	0.265177903	0.508091069	0.611389479	0.693056719
LOC101104797	38.04422267	-0.118614218	0.233690207	-0.507570341	0.611754695	0.693411911
RAD9A	6.999735141	0.232668359	0.459342725	0.506524532	0.612488472	0.694125905
STRIP1	84.70115877	-0.091110422	0.179861919	-0.506557598	0.612465266	0.694125905
IFI6	39.9040427	0.120963754	0.238866438	0.506407492	0.612570616	0.69416014
UBE2B	45.87743184	0.163932556	0.324366906	0.505392358	0.613283285	0.694908815
LOC132657809	1.246923325	0.543759266	1.076582689	0.505078961	0.613503378	0.695099275
DRG2	4.069685736	0.319397446	0.633327147	0.504316683	0.614038856	0.695529097
MTF2	74.23145392	-0.10446039	0.20710232	-0.504390243	0.613987173	0.695529097
POLR3A	4.394784113	0.322731035	0.639917098	0.504332571	0.614027693	0.695529097
DCBLD2	36.07201852	0.125094711	0.248296843	0.503811122	0.614394111	0.695813565
TNF	2.210142197	0.451768142	0.896608904	0.5038631	0.614357582	0.695813565
LOC105604110	3.584000665	0.328095848	0.65160781	0.503517365	0.614600575	0.695935867
SEC31B	3.640822116	-0.313661126	0.622950005	-0.503509308	0.614606237	0.695935867
NT5DC2	1.618300088	-0.496822935	0.986956733	-0.503388769	0.614690966	0.695972847
FBXO21	27.47385143	-0.131146858	0.260669323	-0.503115812	0.614882851	0.696092078
LOC105615606	2.072443556	0.425046144	0.844994052	0.503016729	0.61495251	0.696092078
NUDT18	9.957529093	-0.211513652	0.420476884	-0.503032772	0.614941231	0.696092078
ZBTB43	14.71055395	0.176539489	0.351115304	0.502796338	0.615107469	0.696208521
LOC101109728	14.72213276	-0.16798989	0.334183076	-0.502688205	0.615183504	0.696235623
LOC121819797	1.784206574	-0.473888026	0.943299911	-0.502372596	0.615405452	0.696427844
FARP2	2.039883547	0.447198074	0.890673545	0.502089769	0.615604377	0.696593981
FKBP8	2.238708133	-0.428585796	0.853981964	-0.501867503	0.615760726	0.696623008
GTF3A	2.238335883	-0.41001769	0.817389995	-0.50161819	0.615936122	0.696623008

KATNIP	12.60788504	0.193446393	0.385379672	0.501963146	0.615693445	0.696623008
LOC114110441	1.870056453	-0.4914616	0.979770732	-0.501608779	0.615942743	0.696623008
POLR3K	5.998478673	-0.282151809	0.562412922	-0.50168088	0.615892016	0.696623008
SENP6	169.9296038	0.078685845	0.156853752	0.501651018	0.615913025	0.696623008
EPB41L4A	21.13647815	0.168830466	0.336895283	0.50113633	0.61627518	0.696940017
HTR4	28.81877703	0.128309409	0.25639954	0.500427609	0.616774017	0.697386136
SMC4	284.1134274	0.110260229	0.220326395	0.500440401	0.616765012	0.697386136
MTX1	11.54662102	-0.196976162	0.394164109	-0.49973135	0.617264255	0.69788141
UBALD2	28.94447653	-0.132955373	0.266663398	-0.498588759	0.618069126	0.698732296
MACROD2	1.620738204	0.535932216	1.076174345	0.497997577	0.61848575	0.699144159
DAD1	78.65480049	0.124666316	0.250409906	0.49784898	0.61859049	0.6991443
TRA2A	206.6152699	-0.095384818	0.191574732	-0.497898741	0.618555415	0.6991443
ERAL1	6.367674959	0.245651226	0.493685167	0.497586809	0.618775303	0.699229237
GDAP2	19.34116512	-0.148474884	0.298395288	-0.497577844	0.618781623	0.699229237
REEP3	88.34835326	0.084753494	0.170352021	0.497519745	0.618822583	0.699229237
BMP2	34.64473895	0.119770637	0.240973659	0.497027922	0.619169361	0.699561934
RELT	3.236838084	-0.365052057	0.73476798	-0.4968263	0.619311547	0.699663438
PABPC5	13.56340596	0.179672759	0.361903744	0.496465598	0.619565953	0.699891695
HJURP	2.147130624	-0.463911642	0.934833369	-0.496250623	0.619717598	0.700003838
APIP	24.28279794	-0.129805047	0.261718861	-0.495971311	0.619914652	0.70010809
EEA1	125.5846145	0.085284031	0.171936666	0.496020033	0.619880277	0.70010809
SCYL1	14.94756482	-0.169010881	0.34094862	-0.495707772	0.620100603	0.700258927
ENPP3	8.977401264	0.212708353	0.429528516	0.495213577	0.620449368	0.700593584
ARL1	65.55678562	0.102970108	0.208151453	0.494688395	0.620820095	0.700949288
LOC105611653	1.909038329	0.426734116	0.862883205	0.494544468	0.62092171	0.700949288
ZNF502	4.602258731	-0.293515436	0.593478373	-0.494568041	0.620905066	0.700949288
AARSD1	13.2540475	-0.182517508	0.369339145	-0.494173203	0.621183863	0.701186007
PFKFB2	6.840978502	0.233030387	0.471683553	0.49403967	0.621278163	0.701233232
ANKS1A	19.67971653	-0.146410367	0.296521098	-0.493760371	0.621475424	0.701396649
LOC132657219	2.102313214	-0.43545841	0.88275208	-0.493296385	0.621803183	0.701707306
ANAPC15	4.154483117	-0.29886091	0.606084791	-0.493100824	0.621941349	0.701803973
TFDP2	166.696745	0.103026438	0.208981497	0.49299311	0.622017456	0.701830602
TRMT61B	1.749306495	-0.472930427	0.959971139	-0.492650672	0.622259437	0.702044369
AGTRAP	2.272069822	0.447239511	0.908962261	0.492033092	0.62269595	0.702477555
TPRN	3.737024663	0.326048568	0.662836895	0.49189864	0.622791	0.702525487
DDHD2	7.508183294	-0.232708225	0.47400193	-0.490943624	0.623466322	0.703168581
LOC132658793	1.374839861	0.551952024	1.12417547	0.490983871	0.623437855	0.703168581
RCOR3	52.47259114	0.113650736	0.231706488	0.490494406	0.623784087	0.70346761
GLMN	14.92325884	-0.165083425	0.336932088	-0.489960531	0.624161828	0.703774844
IDH1	38.66763252	-0.185857441	0.379282182	-0.490024181	0.624116788	0.703774844
RAD54L2	170.50094	-0.079626078	0.162624866	-0.489630396	0.624395463	0.703978892
LOC105611516	4.21127361	0.302776352	0.618569673	0.48947817	0.624503206	0.704040979

ACADSB	12.72721538	0.182829807	0.374006683	0.488841017	0.624954257	0.704426677
EIF3A	770.7509324	0.081243765	0.166220322	0.488771555	0.625003439	0.704426677
GPD1	46.4939427	0.260183388	0.532270361	0.488818102	0.624970481	0.704426677
ZNF471	4.643650131	0.32527868	0.666106104	0.488328628	0.625317087	0.704720757
TDRD3	26.47924175	0.131836141	0.270123634	0.48805852	0.625508391	0.704876921
LOC132658203	2.621669192	-0.381983252	0.783398232	-0.487597797	0.625834756	0.705044159
RAPGEF2	17.56056878	-0.161521595	0.331291581	-0.487551163	0.625867795	0.705044159
TRIM68	24.77268606	-0.136306845	0.27950121	-0.487678907	0.625777294	0.705044159
ZNF789	8.733811077	0.216679224	0.444378573	0.487600521	0.625832826	0.705044159
AMBRA1	26.94454149	-0.150893754	0.309700916	-0.487224111	0.626099521	0.705182698
EVI5	87.79884453	-0.076467744	0.156954338	-0.487197393	0.626118453	0.705182698
STARD5	2.977551616	0.348723415	0.71583784	0.487154207	0.626149054	0.705182698
HSPB2	2.898254616	0.344897927	0.709216043	0.486308693	0.626748317	0.705738669
RPN1	259.0158416	0.07029898	0.144541024	0.486360055	0.626711906	0.705738669
HSPBAP1	2.56484785	0.378010386	0.778390603	0.485630716	0.627229014	0.706220454
ZFYVE19	5.296841999	-0.314678565	0.648465689	-0.48526633	0.627487436	0.706451909
LAMTOR3	52.62453531	-0.104348612	0.215309504	-0.484644709	0.627928394	0.706888816
NUP42	11.86857812	0.178821344	0.369043386	0.484553715	0.627992953	0.706901954
CS	75.82981411	-0.0848295	0.175297273	-0.483917968	0.628444088	0.707350205
SERF2	137.3047642	0.099057095	0.204745621	0.483805681	0.628523783	0.707380337
POP1	5.998703333	0.255767779	0.528939995	0.483547816	0.628706817	0.707468041
TMEM144	16.68210745	0.159435829	0.32972164	0.483546755	0.62870757	0.707468041
UCK2	6.507622229	0.259007825	0.536253245	0.482995352	0.629099036	0.707848954
PHKB	11.43969951	-0.181635964	0.376462924	-0.482480353	0.629464752	0.708200831
PARP16	11.30041348	0.179350906	0.371820531	0.482358803	0.629551082	0.708238343
NSL1	4.205775304	0.297815591	0.617775626	0.482077276	0.629751052	0.708344068
NTHL1	8.144627965	0.229500044	0.476011752	0.482131045	0.629712857	0.708344068
ACP1	94.44344776	-0.095872499	0.19906551	-0.481612806	0.630081027	0.708423144
ATF1	18.7606086	0.159346655	0.330745895	0.481779691	0.629962458	0.708423144
FAN1	7.610997327	0.227413177	0.472187507	0.481616252	0.630078579	0.708423144
MCOLN2	8.006615712	-0.249007774	0.516888006	-0.481744152	0.629987707	0.708423144
XPO1	30.2287324	0.124122485	0.257726577	0.481605296	0.630086363	0.708423144
LIAS	4.408398525	-0.278637895	0.578745261	-0.48145171	0.630195493	0.708463543
NCOA5	6.217510057	-0.247589855	0.514306211	-0.481405533	0.630228305	0.708463543
CCDC117	76.49471059	0.09029543	0.187680217	0.481113198	0.630436048	0.708577886
PARP11	13.97150652	0.167231937	0.347588114	0.481120989	0.630430512	0.708577886
DLG2	8.890959248	0.211722344	0.440290395	0.480869777	0.630609054	0.70871274
SPAG8	12.47281272	-0.203880052	0.424080026	-0.48075844	0.630688191	0.708742085
KPTN	2.242898407	0.413407348	0.860359029	0.480505619	0.630867908	0.708884444
CSRNP1	2.004127894	-0.418138799	0.870539206	-0.480321617	0.63099872	0.70897183
GNPDA1	91.46125067	-0.0951405	0.198198172	-0.480027133	0.6312081	0.709147471
TBC1D13	6.252507461	0.246352839	0.513530229	0.479724124	0.631423572	0.709329926

SEC61G	245.3824681	-0.197718209	0.412407268	-0.479424648	0.631636562	0.709509563
DHODH	15.44088088	-0.15914555	0.332010656	-0.47933868	0.631697709	0.70951862
TWIST2	2.331143918	-0.378454701	0.79012397	-0.47898142	0.631951847	0.709744424
EFHB	6.559330795	-0.230164012	0.480682796	-0.478827232	0.632061542	0.709748347
IMMP1L	24.10879013	0.125320572	0.2616976	0.478875511	0.632027194	0.709748347
AQR	27.77588885	0.124519009	0.260577073	0.477858654	0.632750811	0.710343301
HOXC6	10.13105824	-0.20920452	0.437678094	-0.477987184	0.632659327	0.710343301
TMEM128	40.91220027	0.193638248	0.405217948	0.477861972	0.632748449	0.710343301
RANGRF	5.068323331	-0.264564637	0.554853397	-0.476818992	0.633491021	0.711109754
SP2	4.11164117	-0.306015821	0.641878627	-0.476750289	0.633539948	0.711109754
CDHR2	14.3584572	-0.203244218	0.426574008	-0.476457107	0.633748759	0.711284399
PLPP6	20.83912244	0.133390055	0.280455261	0.475619728	0.634345318	0.711894166
ATP23	6.069652454	0.284715855	0.599023659	0.475299849	0.634573267	0.711970644
KDELR2	137.1164955	0.103812772	0.218402496	0.475327775	0.634553365	0.711970644
LOC101114548	18.42710728	0.154451554	0.324871183	0.475423991	0.634484798	0.711970644
LMTK2	12.86404677	-0.177945793	0.374509441	-0.475143678	0.634684568	0.712035751
ITFG1	26.65499578	0.134867867	0.283937859	0.474990786	0.634793541	0.712098234
PCTP	37.59988777	-0.117579888	0.247594574	-0.474888789	0.634866242	0.712120023
LOC101121607	48.30468715	0.092982076	0.195972026	0.474466063	0.635167593	0.712398259
ZNF268	1.672142839	-0.475558846	1.003448006	-0.473924751	0.635553569	0.712771354
EMC9	11.4034129	0.188144126	0.398328982	0.472333508	0.636688756	0.713984555
TATDN3	4.383193943	-0.284928589	0.603891593	-0.471820757	0.637054734	0.714335031
CAMKK1	2.821627374	-0.373457783	0.792340083	-0.471335214	0.637401372	0.714663765
LOC101105010	10.63309587	0.187013934	0.397105875	0.47094225	0.637681976	0.71491841
VSIG8	7.944058327	-0.210936472	0.447978261	-0.470863187	0.637738439	0.714921745
RTL8C	9.319407242	-0.189825746	0.403478426	-0.470473099	0.638017049	0.715127906
ZNF518A	42.16504519	-0.102816403	0.218546323	-0.47045588	0.638029349	0.715127906
EIF4ENIF1	46.20516357	-0.11373984	0.242315698	-0.469387006	0.638793034	0.715923839
CREB5	7.519114142	0.227584588	0.4850486	0.469199557	0.638927002	0.716013944
DENND1C	2.597859226	0.382531369	0.816236241	0.468652763	0.639317857	0.716391891
NLN	10.58768702	0.208919103	0.445957118	0.468473525	0.639446	0.716475415
GPRASP2	24.80445465	0.126402219	0.269940543	0.468259483	0.639599039	0.71658682
ACP3	7.648394816	-0.226836989	0.48494901	-0.467754308	0.639960299	0.716871384
ACTR8	18.37647775	-0.13899455	0.297121061	-0.467804435	0.639924449	0.716871384
LOC121818846	5.382396588	0.306611304	0.657580011	0.466272239	0.641020647	0.717998992
USP12	67.82540547	0.099198141	0.212784856	0.466189856	0.64107961	0.718004865
USP21	2.698221673	-0.374118671	0.80285806	-0.465983577	0.641227256	0.718110055
ZFPM1	12.35817071	-0.166170897	0.356943557	-0.465538299	0.641546017	0.718406841
OSBPL2	24.42690799	0.131762824	0.283414391	0.464912255	0.641994294	0.718848599
INTS2	8.950157337	0.191634381	0.413143775	0.46384429	0.642759309	0.719644909
LOC121817248	10.53304944	0.186710886	0.403294848	0.462963728	0.643390367	0.720291117
EXD3	3.050751607	-0.319874553	0.691056695	-0.462877438	0.64345222	0.720300031

LOC121819875	1.920875185	-0.415615856	0.898099265	-0.462772738	0.643527274	0.720323721
DHX40	90.12576677	-0.08718673	0.188575804	-0.462343145	0.643835264	0.720608118
FLAD1	3.358867517	-0.310207456	0.672486059	-0.46128459	0.644594439	0.72139741
LOC101110171	20.42028445	-0.132114019	0.286602055	-0.460966754	0.644822458	0.721592178
ERCC2	4.174114594	-0.269091838	0.583860285	-0.460883956	0.644881863	0.72159824
LOC101109384	8.713411652	-0.204383981	0.443582053	-0.460758003	0.644972236	0.72163895
METTL5	3.993085095	0.287132505	0.624495698	0.459783	0.645671987	0.722361411
DCAF15	1.836566385	-0.422999356	0.920678985	-0.459442827	0.6459162	0.722453215
PIP4K2C	13.9570002	-0.181111624	0.394144755	-0.45950535	0.645871311	0.722453215
STING1	2.273965875	0.395154537	0.859864518	0.459554416	0.645836085	0.722453215
ACADVL	26.77349912	0.150242475	0.327164323	0.459226341	0.646071637	0.722566605
CAMK1D	3.655868418	0.296515374	0.645892808	0.459078303	0.646177937	0.722625025
SPAG5	3.76383043	-0.312698257	0.681550413	-0.458804296	0.64637471	0.722784604
FAM83G	8.024278464	-0.207763938	0.452971301	-0.458669097	0.646471809	0.722832708
PGAP6	15.45002196	0.158568075	0.346333037	0.457848538	0.647061261	0.723370761
SLC6A14	2.504594526	0.385018161	0.840893467	0.457867942	0.64704732	0.723370761
LENG9	8.333818985	-0.225002009	0.491534736	-0.457754036	0.647129161	0.723386165
GDPGP1	3.201502598	0.325489111	0.711260743	0.457622769	0.647223483	0.723431098
BZW1	205.5835085	-0.068047904	0.149012988	-0.456657535	0.647917218	0.72414596
LOC114118420	3.717619999	0.309297892	0.677989635	0.456198556	0.648247204	0.724454191
TCIM	7.835517387	-0.214130337	0.469733423	-0.455855016	0.648494239	0.724669676
LOC114116612	2.866080806	0.327251966	0.718044644	0.455754345	0.648566638	0.724689992
MRPS34	14.94288764	0.155899839	0.342746736	0.454854335	0.649214037	0.72535274
ANAPC7	41.85686674	-0.095471075	0.209981716	-0.454663753	0.649351162	0.725445305
SCAF1	7.182028803	0.242061313	0.532832768	0.454291341	0.649619147	0.725684039
LOC105609489	2.306170235	-0.367977953	0.810272981	-0.454140718	0.649727548	0.725744477
CD151	46.44272382	-0.102336331	0.225680603	-0.453456474	0.650220079	0.726233941
MOSMO	15.53423465	-0.149010564	0.328759263	-0.4532513	0.650367796	0.726338232
EPS15L1	4.191147359	-0.286994659	0.635104619	-0.451885642	0.651351368	0.72737592
AGA	4.628485646	-0.259950481	0.575707255	-0.451532405	0.651605875	0.727599343
CCDC126	7.50607583	-0.20807816	0.460952302	-0.45140931	0.651694575	0.727637598
CPT2	5.919647047	0.241482634	0.535177783	0.451219468	0.65183138	0.727729554
CARS2	6.440901587	0.227149777	0.503607971	0.45104484	0.651957232	0.727748485
CYB561	30.84785114	0.109138753	0.241940007	0.451098412	0.651918622	0.727748485
ECE2	3.168416085	-0.318841509	0.707170153	-0.450869579	0.65208355	0.727828704
DHX35	4.921539971	-0.26807828	0.595209441	-0.450393191	0.652426954	0.728151191
FAM98C	2.483880363	-0.415514822	0.924538551	-0.449429417	0.653121915	0.728744262
LOC132659681	7.626043828	0.205919386	0.458118219	0.449489624	0.653078491	0.728744262
LUZP1	81.91061151	0.098950773	0.220109467	0.449552556	0.653033104	0.728744262
RNF26	16.35984937	-0.141496701	0.314936227	-0.449286838	0.653224751	0.728798166
MYNN	24.18209252	-0.122885778	0.2736643	-0.449038396	0.653403959	0.728937261
APOO	10.82813392	0.17042793	0.379730594	0.448812744	0.653566744	0.729058012

LTA4H	36.06071381	-0.129227227	0.288323665	-0.448201943	0.65400746	0.729488752
NDUFAF4	22.9106068	-0.120789657	0.269714121	-0.447843282	0.654266305	0.729716574
AKIP1	3.878004849	0.297803383	0.665388041	0.447563474	0.654468269	0.729880925
CFP	30.1992067	0.153962722	0.344393373	0.447054833	0.65483547	0.730229508
BTBD10	16.27556991	-0.152961001	0.342890089	-0.446093387	0.65552979	0.730942785
DOCK7	15.13011267	-0.153711725	0.344867939	-0.445711844	0.655805408	0.731128124
SELENOI	35.79007425	0.107768613	0.241769509	0.445749399	0.655778277	0.731128124
PGBD1	12.65189505	0.184222858	0.413919315	0.445069488	0.656269538	0.731523529
TAF2	56.94718576	-0.088518581	0.198861345	-0.445127139	0.656227877	0.731523529
DDRGK1	18.05936824	0.154324494	0.347230608	0.444443809	0.656721746	0.731966549
LOC101109890	2.989649668	-0.332175809	0.747932825	-0.444125191	0.656952074	0.732162213
TMEM184B	24.6472011	-0.121333978	0.273400224	-0.443796191	0.657189943	0.732366248
MRPS24	16.83300106	-0.153582727	0.346184362	-0.443644324	0.657299754	0.732366498
SMYD2	3.275869474	-0.305263138	0.687968976	-0.443716431	0.657247615	0.732366498
RFNG	7.307448676	-0.210670419	0.475506069	-0.443044648	0.657733441	0.732788628
STX2	22.04907803	-0.120716672	0.272895656	-0.442354686	0.658232564	0.733283585
KANK1	10.48064376	0.172423502	0.390002761	0.442108414	0.658410756	0.733420965
SCG5	13.82785438	0.165392925	0.374240898	0.441942413	0.658530879	0.733493644
LOC101108819	1.513207853	0.441180989	1.003337828	0.439713302	0.660144767	0.735022136
LOC101110202	13.60409858	-0.149371363	0.339684701	-0.439735325	0.660128814	0.735022136
LRRC73	5.09894423	0.244768596	0.556731119	0.43965316	0.660188332	0.735022136
RMI1	7.194963484	-0.240020268	0.545590366	-0.439927614	0.659989535	0.735022136
RNF7	28.06621162	-0.121298745	0.275935245	-0.439591344	0.660233111	0.735022136
USP54	1.886447625	0.391742389	0.890659921	0.439833858	0.660057444	0.735022136
AKAP8	27.02592699	-0.120660027	0.274572858	-0.439446303	0.660338182	0.735077883
PROSER1	13.24341788	-0.187822619	0.427659835	-0.439186951	0.66052608	0.735225815
CRYGS	5.347955895	0.254673586	0.580278898	0.43888135	0.660747511	0.735277281
LOC121820643	3.835932326	0.290095563	0.661081816	0.438819456	0.660792361	0.735277281
SMCR8	24.94680996	-0.121760744	0.277366778	-0.438988204	0.660670083	0.735277281
WDSUB1	3.737337431	0.281379241	0.641002691	0.438967331	0.660685208	0.735277281
SRI	15.77984398	0.16414926	0.374558204	0.438247668	0.661206757	0.735637038
TXNDC2	1.617325764	-0.448143339	1.022641233	-0.438221465	0.66122575	0.735637038
UBOX5	8.792529986	0.195614445	0.446541768	0.43806528	0.661338962	0.735701754
HNRNPA1	232.7726762	-0.064200115	0.147101239	-0.4364349	0.662521223	0.736955614
GTPBP8	18.27182357	0.149526766	0.342735726	0.436274231	0.662637776	0.737023925
DNAJC14	21.04256118	0.124901517	0.286453778	0.436026775	0.662817303	0.737124217
LOC121819116	3.591623297	0.284557114	0.652657055	0.435997913	0.662838244	0.737124217
SLC29A3	8.734717747	0.22007368	0.504905597	0.435870946	0.662930366	0.737165331
RAD18	13.75621404	0.156228041	0.3585506	0.435721041	0.663039138	0.73722495
RICTOR	23.39536478	0.120366368	0.276487276	0.435341437	0.663314612	0.737447552
SPAG6	3.261699214	-0.300799843	0.69102827	-0.435293108	0.663349687	0.737447552
TAF6	13.57157972	0.146950384	0.338871334	0.433646547	0.664545128	0.738715085

KDM7A	29.09213967	0.105786532	0.244035549	0.433488203	0.664660135	0.738781485
CLTC	527.2147354	-0.074262682	0.171615974	-0.432725928	0.665213891	0.739335512
EHMT2	27.31563894	-0.137885973	0.318727942	-0.432613382	0.665295666	0.739364918
TSPAN7	15.73942092	0.142500448	0.329577761	0.432372767	0.665470508	0.73949774
ARHGEF26	13.68570581	0.155122231	0.358903788	0.432211182	0.665587933	0.739505263
CD82	93.87927932	0.073665239	0.170426876	0.432239566	0.665567306	0.739505263
ARID5B	86.27594758	0.085321841	0.197616309	0.43175506	0.665919445	0.739812094
RRAGC	2.503152487	0.365339334	0.846645943	0.431513713	0.666094884	0.739945497
SETD5	332.1322913	-0.055624584	0.128974113	-0.431284869	0.666261251	0.740068801
ZNF398	7.652598125	-0.202164939	0.469301724	-0.430778173	0.666629672	0.740416502
GPN3	7.58880434	-0.201567072	0.468707975	-0.43004831	0.6671605	0.740899213
TAX1BP3	53.95286997	0.104186618	0.242278581	0.430028183	0.66717514	0.740899213
FAM180B	2.336986378	0.346037792	0.805602136	0.429539317	0.667530787	0.741171
NEK4	18.66156511	-0.137655727	0.320452881	-0.429566203	0.667511227	0.741171
SLC8B1	2.870113732	0.315835197	0.73555045	0.429386178	0.667642211	0.741171577
TMEM54	29.22587932	0.165558386	0.385534633	0.429425458	0.667613631	0.741171577
DDO	17.31740425	0.148450357	0.346111361	0.428909229	0.667989284	0.741495288
CABIN1	10.68658656	0.165808922	0.387152751	0.428277783	0.668448892	0.741943854
ERAS	3.676590574	-0.275444871	0.643945616	-0.427745549	0.668836384	0.742312307
CLCC1	28.95046668	0.108433847	0.253799585	0.427242017	0.669203061	0.742657598
GOLM1	498.6796875	-0.072735096	0.170320822	-0.427047589	0.669344666	0.742753076
ACKR2	2.715427641	0.304987101	0.715566218	0.426217858	0.669949106	0.743362091
DBP	2.160211516	0.4123698	0.968176712	0.425924105	0.670163149	0.74347615
GGA2	16.31662875	-0.135455151	0.318026309	-0.425924356	0.670162967	0.74347615
LOC132657792	3.536743022	0.281521796	0.661198413	0.425775062	0.67027176	0.743534928
TMEM216	2.207712026	-0.36023858	0.846689605	-0.42546711	0.670496193	0.743722167
TRIM24	39.3124056	-0.095938029	0.225744132	-0.424985705	0.670847096	0.744049646
GABPA	9.311171681	-0.181484952	0.427487605	-0.424538514	0.671173124	0.744349483
ABHD13	113.0954207	-0.07739402	0.182388319	-0.424336494	0.671320428	0.744430914
ZNF354C	8.770033085	0.182456317	0.430032397	0.42428505	0.671357941	0.744430914
AP4B1	18.26874513	0.122582977	0.289053201	0.424084483	0.671504202	0.744531328
IL10RB	28.67147172	0.126226249	0.297767193	0.423909188	0.671632043	0.744549547
RHNO1	65.39660347	-0.078254653	0.18458032	-0.423959894	0.671595063	0.744549547
ACAA2	32.31160195	0.111094748	0.262143337	0.423793905	0.671716124	0.744581001
LOC114109664	4.920897645	-0.242743352	0.573334415	-0.423388769	0.672011638	0.744723286
MRRF	18.91951545	0.125282679	0.295891219	0.423407897	0.671997684	0.744723286
OSGEP	17.09040094	0.144969282	0.342366261	0.423433318	0.671979141	0.744723286
DCTD	2.473489334	0.356358543	0.842723751	0.422865194	0.672393619	0.745084821
MAN1B1	43.11183705	0.157553604	0.37267727	0.422761507	0.672469275	0.745106883
LOC121816188	2.370741316	0.374432588	0.886673453	0.42228916	0.672813969	0.745303457
MED27	20.71791405	0.133875844	0.317013856	0.422302816	0.672804003	0.745303457
PRKACA	3.284197953	0.304979542	0.722137694	0.422328795	0.672785043	0.745303457

C21H11orf68	6.309585437	-0.246576043	0.584172531	-0.422094552	0.672956004	0.745306071
KRBA1	1.993167599	0.36387765	0.862093359	0.422086131	0.672962151	0.745306071
NUBP2	8.42137793	-0.194533409	0.460917685	-0.422056726	0.672983613	0.745306071
C2H9orf152	34.78651517	-0.099773164	0.236612832	-0.421672665	0.673263959	0.74555477
LIN37	3.387472539	-0.29791693	0.706732259	-0.421541435	0.673359762	0.745599086
BIN1	4.593271686	-0.25319619	0.601604078	-0.420868473	0.673851128	0.745785586
LOC105603227	2.496129857	0.324835724	0.771654968	0.420959803	0.673784434	0.745785586
PPEF2	3.504522741	0.271098552	0.643626316	0.42120489	0.673605474	0.745785586
PPP1R9B	7.885441452	0.198581667	0.471655595	0.421031086	0.673732383	0.745785586
TRPM6	1.097949123	0.519579836	1.23458966	0.42085225	0.673862975	0.745785586
YES1	11.20476413	-0.166781836	0.396181053	-0.420973783	0.673774226	0.745785586
SERGEF	42.89516086	-0.094226495	0.224189962	-0.420297564	0.674268089	0.746162104
ZCCHC7	9.215017472	0.17524768	0.417024464	0.420233571	0.674314832	0.746162104
MMAA	12.55511754	-0.153548291	0.365890013	-0.41965696	0.674736071	0.746566418
PPP1R8	24.72936189	-0.120070164	0.286563226	-0.419000602	0.675215692	0.747035257
DNAJB12	33.88397092	-0.102222286	0.244339145	-0.418362299	0.675682247	0.747427701
TBC1D10A	9.09613623	0.196783619	0.470338128	0.418387555	0.675663784	0.747427701
LOC105608413	1.422525332	-0.422791337	1.01085906	-0.41824954	0.675764678	0.747457025
KYAT3	15.95009393	-0.138898247	0.332467498	-0.417779928	0.676108028	0.747527496
LOC132658359	3.634973359	0.270810072	0.647873087	0.417998645	0.675948108	0.747527496
PDCD6	25.309966	0.123563289	0.295712032	0.417850056	0.67605675	0.747527496
SLC25A4	11.98055183	-0.217900721	0.521538353	-0.41780383	0.67609055	0.747527496
THG1L	15.22998628	0.147218392	0.352229927	0.417961056	0.675975591	0.747527496
HEBP1	1.512308673	0.43687999	1.046072644	0.41763829	0.676211597	0.747580166
ZBTB48	2.068310636	-0.354590478	0.849309834	-0.417504265	0.676309605	0.74762668
B3GALT5	2.803533716	-0.318879839	0.764143103	-0.417303824	0.676456192	0.747722218
HYLS1	3.597731094	-0.275136135	0.659430264	-0.417233103	0.676507915	0.747722218
NKX3-1	2.697314033	0.309636997	0.743734309	0.416327435	0.67717042	0.748330701
WASF2	192.9909392	-0.064309207	0.154441584	-0.416398259	0.677118602	0.748330701
LEMD3	22.32685582	0.112731895	0.270863479	0.416194516	0.677267673	0.748376299
THAP2	19.5110942	0.129042409	0.310848982	0.415128942	0.678047513	0.749176082
ATG2B	16.35419257	-0.143075024	0.344798586	-0.414952467	0.6781767	0.749256883
LOC132657235	2.057387389	0.393684747	0.949077016	0.414808009	0.678282456	0.749311787
TINF2	8.191425793	-0.188272344	0.454003744	-0.414693372	0.678366385	0.74934257
LOC132659441	1.531858001	0.417990731	1.010700011	0.413565575	0.679192289	0.750192888
HIPK3	5.127659526	0.251983248	0.609596825	0.413360499	0.679342511	0.75029681
BCOR	26.16011879	-0.120679128	0.292082132	-0.413168472	0.679483185	0.750342618
C20H6orf226	7.145232944	0.201984617	0.488888571	0.413150623	0.679496262	0.750342618
SPG7	6.56380397	-0.209437312	0.507100989	-0.413009078	0.679599963	0.75036251
WDR76	11.34217412	-0.157890103	0.382325689	-0.412972781	0.679626556	0.75036251
ZNF146	1.622737193	-0.465526492	1.128120412	-0.41265674	0.679858124	0.750556181
LOC101104012	11.62486475	0.153157328	0.371527909	0.4122364	0.68016616	0.750834233

COG4	9.996542076	-0.166792626	0.404894252	-0.411941205	0.680382518	0.751011045
CXXC4	7.206260194	0.202141052	0.490889583	0.411785174	0.680496889	0.751075262
EXOC4	153.8825159	0.060076387	0.146066495	0.411294777	0.680856399	0.75141001
CDC42EP1	11.93497398	-0.165221409	0.402171878	-0.410822879	0.681202416	0.751521924
IQCG	12.33801021	-0.146968683	0.357595798	-0.41099108	0.681079075	0.751521924
PLEKHJ1	82.08307015	0.124955744	0.303985987	0.41105758	0.681030314	0.751521924
SNAPC3	4.933827895	-0.223935306	0.545155752	-0.410773077	0.681238937	0.751521924
TP53I11	2.34126422	0.322099725	0.783896541	0.410895708	0.681149009	0.751521924
WIPF3	6.45140278	0.210872534	0.513602519	0.410575349	0.681383942	0.751619854
CRNKL1	32.05538086	0.097052611	0.23657481	0.410240681	0.681629401	0.751828567
SLX1A	4.534946177	-0.237770617	0.580696584	-0.409457579	0.682203892	0.752400134
LOC101117055_1	1.85055461	-0.365339265	0.895035213	-0.408184237	0.68313842	0.753254828
PTPRG	47.28417655	0.091858615	0.225030951	0.40820436	0.683123648	0.753254828
USP9X	91.38322106	0.069441735	0.1701289	0.408171303	0.683147915	0.753254828
SMAD2	71.69157812	-0.074216323	0.181919451	-0.407962551	0.683301169	0.753361662
BMP1	9.016634494	-0.184128398	0.451668191	-0.407662973	0.683521125	0.753472052
GNAI1	17.66271618	0.140373407	0.344393611	0.407595851	0.683570411	0.753472052
NXF1	7.997649706	-0.184212681	0.451818798	-0.407713628	0.683483931	0.753472052
LAMP2	137.5223911	-0.072811572	0.178902827	-0.4069895	0.6840157	0.753900705
LOC114108699	2.817683968	0.311047928	0.764633639	0.406793413	0.684159725	0.75399727
ZC3H7B	18.1407633	0.171818762	0.422603351	0.406572171	0.68432224	0.754114194
KCNC3	2.02474746	-0.343394791	0.844795712	-0.40648264	0.68438801	0.754124497
TEAD1	178.6127088	0.068579296	0.168852545	0.406149022	0.684633108	0.754332382
ANKRA2	9.061901483	0.1888993	0.465941242	0.405414424	0.68517291	0.754864913
ELP2	11.71207647	-0.162355182	0.400868536	-0.405008543	0.685471231	0.755131334
PIK3CB	13.12699587	0.161381186	0.399069478	0.404393707	0.685923227	0.75556699
LOC101120590	49.49034781	0.086393061	0.21377209	0.4041363	0.686112493	0.755713192
FBXL2	3.14359366	-0.280782594	0.695625672	-0.403640356	0.686477205	0.756052597
ISOC1	30.68519296	-0.113994907	0.282719744	-0.403208157	0.686795099	0.756340388
LOC101121964	13.14692999	-0.144906559	0.359472063	-0.403109376	0.686867763	0.75634268
LOC114110369	7.642934117	0.189233083	0.469590647	0.402974558	0.686966941	0.75634268
MOSPD1	7.887213171	-0.194268776	0.481996042	-0.403050564	0.686911027	0.75634268
LOC101104520	6.423537952	-0.208179052	0.516855403	-0.402780064	0.687110028	0.756437908
TRIOBP	8.752611419	0.172652604	0.428964891	0.402486562	0.687325976	0.756613325
POMK	54.33147359	-0.083774077	0.208219475	-0.402335456	0.687437164	0.756673403
TTC8	2.506615069	0.312305877	0.776497239	0.40219831	0.687538086	0.756722172
ATXN3	23.13556008	0.109278749	0.271918274	0.401880856	0.687771712	0.756916979
HFE	1.515843846	-0.419735604	1.045352262	-0.401525513	0.688033258	0.757142477
TTC38	54.40365615	-0.102193686	0.254790026	-0.401089823	0.688353994	0.757433068
NEURL2	2.388323119	-0.325626883	0.812890802	-0.400578875	0.688730201	0.757784645
HSDL2	48.37998636	-0.080748051	0.202159794	-0.399426857	0.689578708	0.758655775
OSBP	4.167469654	0.291985721	0.731650019	0.399078403	0.689835435	0.758875755

TBK1	11.59858764	-0.164013186	0.411186543	-0.398877806	0.689983243	0.758975889
SEC24B	34.08487632	-0.123725544	0.310781746	-0.39811072	0.690548573	0.759410254
TMEM138	2.015165996	-0.349569922	0.877962203	-0.398160559	0.690511837	0.759410254
USP49	13.75136132	-0.135956259	0.341465192	-0.398155544	0.690515533	0.759410254
ZKSCAN4	2.058529209	-0.339808833	0.853736708	-0.398025328	0.690611516	0.75941699
TAPBP	31.58914712	-0.134095147	0.337387935	-0.397450926	0.691034968	0.75982012
STAP2	5.085450438	0.226118929	0.569827052	0.396820277	0.691499997	0.760268895
SF3B6	17.89775256	0.130750873	0.329603014	0.396691983	0.691594613	0.76031038
SYDE2	19.8347283	-0.124000882	0.31395756	-0.394960651	0.692871928	0.761651961
IP6K1	9.497687202	0.158774091	0.40224342	0.394721411	0.693048499	0.761783408
PLEKHF2	42.26242265	0.090777653	0.230164776	0.394402888	0.693283612	0.761979176
ZNF414	20.93447252	-0.133883836	0.339731087	-0.39408768	0.693516306	0.762172254
LOC101111245	4.181715439	-0.26059378	0.66157368	-0.39389986	0.693654974	0.762261973
NEK10	1.659110322	-0.41961557	1.067969319	-0.392909761	0.694386132	0.763002716
C2H2orf69	12.19015979	0.142924519	0.363935767	0.392719077	0.694526979	0.763094747
ARSJ	2.490997335	-0.296027201	0.754114676	-0.392549317	0.69465238	0.763169793
CIMIP2B	1.84426054	0.360470004	0.919576099	0.391995839	0.695061288	0.763556274
CENATAC	2.684600812	-0.323449277	0.825528647	-0.391808665	0.695199592	0.763645443
YTHDF3	120.3177866	0.05549067	0.141671353	0.39168589	0.695290317	0.763682338
DEGS2	19.85621241	0.122951054	0.314175407	0.391345251	0.695542055	0.763896064
SERINC3	51.54167477	-0.081512886	0.208490589	-0.390966741	0.69582182	0.764140535
CAMSAP2	18.38045174	0.15263716	0.390577069	0.390799081	0.695945755	0.764213848
ZNF891	3.865729507	0.247793549	0.634215271	0.390708897	0.696012421	0.764224269
ADPRM	4.23129057	-0.241540791	0.619268774	-0.390041936	0.696505538	0.76446075
BBS4	17.79614071	-0.141432611	0.362454375	-0.39020804	0.696382717	0.76446075
LIMA1	407.7330262	-0.054655197	0.140030849	-0.390308261	0.696308615	0.76446075
SNX33	2.782433642	0.293575105	0.752697223	0.390030807	0.696513767	0.76446075
TNS4	24.65157422	0.111128795	0.284871046	0.390102108	0.696461044	0.76446075
RAB11FIP1	41.7843862	-0.093281506	0.239216982	-0.389945169	0.696577093	0.764467479
LOC101117703	3.30028665	0.282122732	0.724020461	0.389661269	0.69678704	0.764635105
FHAD1	3.431013477	0.272458457	0.699975107	0.389240209	0.697098462	0.764823784
LRRC20	2.633404449	0.304715629	0.782845988	0.389240838	0.697097996	0.764823784
NPHP1	15.063921	-0.151846061	0.390152484	-0.389196704	0.697130642	0.764823784
LMAN2	223.3127642	0.056718426	0.145992625	0.388501993	0.69764458	0.765324807
LOC101102861	15.47743751	-0.156410442	0.402832258	-0.388276855	0.697811163	0.765444728
RWDD2B	10.86493419	0.15132072	0.389926946	0.388074539	0.697960874	0.765546121
ZFP69	2.312453405	0.325071544	0.838145295	0.38784629	0.698129787	0.765668559
RAD52	4.043991805	-0.261613819	0.675163937	-0.38748192	0.698399467	0.765901484
AMHR2	3.324648617	-0.258014889	0.668131823	-0.386173626	0.699368085	0.766900798
NAA38	10.44945766	0.156843539	0.40640783	0.385926469	0.699551127	0.767038586
FHOD3	3.340551856	0.266870681	0.69235653	0.385452682	0.699902058	0.767360422
MRPL32	20.85211523	0.128920355	0.334836882	0.385024358	0.700219369	0.767645348

H1-2	6.38978198	-0.199323228	0.518272369	-0.384591654	0.700539979	0.767933843
B3GLCT	1.595302474	-0.384577483	1.00023327	-0.384487794	0.700616941	0.76793705
FLII	58.35602091	0.082010773	0.21332937	0.38443264	0.700657813	0.76793705
SMC2	184.9440256	0.075303847	0.196251316	0.383711295	0.70119244	0.768459999
KBTBD4	5.683246881	-0.194657304	0.507805972	-0.38333008	0.701475039	0.768706679
ATPSCKMT	13.6688458	0.148747649	0.388498024	0.38287878	0.701809646	0.769010307
YARS2	25.06077295	0.10746504	0.280819326	0.382683918	0.70195414	0.769105585
LOC121818807	2.086784679	-0.331162358	0.865929966	-0.382435499	0.702138364	0.769244375
PCBP1	246.3977378	-0.060201622	0.157549086	-0.382113434	0.702377228	0.76937994
TXNL4A	65.92341732	-0.089472843	0.234133686	-0.382144253	0.702354369	0.76937994
IRAK4	23.25141846	-0.104347665	0.2733572	-0.38172642	0.702664301	0.769631324
PPHLN1	15.18276624	0.125088758	0.327910997	0.381471676	0.702853284	0.769775238
DRAP1	17.63936612	-0.127392064	0.334696151	-0.380620045	0.703485205	0.770277976
LOC114109662	6.366297875	-0.183110634	0.480993686	-0.380692387	0.703431519	0.770277976
ZNF213	11.97988259	-0.159345428	0.418583091	-0.38067813	0.703442099	0.770277976
TAF9B	6.143411574	-0.211463796	0.556628284	-0.379901278	0.704018699	0.770798978
CDS2	15.93991792	0.124114763	0.326901143	0.37967063	0.704189924	0.770923297
DR1	29.61424383	-0.105019824	0.276704821	-0.379537385	0.704288848	0.770968447
UBE2D3	258.8241078	-0.061667735	0.162998923	-0.378332164	0.705183854	0.771884972
TMBIM4	112.168668	-0.074649991	0.197584143	-0.377813677	0.705569013	0.77224332
CCDC174	32.01362737	-0.091147351	0.241405517	-0.377569463	0.705750453	0.772345911
FTSJ3	29.70724141	-0.103396733	0.27387544	-0.377531964	0.705778315	0.772345911
LGR4	146.5128377	-0.073848324	0.195719225	-0.37731768	0.705937535	0.772456904
LRRC58	19.09817826	0.113617977	0.301229451	0.377180839	0.706039219	0.772504928
LOC105602343	1.543799589	-0.371636061	0.985614866	-0.377060122	0.706128926	0.772506916
LOC121819263	3.437467664	0.256036493	0.679100742	0.377022844	0.706156629	0.772506916
CBFA2T2	3.705858486	0.259539556	0.689035062	0.376671044	0.706418084	0.772729692
SLC25A28	5.226253	0.223286277	0.593294863	0.376349588	0.706657018	0.772927799
LOC114115351	5.356092479	-0.21417584	0.569393401	-0.376147388	0.706807327	0.773028944
NOL9	13.05952449	0.165808795	0.441787868	0.375313147	0.707427589	0.773644016
RPN2	41.779625	-0.094661267	0.252655378	-0.374665552	0.707909214	0.774078225
SUCLA2	9.473242535	-0.170029701	0.453868168	-0.374623541	0.707940463	0.774078225
BRD3	61.41821439	0.077576919	0.207198819	0.374408112	0.708100709	0.774126785
CISD1	64.61254345	0.075492493	0.201623538	0.374423017	0.708089622	0.774126785
HMGXB4	61.31715728	0.099814237	0.266902589	0.373972533	0.708424751	0.774354369
IL17RA	87.71243349	-0.072380124	0.193509512	-0.3740391	0.708375227	0.774354369
HOGA1	2.287751843	0.312170283	0.836627907	0.373129178	0.709052303	0.774948049
LOC101102013	4.834925687	0.218787027	0.586668922	0.372930998	0.7091998	0.774948049
SNX15	3.186373502	0.298672209	0.800723476	0.373002939	0.709146257	0.774948049
TUBGCP6	8.389387304	-0.162469753	0.435548521	-0.373023316	0.709131091	0.774948049
NCEH1	11.46580884	-0.146500739	0.393444029	-0.372354715	0.709628766	0.775290018
SNX7	29.07922367	-0.098781623	0.265284466	-0.372361129	0.709623991	0.775290018

CASP8	4.304304759	0.238103059	0.640452902	0.371772941	0.710061912	0.775699838
PRPF6	94.1147588	0.086295975	0.232190447	0.371660315	0.710145776	0.775728052
LYRM2	7.89365439	0.163134567	0.439074926	0.371541523	0.710234235	0.77576128
EPM2AIP1	51.3507387	0.074642044	0.201196438	0.370990884	0.710644322	0.776145776
ZNF23	6.087291089	-0.200361404	0.540662299	-0.370585121	0.710946566	0.776412435
CMTR1	8.549699926	0.159906671	0.431792172	0.370332491	0.711134768	0.776554518
TRAPPC12	6.417401193	-0.197925897	0.535235754	-0.369791994	0.711537481	0.776930804
STX8	14.91276747	-0.12149035	0.328746898	-0.369555882	0.711713429	0.777059442
LOC132658471	1.556829211	-0.361878142	0.980594942	-0.369039372	0.712098378	0.777416231
ZNF449	4.377534025	0.219759245	0.595924163	0.368770488	0.712298804	0.77757153
ENGASE	5.066621161	-0.210695466	0.572649582	-0.367930882	0.712924772	0.778191303
TCTN2	6.914106185	-0.174877874	0.475634255	-0.367673	0.713117075	0.778337647
ISCA2	3.054631901	-0.258823127	0.704859463	-0.367198201	0.713471181	0.778660554
PFDN1	75.59260503	-0.064244783	0.175148815	-0.366801126	0.713767368	0.778920202
RFX1	88.17506379	-0.076257925	0.207990215	-0.366641886	0.713886161	0.778986237
SERPINH1	34.59911633	0.09624263	0.262606037	0.366490548	0.713999065	0.779045837
CTNNBL1	39.78557897	-0.089479067	0.244244587	-0.366350254	0.714103735	0.779096442
LOC132659926	4.241210963	0.240842454	0.657923013	0.366064796	0.714316726	0.77926521
FAM181B	1.913443966	0.344319804	0.940970312	0.365919944	0.714424814	0.779319518
TBC1D31	41.43099629	-0.105717799	0.289059484	-0.365730256	0.714566367	0.77941032
NEK7	22.53000517	0.109484085	0.299924619	0.365038674	0.715082538	0.779909686
STK3	21.87403281	0.101435856	0.278706906	0.363951714	0.715894067	0.780731078
PTPA	19.53082982	0.109804811	0.301879686	0.363736998	0.716054413	0.780842236
GSKIP	34.43766616	0.099703108	0.274258391	0.36353713	0.716203682	0.780907514
LOC114116344	4.042587427	0.240759842	0.662479804	0.363422161	0.71628955	0.780907514
ZNF34	13.79614381	-0.124452144	0.34244278	-0.363424641	0.716287698	0.780907514
LOC105601893	24.71409847	-0.110909863	0.305683745	-0.362825517	0.716735229	0.781329669
MISP3	1.31596462	-0.414999824	1.144143785	-0.362716496	0.716816676	0.781354729
MGARP	1.810603223	-0.344960102	0.95231998	-0.362231298	0.717179193	0.781686137
DPY30	19.02770178	-0.108021541	0.298359462	-0.362051667	0.717313421	0.781768687
PRKAB1	5.892936561	-0.181702413	0.502051832	-0.36191963	0.71741209	0.781812475
IPO4	15.21746928	-0.128791979	0.35620728	-0.361564703	0.717677345	0.781931223
RMND1	5.87851228	-0.184483397	0.510174458	-0.361608455	0.717644646	0.781931223
SIPA1L2	24.61627666	0.102665937	0.283969198	0.361538992	0.717696562	0.781931223
MAP2K4	1.50419651	0.363877283	1.007328939	0.361229852	0.717927628	0.782119218
SRD5A3	3.271522493	0.252639026	0.699698973	0.361068167	0.718048489	0.782187132
USP25	32.57636784	-0.086051281	0.23844463	-0.360885802	0.718184818	0.782271883
LOC132658110	83.14733588	0.09757794	0.270651279	0.360530126	0.718450732	0.782497758
RHBDL3	1.792260422	0.338179532	0.938382132	0.360385733	0.718558694	0.782551577
GK5	66.27517386	0.078393882	0.217842688	0.359864647	0.718948356	0.78291215
C1H1orf210	11.35325463	0.164295088	0.457020776	0.359491508	0.719227429	0.783152246
ZNF786	11.8615308	0.145171493	0.404952813	0.358489899	0.719976726	0.783904278

ACTR5	3.988228702	-0.227356471	0.634439057	-0.358358252	0.72007523	0.783947668
MPHOSPH9	22.60349586	0.101502488	0.283306473	0.358278039	0.720135251	0.783949158
N6AMT1	4.072704474	0.217844209	0.608443229	0.358035391	0.720316829	0.784082966
P3H3	3.62513487	-0.235399495	0.657822473	-0.357846538	0.720458161	0.784172946
FPGS	5.341998108	0.199675231	0.558229626	0.357693719	0.720572534	0.784233572
MRPS25	4.694823831	-0.214193644	0.599702926	-0.357166248	0.720967352	0.784599383
MAP3K9	2.219819518	-0.292213905	0.818614566	-0.356961526	0.721120608	0.784642107
UBIAD1	8.747075468	-0.176732174	0.495107783	-0.35695697	0.72112402	0.784642107
SLC45A4	3.510023854	-0.238827369	0.669741471	-0.356596358	0.721394004	0.784871979
R3HDM2	117.8947476	0.081903407	0.229776335	0.356448401	0.721504787	0.784928617
CCDC120	5.618437026	-0.212405919	0.597145281	-0.355702248	0.722063561	0.785472578
RREB1	101.9546389	-0.063410212	0.178308612	-0.355620577	0.722124731	0.785475192
PPM1B	57.56661411	-0.093507513	0.26302054	-0.35551411	0.722204476	0.785498009
RPTOR	7.165501318	-0.165182233	0.464816563	-0.355370797	0.722311823	0.785535664
SPRY4	2.259852983	0.314072377	0.883936623	0.355310968	0.72235664	0.785535664
AP3S1	8.619581063	-0.151806013	0.427848933	-0.354812181	0.722730303	0.785838253
LOC121819091	4.882128	0.196842324	0.554825224	0.35478258	0.72275248	0.785838253
ARMH3	16.93534457	0.111939762	0.315687721	0.354590169	0.722896642	0.785854561
HIKESHI	7.446734468	-0.200857692	0.566530253	-0.3545401	0.722934158	0.785854561
LOC114117648	1.50549827	0.360922209	1.018038288	0.354527146	0.722943864	0.785854561
LOC101115571	8.881157645	0.154050901	0.435119717	0.354042565	0.723306984	0.786185342
PHKA2	1.709598866	-0.339052763	0.95839749	-0.353770504	0.72351088	0.786343017
CHAC2	4.802296955	0.199295154	0.563598816	0.353611733	0.72362988	0.786367846
DUSP26	2.470166532	-0.303188673	0.857600476	-0.353531372	0.723690114	0.786367846
LOC132658323	2.981362021	0.245756216	0.695199604	0.35350454	0.723710226	0.786367846
UBE2F	4.179508701	-0.213293602	0.604041849	-0.353110638	0.724005498	0.786624735
SCNM1	7.684311009	0.156298985	0.44307635	0.352758582	0.724269437	0.786847541
LOC121820590	3.248530966	-0.242805226	0.689273002	-0.352262783	0.724641197	0.787187438
SZRD1	62.44458478	0.070714306	0.200838005	0.352096236	0.724766091	0.787259128
RBFOX2	269.5134921	0.068363526	0.194662869	0.351189349	0.7254463	0.787933955
KLHL2	4.53300555	0.203677397	0.580632067	0.35078565	0.725749164	0.788166661
LTA	2.648523022	0.265936999	0.758202717	0.350746566	0.725778487	0.788166661
CRYBG2	5.10296105	-0.210826728	0.601491044	-0.350506845	0.725958354	0.788297941
METTL23	2.329231061	0.288346297	0.824937696	0.34953706	0.726686153	0.789024137
ARL6IP6	14.0724517	0.118523225	0.339511648	0.349099143	0.72701488	0.789060567
LOC132658442	4.617224402	-0.198012147	0.567170374	-0.349122866	0.72699707	0.789060567
SBF1	11.17640861	0.148076077	0.424159563	0.349104653	0.727010744	0.789060567
SSR4	140.788684	0.08628362	0.247084133	0.349207449	0.726933574	0.789060567
TOMM34	10.64045392	0.135412836	0.387640349	0.349325957	0.726844613	0.789060567
POLI	3.76719929	0.286866461	0.822831855	0.34863315	0.727364737	0.789376184
ADAMTSL5	4.335750809	0.223658611	0.642266766	0.348233199	0.727665057	0.789469297
CRIPT	22.44038895	0.114214467	0.328084086	0.348125595	0.727745863	0.789469297

FZD2	3.100047364	0.252791356	0.726129226	0.348135494	0.72773843	0.789469297
SLC4A8	2.062650202	-0.298625762	0.857381071	-0.348299924	0.727614951	0.789469297
VCP	627.7578994	-0.061265807	0.175949306	-0.348201471	0.727688883	0.789469297
CASZ1	25.19396312	0.097365023	0.280290699	0.347371581	0.728312181	0.789955417
CISD2	34.13079213	-0.105277032	0.303039213	-0.347403991	0.728287835	0.789955417
RNF186	8.312362954	-0.153639479	0.44272529	-0.347031179	0.728567895	0.790168654
ZFTA	27.69861206	0.099243302	0.286092602	0.346892236	0.728672279	0.790217743
ADAT1	9.038693329	0.143788314	0.414873074	0.346583865	0.728903969	0.790404872
ZNF354A	20.67203636	0.120694946	0.348400174	0.346426195	0.729022442	0.790469211
LOC101108987	6.421111713	-0.176482942	0.509907061	-0.346108057	0.729261509	0.790664287
LOC105612393	2.421501693	0.273533036	0.790555806	0.34600092	0.729342023	0.790687443
C14H19orf12	3.711342388	0.218654083	0.632195025	0.345864923	0.729444231	0.790734111
ADO	26.83745612	-0.090098839	0.260721728	-0.345574722	0.729662345	0.790906407
SRGAP2	44.27257589	-0.073077839	0.211536553	-0.34546199	0.729747081	0.790934113
ATXN7L1	9.756251713	0.142988093	0.414162828	0.345246081	0.729909378	0.791045873
C3H2orf42	2.475369319	-0.264524598	0.768080515	-0.344396964	0.730547772	0.791635642
GPATCH11	49.21319191	0.076793918	0.223001703	0.344364714	0.730572022	0.791635642
ZBTB7C	30.83473854	-0.120274224	0.349775204	-0.343861493	0.730950452	0.791981497
PSMA6	72.01056926	-0.060525547	0.176118753	-0.343663276	0.731099532	0.792078816
RPL36AL	607.4817773	0.041818132	0.12172721	0.343539728	0.731192459	0.792115286
RPAP1	9.51039178	-0.176514087	0.515318286	-0.342534103	0.731948984	0.792870583
PLPBP	9.408275376	-0.157288916	0.46195804	-0.340483123	0.73349273	0.794478431
NCS1	5.107510031	-0.192763626	0.567734978	-0.339531002	0.734209747	0.795126193
RPL13	669.7967318	-0.044408188	0.1307674	-0.339596781	0.734160202	0.795126193
GOSR2	38.72692347	-0.075158739	0.221639121	-0.339104118	0.734531296	0.795409974
MRPL38	43.46152336	-0.082431185	0.243294827	-0.338811912	0.734751427	0.795583893
PPP2R5A	101.6366512	0.054269392	0.160333989	0.338477153	0.735003642	0.795792521
PTPN9	14.6395173	0.119057973	0.352051252	0.338183639	0.735224805	0.795967499
PIK3R4	14.15456444	0.123130478	0.364265754	0.338023755	0.735345286	0.795968991
SND1	18.77940397	-0.110220826	0.325999595	-0.338101112	0.735286992	0.795968991
CSRNP2	2.874287234	0.244738718	0.72450615	0.337800745	0.735513348	0.796086437
C2	3.564700452	0.21582879	0.63976413	0.337356817	0.735847934	0.796377285
LOC132659212	3.204696678	-0.239339343	0.70960332	-0.337286109	0.73590123	0.796377285
TDRD7	6.931997482	-0.168160083	0.499816102	-0.336443908	0.736536143	0.796999846
IL17RE	2.381957686	-0.289367494	0.86133324	-0.335953009	0.736906302	0.79733584
LOC105614203	29.0018577	0.08727901	0.260122095	0.335530938	0.737224611	0.797615683
STAG2	61.043364	-0.060510073	0.180526094	-0.335187405	0.737483722	0.797831439
BABAM1	16.17926413	-0.118057109	0.352702726	-0.334721284	0.737835344	0.798082642
GRHPR	5.828874549	0.183856822	0.549169082	0.334790919	0.737782811	0.798082642
CINP	18.36022379	-0.11187692	0.33437011	-0.334590075	0.737934333	0.798125125
IFTAP	9.760189259	-0.13751341	0.411327311	-0.334316263	0.738140919	0.798224702
ZNF131	28.782541	0.087097005	0.260527872	0.334309739	0.738145842	0.798224702

C17H12orf76	7.698739648	-0.160263867	0.479698754	-0.334092731	0.738309585	0.798337183
LSG1	35.80606775	0.102771369	0.30800461	0.333668281	0.738629887	0.798618918
GLCE	21.34251867	-0.12617869	0.378435431	-0.333421979	0.738815774	0.798657203
HIBADH	4.762708753	-0.216590109	0.649671875	-0.333383847	0.738844554	0.798657203
TNFAIP1	13.18663756	-0.115212481	0.345498979	-0.333466921	0.738781855	0.798657203
FAM174A	8.575914985	0.150764522	0.452349919	0.333291807	0.738914023	0.798667705
LOC105605028	3.091292762	0.238835253	0.717340042	0.332945659	0.739175303	0.798884042
MRPL27	45.67255534	0.078175575	0.234854386	0.332868279	0.739233715	0.798884042
GADD45B	2.432851387	0.257028967	0.773138453	0.332448821	0.739550379	0.799161643
MCTS1	40.46544722	0.085387689	0.257115092	0.332099095	0.739814433	0.799382354
LOC114118713	1.753178722	0.32634632	0.983301491	0.331888361	0.739973559	0.799489661
LOC101112585	6.326188225	0.176127568	0.53098928	0.331697031	0.740118043	0.799581132
FN3KRP	7.007790471	0.1720992	0.519906341	0.331019621	0.740629665	0.800004534
LOC105611737	1.436130704	-0.348498477	1.052644599	-0.331069458	0.740592021	0.800004534
PBX1	47.32526849	-0.075727408	0.229164778	-0.330449594	0.741060273	0.800404979
B3GNT3	6.815581744	-0.154122157	0.467356916	-0.329773995	0.741570738	0.800826896
ZNF140	2.435208076	0.259445754	0.786652528	0.329809852	0.741543643	0.800826896
EXOSC3	5.252216514	-0.187935789	0.571140355	-0.329053599	0.742115176	0.801350092
TMEM81	1.555510505	0.331329137	1.007637122	0.328817914	0.742293322	0.801477707
LOC132659356	3.032142357	-0.261630313	0.796828854	-0.328339407	0.742655052	0.801803507
APBB1	3.724576124	0.217783614	0.663675419	0.328147778	0.742799931	0.801895152
QARS1	15.12193851	0.10830144	0.330231202	0.32795641	0.742944621	0.801986578
MTF1	30.5582326	-0.083394477	0.254623937	-0.327520176	0.743274485	0.802277862
ARPIN	6.964796207	0.153892208	0.471382154	0.326470161	0.744068661	0.803005388
CBFA2T3	35.79644416	0.081652049	0.250077049	0.326507567	0.744040365	0.803005388
RALGAPA2	49.0336893	0.06984681	0.214297733	0.325933499	0.74447467	0.803378689
SGSM3	65.91157364	-0.070409964	0.216395336	-0.325376533	0.744896114	0.803768585
L3HYPDH	1.141191028	0.374051991	1.150467828	0.325130335	0.745082431	0.803854984
PBX2	84.15771899	-0.090326809	0.277833079	-0.325111787	0.745096468	0.803854984
B4GALT3	28.86375984	0.090911421	0.27998664	0.324699139	0.745408787	0.804127025
GASK1B	9.749207526	-0.147588853	0.455879579	-0.323745261	0.746130902	0.804841064
TRMT1	8.498565242	-0.137093942	0.423707658	-0.323557858	0.746272797	0.804929165
RAB3B	7.491748558	-0.159505805	0.49327936	-0.323357954	0.746424168	0.80502747
HINFP	10.34311628	-0.130747287	0.404627171	-0.323130271	0.746596586	0.805148456
RSKR	3.798684329	-0.216163483	0.669916271	-0.322672387	0.746943366	0.805457445
LOC114118023	16.28090448	-0.111011037	0.344577485	-0.322165672	0.747327189	0.805806325
LOC101107382	3.881179004	-0.207838245	0.647717528	-0.320877907	0.748302917	0.806728246
RRP1B	24.96568849	-0.103599414	0.322857652	-0.320882634	0.748299335	0.806728246
ZCCHC4	7.063907128	-0.148958039	0.464405307	-0.32075008	0.748399792	0.806767612
TJP2	19.52054658	0.09412283	0.293665365	0.320510491	0.748581379	0.806898283
SCAMP4	18.19082392	-0.101852165	0.317966629	-0.320323443	0.748723154	0.806986023
CCDC150	2.393197883	-0.251979336	0.787936837	-0.319796365	0.749122703	0.80735156

TRIM23	10.55557975	0.138050737	0.432192934	0.319419238	0.749408625	0.807594588
NVL	29.64508323	-0.081498447	0.255256479	-0.31928062	0.749513727	0.807642734
AIFM2	6.016229065	-0.162540465	0.509607504	-0.31895226	0.749762714	0.807845904
LOC105602212	60.81921519	0.065448901	0.205307099	0.318785377	0.749889268	0.807917133
PAFAH2	18.87244458	0.103616875	0.32580614	0.318032296	0.750460439	0.808467335
FANCC	3.393515244	0.243378218	0.765778482	0.317818042	0.750622964	0.808577252
MUC1	37.91358238	0.120159397	0.378183029	0.317728158	0.75069115	0.808585536
MCEE	24.73719074	0.095547251	0.301185159	0.31723758	0.751063337	0.808921238
LOC132658424	2.44094147	-0.254959763	0.804460057	-0.316932781	0.751294608	0.809105127
CCNH	12.53510152	-0.113537112	0.358948887	-0.3163044	0.751771472	0.809553456
DYNC1LI2	40.14167672	0.071300541	0.225744901	0.315845633	0.75211968	0.809845649
LOC101109657	7.408423399	0.143946217	0.455980135	0.315685282	0.752241399	0.809845649
LOC132659740	1.948303112	0.272023685	0.861527971	0.315745622	0.752195595	0.809845649
RIPK2	17.59383686	0.107730667	0.34132205	0.315627622	0.752285169	0.809845649
FOXJ2	24.38252957	-0.08805188	0.279101108	-0.315483806	0.752394345	0.809897947
FZD7	9.11536205	-0.142266663	0.451540966	-0.315069227	0.752709093	0.810171504
LUC7L	140.0550704	-0.058779416	0.186698008	-0.314836867	0.752885518	0.810296146
RBM34	51.37483389	-0.077520977	0.246529464	-0.314449137	0.753179941	0.810547753
LOC101109541	24.72270119	0.088101694	0.28093115	0.313605998	0.753820303	0.811171578
ASTE1	17.58764918	0.110749413	0.353385568	0.313395403	0.753980276	0.811172452
SASH1	25.00267214	-0.087668943	0.27973687	-0.313397883	0.753978392	0.811172452
TTLL5	28.41891449	-0.078640534	0.250954866	-0.313365249	0.754003183	0.811172452
LOC101103154	6.982555815	0.142461234	0.454821481	0.313224507	0.754110101	0.811222182
PHF6	50.24616531	0.064173693	0.204956476	0.313108881	0.754197942	0.811251383
LOC105609861	3.502046292	0.213145149	0.682070893	0.312497061	0.754662797	0.811686082
TAMALIN	10.52621842	-0.156129568	0.500847981	-0.311730453	0.755245382	0.812247327
KLF10	50.22578516	-0.088913002	0.285559297	-0.31136441	0.755523607	0.812481175
FADD	13.06628503	0.112132432	0.3611324	0.310502276	0.756179029	0.813120587
NBEAL2	5.823993128	-0.161262772	0.520195893	-0.310003931	0.756557967	0.813331761
RAB33B	13.72298689	-0.107036456	0.345203863	-0.310067377	0.75650972	0.813331761
TERF2	18.55640218	-0.099795263	0.321820079	-0.310096447	0.756487614	0.813331761
PUM1	313.5690337	-0.053994347	0.174287588	-0.309800299	0.756712825	0.813432814
ACSL3	75.89289515	0.068070248	0.220513251	0.308690056	0.757557309	0.814275109
ACE2	3.221148253	0.218154216	0.707038209	0.308546573	0.757666468	0.814326955
LOC114113942	4.411940904	-0.177445995	0.575362389	-0.308407359	0.757772383	0.814375306
PSME4	28.84846887	0.077352604	0.252542833	0.306294989	0.759380054	0.81603745
LOC114114873	2.088485673	-0.26320206	0.860251369	-0.305959478	0.759635499	0.816246329
E2F3	35.85059987	0.080066343	0.261945751	0.305660019	0.759863517	0.816360081
LOC114111357	3.67808628	0.202083586	0.661032581	0.305708965	0.759826247	0.816360081
NDUFB4	19.950316	-0.090132468	0.294955922	-0.30557945	0.759924869	0.816360376
MELTF	3.276098161	0.255018358	0.836835863	0.304741191	0.760563275	0.81698053
ANO8	2.194576914	-0.338697615	1.112252397	-0.304515069	0.760735514	0.817099878

LOC132657957	13.31615125	0.108721079	0.357204465	0.304366516	0.760848675	0.817155756
CHPF2	5.177446153	0.165538836	0.544539387	0.303997911	0.761129483	0.817260336
MPI	25.51588999	0.081914206	0.26940237	0.304058964	0.76108297	0.817260336
MTHFS	31.01630502	0.078630165	0.258600437	0.30406045	0.761081838	0.817260336
DALRD3	13.51691159	-0.107872429	0.355581184	-0.30336934	0.761608408	0.81758175
LOC105603932	1.97883222	0.280163384	0.923481333	0.303377419	0.761602252	0.81758175
SRSF2	209.9966216	-0.046256465	0.152478328	-0.303364197	0.761612328	0.81758175
JMJD7	8.568984583	0.133851688	0.441673837	0.303055505	0.761847563	0.817736758
TMEM164	56.45516046	0.062585408	0.206542862	0.303014142	0.761879084	0.817736758
FSD2	3.105812676	-0.223624027	0.738374926	-0.302859725	0.761996766	0.817797396
LCORL	23.8358372	-0.081600539	0.270175073	-0.302028377	0.76263043	0.818411749
BTC	10.24444363	0.132042982	0.438337115	0.301236143	0.763234431	0.818795596
FANCB	2.321300496	-0.245013276	0.813454427	-0.301200987	0.763261237	0.818795596
RMND5B	1.926676551	-0.283039523	0.939838935	-0.301157478	0.763294413	0.818795596
RTEL1	2.607015575	0.221605182	0.735582782	0.301264775	0.763212599	0.818795596
SNRNP48	48.29525131	-0.083491154	0.277006595	-0.301404934	0.763105732	0.818795596
PMM1	4.563909393	-0.172552485	0.573453706	-0.300900463	0.763490397	0.81887439
ZNF275	2.691594041	-0.227353593	0.755447089	-0.300952372	0.763450814	0.81887439
ELMO3	68.9844632	0.071461548	0.237642229	0.300710646	0.76363515	0.818963926
MCPH1	1.735512889	0.340788688	1.133988564	0.300522156	0.763778899	0.819052372
MYO9A	41.61080545	-0.065884872	0.219310655	-0.300418017	0.763858322	0.819071828
LOC101117851	6.629591447	-0.144051356	0.479644407	-0.300329481	0.763925848	0.81907781
PRPF40A	382.6170196	-0.040926433	0.136307849	-0.300250009	0.763986462	0.81907781
LOC121817540	3.837425325	0.203916155	0.679885473	0.299927213	0.764232676	0.819227694
NEMP2	7.356971161	0.151556228	0.50534575	0.299906011	0.764248849	0.819227694
SPIN1	26.43100437	0.087431004	0.292003248	0.299417916	0.764621194	0.819561097
KLHDC2	18.69291859	0.103193606	0.345216997	0.298923885	0.764998122	0.819899359
CCNY	93.96343673	-0.047826406	0.160061309	-0.298800542	0.765092238	0.819934481
LOC105609426	2.770805353	-0.225197876	0.754313171	-0.298546923	0.765285769	0.820076131
UROC1	2.902025654	-0.222487522	0.74568468	-0.298366761	0.765423255	0.820157707
BCL9	4.275734102	-0.194386031	0.651885349	-0.29819052	0.765557757	0.82023607
ATG13	3.622580333	-0.20930303	0.702231548	-0.298054154	0.765661832	0.820281825
GPATCH3	15.96604066	0.110493726	0.371565067	0.297373828	0.766181122	0.820735049
MTO1	16.11245311	-0.095825639	0.322277366	-0.29733903	0.766207687	0.820735049
KIF4A	26.60949062	0.08912703	0.299946714	0.297142879	0.766357429	0.820829666
GNB5	3.290683785	-0.20779716	0.700273222	-0.296737265	0.766667105	0.821029767
LOC114114008	1.508006735	-0.344296554	1.160057578	-0.296792642	0.766624823	0.821029767
PAK1	40.14313217	-0.073316309	0.247495086	-0.296233391	0.76705185	0.821375984
ZCWPW1	1.972045616	-0.249232533	0.842142755	-0.295950457	0.767267917	0.821541535
ERICH1	7.550670544	0.133904062	0.45310741	0.295523885	0.767593709	0.821758712
GRIN1	2.109379944	0.244716729	0.827948412	0.29557002	0.767558471	0.821758712
TRMT2A	2.677620888	-0.231580071	0.784859118	-0.295059413	0.767948494	0.822072687

DMAC2	3.933689243	-0.174857164	0.59368256	-0.29452973	0.768353149	0.822439993
DMXL1	19.27848172	0.087264401	0.296839666	0.293978236	0.768774534	0.822825146
FBXO45	9.842732649	-0.123979385	0.422204791	-0.293647509	0.769027268	0.823029744
LOC101119611	26.63201716	0.079339211	0.270446336	0.29336397	0.769243962	0.82319574
CPLANE1	16.28253467	-0.091313218	0.31178909	-0.292868548	0.76962263	0.823535032
LOC106990140	4.424305292	-0.180902757	0.618803955	-0.292342599	0.770024691	0.823899297
BBLN	8.189397222	-0.126344338	0.43514011	-0.290353234	0.771546017	0.825263706
FGD2	8.350735334	-0.12858541	0.442867021	-0.290347675	0.77155027	0.825263706
MAX	24.29732191	0.098840366	0.34050858	0.29027276	0.771607576	0.825263706
MOSPD3	9.556951176	0.117111495	0.403099027	0.290527852	0.771412446	0.825263706
STBD1	5.98335766	0.164445735	0.566524025	0.290271423	0.771608599	0.825263706
LOC114110974	15.41329536	-0.100335709	0.346000997	-0.289986763	0.771826364	0.825373926
TMED8	20.50962861	0.086511306	0.298340288	0.28997527	0.771835157	0.825373926
YIPF1	5.641614214	-0.185273797	0.63916854	-0.28986689	0.771918073	0.825396557
COQ8B	13.258925	-0.117605213	0.407262082	-0.288770348	0.772757125	0.82622764
CLYBL	35.36760923	0.080898268	0.280437224	0.288471933	0.772985512	0.826405724
CASK	59.22972968	-0.058226192	0.202085153	-0.288127014	0.773249515	0.826621852
LOC114110582	2.829663005	0.212417172	0.739984349	0.287056304	0.774069209	0.827431946
AFF1	26.587192	-0.076633288	0.267218953	-0.286780886	0.774280099	0.827437401
CHCHD3	9.060141682	0.129777757	0.452578668	0.286751821	0.774302355	0.827437401
MRPL44	5.102349202	0.164949692	0.575286329	0.28672625	0.774321937	0.827437401
TBC1D22A	26.61730989	0.079706424	0.277951541	0.286763742	0.774293227	0.827437401
BDH1	1.932990986	-0.253455096	0.884314826	-0.286611836	0.774409551	0.82746487
SRRM1	372.9649007	-0.056328585	0.196664463	-0.286419744	0.774556654	0.827555895
ARL6	5.845206743	-0.148819836	0.520382107	-0.285981847	0.774892025	0.827848039
LOC101121635	73.26312125	-0.062836185	0.219839687	-0.285827305	0.775010394	0.827908322
LOC101104790	1.604927097	0.269123474	0.94214826	0.285648752	0.77514716	0.827988247
DDX31	35.71211434	0.064355855	0.225454796	0.285449038	0.775300142	0.82808548
MYO19	7.416096192	-0.155637912	0.54544814	-0.285339523	0.775384036	0.828108912
DYM	64.20297089	-0.051689953	0.181416729	-0.284923852	0.775702482	0.82838282
PEX12	4.609961504	-0.164516359	0.577643047	-0.284806266	0.775792571	0.828412839
FAM118A	21.81166429	-0.082023667	0.288393254	-0.284416039	0.776091569	0.828665914
ALG2	26.75972106	-0.074682751	0.262671284	-0.284320196	0.77616501	0.82867277
RGS12	4.514472919	0.17807072	0.626467283	0.284245841	0.776221987	0.82867277
KRBA2	6.399333161	0.148122212	0.522553178	0.283458638	0.776825284	0.829250599
DTYMK	3.359364005	-0.209339307	0.73999826	-0.282891621	0.777259918	0.829648304
BMPR1A	140.463958	0.045502968	0.161227942	0.282227555	0.777769031	0.83007566
PDP1	28.55674769	-0.070628728	0.250272431	-0.282207386	0.777784496	0.83007566
LOC106990145	6.207879132	0.135437215	0.480467756	0.281886168	0.778030796	0.830205941
LOC132659871	6.607440508	-0.14207855	0.503904684	-0.281955209	0.777977855	0.830205941
BLOC1S2	27.11876379	-0.076782537	0.272939752	-0.281316799	0.778467426	0.830539242
CENPE	146.1930637	0.069737907	0.247879461	0.281337981	0.778451181	0.830539242

FEM1C	30.44379637	-0.090085434	0.321782798	-0.279957269	0.779510288	0.831585483
LOC105604571	3.987687396	0.174889494	0.626326475	0.279230563	0.78006789	0.83211392
ZSCAN29	20.69001313	0.090713065	0.325277065	0.278879377	0.780337396	0.83233498
ISG20L2	7.614313132	-0.145776786	0.525270011	-0.277527334	0.78137522	0.83330896
THOC1	21.92942789	0.082023625	0.295521352	0.277555663	0.781353471	0.83330896
RCE1	6.067879602	-0.146980617	0.530801813	-0.276903005	0.781854585	0.833753661
LOC101120607	4.230409454	0.177155817	0.641825638	0.276018605	0.782533777	0.834279167
LOC101122817	1.220637745	0.355241292	1.286680027	0.276091402	0.782477864	0.834279167
MAEA	25.69334619	-0.084690356	0.306829673	-0.276017488	0.782534634	0.834279167
CCDC77	12.0810838	0.104783288	0.381049085	0.274986325	0.783326744	0.834912274
MIS18A	9.134566106	-0.110788187	0.402804741	-0.275041914	0.783284037	0.834912274
TMEM187	9.553072797	-0.125917481	0.458016365	-0.274919175	0.783378335	0.834912274
ZC3H10	11.56327472	0.100704057	0.366118391	0.275058723	0.783271123	0.834912274
KCNH2	3.924537466	0.189630472	0.691687145	0.274156421	0.783964416	0.835387875
KLHL12	2.524510971	0.208224548	0.759681897	0.274094392	0.784012084	0.835387875
TUG1	26.76282387	0.078186714	0.285090452	0.274252307	0.783890733	0.835387875
LOC114118016	2.746120284	-0.212182629	0.774751068	-0.273872006	0.784182985	0.83550337
ACVR1B	15.8924557	0.09404258	0.343734827	0.27359049	0.784399343	0.835605389
LOXL2	4.216027154	0.170989847	0.624997779	0.273584727	0.784403772	0.835605389
RRP8	68.05139247	0.052248962	0.191189914	0.273283047	0.784635646	0.835785786
IRF6	22.61652218	0.079706027	0.292634492	0.272374	0.785334462	0.836463497
BHLHA15	2.542679669	0.232864103	0.857333253	0.271614453	0.785918485	0.836952153
RLIG1	10.12528096	0.106625791	0.392548871	0.271624246	0.785910955	0.836952153
BTBD2	3.209785522	0.205940096	0.759125613	0.271285928	0.786171129	0.837154502
CDYL	6.379248273	0.148827937	0.550042403	0.27057539	0.786717626	0.837669704
CRACR2A	24.94478191	0.07391912	0.273958537	0.269818641	0.787299781	0.838156023
ZNF215	6.618449223	0.142904515	0.52956202	0.269854163	0.787272452	0.838156023
BRWD3	120.4995129	-0.043156072	0.160090551	-0.26957289	0.787488859	0.8382078
LETMD1	2.887895734	-0.194310545	0.720983045	-0.269507787	0.78753895	0.8382078
PRIM1	12.77070642	0.112947552	0.419210196	0.2694294	0.787599264	0.8382078
PYGO1	35.01513204	0.064306115	0.238501863	0.269625209	0.787448604	0.8382078
RPL36	440.5692658	0.044355554	0.165193185	0.268507166	0.78830896	0.838896302
FBXL4	2.20362943	-0.220346516	0.822510673	-0.267895023	0.788780125	0.839273796
RHBDD1	7.199901542	-0.120771291	0.450835735	-0.267883138	0.788789273	0.839273796
PPIH	11.91410073	-0.100709021	0.376764235	-0.267299843	0.789238308	0.839684727
MOSPD2	18.95724565	0.085504292	0.320516531	0.266770304	0.78964602	0.840051632
EPN2	36.12332123	-0.068548122	0.257052998	-0.266669216	0.789723858	0.840067575
REXO4	8.137344339	0.114261472	0.429339431	0.26613319	0.790136635	0.840439779
ADM5	1.751544494	0.248109926	0.933514674	0.265780424	0.790408321	0.840661861
ZNF358	9.021888608	-0.118024886	0.444446048	-0.265555036	0.790581919	0.840779593
ANAPC10	26.30584377	0.07110806	0.268021902	0.265306899	0.79077305	0.840915951
TTYH2	16.87827939	-0.096751586	0.364936986	-0.265118609	0.790918092	0.841003279

TMEM109	10.68903135	0.108910378	0.411066778	0.264945708	0.791051286	0.841077996
LOC132657204	3.610360307	-0.167146683	0.632649944	-0.264200897	0.79162512	0.84162117
NF1	23.01301705	-0.077050557	0.292171994	-0.263716437	0.791998429	0.841934725
PIK3C2B	21.29463339	0.084774232	0.321535108	0.263654668	0.79204603	0.841934725
ALKBH1	4.505062011	-0.157013638	0.595965528	-0.263460939	0.792195327	0.841984746
FBXO8	10.15242106	-0.132842944	0.504660487	-0.263232307	0.792371531	0.841984746
GPR156	2.078747286	-0.244126431	0.927128135	-0.263314662	0.79230806	0.841984746
RRN3	13.93672943	-0.107268202	0.407577325	-0.263184912	0.79240806	0.841984746
RTTN	2.503137296	0.215130468	0.817329372	0.263211473	0.792387589	0.841984746
CRYL1	37.15380512	-0.085406394	0.32465825	-0.26306553	0.792500072	0.842015577
SMARCE1	294.0825687	0.04199876	0.160052841	0.262405588	0.793008765	0.842439531
TICRR	3.020593103	-0.194112741	0.739803098	-0.262384331	0.793025152	0.842439531
LARS2	40.89700218	-0.092274413	0.352311445	-0.261911482	0.793389687	0.842697157
PSPC1	94.58742993	0.044633491	0.170417848	0.261906197	0.793393762	0.842697157
CWF19L1	4.920525959	-0.144379803	0.551987011	-0.261563768	0.79365778	0.842910599
MAPT	4.652133637	-0.152798095	0.585529693	-0.260957039	0.794125638	0.843340479
SEL1L3	37.76373138	0.098914021	0.379977639	0.260315374	0.794620515	0.843798983
LOC101108147	2.238152234	-0.21652275	0.83359876	-0.259744569	0.795060811	0.84419946
SRA1	13.72953379	0.089766875	0.345729486	0.259644835	0.795137749	0.844214088
MRPL21	56.74964025	-0.048804213	0.188123348	-0.259426667	0.795306057	0.844325715
SIAH1	24.47355259	0.070859661	0.27337743	0.259200844	0.79548028	0.844443604
SAMD5	1.642701785	0.270197255	1.043425532	0.258952122	0.795672182	0.84458024
MRPS31	47.34947409	0.055091284	0.212978797	0.258670273	0.795889658	0.844743998
PHLDB1	19.17454592	-0.101103255	0.391016957	-0.258564887	0.795970978	0.844763228
AKAP11	74.78182995	-0.045725776	0.177106623	-0.258182193	0.7962663	0.845009557
CCDC9	10.1973283	0.098299018	0.381694419	0.257533285	0.796767124	0.845383285
CLK3	15.5568488	-0.091330295	0.354549749	-0.257595148	0.796719375	0.845383285
PAPOLB	2.030524427	0.234009945	0.908846908	0.257480047	0.796808216	0.845383285
HNMT	11.01916049	-0.102336636	0.398270733	-0.25695244	0.797215488	0.845748251
LOC105601982	6.758387902	0.127453955	0.496826656	0.256536064	0.797536937	0.84595498
LOC105605611	4.236559072	0.178056091	0.693930124	0.256590808	0.797494671	0.84595498
ROS1	6.761463994	0.121331286	0.473457935	0.256266242	0.797745262	0.846108806
CPSF7	10.97832351	-0.106119151	0.414573111	-0.255972103	0.797972378	0.846282537
FAM161B	7.452463254	0.120155792	0.470831467	0.255199155	0.798569284	0.846848385
LOC105607811	1.947387581	-0.218879734	0.858391143	-0.254988341	0.798732104	0.846953852
FAM89B	19.19908225	0.073965453	0.290593031	0.254532782	0.799083981	0.847259757
NBR1	140.6990487	-0.041941209	0.164886392	-0.254364283	0.799214141	0.847263342
TP53I13	8.240913494	-0.11222775	0.441130273	-0.254409541	0.799179181	0.847263342
ATP13A3	19.70101691	0.079961416	0.315856013	0.253157809	0.800146268	0.848166058
CZIB	4.696713585	-0.147159742	0.581622828	-0.253015761	0.800256034	0.848166058
THSD4	7.296645774	0.129933527	0.513479616	0.253045151	0.800233323	0.848166058
STXBP4	10.8489089	0.102839864	0.407012528	0.252670022	0.800523215	0.848381961

RSPH9	6.923787974	-0.129716023	0.514560739	-0.25209079	0.800970889	0.848789098
TFPT	2.907941488	0.215370956	0.854682966	0.251989293	0.80104934	0.848804937
BARX2	4.704317535	0.171752065	0.684270571	0.251000222	0.801813938	0.849480429
INO80C	3.161452412	0.176578629	0.703376685	0.251044189	0.801779945	0.849480429
RSPH3	10.04881934	-0.105092689	0.419566867	-0.250478999	0.802216943	0.84984003
ARHGAP5	178.3508374	0.040093071	0.160389539	0.249973101	0.80260815	0.850119703
ARRDC2	3.143844126	-0.200260454	0.800897722	-0.250044979	0.802552565	0.850119703
MAN2A1	22.41007338	-0.070250022	0.281397532	-0.249646903	0.802860423	0.850319526
COPS3	48.34854384	0.060152259	0.241371521	0.249210257	0.803198146	0.85054242
WFDC2	8.682157958	-0.158003927	0.633850618	-0.249276284	0.803147075	0.85054242
FLRT3	25.33213855	-0.066163844	0.266340878	-0.248417908	0.80381108	0.851056629
TRIM26	18.86460866	-0.090797275	0.365412287	-0.248478987	0.803763826	0.851056629
ARMH1	9.201319177	0.113469532	0.45743553	0.248055791	0.80409124	0.851059432
BAX	11.79154579	0.094262579	0.379995801	0.248062159	0.804086313	0.851059432
INF2	5.321492667	0.143388053	0.578170663	0.24800299	0.804132094	0.851059432
LOC114113138	1.745125419	0.250578654	1.009325735	0.248263415	0.803930603	0.851059432
UBE2L6	16.22228049	0.078601472	0.31689775	0.248034175	0.804107965	0.851059432
NR4A2	21.46960369	0.085990136	0.347153805	0.247700398	0.804366224	0.851239822
PTOV1	9.046088525	-0.112850076	0.456137201	-0.247403798	0.804595735	0.851415296
PDE7A	33.41143313	-0.083794844	0.340433913	-0.246141295	0.805572858	0.852381793
WRN	30.71525173	0.064389157	0.262070801	0.245693747	0.805919313	0.852680879
DCAF5	26.92685466	-0.061905399	0.252926359	-0.244756614	0.806644889	0.853332054
MCOLN1	9.742525086	-0.100473925	0.410582102	-0.244710924	0.806680269	0.853332054
TANC1	24.04346091	-0.06482282	0.264959865	-0.244651469	0.806726308	0.853332054
FHIT	2.093157402	-0.222469482	0.909887413	-0.244502209	0.806841891	0.853386778
KLC4	8.552145871	0.103086761	0.423411324	0.243467181	0.807643506	0.854167044
SLC25A22	6.88707561	0.120715223	0.496285812	0.243237303	0.807821571	0.854287769
GALT	5.461137126	0.128484739	0.529412686	0.242692974	0.80824325	0.854666083
TRAK1	16.81450049	-0.077917335	0.321359764	-0.242461389	0.808422672	0.854788184
SLX4IP	7.152467049	-0.121423959	0.501430408	-0.242155156	0.808659941	0.854971426
RFC1	88.73346754	0.046058805	0.190458092	0.241831703	0.808910572	0.855168766
GFUS	12.27175648	0.090185748	0.373719882	0.241319106	0.809307804	0.855521046
PARG	18.2110076	-0.077233946	0.320585557	-0.240915239	0.809620811	0.855784244
ARHGAP19	8.269223149	-0.108465233	0.450897391	-0.240554137	0.8099007	0.856012395
GDF11	1.644793103	0.256546114	1.067946123	0.240223836	0.810156736	0.856215303
DERA	19.46869627	0.07874973	0.3283023	0.239869567	0.810431375	0.856437836
USP2	3.100019795	-0.170607074	0.716707495	-0.238042822	0.811847882	0.857866929
COMMD2	2.801818374	-0.179868342	0.757313971	-0.237508284	0.812262493	0.858169353
DEPDC5	4.168014522	-0.144822143	0.609721402	-0.237521829	0.812251987	0.858169353
CKAP4	185.5399058	0.044012747	0.185630073	0.237099226	0.812579813	0.858420846
NAIF1	9.652306957	0.111426148	0.470081422	0.237035846	0.812628981	0.858420846
NOTCH4	2.261984166	-0.194149633	0.821568244	-0.236315893	0.813187555	0.858943011

PAICS	1.864686711	0.238590009	1.009979974	0.236232416	0.813252327	0.858943548
ZPBP2	3.779138232	-0.147693226	0.626073255	-0.235904065	0.813507113	0.85914476
FMN1	58.47895813	0.052984613	0.225477805	0.234988152	0.814217929	0.859827514
ANAPC2	7.402212764	0.107989173	0.460092589	0.234711828	0.814432407	0.85998606
VEZT	43.01307788	0.058019331	0.24745518	0.234464	0.814624778	0.860121241
RBBP4	61.34337302	-0.045378851	0.193692288	-0.23428321	0.81476512	0.860201469
CEBPG	38.45773684	0.05972482	0.255189559	0.234041	0.814953149	0.860332027
LOC121820606	3.41221496	0.148648932	0.635833641	0.233785887	0.815151206	0.86047315
DHX30	11.85567479	-0.098422262	0.421833407	-0.233320217	0.815512761	0.860786825
LOC121816463	1.890929416	-0.236163779	1.012728959	-0.233195443	0.815609644	0.860821107
DHX33	4.994074093	0.130685906	0.561450947	0.232764601	0.815944202	0.861106215
TMEM38A	1.221675437	0.26977038	1.160063867	0.232547869	0.816112512	0.861215841
RAB19	2.552833533	-0.174111043	0.750907472	-0.231867506	0.816640923	0.861705422
CA11	1.625048992	0.253806335	1.095016756	0.23178306	0.816706515	0.861706605
TRIM39	5.058927661	0.137288765	0.594607997	0.230889537	0.817400619	0.86237088
POU2AF2	4.784736986	-0.133671545	0.581209145	-0.229988715	0.818100539	0.863041184
LOC132659393	3.508222757	-0.157167951	0.684491571	-0.22961269	0.818392745	0.863281306
EFCAB7	4.197882032	0.13986788	0.610244527	0.229199728	0.818713684	0.863483556
MIDN	63.54626358	-0.05602596	0.244436582	-0.229204481	0.81870999	0.863483556
UBL4A	41.37333554	0.052847574	0.231450384	0.228332195	0.819387997	0.864126556
SPDL1	17.86761265	0.073871829	0.324925454	0.227350084	0.820151532	0.864795312
ZKSCAN2	1.696687511	0.204097454	0.897632416	0.227373088	0.820133645	0.864795312
LOC101106720	1.907200474	-0.236355504	1.040857081	-0.227077769	0.82036327	0.864882116
TMEM87B	30.09696653	-0.064777777	0.285172155	-0.227153234	0.820304591	0.864882116
MAST3	9.375414985	-0.094971005	0.419831123	-0.226212397	0.821036229	0.865474707
NAA80	4.924785842	0.132199436	0.584465929	0.226188438	0.821054862	0.865474707
BLOC1S5	16.6438506	0.072950642	0.322719885	0.226049417	0.821162986	0.865520422
TEC	13.74493699	0.081405396	0.360378973	0.225888306	0.821288293	0.865584239
EPHX2	16.47471475	0.091762042	0.406978139	0.225471672	0.82161236	0.865720994
PICK1	6.515241228	-0.114605857	0.508239223	-0.225495892	0.821593521	0.865720994
THAP1	5.259007171	0.125338334	0.555624039	0.225581194	0.821527169	0.865720994
GPC1	9.177605782	0.115335344	0.517350809	0.222934499	0.823586485	0.867595915
RAC1	115.3828263	0.03755459	0.168451188	0.222940488	0.823581825	0.867595915
ZCCHC8	70.79056511	0.039514626	0.177219086	0.222970487	0.823558476	0.867595915
INPP5K	13.05817556	0.088632095	0.398684634	0.222311289	0.824071565	0.8680385
TOPBP1	56.50916209	-0.049957755	0.225462628	-0.221578872	0.824641732	0.868570637
TMEM209	5.484639677	-0.119614499	0.54186539	-0.220745782	0.825290382	0.869185348
KDM2B	30.69113586	-0.05271882	0.239954679	-0.219703238	0.826102282	0.869780674
LIPA	16.12893208	0.07186459	0.327122333	0.219687201	0.826114774	0.869780674
LOC105614936	2.089373996	-0.193151169	0.878329317	-0.21990746	0.825943226	0.869780674
LOC121820220	3.216008025	-0.156117086	0.710638351	-0.219685703	0.82611594	0.869780674
CMAS	117.960651	-0.040434982	0.184215848	-0.219497846	0.826262258	0.869866205

PI4KA	47.48142993	-0.050394647	0.229749571	-0.219345988	0.826380541	0.86992221
EIF4EBP3	30.78406529	0.080574372	0.367567949	0.219209461	0.826486886	0.869965641
SMIM20	36.85049833	0.050698302	0.231473838	0.219023897	0.826631432	0.870049273
GABARAPL1	88.14468293	-0.047624228	0.218144703	-0.218314849	0.827183806	0.870493563
THRAP3	571.886324	-0.038256336	0.175178081	-0.218385406	0.827128836	0.870493563
PRPS2	15.30918766	0.072003659	0.330445454	0.217898774	0.827507982	0.870560542
SEZ6	3.276811639	-0.163985759	0.752412628	-0.21794658	0.827470734	0.870560542
SYAP1	74.46338969	-0.049810895	0.228526002	-0.217965986	0.827455613	0.870560542
TULP4	41.97939256	-0.056834434	0.260669256	-0.218032749	0.827403595	0.870560542
TMEM248	61.8854832	-0.072954955	0.335166671	-0.217667691	0.827688039	0.870681436
F10	16.22220547	0.081378384	0.374075253	0.217545488	0.827783261	0.870713077
KIDINS220	73.7613428	0.041068947	0.189136013	0.217139753	0.828099435	0.870977105
MFSD3	3.675565922	-0.164636137	0.760096882	-0.216598885	0.828520955	0.871351883
CFAP36	22.60360938	0.069864316	0.3232221	0.216149564	0.828871167	0.871586158
IL17REL	1.752289239	0.203584326	0.941884438	0.216145758	0.828874133	0.871586158
GRAMD4	3.978617041	0.13735758	0.636301082	0.215868845	0.829089983	0.871675978
HNRNPR	41.80234678	0.044663759	0.206856802	0.21591632	0.829052976	0.871675978
COG6	10.8820728	-0.08761767	0.406552929	-0.215513562	0.829366939	0.871898577
AMER1	9.367008882	-0.099932818	0.464000279	-0.215372324	0.829477046	0.871945749
RABGEF1	15.63725999	0.073230928	0.340245242	0.215229838	0.829588128	0.871993939
ASF1A	24.41881266	0.054837381	0.255084529	0.214977292	0.829785023	0.871995174
LOC121818520	1.487499911	0.224615177	1.044123839	0.21512312	0.829671329	0.871995174
MED18	5.618130882	-0.111648227	0.519304866	-0.214995533	0.829770801	0.871995174
PIGF	4.846342768	0.122740299	0.572634853	0.214343047	0.830279552	0.872446265
STX7	36.02719485	0.05842851	0.273103591	0.213942666	0.830591768	0.872705728
INPP5F	6.64478259	0.100911584	0.472532648	0.213554735	0.830894301	0.872818149
PAWR	105.1792984	-0.041469322	0.194185036	-0.213555704	0.830893546	0.872818149
WRAP53	3.446045953	0.164185144	0.76882166	0.213554265	0.830894668	0.872818149
IBA57	11.5430887	0.085703584	0.401854106	0.213270396	0.831116063	0.872982106
DUSP28	5.326978419	0.120639819	0.566172702	0.21307954	0.831264923	0.873069854
FBF1	11.66077747	-0.085210893	0.400621709	-0.212696644	0.831563585	0.873314911
SLC22A16	12.9624663	0.100007695	0.471314108	0.212189055	0.831959545	0.873662106
NCKAP1	44.38260488	-0.051354744	0.242244144	-0.211995814	0.8321103	0.873683132
SEC14L1	14.11013675	0.073064345	0.344545701	0.212059954	0.832060262	0.873683132
CBLB	21.39211338	0.062237534	0.293857805	0.21179473	0.83226718	0.873779211
LOC105603000	7.538748349	0.125519445	0.593267301	0.211573173	0.83244004	0.87389205
ZFAND4	2.6713351	0.172388751	0.817331118	0.210916662	0.832952302	0.874361146
LOC114114509	17.40863141	-0.068470688	0.325767913	-0.21018242	0.8335253	0.874893918
RNF166	20.84364991	0.061630162	0.294243382	0.209453009	0.834094615	0.875422742
PSEN2	3.077168108	-0.149704371	0.715099834	-0.209347512	0.834176964	0.875440429
S100A1	1.83469694	0.19186182	0.917521891	0.209108711	0.834363374	0.875567313
RWDD3	10.90257757	-0.089633031	0.430898718	-0.20801415	0.835217919	0.876395251

CMTM6	147.0308054	0.051005934	0.245351416	0.207889298	0.835315406	0.876428739
COBL	7.706195129	0.099905907	0.481663448	0.207418494	0.83568304	0.876745645
KIAA1958	11.39806573	0.080928432	0.390455086	0.207266943	0.835801389	0.876800986
KLF3	46.69767996	0.051063173	0.247092804	0.206655848	0.836278641	0.877189972
NIFK	69.78513122	-0.045244568	0.21897044	-0.206624093	0.836303443	0.877189972
MTMR14	24.7670235	0.062403876	0.302796639	0.206091707	0.836719276	0.87755727
CHMP7	9.084136081	0.098100219	0.477044592	0.205641613	0.83707087	0.87785714
ABL2	21.51975424	-0.059438131	0.289303102	-0.205452794	0.837218376	0.877942948
LOC106991594	10.54231125	-0.087808427	0.428107222	-0.205108493	0.837487361	0.87815612
CDC45	21.10523115	0.063094345	0.307890052	0.204924924	0.837630782	0.878237609
POLG2	5.660575277	-0.107744369	0.526785803	-0.204531649	0.837938063	0.878490873
LOC114111287	2.634940268	0.160959283	0.788217929	0.204206575	0.838192074	0.878688256
IKZF4	2.176048658	0.165492721	0.81187868	0.203839225	0.838479139	0.878920255
NSMF	7.549706686	-0.091840621	0.451745318	-0.203301766	0.838899176	0.879276459
QSOX2	6.535289498	-0.11867737	0.583938524	-0.203236068	0.838950523	0.879276459
FGD1	11.36177509	0.079228629	0.39113613	0.202560241	0.839478769	0.879761112
C11H17orf75	4.446106615	-0.129939276	0.642414092	-0.202267163	0.839707869	0.879932213
BCL9L	110.9133076	-0.031995244	0.15838517	-0.202009086	0.839909621	0.879995089
DEF8	5.799064779	0.101314155	0.501709823	0.201937755	0.839965386	0.879995089
LOC101108030	4.390426465	0.124344846	0.615293345	0.202090348	0.839846093	0.879995089
C7H15orf61	1.707343478	-0.216796169	1.074286059	-0.20180488	0.840069267	0.880034941
PDCD6IP	163.5781007	-0.034875194	0.173127071	-0.201442752	0.840352389	0.880262541
AGPAT2	7.962709179	-0.112052969	0.556775128	-0.201253546	0.840500324	0.880305315
ZNF282	3.949827353	0.121459023	0.603606905	0.201222056	0.840524946	0.880305315
LOC105608034	5.397862266	0.117496462	0.585586828	0.200647379	0.840974308	0.880706934
DCAF12	57.88790778	0.038356699	0.191647816	0.200141592	0.841369846	0.880996602
TMEM168	14.68660084	-0.07056769	0.352617866	-0.200125112	0.841382734	0.880996602
ORC3	3.628606347	0.128053298	0.640218523	0.200014984	0.841468862	0.881017767
LOC132657695	1.896264757	0.174139891	0.87181519	0.199744043	0.841680767	0.881170606
PMF1	2.570596903	0.153944452	0.771313385	0.199587424	0.841803264	0.881229827
DHRS13	2.576093371	-0.155899651	0.782287612	-0.199286871	0.842038348	0.881406889
ADHFE1	3.824456003	0.125223399	0.631631724	0.198253815	0.842846484	0.882015945
GPN1	15.98129891	0.064969257	0.327954354	0.198104572	0.842963248	0.882015945
LOC121817232	5.459390739	0.111039722	0.559760545	0.19837004	0.842755556	0.882015945
MANBA	3.094453772	0.140513058	0.708073609	0.19844414	0.842697586	0.882015945
POC5	5.060079146	-0.110855359	0.559454833	-0.198148899	0.842928567	0.882015945
TMEM167A	131.891626	0.031003667	0.156554945	0.198036971	0.843016138	0.882015945
KCNK5	18.19725256	0.069772809	0.352858328	0.197736041	0.84325159	0.882193233
ETHE1	15.23625305	0.06907918	0.349561354	0.197616753	0.843344927	0.882202302
LOC132658681	3.191890289	0.134932661	0.68300881	0.197556253	0.843392265	0.882202302
MAPKAP1	25.41791239	0.052397396	0.26536719	0.19745243	0.843473504	0.882218237
HERPUD2	12.54499674	-0.076821786	0.389444884	-0.197259712	0.843624305	0.882303326

LRPPRC	64.37884755	-0.037817811	0.191793588	-0.197179748	0.843686878	0.882303326
BCL6	50.81187674	-0.041652969	0.211385475	-0.197047452	0.843790404	0.882306266
BFAR	7.503084819	-0.094208069	0.478481836	-0.196889541	0.843913979	0.882306266
TMCO6	12.73922038	-0.071734471	0.36435398	-0.196881262	0.843920457	0.882306266
WWP2	35.71512354	0.061463083	0.312250944	0.19683874	0.843953734	0.882306266
LOC121819105	2.116834626	-0.173106299	0.882115236	-0.196240006	0.844422316	0.882617539
LRCH3	14.01797635	-0.071287416	0.363330974	-0.196205172	0.84444958	0.882617539
ZNF629	22.36414654	-0.066429896	0.338353014	-0.196333101	0.844349455	0.882617539
RBL1	23.1906297	0.055094578	0.281350922	0.195821564	0.844749831	0.882862322
PCNX3	8.239490809	-0.099720826	0.509466722	-0.195735702	0.844817039	0.88286353
LOC114116649	1.861529836	-0.189567635	0.970695305	-0.195290565	0.845165483	0.883158615
RANBP6	16.57593159	-0.061849691	0.316906506	-0.195166996	0.845262216	0.883190649
SNAPC5	29.57725355	-0.060292206	0.310064017	-0.194450831	0.845822892	0.883688175
TYW3	4.600282599	-0.12841129	0.66058626	-0.19438989	0.845870605	0.883688175
AVPI1	3.972182881	0.151988003	0.782253826	0.194294994	0.845944905	0.883696725
CCDC85C	25.98081198	-0.054584149	0.281213891	-0.194101894	0.846096099	0.883785594
BPNT1	5.58223042	0.103569666	0.534153621	0.193894905	0.846258173	0.883885813
RNPS1	321.1610039	-0.030453208	0.157457927	-0.193405363	0.846641516	0.884217104
DOCK5	29.82875405	0.056977557	0.294746294	0.193310514	0.846715793	0.884222344
EPN3	7.824763345	0.085325309	0.44157381	0.193230003	0.846778842	0.884222344
LOC132657765	4.845749012	0.105975615	0.549396122	0.192894727	0.847041415	0.884427432
TRIM16	3.155091903	0.135881302	0.709738388	0.19145266	0.848170966	0.885537661
NUDT6	56.35361563	0.038135014	0.199368265	0.191279259	0.84830681	0.885610312
FER	21.26678345	-0.061276629	0.320978501	-0.190905712	0.848599465	0.885846645
LOC132657417	30.7028268	0.048526987	0.254350842	0.190787603	0.848692002	0.885874057
DYNC2H1	51.6108017	0.052278692	0.275096608	0.190037575	0.849279686	0.886418264
LOC101106719	2.895700179	-0.148733691	0.783049373	-0.189941651	0.849354854	0.886427499
MEF2D	9.785771821	-0.076026727	0.400792526	-0.18969098	0.84955129	0.886563284
ZNF746	8.281335016	0.086876812	0.458686259	0.189403563	0.849776532	0.886729107
NT5M	2.248909487	0.162320863	0.858040625	0.189176197	0.849954722	0.88684581
LOC105611720	1.655909937	-0.177303066	0.937936527	-0.18903525	0.85006519	0.886891837
LOC132658006	1.247805011	0.222380756	1.179641612	0.188515524	0.850472548	0.887247586
VARS2	4.26069784	-0.115630537	0.615299594	-0.187925586	0.850934986	0.887660737
HAUS6	49.25406781	-0.054643728	0.291267657	-0.187606576	0.851185071	0.887852322
CHD4	537.6760919	0.032420296	0.173015403	0.187383869	0.851359669	0.887965146
CCDC22	14.58308416	-0.069623237	0.372855202	-0.18672996	0.851872365	0.888406074
TGDS	43.47733557	0.042426248	0.227273168	0.18667513	0.851915356	0.888406074
NATD1	3.713023127	0.125086262	0.673893144	0.185617354	0.852744844	0.889201715
FAM83H	24.47350672	-0.062621842	0.337796648	-0.185383254	0.852928442	0.889323782
ARL15	3.185692186	0.122592319	0.662806348	0.184959482	0.853260816	0.889531555
NELFB	39.26242812	-0.048995004	0.264836682	-0.185000824	0.853228389	0.889531555
C5H5orf15	56.48266959	0.051433709	0.278991308	0.184355955	0.85373422	0.88991117

TEP1	8.069687199	0.078846084	0.427754624	0.184325497	0.853758113	0.88991117
MCTP2	25.23420565	0.052873181	0.287036364	0.184203773	0.8538536	0.889941299
RMC1	4.816860235	-0.106045065	0.578863992	-0.183195131	0.854644915	0.890696603
SHARPIN	3.954965391	-0.116030394	0.635643992	-0.182539906	0.85515904	0.891162929
DNAJC28	12.27173046	-0.067134666	0.368726224	-0.18207185	0.85552634	0.89133721
PLEKHH3	8.307290869	-0.082896931	0.454960533	-0.182206861	0.855420389	0.89133721
SAT2	4.898801928	0.104972802	0.576422077	0.182111002	0.855495614	0.89133721
TMEM170B	3.795143281	0.112681096	0.620748266	0.181524626	0.855955806	0.891715144
TRIB1	34.336616	-0.055396489	0.305334137	-0.181429072	0.856030801	0.89172377
LOC101114941	4.585432345	0.109231128	0.602354584	0.181340245	0.856100519	0.891726897
IKBKB	9.84145003	0.081683472	0.450823261	0.181187349	0.856220524	0.891782399
TMEM154	2.45421201	0.146117631	0.808794577	0.180660993	0.856633678	0.892143193
STK10	12.57360108	0.069086602	0.382649987	0.18054777	0.856722555	0.892166239
ARHGAP8	1.969197112	0.161256546	0.894047148	0.180366938	0.856864509	0.892244548
LOC101111215	63.0329329	-0.046939622	0.261193815	-0.179711843	0.857378795	0.892686918
LOC121817230	13.09694069	-0.067901472	0.377953444	-0.17965565	0.857422913	0.892686918
IQANK1	3.055950857	0.133403719	0.744696162	0.179138453	0.857828989	0.892904103
LOC105615111	5.516777322	-0.094389338	0.526918203	-0.179134707	0.857831931	0.892904103
LOC121819029	5.72169587	0.098126432	0.547473452	0.17923505	0.857753143	0.892904103
IK	130.8789085	0.028881905	0.162553284	0.17767654	0.85897701	0.893969713
ZDHHC16	4.546831527	0.10632607	0.598478284	0.177660698	0.858989453	0.893969713
ERCC1	6.93537667	-0.09462332	0.535323957	-0.176758987	0.859697705	0.894637148
CLPTM1	6.967553759	-0.09265361	0.526189622	-0.176084069	0.860227897	0.895049515
SBSN	13.94628042	-0.072907928	0.413962469	-0.176122074	0.86019804	0.895049515
WDR45	2.842178153	0.136069919	0.774048056	0.175790014	0.860458915	0.895220196
KLHDC4	24.12635371	0.055004379	0.31365743	0.175364503	0.860793231	0.895498313
MEX3B	1.640190609	-0.169679808	0.968856774	-0.175134047	0.860974306	0.89561698
LOC101110006	276.1232927	0.026970557	0.154618682	0.174432718	0.861525403	0.896120509
P4HTM	2.259898398	0.152027445	0.874099844	0.173924576	0.861924738	0.896466115
LZTFL1	4.173323134	0.106990096	0.61564269	0.173786025	0.862033628	0.896509607
LYSMD4	3.732679127	-0.112313891	0.647649555	-0.173417692	0.862323119	0.896740902
DCLRE1B	1.530847666	-0.16445389	0.951568356	-0.172824043	0.862789738	0.897021423
GNAQ	109.2614949	-0.035259596	0.204026916	-0.172818353	0.86279421	0.897021423
LRRC24	15.34831781	0.061421493	0.355246596	0.172898189	0.862731455	0.897021423
B3GNT6	17.88467402	0.075409895	0.438419349	0.172004031	0.86343436	0.897617146
TMED4	31.80026427	0.051836398	0.30166099	0.171836597	0.863565993	0.89768417
LOC121820628	4.169599474	0.111134844	0.648428305	0.171391105	0.863916249	0.897978426
TTC9	3.187717522	-0.124836625	0.729547747	-0.171115085	0.864133275	0.898134164
ESD	14.2761071	0.060791943	0.356227204	0.170654971	0.864495071	0.898166843
GGA3	7.989636532	0.078337595	0.459285569	0.170564025	0.864566587	0.898166843
IFT25	4.65319507	-0.095513121	0.558741539	-0.170943297	0.864268352	0.898166843
LOC121819593	1.434055864	-0.175686224	1.028292443	-0.170852393	0.864339831	0.898166843

SARNP	13.65874729	0.062542191	0.366586707	0.170606816	0.864532938	0.898166843
UBTF	73.50984352	-0.034935923	0.204827869	-0.170562351	0.864567903	0.898166843
CCNE1	3.469941375	-0.112877042	0.662316661	-0.170427604	0.864673865	0.898207109
LOC132659274	10.21186909	-0.069229567	0.406800172	-0.17018077	0.864867974	0.898338929
TGFA	4.936690601	-0.098559705	0.579459712	-0.170088969	0.864940169	0.898344106
ANAPC5	24.7644928	-0.047244669	0.278326235	-0.169745655	0.865210168	0.898345304
CCDC18	76.24789651	0.038263537	0.225348138	0.169797439	0.865169441	0.898345304
SAMD1	14.83032638	0.058586882	0.344762883	0.169933844	0.865062164	0.898345304
ULK1	4.653121773	-0.108032712	0.636155833	-0.169821145	0.865150797	0.898345304
REEP4	66.20328178	-0.039235409	0.231864115	-0.169217255	0.865625758	0.898706997
CEBPZOS	35.57182798	0.04351918	0.257475455	0.169022634	0.865778838	0.898796113
DAPK1	10.65971003	-0.065111003	0.387777322	-0.167908228	0.866655477	0.899636308
LOC114110852	2.222529452	0.143350987	0.854421622	0.167775467	0.866759923	0.899674857
ERO1B	2.675813434	0.126561546	0.759931076	0.166543454	0.867729289	0.900611095
NOL12	4.931093862	-0.093069669	0.560460119	-0.166059396	0.868110208	0.90089062
PPP3CA	175.6782564	-0.026997099	0.162603828	-0.16602991	0.868133412	0.90089062
KCTD2	30.66829711	-0.057065678	0.345490448	-0.165172954	0.868807852	0.901520515
SLC18A2	1.173976935	-0.181193782	1.101054729	-0.164563829	0.869287303	0.901947997
IRS1	9.490082792	-0.071202517	0.433974394	-0.164070779	0.869675425	0.902093433
MBIP	18.37634773	0.051535427	0.313761419	0.164250362	0.869534056	0.902093433
NPLOC4	18.41753237	-0.054587787	0.332765487	-0.164042813	0.86969744	0.902093433
SAYSD1	4.361014077	0.100350684	0.611355339	0.164144611	0.869617304	0.902093433
ZNF274	10.1747543	0.07113568	0.434398056	0.163756901	0.869922521	0.90225688
ARL13B	53.14936308	0.034512241	0.211211422	0.163401395	0.870202404	0.902337109
MRPL41	79.9249337	-0.031488661	0.192571188	-0.163516989	0.870111398	0.902337109
WWC3	4.94195537	0.090827199	0.555789694	0.163420085	0.87018769	0.902337109
PTS	10.66405293	-0.06761896	0.414236306	-0.163237648	0.870331325	0.902400783
LOC121818611	9.061549155	0.069823988	0.428092147	0.163105043	0.870435729	0.902439029
RDH11	15.34615854	0.057531737	0.353488254	0.162754312	0.870711882	0.902655318
ACOT7	5.711755395	-0.105301356	0.648684964	-0.162330502	0.871045596	0.902905644
NAT8	9.306116501	0.068108746	0.419880608	0.162209791	0.87114065	0.902905644
RBM42	28.31721381	-0.050472147	0.311190927	-0.16219029	0.871156006	0.902905644
LOC114115026	1.986978525	0.148620106	0.920773431	0.1614079	0.871772145	0.903356329
RIMOC1	8.183700445	-0.089152835	0.552163163	-0.161461034	0.871730299	0.903356329
ZC2HC1C	9.371228673	0.063893805	0.395919854	0.161380654	0.871793603	0.903356329
RPS5	389.2919629	-0.033590984	0.208585948	-0.16104145	0.872060754	0.903563104
ZNF628	2.858668947	-0.113024171	0.706157901	-0.160055096	0.872837673	0.904297987
ATP1B3	9.624988605	-0.087272827	0.545611271	-0.159954223	0.872917135	0.904310217
LOC101102399	1.865218749	-0.148402424	0.929130238	-0.159721876	0.873100168	0.904429733
LOC105603698	1.6130858	0.166614189	1.044889777	0.159456234	0.873309437	0.904576406
TAFAZZIN	5.553795016	-0.090513744	0.569242266	-0.159007419	0.873663028	0.904872533
MTMR2	11.58386501	-0.068212307	0.431230418	-0.158180648	0.874314452	0.905336765

SYTL5	33.79476119	0.041079235	0.259632985	0.158220402	0.874283128	0.905336765
TMOD1	1.450336536	-0.162922633	1.029438826	-0.15826354	0.874249137	0.905336765
SUV39H2	2.619370023	0.123677363	0.783656174	0.157820952	0.874597888	0.905560104
LOC105605940	13.37808516	0.058069743	0.369125186	0.157317206	0.874994861	0.905900953
RC3H1	58.98176475	-0.032256039	0.205217064	-0.157180103	0.875102909	0.905942645
ZBTB5	5.261615357	0.082348455	0.525740849	0.156633168	0.875533961	0.90631869
TRUB2	13.11263686	-0.055951967	0.35848436	-0.156079242	0.87597056	0.906700419
C7H15orf62	10.44810258	0.081071718	0.525749704	0.154202119	0.877450372	0.908091496
HADHB	22.16055457	0.049693128	0.32222263	0.154219857	0.877436386	0.908091496
DEPDC7	5.698352945	0.079808524	0.520122186	0.153441877	0.878049823	0.908518829
LATS2	14.85972709	0.0541828	0.353166847	0.153419837	0.878067203	0.908518829
RPP25L	13.67495466	0.056721692	0.369393653	0.153553509	0.877961796	0.908518829
FXYD5	3.60289268	-0.119821632	0.782384889	-0.153149216	0.878280605	0.908669291
FBXO3	3.989288698	-0.101324031	0.663860027	-0.152628607	0.878691164	0.908953339
GPHN	4.17189151	-0.09768724	0.640025823	-0.152630154	0.878689944	0.908953339
ELP6	6.628736365	-0.079155864	0.519675314	-0.152317922	0.87893619	0.909135311
IP6K2	10.73699697	-0.065179411	0.428202755	-0.152216235	0.879016389	0.909135311
VBP1	12.04525317	-0.058757076	0.386186671	-0.152146825	0.879071133	0.909135311
ESCO2	3.314814809	0.108513994	0.713649467	0.152055034	0.87914353	0.909139839
LOC101118248	18.06570147	0.047847808	0.315339838	0.151734106	0.879396656	0.909260904
LOC121818373	1.426474657	0.160821333	1.059546329	0.1517832	0.879357933	0.909260904
URM1	15.76574738	-0.055492665	0.366901585	-0.15124673	0.879781089	0.909588029
LOC121817141	3.520021704	0.10857893	0.721651803	0.150458891	0.880402581	0.910160175
TP53INP2	6.717053132	-0.073457734	0.4892058	-0.15015712	0.880640656	0.910335886
TMEM59	23.63022545	0.044479061	0.297564118	0.149477232	0.881177073	0.910819949
CLCN3	56.19076092	-0.051860909	0.347173782	-0.149380257	0.881253589	0.910828601
SDHAF1	2.732686752	0.122081372	0.821900308	0.148535498	0.881920173	0.911447076
INTS6L	5.157438106	0.079903355	0.538785167	0.148302811	0.882103797	0.911566364
CIAO3	2.51702903	0.114013582	0.777059628	0.146724367	0.883349585	0.912712627
LOC101106962	2.377819076	0.122904283	0.837223457	0.146799856	0.883289998	0.912712627
SSTR2	3.007325032	0.109689394	0.749643991	0.146321982	0.883667214	0.912970239
ECSIT	6.163806409	-0.071861068	0.493382682	-0.145649757	0.884197886	0.913447901
R3HDM1	54.86267557	-0.035085402	0.241136975	-0.145499884	0.884316208	0.913499531
TPCN2	11.19085147	-0.070322032	0.484533424	-0.1451335	0.884605469	0.91372772
KLHL36	5.299723722	0.079930341	0.553458208	0.144419831	0.885168958	0.914239108
CTDSP2	23.00989787	-0.048297454	0.334736027	-0.1442852	0.885275265	0.914278256
MTFR1L	2.723570919	0.10685976	0.741823216	0.144050169	0.885460854	0.914388681
ZNF774	3.023737786	-0.113591128	0.788955921	-0.143976521	0.88551901	0.914388681
BCL2L12	4.883087826	-0.080446072	0.560789203	-0.143451535	0.885933585	0.914746102
TRIT1	13.12518628	-0.053612399	0.374206688	-0.143269483	0.886077356	0.91482388
LOC132659059	13.90362217	0.049103907	0.343027927	0.143148425	0.886172961	0.914851919
EDARADD	2.937113164	-0.104923499	0.734369979	-0.142875528	0.886388486	0.915003746

TMEM38B	17.84749303	0.047755169	0.334782234	0.14264547	0.886570185	0.915120634
SVIP	14.95585266	0.045947728	0.323117429	0.142201329	0.886920982	0.915412035
TCF3	35.54876847	0.037877105	0.266689159	0.142027166	0.887058548	0.915483326
LOC105610127	18.32897132	-0.055119175	0.388825961	-0.14175796	0.887271192	0.91556697
LOC106991342	17.18298772	0.044957126	0.317155352	0.14175112	0.887276595	0.91556697
DCLK1	1.993751208	0.127950566	0.904107278	0.141521442	0.887458022	0.91568349
CHML	17.36048885	0.045977865	0.325525132	0.141242135	0.887678661	0.915840446
C5H5orf63	12.79325713	-0.053996967	0.383927919	-0.140643502	0.88815158	0.916188529
CCNP	2.827439722	0.106711187	0.759203482	0.140556767	0.888220104	0.916188529
SLC25A42	2.295662264	-0.130133422	0.925855584	-0.140554773	0.888221679	0.916188529
LRATD2	21.17400445	0.052996974	0.378094039	0.140168764	0.888526651	0.91643238
LOC132658402	24.31211692	-0.043518938	0.310858993	-0.139995752	0.888663347	0.916502646
SUPT7L	4.283092299	-0.084019238	0.602752255	-0.139392656	0.889139875	0.916923352
TCEAL8	152.8815106	-0.023244529	0.167659807	-0.138641036	0.889733814	0.917465064
SLC25A12	3.897679765	-0.082407186	0.5956594	-0.138346152	0.889966852	0.917630431
TSC22D3	7.882216664	-0.06000297	0.433972788	-0.13826436	0.890031491	0.917630431
CCNF	17.30834692	0.043144858	0.313327876	0.137698752	0.890478506	0.918020494
KDM3B	37.54809843	0.032534213	0.2372059	0.137156003	0.890907487	0.918391907
CEMIP2	25.48206938	-0.038671407	0.283494166	-0.136409888	0.891497257	0.918929
BIRC2	136.5844507	-0.024035511	0.176742869	-0.135991407	0.891828074	0.919199108
ANGEL2	21.15914747	-0.039181738	0.289181083	-0.13549205	0.892222849	0.919535092
LOC132657769	3.336065551	-0.091146372	0.674467835	-0.135138204	0.892502605	0.919752491
RAB2B	1.659394159	-0.123334038	0.917692713	-0.134395791	0.89308961	0.920286464
KRT80	3.019472899	0.101356885	0.756799797	0.133928267	0.893459298	0.920525474
MRPL19	34.11915371	-0.03025311	0.225781441	-0.133992898	0.89340819	0.920525474
ACTR1A	15.27023476	0.044548337	0.334052699	0.133357214	0.893910881	0.920912976
KLHL7	1.226464638	0.158409799	1.189340597	0.133191282	0.894042105	0.920912976
PITPNB	205.3411643	0.021371843	0.160416151	0.133227498	0.894013464	0.920912976
LOC121816030	2.953776152	-0.103609917	0.784170427	-0.132126784	0.894884011	0.921709154
DLD	57.45412959	0.024830938	0.188186957	0.131948239	0.895025233	0.921783577
CCNT2	23.27843283	0.037409615	0.283819325	0.13180785	0.895136277	0.921826911
LOC132660131	7.352241867	-0.063321634	0.480759016	-0.131711797	0.895212254	0.921834128
TAFA4	3.025176578	-0.092241505	0.700927514	-0.131599207	0.895301313	0.921854814
TRIP4	25.25997422	0.044717228	0.340115083	0.131476756	0.895398173	0.921883529
MKRN2	6.061305097	0.078866004	0.600924103	0.131241206	0.8955845	0.922004346
RFESD	11.0996991	0.052413395	0.40168142	0.130484987	0.896182731	0.922549167
AP1S3	38.86553362	0.043789258	0.336005603	0.130323	0.896310884	0.922610033
FNDC7	1.860162061	-0.125194049	0.963830157	-0.129892231	0.896651691	0.922749628
LOC132657141	7.08019457	-0.06337144	0.487644626	-0.129954145	0.896602706	0.922749628
RPLP2	1300.660479	-0.026487801	0.203925174	-0.129889804	0.896653611	0.922749628
SMYD4	1.916239539	0.120865241	0.931434666	0.129762446	0.896754375	0.922762891
UIMC1	21.06421286	-0.039211994	0.302330738	-0.129698998	0.896804575	0.922762891

IQCF1	1.050246218	-0.176566771	1.364827123	-0.12936933	0.897065415	0.922954293
LRP3	1.76826333	-0.124815837	0.96539921	-0.129289351	0.897128698	0.922954293
SIRT4	1.673498978	-0.118710367	0.920406932	-0.128975959	0.897376672	0.923138351
LOC105613289	3.69322854	-0.081912585	0.636752052	-0.128641258	0.897641519	0.923339737
LANCL2	23.17301768	-0.034065279	0.265615425	-0.128250379	0.897950833	0.923541274
USP1	142.0445119	0.023140327	0.180474979	0.128219032	0.897975639	0.923541274
IFT81	17.32100199	0.043121958	0.337344768	0.127827559	0.89828544	0.92371774
LOC132659055	92.20071016	0.021606832	0.169020979	0.127835208	0.898279387	0.92371774
DNAAF10	13.36333526	0.045336574	0.355281536	0.12760746	0.898459627	0.923825785
CXHXorf38	1.801090753	0.116954369	0.923162284	0.126688851	0.899186671	0.924455765
ZNF184	5.79614555	-0.069834941	0.55136373	-0.126658569	0.899210639	0.924455765
C5H19orf25	5.425561342	0.065270493	0.515900961	0.126517487	0.899322307	0.924499459
HACD1	3.804941137	0.0832219	0.65865236	0.126351783	0.899453468	0.924563182
VPS16	20.18709862	-0.040172332	0.318719755	-0.126042806	0.899698041	0.924743464
LOC105603397	6.547312727	-0.060672089	0.482823806	-0.125660931	0.900000329	0.924983037
GRAMD1C	26.86575007	-0.038391938	0.30599531	-0.125465773	0.90015482	0.924999563
SEC14L2	12.78981989	0.063396586	0.505136806	0.125503795	0.90012472	0.924999563
UBXN2B	10.17022832	0.051493969	0.411185005	0.12523309	0.900339021	0.925117724
D2HGDH	19.94245501	-0.042875515	0.342783626	-0.125080405	0.900459896	0.925170802
SASS6	37.06022146	0.0345675	0.276977877	0.124802387	0.900679997	0.925325814
FLCN	19.47061027	0.041657895	0.334900965	0.124388699	0.901007521	0.925591156
PHF8	7.311221872	0.059728541	0.482521095	0.123784311	0.901486054	0.925940413
VEZF1	107.5641257	0.020078848	0.16215603	0.123824242	0.901454437	0.925940413
COL27A1	28.50054326	-0.035796253	0.290036738	-0.12341972	0.901774742	0.92616576
LOC105602476	1.875026191	0.121476752	0.986420728	0.123149027	0.901989089	0.926314726
TIMM10	35.8032164	0.030880837	0.25120755	0.122929574	0.902162866	0.926422009
GAL3ST1	1.690011483	0.117069531	0.958183192	0.122178652	0.902757533	0.926961449
NINL	19.93305565	-0.036979495	0.303890496	-0.121686908	0.903146982	0.927290103
YBEY	8.412286793	0.056706348	0.466707045	0.121503089	0.903292568	0.927368343
LOC101107153	54.03670063	-0.071080953	0.585948044	-0.121309311	0.903446046	0.927454673
LRFN3	2.745906727	-0.096015437	0.792087395	-0.121218236	0.90351818	0.927457492
LOC101103720	1.918566883	0.112781306	0.931987965	0.121011548	0.903681889	0.927554302
IL1RAP	1.462179955	-0.126540895	1.047557351	-0.12079615	0.9038525	0.927602999
ITGB1BP1	18.40769543	0.039756377	0.329891299	0.120513567	0.904076333	0.927602999
NHSL1	33.62447995	0.027866745	0.231042185	0.120613233	0.903997387	0.927602999
PPP1R26	16.79556624	0.040079519	0.332552468	0.120520889	0.904070533	0.927602999
UBA7	1.337219171	0.153855906	1.275915324	0.120584731	0.904019963	0.927602999
TMEM184C	12.69176618	-0.045508401	0.378859809	-0.120119367	0.904388591	0.927852157
ABL1	9.877207975	0.052423875	0.436939223	0.119979788	0.904499159	0.927894371
IRF2BP1	2.109165382	-0.111301636	0.933065798	-0.119285946	0.905048817	0.928315747
PPP2R5B	16.52777772	0.053359351	0.44709542	0.119346673	0.905000708	0.928315747
HK2	19.9924371	0.038483003	0.323266457	0.119044218	0.905240324	0.928440928

LOC101118100	262.218957	-0.021502365	0.181769294	-0.118294817	0.905834064	0.9289786
LOC101110740	8.357660937	0.04954648	0.41938408	0.118141062	0.905955889	0.929032255
PPP1R3C	4.336315707	0.06913521	0.58576877	0.118024746	0.906048051	0.929055485
LOC101106998	3.318168591	0.078534516	0.666796464	0.117778842	0.906242894	0.929183992
CENPL	1.637996694	0.113141354	0.96264101	0.117532239	0.906438298	0.929313054
SEPSECS	20.90223948	0.049979607	0.42680039	0.117103002	0.906778431	0.929590467
MTIF2	51.93712695	0.023780119	0.203277425	0.11698357	0.906873073	0.92961619
CDKL3	4.508470227	0.068212372	0.585464709	0.116509793	0.907248525	0.929787133
CEP162	52.21845281	0.029126786	0.24974591	0.116625679	0.907156688	0.929787133
MEGF8	9.609863538	-0.053002877	0.454860624	-0.116525534	0.907236051	0.929787133
VAMP1	2.753849874	-0.083303755	0.722536804	-0.115293441	0.908212537	0.930703731
FAM210A	21.39514614	-0.033313443	0.291194083	-0.114402886	0.908918427	0.9312843
LOC114115646	1.532912961	-0.118972249	1.039604402	-0.114439924	0.908889067	0.9312843
R3HCC1	19.13717538	-0.033582731	0.295364791	-0.113699167	0.909476273	0.931784444
ARHGEF16	10.28472654	-0.047391885	0.417274998	-0.113574706	0.909574939	0.931814106
PECR	7.281827727	-0.060083784	0.531176819	-0.11311447	0.909939804	0.932116448
EGLN2	42.73280607	-0.02399087	0.21229741	-0.113005946	0.910025842	0.932133144
MECOM	167.0617171	-0.018817737	0.166761383	-0.112842292	0.910155589	0.932194605
GPATCH1	19.44873872	0.038456493	0.341544613	0.112595812	0.910351006	0.932280509
LOC105609191	1.892868806	0.105535865	0.937591937	0.112560551	0.910378962	0.932280509
ZBTB26	17.96331122	0.039207722	0.348891671	0.112377924	0.910523758	0.932357355
ZNF704	78.12616218	0.025017871	0.222818369	0.112279214	0.910602022	0.932366066
ENPP5	11.87033041	0.054432151	0.486997977	0.111770794	0.911005145	0.932707374
TP53RK	15.08470701	0.039006214	0.349495159	0.111607307	0.911134778	0.932768646
LOC101107597	2.081935393	0.107798301	0.970041986	0.111127459	0.911515274	0.933086709
PRDM10	4.952805956	-0.065216402	0.589478228	-0.110634115	0.911906494	0.933415699
C11H17orf100	2.596696509	0.08075987	0.732737814	0.1102166	0.912237598	0.933553497
COMMD10	40.59712167	0.02323937	0.210883314	0.110200138	0.912250653	0.933553497
HAUS7	20.73938103	-0.033263962	0.301465321	-0.110340922	0.912139004	0.933553497
SLC29A1	2.166164695	0.091539236	0.832841257	0.109911985	0.912479177	0.933715869
LOC101112843	8.219587323	-0.048603307	0.446004664	-0.108974886	0.91322241	0.934341941
NACC2	8.445055386	0.044868041	0.411768325	0.108964285	0.913230818	0.934341941
LOC132658188	1.992641609	-0.093697873	0.864337754	-0.108404234	0.913675042	0.934724884
TSPAN4	1.863947451	0.112351202	1.037402226	0.108300522	0.913757309	0.934737501
LOC105611449	6.367333885	0.063140587	0.583579005	0.10819544	0.913840662	0.934751227
DIMT1	11.86500335	0.04107009	0.380317135	0.10798906	0.914004371	0.934794801
KIAA1191	10.51522286	-0.044389093	0.411141833	-0.107965401	0.914023138	0.934794801
LOC114116088	28.63487145	-0.029576567	0.275073267	-0.107522506	0.914374472	0.93508257
IMPG2	7.958653034	-0.052183348	0.487363016	-0.107072852	0.914731185	0.935264943
RILPL1	3.575671805	0.074978194	0.700476281	0.107038877	0.914758138	0.935264943
ZNF646	2.028693462	-0.08739553	0.816528123	-0.107033093	0.914762726	0.935264943
ARL5B	46.67976388	-0.022443275	0.211302838	-0.106213789	0.91541273	0.935825809

FAM110B	4.992474825	0.059290526	0.55847452	0.106165141	0.915451328	0.935825809
PIGM	8.672738607	0.050541823	0.47811822	0.105709887	0.915812536	0.936123459
SPRED1	22.61004626	-0.031205303	0.29692866	-0.105093606	0.916301533	0.936551678
ZNF263	11.84886622	0.041189283	0.393685929	0.104624728	0.916673592	0.936860318
LOC121817131	9.35134621	0.044924706	0.429806972	0.104522981	0.916754332	0.936871198
TMEM14A	4.0193545	-0.067250316	0.647768359	-0.103818464	0.917313413	0.937370878
ACOT8	26.45144703	-0.030639107	0.295563093	-0.103663509	0.917436386	0.937424871
SCML2	3.146715529	-0.070731983	0.693396783	-0.102007948	0.918750367	0.938695719
MMADHC	31.68435194	0.029440678	0.289173347	0.101809791	0.918907654	0.938712909
MTMR9	8.830040434	0.044428626	0.436277595	0.101835681	0.918887103	0.938712909
BBOF1	4.029463511	0.061422962	0.606030137	0.101352983	0.919270258	0.938868058
SNX32	3.134570482	0.076159805	0.750517229	0.101476425	0.919172271	0.938868058
TMEM256	43.3745389	0.026612245	0.26240748	0.101415724	0.919220455	0.938868058
LOC101107957	15.04048889	-0.051147181	0.517172469	-0.098897726	0.921219474	0.940786942
SLC28A3	6.425975336	0.047717866	0.486596793	0.098064489	0.921881086	0.941390678
DAB2IP	28.43634261	-0.02772434	0.28303142	-0.09795499	0.921968034	0.941407544
FGF9	3.238137694	-0.066929101	0.685002068	-0.097706422	0.922165416	0.941537159
CCDC106	2.455328869	0.073404275	0.755815975	0.097119243	0.9226317	0.94172549
LOC101120206	5.274727533	0.054958843	0.565597723	0.097169491	0.922591796	0.94172549
SPSB2	2.519912465	0.075301799	0.77520209	0.097138281	0.922616581	0.94172549
TSHZ2	63.63407817	0.021254951	0.218786952	0.09714908	0.922608005	0.94172549
SNAPIN	11.95413079	0.043439312	0.450018769	0.096527778	0.923101414	0.942132979
HMOX2	7.587786668	-0.046039421	0.479723569	-0.095970729	0.92354382	0.942512537
LOC121819612	4.31808655	-0.067961678	0.710682691	-0.095628723	0.923815452	0.942573842
LURAP1	4.420882023	-0.05737538	0.599868852	-0.095646539	0.923801302	0.942573842
SAMD14	2.455862666	-0.080526594	0.841576153	-0.095685452	0.923770396	0.942573842
LOC101109545	5.342400858	-0.057951798	0.610041527	-0.09499648	0.924317623	0.943014224
LOC132658621	2.825944003	-0.067734941	0.716382453	-0.094551368	0.924671179	0.943158958
LOC132659193	2.342734401	0.0759784	0.802524142	0.094674286	0.924573543	0.943158958
RALGAPA1	59.41629034	-0.020502872	0.216662399	-0.094630506	0.924608319	0.943158958
PTER	1.208840254	-0.106761583	1.13337864	-0.094197631	0.924952166	0.943373572
NOCT	2.375444947	-0.077772899	0.832413389	-0.093430621	0.925561465	0.9438146
RSU1	27.27050659	-0.0249108	0.266602914	-0.09343784	0.92555573	0.9438146
SIPA1L1	11.81578261	0.034363484	0.36797011	0.093386617	0.925596422	0.9438146
MAFG	52.21667985	-0.018493379	0.198791518	-0.093029016	0.92588051	0.943960251
TNKS	12.19897001	-0.034857847	0.374692352	-0.093030579	0.925879268	0.943960251
ATP8B1	214.1448674	-0.012654081	0.136314628	-0.092829953	0.926038654	0.943963222
DNAJC17	9.437505238	-0.044544575	0.479681043	-0.092862906	0.926012475	0.943963222
SENP5	22.22885047	-0.028677643	0.30916406	-0.092758657	0.926095296	0.943963222
THUMPD1	49.83712797	-0.020776935	0.225140214	-0.092284424	0.926472063	0.944275247
MPHOSPH6	124.5358738	0.020059351	0.2183943	0.091849241	0.926817821	0.944483608
PLBD2	1.860608082	0.080308417	0.873763571	0.091910924	0.926768812	0.944483608

SRRD	4.81577905	-0.055085323	0.600448982	-0.091740221	0.92690444	0.944499866
LOC105612334	9.048763207	0.039405465	0.432173674	0.091179698	0.927349807	0.944809629
ZWINT	8.696677087	0.047797117	0.523791242	0.091252226	0.927292178	0.944809629
SC5	2.33041754	-0.070248866	0.779689264	-0.090098543	0.928208907	0.945612824
POLE	4.379971534	-0.055943933	0.623771333	-0.089686605	0.928536261	0.945874222
HINT3	19.47466428	0.028641786	0.321184297	0.089175548	0.928942398	0.946215827
SH3BP1	2.98437532	0.075683029	0.850323853	0.089004946	0.929077979	0.946281816
KDELR1	319.4876621	0.01472535	0.166334759	0.088528397	0.929456715	0.946595433
FAM168B	15.46550546	-0.028661659	0.327832929	-0.08742764	0.9303316	0.94741426
NOL4L	19.17708917	0.030033248	0.344343421	0.08721888	0.930497532	0.947511048
MBD4	23.44603038	-0.025711369	0.29543718	-0.087028211	0.930649088	0.947521002
TRAF4	71.5797756	0.022630887	0.260037338	0.087029375	0.930648163	0.947521002
EXOSC8	18.96461298	0.028765862	0.333231623	0.086323927	0.931208919	0.948018768
METTL1	6.178123958	-0.045771894	0.530920799	-0.086212282	0.931297667	0.948036909
SRSF3	223.6685827	0.012823495	0.1490094	0.086058292	0.931420079	0.948089313
LOC105612015	3.694449641	0.057918953	0.674169806	0.08591152	0.931536754	0.948135871
TTC32	29.80994386	0.022221123	0.260661488	0.085248969	0.932063461	0.948599727
PINK1	29.51251126	-0.021984203	0.25902732	-0.084872139	0.932363042	0.94878145
PNKP	3.358410282	0.056880579	0.670399497	0.084845796	0.932383985	0.94878145
KNL1	29.83603384	-0.025751217	0.304633792	-0.084531716	0.932633687	0.948963296
RMDN1	10.0684778	-0.040182936	0.476148997	-0.084391517	0.932745152	0.949004468
GON7	28.01153824	0.02237766	0.265522174	0.084277933	0.932835457	0.949024107
GRIPAP1	36.26248652	0.022324345	0.266201098	0.083862707	0.933165591	0.949287715
NAP1L3	2.726336935	0.06537578	0.781205117	0.083685806	0.933306243	0.949358542
GPBP1L1	108.5254435	-0.014335328	0.172788616	-0.082964537	0.933879738	0.949869612
KIF16B	13.28199626	0.030954355	0.37496928	0.082551709	0.934208001	0.950131193
DUS3L	5.700039459	-0.04434031	0.542049992	-0.081801146	0.934804845	0.950665872
FEZ1	3.285287741	0.057103856	0.699480678	0.081637503	0.934934978	0.950725876
NDUFA8	41.59288788	-0.024299017	0.29878561	-0.081325929	0.935182754	0.950905491
ARMC1	74.67151178	0.020244364	0.249322676	0.081197443	0.935284933	0.950937047
FHIP1A	10.98383153	0.033082325	0.408817962	0.080921898	0.935504065	0.951087498
CALCOCO1	2.012770088	-0.086167787	1.079164322	-0.079846772	0.936359125	0.951884398
ARMC5	5.817895047	-0.043845344	0.553321291	-0.079240297	0.936841493	0.952302334
FBXL16	2.089917405	-0.069916111	0.884170882	-0.079075338	0.936972699	0.952363278
LOC101102078	8.543057743	0.035040921	0.444988023	0.078745762	0.937234846	0.952485077
PTBP2	21.9369742	-0.023079019	0.29308366	-0.0787455	0.937235054	0.952485077
VWA5B2	1.613472703	-0.074355586	0.947256091	-0.078495759	0.937433704	0.952614528
HACE1	8.813332562	-0.034810236	0.444674234	-0.078282557	0.937603292	0.95271443
IFIT1	10.70191212	-0.030467838	0.391152731	-0.077892433	0.937913618	0.952957311
ZNF248	3.71109634	-0.050741356	0.656445994	-0.077297077	0.938387216	0.953366033
LPIN1	3.701302299	0.049292603	0.642660888	0.076700799	0.938861569	0.953775461
PTBP3	144.3819344	0.014244391	0.187287544	0.076056262	0.939374337	0.954223848

LOC121818057	3.065494915	0.056750595	0.751754627	0.075490848	0.939824179	0.954608251
NUDT17	11.66213646	-0.029462733	0.391886514	-0.075181798	0.940070066	0.954785449
ZDHHC5	11.65886538	0.030057308	0.400426592	0.075063218	0.940164413	0.954808719
LOC121818606	4.37245745	0.046643416	0.624904477	0.074640874	0.940500452	0.955077423
HNRNPH1	136.4623078	0.012774855	0.171784181	0.074365724	0.940719381	0.955227171
LOC121819275	9.574122347	-0.032590638	0.442607375	-0.073633292	0.94130218	0.955746349
MORC3	20.71213521	-0.022805687	0.310483112	-0.073452263	0.94144623	0.955747403
TIMM50	6.916433152	0.035879115	0.487913214	0.073535855	0.941379713	0.955747403
LOC132658129	4.128561734	-0.04526005	0.619594627	-0.073047841	0.941768048	0.956001498
SZT2	2.62907414	0.056634946	0.777425119	0.072849391	0.941925967	0.956089192
CHMP6	6.033146921	-0.03610761	0.497758235	-0.072540457	0.94217181	0.956266111
LOC114116349	2.584538053	0.05723573	0.790268181	0.072425705	0.942263129	0.956286179
CHADL	4.891288595	0.041278292	0.570958036	0.072296542	0.942365916	0.956317883
LOC132659615	20.26650896	0.020339115	0.289021787	0.070372254	0.943897375	0.957799295
LOC780455	16.71162126	0.022850235	0.328973825	0.069459127	0.944624166	0.958464025
IPO11	9.633541229	0.027474197	0.401866932	0.068366403	0.945493966	0.959200937
LCLAT1	21.8530454	-0.019918671	0.291150688	-0.068413614	0.945456385	0.959200937
B3GAT3	9.716311065	-0.028765149	0.423246085	-0.067963179	0.945814946	0.959380934
ZNF557	10.09053468	0.026764528	0.393621823	0.06799554	0.945789185	0.959380934
OGDH	18.68567061	-0.025099726	0.370054765	-0.067827059	0.945923303	0.959418035
LSMEM1	2.678487656	0.05095717	0.754338183	0.067552156	0.946142143	0.959567181
GTF2A2	1.513703672	0.068657698	1.020292644	0.067292162	0.946349117	0.959704271
LOC114109333	4.185163801	-0.04023641	0.599092961	-0.067162215	0.946452565	0.959736362
SMYD3	4.564104068	-0.040479404	0.604181141	-0.066998787	0.946582669	0.959795475
TEFM	6.476827158	0.031452386	0.471523198	0.066703794	0.946817514	0.959960774
SMU1	62.11988073	0.013428569	0.202842029	0.066202104	0.947216922	0.960292883
ABHD14A	14.20647145	-0.024920989	0.377359204	-0.066040495	0.947345585	0.960305961
SUN2	2.848482192	0.049303357	0.746959287	0.066005414	0.947373515	0.960305961
LOC105612788	6.743053957	0.032344629	0.490980796	0.065877585	0.947475286	0.960336292
AMZ2	7.630912186	0.031680212	0.484207041	0.065426995	0.947834031	0.96062706
LOC114114069	5.344812424	-0.033846809	0.528838551	-0.064002158	0.948968508	0.961703926
LOC121818816	3.262023692	0.04995056	0.782716791	0.063816901	0.949116021	0.961780495
RSBN1	43.47819855	0.014743607	0.232250981	0.063481356	0.949383205	0.961978313
LOC132657700	1.850939904	-0.061798787	0.976567076	-0.063281662	0.949542218	0.962066502
FAM241A	7.242469503	0.035573207	0.562982637	0.06318704	0.949617564	0.962069913
B4GALNT3	5.946595069	-0.032413304	0.516404279	-0.062767303	0.949951803	0.962335592
CCNB1IP1	10.30844048	0.026464922	0.42608937	0.0621112	0.950474278	0.962791906
LOC132660182	8.853737548	-0.027292902	0.44307487	-0.061598849	0.950882293	0.963116838
TGS1	91.49941126	-0.013249843	0.21534855	-0.061527433	0.950939168	0.963116838
LOC121816498	4.157123555	-0.042172585	0.688626505	-0.061241594	0.951166804	0.963201417
PARP8	7.001642914	-0.029502584	0.481680923	-0.061249227	0.951160726	0.963201417
CGN	30.84845889	0.015968799	0.263956338	0.060497881	0.951759103	0.963728195

RCBTB1	30.80323272	-0.015461908	0.257062236	-0.060148499	0.952037364	0.963936929
TOX4	134.8682774	0.011906605	0.198538883	0.059971152	0.952178611	0.964006917
LOC114118416	48.05691682	0.012617786	0.212577233	0.059356245	0.952668363	0.964429701
LOC101104297	1.621276778	0.057750874	0.975018089	0.059230567	0.952768464	0.964457989
LOC114109616	2.277452344	0.052471765	0.887632899	0.059114263	0.952861098	0.964478716
SLC31A2	5.518244497	0.031164757	0.529620875	0.05884352	0.953076744	0.964623941
ZNF287	18.0400218	0.017506283	0.299859677	0.058381586	0.953444682	0.96492327
ORAI2	7.062254061	0.028553347	0.500523747	0.057046938	0.954507805	0.965852927
USP19	8.138690257	0.026052429	0.455973478	0.057135842	0.954436985	0.965852927
LASP1	181.9755917	-0.015630017	0.276125105	-0.056604839	0.95485998	0.966131775
LOC101111388	12.99788177	-0.021751591	0.384850933	-0.056519524	0.954927943	0.966131775
KCNK1	15.80591784	-0.020772849	0.368329184	-0.056397511	0.95502514	0.96615698
MAN1C1	10.14023658	-0.024252452	0.431153819	-0.056250115	0.955142558	0.966202636
AMIGO3	2.113158913	0.05369146	0.982256246	0.054661358	0.956408255	0.96710795
BMS1	194.885857	0.010295106	0.188457783	0.05462818	0.956434688	0.96710795
DOCK9	10.94393289	0.023221153	0.423638019	0.054813667	0.956286912	0.96710795
GINS3	5.863188501	0.028062845	0.512733121	0.054731875	0.956352075	0.96710795
KCNIP2	9.744535752	0.022074071	0.401120659	0.055030999	0.956113768	0.96710795
LOC114111667	20.59587853	0.01660745	0.304267217	0.054581794	0.956471644	0.96710795
USP46	11.49227422	-0.021720963	0.398992446	-0.054439535	0.956584981	0.967149384
CMTM7	9.577789479	-0.02330492	0.429172444	-0.054301994	0.956694561	0.967187013
NAB2	3.379296322	-0.037252913	0.691712184	-0.053856089	0.957049822	0.967472993
BBS12	3.465983157	-0.034806256	0.657952969	-0.052900827	0.957810926	0.968112457
CA2	7.771062872	-0.027824082	0.526170747	-0.052880328	0.957827258	0.968112457
TNS2	5.563869608	0.026719682	0.507526123	0.052646911	0.95801324	0.968227218
LOC101109675	2.456363456	-0.04675227	0.891012635	-0.05247094	0.95815345	0.968295707
PALM3	21.6729258	0.015530911	0.303295478	0.051207196	0.959160418	0.96924005
ELP4	2.382092516	-0.041349145	0.812207419	-0.050909588	0.959397565	0.9694064
ALG13	28.01522487	-0.014268496	0.286059437	-0.049879482	0.960218428	0.970162485
EIF2A	41.46155175	-0.014366608	0.291743739	-0.049243929	0.960724904	0.970600836
SPATA9	1.819319979	-0.046184892	0.947280981	-0.048755219	0.96111437	0.970920918
LOC114109630	5.273384365	0.027434548	0.581805972	0.047154119	0.962390394	0.972136488
NCOA4	4.616090659	-0.026550379	0.567530351	-0.046782307	0.962686731	0.972359057
ZSCAN20	4.538036699	-0.026426073	0.565928087	-0.046695108	0.962756229	0.972359057
LOC114112895	5.804996696	-0.028687495	0.625489858	-0.045864044	0.963418612	0.972811848
NBDY	17.83542824	0.014010604	0.305454399	0.045868071	0.963415403	0.972811848
QSER1	97.7406623	0.007538358	0.164382393	0.045858669	0.963422897	0.972811848
ITGB5	6.271485714	0.024813	0.544579026	0.045563636	0.963658053	0.972886374
RBM8A	498.7159678	-0.010014557	0.220138347	-0.045492106	0.963715067	0.972886374
TMEM237	4.591951914	-0.032044239	0.702904615	-0.045588317	0.963638381	0.972886374
LOC101104051	25.80652754	-0.016283084	0.362963218	-0.044861527	0.964217683	0.973246757
PHKG2	5.368416581	0.024372196	0.542698209	0.044909299	0.964179604	0.973246757

AP3B2	4.708476763	-0.026325183	0.595509234	-0.044206171	0.964740063	0.973408578
C20H6orf141	32.81769356	0.010620959	0.239135437	0.044413992	0.964574409	0.973408578
HENMT1	2.641125975	0.033871874	0.764019174	0.0443338	0.964638329	0.973408578
LOC121818605	1.625714301	0.043345096	0.980579153	0.044203566	0.964742139	0.973408578
LOC132658268	1.76645824	0.040718804	0.913672846	0.044566065	0.964453192	0.973408578
DYNC1I2	456.3536646	0.008799284	0.2013969	0.043691257	0.965150508	0.973747109
HMGN4	3.319694033	-0.029549715	0.689174185	-0.042876991	0.96579959	0.974328427
TBCK	3.994499449	-0.026527769	0.620354754	-0.042762257	0.965891051	0.974347154
NIF3L1	7.79670045	0.018681493	0.438679048	0.042585787	0.966031725	0.974415519
AKT1	13.64459993	0.01549299	0.370570151	0.04180852	0.966651343	0.974769803
LOC101111099	22.04978174	0.012247831	0.293155835	0.041779249	0.966674678	0.974769803
SRCAP	33.03665582	-0.010836399	0.258709508	-0.041886357	0.966589292	0.974769803
TEDC1	6.100371039	-0.02124492	0.505308318	-0.042043479	0.966464037	0.974769803
LAMTOR1	60.37388587	0.007200145	0.178830544	0.040262388	0.96788394	0.975915566
VTI1B	85.20575133	-0.008548751	0.213895631	-0.039966926	0.968119494	0.976079442
LNX2	38.16741141	-0.009768954	0.250686732	-0.038968773	0.968915285	0.976734422
TMEM182	2.166892793	-0.033620842	0.862309268	-0.038989308	0.968898913	0.976734422
LOC132659194	1.831397311	-0.03535587	0.934737815	-0.037824371	0.969827713	0.977506765
S100PBP	25.89601925	-0.010079871	0.265866402	-0.037913293	0.969756814	0.977506765
ABCC10	2.601219534	-0.027623728	0.746998922	-0.036979609	0.970501264	0.978111891
ARAP3	16.45956068	-0.013141063	0.360281154	-0.036474467	0.970904038	0.978444045
FAM50A	4.692599909	0.022066515	0.62301453	0.035418942	0.971745681	0.979218394
RNF8	28.08954747	-0.009968335	0.283348323	-0.035180499	0.971935812	0.979336152
COMTD1	23.95832269	-0.009611774	0.276814539	-0.034722795	0.972300784	0.979408568
LOC101116303	12.29182569	-0.013743375	0.393615409	-0.034915745	0.972146926	0.979408568
TTC39B	56.80134718	-0.007216255	0.207702483	-0.034743228	0.972284491	0.979408568
ZC3H14	33.98737759	0.008666795	0.249022743	0.034803228	0.972236646	0.979408568
PBLD	26.94129581	-0.008992373	0.262840603	-0.034212269	0.972707883	0.979744805
TRAPPC13	7.070694697	-0.017091763	0.50318488	-0.033967164	0.972903335	0.97986783
THEM4	31.5819735	0.010744597	0.324993522	0.033060956	0.973625978	0.980521762
EZH1	42.6431319	0.008031967	0.243923771	0.032928186	0.973731856	0.980554508
C1GALT1	42.92160457	0.012748263	0.388361214	0.032825788	0.973813514	0.980562861
XAB2	11.41262153	-0.013211871	0.406752795	-0.032481329	0.974088205	0.980765571
LOC101115632	1.588793873	0.030698089	0.948762475	0.032355927	0.974188209	0.980792377
RAB27B	86.54650339	0.007475235	0.23671562	0.031578967	0.974807817	0.981342267
PYCR3	1.094492791	0.038303059	1.21931188	0.031413668	0.974939641	0.981354992
TRIO	87.39638139	-0.00698984	0.222755454	-0.031378985	0.9749673	0.981354992
ANKRD29	2.118461524	0.024289768	0.818752926	0.029666786	0.976332801	0.982618245
LOC101102072	58.16946688	-0.005986342	0.202098313	-0.029620941	0.976369364	0.982618245
CLCN5	4.653272436	0.016307915	0.554769992	0.029395814	0.976548912	0.982724948
PFDN2	55.553355	-0.005691252	0.19712461	-0.028871344	0.9769672	0.983071866
ABCB10	15.85857242	0.010959811	0.384090951	0.028534417	0.977235918	0.983194225

C1D	19.59566578	-0.009946815	0.348204586	-0.028566008	0.977210723	0.983194225
USP31	11.67277125	-0.011318667	0.404231213	-0.028000478	0.97766177	0.983548639
LOC101122457	20.68372542	-0.008057978	0.28947248	-0.027836769	0.97779234	0.983605962
SUPT5H	35.98640066	-0.007251262	0.265168605	-0.027345853	0.978183885	0.983851742
VSNL1	15.06390562	-0.010172908	0.3717412	-0.027365566	0.978168162	0.983851742
C1H1orf226	34.69599874	0.008545431	0.313795299	0.027232501	0.978274293	0.983868638
ERCC6	14.62635713	-0.009096116	0.339301302	-0.026808372	0.978612576	0.984134804
CPLX1	16.54994108	-0.008693803	0.327854631	-0.026517249	0.978844776	0.984195778
DHX8	10.83803266	0.010662978	0.404466355	0.026363078	0.978967744	0.984195778
RAB18	76.48966681	-0.005265251	0.199253734	-0.026424853	0.978918471	0.984195778
TOP2B	154.395131	-0.003919275	0.14819821	-0.026446166	0.978901472	0.984195778
COQ10B	31.02852624	0.005839792	0.233522451	0.025007412	0.980049052	0.985208756
LOC114118756	10.19349083	0.010372032	0.417451651	0.024846067	0.980177746	0.985264026
KCNE3	126.5294498	0.005797245	0.237598264	0.024399356	0.980534062	0.985548073
DARS1	43.78489323	0.005495854	0.226298376	0.024285877	0.980624579	0.985564939
ANP32E	205.1705734	-0.004357079	0.180530841	-0.024134817	0.980745071	0.985611927
GRIA3	2.499620587	0.018657955	0.818850345	0.02278555	0.981821335	0.986619349
SNIP1	12.65776469	-0.009014213	0.398709631	-0.022608465	0.981962591	0.986687115
EMSY	11.59021923	-0.008090558	0.363684266	-0.022246104	0.982251641	0.986903362
CTDSP1	15.27319947	0.008009336	0.373890072	0.021421633	0.982909317	0.987489922
BORCS6	33.76758656	-0.005032258	0.246007676	-0.020455695	0.983679855	0.988189774
PHKA1	8.26530061	-0.009840565	0.487475038	-0.020186809	0.983894351	0.98833097
ITPA	7.852729012	0.008807662	0.438377694	0.020091492	0.983970387	0.988333073
C1QBP	12.91423502	0.007350277	0.370366392	0.019845962	0.984166253	0.988455526
CHMP4C	13.81877747	-0.007439232	0.390494764	-0.019050786	0.984800591	0.989018311
CTDSPL2	70.07179896	0.003614846	0.197568469	0.018296673	0.985402181	0.989548126
CHST9	1.703296707	-0.016375776	0.953552909	-0.017173432	0.986298258	0.990299167
EDEM2	6.015744738	-0.008982286	0.520336621	-0.017262451	0.986227241	0.990299167
PRXL2B	14.6742156	-0.006725816	0.394764717	-0.01703753	0.986406675	0.990333631
LOC105604171	106.3806139	0.011919558	0.724245022	0.016457909	0.986869082	0.99072346
CLDN23	4.722730741	-0.00940228	0.579749578	-0.016217829	0.987060612	0.990841317
AMOTL2	15.85219028	-0.007207455	0.452363495	-0.015932884	0.987287936	0.990936944
CUEDC2	2.453825063	0.012425606	0.780867875	0.015912559	0.987304151	0.990936944
SNRPE	29.67650865	-0.004605526	0.310939751	-0.014811635	0.988182458	0.99174401
DIP2A	15.76693526	-0.005072204	0.347054613	-0.014615001	0.988339331	0.991826977
NHERF1	18.51408847	-0.005058948	0.356259099	-0.014200194	0.988670265	0.992084592
LOC105602020	1.733786138	-0.0131331	0.97886604	-0.013416647	0.989295386	0.992637349
COPB1	104.2728943	0.002162989	0.167468715	0.012915778	0.989694986	0.992963758
P4HB	249.3276821	-0.001971804	0.155668519	-0.012666685	0.989893718	0.993088601
PHC3	97.42709414	0.002201732	0.178203973	0.012355123	0.990142289	0.993263422
ARHGAP23	5.705726302	-0.007132119	0.603678361	-0.011814435	0.990573664	0.993621583
TRIM21	32.35252001	0.002760219	0.235671136	0.011712162	0.99065526	0.993628862

ZNF367	20.42669726	0.003513179	0.303928112	0.011559242	0.990777264	0.993676665
LOC101123629	1.833431948	-0.010804811	0.943836434	-0.011447758	0.99086621	0.993691309
XKR6	14.40573661	-0.004288776	0.377897118	-0.011349057	0.990944957	0.993695723
KIF11	52.46275458	0.002969963	0.26927775	0.011029368	0.991200016	0.993802372
PDCD7	18.55540978	-0.003516869	0.318320475	-0.011048202	0.99118499	0.993802372
NEB	9.38215395	-0.004267521	0.456592098	-0.009346462	0.992542711	0.995073948
EXD2	1.928386731	0.008069866	0.875168796	0.009220925	0.992642871	0.995099723
IL1A	4.312033495	-0.005565671	0.61858005	-0.008997495	0.992821134	0.995199495
PLCXD3	43.70288936	0.002632626	0.295483974	0.008909538	0.992891311	0.995199495
TIGAR	15.51841942	0.002958931	0.344116582	0.008598629	0.993139371	0.995373488
NREP	8.55673324	-0.003755047	0.449858581	-0.008347171	0.993339998	0.995499919
ZDHHC21	42.00571887	0.001649413	0.21234414	0.007767643	0.99380238	0.995888635
BOK	11.91302587	-0.00297101	0.404486225	-0.007345146	0.994139474	0.995977649
FAM120A	534.647757	-0.001033297	0.14192071	-0.007280804	0.99419081	0.995977649
MEF2B	2.920373928	-0.00589557	0.815483351	-0.007229541	0.994231711	0.995977649
TFCP2L1	20.90500222	0.002091335	0.288993733	0.007236609	0.994226071	0.995977649
TMEM192	12.23165653	-0.003005758	0.418085519	-0.007189339	0.994263787	0.995977649
LOC132659391	1.755875097	-0.006945197	1.034142986	-0.006715897	0.99464153	0.996206721
ZNF283	4.063272837	0.004250186	0.624354058	0.006807334	0.994568575	0.996206721
LOC101110376	8.431380686	-0.002799173	0.446576989	-0.006268063	0.994998842	0.996360023
LOC101117112	61.29624941	-0.001212535	0.194199158	-0.00624377	0.995018225	0.996360023
TWNK	11.12852549	0.002554797	0.403523191	0.006331227	0.994948445	0.996360023
IGSF3	14.14290392	-0.002009192	0.358784529	-0.005599997	0.995531872	0.996799685
BLM	12.20829001	-0.001971174	0.386870001	-0.005095185	0.995934649	0.997076503
SDAD1	65.08701542	-0.00099811	0.197001884	-0.005066502	0.995957534	0.997076503
UBP1	46.46434055	-0.001226238	0.257010357	-0.004771163	0.996193177	0.997237718
INTS13	20.39505274	0.001467349	0.319250396	0.004596233	0.996332749	0.997302743
RIMS3	3.136396447	0.002703719	0.675136715	0.004004698	0.996804722	0.997700458
CXCL16	8.421054192	-0.001816785	0.474618745	-0.003827883	0.996945799	0.997766945
LRP5	64.22705684	-0.000929118	0.250493046	-0.003709156	0.997040528	0.99778704
ZNF2	7.803906439	-0.001874822	0.52170735	-0.003593627	0.997132707	0.997804579
MCM10	45.15484236	0.000815782	0.252362012	0.003232587	0.997420773	0.998018121
HDDC3	20.08910375	-0.000720736	0.291552682	-0.002472061	0.998027583	0.998550541
LRP11	11.46537781	-0.000883373	0.406491657	-0.002173165	0.998266067	0.99871439
BORCS5	22.19856792	0.000554921	0.285473336	0.001943864	0.998449022	0.998822665
NFS1	7.290885223	0.000865355	0.488601559	0.001771086	0.998586879	0.998885812
ING2	26.6932981	0.000395681	0.280209526	0.001412091	0.998873315	0.999097562
PLXNA1	26.54058405	0.000269641	0.297248347	0.000907124	0.99927622	0.999425767
LOC101115005_1	6.23222084	0.000333523	0.542499306	0.000614789	0.999509469	0.999584255
DNAJC21	125.922374	-5.23731E-06	0.175984898	-2.976E-05	0.999976255	0.999976255
